# Regioselective *ortho* halogenation of *N*-aryl amides and ureas *via* oxidative halodeboronation: harnessing boron reactivity for efficient C–halogen bond installation†

**DOI:** 10.1039/d3sc04628a

**Published:** 2023-10-23

**Authors:** Ganesh H. Shinde, Ganesh S. Ghotekar, Francoise M. Amombo Noa, Lars Öhrström, Per-Ola Norrby, Henrik Sundén

**Affiliations:** a Department of Chemistry and Molecular Biology, University of Gothenburg SE-41296 Gothenburg Sweden henrik.sunden@chem.gu.se; b Department of Chemistry and Chemical Engineering, Chalmers University of Technology SE-41296 Gothenburg Sweden; c Data Science and Modelling, Pharmaceutical Sciences, R&D, AstraZeneca Gothenburg Pepparedsleden 1 Mölndal SE-43183 Sweden

## Abstract

The installation of the C–halogen bond at the *ortho* position of *N*-aryl amides and ureas represents a tool to prepare motifs that are ubiquitous in biologically active compounds. To construct such prevalent bonds, most methods require the use of precious metals and a multistep process. Here we report a novel protocol for the long-standing challenge of regioselective *ortho* halogenation of *N*-aryl amides and ureas using an oxidative halodeboronation. By harnessing the reactivity of boron over nitrogen, we merge carbonyl-directed borylation with consecutive halodeboronation, enabling the precise introduction of the C–X bond at the desired *ortho* position of *N*-aryl amides and ureas. This method offers an efficient, practical, and scalable solution for synthesizing halogenated *N*-heteroarenes under mild conditions, highlighting the superiority of boron reactivity in directing the regioselectivity of the reaction.

## Abstract 2

We report the discovery of an *ortho* regioselective oxidative halodeboronation of *N*-aryl amides and ureas with wide-ranging applications in pharmaceuticals, materials science, and synthetic organic chemistry.

## Graphical abstract

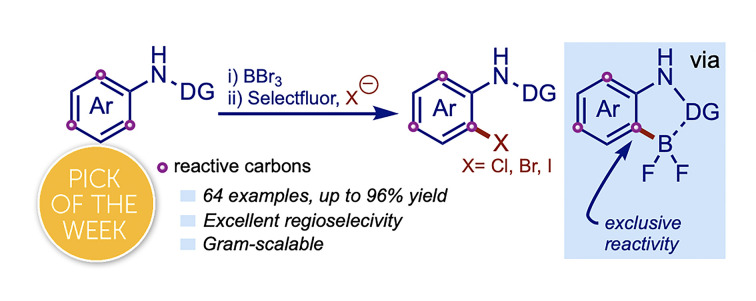

## Introduction

The amide functional group is an important structural motif found in a variety of natural products and biologically active molecules. For example, 25% of known drugs exhibit an amide motif in their complex structure and several of these substances contain the *N*-aryl amide functional group, for example, atorvastatin, aubagio, and prilocaine.^1^ Thus, transformations that are designed to cause structural modifications in the proximity of the amide functional group are of utmost importance as structural modifications, often drastically change the physiological and biological properties of pharmaceutically active molecules.^2^ In this regard, regioselective *ortho*-halogenation of *N*-aryl amides is of high interest as the halogen serves as one of the most versatile synthetic handles for functional group interconversions.^3^

**Fig. 1:**
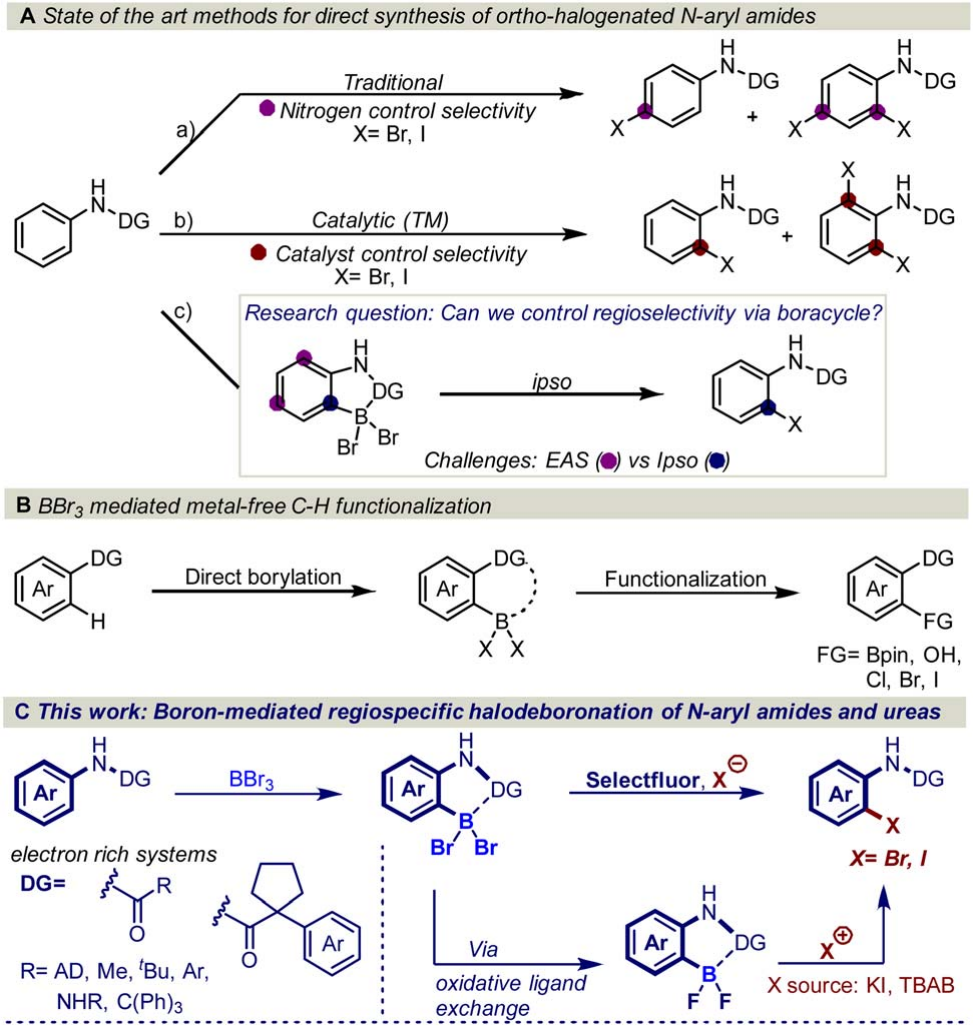
(A) Literature reports on *ortho* halogenation of *N*-aryl amides and our research question. (B) Recent reports on BBr_3_-mediated C–H borylation and functionalization. (C) This work: boron-mediated regioselective halodeboronation of *N*-aryl amides and ureas.

However, traditional electrophilic aromatic halogenation of *N*-aryl amides typically results in mixtures of *para* and *ortho*/*para* substitution (Fig. 1Aa).^4*b*,*d*,*e*^ A higher selectivity can be obtained with transition metal-catalyzed C–H functionalization (Fig. 1Ab).^4^ However, these halogenations are limited by the need for tailored activating groups and inert atmosphere conditions, which can impede synthetic efficiency.^5^ In addition, these transformations are generally restricted to *meta* and *para*-substituted aryl amides due to the formation of di-substituted halogenated side products.^4*b*–*f*^

Despite the maturity of the field *ortho* functionalization remain elusive. To address the selectivity issue, an alternative strategy would be to install boron on the aromatic ring to alter the aromatic reactivity (Fig. 1Ac) and achieve *ipso*-substitution *via* an oxidative halodeboronation at the C–B aromatic carbon instead of electrophilic aromatic substitution (EAS), providing boron control over nitrogen control. An attractive approach to installing the boron is to use the anilide amide as a handle to direct the aromatic borylation, as first developed by Murakami in 2010 (Fig. 1B). Murakami disclosed a BBr_3_-mediated synthesis of pyridine-based boracycles, which has proven to be an exceptionally attractive approach in functionalizing *N*-heteroarenes under metal-free conditions.^6^ Recently, BBr_3_-mediated borylative functionalization *via* stable boracycles has developed into a reliable synthetic route for installing hydroxyl and Bpin functionality in the *ortho*-position to a variety of directing groups (Fig. 1B).^7^ More recently, we showed that the chemistry is not limited to only hydroxyl and Bpin, but it can also be used for installing halogens to phenyl pyridines and aryl aldehydes (Fig. 1B).^8^ One of the key findings in this study is that the reaction proceeds through a difluoroboracycle, which plays a critical role in controlling the reaction's regioselectivity and preventing side reactions. However, the reaction has a limitation in that it requires an electronically deficient system. This is because electronically rich systems can lead to challenges in achieving regioselective halogenations, as competing electrophilic aromatic substitution (EAS) reactions may occur. The reactivity of electronically rich systems is highly dependent on the directing groups, making it difficult to control regioselectivity, especially with electron-rich directing groups. Inspired by the synthetic opportunities that would arise from an oxidative halodeboronation on an electronically rich system, we set out to test the capabilities of a new difluoroborane system on electronically rich *N*-aryl amides (Fig. 1C). Herein we report a convenient protocol for the unexplored regioselective *ortho*-halogenation of *N*-aryl amides and ureas. Our developed protocol shows that by using a boron handle, it is possible to control the regioselectivity of the halogenation through *ipso*-addition at the C–B carbon.

## Results and discussion

Our study was initiated by examining the sequential addition of BBr_3_ and Selectfluor to *N*-phenylpivalamide (1a) in the expectation of forming BBr_2_-complex 2a, which would undergo oxidative ligand exchange with Selectfluor to yield BF_2_-complex 4a and cationic bromine. Ultimately, this reaction pathway was expected to generate *ortho*-brominated anilide 5a*via ipso*-substitution. However, a mixture of regioisomers (5a and 5b) was obtained, which suggests that the electronically rich anilide substrate was challenging to react selectively and led to side reactions involving the radical and cationic forms of bromine promoted by Selectfluor. It is known that the combination of bromide and Selectfluor generates the cationic form of bromine, and we hypothesize that over time, the cationic form of bromine will generate bromide radicals,^9^ bromine, or HBr, which could explain the formation of the side product 5b. A potential solution to address this issue would be to avoid adding Selectfluor directly to boracycle 2a and instead prepare the electrophilic halogenating reagent by mixing Selectfluor with an anionic halogen source prior to its addition to 2a. This would create a mixture of a cationic halogen source and fluoride, both of which are necessary for the formation of aryl-BF_2_ species (4a). Our experiments showed that by premixing 1 equivalent of Selectfluor with 1.2 equivalents of potassium iodide (KI) and subsequently adding this mixture to a solution of 2a in an acetonitrile-water system at 60 °C, we were able to obtain *ortho*-iodinated product 3a exclusively with a yield of 41% (Table 1, entry 2). We attempted to further enhance the yield by increasing the loading of Selectfluor and KI, which led to the successful generation of 3a in 93% yield (Table 1, entry 4 *vs.* entries 2–3). Further, we investigated other inorganic and organic iodine sources and the result showed that KI delivered the best result (ESI, optimization Table S1,† entries 5–8). To be noted, without BBr_3_ no product formation was observed, which shows the importance of the six-member ring boron handle in this transformation (Table 1, entry 5). Next, subjecting boracycle 2a with direct electrophilic iodinating reagents provided 3a in a poor yield (Table 1, entries 6–8) and no reaction, respectively (Table 1, entry 9).

**Table 1:** Selected reaction optimization (0.22 mmol Scale)^a^

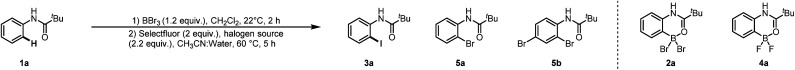						
Entry	Halogen source (equiv.)	Oxidant (equiv.)	Solvent	Time (h)	Yield 3a^c^	NMR ratio 5a:5b
1	—	Selectfluor (2)	CH3CN:Water	2	—	0.26 : 1
2	KI (1.2)	Selectfluor (1)	CH3CN:Water	5	41%	—
3	KI (1.7)	Selectfluor (1.5)	CH3CN:Water	5	66%	—
4^a^	KI (2.2)	Selectfluor (2)	CH3CN:Water	5	93%	—
5^b^	KI (2.2)	Selectfluor (2)	CH3CN:Water	5	0%	—
6	NIS (1)	—	CH3CN:Water	5	14%	—
7	ICl (1)	—	CH3CN:Water	5	12%	—
8	Barluenga's reagent (1)	—	CH3CN:Water	5	17%	—
9	I2 (2)	—	CH3CN:Water	5	0%	—

a Reaction conditions iodination: step (i) 1a (0.22 mmol), BBr_3_ (0.26 mmol), in 0.5 mL anhydrous CH_2_Cl_2_ at 22 °C, 2 h; step (ii) Selectfluor (SF) (0.44 mmol), potassium iodide (KI) (0.48 mmol) in 1.5 mL CH_3_CN and 1 mL water at 22 °C, 2 h then 60 °C for 5 h.

b Without BBr_3_.

c Isolated yields. Barluenga's reagent = bis(pyridine)iodonium(I) tetrafluoroborate.

**Scheme 1:**
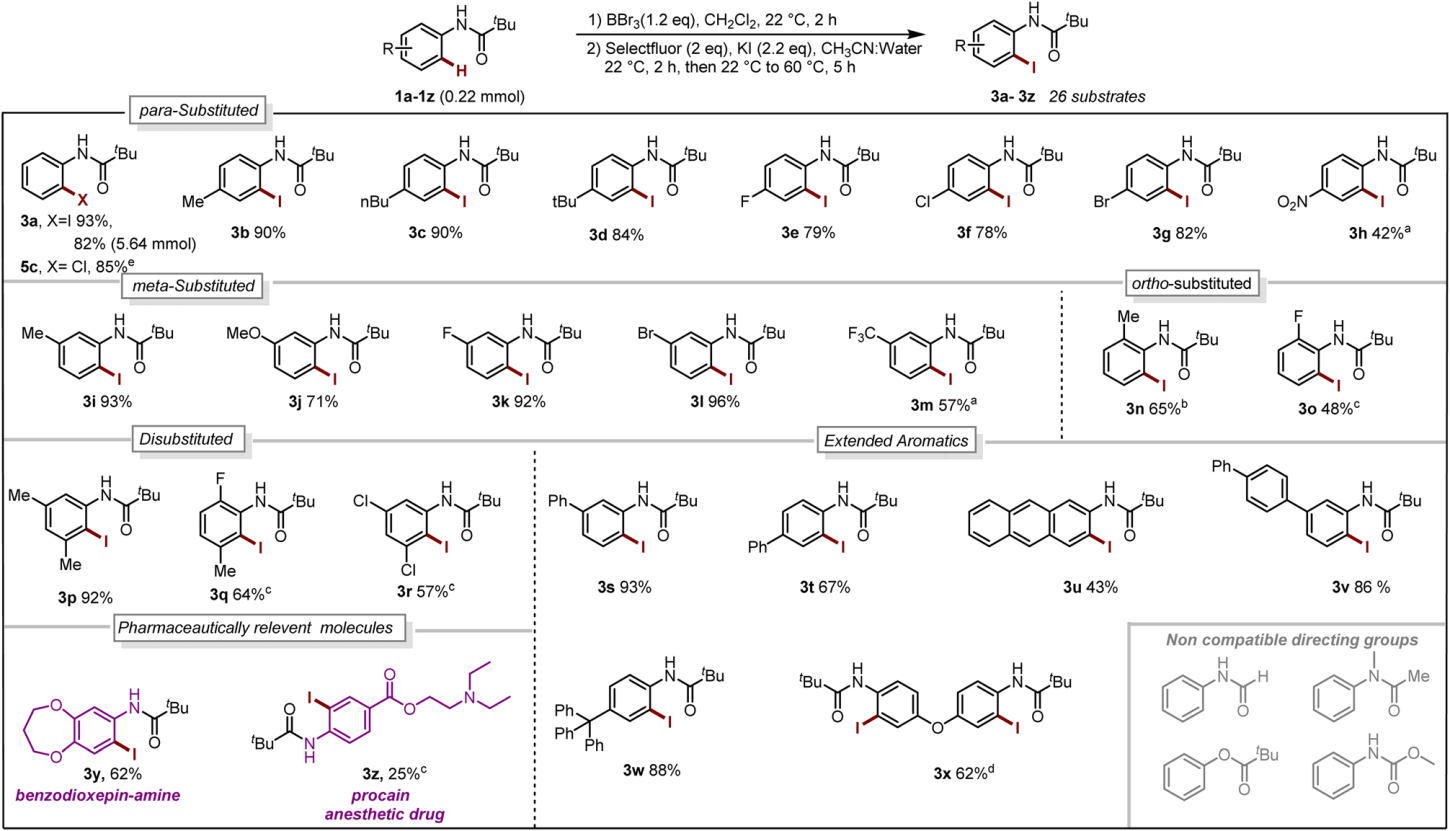
Reaction Scope. Iodination (1a–1z 0.22 mmol):^*a*^ 2.2 mmol of BBr_3._^*b*^step-1 for 16 h. ^*c*^Step-1 at 40 °C, 16 h. ^*d*^0.48 mmol of BBr_3,_ 0.88 mmol of Selectfluor, 0.92 mmol of KI. Chlorination:^e^ 0.1 mmol of 4a, 0.1 mmol of chloramine T trihydrate, CH_3_CN:Water, 70 °C, 4 h (for reaction condition see Section 4.4 in ESI†).

**Scheme 2:**
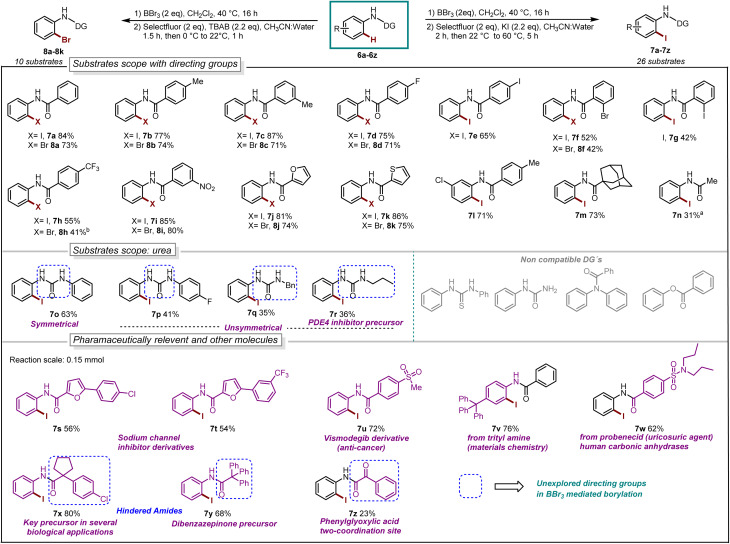
Reaction scope, iodination: ^a^BBr_3_ (0.66 mmol), 60 °C, 24 h. Bromination: ^*b*^step-2 at 40 °C, 1.5 h.

With our optimized reaction conditions in hand, the reaction scope was investigated. Several *para*-substituted *N*-aryl amides including donating groups, halogenated, and withdrawing groups, were subjected to the protocol, yielding *ortho*-iodinated products 3a–3h in moderate to excellent yields (42–93%, Scheme 1). Similarly, substitution at *meta*-position including methyl, strongly donating methoxy, halogens and withdrawing trifluoromethyl (–CF_3_) were also well tolerated providing exclusively the desired iodinated product 3i–3m in good to excellent yields (51–96%). Suggesting that the boracycle formation on the less hindered carbon is much faster than the hindered one. A sluggish reaction was observed in the case of substitution at *ortho*-position for substrates 1n and 1o. This could be explained by the steric hindrance at the *ortho*-position which potentially hinders the necessary planar conformation of the amide in the boracycle intermediate. However, by modifying the first step time, this issue could be addressed and 3n could be obtained in 65% and 3o 48%, respectively. Disubstitution on the *N*-aryl ring provided 3p–3r in good to excellent yields (57–92%). Furthermore, extended aromatics 3s–3u and substrates with a higher degree of complexity 3v–3w are also compatible with the reaction, provided the desired products in moderate to excellent yields without any side reactions on the phenyl rings. Notably, under similar conditions diiodination was performed on oxydibenzene substrate 1x giving desired product 3x in 62% yield. The reaction is scalable and can be performed on gram-scale (5.64 mmol) without much loss of efficiency and compound 3a can be obtained in 82% yield (Scheme 1). Compound 1y exhibiting an unsymmetrical *N*-aryl ring delivered the single isomer exclusively in 62% yield (3y). The biologically active procain derivative 1z with an electron-deficient system also provided desired product albeit in lower yield (3z). Next, we investigated the generality of the protocol with respect to the directing group. It was found that with slight alterations in the reaction conditions, including heating at 40 °C, the reaction was compatible with a range of directing groups, and the *ortho*-iodination exhibited excellent regioselectivity, yielding products in good to excellent yields (Scheme 2). For instance, electron-donating groups at both *para* and *meta* positions (7a–7c, Scheme 2) were well tolerated, providing the desired iodinated products in yields ranging from 77–87%. Halogen-bearing substrates on *para* and *ortho* positions also delivered iodinated products in moderate to good yields (7d–7g, 42–75%, Scheme 2). It is noteworthy to mention that these di-halogenated substrates possess high importance due to their facile cyclization for the synthesis of biologically active compounds.^12^ Electron withdrawing groups on the directing group, such as –CF_3_, –NO_2_, do not impede the reaction (7h 55%, 7i 85%, Scheme 2). Electron-rich heteroaromatics worked exceptionally well without any side reaction (7j 81%, 7k 86%, Scheme 2). Substitution on the aniline part was also well tolerated under similar conditions (7l 71%). Notably, alkyl-based directing groups such as adamantane-1-carboxamide and acetamide also provided desired products (7m 73%, 7n 31%).

It is also possible to install bromine to the *ortho*-position of anilides by using TBAB as the bromine source. The bromination worked exceptionally well for a series of substrates such as donating groups (8a–8c, 71–74%), halogens (8d 71% and 8f 42%), withdrawing groups (8h 41% and 8i 80%), heteroaromatics (8j 74% and 8k 75%).

Next, we explored urea substrates for our deborylative iodination. Urea represents a more complex substrate class in BBr_3_ chemistry due to its polarity, which gives rise to difficulties in purification. The reaction was found to proceed efficiently with a range of ureas, affording low to moderate yields of the corresponding iodinated ureas with tolerance for phenyl (7o 63%), *p*-fluorophenyl- (7p 41%), benzyl- (7q 35%) and propyl- (7r 36%) substituents. It is noteworthy to mention that this is the first approach for the halogenation as well as the borylation of *N*-aryl urea using BBr_3_. Next, we examined late-stage skeletal editing on a series of bioactive molecules and other applicative substrates.

**Scheme 3:**
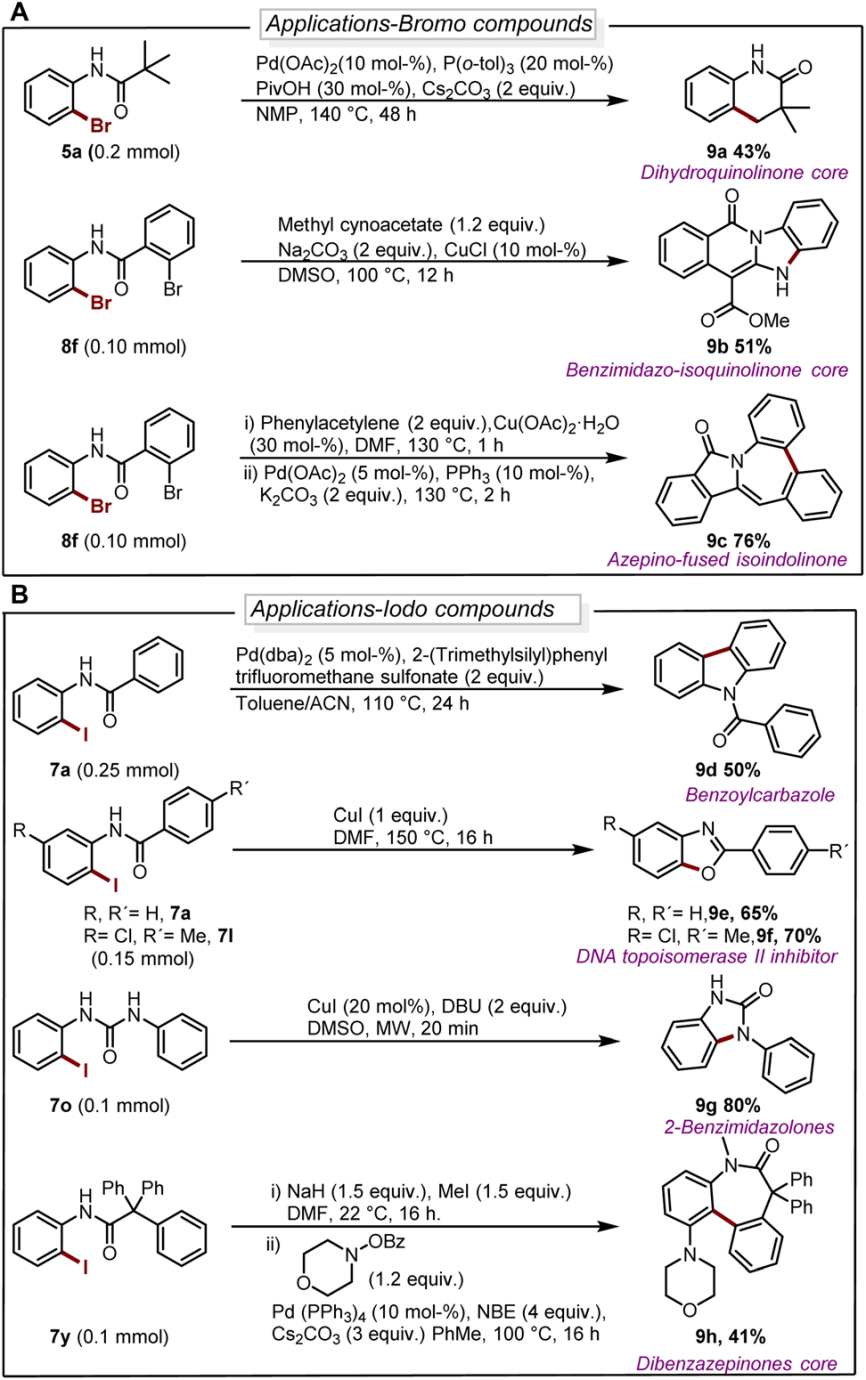
(A) Diversification of brominated substrates. For reaction details see ESI.† (B) Diversification of iodinated substrates. For reaction details see ESI.†

The deborylative iodination worked exceptionally well for sodium channel inhibitors^10*a*^ with tolerance for furan heterocycle and –CF_3_ functionality (7s 56%, 7t 54%, Scheme 2), and vismodegib derivative precursor (7u 72%), hindered *p*-trityl anilide^10*b*^ (7v 76%), probenecid derivative^10*c*^ (7w 62%) respectively. Notably, hindered amide substrates such as 6x^10*d*,*e*^ and 6y undergo smooth transformation under our condition providing valuable and challenging substrates in good to excellent yields (7x 80%, 7y 68%). Substrate with two coordinating sites such as 6z also provided desired iodination product albeit in lower yield (7z 23%). To explore the utility of deborylative halogenation we carried out a few transformations on halogenated products (Scheme 3A and B). The biologically active 3,4-dihydroquinolinone derivative 9a was synthesized from substrate 5a using a known palladium-catalyzed approach in 43% yield.^11^ Next, the benzimidazo-isoquinlinone^12*a*^9b and azepino-fused isoindolinone core^12*b*^9c were synthesized from 8f in 51% and 76% yield respectively (Scheme 3A). Further, the biologically active benzoyl carbazole^13*a*^9d and benzoxazole core^13*b*^9e–9f were synthesized using known palladium-aryne and copper chemistry from substrates 7a and 7l in 50%, 65% and 70% yield, respectively (Scheme 3B). In a copper-catalyzed reaction, iodo urea 7o was converted into 2-benzimidazolones^14^9g in 80% yield (Scheme 3B).

Finally, we demonstrated the utility of this strategy in the synthesis of the dibenzazepinones core^15^ in a reaction where 9h can be obtained from 7y in a yield of 41% (Scheme 3B).

**Scheme 4:**
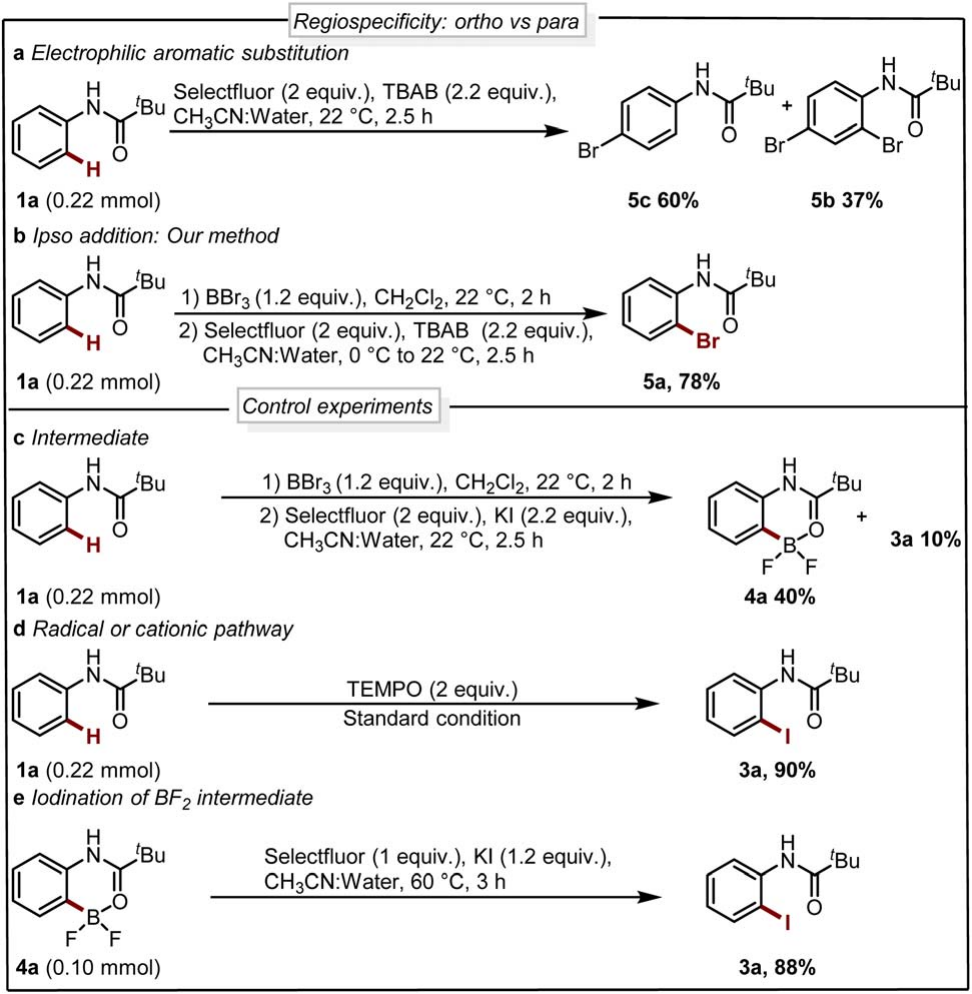
(a) Nitrogen control reaction (EAS). (b) Boron control reaction (*ipso*). (c–e) Control experiments.

Next, we analyzed the selectivity of our protocol, we conducted experiments with the oxidative halogenation conditions and the results are collected in Scheme 4a and b. In the presence of Selectfluor and bromine source tetra butyl ammonium bromide (TBAB), we observed electrophilic aromatic substitution (EAS) of 1a providing a mixture of brominated products 5b and 5c with no selectivity for the *ortho*-bromination (Scheme 4a). Further, with similar conditions in the presence of a boron handle, we observed exclusively the *ortho* bromination and 5a could be isolated in 78% yield (Scheme 4b). This result reveals that, using the boron complex the reactivity completely shifted to *ipso* rather than EAS. This experiment supports the fact that our protocol is regioselective where the reactivity of the system is governed by boron rather than nitrogen.

**Fig. 2:**
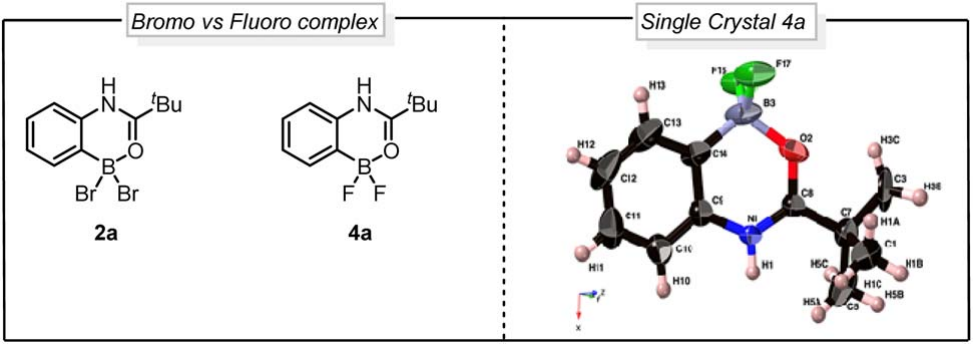
Comparison between bromo *vs.* fluoro complex and single crystal X-ray structure of 4a.

Seeking to gain mechanistic insight into the bromide-to-fluoride ion exchange behavior and C–B bond cleavage, we performed a series of experimental and computational studies. We identified two crucial steps to be investigated, *i.e.* (i) the generation of the difluoroborane species and (ii) the occurrence of ionic *ipso*-addition. To confirm the difluoroborane species 4a we quenched the reaction prematurely and isolated the 4a in 40% yield along with 10% of 3a (Scheme 4c). This result shows the formation of intermediate 4a in the reaction. The compound 4a is also confirmed by a single crystal X-ray structure (Fig. 2, ESI Section 9†). In order to get insights into the radical or ionic *ipso* addition mechanism, we conducted the reaction in the presence of radical scavenger TEMPO (Scheme 4d). However, the excess TEMPO did not affect the reaction outcome providing desired iodinated product in 90% yield. Furthermore, to reveal the reactivity and support the mechanism we subjected BF_2_ intermediate 4a to the reaction conditions of the oxidative halodeboronation (Scheme 4e) and obtained 88% yield of desired product 3a.

**Scheme 5:**
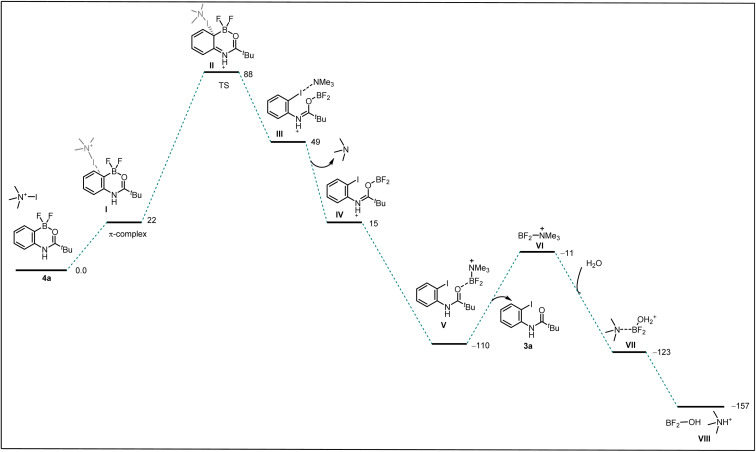
Free energy surface of *ipso*-substitution. We use a simplified model for the base, which is hypothesized to act as a carrier of electrophilic I^+^, to drive the final dissociation of boron, and to deprotonate the water adding to the boron product.

We performed a computational investigation intended to model the *ipso* addition using trimethyl amine-iodo as a model source for iodination (Scheme 5). Thus, we adopted DFT at the ωB97xD/LACVP* level with a PBF continuum model for water solvation, to evaluate the effect of dibromo-boracycle *vs.* difluoro-boracycle on *ipso* addition. The water model may slightly overestimate the stabilization provided by solvent compared to the actual solvent mix but is expected to give results superior to any other type of solvent model available to us.

We started by considering tetrahedral difluoro-boracycle 4a reacting with iodo reagent to π-complex (I) which is endergonic by 22 kJ mol^−1^. This reacts through TS(II) to complex (III), with a barrier of 88 kJ mol^−1^ compared to separated starting materials. In complex (III) boron is trigonal and has a weak interaction with π electrons of the aromatic ring. The iodine carrier, here modelled by NMe_3_, is weakly associated to the iodine by a halogen bond.

Dissociation of NMe_3_ is monotonous on the potential energy surface, but exergonic. Reassociation to boron is very strongly exergonic and drives the overall reaction. Dissociation of final product 3a is slightly endergonic, but boron can in turn associate to water, and through dissociation and final proton transfer arrive at the endpoint of the computational investigation, (VIII). Overall, formation of 3a and byproducts from 4a is exergonic by 157 kJ mol^−1^, with a barrier of 88 kJ mol^−1^. The limiting TS, (II), was located by coordinate driving of the iodine-carbon distance to shorter values. At a C–I distance of 2.2 Å, the structure is similar to the final structure of the TS, with similar energy, and could be used as a starting point for the subsequent TS search. Similar coordinate drives were performed for all positions of the aromatic ring, as well as for the *ipso*-position of the bromo-boracycle substrate 2a. In all cases, the energy rose to >150 kJ mol^−1^ without snapping into a product-like geometry, clearly showing that iodination is very strongly preferred at the *ipso* position of 4a compared to 2a.

To understand the reactivity difference between bromo boracycle 2a and fluoro-boracycle 4a (Fig. 2, single crystal X-ray structure), we analyzed the structures of both compounds, as well as the immediate product (III) from *ipso*-substitution of both complexes, where the C–B bond has been broken. In the bromo complex, the boron is electron deficient as compared to the fluoro complex. Despite the higher electronegativity of fluorine, the shorter B–F bonds, and the potential for π-donation from fluorine to the formally empty orbital of boron in (III) stabilize the boron. The BBr_2_ complex 2a has significantly shorter bonds to oxygen and carbon.

In a tetrahedral environment, the boron can compensate for the low electron density by stronger bonds to the other atoms, but in the trigonal planar complex (III), the BBr_2_ moiety lacks the stabilization which can be found from *p*-donation in the BF_2_ moiety. In other words, RBBr_2_ is a stronger Lewis acid than RBF_2_. In the tetrahedral complex, there is not much π-donation from fluorine to boron; the boron p-orbital is not empty as it is occupied with lone pair of carbonyl. Thus, the empty p-orbital can only be stabilized by π-donation from the three attached atoms, oxygen and two halides. The oxygen contribution is the same in both complexes, but the halides are very different. For instance, Br basically cannot do π-stabilization, and the orbitals of Br lone pairs are of the wrong size and distance to give efficient π overlap. On the other hand, F has a perfectly matched, filled p-orbital, which gives a very good π-stabilization.

**Scheme 6:**
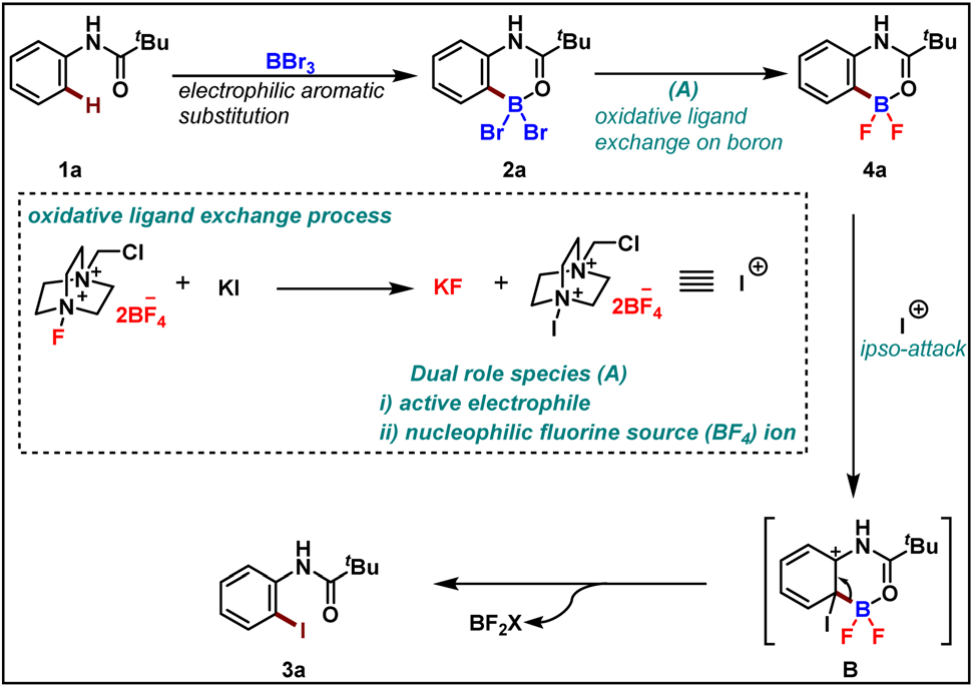
Proposed mechanism.

To rationalize the observed reactivity, based on control experiments and DFT studies we propose a mechanism (Scheme 6) that starts with the formation of boracycle 2a*via* an electrophilic aromatic substitution in the presence of boron tribromide. Next, the bromo-boracycle 2a converts into fluoro boracycle 4a in the presence of an oxidative system (A). In the classical oxidative process,^9^ Selectfluor oxidizes halogen anion X^−^ to halogen cation X^+^. However, in our case, the oxidative condition plays a dual role which is the generation of electrophile and nucleophilic fluorines for the ligand exchange on boron. In the presence of this oxidative condition, the fluoro boracycle undergoes *ipso* addition on cationic iodine to form intermediate B which upon deboryalation forms desired iodinated product 3a.

## Conclusion

In summary, we have demonstrated a regioselective, efficient, and practical *ortho* halogenation of *N*-aryl amides and ureas promoted by a boron handle. The power of this method is showcased by halogenation on a set of different carbonyls as directing groups. Our strategy leverages the dual role of Selectfluor as a fluoride source and oxidant, enabling regioselective deborylative *ortho*-halogenation of *N*-aryl amides and ureas. The proposed mechanism involves oxidative ligand exchange on boron followed by *ipso*-addition with concomitant release of the boron species. This newfound reactivity of the C–B bond and B–X bond holds promise as an alternative for the functionalization of a broad range of *N*-heterocycles in the coming years. Overall, our protocol demonstrates its applicability in the synthesis of urea substrates, pharmaceutically active molecules, and hindered amides, showcasing its strength in material science, medicinal chemistry, and synthetic organic chemistry.

## Data availability

All detailed procedures, characterization data, and spectra are available in the ESI.†

## Author contributions

H. S. supervised the overall project. G. H. S. conceived the idea and designed the study with G. S. G. F. M. A. N. and L. Ö. contributed to the crystal structure. P. O. N. contributed to the computational study. G. S. G., F. M. A. N., L. Ö. and P. O. N. provided valuable suggestions on the manuscript. H. S. and G. H. S. co-wrote the manuscript.

## Conflicts of interest

The authors declare no conflict of interest.

## Supplementary Material

SC-014-D3SC04628A-s001

SC-014-D3SC04628A-s002
