# Supplementary material for: Regioselective ortho halogenation of N-aryl amides and ureas via oxidative halodeboronation: harnessing boron reactivity for efficient C–halogen bond installation
Source: Chem Sci. 2023 Oct 23;14(46):13429–36. doi: 10.1039/d3sc04628a (PMC10685333; doi:10.1039/d3sc04628a)
Supplement: SC-014-D3SC04628A-s001 [file SC-014-D3SC04628A-s001.pdf]

Electronic Supplementary Material (ESI) for Chemical Science.
This journal is © The Royal Society of Chemistry 2023

Regioselective *Ortho* Halogenation of N-Aryl Amides and Ureas via Oxidative Halodeboronation: Harnessing Boron Reactivity for Efficient C-Halogen Bond Installation

Ganesh H. Shinde,[a†] Ganesh S. Ghotekar,[a†] Francoise M. Amombo Noa,[b] Lars Öhrström,[b] Per-Ola Norrby[c] and Henrik Sundén[ab*]

[a] Department of Chemistry and Molecular Biology, University of Gothenburg, SE-412 96 Gothenburg, Sweden.

[b] Department of Chemistry and Chemical Engineering, Chalmers University of Technology, SE-41296 Gothenburg, Sweden.

[c] Data Science and Modelling, Pharmaceutical Sciences, R&D, AstraZeneca Gothenburg, Pepparedsleden 1, Mölndal SE-43183, Sweden.

[†] Equal contributors

* Corresponding author Email: henrik.sunden@chem.gu.se

Supporting Information

Table of Contents

1. General information 3
2. Experimental section 4
3. Optimization table S1 9
4. Metal-free electrophilic deborylative halogenation of N-heteroarenes 10
5. Applications 34
6. Synthesis of difluoro boracycle (4a) 39
7. Gram scale experiment 42
8. Computational details 43
9. Single crystal X-ray diffraction 66
10. Reference 68
11. NMR spectra 70

1. General information

Experimental procedures, reagents, and glassware: All reactions were carried out in dry glassware under a nitrogen atmosphere using standard Schlenk techniques. 5 mL and 3 mL screw-top V-Vial, (sigma Aldrich, Product code-Z115150-12EA) were used for the final reactions. Acetonitrile was obtained from the SPS machine prior to use and anhydrous dichloromethane was purchased from Sigma Aldrich. All reagents were used as received from commercial suppliers unless otherwise stated. Reported yields of final compounds are calculated based on the amide starting material without consideration of dibromoboraneyl complex formation.

Chromatography: Reaction progress was monitored by thin-layer chromatography (TLC) performed on aluminum plates coated with silica gel 60 F254 (Art 5715, 0.25 mm). Chromatograms were visualized with UV light at 254 nm or by staining using potassium permanganate. Column chromatography was performed on automated column chromatography Biotage Isolera™ Spektra One with (Biotage® Sfär Silica D and HC-10g) columns. Purifications were usually done by gradient elution using binary mixtures of pentane and ethyl acetate and UV detection at 254 nm and 280 nm.

Melting Points (MP): Melting points performed on solids were recorded on a Büchi M560 and are uncorrected.

Infrared Spectroscopy: Infrared (IR) spectra were recorded on a Bruker Invenio R FTIR spectrophotometer, νmax in cm^{-1}. Samples were recorded as thin films of solids. Bands are characterised as broad (br), strong (s), medium (m), and weak (w).

Mass Spectrometry: High-resolution mass spectrometry measurements were performed by CMSI service at the Chalmers University of Technology. An Agilent 6520 equipped with an electrospray interface was operated in the positive ionization mode.

NMR Spectroscopy: All ^{1}H-NMR, ^{13}C-NMR, and ^{19}F-NMR spectra were recorded using Varian AV-400, AV-500 spectrometers and Bruker spectrometer at 300K. ^{11}B NMR was recorded on a 600 MHz Bruker spectrometer using a BBO room temperature 5 mm probe. Chemical shifts are given in parts per million (ppm, δ), referenced to the solvent peak of $CDCl_3$, defined at δ = 7.26 ppm (^{1}H-NMR) and δ = 77.16 ppm (^{13}C-NMR); $(CD_3)_2SO$ defined at δ = 2.50 ppm (^{1}H-NMR), δ = 39.52 ppm (^{13}C-NMR). Coupling constants are quoted in Hz (*J*). ^{1}H, ^{13}C, ^{19}F, and ^{11}B NMR splitting patterns are designated as singlet (s), doublet (d), triplet (t), quartet (q), bs (broad singlet) as they appeared in the spectrum. Splitting patterns that could not be interpreted or easily visualized are designated as multiplet (m). Urea starting materials[(6)] and compound 6z[(7)] were synthesized according to literature protocol.

2. Experimental section

2.1 General procedure A for the preparation of amide[1]

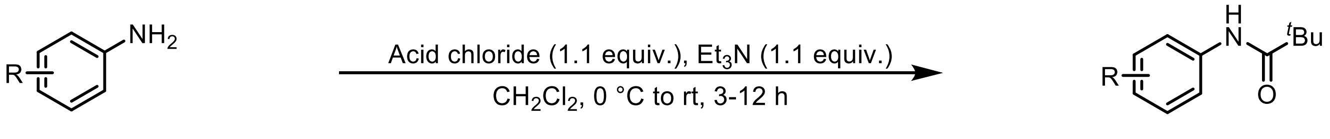

Procedure adapted from the literature.[1]

To a stirred solution of amine (3.0 mmol, 1 equiv.) and triethylamine (3.3 mmol, 1.1 equiv.) in 10 mL of anhydrous CH_2Cl_2 was added dropwise acid chloride (3,3 mmol, 1.1 equiv., in 5 mL anhydrous CH_2Cl_2) over a 5 min while maintaining the internal temperature below 5°C. Then the mixture was stirred at room temperature for 3-12 h. Completion of reaction was monitored by TLC. The mixture was diluted with additional 10 mL dichloromethane and washed with 20 mL 1M HCl followed by saturated $NaHCO_3$ (aq.) (2×20.0 mL) of and saturated NaCl (aq.), dried over sodium sulfate, and concentrated *in vacuo*. The crude product was washed with pentane to afford the analytical pure amides.

2.2 General procedure B for the preparation of Benzamide derivatives (6a-6m, 6v, 6y)

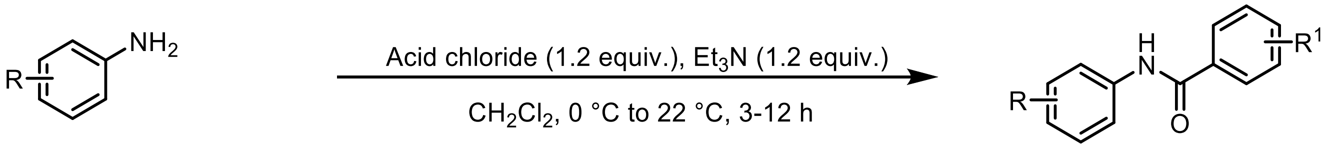

Procedure adapted from the literature.[2,3]

To a stirred solution of amine (3.0 mmol, 1 equiv.) and triethylamine (3.6 mmol, 1.2 equiv.) in 10 mL of anhydrous CH_2Cl_2 was added dropwise acid chloride (3.6 mmol, 1.2 equiv., in 5 mL anhydrous CH_2Cl_2) over a 5 min while maintaining the internal temperature below 5°C. Then the mixture was stirred at room temperature for 3-12 h. Completion of reaction was monitored by TLC. The mixture was diluted with additional 10 mL dichloromethane and washed with 20 mL water followed by saturated NH_4Cl (20 mL), 2×20.0 mL of saturated $NaHCO_3$ (aq.), and saturated NaCl (aq.), dried over sodium sulfate, and concentrated *in vacuo* to afford the analytical pure amides. A pentane wash was given if the compounds contained any impurity after the evaporation step.

2.3 General procedure C for the preparation of Benzamide derivatives (6s, 6t, 6u, 6w)

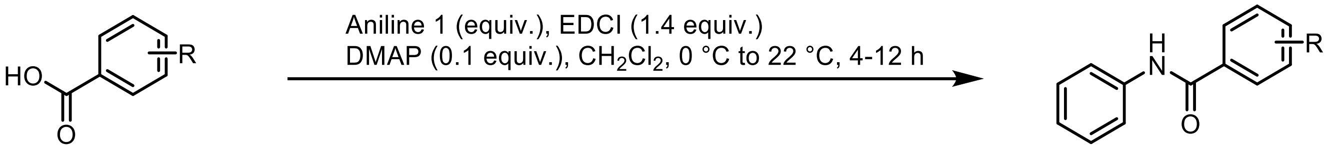

Procedure adapted from the literature.[4]

To a stirred solution of aniline (1.61 mmol, 1 equiv.) acid (1.85 mmol, 1.15 equiv.) in 15 mL of anhydrous CH_2Cl_2 at 0 ºC were added EDCI (2.25 mmol, 1.4 equiv.), DMAP (0.161 mmol, 0.1 equiv.) and the reaction mixture stirred at 22 ºC for 4-12 h. Completion of reaction was monitored by TLC. The mixture

was diluted with additional 10 mL dichloromethane and then washed with 20 mL water followed by 2×20.0 mL of saturated $NaHCO_3$ (aq.), saturated NH_4Cl (20 mL) and saturated NaCl (aq.), dried over sodium sulfate, and concentrated *in vacuo* to afford a crude gummy liquid which upon a pentane wash gives analytically pure amides.

2.4 Synthesis of N-([1,1':4',1''-terphenyl]-3-yl)pivalamide (1v)

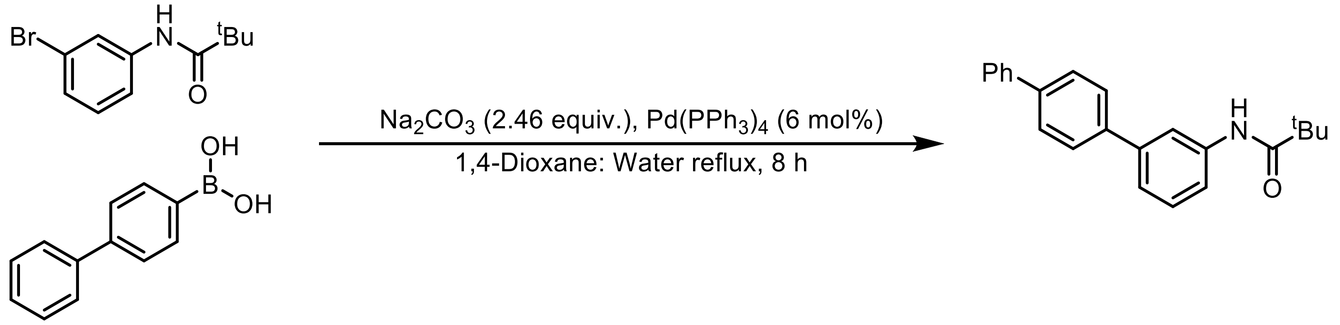

Procedure adapted from the literature.[(5)]

To a solution of N-(3-bromophenyl)pivalamide (2.0 mmol) in degassed dimethoxyethane (DME) (6 mL), was added $Pd(PPh_3)_4$ (0.06 mmol) and stirred at room temperature for 20 min. Then [1,1'-biphenyl]-4-ylboronic acid (2.4 mmol) and 2 M Na_2CO_3 solution (2 mL) were added and the reaction mixture was stirred at 80 °C under nitrogen. After completion of the reaction (TLC monitoring) DME was partially evaporated under reduced pressure, the mixture was poured on ice-water and extracted with dichloromethane (3×15 mL), separated and dried over sodium sulfate, and concentrated *in vacuo* to afford the crude product, which was purified by automated column chromatography using pentane/EtOAc.

2.5 Spectral data

***N*-(3,5-dichlorophenyl)pivalamide (1r):**

Following the general procedure A, using 3,5-dichloroaniline (486 mg, 3.0 mmol, 1 equiv.), pivaloyl chloride (3.3 mmol, 1.1 equiv.), triethyl amine (3.3 mmol, 1.1 equiv.) at room temperature for 3 h. The desired product was obtained as an off white solid (657 mg, 89%) **Rf:** 0.34 (hexane/EtOAc, 90:10); **Mp:**159-161 °C; **^{1}H NMR (400 MHz, $CDCl_3$) δ=** 7.49 (d, *J* = 1.8 Hz, 2H), 7.47 (bs, 1H), 7.06 (t, *J* = 1.8 Hz, 1H), 1.29 (s, 9H); **^{13}C{^{1}H} NMR (101 MHz, $CDCl_3$) δ=** 177.1, 139.9, 135.2, 124.2, 118.5, 39.9, 27.6; **IR (neat, cm^{-1})** = 3305 (br), 2970 (w), 2932 (br), 1661 (m), 1584 (s), 1527 (m), 1444 (m), 1404 (m), 1172 (m), 796 (m), 671 (m); **HRMS (ESI) (m/z):** calculated for $[M+H]^+$ $C_{11}H_{14}Cl_2NO^+$ 246.0452; found 246.0452.

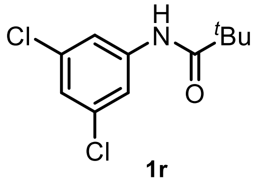

1r

***N*-([1,1':4',1''-terphenyl]-3-yl)pivalamide (1v):**

Following the general procedure 2.1.4, the desired product was obtained as an pale yellow solid (381 mg, 58%); **Rf:** 0.6 (hexane/EtOAc, 70:30); **Mp:**196-198 ºC; **^{1}H NMR (400 MHz, CDCl$_3$) δ=** 7.91 (bs, 1H),

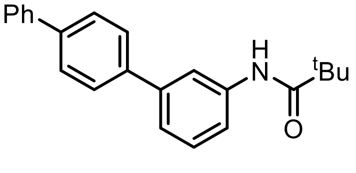

1v

7.73 – 7.63 (m, 6H), 7.52 – 7.43 (m, 4H), 7.42 – 7.35 (m, 3H), 1.36 (s, 9H); **^{13}C{^{1}H} NMR (101 MHz, CDCl$_3$) δ=** 176.9, 141.7, 140.8, 140.4, 139.7, 138.6, 129.5, 128.9, 127.7, 127.6, 127.5, 127.2, 123.0, 119.0, 118.8, 39.8, 27.8; **IR (neat, cm^{-1})** = 3345 (br), 2969 (w), 2924 (w), 1699 (s), 1538 (m), 1521 (s), 761 (w), 686 (m), 669 (s); **HRMS (ESI) (m/z):** calculated for [M+H]$^+$ $C_{23}H_{24}NO^+$ 330.1868; found 330.1858.

***N*-(4-tritylphenyl)pivalamide (1w):**

Following the general procedure A, using 4-tritylaniline (337 mg, 1.0 mmol, 1 equiv.), pivaloyl chloride (1.1 mmol, 1.1 equiv.), triethyl amine (1.1 mmol, 1.1 equiv.) at room temperature for 3 h. The desired

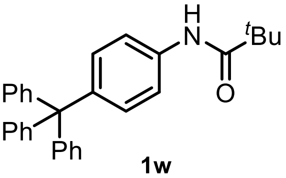

1w

product was obtained as an off white solid (392 mg, 93%); **Rf:** 0.53 (hexane/EtOAc, 80:20); **Mp:** 252-254 ºC; **^{1}H NMR (400 MHz, CDCl$_3$) δ=** 7.41 (d, *J* = 8.8 Hz, 2H), 7.30 (bs, 1H), 7.29 – 7.13 (m, 17H), 1.30 (s, 9H); **^{13}C{^{1}H} NMR (101 MHz, CDCl$_3$) δ=** 176.7, 146.9, 142.9, 135.9, 131.8, 131.2, 127.6, 126.1, 119.1, 64.7, 39.7, 27.8; **IR (neat, cm^{-1})** = 3271 (br), 2960 (w), 2922 (w), 1645 (s), 1594 (m), 1510 (s), 1404 (m), 1320 (m), 1254 (m), 1189 (m), 826 (m), 753 (m), 700 (s), 631 (w); **HRMS (ESI) (m/z):** calculated for [M+H]$^+$ $C_{30}H_{29}NO^+$ 420.2327; found 420.2329.

***N*-(3,4-dihydro-2*H*-benzo[*b*][1,4]dioxepin-7-yl)pivalamide (1y):**

Following the general procedure A, using 3,4-dihydro-2*H*-benzo[*b*][1,4]dioxepin-7-amine (496 mg, 3 mmol, 1 equiv.), pivaloyl chloride (3.3 mmol, 1.1 equiv.), triethyl amine (3.3 mmol, 1.1 equiv.) at room

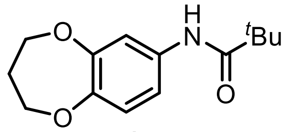

1y

temperature for 3 h. The desired product was obtained as an off white solid (718 mg, 96%); **Rf:** 0.23 (hexane/EtOAc, 80:20); **Mp:**147-149 ºC; **^{1}H NMR (400 MHz, CDCl$_3$) δ=** 7.25 (bs, 1H), 7.20 (d, *J* = 2.6 Hz, 1H), 7.04 (dd, *J* = 8.6, 2.6 Hz, 1H), 6.90 (d, *J* = 8.7 Hz, 1H), 4.15 (dt, *J* = 9.7, 5.6 Hz, 4H), 2.19 – 2.12 (m, 2H), 1.28 (s, 9H); **^{13}C{^{1}H} NMR (101 MHz, CDCl$_3$) δ=** 176.5, 151.5, 148.0, 133.5, 121.7, 115.5, 114.0, 70.9, 70.8, 39.6, 32.1, 27.7; **IR (neat, cm^{-1})** = 2963 (w), 1653 (m), 1559 (m), 1505 (s), 1457 (m), 1305 (m), 1202 (m), 1050 (m), 668 (s); **HRMS (ESI) (m/z):** calculated for [M+H]$^+$ $C_{14}H_{20}NO_3^+$ 250.1443; found 250.1445.

2-(diethylamino)ethyl 4-pivalamidobenzoate (1z):

Following the general procedure A, using 2-(diethylamino)ethyl 4-aminobenzoate hydrochloride salt (410 mg, 1.5 mmol, 1 equiv.), pivaloyl chloride (1.65 mmol, 1.1 equiv.), triethyl amine (4.5 mmol, 3 equiv.)

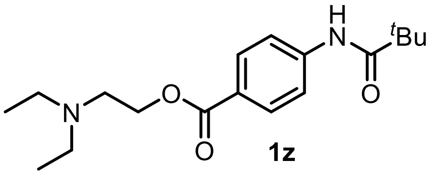

1z

at room temperature for 12 h. The desired product was obtained as an brown solid (440 mg, 91%); **Rf:** 0.10 (EtOAc, 100); **Mp:** 86-88 °C; **^{1}H NMR (400 MHz, $CDCl_3$) δ=** 7.99 (d, *J* = 8.6 Hz, 2H), 7.62 (d, *J* = 8.8 Hz, 2H), 7.48 (bs, 1H), 4.38 (t, *J* = 6.3 Hz, 2H), 2.85 (t, *J* = 6.3 Hz, 2H), 2.63 (q, *J* = 7.1 Hz, 4H), 1.32 (d, *J* = 0.6 Hz, 9H), 1.07 (t, *J* = 7.1 Hz, 6H); **^{13}C{^{1}H} NMR (101 MHz, $CDCl_3$) δ=** 177.0, 166.2, 142.4, 130.9, 125.7, 119.1, 63.3, 51.0, 47.9, 39.9, 27.6, 12.1; **IR (neat, cm^{-1})** = 3360 (br), 2968 (w), 1716 (s), 1693 (s), 1595 (m), 1518 (s), 1403 (m), 1273 (s), 1173 (m), 1111 (m), 769 (w), 696 (w); **HRMS (ESI) (m/z):** calculated for $[M+H]^+$ $C_{18}H_{29}N_2O_3^+$ 321.2178; found 321.2179.

5-(4-chlorophenyl)-*N*-phenylfuran-2-carboxamide (6s):

Following the general procedure C, using, aniline (0.15 g, 1.61 mmol, 1 equiv.) 5-(4-chlorophenyl)furan-2-carboxylic acid (1.85 mmol, 1.15 equiv.), EDCI (2.25 mmol, 1.4 equiv.), DMAP (0.161 mmol, 0.1

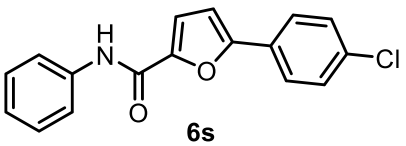

6s

equiv.) at room temperature for 4 h. The crude product was purified by automated column chromatography (pentane/EtOAc, 80:20) and the desired product was obtained as an off white solid (422 mg, 88%); **Rf:** 0.44 (hexane/EtOAc, 80:20); **Mp:**151-153 °C; **^{1}H NMR (400 MHz, $CDCl_3$) δ=** 8.07 (bs, 1H), 7.71 – 7.65 (m, 4H), 7.43 (d, *J* = 2.0 Hz, 1H), 7.42 – 7.35 (m, 3H), 7.31 (d, *J* = 3.6 Hz, 1H), 7.20 – 7.14 (m, 1H), 6.78 (d, *J* = 3.6 Hz, 1H); **^{13}C{^{1}H} NMR (101 MHz, $CDCl_3$) δ=** 156.1, 154.8, 147.2, 137.4, 134.9, 129.4, 129.3, 128.1, 125.9, 124.8, 120.2, 117.7, 108.3; **IR (neat, cm^{-1})** = 3292 (br), 2925 (w), 1653 (s), 1601 (s), 1540 (m), 1475 (s), 1441 (s), 1325 (s), 1093 (m), 1018 (m), 799 (m), 754 (m), 692 (m); **HRMS (ESI) (m/z):** calculated for $[M+H]^+$ $C_{17}H_{13}ClNO_2^+$ 298.0635; found 298.0640.

***N*-phenyl-5-(3-(trifluoromethyl)phenyl)furan-2-carboxamide (6t):**

Following the general procedure C, using, aniline (0.15 g, 1.61 mmol, 1 equiv.) 5-(3-(trifluoromethyl)phenyl)furan-2-carboxylic acid (1.85 mmol, 1.15 equiv.), EDCI (2.25 mmol, 1.4 equiv.),

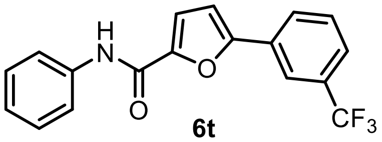

6t

DMAP (0.161 mmol, 0.1 equiv.) at room temperature for 4 h. The crude product was purified by automated column chromatography (pentane/EtOAc, 90:10) and the desired product was obtained as an pale yellow solid (351 mg, 85%); **Rf:** 0.60 (hexane/EtOAc, 80:20); **Mp:**168-170 °C; **^{1}H NMR (400 MHz, $CDCl_3$) δ=** 8.16 (bs, 1H), 7.97 (s, 1H), 7.91 (dd, *J* = 7.7, 1.7 Hz, 1H), 7.70 (dd, *J* = 8.6, 1.2 Hz, 2H), 7.59 (dtd, *J* = 15.5, 7.8, 2.0 Hz, 2H), 7.38 (ddd, *J* = 8.5, 7.4, 1.8 Hz, 2H), 7.33 (dd, *J* = 3.7, 1.9 Hz, 1H), 7.17 (tt, *J* = 7.4, 1.2 Hz, 1H), 6.87 (dd, *J* = 4.2, 1.8 Hz, 1H); **^{13}C{^{1}H} NMR (101 MHz, $CDCl_3$) δ=** 156.0,

154.2, 147.6, 137.3, 131.62 (q, J = 32.6 Hz), 130.3, 129.6, 129.3, 127.8, 125.4 (q, J = 3.8 Hz), 124.9, 124.0 (q, J=274.7) 121.4 (q, J = 3.8 Hz), 120.4, 117.6, 109.1; **^{19}F NMR (470 MHz, $CDCl_3$) δ=** -62.85; **IR (neat, cm^{-1})** = 3294 (br), 2925 (w), 1653 (m), 1601 (m), 1543 (s), 1441 (m), 1330 (s), 1167 (m), 1125 (s), 797 (m), 753 (m), 696 (m); **HRMS (ESI) (m/z):** calculated for $[M+H]^+$ $C_{18}H_{13}F_3NO_2^+$ 332.0898; found 332.0899.

N-(4-tritylphenyl)benzamide (6v):

Following the general procedure B, using 4-tritylaniline (337 mg, 1.0 mmol, 1 equiv.), triethyl amine (1.2 mmol, 1.2 equiv.) benzoyl chloride (1.2 mmol, 1.2 equiv.), at room temperature for 3 h. The crude product

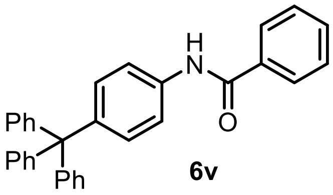

6v

was purified by automated column chromatography (pentane/EtOAc, 75:25) and the desired product was obtained as an off white solid (410 mg, 93%); **Rf:** 0.53 (hexane/EtOAc, 70:30); **Mp:** 271-273 °C; **^{1}H NMR (400 MHz, DMSO-d_6) δ=** 10.32 (bs, 1H), 7.94 (d, J = 7.0 Hz, 2H), 7.70 (d, J = 8.6 Hz, 2H), 7.57 (d, J = 7.1 Hz, 1H), 7.52 (dd, J = 8.2, 6.4 Hz, 2H), 7.31 (t, J = 7.6 Hz, 6H), 7.24 – 7.14 (m, 9H), 7.11 (d, J = 8.6 Hz, 2H); **^{13}C{^{1}H} NMR (101 MHz, DMSO-d_6) δ=** 165.6, 146.5, 141.6, 137.0, 135.0, 131.6, 130.7, 130.5, 128.4, 127.8, 127.7, 126.0, 119.6, 64.1; **IR (neat, cm^{-1})** = 3351 (br), 2921 (w), 1650 (s), 1521 (s), 1488 (m), 1321 (m), 1256 (w), 823 (w), 697 (s), 665 (s), 630 (m); **HRMS (ESI) (m/z):** calculated for $[M+H]^+$ $C_{32}H_{26}NO^+$ 440.2014; found 440.2019.

3. Optimization table S1

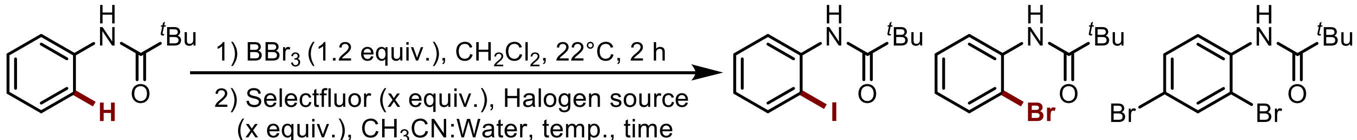

1a 3a 5a 5b

Entry	Halogen source X (equiv.)	Oxidant (equiv.)	Solvent	Time (h)	Yield[c] 3a	NMR ratio	
						**5a:5b**	
1	LiBr (2.2)	Selectlfluor (2)	CH_3CN:Water	2.5	---	1	0.14
2	NaBr (2.2)	Selectlfluor (2)	CH_3CN:Water	2.5	---	1	0.15
3	KBr (2.2)	Selectlfluor (2)	CH_3CN:Water	2.5	---	1	0.21
4	TBAB (2.2)	Selectlfluor (2)	CH_3CN:Water	2.5	---	78%[c]	trace
5	LiI (2.2)	Selectlfluor (2)	CH_3CN:Water	5	91%	-----	
6	NaI (2.2)	Selectlfluor (2)	CH_3CN:Water	5	84%	-----	
7	TBAI (2.2)	Selectlfluor (2)	CH_3CN:Water	5	91%	-----	
8	KI (2.7)	Selectlfluor (2.5)	CH_3CN:Water	5	96%	-----	

Reaction conditions for iodination: Step i) 1a (0.22 mmol), BBr_3 (0.26 mmol), in 0.5 mL anhydrous CH_2Cl_2 at 22 ºC, 2 h; Step ii) Selectfluor (SF) (0.44 mmol), potassium idodide (KI) (0.48 mmol) in 1.5 mL CH_3CN and 1 mL water at 22 ºC, 2 h then 60 ºC for 5 h. [c]Isolated yields. [d]NMR ratio

Entry 1-4: Different bromine sources were investigated and the organic source of bromine TBAB provided mono bromination however a mixture of mono and dibrominated products were observed in the case of alkali metal-based bromine sources.

Entry 5-7: Similarly organic and inorganic iodine sources were investigated and the reaction worked exceptionally well provided exclusively mono iodination. Based on yield and cost we chose potassium iodide (KI) as an iodine source.

Entry 8: Excess of Selectfluor and KI were used and it was observed that 2 equivalents of Selectfluor is required for the reaction to completion.

4. Metal-free electrophilic deborylative halogenation of N-heteroarenes

4.1 General Procedure D for reaction optimization:Iodination

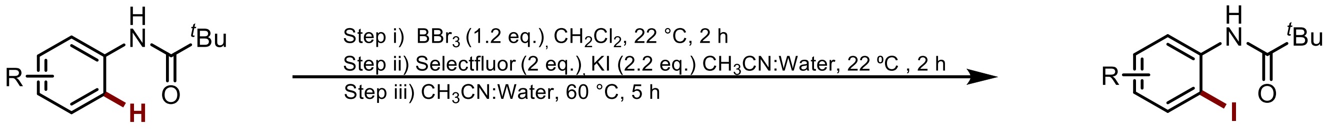

1a-1z **3a-3z**

Step i) To a dry 5 mL, screw-top V-Vial, (Sigma Aldrich, Product code-Z115150-12EA), equipped with a rubber septum, stir bar, the amide derivative (0.22 mmol, 1 equiv.) in anhydrous CH_2Cl_2 (0.5 mL) under a nitrogen atmosphere was added dropwise BBr_3 (0.26 mmol, 1.2 equiv., 1M solution in CH_2Cl_2). After the complete addition of BBr_3, the reaction mixture was stirred at 22 ºC for 2 h after which the solvent was removed under reduced pressure.

Step ii) Simultaneously, Selectfluor (0.44 mmol, 2 equiv.), and KI (0.48 mmol, 2.2 equiv.), were dissolved in CH_3CN (1 mL) and water (1 mL) in a 3 mL, screw-top V-vial and the reaction mixture stirred at 22 ºC for 2 h.

Step iii) The crude residue from step i) was dissolved in CH_3CN (0.5 mL). To this mixture, the solution from step ii) was added dropwise at 22 ºC, and the reaction mixture was heated at 60 ºC for 5 h. The reaction was quenched with saturated sodium thiosulfite solution at room temperature and the crude mixture was dissolved in EtOAc (10 mL) and H_2O (10 mL). The aqueous layer was washed with EtOAc (10 mL), and the combined organic layer was washed with brine, dried over sodium sulfate, filtered, and evaporated *in vacuo* to afford the crude product, which was purified using automated column chromatography (pentane/EtOAc).

4.2 General Procedure E for reaction optimization:Iodination of benzanilides and urea

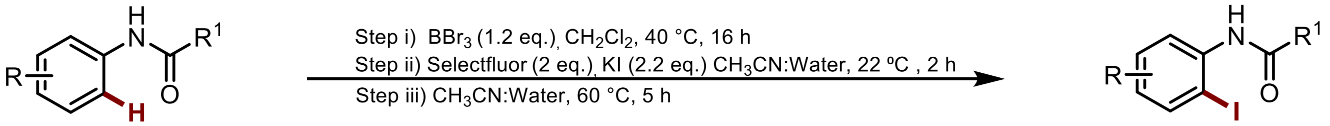

6a-6z **7a-7z**

Step i) To a dry 5 mL, screw-top V-Vial, (Sigma Aldrich, Product code-Z115150-12EA), equipped with a rubber septum, stir bar, the amide derivative/urea (0.22 mmol, 1 equiv.) in anhydrous CH_2Cl_2 (0.5 mL) under a nitrogen atmosphere was added dropwise BBr_3 (0.26 mmol, 1.2 equiv., 1M solution in CH_2Cl_2). After the complete addition of BBr_3, the reaction mixture was stirred at 40 ºC for 16 h after which the solvent was removed under reduced pressure.

Step ii) Simultaneously, Selectfluor (0.44 mmol, 2 equiv.), and KI (0.48 mmol, 2.2 equiv.), were dissolved in the CH_3CN (1 mL) and water (1 mL) in a 3 mL, screw-top V-vial and the reaction mixture stirred at 22 ºC for 2 h.

Step iii) The crude residue from step i) was dissolved in CH_3CN (0.5 mL). To this mixture, the solution from step ii) was added dropwise at 22 ºC, and the reaction mixture was heated at 60 ºC for 5 h. The reaction was quenched with saturated sodium thiosulfite solution at room temperature and the crude mixture was dissolved in EtOAc (10 mL) and H_2O (10 mL). The aqueous layer was washed with EtOAc (10 mL), and the combined organic layer was washed with brine, dried over sodium sulfate, filtered, and evaporated *in vacuo* to afford the crude product, which was purified using automated column chromatography (pentane/EtOAc).

4.3 Spectral data

***N*-(2-iodophenyl)pivalamide (3a):**[(8)]

Following the general procedure D using *N*-phenylpivalamide (39 mg, 0.22 mmol, 1 equiv.), BBr_3 (0.26 mmol, 1.2 equiv., 1M in CH_2Cl_2), at 22 ºC for 2 h and Selectfluor (0.44 mmol, 2 equiv.), KI (0.48 mmol, 2.2 equiv.) at 60 ºC for 5 h. The crude product was purified by automated column chromatography (pentane/EtOAc, 90:10) and the desired product was obtained as an off white solid (62 mg, 93%); **Rf:** 0.66 (hexane/EtOAc, 90:10); **^{1}H NMR (400 MHz, CDCl$_3$) δ=** 8.28 (d, *J* = 8.2 Hz, 1H), 7.80 (bs, 1H), 7.75 (d, *J* = 7.9 Hz, 1H), 7.33 (t, *J* = 7.8 Hz, 1H), 6.82 (t, *J* = 7.6 Hz, 1H), 1.36 (s, 9H); **^{13}C{^{1}H} NMR (101 MHz, CDCl$_3$) δ=** 176.8, 138.8, 138.4, 129.3, 125.8, 121.8, 90.2, 40.2, 27.8.

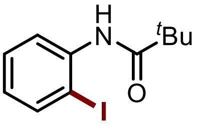

3a

***N*-(2-iodo-4-methylphenyl)pivalamide (3b):** [(9)]

Following the general procedure D using *N*-(*p*-tolyl)pivalamide (43 mg, 0.22 mmol, 1 equiv.), BBr_3 (0.26 mmol, 1.2 equiv., 1M in CH_2Cl_2), at 22 ºC for 2 h and Selectfluor (0.44 mmol, 2 equiv.), KI (0.48 mmol, 2.2 equiv.) at 60 ºC for 5 h. The crude product was purified by automated column chromatography (pentane/EtOAc, 90:10) and the desired product was obtained as an off white solid (63 mg, 90%); **Rf:** 0.63 (hexane/EtOAc, 90:10); **^{1}H NMR (400 MHz, CDCl$_3$) δ=** 8.12 (d, *J* = 8.3 Hz, 1H), 7.71 (bs, 1H), 7.60 (dd, *J* = 1.9, 0.9 Hz, 1H), 7.14 (dd, *J* = 8.4, 2.0 Hz, 1H), 2.27 (s, 3H), 1.36 (s, 9H); **^{13}C{^{1}H} NMR (101 MHz, CDCl$_3$) δ=** 176.8, 139.0, 135.9, 135.8, 130.0, 121.7, 90.3, 40.2, 27.8, 20.5.

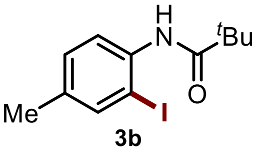

3b

***N*-(4-butyl-2-iodophenyl)pivalamide (3c):**

Following the general procedure D using *N*-(4-butylphenyl)pivalamide (51 mg, 0.22 mmol, 1 equiv.), BBr_3 (0.26 mmol, 1.2 equiv., 1M in CH_2Cl_2), at 22 ºC for 2 h and Selectfluor (0.44 mmol, 2 equiv.), KI (0.48 mmol, 2.2 equiv.) at 60 ºC for 5 h. The crude product was purified by automated column chromatography (pentane/EtOAc, 90:10) and the desired product was obtained as a colorless gummy liquid (71 mg, 90%); **Rf:** 0.73 (hexane/EtOAc, 90:10); **^{1}H NMR (400 MHz, CDCl$_3$) δ=** 8.14 (d, *J* = 8.4 Hz, 1H), 7.72 (bs, 1H), 7.59 (d, *J* = 1.9 Hz, 1H), 7.15 (dd, *J* = 8.4, 2.0 Hz, 1H), 2.52 (t, *J* = 7.7 Hz, 2H), 1.57-1.51 (m, 2H), 1.36 (s, 9H), 1.35 – 1.27 (m, 2H), 0.91 (t, *J* = 7.3 Hz, 3H); **^{13}C{^{1}H} NMR (101 MHz, CDCl$_3$)**

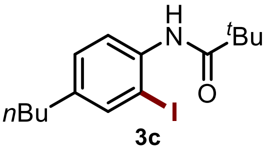

3c

δ= 176.8, 140.9, 138.4, 136.0, 129.4, 121.7, 90.4, 40.2, 34.7, 33.6, 27.8, 22.3, 14.0; **IR (neat, cm^{-1})** = 3402 (br), 2956 (w), 2927 (w), 2864 (m), 1686 (s), 1569 (m), 1508 (s), 1395 (m), 1294 (m), 1156 (m), 831 (w), 665 (w), 565 (w); **HRMS (ESI) (m/z):** calculated for $[M+H]^+$ $C_{15}H_{23}INO^+$ 360.0824; found 360.0837.

***N*-(4-(tert-butyl)-2-iodophenyl)pivalamide (3d):**

Following the general procedure D using *N*-(4-(*tert*-butyl)phenyl)pivalamide (51 mg, 0.22 mmol, 1 equiv.), BBr_3 (0.26 mmol, 1.2 equiv., 1M in CH_2Cl_2), at 22 °C for 2 h and Selectfluor (0.44 mmol, 2 equiv.), KI (0.48 mmol, 2.2 equiv.) at 60 °C for 5 h. The crude product was purified by automated column chromatography (pentane/EtOAc, 90:10) and the desired product was obtained as a colorless gummy liquid (66 mg, 84%); **Rf:** 0.73 (hexane/EtOAc, 90:10); **^{1}H NMR (400 MHz, $CDCl_3$) δ=** 8.16 (d, J = 8.6 Hz, 1H), 7.74 (d, J = 2.2 Hz, 1H), 7.72 (bs, 1H), 7.36 (dd, J = 8.5, 2.2 Hz, 1H), 1.36 (s, 9H), 1.28 (s, 9H); **^{13}C{^{1}H} NMR (101 MHz, $CDCl_3$) δ=** 176.8, 149.2, 135.9, 135.6, 126.5, 121.5, 90.6, 40.2, 34.5, 31.4, 27.9; **IR (neat, cm^{-1})** = 3402 (br), 2958 (w), 2905 (w), 2868 (w), 1689 (s), 1565 (m), 1506 (s), 1476 (s), 1386 (m), 1298 (s), 1263 (m), 1165 (m), 829 (w), 709 (w), 563 (w); **HRMS (ESI) (m/z):** calculated for $[M+H]^+$ $C_{15}H_{23}INO^+$ 360.0824; found 360.0837.

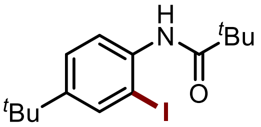

3d

***N*-(4-fluoro-2-iodophenyl)pivalamide (3e):** [10]

Following the general procedure D using *N*-(4-fluorophenyl)pivalamide (43 mg, 0.22 mmol, 1 equiv.), BBr_3 (0.26 mmol, 1.2 equiv., 1M in CH_2Cl_2), at 22 °C for 2 h and Selectfluor (0.44 mmol, 2 equiv.), KI (0.48 mmol, 2.2 equiv.) at 60 °C for 5 h. The crude product was purified by automated column chromatography (pentane/EtOAc, 90:10) and the desired product was obtained as an off white solid (56 mg, 79%); **Rf:** 0.56 (hexane/EtOAc, 90:10); **^{1}H NMR (400 MHz, $CDCl_3$) δ=** 8.19 (dd, J = 9.1, 5.5 Hz, 1H), 7.67 (bs, 1H), 7.49 (dd, J = 7.7, 2.8 Hz, 1H), 7.08 (ddd, J = 9.1, 7.8, 2.9 Hz, 1H), 1.36 (s, 9H); **^{13}C{^{1}H} NMR (101 MHz, $CDCl_3$) δ=** 176.9, 156.6 (d, J = 248.6 Hz), 134.9 (d, J = 3.1 Hz), 125.3 (d, J = 24.8 Hz), 122.8 (d, J = 7.7 Hz), 116.1 (d, J = 21.5 Hz), 89.7 (d, J = 8.3 Hz), 40.2, 27.8; **^{19}F NMR (470 MHz, $CDCl_3$) δ=** -116.61.

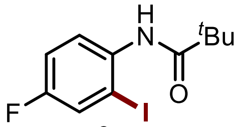

3e

***N*-(4-chloro-2-iodophenyl)pivalamide (3f):**

Following the general procedure D using *N*-(4-chlorophenyl)pivalamide (47 mg, 0.22 mmol, 1 equiv.), BBr_3 (0.26 mmol, 1.2 equiv., 1M in CH_2Cl_2), at 22 °C for 2 h and Selectfluor (0.44 mmol, 2 equiv.), KI (0.48 mmol, 2.2 equiv.) at 60 °C for 5 h. The crude product was purified by automated column chromatography (pentane/EtOAc, 90:10) and the desired product was obtained as an off white solid (61 mg, 82%); **Rf:** 0.70 (hexane/EtOAc, 90:10); **Mp:** 73-75 °C; **^{1}H NMR (400 MHz, $CDCl_3$) δ=** 8.25 (d, J = 8.9 Hz, 1H), 7.77 (bs, 1H), 7.75 (d, J = 2.4 Hz, 1H), 7.32 (dd, J = 8.8, 2.4 Hz, 1H), 1.36 (s, 9H); **^{13}C{^{1}H} NMR (101**

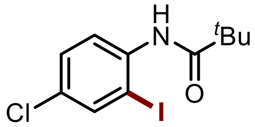

3f

MHz, $CDCl_3$) δ= 176.9, 137.8, 137.3, 129.6, 129.4, 122.1, 89.8, 40.3, 27.8; **IR (neat, cm^{-1})** = 3400 (br), 3327 (w), 2961 (w), 1687 (s), 1571 (m), 1501 (s), 1375 (m), 1292 (m), 1156 (w), 1031 (w), 865 (w), 821 (w), 710 (w), 540 (w); **HRMS (ESI) (m/z):** calculated for $[M+H]^+$ $C_{11}H_{14}ClINO^+$ 337.9809; found 337.9809.

***N*-(4-bromo-2-iodophenyl)pivalamide (3g):**

Following the general procedure D using *N*-(4-bromophenyl)pivalamide (56 mg, 0.22 mmol, 1 equiv.), BBr_3 (0.26 mmol, 1.2 equiv., 1M in CH_2Cl_2), at 22 °C for 2 h and Selectfluor (0.44 mmol, 2 equiv.), KI

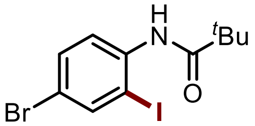

3g

(0.48 mmol, 2.2 equiv.) at 60 °C for 5 h. The crude product was purified by automated column chromatography (pentane/EtOAc, 90:10) and the desired product was obtained as an off white solid (69 mg, 82%); **Rf:** 0.70 (hexane/EtOAc, 90:10); **Mp:** 72-74 °C; **^{1}H NMR (400 MHz, $CDCl_3$) δ=** 8.21 (d, *J* = 8.8 Hz, 1H), 7.89 (d, *J* = 2.2 Hz, 1H), 7.77 (bs, 1H), 7.45 (dd, *J* = 8.9, 2.2 Hz, 1H), 1.36 (s, 9H); **^{13}C{^{1}H} NMR (101 MHz, $CDCl_3$) δ=** 176.9, 140.5, 137.7, 132.3, 122.5, 117.0, 90.2, 40.4, 27.8; **IR (neat, cm^{-1})** = 3399 (br), 2961 (w), 2928 (w), 1690 (m), 1589 (m), 1503 (s), 1370 (m), 1291 (m), 1156 (m), 819 (w), 672 (w), 535 (w); **HRMS (ESI) (m/z):** calculated for $[M+H]^+$ $C_{11}H_{14}BrINO^+$ 381.9303; found 381.9305.

***N*-(2-iodo-4-nitrophenyl)pivalamide (3h):** [8]

Following the general procedure D using *N*-(4-nitrophenyl)pivalamide (49 mg, 0.22 mmol, 1 equiv.), BBr_3 (2.64 mmol, 12 equiv., 1M in CH_2Cl_2), at 22 °C for 2 h and Selectfluor (0.44 mmol, 2 equiv.), KI

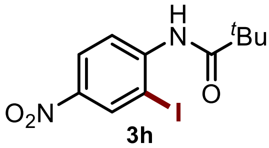

3h

(0.48 mmol, 2.2 equiv.) at 60 °C for 5 h. The crude product was purified by automated column chromatography (pentane/EtOAc, 90:10) and the desired product was obtained as a yellow solid (32 mg, 42%); **Rf:** 0.43 (hexane/EtOAc, 90:10); **^{1}H NMR (400 MHz, $CDCl_3$) δ=** 8.66 (d, *J* = 2.6 Hz, 1H), 8.60 (d, *J* = 9.2 Hz, 1H), 8.23 (dd, *J* = 9.2, 2.6 Hz, 1H), 8.14 (bs, 1H), 1.39 (s, 9H); **^{13}C{^{1}H} NMR (101 MHz, $CDCl_3$) δ=** 177.3, 144.1, 143.4, 134.2, 125.1, 119.6, 87.8, 40.8, 27.7.

***N*-(2-iodo-5-methylphenyl)pivalamide (3i):**

Following the general procedure D using *N*-(*m*-tolyl)pivalamide (43 mg, 0.22 mmol, 1 equiv.), BBr_3 (0.26 mmol, 1.2 equiv., 1M in CH_2Cl_2), at 22 °C for 2 h and Selectfluor (0.44 mmol, 2 equiv.), KI (0.48 mmol,

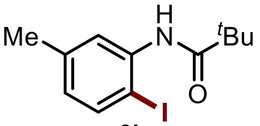

3i

2.2 equiv.) at 60 °C for 5 h. The crude product was purified by automated column chromatography (pentane/EtOAc, 90:10) and the desired product was obtained as an off white solid (65 mg, 93%); **Rf:** 0.66 (hexane/EtOAc, 90:10); **Mp:** 76-78 °C; **^{1}H NMR (400 MHz, $CDCl_3$) δ=** 8.16 (d, *J* = 2.1 Hz, 1H), 7.76 (bs, 1H), 7.62 (d, *J* = 8.1 Hz, 1H), 6.67 (dd, *J* = 8.2, 2.2 Hz, 1H), 2.32 (s, 3H), 1.36 (s, 9H); **^{13}C{^{1}H} NMR (101 MHz, $CDCl_3$) δ=** 177.0, 139.8, 138.3, 138.1, 126.9, 122.5, 86.1, 40.3, 27.8, 21.4; **IR (neat, cm^{-1})** = 3301 (br), 2957 (m), 2924 (m), 2866 (m), 1691 (s), 1574 (m), 1520 (s), 1407 (s), 1289 (m), 1184 (m), 1151 (w),

1102 (w), 801 (w), 668 (w), 577 (w); **HRMS (ESI) (m/z):** calculated for $[M+H]^+$ $C_{12}H_{17}INO^+$ 318.0355; found 318.037.

***N*-(2-iodo-5-methoxyphenyl)pivalamide (3j):**

Following the general procedure D using *N*-(3-methoxyphenyl)pivalamide (46 mg, 0.22 mmol, 1 equiv.), BBr_3 (0.53 mmol, 2.4 equiv., 1M in CH_2Cl_2), at 22 ºC for 2 h and Selectfluor (0.44 mmol, 2 equiv.), KI (0.48 mmol, 2.2 equiv.) at 60 ºC for 5 h. The crude product was purified by automated column chromatography (pentane/EtOAc, 90:10) and the desired product was obtained as a colorless gummy liquid (52 mg, 71%); **Rf:** 0.43 (hexane/EtOAc, 90:10); **^{1}H NMR (400 MHz, $CDCl_3$) δ=** 8.10 (d, *J* = 3.0 Hz, 1H), 7.84 (bs, 1H), 7.59 (d, *J* = 8.8 Hz, 1H), 6.47 (dd, *J* = 8.8, 3.0 Hz, 1H), 3.81 (s, 3H), 1.37 (s, 9H); **$^{13}C\{^1H\}$ NMR (101 MHz, $CDCl_3$) δ=** 177.2, 160.8, 139.2, 138.6, 113.2, 106.4, 77.9, 55.6, 40.5, 27.8; **IR (neat, cm^{-1})** = 3398 (br), 2960 (w), 1690 (s), 1580 (s), 1519 (s), 1452 (m), 1414 (m), 1301 (m), 1206 (s), 1175 (m), 1050 (w), 1008 (w), 859 (w), 590 (w); **HRMS (ESI) (m/z):** calculated for $[M+H]^+$ $C_{12}H_{17}INO_2^+$ 334.0304; found 334.0317.

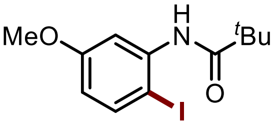

3j

***N*-(5-fluoro-2-iodophenyl)pivalamide (3k):**

Following the general procedure D using *N*-(3-fluorophenyl)pivalamide (43 mg, 0.22 mmol, 1 equiv.), BBr_3 (0.26 mmol, 1.2 equiv., 1M in CH_2Cl_2), at 22 ºC for 2 h and Selectfluor (0.44 mmol, 2 equiv.), KI (0.48 mmol, 2.2 equiv.) at 60 ºC for 5 h. The crude product was purified by automated column chromatography (pentane/EtOAc, 90:10) and the desired product was obtained as a off white solid (65 mg, 92%); **Rf:** 0.73 (hexane/EtOAc, 90:10); **Mp:** 47-49 ºC; **^{1}H NMR (400 MHz, $CDCl_3$) δ=** 8.23 (dd, *J* = 11.2, 3.0 Hz, 1H), 7.87 (bs, 1H), 7.69 (ddd, *J* = 8.8, 6.0, 1.1 Hz, 1H), 6.62 (tdd, *J* = 8.7, 3.0, 1.1 Hz, 1H), 1.36 (s, 9H); **$^{13}C\{^1H\}$ NMR (101 MHz, $CDCl_3$) δ=** 177.1, 163.4 (d, *J* = 246.3 Hz), 139.7 (d, *J* = 11.9 Hz), 139.1 (d, *J* = 9.0 Hz), 112.8 (d, *J* = 22.6 Hz), 109.1 (d, *J* = 28.9 Hz), 82.21, 40.4, 27.8; **^{19}F NMR (470 MHz, $CDCl_3$) δ=** -110.59; **IR (neat, cm^{-1})** = 3398 (br), 2959 (w), 2928 (w), 1688 (m), 1596 (m), 1561 (s), 1491 (s), 1379 (m), 1294 (s), 1157 (s), 833 (w), 761 (s), 696 (m), 665 (w), 571 (w); **HRMS (ESI) (m/z):** calculated for $[M+H]^+$ $C_{11}H_{14}FINO^+$ 322.0104; found 322.0119.

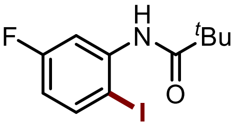

3k

***N*-(5-bromo-2-iodophenyl)pivalamide (3l):**

Following the general procedure D using *N*-(3-bromophenyl)pivalamide (56 mg, 0.22 mmol, 1 equiv.), BBr_3 (0.26 mmol, 1.2 equiv., 1M in CH_2Cl_2), at 22 ºC for 2 h and Selectfluor (0.44 mmol, 2 equiv.), KI (0.48 mmol, 2.2 equiv.) at 60 ºC for 5 h. The crude product was purified by automated column chromatography (pentane/EtOAc, 90:10) and the desired product was obtained as an off white solid (81 mg, 96%); **Rf:** 0.73 (hexane/EtOAc, 90:10); **Mp:** 91-93 ºC; **^{1}H NMR (400 MHz, $CDCl_3$) δ=** 8.57 (d, *J* = 2.3 Hz, 1H),

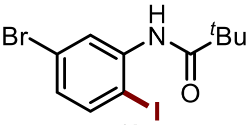

3l

7.80 (bs, 1H), 7.60 (d, J = 8.4 Hz, 1H), 6.97 (dd, J = 8.4, 2.3 Hz, 1H), 1.36 (s, 9H); **^{13}C{^{1}H} NMR (101 MHz, CDCl$_3$) δ=** 177.0, 139.5, 128.7, 124.4, 123.5, 87.6, 40.4, 27.8.

***N*-(2-iodo-5-(trifluoromethyl)phenyl)pivalamide (3m):**

Following the general procedure D using *N*-(3-(trifluoromethyl)phenyl)pivalamide (54 mg, 0.22 mmol, 1 equiv.), BBr$_3$ (2.2 mmol, 10 equiv., 1M in CH$_2$Cl$_2$), at 22 ºC for 2 h and Selectfluor (0.44 mmol, 2 equiv.), KI (0.48 mmol, 2.2 equiv.) at 60 ºC for 5 h. The crude product was purified by automated column chromatography (pentane/EtOAc, 90:10) and the desired product was obtained as an off white solid (47 mg, 57%); **Rf:** 0.43 (hexane/EtOAc, 90:10); **Mp:** 70-72 ºC; **^{1}H NMR (400 MHz, CDCl$_3$) δ=** 8.67 (d, J = 2.2 Hz, 1H), 7.93 (s, 1H), 7.89 (d, J = 8.2 Hz, 1H), 7.08 (dd, J = 8.3, 1.5 Hz, 1H), 1.38 (s, 9H); **^{13}C{^{1}H} NMR (101 MHz, CDCl$_3$) δ=** 177.2, 139.3, 139.1, 132.0 (q, J = 32.9 Hz), 123.7 (q, J = 272.7 Hz), 121.9 (q, J = 3.8 Hz), 118.1 (q, J = 4.1 Hz), 93.6, 40.4, 27.7; **^{19}F NMR (470 MHz, CDCl$_3$) δ=** -63.03; **IR (neat, cm^{-1})** = 3398 (br), 2960 (w), 2925 (w), 1692 (m), 1578 (w), 1520 (s), 1417 (s), 1328 (s), 1260 (m), 1126 (s), 1079 (s), 1015 (s), 817 (m), 752 (w), 568 (w); **HRMS (ESI) (m/z):** calculated for [M+H]$^+$ C$_{12}$H$_{14}$F$_3$INO$^+$ 372.0072; found 372.0086.

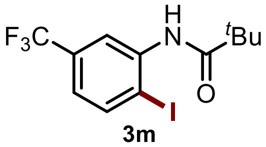

3m

***N*-(2-iodo-6-methylphenyl)pivalamide (3n):**

Following the general procedure D using *N*-(*o*-tolyl)pivalamide (43 mg, 0.22 mmol, 1 equiv.), BBr$_3$ (0.26 mmol, 1.2 equiv., 1M in CH$_2$Cl$_2$), at 22 ºC for 16 h and Selectfluor (0.44 mmol, 2 equiv.), KI (0.48 mmol, 2.2 equiv.) at 60 ºC for 5 h. The crude product was purified by automated column chromatography (pentane/EtOAc, 90:10) and the desired product was obtained as an off white solid (45 mg, 64%); **Rf:** 0.26 (hexane/EtOAc, 90:10); **Mp:** 189-191 ºC; **^{1}H NMR (400 MHz, CDCl$_3$) δ=** 7.67 (dd, J = 8.0, 0.8 Hz, 1H), 7.20 (d, J = 7.6 Hz, 1H), 7.10 (bs, 1H), 6.89 (t, J = 7.8 Hz, 1H), 2.25 (s, 3H), 1.38 (s, 9H); **^{13}C{^{1}H} NMR (101 MHz, CDCl$_3$) δ=** 176.2, 137.9, 137.1, 136.7, 131.1, 128.8, 99.32, 39.5, 27.9, 19.7; **IR (neat, cm^{-1})** = 3267 (br), 2958 (m), 2924 (m), 1650 (s), 1504 (s), 1229 (w), 1177 (m), 936 (w), 762 (m), 709 (w), 642 (w); **HRMS (ESI) (m/z):** calculated for [M+H]$^+$ C$_{12}$H$_{17}$INO$^+$ 318.0355; found 318.0369.

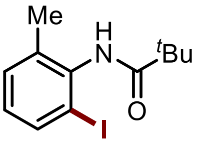

3n

***N*-(2-fluoro-6-iodophenyl)pivalamide (3o):**

Following the general procedure D using *N*-(2-fluorophenyl)pivalamide (43 mg, 0.22 mmol, 1 equiv.), BBr$_3$ (0.26 mmol, 1.2 equiv., 1M in CH$_2$Cl$_2$), at 40 ºC for 16 h and Selectfluor (0.44 mmol, 2 equiv.), KI (0.48 mmol, 2.2 equiv.) at 60 ºC for 5 h. The crude product was purified by automated column chromatography (pentane/EtOAc, 90:10) and the desired product was obtained as an off white solid (34 mg, 48%); **Rf:** 0.20 (hexane/EtOAc, 90:10); **Mp:**159-161 ºC; **^{1}H NMR (400 MHz, CDCl$_3$) δ=** 7.61 (dt, J = 8.0, 1.1 Hz, 1H), 7.12 (tt, J = 8.3, 1.2 Hz, 1H), 7.02 – 6.94 (m, 2H), 1.37 (s, 9H); **^{13}C{^{1}H} NMR (101 MHz, CDCl$_3$) δ=** 176.5, 157.4 (d, J = 254.8

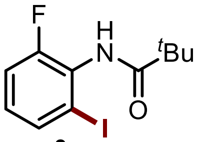

3o

Hz), 134.3 (d, J = 3.7 Hz), 127.6, 129.6 (d, J = 8.4 Hz), 116.74 (d, J = 21.2 Hz)., 98.7, 39.5, 27.7; **^{19}F NMR (470 MHz, CDCl$_3$) δ=** -112.48; **IR (neat, cm^{-1})** = 3271 (br), 2973 (w), 2927 (w), 1659 (s), 1506 (s), 1472 (m), 1265(m), 1239 (w), 872 (w), 842 (w), 779 (m), 707 (w), 668 (w); **HRMS (ESI) (m/z):** calculated for $[M+H]^+$ $C_{11}H_{14}FINO^+$ 322.0104; found 322.0111.

***N*-(2-iodo-3,5-dimethylphenyl)pivalamide (3p):**

Following the general procedure D using *N*-(3,5-dimethylphenyl)pivalamide (45 mg, 0.22 mmol, 1 equiv.), BBr_3 (0.26 mmol, 1.2 equiv., 1M in CH_2Cl_2), at 22 °C for 2 h and Selectfluor (0.44 mmol, 2 equiv.), KI (0.48 mmol, 2.2 equiv.) at 60 °C for 5 h. The crude product was purified by automated column chromatography (pentane/EtOAc, 90:10) and the desired product was obtained as an off white solid (67 mg, 92%); **Rf:** 0.66 (hexane/EtOAc, 90:10); **Mp:** 49-51 °C; **^{1}H NMR (400 MHz, CDCl$_3$) δ=** 7.95 (d, J = 2.1 Hz, 1H), 7.93 (bs, 1H), 6.85 (d, J = 2.2 Hz, 1H), 2.43 (s, 3H), 2.29 (s, 3H), 1.37 (s, 9H); **^{13}C{^{1}H} NMR (101 MHz, CDCl$_3$) δ=** 177.0, 141.8, 138.9, 138.2, 126.7, 119.9, 93.8, 40.4, 29.5, 27.9, 21.2; **IR (neat, cm^{-1})** = 3397 (br), 2959 (w), 1692 (s), 1521 (w), 1456 (s), 1417 (w), 1182 (w), 668 (s), 582 (w); **HRMS (ESI) (m/z):** calculated for $[M+H]^+$ $C_{13}H_{19}INO^+$ 332.0511; found 332.0527.

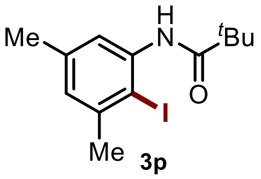
3p

***N*-(6-fluoro-2-iodo-3-methylphenyl)pivalamide (3q):**

Following the general procedure D using *N*-(2-fluoro-5-methylphenyl)pivalamide (46 mg, 0.22 mmol, 1 equiv.), BBr_3 (0.26 mmol, 1.2 equiv., 1M in CH_2Cl_2), at 40 °C for 16 h and Selectfluor (0.44 mmol, 2 equiv.), KI (0.48 mmol, 2.2 equiv.) at 60 °C for 5 h. The crude product was purified by automated column chromatography (pentane/EtOAc, 90:10) and the desired product was obtained as an off white solid (47 mg, 64%); **Rf:** 0.23 (hexane/EtOAc, 90:10); **Mp:** 189-191 °C; **^{1}H NMR (400 MHz, CDCl$_3$) δ=** 7.14 (dd, J = 8.5, 5.4 Hz, 1H), 7.04 (d, J = 8.8 Hz, 1H), 7.01 (bs, 1H), 2.45 (s, 3H), 1.38 (s, 9H); **^{13}C{^{1}H} NMR (101 MHz, CDCl$_3$) δ=** 176.5, 155.3 (d, J = 252.1 Hz), 138.2 (d, J = 3.6 Hz), 128.5 (d, J = 7.8 Hz), 127.12 (d, J = 14.5 Hz), 115.7 (d, J = 20.7 Hz), 105.5, 39.5, 28.6, 27.8; **^{19}F NMR (470 MHz, CDCl$_3$) δ=** -116.59; **IR (neat, cm^{-1})** = 3317 (br), 2980 (w), 2956 (w), 2920 (w), 1654 (s), 1500 (s), 1478 (s), 1285 (w), 1185 (w), 1157 (m), 838 (w), 816 (m), 573 (w); **HRMS (ESI) (m/z):** calculated for $[M+H]^+$ $C_{12}H_{16}FINO^+$ 336.0261; found 336.0271.

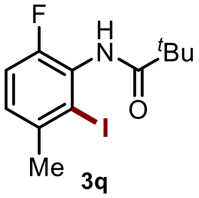
3q

***N*-(3,5-dichloro-2-iodophenyl)pivalamide (3r):**

Following the general procedure D using *N*-(3,5-dichlorophenyl)pivalamide (54 mg, 0.22 mmol, 1 equiv.), BBr_3 (0.26 mmol, 1.2 equiv., 1M in CH_2Cl_2), at 40 °C for 16 h and Selectfluor (0.44 mmol, 2 equiv.), KI (0.48 mmol, 2.2 equiv.) at 60 °C for 5 h. The crude product was purified by automated column chromatography (pentane/EtOAc, 90:10) and the desired product was obtained as an off white solid (47 mg, 57%); **Rf:** 0.53 (hexane/EtOAc, 90:10);

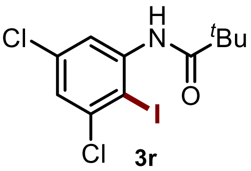
3r

Mp: 99-101 ºC; **^{1}H NMR (400 MHz, CDCl$_3$) δ=** 8.36 (d, *J* = 2.3 Hz, 1H), 8.04 (bs, 1H), 7.24 (d, *J* = 2.3 Hz, 1H), 1.36 (s, 9H); **^{13}C{^{1}H} NMR (101 MHz, CDCl$_3$) δ=** 177.2, 141.1, 139.2, 135.9, 124.3, 119.1, 92.3, 40.5, 27.7; **IR (neat, cm^{-1})** = 3385 (br), 2952 (w), 1698 (s), 1560 (s), 1500 (s), 1399 (s), 1370 (s), 1141 (s), 1012 (m), 919 (m), 804 (m), 577 (m); **HRMS (ESI) (m/z):** calculated for $[M+H]^+$ $C_{11}H_{13}Cl_2INO^+$ 371.9419; found 371.9414.

***N*-(4-iodo-[1,1'-biphenyl]-3-yl)pivalamide (3s):**

Following the general procedure D using *N*-([1,1'-biphenyl]-3-yl)pivalamide (56 mg, 0.22 mmol, 1 equiv.), BBr_3 (0.26 mmol, 1.2 equiv., 1M in CH_2Cl_2), at 22 ºC for 2 h and Selectfluor (0.44 mmol, 2 equiv.), KI (0.48 mmol, 2.2 equiv.) at 60 ºC for 5 h. The crude product was purified by automated column chromatography (pentane/EtOAc, 90:10) and the desired product was obtained as an off white solid (81 mg, 97%); **Rf:** 0.70 (hexane/EtOAc, 90:10); **Mp:**113-115 ºC; **^{1}H NMR (400 MHz, CDCl$_3$) δ=** 8.63 (d, *J* = 2.2 Hz, 1H), 7.87 (bs, 1H), 7.82 (d, *J* = 8.2 Hz, 1H), 7.65 – 7.59 (m, 2H), 7.42 (dd, *J* = 8.3, 6.6 Hz, 2H), 7.38 – 7.31 (m, 1H), 7.09 (dd, *J* = 8.2, 2.2 Hz, 1H), 1.40 (s, 9H); **^{13}C{^{1}H} NMR (101 MHz, CDCl$_3$) δ=** 177.1, 142.7, 139.9, 138.9, 138.7, 128.9, 127.9, 127.2, 124.4, 120.4, 88.6, 40.4, 27.8; **IR (neat, cm^{-1})** = 3399 (br), 2960 (w), 2928 (w), 1688 (s), 1560 (s), 1521 (s), 1491 (s), 1400 (s), 1157 (m), 759 (s), 697 (m), 571 (w); **HRMS (ESI) (m/z):** calculated for $[M+H]^+$ $C_{17}H_{19}INO^+$ 380.0511; found 380.0518.

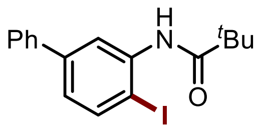

3s

***N*-(3-iodo-[1,1'-biphenyl]-4-yl)pivalamide (3t):**

Following the general procedure D using *N*-([1,1'-biphenyl]-4-yl)pivalamide (56 mg, 0.22 mmol, 1 equiv.), BBr_3 (0.26 mmol, 1.2 equiv., 1M in CH_2Cl_2), at 22 ºC for 2 h and Selectfluor (0.44 mmol, 2 equiv.), KI (0.48 mmol, 2.2 equiv.) at 60 ºC for 5 h. The crude product was purified by automated column chromatography (pentane/EtOAc, 90:10) and the desired product was obtained as an off white solid (56 mg, 67%); **Rf:** 0.60 (hexane/EtOAc, 90:10); **Mp:** 106-108 ºC; **^{1}H NMR (400 MHz, CDCl$_3$) δ=** 8.36 (d, *J* = 8.5 Hz, 1H), 8.01 (d, *J* = 2.1 Hz, 1H), 7.86 (bs, 1H), 7.58 (dd, *J* = 8.6, 2.1 Hz, 1H), 7.56 – 7.52 (m, 2H), 7.43 (td, *J* = 7.2, 1.2 Hz, 2H), 7.37 – 7.31 (m, 1H), 1.39 (s, 9H); **^{13}C{^{1}H} NMR (101 MHz, CDCl$_3$) δ=** 176.9, 139.2, 138.8, 137.6, 137.1, 129.0, 128.0, 127.7, 127.0, 121.7, 90.6, 40.3, 27.9; **IR (neat, cm^{-1})** = 3398 (br), 2959 (w), 2928 (w), 1688 (m), 1596 (m), 1561 (s), 1491 (s), 1379 (m), 1294 (s), 1157 (s), 833 (w), 761 (s), 696 (m), 665 (w), 571 (w); **HRMS (ESI) (m/z):** calculated for $[M+H]^+$ $C_{17}H_{19}INO^+$ 380.0511; found 380.0523.

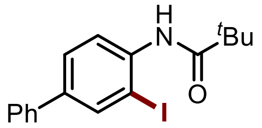

3t

N-(3-iodoanthracen-2-yl)pivalamide (3u):

Following the general procedure D using N-(anthracen-2-yl)pivalamide (61 mg, 0.22 mmol, 1 equiv.), BBr_3 (0.26 mmol, 1.2 equiv., 1M in CH_2Cl_2), at 22 °C for 2 h and Selectfluor (0.44 mmol, 2 equiv.), KI (0.48 mmol, 2.2 equiv.) at 60 °C for 5 h. The crude product was purified by automated column chromatography (hexane/EtOAc, 90:10) and the desired product was obtained as a yellow solid (38 mg, 43%); **Rf:** 0.5 (pentane/EtOAc, 90:10); **Mp:**163-165 °C; **^{1}H NMR (400 MHz, $CDCl_3$) δ=** 8.60 (d, J = 1.2 Hz, 1H), 8.36 – 8.31 (m, 2H), 8.18 (bs, 1H), 8.05 (dd, J = 8.6, 1.1 Hz, 1H), 8.00 (d, J = 8.1 Hz, 1H), 7.95 (d, J = 9.3 Hz, 1H), 7.54 – 7.45 (m, 2H), 1.45 (s, 9H); **$^{13}C\{^1H\}$ NMR (101 MHz, $CDCl_3$) δ=** 177.2, 138.0, 133.0, 132.4, 131.4, 130.6, 129.9, 129.8, 128.3, 127.9, 127.2, 126.4, 125.9, 121.4, 92.9, 40.4, 27.9; **IR (neat, cm^{-1})** = 3391 (br), 2958 (m), 2925 (m), 1683 (s), 1617 (m), 1533 (s), 1483 (s), 1419 (m), 1297 (s), 1153 (m), 872 (m), 739 (m), 669 (w), 577 (w); **HRMS (ESI) (m/z):** calculated for $[M+H]^+$ $C_{19}H_{19}INO^+$ 404.0511; found 404.0515.

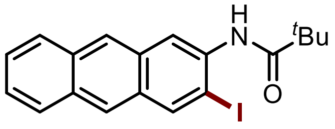

3u

N-(4-iodo-[1,1':4',1''-terphenyl]-3-yl)pivalamide (3v):

Following the general procedure D using N-([1,1':4',1''-terphenyl]-3-yl)pivalamide (72.5 mg, 0.22 mmol, 1 equiv.), BBr_3 (0.26 mmol, 1.2 equiv., 1M in CH_2Cl_2), at 22 °C for 2 h and Selectfluor (0.44 mmol, 2 equiv.), KI (0.48 mmol, 2.2 equiv.) at 60 °C for 5 h. The crude product was purified by automated column chromatography (pentane/EtOAc, 90:10) and the desired product was obtained as an off white solid (88 mg, 88%); **Rf:** 0.43 (hexane/EtOAc, 90:10); **Mp:**185-187 °C; **^{1}H NMR (400 MHz, $CDCl_3$) δ=** 8.68 (d, J = 2.2 Hz, 1H), 7.89 (bs, 1H), 7.84 (d, J = 8.2 Hz, 1H), 7.71 (d, J = 8.1 Hz, 2H), 7.68 – 7.61 (m, 4H), 7.46 (t, J = 7.5 Hz, 2H), 7.37 (t, J = 7.3 Hz, 1H), 7.14 (dd, J = 8.3, 2.3 Hz, 1H), 1.41 (s, 9H); **$^{13}C\{^1H\}$ NMR (101 MHz, $CDCl_3$) δ=** 177.1, 142.1, 140.8, 140.7, 139.0, 138.8, 138.8, 129.0, 127.6, 127.6, 127.6, 127.2, 124.2, 120.2, 88.6, 40.4, 27.9; **IR (neat, cm^{-1})** = 3260 (br), 2962 (m), 2927 (m), 1684 (s), 1558 (m), 1506 (s), 814 (w), 763 (s), 668 (m); **HRMS (ESI) (m/z):** calculated for $[M+H]^+$ $C_{23}H_{23}INO^+$ 456.0824; found 456.0828.

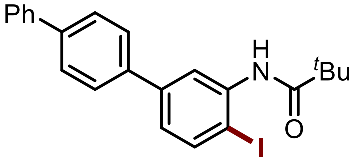

3v

N-(2-iodo-4-tritylphenyl)pivalamide (3w):

Following the general procedure D using N-(4-tritylphenyl)pivalamide (63 mg, 0.15 mmol, 1 equiv.), BBr_3 (0.18 mmol, 1.2 equiv., 1M in CH_2Cl_2), at 22 °C for 2 h and Selectfluor (0.30 mmol, 2 equiv.), KI (0.33 mmol, 2.2 equiv.) at 60 °C for 5 h. The crude product was purified by automated column chromatography (pentane/EtOAc, 90:10) and the desired product was obtained as an off white solid (72 mg, 88%); **Rf:** 0.43 (hexane/EtOAc, 90:10); **Mp:** 221-223 °C; **^{1}H NMR (400 MHz, $CDCl_3$) δ=** 8.15 (d, J = 8.7 Hz, 1H), 7.78 (bs, 1H), 7.63 (d, J = 2.2 Hz, 1H), 7.26 – 7.22 (m, 6H), 7.21 – 7.16 (m, 10H),

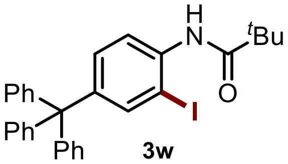

3w

1.34 (s, 9H); **$^{13}C\{^1H\}$ NMR (101 MHz, $CDCl_3$) δ=** 176.9, 146.4, 144.7, 140.6, 136.3, 132.7, 131.1, 127.8, 126.3, 120.4, 89.5, 64.4, 40.3, 27.8; **IR (neat, cm^{-1})** = 2958 (w), 2926 (w), 1697 (m), 1560 (m), 1505 (s), 1298 (m), 702 (m), 668 (m); **HRMS (ESI) (m/z):** calculated for $[M+H]^+$ $C_{30}H_{29}INO^+$ 546.1294; found 546.1292.

***N,N'*-(oxybis(2-iodo-4,1-phenylene))bis(2,2-dimethylpropanamide) (3x):**

Following the general procedure D using *N,N'*-(oxybis(4,1-phenylene))bis(2,2-dimethylpropanamide) (81 mg, 0.22 mmol, 1 equiv.), BBr_3 (0.48 mmol, 2.2 equiv., 1M in CH_2Cl_2), at 22 ºC for 2 h and Selectfluor (0.88 mmol, 4 equiv.), KI (0.92 mmol, 4.2 equiv.) at 60 ºC for 5 h. The reaction was performed in 10 mL R.B.F. The crude product was purified by automated column chromatography (pentane/EtOAc, 80:20) and the desired product was obtained as an off white solid (85 mg, 62%); **Rf:** 0.16 (hexane/EtOAc, 90:10); **Mp:**191-193 ºC; **^1H NMR (400 MHz, $CDCl_3$) δ=** 8.18 (d, *J* = 9.0 Hz, 2H), 7.70 (bs, 2H), 7.40 (d, *J* = 2.8 Hz, 2H), 7.00 (dd, *J* = 9.0, 2.7 Hz, 2H), 1.37 (s, 18H); **$^{13}C\{^1H\}$ NMR (101 MHz, $CDCl_3$) δ=** 176.9, 153.5, 134.5, 128.7, 122.8, 119.6, 90.5, 40.2, 27.8; **IR (neat, cm^{-1})** = 3336 (br), 3270 (br), 2960 (w), 2925 (w), 1655 (s), 1583 (w), 1507 (s), 1469 (s), 1395 (w), 1259 (m), 1204 (s), 1168 (s), 1028 (w), 915 (m), 809 (w), 606 (w); **HRMS (ESI) (m/z):** calculated for $[M+H]^+$ $C_{22}H_{27}I_2N_2O_3^+$ 621.0111; found 621,011.

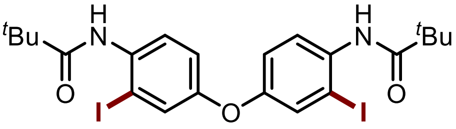

3x

***N*-(8-iodo-3,4-dihydro-2H-benzo[b][1,4]dioxepin-7-yl)pivalamide (3y):**

Following the general procedure D using *N*-(3,4-dihydro-2H-benzo[b][1,4]dioxepin-7-yl)pivalamidede (55 mg, 0.22 mmol, 1 equiv.), BBr_3 (0.26 mmol, 1.2 equiv., 1M in CH_2Cl_2), at 22 ºC for 2 h and Selectfluor (0.44 mmol, 2 equiv.), KI (0.48 mmol, 2.2 equiv.) at 60 ºC for 5 h. The crude product was purified by automated column chromatography (pentane/EtOAc, 90:10) and the desired product was obtained as an off white solid (51 mg, 62%); **Rf:** 0.56 (hexane/EtOAc, 70:30); **Mp:**127-129 ºC; **^1H NMR (400 MHz, $CDCl_3$) δ=** 7.93 (s, 1H), 7.60 (bs, 1H), 7.38 (s, 1H), 4.16 (dt, *J* = 13.3, 5.6 Hz, 4H), 2.16 (p, *J* = 5.6 Hz, 2H), 1.34 (s, 9H); **$^{13}C\{^1H\}$ NMR (101 MHz, $CDCl_3$) δ=** 176.6, 152.0, 148.5, 133.9, 130.5, 115.1, 81.1, 71.1, 70.7, 40.1, 31.9, 27.8; **IR (neat, cm^{-1})** = 3401 (br), 2960 (w), 1681 (m), 1572 (w), 1508 (s), 1401 (m), 1308 (s), 1197 (s), 979 (m), 923 (w), 836 (w), 653 (w); **HRMS (ESI) (m/z):** calculated for $[M+H]^+$ $C_{14}H_{19}INO_3^+$ 376.041; found 376.0406.

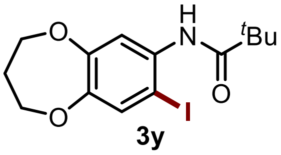

3y

2-(diethylamino)ethyl 3-iodo-4-pivalamidobenzoate (3z):

Following the general procedure D using 2-(diethylamino)ethyl 4-pivalamidobenzoate (48.9 mg, 0.15 mmol, 1 equiv.), BBr_3 (0.18 mmol, 1.2 equiv., 1M in CH_2Cl_2), at 40 ºC for 16 h and Selectfluor (0.30 mmol, 2 equiv.), KI (0.33 mmol, 2.2 equiv.) at 60 ºC for 5 h. The crude product was purified by automated

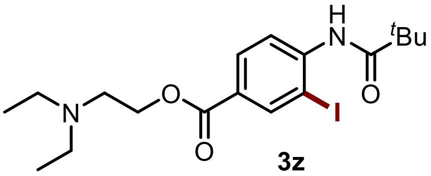

3z

column chromatography (pentane/EtOAc, 90:10) and the desired product was obtained as an semi solid (17 mg, 25%); **Rf:** 0.15 (EtOAc, 100); **^{1}H NMR (400 MHz, $CDCl_3$) δ=** 8.46 – 8.41 (m, 2H), 8.04 (bs, 1H), 7.98 (dd, *J* = 8.6, 2.0 Hz, 1H), 4.55 (t, *J* = 5.7 Hz, 2H), 3.23 (t, *J* = 5.7 Hz, 2H), 3.02 (q, *J* = 7.2 Hz, 4H), 1.37 (s, 9H), 1.25 (t, *J* = 7.2 Hz, 6H); **$^{13}C\{^1H\}$ NMR (101 MHz, $CDCl_3$) δ=** 176.6, 152.0, 148.5, 133.9, 130.5, 115.1, 81.1, 71.1, 70.7, 40.1, 31.9, 27.8; **IR (neat, cm^{-1})** = 3390 (br), 2963 (w), 2924 (w), 1719 (s), 1700 (s), 1592 (m), 1512 (s), 1385 (m), 1259 (s), 1228 (m), 1112 (s), 1067 (s), 1034 (s), 762 (m), 682 (w); **HRMS (ESI) (m/z):** calculated for $[M+H]^+$ $C_{18}H_{28}IN_2O_3^+$ 447.1145; found 447.1147.

***N*-(2-iodophenyl)benzamide (7a):**[11]

Following the general procedure E using *N*-phenylbenzamide (44 mg, 0.22 mmol, 1 equiv.), BBr_3 (0.26 mmol, 1.2 equiv., 1M in CH_2Cl_2), at 40 ºC for 16 h and Selectfluor (0.44 mmol, 2 equiv.), KI (0.48 mmol, 2.2 equiv.) at 60 ºC for 5 h. The crude product was purified by automated column chromatography (pentane/EtOAc, 90:10) and the desired product was obtained as an off white solid (60 mg, 84%); **Rf:** 0.43 (hexane/EtOAc, 90:10); **^{1}H NMR (400 MHz, $CDCl_3$) δ=** 8.47 (dd, *J* = 8.3, 1.6 Hz, 1H), 8.30 (bs, 1H), 7.98 (dt, *J* = 7.1, 1.5 Hz, 2H), 7.82 (dd, *J* = 8.0, 1.5 Hz, 1H), 7.63 – 7.57 (m, 1H), 7.56 – 7.49 (m, 2H), 7.45 – 7.37 (m, 1H), 6.89 (td, *J* = 7.6, 1.6 Hz, 1H); **$^{13}C\{^1H\}$ NMR (101 MHz, $CDCl_3$) δ=** 165.4, 138.9, 138.4, 134.7, 132.3, 129.6, 129.1, 127.3, 126.2, 121.9, 90.3.

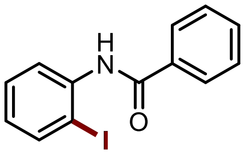

7a

***N*-(2-iodophenyl)-4-methylbenzamide (7b):**[12]

Following the general procedure E using 4-methyl-*N*-phenylbenzamide (46 mg, 0.22 mmol, 1 equiv.), BBr_3 (0.26 mmol, 1.2 equiv., 1M in CH_2Cl_2), at 40 ºC for 16 h and Selectfluor (0.44 mmol, 2 equiv.), KI (0.48 mmol, 2.2 equiv.) at 60 ºC for 5 h. The crude product was purified by automated column chromatography (pentane/EtOAc, 90:10) and the desired product was obtained as an off white solid (57 mg, 77%); **Rf:** 0.50 (hexane/EtOAc, 90:10); **^{1}H NMR (400 MHz, $CDCl_3$) δ=** 8.47 (dd, *J* = 8.2, 1.6 Hz, 1H), 8.27 (bs, 1H), 7.87 (d, *J* = 7.9 Hz, 2H), 7.81 (dd, *J* = 8.0, 1.5 Hz, 1H), 7.44 – 7.37 (m, 1H), 7.33 (d, *J* = 7.9 Hz, 2H), 6.88 (td, *J* = 7.6, 1.6 Hz, 1H), 2.44 (s, 3H); **$^{13}C\{^1H\}$ NMR (101 MHz, $CDCl_3$) δ=** 165.4, 142.9, 138.9, 138.5, 131.8, 129.8, 129.5, 127.3, 126.0, 121.8, 90.3, 21.7.

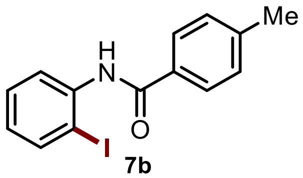

7b

***N*-(2-iodophenyl)-3-methylbenzamide (7c):** [13]

Following the general procedure E using 3-methyl-*N*-phenylbenzamide (46 mg, 0.22 mmol, 1 equiv.), BBr_3 (0.26 mmol, 1.2 equiv., 1M in CH_2Cl_2), at 40 ºC for 16 h and Selectfluor (0.44 mmol, 2 equiv.), KI (0.48 mmol, 2.2 equiv.) at 60 ºC for 5 h. The crude product was purified by automated column chromatography (pentane/EtOAc, 90:10) and the desired product was obtained as a off white solid (71 mg, 96%); **Rf:** 0.50 (hexane/EtOAc, 90:10); **^{1}H NMR (400 MHz, $CDCl_3$) δ=** 8.46 (d, *J* = 8.3 Hz,

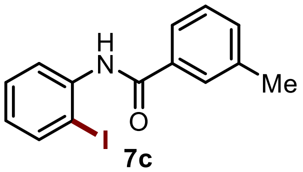

7c

1H), 8.27 (bs, 1H), 7.82 (d, *J* = 7.9 Hz, 1H), 7.79 (s, 1H), 7.75 (dt, *J* = 6.5, 2.2 Hz, 1H), 7.41 (dq, *J* = 8.0, 4.3 Hz, 3H), 6.88 (t, *J* = 7.6 Hz, 1H), 2.46 (s, 3H); **$^{13}C\{^1H\}$ NMR (101 MHz, $CDCl_3$) δ=** 165.7, 139.0, 138.9, 138.5, 134.7, 133.1, 129.5, 128.9, 128.2, 126.1, 124.1, 121.9, 90.4, 21.6.

4-fluoro-N-(2-iodophenyl)benzamide (7d): [11]

Following the general procedure E using 4-fluoro-*N*-phenylbenzamide (47 mg, 0.22 mmol, 1 equiv.), BBr_3 (0.26 mmol, 1.2 equiv., 1M in CH_2Cl_2), at 40 ºC for 16 h and Selectfluor (0.44 mmol, 2 equiv.), KI (0.48 mmol, 2.2 equiv.) at 60 ºC for 5 h. The crude product was purified by automated column chromatography (pentane/EtOAc, 90:10) and the desired product was obtained as an off white solid (56 mg, 75%); **Rf:** 0.56 (hexane/EtOAc, 80:20); **^1H NMR (400 MHz, $CDCl_3$) δ=** 8.42 (dd, *J* = 8.3, 1.6 Hz, 1H), 8.22 (s, 1H), 8.02 – 7.96 (m, 2H), 7.82 (dd, *J* = 8.0, 1.5 Hz, 1H), 7.40 (ddd, *J* = 8.6, 7.7, 1.5 Hz, 1H), 7.24 – 7.18 (m, 2H), 6.89 (ddd, *J* = 8.0, 7.3, 1.6 Hz, 1H); **$^{13}C\{^1H\}$ NMR (101 MHz, $CDCl_3$) δ=** 165.3 (d, *J* = 249.6 Hz), 164.4, 139.0, 138.2, 130.8 (d, *J* = 3.3 Hz), 129.8, 129.6 (d, *J* = 7.7 Hz), 126.3, 121.9, 116.2 (d, *J* = 22.0 Hz), 90.5; **^{19}F NMR (470 MHz, $CDCl_3$) δ=** -106.82.

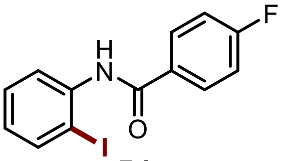

7d

4-iodo-*N*-(2-iodophenyl)benzamide (7e): [14]

Following the general procedure E using 4-iodo-*N*-phenylbenzamide (71 mg, 0.22 mmol, 1 equiv.), BBr_3 (0.26 mmol, 1.2 equiv., 1M in CH_2Cl_2), at 40 ºC for 16 h and Selectfluor (0.44 mmol, 2 equiv.), KI (0.48 mmol, 2.2 equiv.) at 60 ºC for 5 h. The crude product was purified by automated column chromatography (pentane/EtOAc, 90:10) and the desired product was obtained as an off white solid (64 mg, 65%); **Rf:** 0.46 (hexane/EtOAc, 90:10); **^1H NMR (400 MHz, $CDCl_3$) δ=** 8.42 (dd, *J* = 8.3, 1.6 Hz, 1H), 8.23 (bs, 1H), 7.88 (d, *J* = 8.4 Hz, 2H), 7.82 (dd, *J* = 7.9, 1.5 Hz, 1H), 7.69 (d, *J* = 8.4 Hz, 2H), 7.44 – 7.36 (m, 1H), 6.90 (td, *J* = 7.6, 1.6 Hz, 1H); **$^{13}C\{^1H\}$ NMR (101 MHz, $CDCl_3$) δ=** 164.7, 139.0, 138.3, 138.1, 134.1, 129.6, 128.8, 126.4, 121.9, 99.5, 90.4.

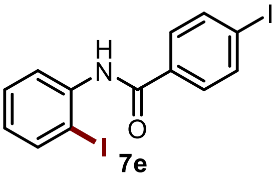

7e

2-bromo-*N*-(2-iodophenyl)benzamide (7f): [15]

Following the general procedure E using 2-bromo-*N*-phenylbenzamide (61 mg, 0.22 mmol, 1 equiv.), BBr_3 (0.26 mmol, 1.2 equiv., 1M in CH_2Cl_2), at 40 ºC for 16 h and Selectfluor (0.44 mmol, 2 equiv.), KI (0.48 mmol, 2.2 equiv.) at 60 ºC for 5 h. The crude product was purified by automated column chromatography (pentane/EtOAc, 90:10) and the desired product was obtained as an off white solid (46 mg, 52%); **Rf:** 0.46 (hexane/EtOAc, 80:20); **^1H NMR (400 MHz, $CDCl_3$) δ=** 8.42 (d, *J* = 8.2 Hz, 1H), 7.93 (s, 1H), 7.83 (dd, *J* = 7.9, 1.5 Hz, 1H), 7.68 (d, *J* = 7.8 Hz, 2H), 7.47 – 7.39 (m, 2H), 7.36 (td, *J* = 7.7, 1.8 Hz, 1H), 6.91 (td, *J* = 7.6, 1.6 Hz, 1H); **$^{13}C\{^1H\}$ NMR (101 MHz, $CDCl_3$) δ=** 165.8, 139.2, 138.2, 137.7, 133.9, 132.0, 129.6, 129.5, 127.9, 126.7, 122.4, 119.6, 90.3.

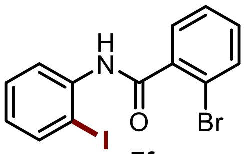

7f

2-iodo-*N*-(2-iodophenyl)benzamide (7g): [14]

Following the general procedure E using 2-iodo-*N*-phenylbenzamide (61 mg, 0.22 mmol, 1 equiv.), BBr_3 (0.26 mmol, 1.2 equiv., 1M in CH_2Cl_2), at 40 ºC for 16 h and Selectfluor (0.44 mmol, 2 equiv.), KI (0.48 mmol, 2.2 equiv.) at 60 ºC for 5 h. The crude product was purified by automated column chromatography (pentane/EtOAc, 90:10) and the desired product was obtained as an off white solid (46 mg, 52%); **Rf:** 0.46 (hexane/EtOAc, 80:20); **^{1}H NMR (400 MHz, $CDCl_3$) δ=** 8.41 (d, J = 8.2 Hz, 1H), 7.96 (dd, J = 8.0, 1.1 Hz, 1H), 7.83 (dd, J = 8.0, 1.5 Hz, 1H), 7.75 (bs, 1H), 7.58 (d, J = 7.6 Hz, 1H), 7.47 (t, J = 7.5 Hz, 1H), 7.42 (t, J = 7.8 Hz, 1H), 7.19 (td, J = 7.7, 1.7 Hz, 1H), 6.92 (td, J = 7.7, 1.6 Hz, 1H); **^{13}C{^{1}H} NMR (101 MHz, $CDCl_3$) δ=** 167.4, 141.8, 140.5, 139.1, 138.1, 131.9, 129.5, 128.6, 128.3, 126.7, 122.4, 92.7, 90.4.

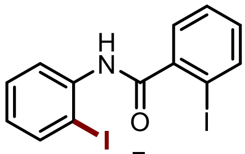

7g

***N*-(2-iodophenyl)-4-(trifluoromethyl)benzamide (7h):**[16]

Following the general procedure E using *N*-phenyl-4-(trifluoromethyl)benzamide (58 mg, 0.22 mmol, 1 equiv.), BBr_3 (0.26 mmol, 1.2 equiv., 1M in CH_2Cl_2), at 40 ºC for 16 h and Selectfluor (0.44 mmol, 2 equiv.), KI (0.48 mmol, 2.2 equiv.) at 60 ºC for 5 h. The crude product was purified by automated column chromatography (pentane/EtOAc, 90:10) and the desired product was obtained as a off white solid (47 mg, 55%); **Rf:** 0.56 (hexane/EtOAc, 80:20); **^{1}H NMR (400 MHz, $CDCl_3$) δ=** 8.42 (dd, J = 8.3, 1.6 Hz, 1H), 8.29 (bs, 1H), 8.08 (d, J = 8.2 Hz, 2H), 7.83 (dd, J = 7.9, 1.4 Hz, 1H), 7.80 (d, J = 8.3 Hz, 2H), 7.42 (td, J = 7.5, 1.5 Hz, 1H), 6.92 (td, J = 7.7, 1.6 Hz, 1H); **^{13}C{^{1}H} NMR (101 MHz, $CDCl_3$) δ=** 164.1, 139.0, 137.9, 137.9 (q, J = 1.4 Hz), 134.0 (q, J = 32.8 Hz), 129.6, 127.8, 126.7, 126.2 (q, J = 3.8 Hz), 123.6 (d, J = 272.5 Hz), 122.1, 90.6; **^{19}F NMR (470 MHz, $CDCl_3$) δ=** -63.01.

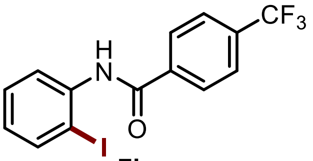

7h

***N*-(2-iodophenyl)-3-nitrobenzamide (7i):**

Following the general procedure E using 3-nitro-*N*-phenylbenzamide (53 mg, 0.22 mmol, 1 equiv.), BBr_3 (0.26 mmol, 1.2 equiv., 1M in CH_2Cl_2), at 40 ºC for 16 h and Selectfluor (0.44 mmol, 2 equiv.), KI (0.48 mmol, 2.2 equiv.) at 60 ºC for 5 h. The crude product was purified by automated column chromatography (pentane/EtOAc, 90:10) and the desired product was obtained as a off white solid (60 mg, 74%); **Rf:** 0.30 (hexane/EtOAc, 80:20); **^{1}H NMR (400 MHz, $CDCl_3$) δ=** 8.83 (t, J = 2.0 Hz, 1H), 8.44 (ddd, J = 8.2, 2.3, 1.1 Hz, 1H), 8.38 (dd, J = 8.2, 1.6 Hz, 1H), 8.31 (bs, 1H), 8.29 (t, J = 1.4 Hz, 1H), 7.84 (dd, J = 8.0, 1.5 Hz, 1H), 7.75 (t, J = 8.0 Hz, 1H), 7.43 (ddd, J = 8.5, 7.4, 1.5 Hz, 1H), 6.94 (td, J = 7.7, 1.6 Hz, 1H); **^{13}C{^{1}H} NMR (101 MHz, $CDCl_3$) δ=** 163.0, 148.6, 139.1, 137.7, 136.3, 133.1, 130.4, 129.7, 126.9, 126.8, 122.5, 122.2, 90.9.

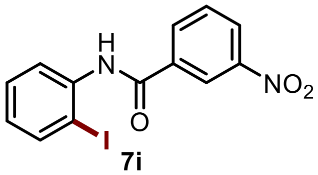

7i

***N*-(2-iodophenyl)furan-2-carboxamide (7j):** (16)

Following the general procedure E using *N*-phenylfuran-2-carboxamide (41 mg, 0.22 mmol, 1 equiv.), BBr_3 (0.26 mmol, 1.2 equiv., 1M in CH_2Cl_2), at 40 ºC for 16 h and Selectfluor (0.44 mmol, 2 equiv.), KI

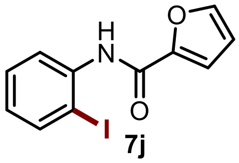

7j

(0.48 mmol, 2.2 equiv.) at 60 ºC for 5 h. The crude product was purified by automated column chromatography (pentane/EtOAc, 90:10) and the desired product was obtained as an off white solid (56 mg, 86%); **Rf:** 0.50 (hexane/EtOAc, 80:20); **^{1}H NMR (400 MHz, $CDCl_3$) δ=** 8.54 (bs, 1H), 8.40 (dd, *J* = 8.2, 1.6 Hz, 1H), 7.81 (dd, *J* = 8.0, 1.5 Hz, 1H), 7.58 (dd, *J* = 1.8, 0.8 Hz, 1H), 7.38 (td, *J* = 7.8, 1.5 Hz, 1H), 7.27 (dd, *J* = 3.5, 0.9 Hz, 1H), 6.87 (ddd, *J* = 7.9, 7.3, 1.6 Hz, 1H), 6.58 (dd, *J* = 3.5, 1.8 Hz, 1H); **^{13}C{^{1}H} NMR (101 MHz, $CDCl_3$) δ=** 156.1, 147.8, 144.8, 139.1, 138.0, 129.5, 126.1, 121.7, 115.8, 112.8, 89.9.

***N*-(2-iodophenyl)thiophene-2-carboxamide (7k):** (16)

Following the general procedure E using *N*-phenylthiophene-2-carboxamide (45 mg, 0.22 mmol, 1 equiv.), BBr_3 (0.26 mmol, 1.2 equiv., 1M in CH_2Cl_2), at 40 ºC for 16 h and Selectfluor (0.44 mmol, 2 equiv.), KI

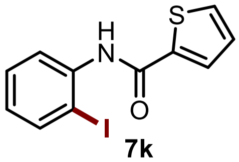

7k

(0.48 mmol, 2.2 equiv.) at 60 ºC for 5 h. The crude product was purified by automated column chromatography (pentane/EtOAc, 90:10) and the desired product was obtained as an off white solid (62 mg, 86%); **Rf:** 0.50 (hexane/EtOAc, 80:20); **^{1}H NMR (400 MHz, $CDCl_3$) δ=** 8.39 (dd, *J* = 8.2, 1.6 Hz, 1H), 8.17 (bs, 1H), 7.81 (dd, *J* = 7.9, 1.5 Hz, 1H), 7.72 (dd, *J* = 3.8, 1.2 Hz, 1H), 7.59 (dd, *J* = 5.0, 1.2 Hz, 1H), 7.39 (ddd, *J* = 8.5, 7.3, 1.5 Hz, 1H), 7.17 (dd, *J* = 5.0, 3.7 Hz, 1H), 6.88 (ddd, *J* = 8.0, 7.3, 1.6 Hz, 1H); **^{13}C{^{1}H} NMR (101 MHz, $CDCl_3$) δ=** 159.8, 139.3, 138.9, 138.1, 131.5, 129.6, 128.8, 128.1, 126.2, 121.8, 90.1.

***N*-(5-chloro-2-iodophenyl)-4-methylbenzamide (7l):**

Following the general procedure E using *N*-(3-chlorophenyl)-4-methylbenzamide (54.08 mg, 0.22 mmol, 1 equiv.), BBr_3 (0.26 mmol, 1.2 equiv., 1M in CH_2Cl_2), at 40 ºC for 16 h and Selectfluor (0.44 mmol, 2

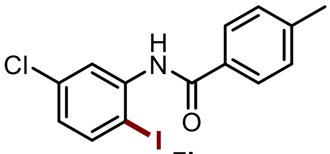

7l

equiv.), KI (0.48 mmol, 2.2 equiv.) at 60 ºC for 5 h. The crude product was purified by automated column chromatography (pentane/EtOAc, 90:10) and the desired product was obtained as a off white solid (58 mg, 71%); **Rf:** 0.40 (hexane/EtOAc, 90:10); **Mp:**133-135 ºC; **^{1}H NMR (400 MHz, $CDCl_3$) δ=** 8.58 (d, *J* = 2.5 Hz, 1H), 8.27 (bs, 1H), 7.85 (d, *J* = 8.2 Hz, 2H), 7.70 (d, *J* = 8.5 Hz, 1H), 7.32 (d, *J* = 7.9 Hz, 2H), 6.87 (dd, *J* = 8.5, 2.5 Hz, 1H), 2.44 (s, 3H); **^{13}C{^{1}H} NMR (101 MHz, $CDCl_3$) δ=** 165.3, 143.3, 139.4, 139.3, 135.6, 131.4, 129.8, 127.3, 125.9, 121.5, 86.9, 21.7; **HRMS (ESI) (m/z):** calculated for $[M+H]^+$ $C_{14}H_{12}ClINO^+$ 371.9652; found 371.9658.

(*3r,5r,7r*)-*N*-(2-iodophenyl)adamantane-1-carboxamide (7m): (17)

Following the general procedure E using (*3r*,*5r*,*7r*)-*N*-phenyladamantane-1-carboxamide (56 mg, 0.22 mmol, 1 equiv.), BBr_3 (0.26 mmol, 1.2 equiv., 1M in CH_2Cl_2), at 40 ºC for 16 h and Selectfluor (0.44

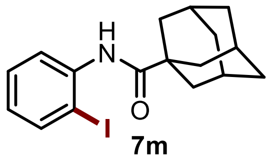

7m

mmol, 2 equiv.), KI (0.48 mmol, 2.2 equiv.) at 60 ºC for 5 h. The crude product was purified by automated column chromatography (pentane/EtOAc, 90:10) and the desired product was obtained as an off white solid (61 mg, 73%); **Rf:** 0.63 (hexane/EtOAc, 90:10); **^{1}H NMR (400 MHz, $CDCl_3$) δ=** 8.31 (dd, *J* = 8.3, 1.6 Hz, 1H), 7.76 (dd, *J* = 9.4, 2.3 Hz, 2H), 7.33 (ddd, *J* = 8.5, 7.3, 1.5 Hz, 1H), 6.82 (td, *J* = 7.6, 1.6 Hz, 1H), 2.12 (dd, *J* = 6.1, 3.1 Hz, 3H), 2.02 (d, *J* = 3.0 Hz, 6H), 1.81 – 1.74 (m, 6H); **^{13}C{^{1}H} NMR (101 MHz, $CDCl_3$) δ=** 176.4, 138.8, 138.4, 129.4, 125.7, 121.9, 90.2, 42.2, 39.5, 36.6, 28.3.

***N*-(2-iodophenyl)acetamide (7n):** [(16)]

Following the general procedure E using *N*-phenylacetamide (29.75 mg, 0.22 mmol, 1 equiv.), BBr_3 (0.66 mmol, 3 equiv., 1M in CH_2Cl_2), at 60 ºC for 24 h and Selectfluor (0.44 mmol, 2 equiv.), KI (0.48 mmol, 2.2 equiv.) at 60 ºC for 5 h. The crude product was purified by automated column chromatography (pentane/EtOAc, 90:10) and the desired product was obtained as a off white solid (18 mg, 31%); **^{1}H NMR (400 MHz, $CDCl_3$) δ=** 8.19 (d, *J* = 8.2 Hz, 1H), 7.77 (dd, *J* = 8.0, 1.5 Hz, 1H), 7.43 (bs, 1H), 7.34 (td, *J* = 7.8, 1.5 Hz, 1H), 6.84 (t, *J* = 7.7 Hz, 1H), 2.24 (s, 3H); **^{13}C{^{1}H} NMR (101 MHz, $CDCl_3$) δ=** 168.4, 138.9, 138.3, 129.4, 126.1, 122.2, 90.1, 25.0.

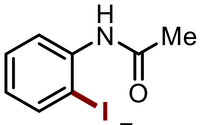

7n

1-(2-iodophenyl)-3-phenylurea (7o):[(18)]

Following the general procedure E using 1,3-diphenylurea (47 mg, 0.22 mmol, 1 equiv.), BBr_3 (0.26 mmol, 1.2 equiv., 1M in CH_2Cl_2), at 40 ºC for 16 h and Selectfluor (0.44 mmol, 2 equiv.), KI (0.48 mmol, 2.2 equiv.) at 60 ºC for 5 h. The crude product was purified by automated column chromatography (pentane/EtOAc, 80:20) and the desired product was obtained as an off white solid (47 mg, 63%); **Rf:** 0.53 (hexane/EtOAc, 70:30); **^{1}H NMR (400 MHz, dmso-d^6) δ=** 9.42 (bs, 1H), 7.88 (bs, 1H), 7.83 (dt, *J* = 8.2, 1.3 Hz, 2H), 7.47 (dd, *J* = 8.6, 1.2 Hz, 2H), 7.34 (ddd, *J* = 8.5, 7.3, 1.5 Hz, 1H), 7.29 (dd, *J* = 8.5, 7.3 Hz, 2H), 6.98 (tt, *J* = 7.4, 1.2 Hz, 1H), 6.84 (td, *J* = 7.7, 1.6 Hz, 1H); **^{13}C{^{1}H} NMR (101 MHz, dmso-d^6) δ=** 152.4, 139.9, 139.6, 139.0, 128.9, 128.6, 125.1, 123.1, 122.0, 118.2, 91.4.

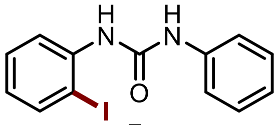

7o

1-(4-fluorophenyl)-3-(2-iodophenyl)urea (7p):

Following the general procedure E using 1-(4-fluorophenyl)-3-phenylurea (51 mg, 0.22 mmol, 1 equiv.), BBr_3 (0.26 mmol, 1.2 equiv., 1M in CH_2Cl_2), at 40 ºC for 16 h and Selectfluor (0.44 mmol, 2 equiv.), KI (0.48 mmol, 2.2 equiv.) at 60 ºC for 5 h. The crude product was purified by automated column chromatography (pentane/EtOAc, 80:20) and the desired product was obtained as an off white solid (32 mg, 41%); **Rf:** 0.50 (hexane/EtOAc, 70:30); **Mp:**224-226 ºC; **^{1}H NMR (400 MHz, dmso-d^6) δ=** 9.45 (bs, 1H), 7.86 (bs, 1H), 7.83 (ddd, *J* = 8.2, 5.1, 1.5 Hz, 2H), 7.51 – 7.44 (m, 2H), 7.34 (ddd, *J* = 8.4, 7.3, 1.5 Hz, 1H), 7.17 – 7.09

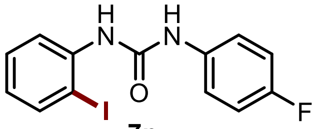

7p

(m, 2H), 6.84 (ddd, J = 7.8, 7.3, 1.6 Hz, 1H); **$^{13}C\{^1H\}$ NMR (101 MHz, dmso-d^6) δ=** 157.4 (d, J = 238.1 Hz), 152.5, 139.8, 139.0, 136.0 (d, J = 2.3 Hz), 128.6, 125.1, 123.1, 119.9 (d, J = 7.7 Hz), 115.4 (d, J = 22.2 Hz), 91.5; **^{19}F NMR (470 MHz, dmso-d^6) δ=** -121.19; **IR (neat, cm^{-1})** = 3278 (br), 2917 (w), 1643 (s), 1575 (m), 1568 (s), 1508 (s), 1288 (w), 1213 (w), 1015 (w), 747 (w), 668 (m); **HRMS (ESI) (m/z):** calculated for $[M+H]^+$ $C_{13}H_{11}FIN_2O^+$ 356.99; found 356.9912.

1-benzyl-3-(2-iodophenyl)urea (7q):[(19)]

Following the general procedure E using 1-benzyl-3-phenylurea (49.8 mg, 0.22 mmol, 1 equiv.), BBr_3 (0.26 mmol, 1.2 equiv., 1M in CH_2Cl_2), at 40 ºC for 16 h and Selectfluor (0.44 mmol, 2 equiv.), KI (0.48 mmol, 2.2 equiv.) at 60 ºC for 5 h. The crude product was purified by automated column chromatography (pentane/EtOAc, 80:20) and the desired product was obtained as an off white solid (27 mg, 35%); **Rf:** 0.5 (hexane/EtOAc, 70:30); **^1H NMR (400 MHz, acetone d$_6$) δ=** 8.12 (dd, J = 8.3, 1.6 Hz, 1H), 7.79 (dd, J = 7.9, 1.5 Hz, 1H), 7.40 – 7.27 (m, 6H), 7.28 – 7.20 (m, 1H), 7.01 (s, 1H), 6.77 (td, J = 7.6, 1.6 Hz, 1H), 4.43 (d, J = 5.8 Hz, 2H); **$^{13}C\{^1H\}$ NMR (101 MHz, dmso-d^6) δ=** 155.7, 141.6, 141.0, 139.8, 129.5, 129.2, 128.3, 127.7, 125.0, 122.7, 89.6, 44.3.

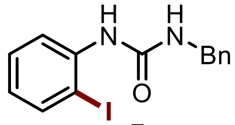

7q

1-(2-iodophenyl)-3-propylurea (7r):[(20)]

Following the general procedure E using 1-phenyl-3-propylurea (39.2 mg, 0.22 mmol, 1 equiv.), BBr_3 (0.26 mmol, 1.2 equiv., 1M in CH_2Cl_2), at 40 ºC for 16 h and Selectfluor (0.44 mmol, 2 equiv.), KI (0.48 mmol, 2.2 equiv.) at 60 ºC for 5 h. The crude product was purified by automated column chromatography (pentane/EtOAc, 80:20) and the desired product was obtained as an off white solid (24 mg, 36%); **Rf:** 0.4 (hexane/EtOAc, 70:30); **^1H NMR (400 MHz, $CDCl_3$) δ=** 7.89 (dd, J = 8.2, 1.6 Hz, 1H), 7.75 (dd, J = 7.9, 1.5 Hz, 1H), 7.29 (ddd, J = 8.4, 7.3, 1.5 Hz, 1H), 6.77 (td, J = 7.6, 1.6 Hz, 1H), 6.64 (bs, 1H), 5.15 (bs, 1H), 3.21 (t, J = 7.2 Hz, 2H), 1.56 (h, J = 7.4 Hz, 2H), 0.93 (t, J = 7.4 Hz, 3H); **$^{13}C\{^1H\}$ NMR (101 MHz, dmso-d^6) δ=** 155.4, 139.5, 139.1, 129.3, 125.3, 122.8, 91.6, 42.5, 23.4, 11.5.

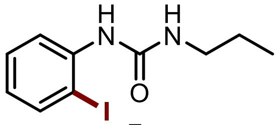

7r

5-(4-chlorophenyl)-*N*-(2-iodophenyl)furan-2-carboxamide (7s):

Following the general procedure E using 5-(4-chlorophenyl)-*N*-phenylfuran-2-carboxamide (45 mg, 0.15 mmol, 1 equiv.), BBr_3 (0.18 mmol, 1.2 equiv., 1M in CH_2Cl_2), at 40 ºC for 16 h and Selectfluor (0.30 mmol, 2 equiv.), KI (0.33 mmol, 2.2 equiv.) at 60 ºC for 5 h. The crude product was purified by automated column chromatography (pentane/EtOAc, 90:10) and the desired product was obtained as an off white solid (35 mg, 54%); **Rf:** 0.57 (hexane/EtOAc, 70:30); **Mp:**190-192 ºC; **^1H NMR (400 MHz, $CDCl_3$) δ=** 8.68 (bs, 1H), 8.47 (dd, J = 8.3, 1.6 Hz, 1H), 7.83 (dd, J = 7.9, 1.4 Hz, 1H), 7.71 (d, J = 8.7 Hz, 2H), 7.43 (d, J = 8.7 Hz, 2H), 7.41 – 7.36 (m, 1H), 7.34 (d, J = 3.6 Hz,

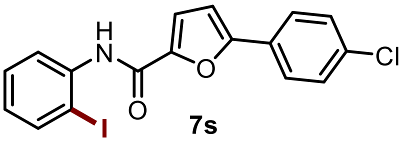

7s

1H), 6.88 (ddd, J = 8.0, 7.3, 1.5 Hz, 1H), 6.81 (d, J = 3.6 Hz, 1H); **$^{13}C\{^1H\}$ NMR (101 MHz, $CDCl_3$) δ=** 155.9, 155.0, 146.8, 139.0, 138.0, 134.9, 129.6, 129.4, 128.0, 126.1, 125.9, 121.3, 117.9, 108.3, 89.8; **IR (neat, cm^{-1})** = 3366 (br), 1684 (m), 1587 (m), 1539 (s), 1456 (w), 1431 (w), 1312 (m), 746 (w), 668 (s), 580 (w); **HRMS (ESI) (m/z):** calculated for $[M+H]^+$ $C_{17}H_{12}ClINO_2^+$ 423.9601; found 423.9608.

***N*-(2-iodophenyl)-5-(3-(trifluoromethyl)phenyl)furan-2-carboxamide (7t):**

Following the general procedure E using *N*-phenyl-5-(3-(trifluoromethyl)phenyl)furan-2-carboxamide (50 mg, 0.15 mmol, 1 equiv.), BBr_3 (0.18 mmol, 1.2 equiv., 1M in CH_2Cl_2), at 40 ºC for 16 h and Selectfluor (0.30 mmol, 2 equiv.), KI (0.33 mmol, 2.2 equiv.) at 60 ºC for 5 h. The crude product was purified by automated column chromatography (pentane/EtOAc, 90:10) and the desired product was obtained as an off white solid (38 mg, 54%); **Rf:** 0.40 (hexane/EtOAc, 80:20); **Mp:**145-147 ºC; **^1H NMR (400 MHz, $CDCl_3$) δ=** 8.77 (bs, 1H), 8.49 (dd, J = 8.2, 1.5 Hz, 1H), 8.08 (tt, J = 1.7, 0.8 Hz, 1H), 7.95 (dt, J = 7.1, 1.8 Hz, 1H), 7.84 (dd, J = 7.9, 1.4 Hz, 1H), 7.64 – 7.55 (m, 2H), 7.40 (ddd, J = 8.6, 7.2, 1.5 Hz, 1H), 7.36 (d, J = 3.6 Hz, 1H), 6.93 – 6.86 (m, 2H); **$^{13}C\{^1H\}$ NMR (101 MHz, $CDCl_3$) δ=** 155.7, 154.2, 147.2, 139.1, 137.9, 131.70 (q, J = 32.6 Hz), 130.3, 129.8, 129.7, 127.6, 126.7 (q, J = 272.7 Hz), 126.1, 125.5 (q, J = 3.7 Hz), 121.5 (q, J = 3.8 Hz), 121.2, 117.7, 109.0, 89.6; **^{19}F NMR (564 MHz, CDCl3) δ=** -62.97; **IR (neat, cm^{-1})** = 3357 (br), 2922 (w), 1684 (s), 1596 (s), 1508 (s), 1585 (s), 1539 (s), 1522 (s), 1433 (s), 1366 (s), 1311 (m), 1258 (m), 1120 (s), 806 (w), 750 (m), 668 (w); **HRMS (ESI) (m/z):** calculated for $[M+H]^+$ $C_{18}H_{12}F_3INO_2^+$ 457.9865; found 457.9865.

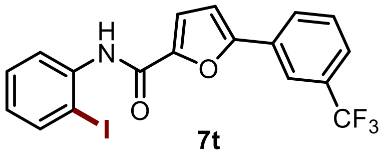

7t

***N*-(2-iodophenyl)-4-(methylsulfonyl)benzamide (7u):**

Following the general procedure E using 4-(methylsulfonyl)-*N*-phenylbenzamidede (42 mg, 0.15 mmol, 1 equiv.), BBr_3 (0.18 mmol, 1.2 equiv., 1M in CH_2Cl_2), at 40 ºC for 16 h and Selectfluor (0.30 mmol, 2 equiv.), KI (0.33 mmol, 2.2 equiv.) at 60 ºC for 5 h. The crude product was purified by automated column chromatography (pentane/EtOAc, 85:15) and the desired product was obtained as an off white solid (44 mg, 72%); **Rf:** 0.23 (hexane/EtOAc, 60:40); **Mp:**161-163 ºC; **^1H NMR (400 MHz, $CDCl_3$) δ=** 8.40 (dd, J = 8.2, 1.5 Hz, 1H), 8.29 (bs, 1H), 8.18 – 8.09 (m, 4H), 7.84 (dd, J = 8.0, 1.4 Hz, 1H), 7.43 (ddd, J = 8.5, 7.3, 1.5 Hz, 1H), 6.94 (ddd, J = 8.0, 7.4, 1.6 Hz, 1H), 3.11 (s, 3H); **$^{13}C\{^1H\}$ NMR (101 MHz, $CDCl_3$) δ=** 163.7, 143.7, 139.5, 139.1, 137.7, 129.7, 128.4, 128.3, 126.9, 122.1, 90.7, 44.5; **IR (neat, cm^{-1})** = 2923 (br), 1684 (m), 1653 (m), 1559 (m), 1521 (s), 1313 (s), 1297 (s), 1152 (s), 749 (w), 668 (s); **HRMS (ESI) (m/z):** calculated for $[M+H]^+$ $C_{14}H_{13}INO_3S^+$ 401.9661; found 401.9666.

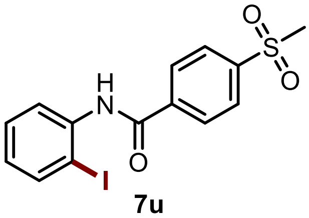

7u

***N*-(2-iodo-4-tritylphenyl)benzamide (7v):**

Following the general procedure E using *N*-(4-tritylphenyl)benzamide (63 mg, 0.15 mmol, 1 equiv.), BBr_3 (0.18 mmol, 1.2 equiv., 1M in CH_2Cl_2), at 40 ºC for 16 h and Selectfluor (0.30 mmol, 2 equiv.), KI (0.33 mmol, 2.2 equiv.) at 60 ºC for 5 h. The crude product was purified by automated column chromatography (pentane/EtOAc, 90:10) and the desired product was obtained as an off white solid (72 mg, 88%); **Rf:** 0.65 (hexane/EtOAc, 70:30); **Mp:** 199-201 ºC; **^{1}H NMR (400 MHz, $CDCl_3$) δ=** 8.31 (d, *J* = 8.7 Hz, 1H), 8.26 (bs, 1H), 7.94 (dt, *J* = 7.1, 1.4 Hz, 2H), 7.68 (d, *J* = 2.3 Hz, 1H), 7.61 – 7.55 (m, 1H), 7.51 (dd, *J* = 8.2, 6.5 Hz, 2H), 7.28 – 7.23 (m, 7H), 7.23 – 7.18 (m, 9H); **$^{13}C\{^1H\}$ NMR (101 MHz, $CDCl_3$) δ=** 165.5, 146.3, 145.1, 140.6, 136.2, 134.6, 132.8, 132.4, 131.1, 129.1, 127.8, 127.3, 126.3, 120.5, 89.7, 64.4; **IR (neat, cm^{-1})** = 3388 (br), 2924 (m), 1684 (m), 1560 (m), 1508 (s), 1491 (s), 1300 (s), 748 (w), 701 (s), 667 (w); **HRMS (ESI) (m/z):** calculated for $[M+H]^+$ $C_{32}H_{25}INO^+$ 566.0981; found 566.0983.

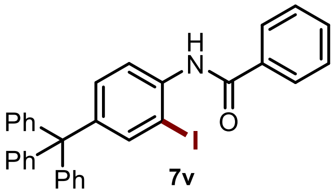

7v

4-(*N,N*-dipropylsulfamoyl)-*N*-(2-iodophenyl)benzamide (7w):

Following the general procedure E using 4-(*N,N*-dipropylsulfamoyl)-*N*-phenylbenzamide (55 mg, 0.15 mmol, 1 equiv.), BBr_3 (0.18 mmol, 1.2 equiv., 1M in CH_2Cl_2), at 40 ºC for 16 h and Selectfluor (0.30 mmol, 2 equiv.), KI (0.33 mmol, 2.2 equiv.) at 60 ºC for 5 h. The crude product was purified by automated column chromatography (pentane/EtOAc, 80:20) and the desired product was obtained as an off white solid (46 mg, 62%); **Rf:** 0.45 (hexane/EtOAc, 70:30); **Mp:** 115-117 ºC; **^{1}H NMR (400 MHz, $CDCl_3$) δ=** 8.41 (dd, *J* = 8.2, 1.5 Hz, 1H), 8.28 (s, 1H), 8.08 (d, *J* = 8.1 Hz, 2H), 7.96 (d, *J* = 8.2 Hz, 2H), 7.84 (dd, *J* = 8.0, 1.5 Hz, 1H), 7.47 – 7.37 (m, 1H), 6.92 (td, *J* = 7.6, 1.6 Hz, 1H), 3.12 (t, *J* = 7.4 Hz, 4H), 1.57 (h, *J* = 7.5 Hz, 4H), 0.89 (t, *J* = 7.4 Hz, 6H); **$^{13}C\{^1H\}$ NMR (101 MHz, $CDCl_3$) δ=** 164.0, 143.9, 139.1, 138.0, 137.9, 129.7, 128.0, 127.8, 126.7, 122.1, 90.6, 50.2, 22.2, 11.3; **IR (neat, cm^{-1})** = 3269 (br), 2922 (w), 2852 (w), 1651 (m), 1525 (m), 1500 (s), 1432 (m), 1312 (m), 1293 (m), 1232 (m), 850 (w), 756 (w), 651 (w); **HRMS (ESI) (m/z):** calculated for $[M+H]^+$ $C_{19}H_{24}IN_2O_3S^+$ 487.0552; found 487,0555.

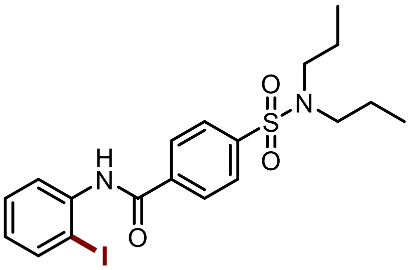

7w

1-(4-chlorophenyl)-*N*-(2-iodophenyl)cyclopentane-1-carboxamide (7x):

Following the general procedure E using 1-(4-chlorophenyl)-*N*-phenylcyclopentane-1-carboxamide (65 mg, 0.22 mmol, 1 equiv.), BBr_3 (0.26 mmol, 1.2 equiv., 1M in CH_2Cl_2), at 40 ºC for 16 h and Selectfluor (0.44 mmol, 2 equiv.), KI (0.48 mmol, 2.2 equiv.) at 60 ºC for 5 h. The crude product was purified by automated column chromatography (pentane/EtOAc, 91:9) and the desired product was obtained as an

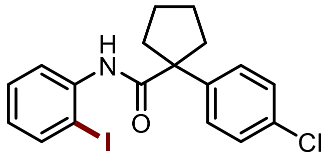

7x

off white solid (74 mg, 80%); **Rf:** 0.48 (hexane/EtOAc, 90:10); **Mp:** 94-96 °C; **^{1}H NMR (400 MHz, $CDCl_3$) δ=** 8.42 (dd, *J* = 8.3, 1.6 Hz, 1H), 7.95 (bs, 1H), 7.70 (dd, *J* = 7.9, 1.5 Hz, 1H), 7.37 – 7.33 (m, 13H), 7.30 (ddt, *J* = 6.8, 5.4, 2.3 Hz, 3H), 6.82 (td, *J* = 7.6, 1.6 Hz, 1H); **$^{13}C\{^1H\}$ NMR (101 MHz, $CDCl_3$) δ=** 172.1, 143.0, 139.1, 138.6, 130.9, 129.3, 128.4, 127.4, 126.1, 121.6, 89.3, 68.9; **IR (neat, cm^{-1})** = 3359 (br), 2955 (w), 1691 (s), 1579 (m), 1512 (s), 1429 (s), 1292 (m), 1012 (w), 752 (w), 668 (w);**HRMS (ESI) (m/z):** calculated for $[M+H]^+$ $C_{18}H_{18}ClINO^+$ 426.0122; found 426.0121.

***N*-(2-iodophenyl)-2,2,2-triphenylacetamide (7y):**[(14)]

Following the general procedure E using *N*,2,2,2-tetraphenylacetamide (56 mg, 0.15 mmol,1 equiv.), BBr_3 (0.18 mmol, 1.2 equiv., 1M in CH_2Cl_2), at 40 °C for 16 h and Selectfluor (0.30 mmol, 2 equiv.), KI (0.33 mmol, 2.2 equiv.) at 60 °C for 5 h. The crude product was purified by automated column chromatography (pentane/EtOAc, 93:7) and the desired product was obtained as an off white solid (46 mg, 62%); **Rf:** 0.36 (hexane/EtOAc, 95:5); **^{1}H NMR (400 MHz, $CDCl_3$) δ=** 8.42 (dd, *J* = 8.3, 1.6 Hz, 1H), 7.95 (bs, 1H), 7.70 (dd, *J* = 7.9, 1.5 Hz, 1H), 7.37 – 7.33 (m, 13H), 7.30 (ddt, *J* = 6.8, 5.4, 2.3 Hz, 3H), 6.82 (td, *J* = 7.6, 1.6 Hz, 1H); **$^{13}C\{^1H\}$ NMR (101 MHz, $CDCl_3$) δ=** 172.1, 143.0, 139.1, 138.6, 130.9, 129.3, 128.4, 127.4, 126.1, 121.6, 89.3, 68.9.

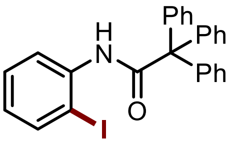

7y

***N*-(2-iodophenyl)-2-oxo-2-phenylacetamide (7z):** [(21)]

Following the general procedure E using 2-oxo-*N*,2-diphenylacetamide (51 mg, 0.22 mmol, 1 equiv.), BBr_3 (0.26 mmol, 1.2 equiv., 1M in CH_2Cl_2), at 40 °C for 16 h and Selectfluor (0.44 mmol, 2 equiv.), KI (0.48 mmol, 2.2 equiv.) at 60 °C for 5 h. The crude product was purified by automated column chromatography (pentane/EtOAc, 95:5) and the desired product was obtained as an off white solid (18 mg, 23%); **Rf:** 0.52 (hexane/EtOAc, 90:10); **^{1}H NMR (400 MHz, $CDCl_3$) δ=** 9.45 (bs, 1H), 8.47 – 8.38 (m, 3H), 7.85 (dd, *J* = 8.0, 1.5 Hz, 1H), 7.71 – 7.64 (m, 1H), 7.53 (t, *J* = 7.8 Hz, 2H), 7.46 – 7.38 (m, 1H), 6.93 (td, *J* = 7.7, 1.6 Hz, 1H); **$^{13}C\{^1H\}$ NMR (101 MHz, $CDCl_3$) δ=** 186.9, 159.0, 139.4, 137.4, 134.9, 133.1, 131.6, 129.5, 128.8, 127.0, 121.5, 90.3.

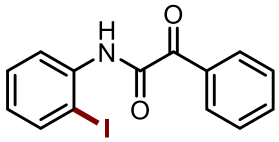

7z

4.4 General Procedure F for Chlorination of *N*-phenylpivalamide:

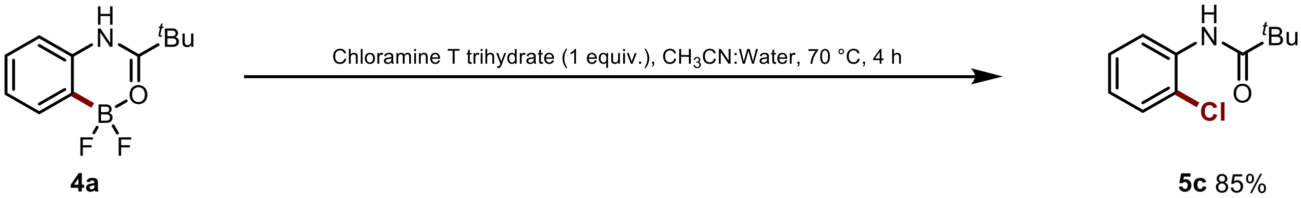

Step i) To a dry 3 mL, screw-top V-Vial, equipped with a stir bar, the difluoro boron complex (4a) (22.5 mg, 0.1 mmol, 1 equiv.) and Chloramine T Trihydrate (28.2 mg, 0.1 mmol, 1 equiv.). To this 0.7 mL

acetonitrile and 0.3 mL water was added and the reaction mixture was stirred at 70 ºC for 4 h. The reaction was diluted with EtOAc (10 mL) and H_2O (10 mL). The aqueous layer was washed with EtOAc (10 mL), and the combined organic layer was washed with brine, dried over sodium sulfate, filtered, and evaporated *in vacuo* to afford the crude product, which was purified using automated column chromatography (pentane/EtOAc 92:08) to afford the *N*-(2-chlorophenyl)pivalamide in 85% yield.

***N*-(2-chlorophenyl)pivalamide (5c):**[(14)]

Following the general procedure F. The crude product was purified by automated column chromatography (pentane/EtOAc, 92:08) and the desired product was obtained as beige color solid (18 mg, 85%); **Rf:** 0.37 (hexane/EtOAc, 95:5)**;** **^{1}H NMR (600 MHz, $CDCl_3$) δ=** 8.42 (dd, *J* = 8.3, 1.5 Hz, 1H), 8.02 (bs, 1H), 7.36 (dd, *J* = 8.1, 1.5 Hz, 1H), 7.30 – 7.24 (m, 1H), 7.02 (td, *J* = 7.7, 1.5 Hz, 1H), 1.35 (s, 9H); **^{13}C{^{1}H} NMR (151 MHz, $CDCl_3$) δ=** 176.8, 134.9, 129.0, 127.90, 124.5, 123.0, 121.5, 40.3, 27.7.

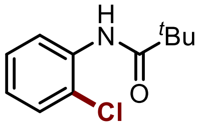

5c

4.5 General Procedure G for Bromination of *N*-phenylpivalamide

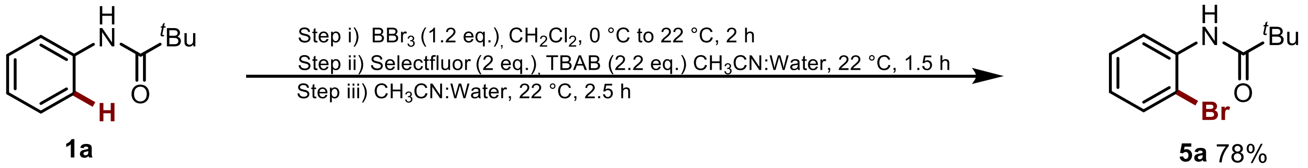

Step i) To a dry 5 mL, screw-top V-Vial, (Sigma Aldrich, Product code-Z115150-12EA), equipped with a rubber septum, stir bar, the *N*-phenylpivalamide (0.22 mmol, 1 equiv.) in anhydrous CH_2Cl_2 (0.5 mL) under a nitrogen atmosphere was added dropwise BBr_3 (0.26 mmol, 1.2 equiv., 1M solution in CH_2Cl_2). After the complete addition of BBr_3, the reaction mixture was stirred at 22 ºC for 2 h after which the solvent was removed under reduced pressure.

Step ii) Simultaneously, selectfluor (0.44 mmol, 2 equiv.), and TBAB (0.48 mmol, 2.2 equiv.), were dissolved in CH_3CN (1 mL) and water (0.5 mL) in a 3 mL, screw-top V-vial and the reaction mixture stirred at 22 ºC for 1.5 h.

Step iii) The crude residue from step i) was dissolved in CH_3CN (0.5 mL) and water (0.5 mL) at 0 ºC. To this mixture, the solution from step ii) was added dropwise at 0 ºC, and the reaction mixture was stirred at 22 ºC for 2.5 h. The reaction was quenched with saturated sodium thiosulfite solution at room temperature and the crude mixture was dissolved in EtOAc (10 mL) and H_2O (10 mL). The aqueous layer was washed with EtOAc (10 mL), and the combined organic layer was washed with brine, dried over sodium sulfate, filtered, and evaporated *in vacuo* to afford the crude product, which was purified using automated column chromatography (pentane/EtOAc) to afford the *N*-(2-bromophenyl)pivalamide in 78% yield.

***N*-(2-bromophenyl)pivalamide (5a):**[8]

Following the general procedure G using *N*-phenylpivalamide (39 mg, 0.22 mmol,1 equiv.), BBr_3 (0.26 mmol, 1.2 equiv., 1M in CH_2Cl_2), at 22 ºC for 2 h and Selectfluor (0.44 mmol, 2 equiv.), TBAB (0.48 mmol, 2.2 equiv.) and step iii) at 22 ºC for 2.5 h. The crude product was purified by automated column chromatography (pentane/EtOAc, 93:7) and the desired product was obtained as an off white solid (46 mg, 82%); **Rf:** 0.37 (hexane/EtOAc, 95:5)**; ^{1}H NMR (400 MHz, $CDCl_3$) δ=** 8.39 (dd, *J* = 8.3, 1.6 Hz, 1H), 8.01 (bs, 1H), 7.52 (dd, *J* = 8.0, 1.5 Hz, 1H), 7.31 (ddd, *J* = 8.7, 7.2, 1.5 Hz, 1H), 6.96 (ddd, *J* = 8.1, 7.4, 1.6 Hz, 1H), 1.35 (s, 9H); **^{13}C{^{1}H} NMR (101 MHz, $CDCl_3$) δ=** 176.8, 136.0, 132.2, 128.5, 125.0, 121.7, 113.7, 40.3, 27.7.

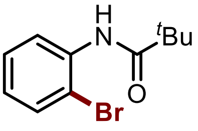

5a

4.6 General Procedure H for reaction optimization:Bromination of benzanilide derivatives

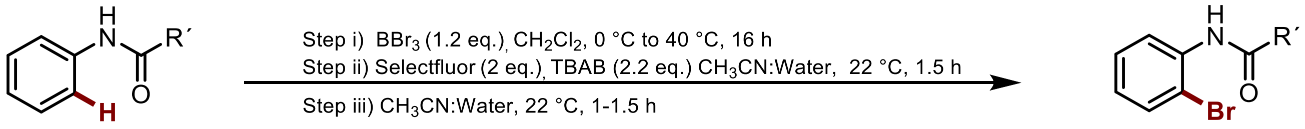

Step i) To a dry 5 mL, screw-top V-Vial, (Sigma Aldrich, Product code-Z115150-12EA), equipped with a rubber septum, stir bar, the amide derivative (0.22 mmol, 1 equiv.) in anhydrous CH_2Cl_2 (0.5 mL) under a nitrogen atmosphere was added dropwise BBr_3 (0.26 mmol, 1.2 equiv., 1M solution in CH_2Cl_2). After the complete addition of BBr_3, the reaction mixture was stirred at 40 ºC for 16 h after which the solvent was removed under reduced pressure.

Step ii) Simultaneously, selectfluor (0.44 mmol, 2 equiv.), and TBAB (0.48 mmol, 2.2 equiv.), were dissolved in CH_3CN (1 mL) and water (0.5 mL) in a 3 mL, screw-top V-vial and the reaction mixture stirred at 22 ºC for 1.5 h.

Step iii) The crude residue from step i) was dissolved in CH_3CN (0.5 mL) and water (0.5 mL) at 0 ºC. To this mixture, the solution from step ii) was added dropwise at 0 ºC, and the reaction mixture was stirred at 22 ºC for 1-1.5 h. The reaction was quenched with saturated sodium thiosulfite solution at room temperature and the crude mixture was dissolved in EtOAc (10 mL) and H_2O (10 mL). The aqueous layer was washed with EtOAc (10 mL), and the combined organic layer was washed with brine, dried over sodium sulfate, filtered, and evaporated *in vacuo* to afford the crude product, which was purified using automated column chromatography (pentane/EtOAc).

***N*-(2-bromophenyl)benzamide (8a):**[11]

Following the general procedure H using *N*-phenylbenzamide (44 mg, 0.22 mmol,1 equiv.), BBr_3 (0.26 mmol, 1.2 equiv., 1M in CH_2Cl_2), at 40 ºC for 16 h and Selectfluor (0.44 mmol, 2 equiv.), TBAB (0.48 mmol, 2.2 equiv.) at 22 ºC for 1 h. The crude product was purified by automated column chromatography (pentane/EtOAc, 94:6) and the desired product was obtained as an off white solid (45 mg, 73%); **Rf:** 0.25

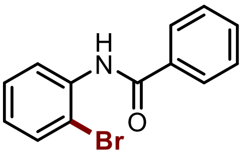

8a

(hexane/EtOAc, 95:5); **^{1}H NMR (400 MHz, $CDCl_3$) δ=** 8.56 (dd, *J* = 8.3, 1.6 Hz, 1H), 8.48 (s, 1H), 7.97 – 7.91 (m, 2H), 7.61 – 7.56 (m, 2H), 7.55 – 7.50 (m, 2H), 7.38 (ddd, *J* = 8.6, 7.4, 1.5 Hz, 1H), 7.02 (ddd, *J* = 8.0, 7.4, 1.6 Hz, 1H); **^{13}C{^{1}H} NMR (101 MHz, $CDCl_3$) δ=** 165.4, 135.9, 134.7, 132.4, 132.3, 129.1, 128.7, 127.2, 125.4, 121.8, 113.9.

***N*-(2-bromophenyl)-4-methylbenzamide (8b):**[12]

Following the general procedure H using 4-methyl-*N*-phenylbenzamide (47 mg, 0.22 mmol,1 equiv.), BBr_3 (0.26 mmol, 1.2 equiv., 1M in CH_2Cl_2), at 40 ºC for 16 h and Selectfluor (0.44 mmol, 2 equiv.), TBAB (0.48 mmol, 2.2 equiv.) at 22 ºC for 1 h. The crude product was purified by automated column chromatography (pentane/EtOAc, 94:6) and the desired product was obtained as an off white solid (48 mg, 74%); **Rf:** 0.28 (hexane/EtOAc, 95:5); **^{1}H NMR (400 MHz, $CDCl_3$) δ=** 8.47 (dd, *J* = 8.2, 1.6 Hz, 1H), 8.27 (bs, 1H), 7.87 (d, *J* = 7.9 Hz, 2H), 7.81 (dd, *J* = 8.0, 1.5 Hz, 1H), 7.44 – 7.37 (m, 1H), 7.33 (d, *J* = 7.9 Hz, 2H), 6.88 (td, *J* = 7.6, 1.6 Hz, 1H), 2.44 (s, 3H); **^{13}C{^{1}H} NMR (101 MHz, $CDCl_3$) δ=** 165.4, 142.9, 138.9, 138.5, 131.8, 129.8, 129.5, 127.3, 126.0, 121.8, 90.3, 21.7.

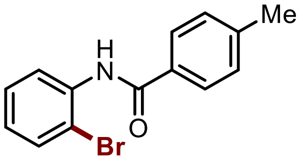

8b

***N*-(2-bromophenyl)-3-methylbenzamide (8c):**[22]

Following the general procedure H using 3-methyl-*N*-phenylbenzamide (47 mg, 0.22 mmol,1 equiv.), BBr_3 (0.26 mmol, 1.2 equiv., 1M in CH_2Cl_2), at 40 ºC for 16 h and Selectfluor (0.44 mmol, 2 equiv.), TBAB (0.48 mmol, 2.2 equiv.) at 22 ºC for 1 h. The crude product was purified by automated column chromatography (pentane/EtOAc, 94:6) and the desired product was obtained as a off white solid (46 mg, 71%); **Rf:** 0.33 (hexane/EtOAc, 95:5); ^{1}H NMR (400 MHz, $CDCl_3$) δ= 8.55 (dd, *J* = 8.3, 1.6 Hz, 1H), 8.45 (bs, 1H), 7.76 (s, 1H), 7.74 – 7.66 (m, 1H), 7.58 (dd, *J* = 8.0, 1.5 Hz, 1H), 7.38 (q, *J* = 6.5 Hz, 3H), 7.01 (td, *J* = 7.7, 1.6 Hz, 1H), 2.45 (s, 3H); **^{13}C{^{1}H} NMR (101 MHz, $CDCl_3$) δ=** 165.6, 139.0, 136.0, 134.7, 133.1, 132.4, 128.9, 128.7, 128.1, 125.3, 124.1, 121.9, 113.8, 21.6.

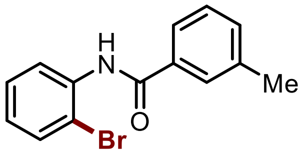

8c

***N*-(2-bromophenyl)-4-fluorobenzamide (8d):**[23]

Following the general procedure H using 4-fluoro-*N*-phenylbenzamide (47 mg, 0.22 mmol,1 equiv.), BBr_3 (0.26 mmol, 1.2 equiv., 1M in CH_2Cl_2), at 40 ºC for 16 h and Selectfluor (0.44 mmol, 2 equiv.), TBAB (0.48 mmol, 2.2 equiv.) at 22 ºC for 1 h. The crude product was purified by automated column chromatography (pentane/EtOAc, 94:6) and the desired product was obtained as an off white solid (45.5 mg, 71%); **Rf:** 0.25 (hexane/EtOAc, 95:5); **^{1}H NMR (400 MHz, $CDCl_3$) δ=** 8.51 (dd, *J* = 8.3, 1.6 Hz, 1H), 8.39 (bs, 1H), 8.01 – 7.91 (m, 2H), 7.58 (dd, *J* = 8.0, 1.5 Hz, 1H), 7.42 – 7.33 (m, 1H), 7.20 (t, *J* = 8.6 Hz, 2H), 7.02 (td, *J* = 7.7, 1.6 Hz, 1H); **^{13}C{^{1}H} NMR (101 MHz, $CDCl_3$) δ=** 165.2 (d, *J* = 253.5

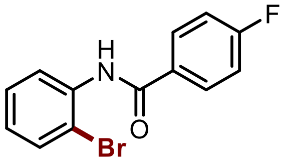

8d

Hz), 164.3, 135.8, 132.4, 130.9 (d, J = 3.1 Hz), 129.6 (d, J = 9.0 Hz), 128.7, 125.5, 121.9, 116.2 (d, J = 22.1 Hz), 113.9; **^{19}F NMR (470 MHz, $CDCl_3$) δ=** -106.79.

2-bromo-*N*-(2-bromophenyl)benzamide (8f): (14)

Following the general procedure H using 2-bromo-*N*-phenylbenzamide (60.3 mg, 0.22 mmol,1 equiv.), BBr_3 (0.26 mmol, 1.2 equiv., 1M in CH_2Cl_2), at 40 °C for 16 h and Selectfluor (0.44 mmol, 2 equiv.), TBAB (0.48 mmol, 2.2 equiv.) at 22 °C for 1 h. The crude product was purified by automated column chromatography (pentane/EtOAc, 92:8) and the desired product was obtained as an off white solid (33 mg, 42%); **Rf:** 0.2 (hexane/EtOAc, 95:5); **^{1}H NMR (700 MHz, $CDCl_3$) δ=** 8.54 (d, J = 8.3 Hz, 1H), 8.16 (bs, 1H), 7.67 (dt, J = 8.1, 1.7 Hz, 2H), 7.58 (dd, J = 8.0, 1.5 Hz, 1H), 7.44 (t, J = 7.0 Hz, 1H), 7.41 – 7.37 (m, 1H), 7.35 (td, J = 7.7, 1.7 Hz, 1H), 7.04 (td, J = 7.7, 1.6 Hz, 1H); **^{13}C{^{1}H} NMR (176 MHz, $CDCl_3$) δ=** 165.6, 137.7, 135.6, 133.9, 132.6, 132.0, 129.8, 128.6, 127.9, 125.8, 122.2, 119.5, 113.8.

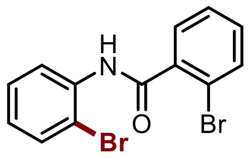

8f

***N*-(2-bromophenyl)-4-(trifluoromethyl)benzamide (8h):** (24)

Following the general procedure H using *N*-phenyl-4-(trifluoromethyl)benzamide (58 mg, 0.22 mmol,1 equiv.), BBr_3 (0.26 mmol, 1.2 equiv., 1M in CH_2Cl_2), at 40 °C for 16 h and Selectfluor (0.44 mmol, 2 equiv.), TBAB (0.48 mmol, 2.2 equiv.) and step iii) at 40 °C for 1.5 h. The crude product was purified by automated column chromatography (pentane/EtOAc, 94:6) and the desired product was obtained as a off white solid (31 mg, 41%); **Rf:** 0.27 (hexane/EtOAc, 95:5); **^{1}H NMR (400 MHz, $CDCl_3$) δ=** 8.53 (dd, J = 8.3, 1.6 Hz, 1H), 8.46 (bs, 1H), 8.05 (d, J = 8.1 Hz, 2H), 7.79 (d, J = 8.2 Hz, 2H), 7.60 (dd, J = 8.1, 1.5 Hz, 1H), 7.40 (td, J = 7.8, 1.5 Hz, 1H), 7.06 (td, J = 7.7, 1.6 Hz, 1H); **^{13}C{^{1}H} NMR (101 MHz, $CDCl_3$) δ=** 164.1, 138.0, 135.5, 134.0 (q, J = 32.8 Hz), 132.5, 128.8, 127.7, 126.2 (q, J = 3.7 Hz), 125.9, 123.7 (q, J = 272.6 Hz) 122.0, 114.0; **^{19}F NMR (470 MHz, $CDCl_3$) δ=** -63.03.

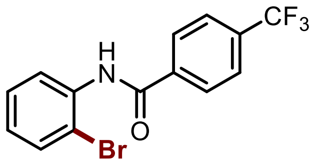

8h

***N*-(2-bromophenyl)-3-nitrobenzamide (8i):** (22)

Following the general procedure H using 3-nitro-*N*-phenylbenzamide (53.4 mg, 0.22 mmol,1 equiv.), BBr_3 (0.26 mmol, 1.2 equiv., 1M in CH_2Cl_2), at 40 °C for 16 h and Selectfluor (0.44 mmol, 2 equiv.), TBAB (0.48 mmol, 2.2 equiv.) at 22 °C for 1 h. The crude product was purified by automated column chromatography (pentane/EtOAc, 85:15) and the desired product was obtained as an off white solid (57 mg, 81%); **Rf:** 0.17 (hexane/EtOAc, 90:10); **^{1}H NMR (400 MHz, $CDCl_3$) δ=** 8.78 (t, J = 2.0 Hz, 1H), 8.47 (dd, J = 8.1, 1.6 Hz, 2H), 8.44 (ddd, J = 8.2, 2.3, 1.0 Hz, 1H), 8.26 (ddd, J = 7.8, 1.8, 1.0 Hz, 1H), 7.74 (t, J = 8.0 Hz, 1H), 7.60 (dd, J = 8.0, 1.4 Hz, 1H), 7.39 (ddd, J = 8.6, 7.5, 1.5 Hz, 1H), 7.07 (ddd, J = 8.0, 7.4, 1.6 Hz, 1H); **^{13}C{^{1}H} NMR (101 MHz, $CDCl_3$) δ=** 163.0, 148.6, 136.3, 135.2, 133.0, 132.5, 130.4, 128.8, 126.8, 126.2, 122.4, 122.2, 114.3.

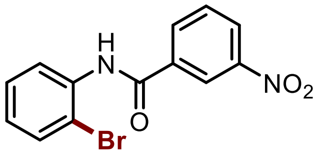

8i

N-(2-bromophenyl)furan-2-carboxamide (8j):[25]

Following the general procedure H using *N*-phenylfuran-2-carboxamide (41 mg, 0.22 mmol,1 equiv.), BBr_3 (0.26 mmol, 1.2 equiv., 1M in CH_2Cl_2), at 40 ºC for 16 h and Selectfluor (0.44 mmol, 2 equiv.), TBAB (0.48 mmol, 2.2 equiv.) at 22 ºC for 1 h. The crude product was purified by automated column chromatography (pentane/EtOAc, 94:6) and the desired product was obtained as an off white solid (43 mg, 74%); **Rf:** 0.25 (hexane/EtOAc, 95:5); **^{1}H NMR (400 MHz, CDCl$_3$) δ=** 8.71 (bs, 1H), 8.50 (dt, *J* = 8.3, 1.8 Hz, 1H), 7.57 (dq, *J* = 8.0, 1.4 Hz, 2H), 7.39 – 7.31 (m, 1H), 7.29 – 7.24 (m, 1H), 7.00 (dd, *J* = 7.9, 1.4 Hz, 1H), 6.58 (dq, *J* = 3.0, 1.6 Hz, 1H); **^{13}C{^{1}H} NMR (101 MHz, CDCl$_3$) δ=** 156.0, 147.8, 144.8, 135.5, 132.5, 128.6, 125.4, 121.7, 115.8, 113.6, 112.8.

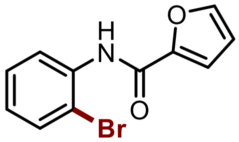

8j

N-(2-bromophenyl)thiophene-2-carboxamide (8k):[26]

Following the general procedure H using *N*-phenylthiophene-2-carboxamide (44 mg, 0.22 mmol,1 equiv.), BBr_3 (0.26 mmol, 1.2 equiv., 1M in CH_2Cl_2), at 40 ºC for 16 h and Selectfluor (0.44 mmol, 2 equiv.), TBAB (0.48 mmol, 2.2 equiv.) at 22 ºC for 1 h. The crude product was purified by automated column chromatography (pentane/EtOAc, 94:6) and the desired product was obtained as an off white solid (46 mg, 75%); **Rf:** 0.27 (hexane/EtOAc, 95:5); **^{1}H NMR (400 MHz, CDCl$_3$) δ=** 8.48 (dd, *J* = 8.3, 1.6 Hz, 1H), 8.34 (bs, 1H), 7.68 (dd, *J* = 3.8, 1.2 Hz, 1H), 7.61 – 7.53 (m, 2H), 7.36 (td, *J* = 7.9, 1.5 Hz, 1H), 7.16 (dd, *J* = 5.0, 3.7 Hz, 1H), 7.01 (td, *J* = 7.7, 1.6 Hz, 1H); **^{13}C{^{1}H} NMR (101 MHz, CDCl$_3$) δ=** 159.7, 139.2, 135.6, 132.4, 131.5, 128.7, 128.7, 128.1, 125.4, 121.7, 113.6.

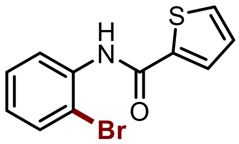

8k

5. Applications

Synthesis of 3,3-dimethyl-3,4-dihydroquinolin-2(1H)-one (9a)[27]

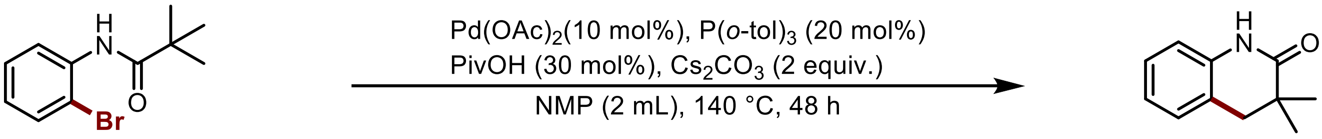

5a **9a 43%**

To a 10 mL seal tube, $Pd(OAc)_2$ (4.5 mg, 0.02 mmol), P(*o*-tol)$_3$ (12.2 mg, 0.04 mmol), Cs_2CO_3 (130.3 mg, 0.40 mmol) were added respectively. Then PivOH (6.1 mg, 0.06 mmol, ~ 6.7 µL) was added to the tube with a micro injector. A solution of N-(2-bromophenyl)pivalamide (51 mg, 0.20 mmol) in NMP (2.0 mL) was added by a syringe and the reaction was stirred at 140 ºC for 48 hours. After the reaction was finished, the reaction mixture was cooled to room temperature and filtered through a celite pad using EtOAc as the eluent. The reaction mixture was concentrated under reduced pressure, and the resulting mixture was purified by automated column chromatography (pentane/EtOAc, 80:20) and the desired product was obtained as a beige color solid (43%).

Spectral data: ^{1}H NMR (700 MHz, CDCl$_3$) δ= 7.94 (bs, 1H), 7.17 (td, *J* = 7.6, 1.5 Hz, 1H), 7.14 (d, *J* = 7.5 Hz, 1H), 6.98 (td, *J* = 7.4, 1.2 Hz, 1H), 6.74 (dd, *J* = 7.9, 1.2 Hz, 1H), 2.80 (s, 2H), 1.21 (s, 6H); **^{13}C{^{1}H} NMR (176 MHz, CDCl$_3$) δ=** 176.7, 137.0, 128.6, 127.5, 123.5, 123.1, 114.7, 40.4, 37.5, 24.5.

Synthesis of methyl 11-oxo-5,11-dihydrobenzo[4,5]imidazo[1,2-*b*]isoquinoline-6-carboxylate (9b)[28]

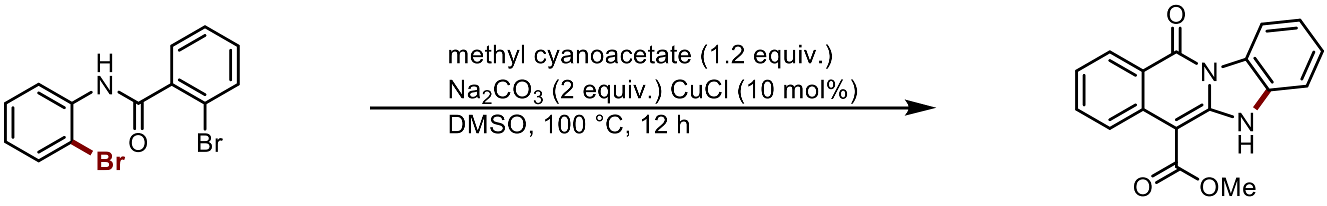

8f **9b 51%**

To a 10 mL seal tube, 2-bromo-N-(2-bromophenyl)benzamide (35.5 mg, 0.1 mmol,) methyl cyanocacetate (0.12 mmol), Na_2CO_3 (0.2 mmol) were dissolved in 1 mL DMSO and the mixture was stirred at room temperature for 10 min. Then CuCl (0.01 mmol) was added, and the mixture was stirred at 100 ºC for 12 h. After comforming with the TLC, the mixture was filtered, washed with ethyl acetate and concentrated under reduced pressure, and the resulting mixture was purified by automated column chromatography (pentane/EtOAc, 80:20) and the desired product was obtained as a beige color solid (51%).

Spectral data: ^{1}H NMR (400 MHz, CDCl$_3$) δ= 11.25 (bs, 1H), 8.76 (dd, *J* = 15.1, 8.3 Hz, 2H), 8.57 (dd, *J* = 8.2, 1.6 Hz, 1H), 7.72 (ddd, *J* = 8.6, 6.9, 1.6 Hz, 1H), 7.47 (t, *J* = 7.0 Hz, 1H), 7.42 – 7.33 (m, 3H),

4.06 (s, 3H); **$^{13}C\{^1H\}$ NMR (101 MHz, $CDCl_3$) δ=** 168.9, 160.2, 147.0, 135.5, 133.6, 131.0, 128.3, 128.0, 126.6, 124.6, 123.5, 122.9, 119.5, 117.3, 110.2, 82.6, 51.8.

Synthesis of 14*H*-dibenzo[4,5:6,7]azepino[2,1-*a*]isoindol-14-one (9c)[(29)]

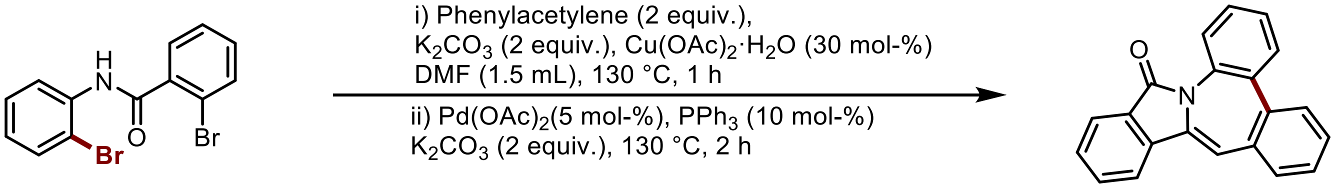

8f **9c 76%**

To a oven-dried 10 mL round-bottomed flask, 2-bromo-N-(2-bromophenyl)benzamide (35.5 mg, 0.1 mmol,) phenylacetylene (22 µl, 0.2 mmol), $Cu(OAc)_2 \cdot H_2O$ (6.0 mg, 30 mol-%), K_2CO_3 (27.6 mg, 0.2 mmol) were dissolved in 1 mL DMF and the mixture was stirred at 130 °C for 1 h. Then $Pd(OAc)_2$ (1.1 mg, 5 mol-%) and PPh_3 (2.6 mg, 10 mol-%) were added to the reaction mixture at ambient temperature and stirring was continued while heating at 130 °C for another 2 h. After 2 h, the reaction mixture was cooled to room temperature, diluted with water (10 mL) and extracted with EtOAc (2 × 10 mL). The combined organic layers were dried with anhydrous sodium sulfate and concentrated under reduced pressure. The resulting mixture was purified by automated column chromatography (pentane/EtOAc, 85:15) and the desired product was obtained as a off white solid (22.5 mg, 76%).

Spectral data: ^1H NMR (400 MHz, $CDCl_3$) δ= 7.96 (d, *J* = 7.6 Hz, 1H), 7.77 (dd, *J* = 8.1, 1.4 Hz, 1H), 7.73 (d, *J* = 7.8 Hz, 1H), 7.65 (t, *J* = 8.1 Hz, 1H), 7.58 – 7.50 (m, 2H), 7.39 (dd, *J* = 7.7, 1.9 Hz, 1H), 7.35 (dd, *J* = 8.1, 1.9 Hz, 1H), 7.29 (tt, *J* = 7.1, 2.1 Hz, 3H), 7.17 (dd, *J* = 7.2, 1.9 Hz, 1H), 6.58 (s, 1H); **$^{13}C\{^1H\}$ NMR (101 MHz, $CDCl_3$) δ=** 168.9, 144.6, 140.6, 138.4, 137.0, 135.7, 133.5, 132.9, 132.9, 130.5, 129.9, 129.3, 128.9, 128.6, 128.5, 128.2, 127.1, 124.0, 123.9, 118.6, 112.3.

Synthesis of (9*H*-carbazol-9-yl)(phenyl)methanone (9d)[(30)]

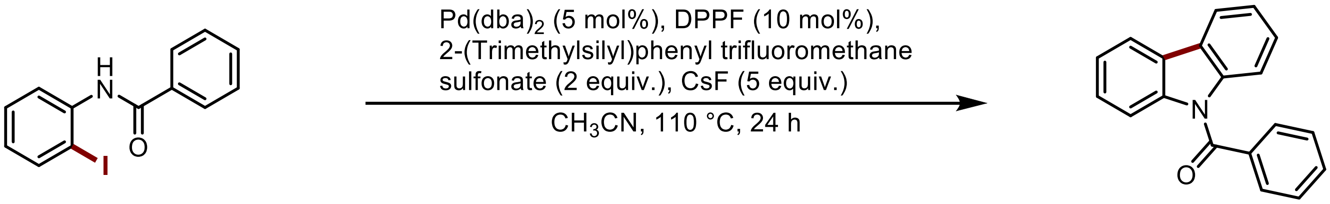

7a **9d 50%**

To the seal tube, N-(2-iodophenyl)benzamide (7a) (80.78 mg, 0.25 mmol, 1 equiv.), 2-(Trimethylsilyl) phenyltrifluoromethane sulfonate (121.4 µl, 0.5 mmol, 2.0 equiv.), CsF (190 mg, 1.25 mmol, 5.0 equiv.), $Pd(dba)_2$ (7.19 mg, 5 mol %), dppf (14 mg, 10 mol %), 8 mL of toluene, and 2 mL of MeCN were taken under nitrogen. The reaction mixture was stirred, first, at room temperature for 1 min and then heated to 110 °C for 24 h. The mixture was allowed to cool to room temperature, diluted with ethyl acetate, washed

with brine, dried over anhydrous MgSO4, and concentrated under reduced pressure. The resulting mixture was purified by automated column chromatography (pentane/EtOAc, 90:10) and the desired product was obtained as a beige color solid (50%).

Spectral data: ^{1}H NMR (700 MHz, CDCl$_3$) δ= 8.02 (dd, *J* = 7.4, 1.9 Hz, 2H), 7.73 (dd, *J* = 7.9, 1.4 Hz, 2H), 7.67 – 7.64 (m, 1H), 7.55 – 7.51 (m, 4H), 7.36 (td, *J* = 7.4, 1.2 Hz, 2H), 7.33 (td, *J* = 7.7, 1.5 Hz, 2H); **^{13}C{^{1}H} NMR (176 MHz, CDCl$_3$) δ=** 169.8, 139.3, 135.9, 132.5, 129.2, 129.0, 126.9, 126.2, 123.5, 120.0, 115.9.

Synthesis of 1-phenyl-1,3-dihydro-2H-benzo[d]imidazol-2-one (9e)(31)

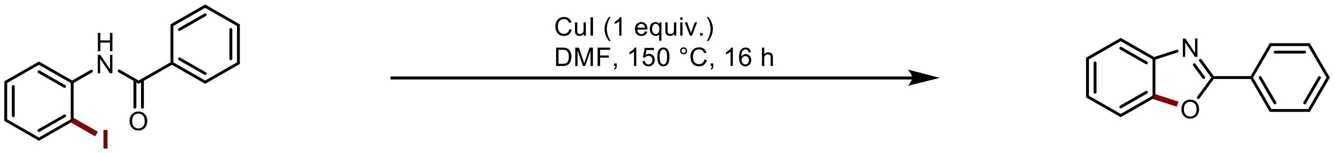

7a

9e 65%

To a 5 mL seal tube, N-(2-iodophenyl)benzamide (51 mg, 0.15 mmol), CuI (33.6 mg, 0.17 mmol), and DMF (1 mL) were added respectively. Then the reaction was stirred at 140 ^{o}C for 16 h. After the reaction was finished, the reaction mixture was cooled to room temperature and filtered through a celite pad using EtOAc as the eluent. The reaction mixture was concentrated under reduced pressure, and the resulting mixture was purified by automated column chromatography (pentane/EtOAc, 95:5) and the desired product was obtained as a an off white solid (65%).

Spectral data: ^{1}H NMR (700 MHz, CDCl$_3$) δ= 8.27 (dd, *J* = 7.2, 2.5 Hz, 2H), 7.79 (dd, *J* = 6.1, 3.2 Hz, 1H), 7.59 (d, *J* = 9.2 Hz, 1H), 7.53 (d, *J* = 6.8 Hz, 3H), 7.36 (dd, *J* = 6.0, 3.1 Hz, 2H); **^{13}C{^{1}H} NMR (176 MHz, CDCl$_3$) δ=** 163.2, 150.9, 142.2, 131.7, 129.1, 127.8, 127.3, 125.3, 124.7, 120.2, 110.7.

Synthesis of 5-chloro-2-(p-tolyl)benzo[d]oxazole (9f) (31)

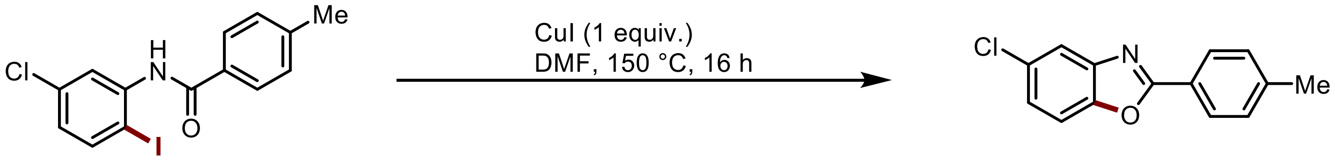

7l

9f 70%

To a 5 mL seal tube, N-(5-chloro-2-iodophenyl)-4-methylbenzamide (56 mg, 0.15 mmol), CuI (33.6 mg, 0.17 mmol), and DMF (1 mL) were added respectively. Then the reaction was stirred at 140 ^{o}C for 16 h. After the reaction was finished, the reaction mixture was cooled to room temperature and filtered through a celite pad using EtOAc as the eluent. The reaction mixture was concentrated under reduced pressure, and the resulting mixture was purified by automated column chromatography (pentane/EtOAc, 95:5) and the desired product was obtained as a an off white solid (70%).

Spectral data:[32] **^{1}H NMR (400 MHz, $CDCl_3$) δ=** 8.10 (d, *J* = 7.8 Hz, 2H), 7.71 (d, *J* = 2.1 Hz, 1H), 7.46 (dd, *J* = 8.6, 1.0 Hz, 1H), 7.32 (d, J = 8.0 Hz, 2H), 7.29 (ddd, *J* = 8.6, 2.1, 0.8 Hz, 1H), 2.43 (s, 3H); **$^{13}C\{^1H\}$ NMR (101 MHz, $CDCl_3$) δ=** 164.7, 149.4, 143.4, 142.7, 130.0, 129.8, 127.8, 125.2, 124.0, 119.9, 111.3, 21.8.

Synthesis of 1-phenyl-1,3-dihydro-2H-benzo[d]imidazol-2-one (9g)[33]

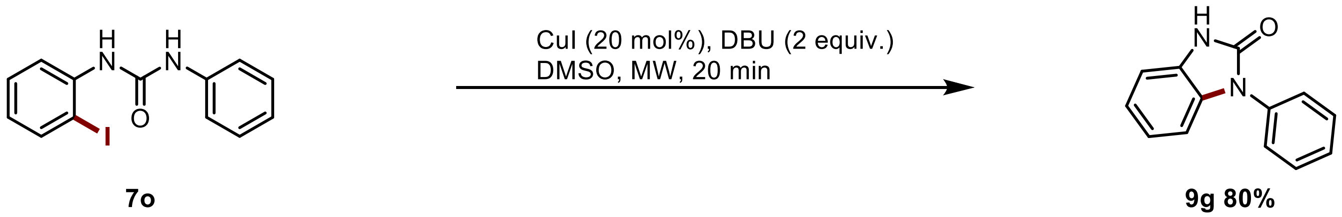

To a microwave vial, 1-(2-iodophenyl)-3-phenylurea (34 mg, 1.0 mmol), CuI (3.83 mg, 0.020 mmol), and DBU (29.85 µl, 0.2 mmol) were added respectively. DMSO (0.2 mL) was added by a syringe and the reaction was stirred at 120 ºC for 20 min under microwave irradiation. After the reaction was finished, the reaction mixture was cooled to room temperature and filtered through a celite pad using EtOAc as the eluent. mixture was purified by column chromatography using (pentane/EtOAc, 70:30) as the eluent. The reaction mixture was concentrated under reduced pressure, and the resulting mixture was purified by automated column chromatography (pentane/EtOAc, 70:30) and the desired product was obtained as a an off white solid (80%).

Spectral data: ^{1}H NMR (400 MHz, $CDCl_3$) δ= 10.54 (bs, 1H), 7.61 – 7.54 (m, 4H), 7.45 (dt, *J* = 5.1, 3.4 Hz, 1H), 7.17 (dt, *J* = 7.7, 1.0 Hz, 1H), 7.13 – 7.08 (m, 1H), 7.06 (dd, *J* = 4.1, 0.9 Hz, 2H); **$^{13}C\{^1H\}$ NMR (101 MHz, $CDCl_3$) δ=** 155.3, 134.5, 130.6, 129.8, 128.2, 128.1, 126.4, 122.4, 121.6, 110.2, 109.0.

Synthesis of 5-methyl-1-morpholino-7,7-diphenyl-5,7-dihydro-6*H*-dibenzo[*b*,*d*]azepin-6-one (9h)[34]

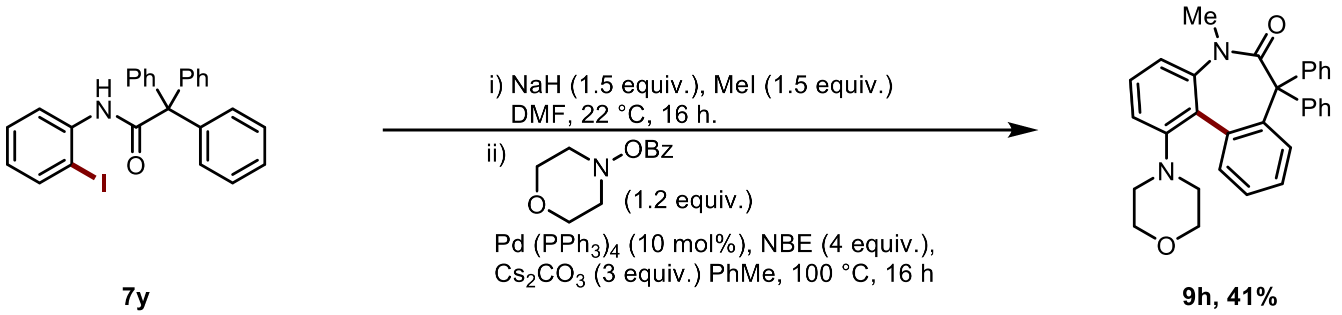

First step: In a 5 mL round bottom flask, *N*-(2-iodophenyl)-2,2,2-triphenylacetamide (36.36 mg, 0.01 mmol) was stirred in 2 mL dry DMF at 0 °C. Sodium hydride, 60% dispersion in mineral oil (6mg, 1.5 equiv.) was added followed by iodomethane (9.3 µl, 1.5 equiv.) five min. later and the reaction was stirred at room temperature for 16 h. The reaction was quenched with cold brine and extracted three times with

EtOAc. The organic layer was dried over sodium sulfate, filtered and concentrated under reduced pressure. The resulting mixture was purified by filteration through silica.

Second step: Vials and stir bars were dried in 110 °C oven overnight prior to use. Two dry 5 mL, screw-top V-Vial, (Sigma Aldrich, Product code-Z115150-12EA), one equipped with a stir bar and one without were cooled to room temperature while under argon flow. Norbornene (41.43 mg, 0.44 mmol) was weighed in the vial without stir bar. Cesium carbonate (97.76 mg, 0.3 mmol), *N*-(2-iodophenyl)-*N*-methyl-2,2,2-triphenylacetamide from first step, morpholinobenzoate (24.87 mg, 0.12 mmol) and tetrakis(triphenylphosphine)palladium(0) (11.56 mg, 0.01 mmol) were added in that order to the vial with stir bar. 2.2 mL of freshly distilled toluene were added via syringe to the norbornene-containing vial and the latter was sonicated for 15 s to make sure all the norbornene was fully dissolved. 2 mL of the resulting solution were transferred via syringe to the other vial. The vial was equipped with a Teflon-sealed cap and immediately stirred at 100 °C for 16 h. The reaction was passed through a silica pad washing with ethyl acetate. The filtrate was concentrated under reduced pressure and the resulting residue and the resulting mixture was purified by automated column chromatography (pentane/EtOAc, 85:15) and the desired product was obtained as a an off white solid (19 mg, 41%).

Spectral data: ^{1}H NMR (400 MHz, $CDCl_3$) δ= 8.23 (dd, *J* = 7.8, 1.5 Hz, 1H), 7.37 – 7.24 (m, 7H), 7.06 – 6.95 (m, 4H), 6.92 – 6.85 (m, 2H), 6.70 (dd, *J* = 7.5, 2.5 Hz, 1H), 6.67 (dd, *J* = 8.2, 1.1 Hz, 1H), 6.50 (dd, *J* = 8.1, 1.1 Hz, 1H), 3.62 – 3.53 (m, 4H), 3.40 (s, 3H), 2.65 (m, 2H), 2.53 (m, 2H); **^{13}C{^{1}H} NMR (101 MHz, $CDCl_3$) δ=** 173.6, 149.0, 142.9, 142.8, 141.6, 139.4, 134.2, 131.5, 130.0, 128.78 128.7, 128.5, 128.1, 127.6, 127.4, 127.4, 126.9, 126.9, 126.5, 126.1, 116.5, 115.0, 67.3, 66.8, 51.0, 39.2.

6. Synthesis of difluoro boracycle (4a)

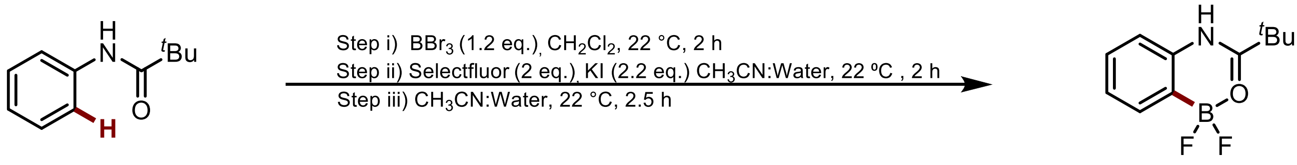

1a **4a**

Step i) To a dry 5 mL, screw-top V-Vial, (Sigma Aldrich, Product code-Z115150-12EA), equipped with a rubber septum, stir bar, the amide derivative (0.22 mmol, 1 equiv.) in anhydrous CH_2Cl_2 (0.5 mL) under a nitrogen atmosphere was added dropwise BBr_3 (0.26 mmol, 1.2 equiv., 1M solution in CH_2Cl_2). After the complete addition of BBr_3, the reaction mixture was stirred at 22 ºC for 2 h after which the solvent was removed under reduced pressure.

Step ii) Simultaneously, Selectfluor (0.44 mmol, 2 equiv.), and KI (0.48 mmol, 2.2 equiv.), were dissolved in CH_3CN (1 mL) and water (1 mL) in a 3 mL, screw-top V-vial and the reaction mixture stirred at 22 ºC for 2 h.

Step iii) The crude residue from step i) was dissolved in CH_3CN (0.5 mL). To this mixture, the solution from step ii) was added dropwise at 22 ºC, and the reaction mixture was heated at 22 ºC for 2.5 h. The reaction was quenched with saturated sodium thiosulfite solution at room temperature and the crude mixture was dissolved in EtOAc (10 mL) and H_2O (10 mL). The aqueous layer was washed with EtOAc (10 mL), and the combined organic layer was washed with brine, dried over sodium sulfate, filtered, and evaporated *in vacuo* to afford the crude product, which was purified using automated column chromatography (pentane/EtOAc, 80:20) to afford an off white solid (20 mg, 40%).

Spectral data: Rf: 0.41 (hexane/EtOAc, 50:50); **M.P:** 235-237 ºC; **^{1}H NMR (600 MHz, dmso-d^6) δ=** 11.75 (s, 1H), 7.45 (d, *J* = 8.1 Hz, 2H), 7.37 (td, *J* = 7.6, 1.6 Hz, 1H), 7.28 (td, *J* = 7.3, 1.1 Hz, 1H), 1.37 (s, 9H); **^{13}C{^{1}H} NMR (151 MHz, dmso-d^6) δ=** 178.0, 136.5, 130.6, 128.2, 127.0, 117.0, 38.5, 26.6; **^{19}F NMR (564 MHz, dmso-d^6) δ=** -129.60 (d, *J* = 40.8 Hz); **^{11}B NMR (193 MHz, dmso-d^6) δ=** 2.38; **HRMS (ESI) (m/z):** calculated for $[M-H]^-$ $C_{11}H_{14}BF_2NO^+$ 224.1058; found 224.1069.

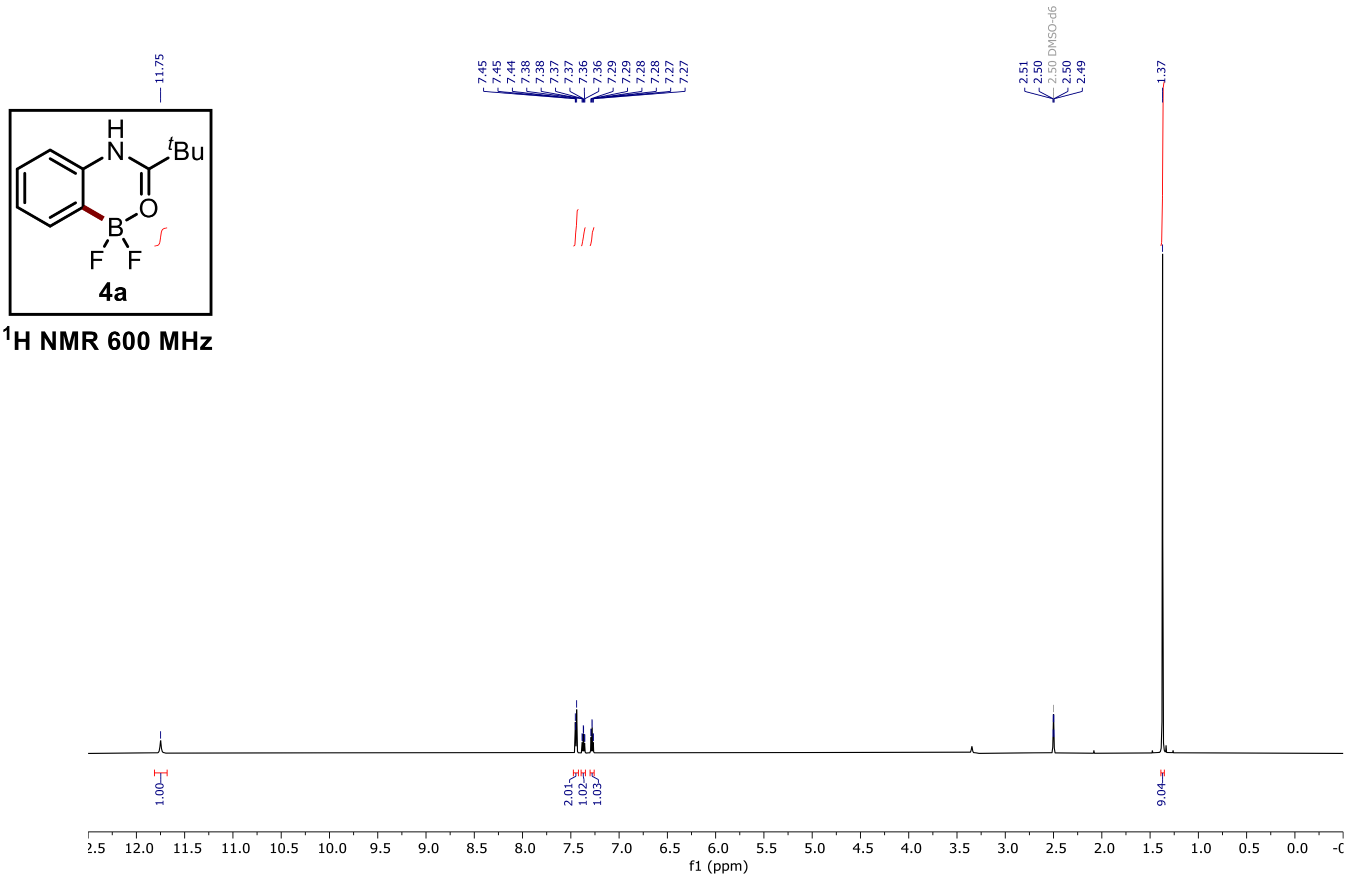

^{1}H NMR 600 MHz

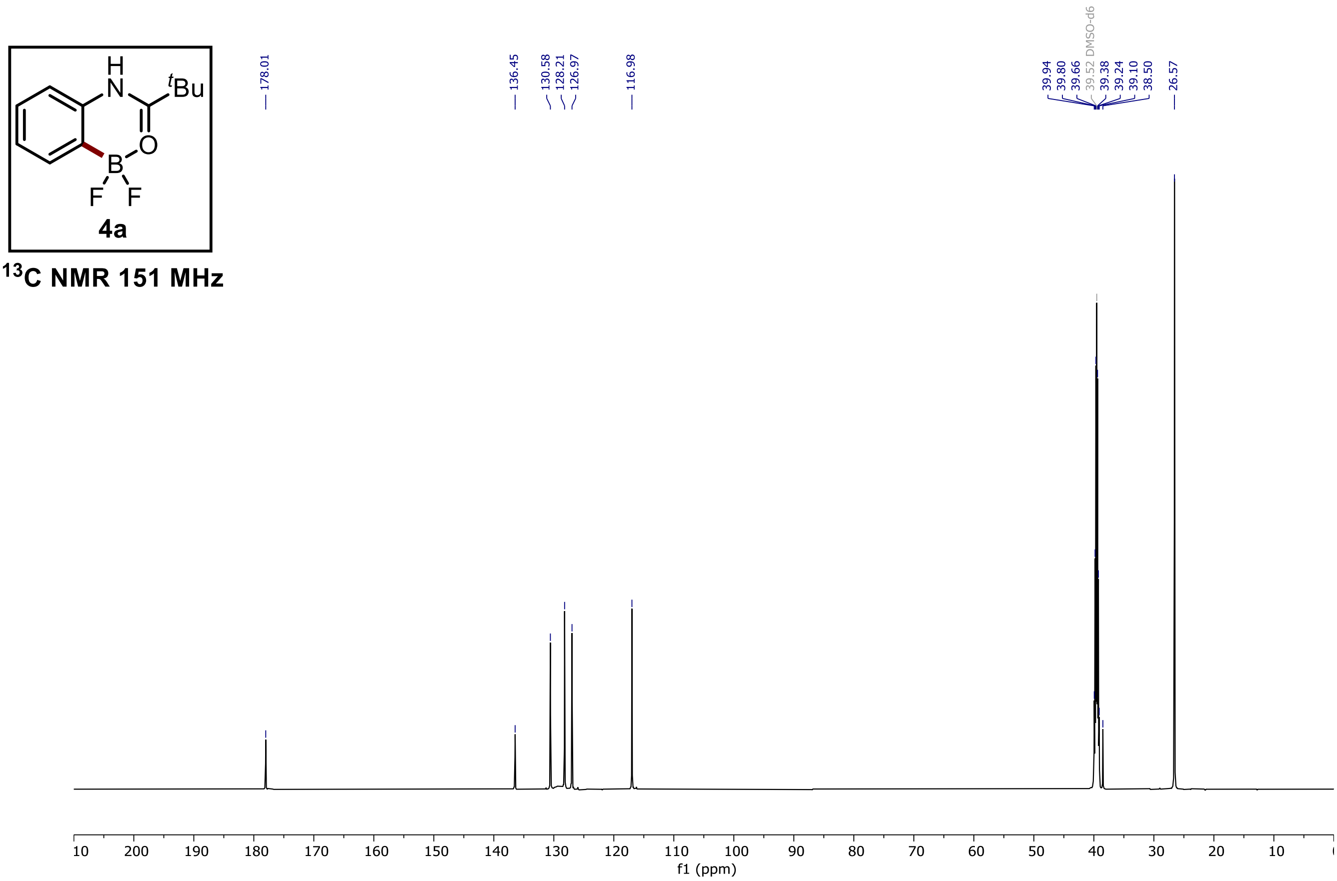

^{13}C NMR 151 MHz

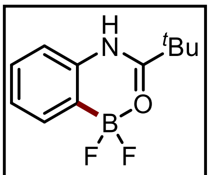

4a

^{19}F NMR 564 MHz

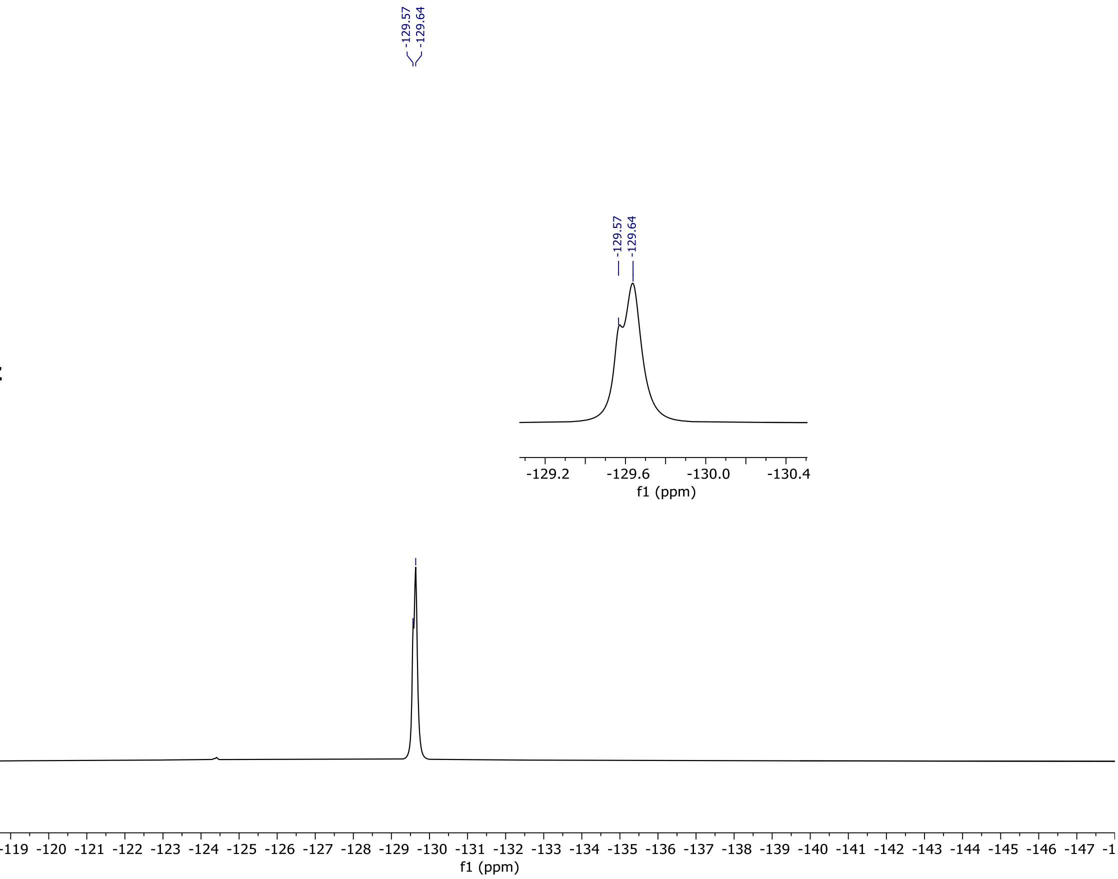

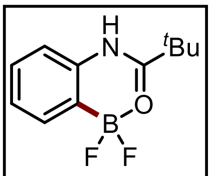

4a

^{11}B NMR 193 MHz

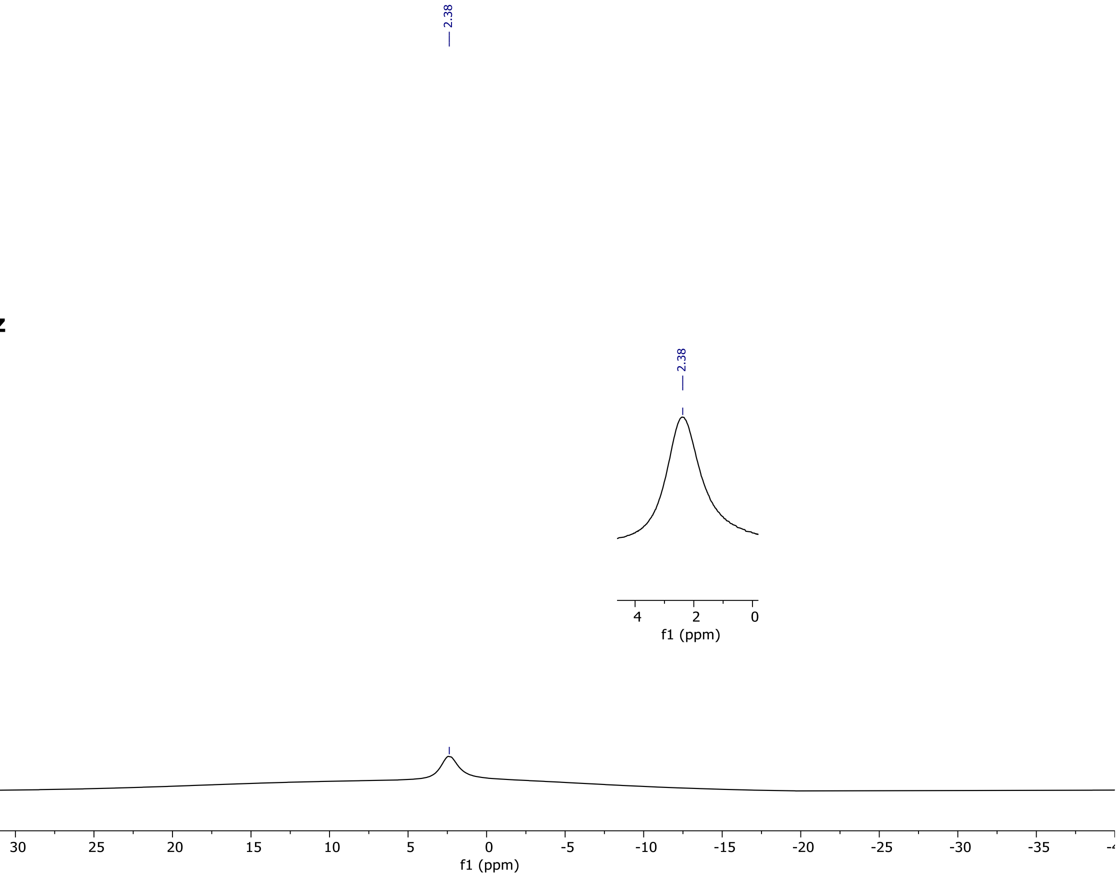

7. Gram scale experiment

Synthesis of N-(2-iodophenyl)pivalamide (3a)

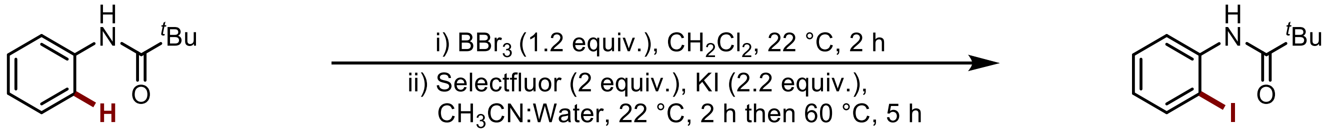

Step i) To a dry 50 mL, seal tube equipped with a rubber septum, stir bar, under a nitrogen atmosphere, the N-phenylpivalamide (1g, 5.64 mmol, 1 equiv.), was taken in anhydrous CH_2Cl_2 (10 mL). To this mixture, BBr_3 (6.77 mL, 6.77 mmol, 1.2 equiv., 1M solution in CH_2Cl_2), was added dropwise. After the complete addition of BBr_3, the reaction mixture was stirred at 22 ºC for 2 h. The solvent was evaporated under reduced pressure.

In the meantime, step ii) was set up in a 25 mL round bottom flask, in which Selectfluor (11.28 mmol, 2 equiv.), and KI (12.41 mmol, 2.2 equiv.), were dissolved in CH_3CN (15 mL) and water (15 mL) and the reaction mixture stirred at 22 ºC for 2 h.

Step iii) The crude residue from step i) was dissolved in CH_3CN (5 mL). To this residual mixture, step ii) solution was added dropwise at 22 ºC, and the reaction mixture was heated at 60 ºC for 5 h. The reaction was quenched with sat. sodium thiosulfite solution at room temperature and the crude mixture was dissolved in EtOAc (50 mL) and H_2O (50 mL). The aqueous layer was washed with EtOAc (2 x 30 mL), and the combined organic layer was washed with brine, dried over sodium sulfate, filtered, and evaporated *in vacuo* to afford the crude product, which was purified using automated column chromatography (pentane/EtOAc, 90:10).

8. Computational details

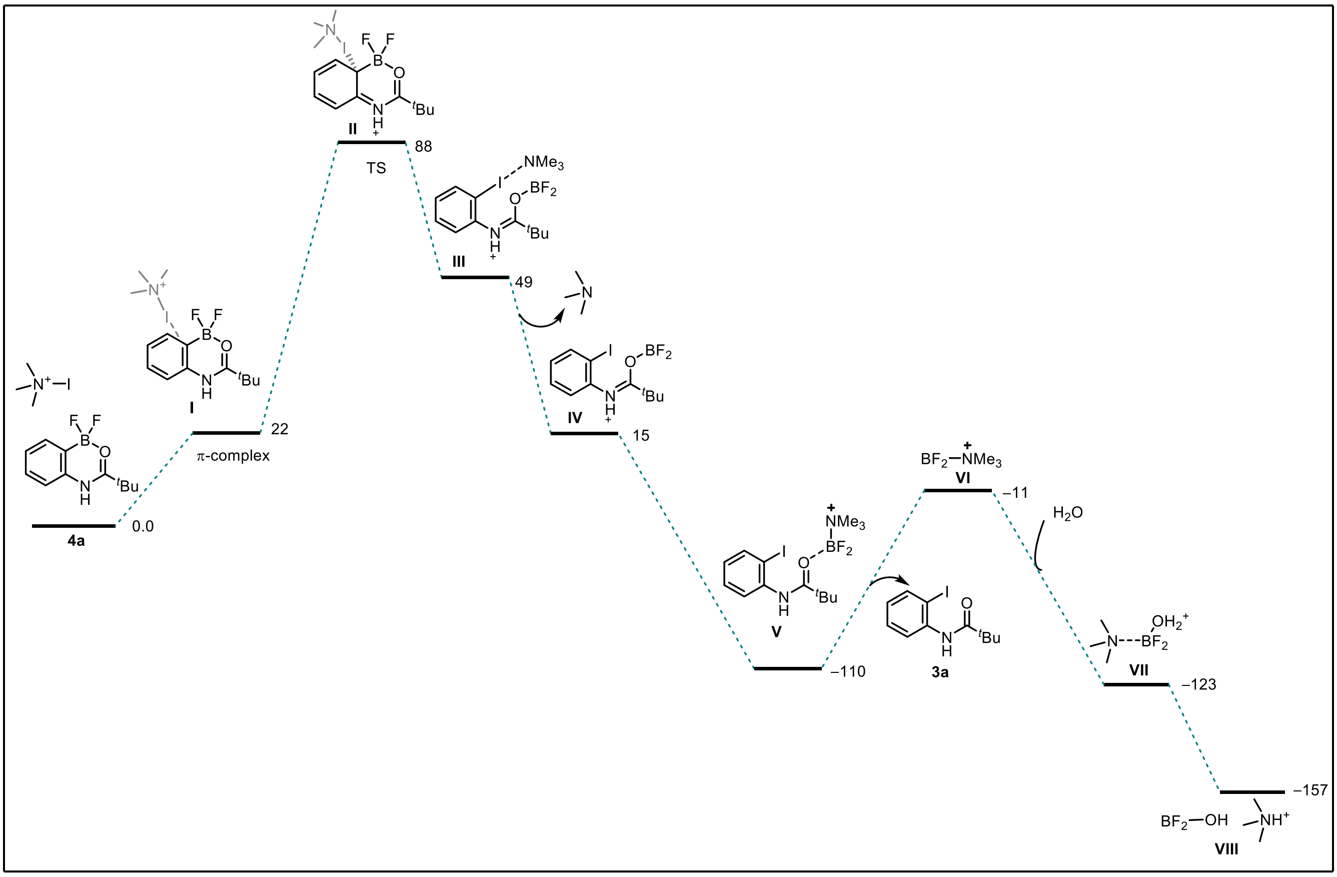

Neutral water

File: jag_H₂O_opt_wB97X-D_LACVPs/jag_H2O_opt_wB97X-D_LACVPs.out; structure: final

Method: DFT(wb97x-d)/lacvp*; Solvent: water

Total Quantum Mechanical Energy: -76.4227396101 au

Solution phase energy: -76.39989734098 au

Gibbs free energy: -76.396899 au

Low frequencies 1788.02 3740.03 3832.78

O1	-0.0671053953	-0.0078280998	0.0000000000
H2	0.6204661612	-0.6917206505	0.0000000000
H3	0.4445451591	0.8159582756	0.0000000000

Iodine carrier model, cationic I-NMe$_3$

File: jag_I-NMe3_cat_opt_wB97X-D_LACVPs/jag_I-NMe3_cat_opt_wB97X-D_LACVPs.out; structure: final

Method: DFT(wb97x-d)/lacvp*; Solvent: water

Total Quantum Mechanical Energy: -185.727034921 au

Solution phase energy: -185.63252451833 au

Gibbs free energy: -185.536758 au

Low frequencies 264.04 277.11 279.00 279.34 343.95 344.13

I1	0.0000000000	-0.0000000000	-0.8088211765
N2	0.0000000000	-0.0000000000	1.3504658552
C3	1.2328461702	-0.7117840682	1.8334430317
H4	1.2114464826	-0.6994289528	2.9286218295
H5	1.2210115718	-1.7410717142	1.4674808863
H6	2.1183181202	-0.1868911824	1.4674808863
C7	-0.0000000000	1.4235681365	1.8334430317
H8	0.0000000000	1.3988579056	2.9286218295
H9	0.8973065484	1.9279628966	1.4674808863
H10	-0.8973065484	1.9279628966	1.4674808863
C11	-1.2328461702	-0.7117840682	1.8334430317
H12	-1.2114464826	-0.6994289528	2.9286218295
H13	-2.1183181202	-0.1868911824	1.4674808863
H14	-1.2210115718	-1.7410717142	1.4674808863

Start: BF_2 intermediate

File: jag_4a_opt_wB97X-D_LACVPs/jag_4a_opt_wB97X-D_LACVPs.out; structure: final

Method: DFT(wb97x-d)/lacvp*; Solvent: water

Total Quantum Mechanical Energy: -782.11089357 au

Solution phase energy: -782.07289788573 au

Gibbs free energy: -781.865946 au

Low frequencies 69.15 90.82 116.59 143.44 172.03 225.67

C1	1.1510942645	0.8603169971	0.0000000000
C2	-0.0543119976	1.5652931794	0.0000000000
C3	0.0334781344	2.9627583604	0.0000000000
C4	1.2604108676	3.6194696071	0.0000000000
C5	2.4446883042	2.8791711697	0.0000000000
C6	2.3967027360	1.4905753926	0.0000000000
B7	-1.4509192202	0.7847490810	0.0000000000
H8	-0.8835310586	3.5484542037	0.0000000000
H9	1.2984764132	4.7053985341	0.0000000000
H10	3.4063404288	3.3841854346	0.0000000000
H11	3.3116278109	0.9018917037	0.0000000000

F12	-2.2251590689	0.9947894716	-1.1431864940
F13	-2.2251590689	0.9947894716	1.1431864940
H14	2.0124705299	-1.0440504475	0.0000000000
N15	1.1177359269	-0.5589304159	0.0000000000
C16	0.0055569309	-1.2810882744	0.0000000000
O17	-1.1437922544	-0.7415078708	0.0000000000
C18	0.0976657978	-2.7997656947	0.0000000000
C19	-1.3151583435	-3.3991818468	0.0000000000
H20	-1.2360559114	-4.4915416921	0.0000000000
H21	-1.8791568931	-3.0935804786	0.8867922026
H22	-1.8791568931	-3.0935804786	-0.8867922026
C23	0.8527552738	-3.2478332305	-1.2682858923
H24	0.8832274585	-4.3423982269	-1.2963401420
H25	0.3436701627	-2.8940971808	-2.1720170790
H26	1.8860081306	-2.8830322758	-1.2872023366
C27	0.8527552738	-3.2478332305	1.2682858923
H28	0.8832274585	-4.3423982269	1.2963401420
H29	1.8860081306	-2.8830322758	1.2872023366
H30	0.3436701627	-2.8940971808	2.1720170790

pi complex

File: jag_4a_I_NMe3_ipso_pi_opt_wB97X-D_LACVPs_2/jag_4a_I_NMe3_ipso_pi_opt_wB97X-D_LACVPs_2.out; structure: final

Method: DFT(wb97x-d)/lacvp*; Solvent: water

Total Quantum Mechanical Energy: -967.834458377 au

Solution phase energy: -967.71934920983 au

Gibbs free energy: -967.394383 au

Low frequencies 55.02 59.44 63.85 67.47 73.41 86.98

I1	2.6295550000	-2.0969280000	-0.4513360000
C2	-1.3827340000	-3.5599760000	-2.3426780000
C3	-1.5755960000	-2.7475900000	-1.2244350000
C4	-0.7733080000	-3.0121310000	-0.1088030000
C5	0.1707820000	-4.0427290000	-0.1119290000
C6	0.3378490000	-4.8309340000	-1.2605840000
C7	-0.4379430000	-4.5898970000	-2.3845050000

B8	-2.7028770000	-1.6087640000	-1.2360800000
H9	-0.9033940000	-2.4155300000	0.7918780000
H10	0.7188260000	-4.2842080000	0.7967080000
H11	1.0637110000	-5.6391040000	-1.2664070000
H12	-0.3230900000	-5.1963700000	-3.2805860000
F13	-2.2182750000	-0.3242300000	-0.9949050000
F14	-3.7818690000	-1.8795930000	-0.3909970000
H15	-2.0293170000	-3.9582380000	-4.2875700000
N16	-2.1763230000	-3.3376060000	-3.4959380000
C17	-3.0701760000	-2.3676250000	-3.6141330000
O18	-3.3094000000	-1.5554530000	-2.6637330000
C19	-3.8452950000	-2.1626170000	-4.9038920000
C20	-5.3467780000	-2.1691070000	-4.5511610000
H21	-5.9272970000	-1.9860420000	-5.4621680000
H22	-5.6515340000	-3.1366570000	-4.1357200000
H23	-5.5854430000	-1.3864520000	-3.8248740000
C24	-3.4407100000	-0.7789190000	-5.4565590000
H25	-4.0074230000	-0.5810230000	-6.3732100000
H26	-3.6605670000	0.0133200000	-4.7344200000
H27	-2.3717250000	-0.7475800000	-5.6974260000
C28	-3.5484210000	-3.2494200000	-5.9449730000
H29	-4.1491150000	-3.0533810000	-6.8395120000
H30	-2.4952540000	-3.2481290000	-6.2536490000
H31	-3.8172250000	-4.2483020000	-5.5787720000
N32	4.4817010000	-0.9434520000	-0.4832660000
C33	5.4843510000	-1.6211600000	0.4030570000
H34	6.4069460000	-1.0312640000	0.3698650000
H35	5.0971680000	-1.6546710000	1.4243650000
H36	5.6708710000	-2.6310240000	0.0296720000
C37	4.9949470000	-0.8868290000	-1.8911010000
H38	5.9246430000	-0.3075200000	-1.8806550000
H39	5.1898450000	-1.9022570000	-2.2443920000
H40	4.2533030000	-0.3950470000	-2.5250380000
C41	4.2040590000	0.4416950000	0.0208190000

H42	5.1486010000	0.9963070000	-0.0016450000
H43	3.4709890000	0.9228790000	-0.6310150000
H44	3.8279020000	0.3833900000	1.0449460000

Backward QRC from Ipso TS

File:jag_4a_I_NMe3_ipso_ts_wB97X-D_LACVPs_2/jag_4a_I_NMe3_ipso_ts_wB97X-D_LACVPs_2_bQRC.out; structure: final

Method: DFT(wb97x-d)/lacvp*; Solvent: water

Total Quantum Mechanical Energy: -967.830463668 au

Solution phase energy: -967.71434010982 au

I1	1.1337528386	-0.8672721831	-0.0321604824
C2	-1.5107885973	-3.9569003161	-2.4413450884
C3	-2.1022532784	-2.9662505363	-1.6602791712
C4	-1.6236289443	-2.8228832463	-0.3504269617
C5	-0.5847076330	-3.6194343494	0.1380686376
C6	0.0117957006	-4.5748811823	-0.6959199548
C7	-0.4542284445	-4.7537181868	-1.9893092024
B8	-3.2726195449	-2.0706086510	-2.2841636476
H9	-2.0995732514	-2.0994064897	0.3090146730
H10	-0.2669559922	-3.5305249891	1.1748594196
H11	0.8209522949	-5.1961106813	-0.3227556785
H12	-0.0246084075	-5.5179023182	-2.6338456877
F13	-2.8329996194	-0.8458383380	-2.7933664037
F14	-4.3610437305	-1.8807629906	-1.4377171021
H15	-1.5709010434	-4.9131662486	-4.3003800261
N16	-2.0510447340	-4.2245450606	-3.7267977308
C17	-3.2047742655	-3.7370019351	-4.1608897973
O18	-3.8369398850	-2.8414200889	-3.5117519190
C19	-3.8486044049	-4.2448175113	-5.4404141594
C20	-5.2415671483	-4.7916647681	-5.0582249848
H21	-5.7490178434	-5.1380263034	-5.9656175514
H22	-5.1567547145	-5.6380764683	-4.3664781320
H23	-5.8564403406	-4.0174936070	-4.5895360458
C24	-3.9987375438	-3.0428980969	-6.3947964911
H25	-4.5059563283	-3.3718491545	-7.3085659773

H26	-4.5918839181	-2.2464962848	-5.9349790820
H27	-3.0190914710	-2.6360023442	-6.6709506793
C28	-3.0251917984	-5.3525667339	-6.1110703124
H29	-3.5453378814	-5.6776335764	-7.0184172513
H30	-2.0302038083	-5.0008705106	-6.4123084723
H31	-2.9170388872	-6.2308434566	-5.4622230902
N32	2.5273154820	0.7831691375	0.2599304963
C33	2.1961173128	1.8847979746	-0.7055022948
H34	2.9112266542	2.6976351914	-0.5361986167
H35	2.2903335930	1.5100876306	-1.7277861633
H36	1.1783367059	2.2365336424	-0.5183562096
C37	2.3986748920	1.2812366127	1.6713049247
H38	3.1171314916	2.0985805312	1.7994917919
H39	1.3822580071	1.6492216849	1.8336093516
H40	2.6285683329	0.4685452824	2.3645161472
C41	3.9218042542	0.2811279969	0.0148440097
H42	4.6094384884	1.1204350061	0.1679134178
H43	4.1466260954	-0.5205181715	0.7230213901
H44	3.9980687928	-0.0828986504	-1.0126087879

Ipso TS

File:jag_4a_I_NMe3_ipso_ts_wB97X-D_LACVPs_2/jag_4a_I_NMe3_ipso_ts_wB97X-D_LACVPs_2.out; structure: final

Method: DFT(wb97x-d)/lacvp*; Solvent: water

Total Quantum Mechanical Energy: -967.777194174 au

Solution phase energy: -967.69170367785 au

Gibbs free energy: -967.369279 au

Low frequencies -58.13 86.70 92.74 95.58 99.32 106.19

I1	0.3234206804	-1.4251059816	-1.1419906205
C2	-1.0116165369	-4.2309949889	-1.7275416952
C3	-0.7672394967	-3.2999129934	-0.6818253330
C4	-0.5587704960	-3.8330698077	0.6167264431
C5	-0.6485562157	-5.1947146973	0.8556758370
C6	-0.8932403970	-6.0706251357	-0.2055007625
C7	-1.0912193464	-5.5930346656	-1.5038165373

B8	-2.6148875424	-2.7061760770	-0.6270697775
H9	-0.3528676082	-3.1508680756	1.4381335145
H10	-0.5193936702	-5.5797018679	1.8626941005
H11	-0.9619033550	-7.1386262333	-0.0179720699
H12	-1.3415696934	-6.2688395719	-2.3171739179
F13	-2.6634672011	-1.6568522838	0.2129219411
F14	-3.3850120681	-3.7655547880	-0.2974398419
H15	-0.9888552650	-4.1311252002	-3.8425107480
N16	-1.3833460038	-3.7089978777	-3.0008935673
C17	-2.3048362924	-2.7654732205	-3.0898722072
O18	-2.8280326137	-2.2716201447	-2.0256683471
C19	-2.7612212462	-2.1767844768	-4.4021744954
C20	-4.2992920580	-2.0701036124	-4.3688921381
H21	-4.6378845306	-1.6328113140	-5.3139877077
H22	-4.7635624683	-3.0563819257	-4.2576416212
H23	-4.6375579818	-1.4283657094	-3.5502928776
C24	-2.1387485601	-0.7618465740	-4.4853847247
H25	-2.4899658012	-0.2841722768	-5.4064966307
H26	-2.4420339163	-0.1428154799	-3.6349567015
H27	-1.0446616652	-0.8110260946	-4.5174935849
C28	-2.3084310300	-3.0277383896	-5.5938635759
H29	-2.6966823327	-2.5751580062	-6.5118465765
H30	-1.2156188008	-3.0627047223	-5.6862433364
H31	-2.7009854527	-4.0495688380	-5.5295894541
N32	1.6969723212	0.7930918066	-1.7083875137
C33	2.7944378689	0.4365601202	-2.6097872028
H34	3.3904168148	1.3218847553	-2.8867028737
H35	3.4493456877	-0.2905345011	-2.1202818643
H36	2.3892786559	-0.0132336446	-3.5217434492
C37	0.7828848519	1.7386485277	-2.3529138238
H38	1.2945187094	2.6836261867	-2.5998843573
H39	0.3923564728	1.3023907836	-3.2781342463
H40	-0.0560699359	1.9568001410	-1.6835772729
C41	2.2124529662	1.3460865077	-0.4533771933

H42	2.7924250636	2.2670012207	-0.6305026030
H43	1.3772472954	1.5794032940	0.2155298714
H44	2.8603184233	0.6106904163	0.0347616669

Forward QRC from ipso TS

File:jag_4a_I_NMe3_ipso_ts_wB97X-D_LACVPs_2/jag_4a_I_NMe3_ipso_ts_wB97X-D_LACVPs_2_fQRC.out; structure: final

Method: DFT(wb97x-d)/lacvp*; Solvent: water

Total Quantum Mechanical Energy: -967.789836689 au

Solution phase energy: -967.70339201333 au

I1	0.1262890199	-1.3223825077	-0.6954446975
C2	-1.2909010480	-4.0837020743	-1.0583320001
C3	-0.9558647591	-3.0736524093	-0.1484100627
C4	-1.3963648383	-3.2047590462	1.1699828288
C5	-2.1391318258	-4.3153488098	1.5633802365
C6	-2.4553198048	-5.3171003957	0.6476142664
C7	-2.0279993469	-5.2022573138	-0.6698779298
B8	-3.5437408371	-2.5640454957	-2.2643841348
H9	-1.1625760015	-2.4313427250	1.8965082893
H10	-2.4754990867	-4.3912703984	2.5938487442
H11	-3.0406355044	-6.1789534648	0.9526675711
H12	-2.2729997010	-5.9621584171	-1.4074381914
F13	-4.0704845629	-1.5648549237	-1.6055651793
F14	-4.1577788108	-3.7176596992	-2.3990201114
H15	-0.1646869670	-4.6741406319	-2.7570326385
N16	-0.8865081860	-4.0164863701	-2.4452323139
C17	-1.4260065139	-3.2055312350	-3.3077031243
O18	-2.3288777350	-2.3466110586	-2.8811948947
C19	-1.0518089276	-3.0976098869	-4.7572462390
C20	-2.3640067986	-3.2133821130	-5.5694498050
H21	-2.1126658512	-3.1669969566	-6.6341644061
H22	-2.8732153896	-4.1648170658	-5.3763217641
H23	-3.0494792391	-2.3880406855	-5.3489827033
C24	-0.4287888600	-1.6945647544	-4.9619829257
H25	-0.2028531209	-1.5750943204	-6.0268979724

H26	-1.1204473581	-0.9004493229	-4.6634753477
H27	0.5031869238	-1.5890067662	-4.3960695695
C28	-0.0621851717	-4.1935619347	-5.1634293779
H29	0.1645183201	-4.0817834729	-6.2285621533
H30	0.8843188809	-4.1128549833	-4.6146018085
H31	-0.4866944665	-5.1936829921	-5.0128724317
N32	1.3744037986	1.2556850955	-1.4035919549
C33	2.6349033230	1.3852590305	-0.6815776540
H34	3.1198984824	2.3625839354	-0.8624541282
H35	2.4566042307	1.2815188061	0.3941469386
H36	3.3234280126	0.5939568478	-0.9971499393
C37	1.5844912572	1.3327357768	-2.8444202995
H38	2.0254812659	2.2997969302	-3.1499435079
H39	2.2572995371	0.5299856809	-3.1654160622
H40	0.6271620072	1.2133509903	-3.3639457695
C41	0.4262497906	2.2741996875	-0.9639572184
H42	0.7927146905	3.2978207309	-1.1658691816
H43	-0.5291032241	2.1392387166	-1.4827418147
H44	0.2516748420	2.1775366078	0.1132530824

Addition product with carrier model (NMe_3) still attached

File:jag_4a_I_NMe3_ipsoprod_opt_wB97X-D_LACVPs_2/jag_4a_I_NMe3_ipsoprod_opt_wB97X-D_LACVPs_2.out; structure: final

Method: DFT(wb97x-d)/lacvp*; Solvent: water

Total Quantum Mechanical Energy: -967.787831068 au

Solution phase energy: -967.70516735607 au

Gibbs free energy: -967.383991 au

Low frequencies 38.42 66.63 87.37 88.32 94.88 98.08

I1	-2.1326026271	-1.3469734164	-0.4783084919
C2	-1.0362011672	-4.0071797712	-1.6976043873
C3	-1.1917296645	-3.2574278316	-0.5268191165
C4	-0.7177906888	-3.8011119708	0.6687653562
C5	-0.1024299668	-5.0508277459	0.6868852385
C6	0.0534231977	-5.7794429954	-0.4900052199
C7	-0.4169915101	-5.2573622864	-1.6889251232

B8	-3.8335729330	-5.0862481858	-1.6765719617
H9	-0.8316891310	-3.2470317042	1.5965565091
H10	0.2546410251	-5.4544281135	1.6304231718
H11	0.5294026980	-6.7551987008	-0.4767060423
H12	-0.3240937974	-5.8164889038	-2.6164274497
F13	-4.5237667208	-5.0947913795	-0.5652339245
F14	-3.3467701836	-6.1846593297	-2.2113756798
H15	-0.8641403862	-3.2380350374	-3.6727404547
N16	-1.5454038849	-3.5417724208	-2.9699084750
C17	-2.8134335599	-3.5357002395	-3.2602240942
O18	-3.6686919956	-3.8744715183	-2.3154503785
C19	-3.4110640778	-3.1105364564	-4.5697248940
C20	-4.3309140169	-4.2647542692	-5.0370198442
H21	-4.7534971748	-3.9923902895	-6.0099134004
H22	-3.7750247600	-5.2025854426	-5.1547266740
H23	-5.1637791761	-4.4247870391	-4.3434705478
C24	-4.2624695438	-1.8473608313	-4.2946917130
H25	-4.7593524934	-1.5628831089	-5.2283621502
H26	-5.0306144504	-2.0374794025	-3.5385952812
H27	-3.6359214962	-1.0115100893	-3.9666436618
C28	-2.3285185036	-2.8197777224	-5.6136676487
H29	-2.8157768720	-2.5327715415	-6.5510416384
H30	-1.6819578167	-1.9872319623	-5.3099646109
H31	-1.7119791409	-3.7049044370	-5.8133057797
N32	-3.5135068788	1.1949901103	-0.2962275679
C33	-4.8296935573	0.9322729541	0.2771916704
H34	-5.4272737103	1.8555999623	0.3879571244
H35	-5.3807545226	0.2373358973	-0.3662887395
H36	-4.7171821208	0.4722770742	1.2647605844
C37	-2.7315386839	2.0707732168	0.5710401303
H38	-3.2041987808	3.0625005811	0.6979773093
H39	-2.6230655147	1.6098547002	1.5595264825
H40	-1.7329177324	2.2156320262	0.1441004925
C41	-3.6316566928	1.7585629347	-1.6362094574

H42	-4.1732273798	2.7221690272	-1.6391089665
H43	-2.6340157943	1.9210022146	-2.0589012805
H44	-4.1703066479	1.0581842140	-2.2840375783

Carrier model, NMe_3

File: jag_Me3N_opt_wB97X-D_LACVPs/jag_Me3N_opt_wB97X-D_LACVPs.out; structure: final

Method: DFT(wb97x-d)/lacvp*; Solvent: water

Total Quantum Mechanical Energy: -174.427376604 au

Solution phase energy: -174.42152553246 au

Gibbs free energy: -174.325895 au

Low frequencies 285.38 311.84 311.95 405.31 459.36 459.53

N32	3.9888759706	-1.3795094237	0.0357222146
C33	4.3577015380	-1.2522507690	-1.3679373686
H34	5.3418008468	-0.7619685192	-1.5070838302
H35	4.4045237851	-2.2428961783	-1.8346071504
H36	3.6059351739	-0.6587693742	-1.9007975269
C37	3.8828929321	-0.0664505707	0.6580120112
H38	4.8401418073	0.4917420098	0.6410378724
H39	3.1309836062	0.5363444632	0.1358764047
H40	3.5707049282	-0.1708533198	1.7034812828
C41	4.9555621142	-2.2062036265	0.7458898489
H42	5.9766568838	-1.7755171301	0.7319438815
H43	4.6521885201	-2.3224373346	1.7927099296
H44	5.0024109103	-3.2023756492	0.2911975427

Addition product with carrier model (NMe_3) dissociated

File: jag_4a_I_ipsoprod_opt_wB97X-D_LACVPs/jag_4a_I_ipsoprod_opt_wB97X-D_LACVPs.out; structure: final

Method: DFT(wb97x-d)/lacvp*; Solvent: water

Total Quantum Mechanical Energy: -793.360381789 au

Solution phase energy: -793.27462612311 au

Gibbs free energy: -793.071175 au

Low frequencies 44.33 74.64 91.10 92.25 105.80 120.35

I1	-1.6844790432	-1.1789344053	-0.7724360300
C2	-1.1415614346	-4.0827096026	-1.6682916934
C3	-1.0881571022	-3.1978044197	-0.5864466263

C4	-0.6276826119	-3.6543904512	0.6453445102
C5	-0.2159490406	-4.9783718127	0.7851800656
C6	-0.2504248615	-5.8487855893	-0.3005628302
C7	-0.7168175614	-5.4021411630	-1.5339799566
B8	-4.0401408016	-5.0520806464	-1.5303949458
H9	-0.5918244537	-2.9838657625	1.4980222878
H10	0.1334016018	-5.3237789751	1.7543337210
H11	0.0725711193	-6.8789931101	-0.1883357347
H12	-0.7692503103	-6.0696371092	-2.3904028201
F13	-4.6894223873	-5.0415635463	-0.3962225096
F14	-3.6793115151	-6.1666434752	-2.1263961919
H15	-1.0294392343	-3.4490273368	-3.6990348711
N16	-1.6847228857	-3.6701048586	-2.9415959252
C17	-2.9681066650	-3.5787526594	-3.1408204310
O18	-3.7645624369	-3.8340761986	-2.1168914733
C19	-3.6354737891	-3.1401328631	-4.4096957530
C20	-4.5347932551	-4.3174140476	-4.8641690390
H21	-5.0087085288	-4.0356278174	-5.8100415793
H22	-3.9502990170	-5.2296492830	-5.0283701077
H23	-5.3320459857	-4.5238298714	-4.1411734332
C24	-4.5191640508	-1.9126211021	-4.0716538377
H25	-5.0583393727	-1.6260504340	-4.9814135347
H26	-5.2541274200	-2.1471063143	-3.2948468889
H27	-3.9149193081	-1.0586363925	-3.7453233508
C28	-2.6088474150	-2.7890128445	-5.4898016655
H29	-3.1451120141	-2.4732373125	-6.3902306655
H30	-1.9652538462	-1.9572617230	-5.1780105978
H31	-1.9877359242	-3.6527289508	-5.7564064373

Addition product with water added to B

File:jag_4a_I_ipsoprod_H2O_opt_wB97X-D_LACVPs/jag_4a_I_ipsoprod_H2O_opt_wB97X-D_LACVPs.out; structure: final

Method: DFT(wb97x-d)/lacvp*; Solvent: water

Total Quantum Mechanical Energy: -869.818555404 au

Solution phase energy: -869.72765128785 au

Gibbs free energy: -869.496955 au

Low frequencies 67.24 81.97 88.47 110.23 122.72 130.74

I1	-2.0948944161	-1.0281433612	-0.5943410190
C2	-1.1065512001	-3.0078466345	-2.7336419276
C3	-0.7324164995	-2.3100842400	-1.5842841924
C4	0.5642204105	-2.4306633956	-1.0935458361
C5	1.4830704462	-3.2366243848	-1.7623847597
C6	1.1126279449	-3.9241098785	-2.9153409638
C7	-0.1857332537	-3.8106054279	-3.4013108230
B8	-4.3064246561	-5.0254703056	-1.0326801989
H9	0.8643442860	-1.8905909457	-0.2016185498
H10	2.4959247086	-3.3160843689	-1.3778119404
H11	1.8330581939	-4.5435766735	-3.4407225417
H12	-0.4957390787	-4.3295253021	-4.3049944520
F13	-4.8668648974	-4.1655450421	-0.1433286652
F14	-5.1563222468	-5.8547679251	-1.7006618603
H15	-2.4516107022	-2.1921505547	-4.0952632625
N16	-2.4048763690	-2.8412385572	-3.3119473956
C17	-3.5080284613	-3.4527145731	-2.9226319845
O18	-3.3754520379	-4.2966315370	-1.9568474914
C19	-4.7982457175	-3.1432281675	-3.6872434080
C20	-5.1064093612	-4.3694673825	-4.5766314101
H21	-6.0218417463	-4.1613601995	-5.1418378131
H22	-4.2960100069	-4.5459423746	-5.2935264939
H23	-5.2611653193	-5.2720182096	-3.9824944545
C24	-5.9637043036	-2.8587912067	-2.7191131457
H25	-6.8182439740	-2.5195583721	-3.3148469561
H26	-6.2772332698	-3.7475528044	-2.1713901909
H27	-5.7126916113	-2.0660431100	-2.0058097760
C28	-4.6253940791	-1.9095980935	-4.5983156805
H29	-5.5842227243	-1.7100185269	-5.0871234794
H30	-4.3511987353	-1.0130286949	-4.0296938593
H31	-3.8957506940	-2.0691270467	-5.4027327118
H32	-3.6414549385	-6.9428430616	-0.1841772539

O33	-3.3657121583	-5.9974165788	-0.1811430227
H34	-3.2436130111	-5.7532970265	0.7644470569

Product w. boron moiety dissociated (neutral)

File: jag_3a_opt_wB97X-D_LACVPs/jag_3a_opt_wB97X-D_LACVPs.out; structure: final

Method: DFT(wb97x-d)/lacvp*; Solvent: water

Total Quantum Mechanical Energy: -568.845166744 au

Solution phase energy: -568.81903440318 au

Gibbs free energy: -568.624770 au

Low frequencies 80.70 103.05 107.05 123.90 168.56 197.37

C1	-0.6738494270	0.9377346404	-0.0331366688
C2	-0.0104934712	1.2022067666	1.1690452442
C3	1.3794167733	1.2495134527	1.2236127536
C4	2.1185637864	1.0316867860	0.0630549217
C5	1.4712225224	0.7678777521	-1.1415082087
C6	0.0812052145	0.7202645022	-1.1853954499
H8	1.8869646957	1.4557161966	2.1601884205
H9	3.2030328002	1.0712308882	0.1086778886
H10	2.0472460660	0.6005553976	-2.0464017707
H11	-0.4423218775	0.5171722286	-2.1156722356
H14	-2.5673784405	1.7810037803	-0.2970585938
N15	-2.0934794626	0.9066023230	-0.1047682903
C16	-2.7999313139	-0.2369677547	0.1122887164
O17	-2.2351486952	-1.3093140346	0.3306628013
C18	-4.3342376473	-0.1352118431	0.0798552144
C19	-4.8240430254	-1.0434068097	-1.0627179767
H20	-5.9199255348	-1.0548495535	-1.0836897534
H21	-4.4676635818	-0.6812131149	-2.0342969222
H22	-4.4678966088	-2.0687315339	-0.9237222937
C23	-4.8517129726	-0.6709744443	1.4278540874
H24	-5.9481271414	-0.6860264839	1.4241585546
H25	-4.4892384048	-1.6878200950	1.6051698335
H26	-4.5220439304	-0.0367246660	2.2596279010
C27	-4.8575190788	1.2914108784	-0.1360160144
H28	-5.9531779029	1.2782648357	-0.1306317760

H29	-4.5345115610	1.9692658358	0.6635784691
H30	-4.5436037230	1.7055289162	-1.1022875918
I32	-1.1188377231	1.5460732340	2.9453630594

Dissociated boron w. water, cationic

File: jag_F2B_OH2_opt_wB97X-D_LACVPs/jag_F2B_OH2_opt_wB97X-D_LACVPs.out; structure: final

Method: DFT(wb97x-d)/lacvp*; Solvent: water

Total Quantum Mechanical Energy: -300.991158351 au

Solution phase energy: -300.85320340927 au

Gibbs free energy: -300.842564 au

Low frequencies 405.80 477.19 592.79 616.64 633.54 846.27

B8	-3.4599393577	-5.0278486817	-1.0128985599
F13	-4.4555423324	-4.5274448994	-0.3445259206
F14	-3.5524927421	-6.0214850022	-1.8449576604
H32	-1.3625538326	-4.7422343892	-1.3072857005
O33	-2.1708391545	-4.4510106289	-0.8109982222
H34	-2.0133779977	-3.6700346224	-0.2195319893

Dissociated boron with hydroxide, neutral

File: jag_F2B_OH_opt_wB97X-D_LACVPs/jag_F2B_OH_opt_wB97X-D_LACVPs.out; structure: final

Method: DFT(wb97x-d)/lacvp*; Solvent: water

Total Quantum Mechanical Energy: -300.465627492 au

Solution phase energy: -300.44831736065 au

Gibbs free energy: -300.449151 au

Low frequencies 474.64 478.47 676.94 747.68 883.56 1147.92

B8	-3.3897200218	-5.0058478822	-1.0088016737
F13	-4.4610987784	-4.5585644967	-0.3585197381
F14	-3.5609059346	-6.0259509904	-1.8481742109
H32	-1.4696994409	-4.8025970035	-1.3133467676
O33	-2.1958266973	-4.4200833105	-0.7929947138

Protonated carrier model (NMe_3), cationic

File: jag_Me3NH_cat_opt_wB97X-D_LACVPs/jag_Me3NH_cat_opt_wB97X-D_LACVPs.out; structure: final

Method: DFT(wb97x-d)/lacvp*; Solvent: water

Total Quantum Mechanical Energy: -175.004035492 au

Solution phase energy: -174.89609487154 au

Gibbs free energy: -174.785547 au

Low frequencies 202.57 283.61 286.68 428.47 429.27 483.18

N32	3.9889827585	-1.3794822470	0.0355733107
C33	4.3579829168	-1.2537327422	-1.4082007753
H34	5.3346819361	-0.7640239619	-1.4703383756
H35	4.4063941102	-2.2528129698	-1.8456481419
H36	3.5994922105	-0.6516268878	-1.9122807580
C37	3.8696699454	-0.0336551367	0.6765032719
H38	4.8458727012	0.4580020504	0.6225940779
H39	3.1219327245	0.5479621752	0.1337683265
H40	3.5692428997	-0.1667215326	1.7176318028
C41	4.9728760995	-2.2353033643	0.7675477095
H42	5.9528529750	-1.7511203329	0.7148448139
H43	4.6516024498	-2.3278244310	1.8068789480
H44	5.0080857913	-3.2160632024	0.2892283067
H14	3.0679792531	-1.8390852496	0.0882852431

Dissociated boron w. carrier model (NMe_3), cationic

File:jag_Me3N_F2B_OH2_opt_wB97X-D_LACVPs/jag_Me3N_F2B_OH2_opt_wB97X-D_LACVPs.out; structure: final

Method: DFT(wb97x-d)/lacvp*; Solvent: water

Total Quantum Mechanical Energy: -475.457214494 au

Solution phase energy: -475.35384964252 au

Gibbs free energy: -475.221816 au

Low frequencies 104.86 222.09 228.93 233.59 295.07 305.67

B8	2.6341640000	-2.2560580000	0.2739540000
F13	1.5473120000	-1.5047660000	-0.0568910000
F14	2.8043990000	-3.4150760000	-0.4209770000
H32	2.4789430000	-3.6433960000	1.9813100000
O33	2.4331960000	-2.6765740000	1.7974590000
H34	1.6025610000	-2.3545320000	2.2184360000
N32	3.9617120000	-1.3566030000	0.1343980000
C33	4.1232730000	-0.9887810000	-1.3174090000

H9	5.0206160000	-0.3704290000	-1.4228530000
H35	4.2316140000	-1.8984670000	-1.9112060000
H36	3.2479900000	-0.4253910000	-1.6468810000
C37	3.8556620000	-0.0898150000	0.9244830000
H38	4.7652270000	0.4983530000	0.7677090000
H39	2.9896970000	0.4767780000	0.5770560000
H40	3.7526860000	-0.3314600000	1.9843770000
C41	5.1838460000	-2.1071570000	0.5619430000
H42	6.0595960000	-1.4696700000	0.4055460000
H43	5.0998860000	-2.3618280000	1.6204560000
H44	5.2777030000	-3.0116930000	-0.0420240000

Addition product with carrier model (NMe_3) added to B

File: jag_4a_I_ipsoprod_NMe3_opt_wB97X-D_LACVPs/jag_4a_I_ipsoprod_NMe3_opt_wB97X-D_LACVPs.out; structure: final

Method: DFT(wb97x-d)/lacvp*; Solvent: water

Total Quantum Mechanical Energy: -967.854252245 au

Solution phase energy: -967.77268601493 au

Gibbs free energy: -967.444869 au

Low frequencies 73.65 79.67 90.52 110.65 114.52 118.25

I1	-2.1231501766	-0.9514693178	-0.6665119408
C2	-1.1411490180	-3.0575244948	-2.6844633283
C3	-0.7977309577	-2.3586243310	-1.5251669782
C4	0.4272891707	-2.5980612477	-0.9084368271
C5	1.3036086349	-3.5340081651	-1.4541859502
C6	0.9630921650	-4.2288468415	-2.6118805928
C7	-0.2618893318	-3.9902367349	-3.2279218661
H9	0.6999296391	-2.0628035020	-0.0046459726
H10	2.2567443174	-3.7150871422	-0.9650635925
H11	1.6476231004	-4.9571004696	-3.0360525443
H12	-0.5498452352	-4.5229158927	-4.1309591337
H15	-2.4074018236	-2.1965754885	-4.1265540441
N16	-2.3979963320	-2.8379261662	-3.3353813177
C17	-3.5113838100	-3.4398375174	-2.9520055764
O18	-3.3759703305	-4.2620937105	-1.9787002858

C19	-4.8153259096	-3.1654024119	-3.6878456422
C20	-5.1658262763	-4.4384136024	-4.4912303962
H21	-6.1059867791	-4.2609033379	-5.0249675693
H22	-4.3885270799	-4.6621719593	-5.2308498304
H23	-5.2957816551	-5.3016217214	-3.8365517023
C24	-5.9343898945	-2.8331741490	-2.6781105048
H25	-6.8166950443	-2.5164743614	-3.2449025658
H26	-6.2194205999	-3.6967183267	-2.0768280426
H27	-5.6467891380	-2.0137042411	-2.0099831429
C28	-4.6644829424	-1.9829333965	-4.6602212067
H29	-5.6305180463	-1.8128129411	-5.1459180120
H30	-4.3875526492	-1.0593512222	-4.1381112411
H31	-3.9388819316	-2.1836098529	-5.4584751541
B8	-4.2346991938	-5.0203249701	-1.0005152220
F13	-5.1531644834	-5.8233016601	-1.6371784817
F14	-4.7934904860	-4.1544771244	-0.0959969551
N32	-3.1918282600	-5.9773741048	-0.2190133938
C33	-3.9635802430	-6.8047502714	0.7636063433
H34	-3.2668068206	-7.4469988604	1.3103209787
H35	-4.4807682904	-6.1408268805	1.4594226417
H36	-4.6879068946	-7.4169451794	0.2237888222
C37	-2.4895189737	-6.8883843668	-1.1716486566
H38	-1.8209139222	-7.5475968736	-0.6102265614
H39	-3.2301668043	-7.4817167728	-1.7110660532
H40	-1.9099295608	-6.2881944385	-1.8747695619
C41	-2.1812962987	-5.1720731782	0.5287658789
H42	-1.5190266781	-5.8475550115	1.0784417142
H43	-1.6003976881	-4.5836623340	-0.1818171137
H44	-2.6981535248	-4.5098218507	1.2251025920

Dissociated boron with carrier model (NMe_3), neutral

File: jag_Me3NBF2_opt_wB97X-D_LACVPs/jag_Me3NBF2_opt_wB97X-D_LACVPs.out; structure: final

Method: DFT(wb97x-d)/lacvp*; Solvent: water

Total Quantum Mechanical Energy: -398.98856669 au

Solution phase energy: -398.88751277646 au

Gibbs free energy: -398.782395 au

Low frequencies 58.13 205.51 229.58 257.03 295.88 319.14

B8	-4.4192717518	-5.2278456043	-0.7961474177
F13	-5.1749337233	-5.7910267955	-1.6996655789
F14	-4.4839251432	-3.9531401044	-0.5271976986
N32	-3.4548778781	-6.1099353342	-0.0162555918
C33	-4.2983215020	-6.9077411802	0.9601161594
H9	-3.6242781367	-7.5479793923	1.5356407306
H35	-4.8191449628	-6.2132263784	1.6265397120
H36	-5.0124338341	-7.5162654872	0.3993701754
C37	-2.7442344457	-7.0471732116	-0.9649385187
H38	-2.0689623897	-7.6709387111	-0.3742929925
H39	-3.4826070730	-7.6681263992	-1.4757567629
H40	-2.1765419615	-6.4522364394	-1.6862437471
C41	-2.4445044163	-5.2874596875	0.7398349715
H42	-1.7894731220	-5.9750877543	1.2799718121
H43	-1.8664364435	-4.6949690797	0.0264207876
H44	-2.9684177400	-4.6357172784	1.4419765759

Alternative starting material before Br_2 has been replaced by F_2

File: jag_4Br2_opt_wB97X-D_LACVPs/jag_4Br2_opt_wB97X-D_LACVPs.out; structure: final

Method: DFT(wb97x-d)/lacvp*; Solvent: water

Total Quantum Mechanical Energy: -608.804480964 au

Solution phase energy: -608.75817058117 au

Gibbs free energy: -608.558364 au

Low frequencies 65.92 92.74 97.92 120.65 124.02 138.82

C1	2.0879535347	0.4752650629	0.0000000000
C2	0.8769252181	1.1704816315	0.0000000000
C3	0.9364712231	2.5687558684	0.0000000000
C4	2.1539705743	3.2366552817	0.0000000000
C5	3.3486628032	2.5108005617	0.0000000000
C6	3.3238641154	1.1223397756	0.0000000000
B7	-0.4542339388	0.3318110197	0.0000000000
H8	0.0086367345	3.1365892045	0.0000000000

H9	2.1778224778	4.3229434310	0.0000000000
H10	4.3021199670	3.0309592433	0.0000000000
H11	4.2465025617	0.5463139775	0.0000000000
Br12	-1.6341638095	0.6978978426	-1.6970691102
Br13	-1.6341638095	0.6978978426	1.6970691102
H14	2.9644019383	-1.4266717078	0.0000000000
N15	2.0648167983	-0.9460491305	0.0000000000
C16	0.9645087760	-1.6692539300	0.0000000000
O17	-0.1962722400	-1.1184714221	0.0000000000
C18	1.0178973903	-3.1847509210	0.0000000000
C19	-0.4093889531	-3.7532539915	0.0000000000
H20	-0.3515033288	-4.8466405042	0.0000000000
H21	-0.9677478571	-3.4372489388	0.8867318677
H22	-0.9677478571	-3.4372489388	-0.8867318677
C23	1.7623613548	-3.6440850705	-1.2708713181
H24	1.7731307939	-4.7389087600	-1.2968211955
H25	1.2548700066	-3.2825149279	-2.1725554738
H26	2.8006506412	-3.2942014944	-1.2920311824
C27	1.7623613548	-3.6440850705	1.2708713181
H28	1.7731307939	-4.7389087600	1.2968211955
H29	2.8006506412	-3.2942014944	1.2920311824
H30	1.2548700066	-3.2825149279	2.1725554738

TS for alternative Br_2 starting material

File: jag_4Br2_I_NMe3_ipso_ts_wB97X-D_LACVPs/jag_4Br2_I_NMe3_ipso_ts_wB97X-D_LACVPs.out; structure: final

Method: DFT(wb97x-d)/lacvp*; Solvent: water

Total Quantum Mechanical Energy: -794.432869124 au

Solution phase energy: -794.34573821335 au

Gibbs free energy: -794.030792 au

Low frequencies -248.25 62.33 81.37 92.09 98.50 101.14

I1	0.4383802561	-1.4836252488	-1.1471461382
C2	-0.9630259501	-4.2543947179	-1.6579460478
C3	-0.6425235444	-3.3196780228	-0.6428116283
C4	-0.4743354783	-3.8233881671	0.6685367410

C5	-0.6338751214	-5.1730087541	0.9406745903
C6	-0.9248885131	-6.0641836527	-0.0957443271
C7	-1.1048832729	-5.6072812242	-1.4022330416
B8	-2.7373277121	-2.6632308028	-0.6716360423
H9	-0.2136947206	-3.1368123102	1.4701819767
H10	-0.5236318999	-5.5368106748	1.9578617548
H11	-1.0463575676	-7.1222596849	0.1184647841
H12	-1.4023099192	-6.2855646250	-2.1973375638
Br13	-2.8216585444	-1.1148418934	0.5489873769
Br14	-3.9629681427	-4.1467732270	-0.2047780353
H15	-0.8990421018	-4.1965354526	-3.7787532660
N16	-1.3154987380	-3.7573646375	-2.9538544353
C17	-2.2269830575	-2.8229538785	-3.0939265375
O18	-2.7644212490	-2.2799237221	-2.0437769909
C19	-2.6817702675	-2.2632938055	-4.4154437458
C20	-4.2246770303	-2.2033562032	-4.3965815729
H21	-4.5657275073	-1.7843982755	-5.3489914718
H22	-4.6617951977	-3.2010941490	-4.2796212214
H23	-4.5900015759	-1.5613855228	-3.5895819118
C24	-2.1028376718	-0.8298711737	-4.5075504206
H25	-2.4615107945	-0.3749867089	-5.4369997221
H26	-2.4351201989	-0.2118473360	-3.6674823580
H27	-1.0077982867	-0.8440762699	-4.5292969546
C28	-2.1891041754	-3.1191306352	-5.5863785670
H29	-2.5690588398	-2.6848198812	-6.5165343611
H30	-1.0942580270	-3.1319406441	-5.6549237429
H31	-2.5626113332	-4.1477890808	-5.5167765284
N32	1.8030495544	0.8129042081	-1.6589108089
C33	2.7422262338	0.5906567383	-2.7587766111
H34	3.3021962347	1.5083557060	-3.0067164275
H35	3.4557674956	-0.1923368502	-2.4795707922
H36	2.1948687906	0.2643501357	-3.6502592035
C37	0.8134147282	1.8315414435	-2.0152737970
H38	1.2896117937	2.8095773732	-2.2004892442

H39	0.2780068135	1.5272957867	-2.9213345756
H40	0.0895292253	1.9448514400	-1.2004771305
C41	2.5085601957	1.1860170638	-0.4314484480
H42	3.0630217129	2.1316587256	-0.5557977977
H43	1.7871060741	1.3094828597	0.3843965392
H44	3.2163325529	0.3957749746	-0.1570150432

Alternative addtion product from Br_2 starting material

File:jag_4Br2_ipso_I_NMe3_prod_opt_wB97XD_LACVPs/jag_4Br2_ipso_I_NMe3_prod_opt_wB97X-D_LACVPs.out; structure: final

Method: DFT(wb97x-d)/lacvp*; Solvent: water

Total Quantum Mechanical Energy: -794.444219815 au

Solution phase energy: -794.35870894336 au

Gibbs free energy: -794.043837 au

Low frequencies 49.56 59.40 77.51 84.37 90.98 98.45

I1	-0.2594983420	-1.0896045726	-0.5993953483
C2	-1.3390527612	-4.0128060675	-0.8469190345
C3	-1.3797944634	-2.8303462462	-0.1015670550
C4	-2.2070871800	-2.7921524304	1.0222397051
C5	-2.9547880424	-3.9069430563	1.3934189313
C6	-2.8813303871	-5.0862726991	0.6548022205
C7	-2.0685124518	-5.1402954664	-0.4713898838
B8	-3.2422042797	-2.7134559633	-2.9926738643
H9	-2.2708342235	-1.8829046999	1.6136638516
H10	-3.5982331653	-3.8484456599	2.2671980835
H11	-3.4645101055	-5.9556854973	0.9422423214
H12	-2.0115443622	-6.0407913316	-1.0777370313
Br13	-4.1005589120	-1.0531827575	-2.5736763388
Br14	-4.2320122736	-4.3371192225	-3.2713238710
N16	-0.5632685094	-4.1185406748	-2.0645876686
C17	-0.8736057943	-3.5105631166	-3.1703123999
O18	-1.8903175910	-2.6676678738	-3.1494761368
C19	-0.1182045387	-3.5882296422	-4.4659422918
C20	-1.1323038442	-3.9686885678	-5.5709225061
H21	-0.5891030657	-4.0391274823	-6.5189834934

H22	-1.6027469247	-4.9377027488	-5.3703914135
H23	-1.9112667135	-3.2073215030	-5.6896230010
C24	0.4435535034	-2.1726455523	-4.7489724856
H25	0.9603295334	-2.2017082181	-5.7140779982
H26	-0.3570530723	-1.4286340661	-4.8094740303
H27	1.1602645972	-1.8635789964	-3.9809728332
C28	1.0111125033	-4.6193733570	-4.3865424119
H29	1.5196969717	-4.6519183859	-5.3551162524
H30	1.7579657157	-4.3484390080	-3.6300214456
H31	0.6245315828	-5.6239503112	-4.1754453715
N32	1.0571727735	1.4286989933	-1.3334573566
C33	0.6086894183	1.7418895387	-2.6853502187
H34	0.9936537308	2.7173735382	-3.0359861650
H35	0.9609139603	0.9669941063	-3.3747351906
H36	-0.4859330145	1.7694125156	-2.7165434255
C37	0.5980517732	2.4390912089	-0.3869254542
H38	1.0075692929	3.4400840255	-0.6169821739
H39	-0.4955236300	2.4993082588	-0.4110716971
H40	0.9076032947	2.1632751527	0.6269041497
C41	2.5077754152	1.2898242517	-1.2868755326
H42	3.0255682845	2.2238402782	-1.5734034561
H43	2.8206037146	1.0178093916	-0.2732062057
H44	2.8243299656	0.4958654811	-1.9721508007
H45	0.2547585869	-4.7374783246	-2.0510925440

9. Single crystal X-ray diffraction

A suitable single crystal of **4a** data was collected on a Rigaku XtaL AB Synergy-DW diffractor equipped with a HyPix-Arc 150° detector using CuKα radition at λ = 1.54184 Å. The data diffraction was obtained and processed with CrysAlisPro software.[35,36] Direct methods was used for all crystal structures and the refinements were established by full-matrix least square with SHELXL,[37] SHELXT[37] and Olex2[38] softwares. Gaussian absorption correction was applied for absorption effects. Anisotropic refinement was applied to all non-hydrogen atoms. All hydrogen except the hydrogen attached to the amine nitrogen were placed with geometric constraints and refined isotropically. The hydrogen attached to the N amine was found in the difference electron density map and was refined isotropically.

Table S2. Crystallographic data and refinement of **4a**.

Code	**4a**
Structural formula	$C_{11}H_{14}B_1F_2N_1O_1$
Molecular mass (g mol^{-1})	225.04
Data collection temp. (K)	101 (3)
Crystal system	Orthorhombic
Space group	$P2_12_12_1$
a (Å)	6.4333(2)
b (Å)	11.2099(3)
c (Å)	15.6487(5)
α (°)	90
β (°)	90
γ (°)	90
Volume (Å^3)	1128.53(6)
Z	4
Dc, calc density (g cm^{-3})	1.325
Absorption coefficient (mm^{-1})	0.891
θ range	4.847- 75.286
Reflections collected	10669
No data I >2 sigma (*I*)	1972
Final *R* indices [I >2 sigma (*I*)]	R_1 = 0.0921<br>wR_2 = 0.2194
R indices (all data)	R_1 = 0.1000

	wR_2 = 0.2245
Goodness-of-fit on F^2	1.073
Largest diff. peak and hole (eÅ^{-3})	0.291; -0.254
CCDC no.	2279239

10. Reference

[1] J. Lv, X. Chen, X. S. Xue, B. Zhao, Y. Liang, M. Wang, L. Jin, Y. Yuan, Y. Han, Y. Zhao, Y. Lu, J. Zhao, W. Y. Sun, K. N. Houk, Z. Shi, *Nature* 2019, **575**, 336–340.

[2] Y. Gao, Y. Mao, B. Zhang, Y. Zhan, Y. Huo, *Org. Biomol. Chem*. 2018, **16**, 3881–3884.

[3] R. S. Dothager, K. S. Putt, B. J. Allen, B. J. Leslie, V. Nesterenko, P. J. Hergenrother, *J. Am. Chem. Soc.* 2005, **127**, 8686–8696.

[4] M. Kumar, S. Verma, A. K. Verma, *Org. Lett.* 2020, **22**, 4620–4626.

[5] T. Xiong, Y. Li, X. Bi, Y. Lv, Q. Zhang, *Angew. Chemie - Int. Ed.* 2011, **50**, 7140–7143.

[6] X. Zhu, M. Xu, J. Sun, D. Guo, Y. Zhang, S. Zhou, S. Wang, *Eur. J. Org. Chem.* 2021, **2021**, 5213–5218.

[7] J. Khan, N. Yadav, A. Tyagi, C. K. Hazra, *J. Org. Chem.* 2022, **87**, 11097–11111.

[8] E. Kianmehr, H. Afaridoun, *Synthesis* 2021, **53**, 1513–1523.

[9] R. Das, M. Kapur, *J. Org. Chem.* 2017, **82**, 1114−1126.

[10] Á. Sinai, Á. Mészáros, T. Gáti, V. Kudar, A. Palló, Z. Novák, *Org. Lett.* 2013, **15**, 5654−5657.

[11] D. J. C. Prasad, G. Sekar, *Org. Biomol. Chem.* 2013, **11**, 1659–1665.

[12] J. I. Urzúa, R. Contreras, C. O. Salas, R. A. Tapia, *RSC Adv.* 2016, **6**, 82401–82408.

[13] X. Abel-Snape, A. Whyte, M. Lautens, *Org. Lett.* 2020, **22**, 7920–7925.

[14] Y. Y. Zhu, H. P. Yi, C. Li, X. K. Jiang, Z. T. Li, *Cryst. Growth Des.* 2008, **8**, 1294–1300.

[15] N. Chaisan, S. Ruengsangtongkul, J. Tummatorn, S. Ruchirawat, K. Chainok, C. Thongsornkleeb, *J. Org. Chem.* 2021, **86**, 4671–4698.

[16] S. Redon, Y. Kabri, M. D. Crozet, P. Vanelle, *Tetrahedron Lett.* 2014, **55**, 5052– 5054.

[17] C. Gimbert, A. Vallribera, *Org. Lett.* 2009, **11**, 269–271.

[18] J. Clayden, H. Turner, M. Pickworth, T. Adler, *Org. Lett.* 2005, **7**, 3147–3150.

[19] S. Sharma, K. C. Basavaraju, A. K. Singh, D. P. Kim, *Org. Lett.* 2014, **16**, 3974–3977.

[20] F. Hasegawa, K. Kawamura, H. Tsuchikawa, M. Murata, *Bioorganic Med. Chem.* 2017, **25**, 4506–4511.

[21)] K. Ni, L. G. Meng, K. Wang, L. Wang, *Org. Lett.* 2018, **20**, 2245–2248.

[22] N. Mishra, A. S. Singh, A. K. Agrahari, S. K. Singh, M. Singh, V. K. Tiwari, *ACS Comb. Sci.* 2019, **21**, 389–399.

[23] W. Li, X. F. Wu, *J. Org. Chem.* 2014, **79**, 10410–10416.

[24] Y. Zheng, M. Dong, E. Qu, J. Bai, X. F. Wu, W. Li, *Chem. Eur. J.* 2021, **27**, 16219–16224.

[25] B. R. Aluri, B. Niaz, M. K. Kindermann, P. G. Jones, J. Heinicke, *Dalt. Trans.* 2011, **40**, 211–224.

[26] G. Evindar, R. A. Batey, *J. Org. Chem.* 2006, **71**, 1802–1808.

[27] T. Saget, N. Cramer, *Angew. Chemie - Int. Ed.* 2013, **52**, 7865–7868.

[28] J. Lu, X. Gong, H. Yang, H. Fu, *Chem. Commun.* 2010, **46**, 4172–4174.

[29] H. K. Saini, N. K. Nandwana, S. Dhiman, K. Rangan, A. Kumar, *Eur. J. Org. Chem.* 2017, **2017**, 7277–7282.

[30] C. Lu, N. A. Markina, R. C. Larock, *J. Org. Chem.* 2012, **77**, 11153–11160.

[31] W. R. Bowman, H. Heaney, P. H. G. Smith, *Tetrahedron Lett.* 1982, **23**, 5093− 5096.

[32] R. Kumar, R. R. Nair, S. S. Dhiman, J. Sharma, O. Prakash, *Med. Chem. Res.* 2010, **19**, 541–550.

[33] Z. Li, H. Sun, H. Jiang, H. Liu, *Org. Lett.* 2008, **10**, 3263–3266.

[34] X. Abel-Snape, A. Whyte, M. Lautens, *Org. Lett.* 2020, **22**, 7920–7925.

[35] Crysalis CCD; Oxford Diffraction Ltd: Abingdon, Oxfordshire, UK, 2005.

[36] Crysalis RED; Oxford Diffraction Ltd: Abingdon, Oxfordshire, UK, 2005.

[37] G. M. Sheldrick, *Acta Crystallogr., Sect. C: Struct Chem.* 2015, **71**, 3-8

[38] O. V. Dolomav, L. J. Bourhis, R. J. Gildea, J. A. K. Howard, H. Puschman, *J. Appl. Crystallogr.* 2009, **42**, 339-341.

11. NMR spectra

11.1. NMR spectra of starting materials:

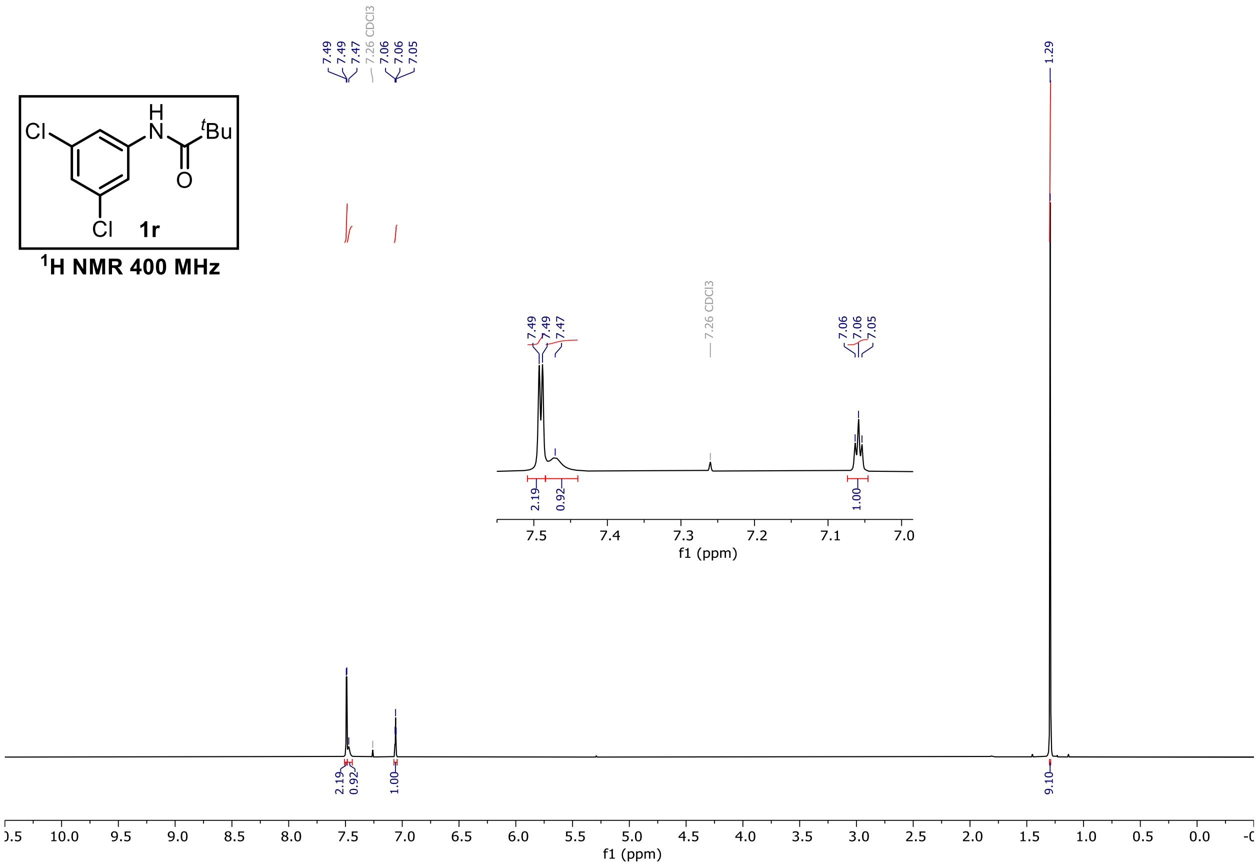
1r
¹H NMR 400 MHz
7.49
7.49
7.47
7.26 CDCl3
7.06
7.06
7.05
1.29
2.19
0.92
1.00
9.10
f1 (ppm)

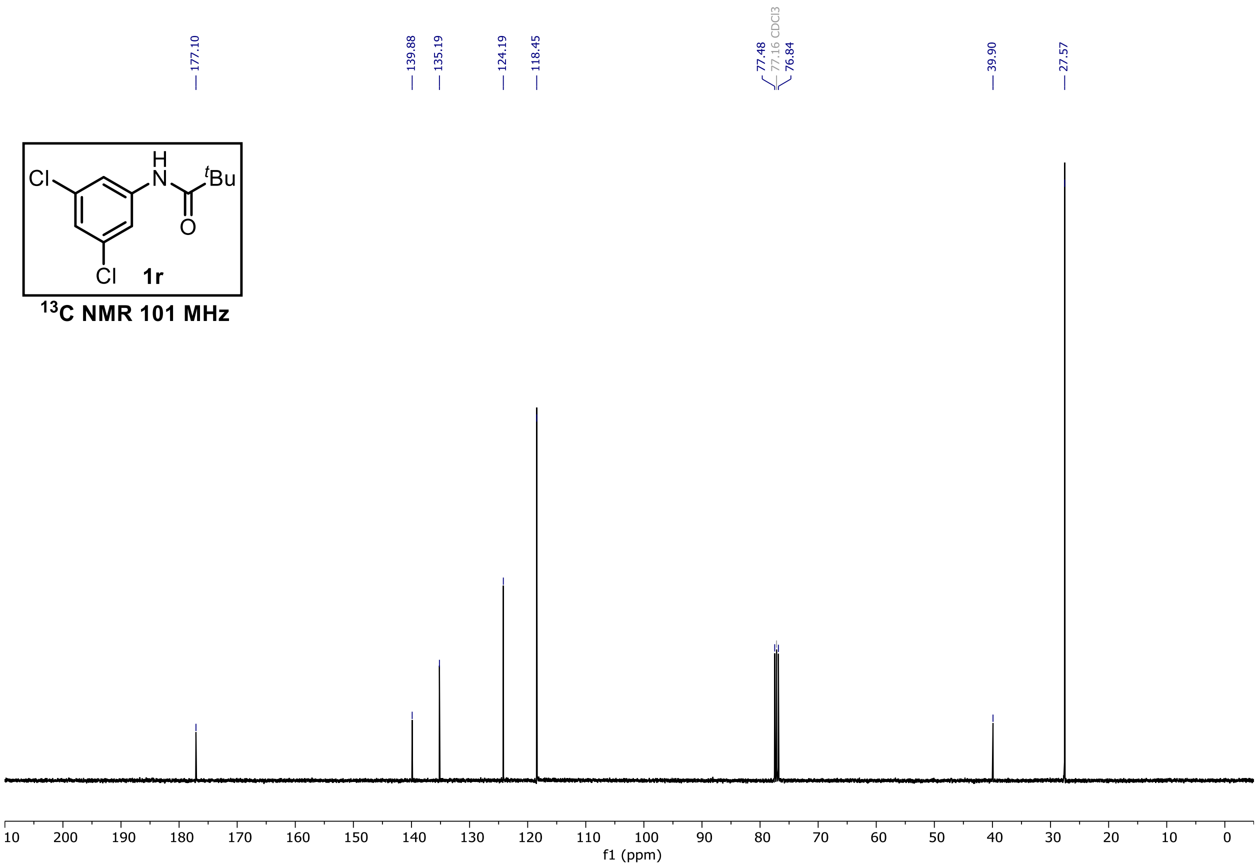
1r
¹³C NMR 101 MHz
177.10
139.88
135.19
124.19
118.45
77.48
77.16 CDCl3
76.84
39.90
27.57
f1 (ppm)

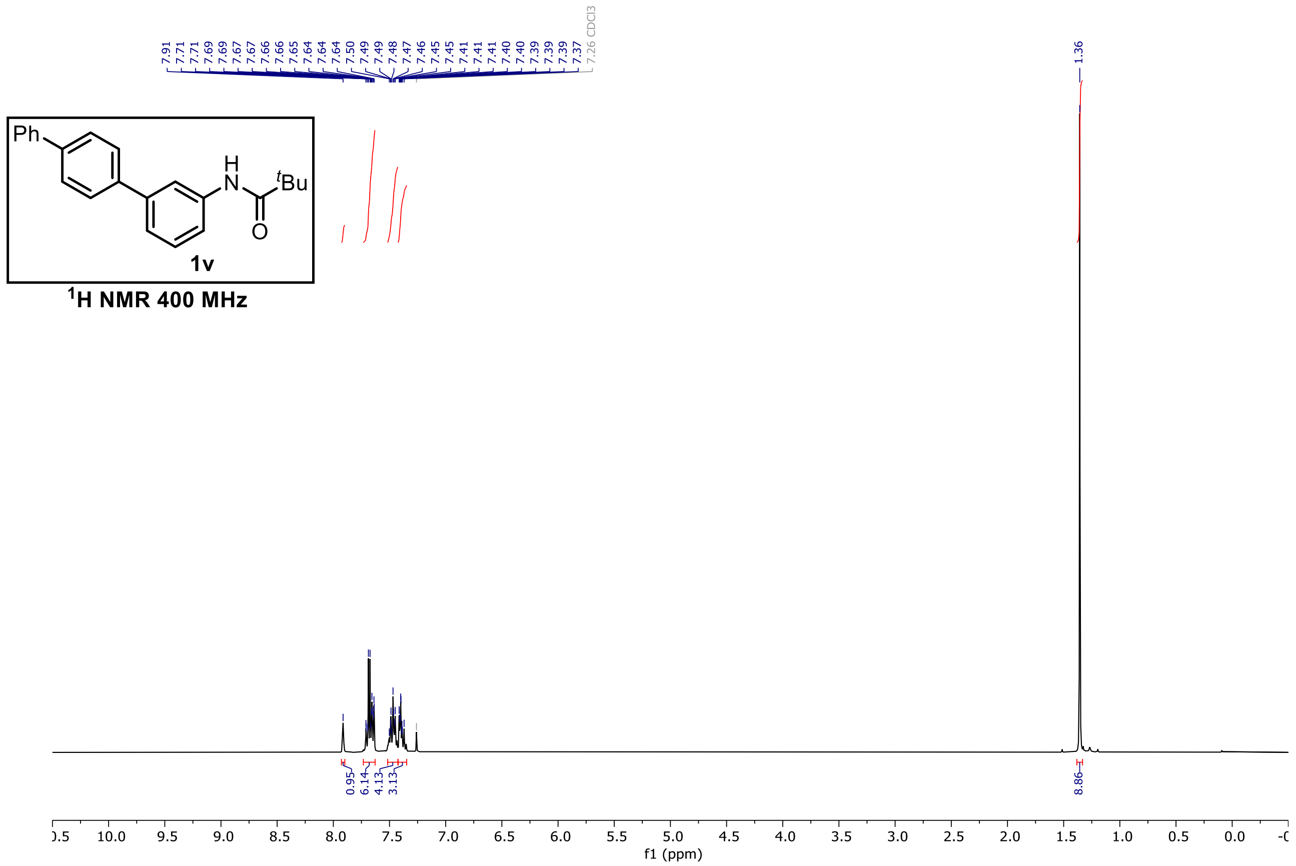
Ph
H
N
tBu
O
1v
1H NMR 400 MHz
7.91
7.71
7.71
7.69
7.69
7.67
7.66
7.66
7.65
7.64
7.64
7.64
7.50
7.49
7.49
7.48
7.47
7.46
7.45
7.45
7.41
7.41
7.41
7.40
7.40
7.39
7.39
7.39
7.37
7.26 CDCl3
1.36
0.95
6.14
4.13
3.13
8.86
f1 (ppm)

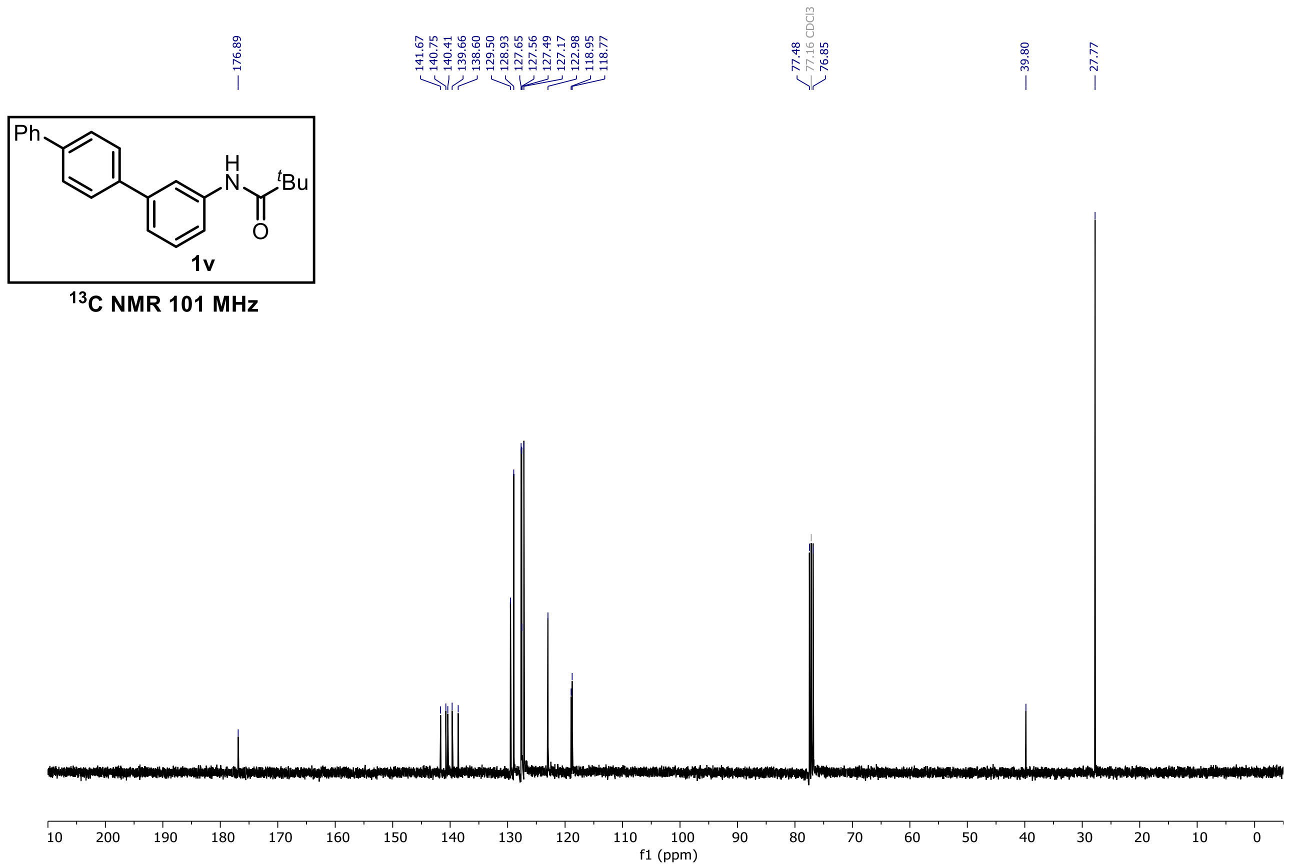
Ph
H
N
tBu
O
1v
13C NMR 101 MHz
176.89
141.67
140.75
140.41
139.66
138.60
129.50
128.93
127.65
127.56
127.49
127.17
122.98
118.95
118.77
77.48
77.16 CDCl3
76.85
39.80
27.77
f1 (ppm)

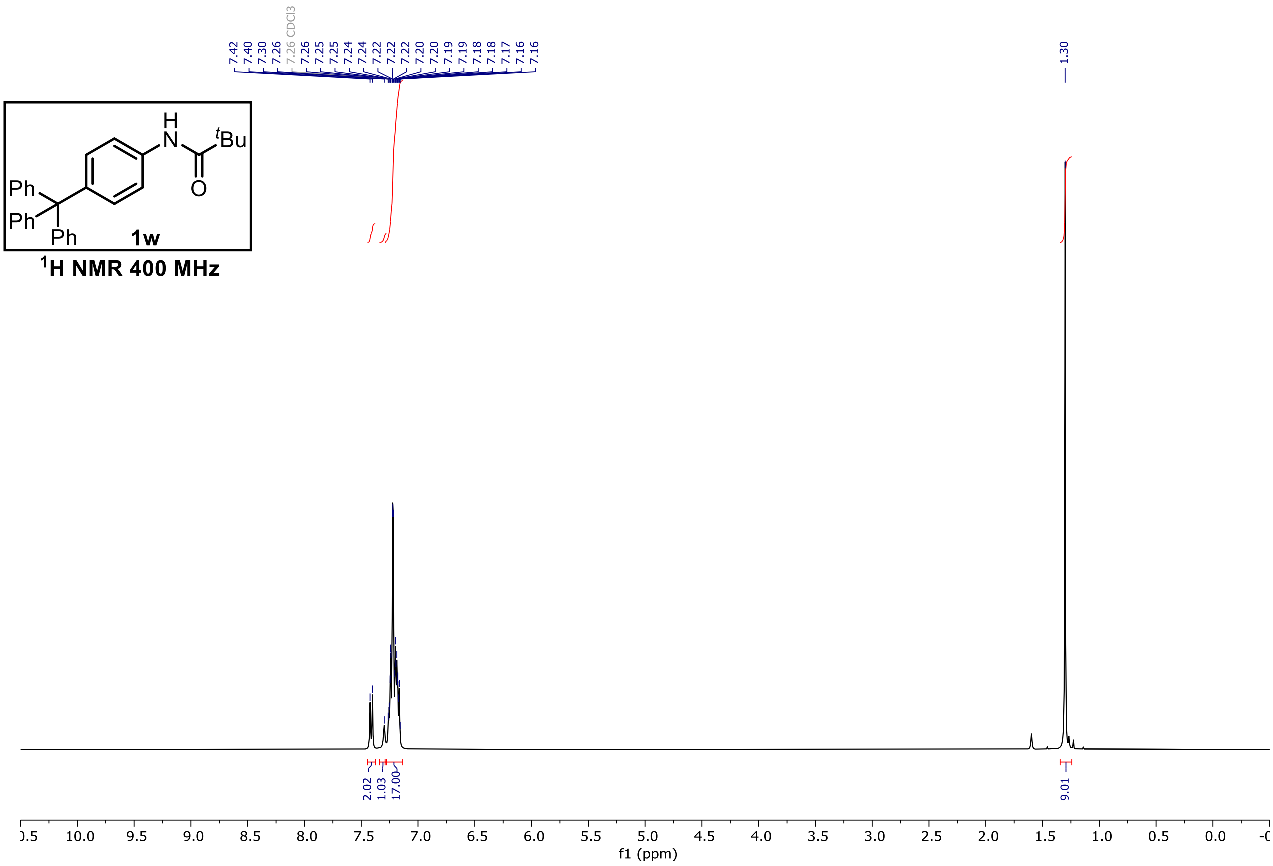

Ph
Ph
Ph
1w
tBu
H
N
O
1H NMR 400 MHz
7.42 7.40 7.30 7.26 7.26 CDCl3 7.26 7.25 7.25 7.24 7.24 7.22 7.22 7.22 7.20 7.20 7.19 7.19 7.18 7.18 7.17 7.16 7.16
1.30
2.02 1.03 17.00
9.01
f1 (ppm)

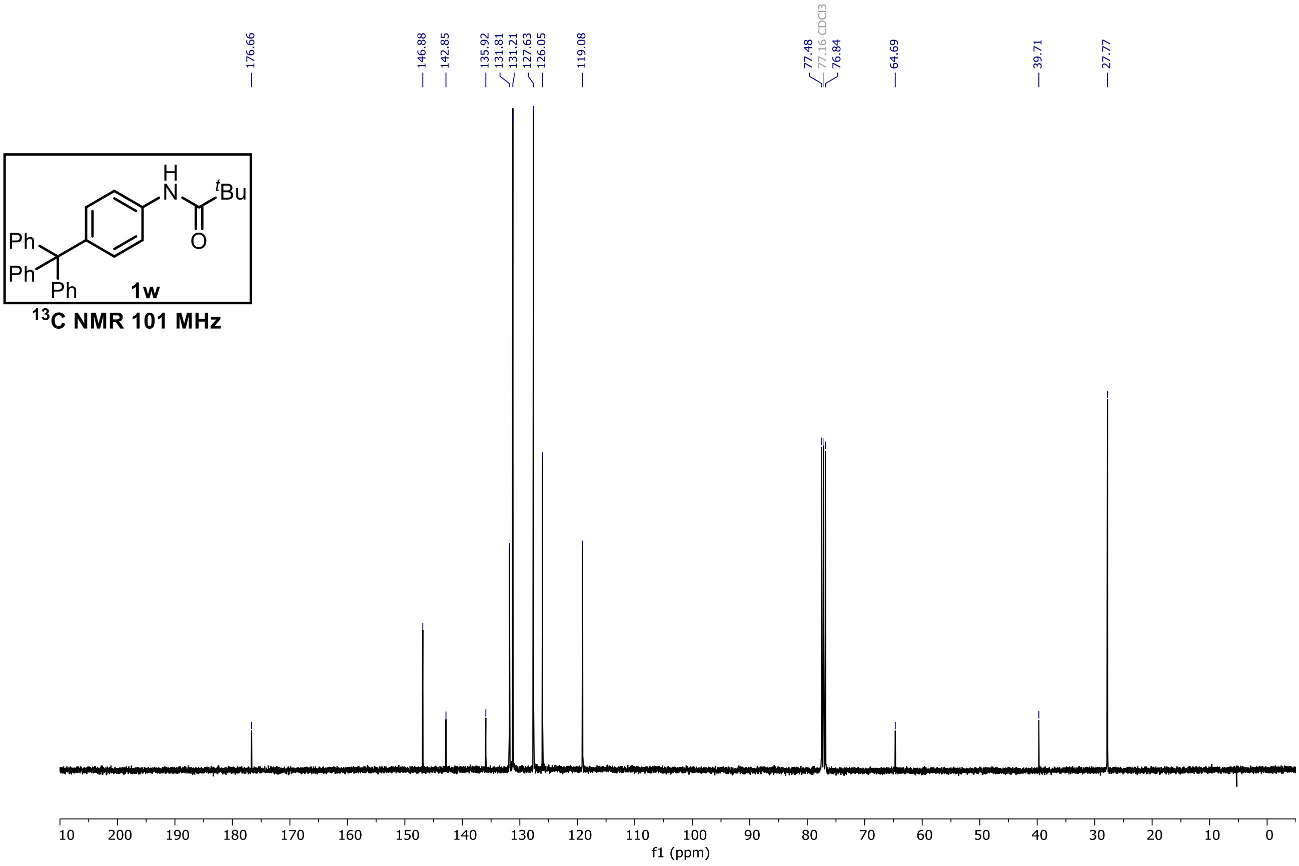

Ph
Ph
Ph
1w
tBu
H
N
O
13C NMR 101 MHz
176.66
146.88 142.85 135.92 131.81 131.21 127.63 126.05 119.08
77.48 77.16 CDCl3 76.84
64.69
39.71
27.77
f1 (ppm)

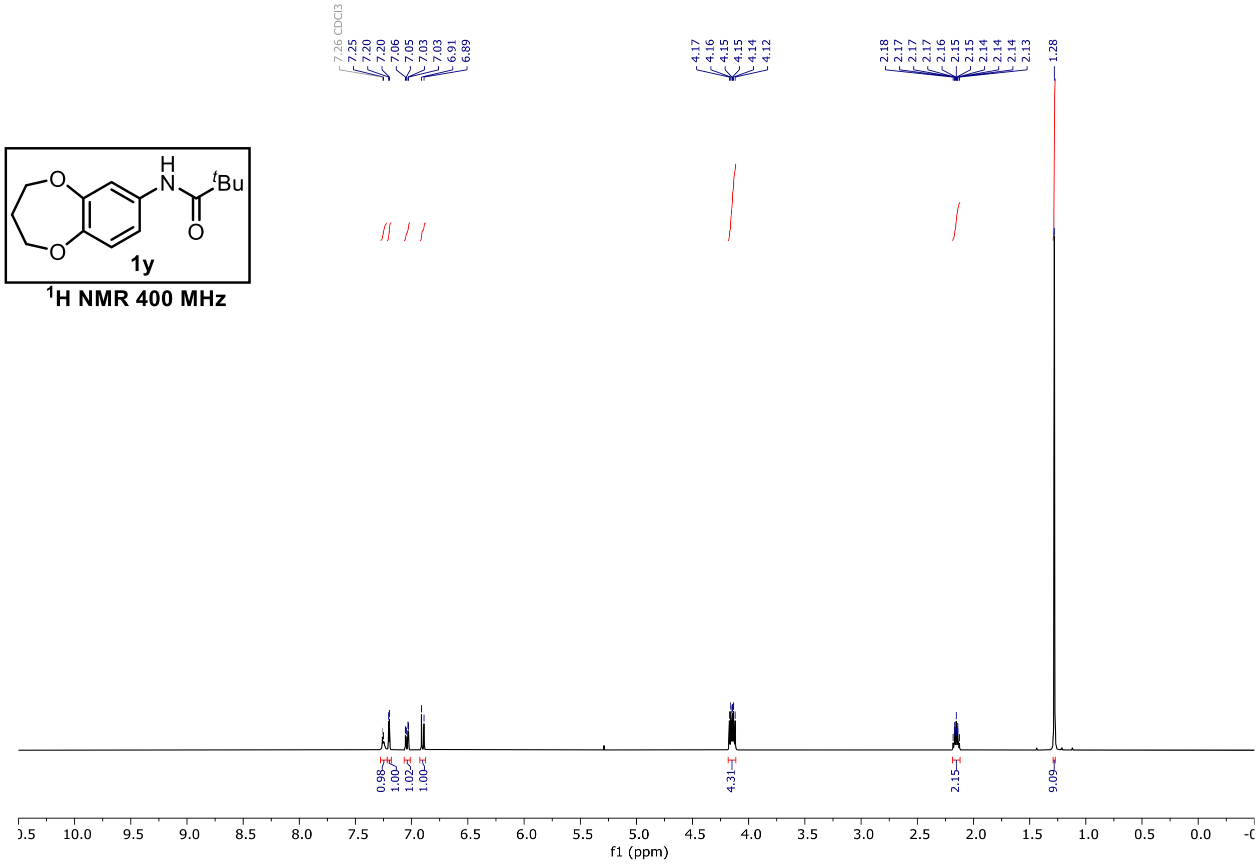
1y
1H NMR 400 MHz
7.26 CDCl3
7.25
7.20
7.20
7.06
7.05
7.03
7.03
6.91
6.89
4.17
4.16
4.15
4.15
4.14
4.12
2.18
2.17
2.17
2.17
2.16
2.15
2.15
2.14
2.14
2.14
2.13
1.28
0.98
1.00
1.02
1.00
4.31
2.15
9.09
f1 (ppm)

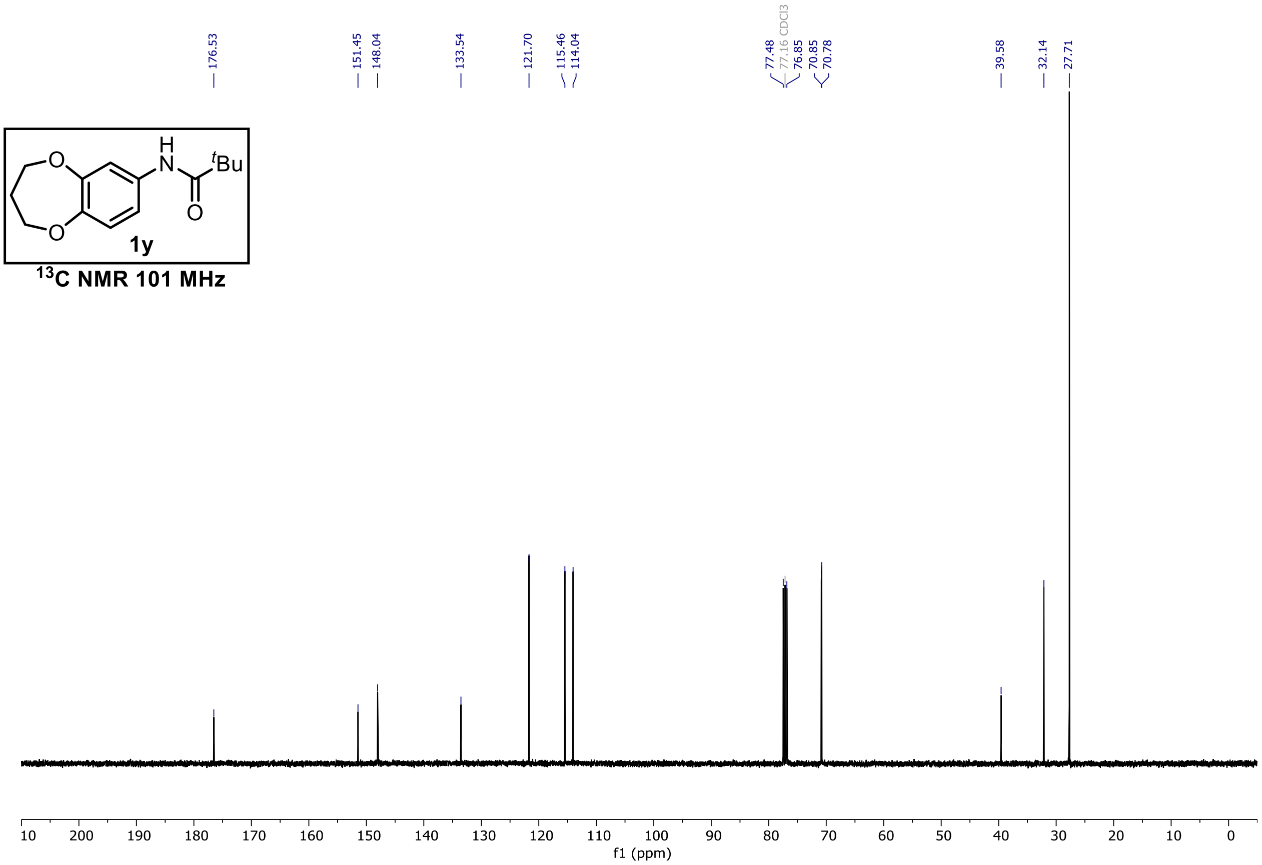
1y
13C NMR 101 MHz
176.53
151.45
148.04
133.54
121.70
115.46
114.04
77.48
77.16 CDCl3
76.85
70.85
70.78
39.58
32.14
27.71
f1 (ppm)

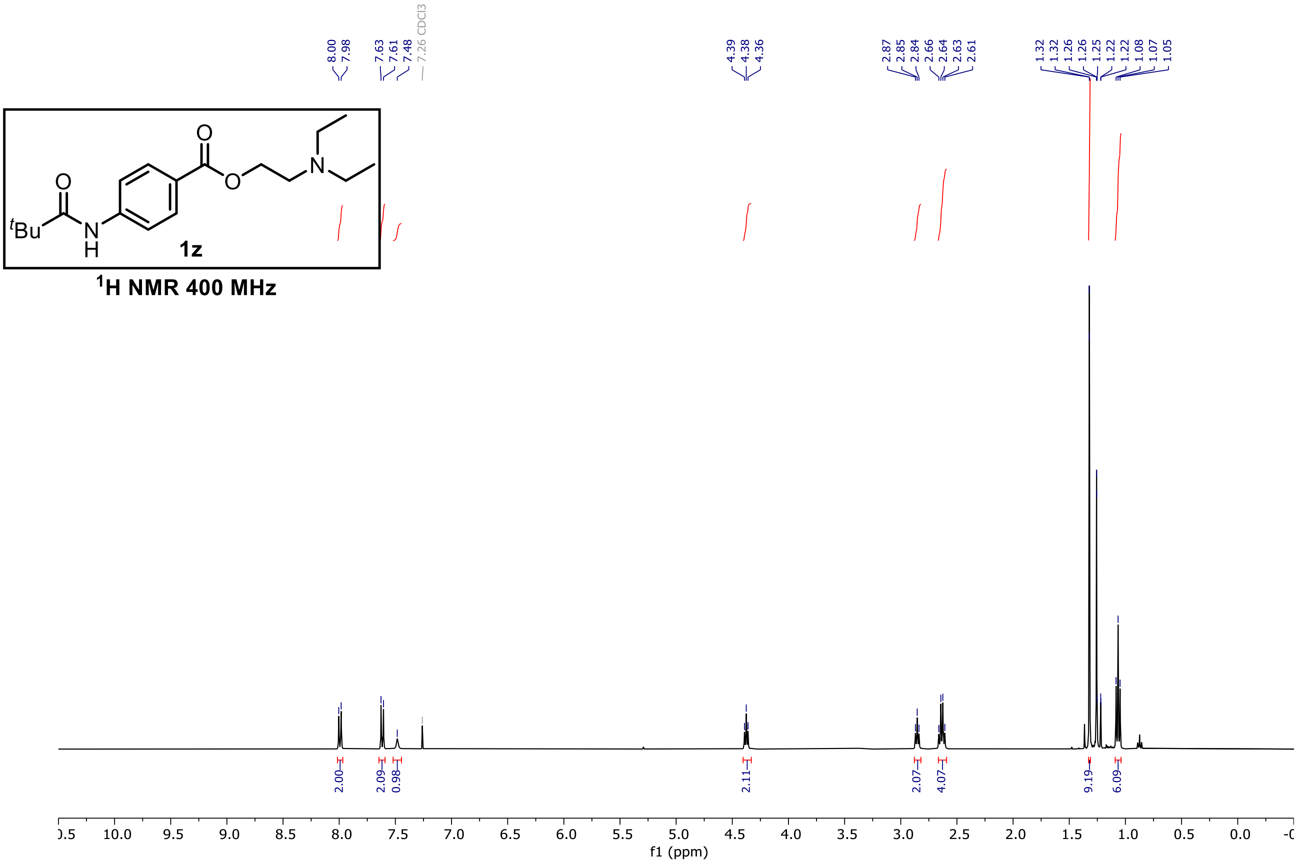

^{1}H NMR 400 MHz

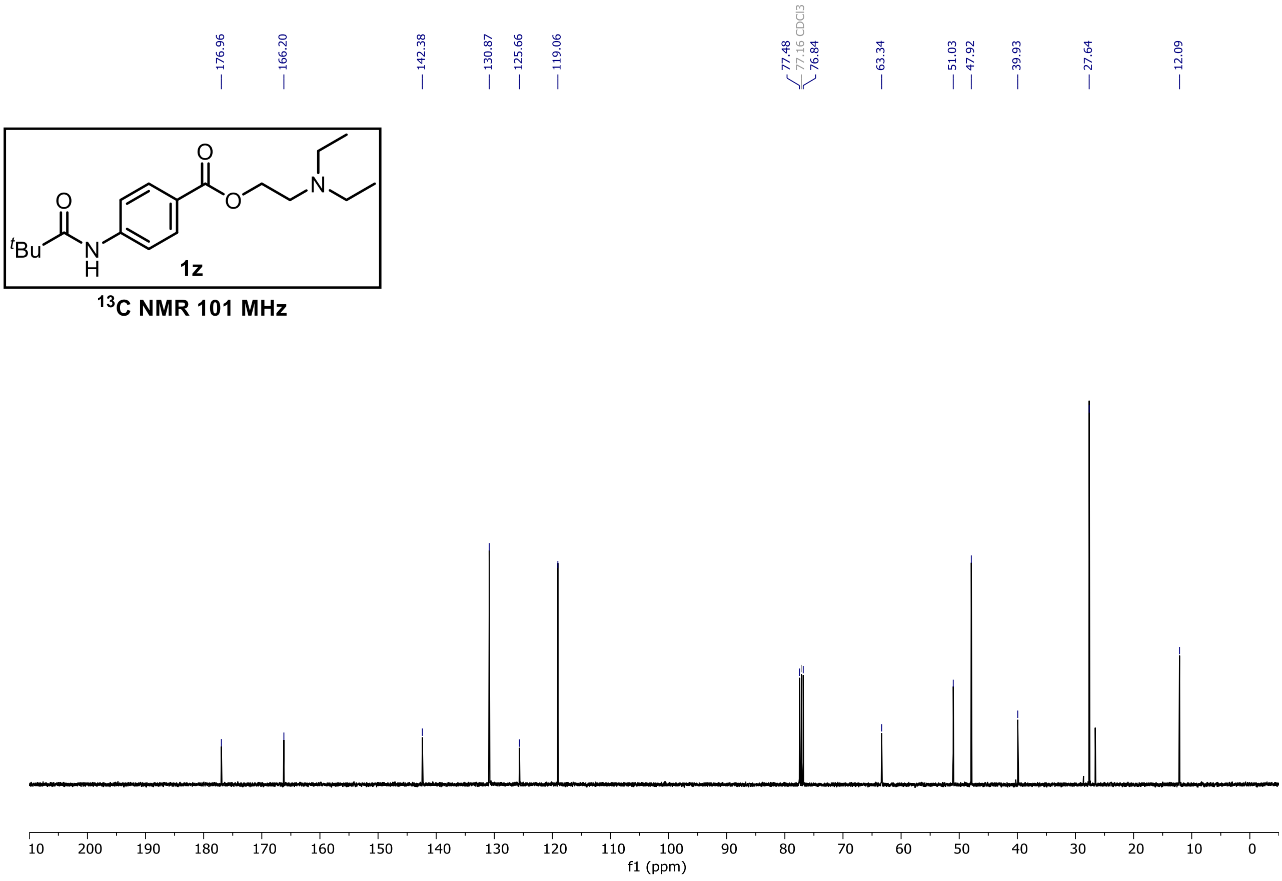

^{13}C NMR 101 MHz

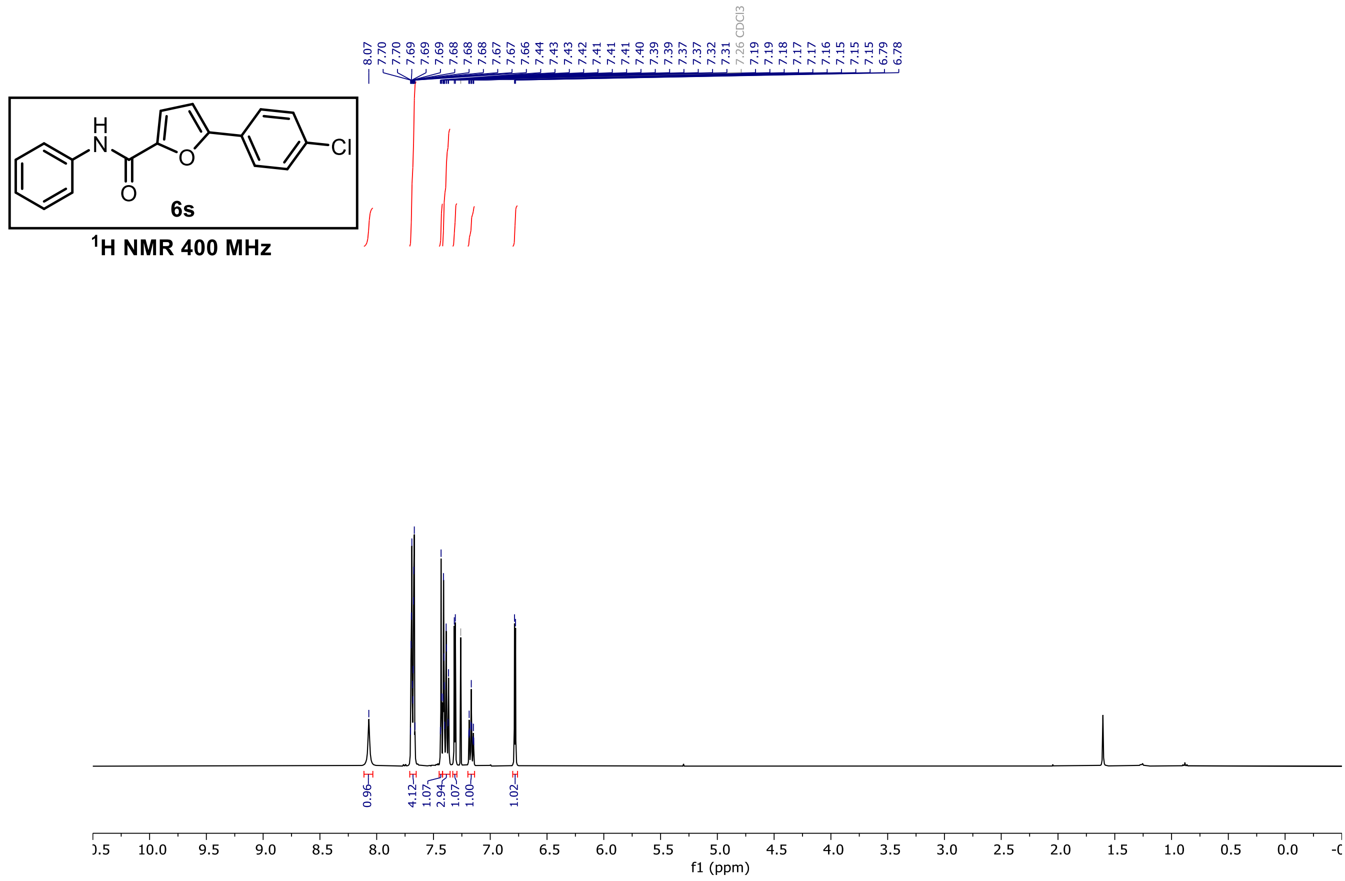
6s
1H NMR 400 MHz
8.07 7.70 7.70 7.69 7.69 7.69 7.68 7.68 7.68 7.67 7.67 7.66 7.44 7.43 7.43 7.42 7.41 7.41 7.41 7.40 7.39 7.39 7.37 7.37 7.37 7.32 7.31 7.26 CDCl3 7.19 7.19 7.18 7.17 7.17 7.16 7.15 7.15 7.15 6.79 6.78
0.96 4.12 1.07 2.94 1.07 1.00 1.02
f1 (ppm)

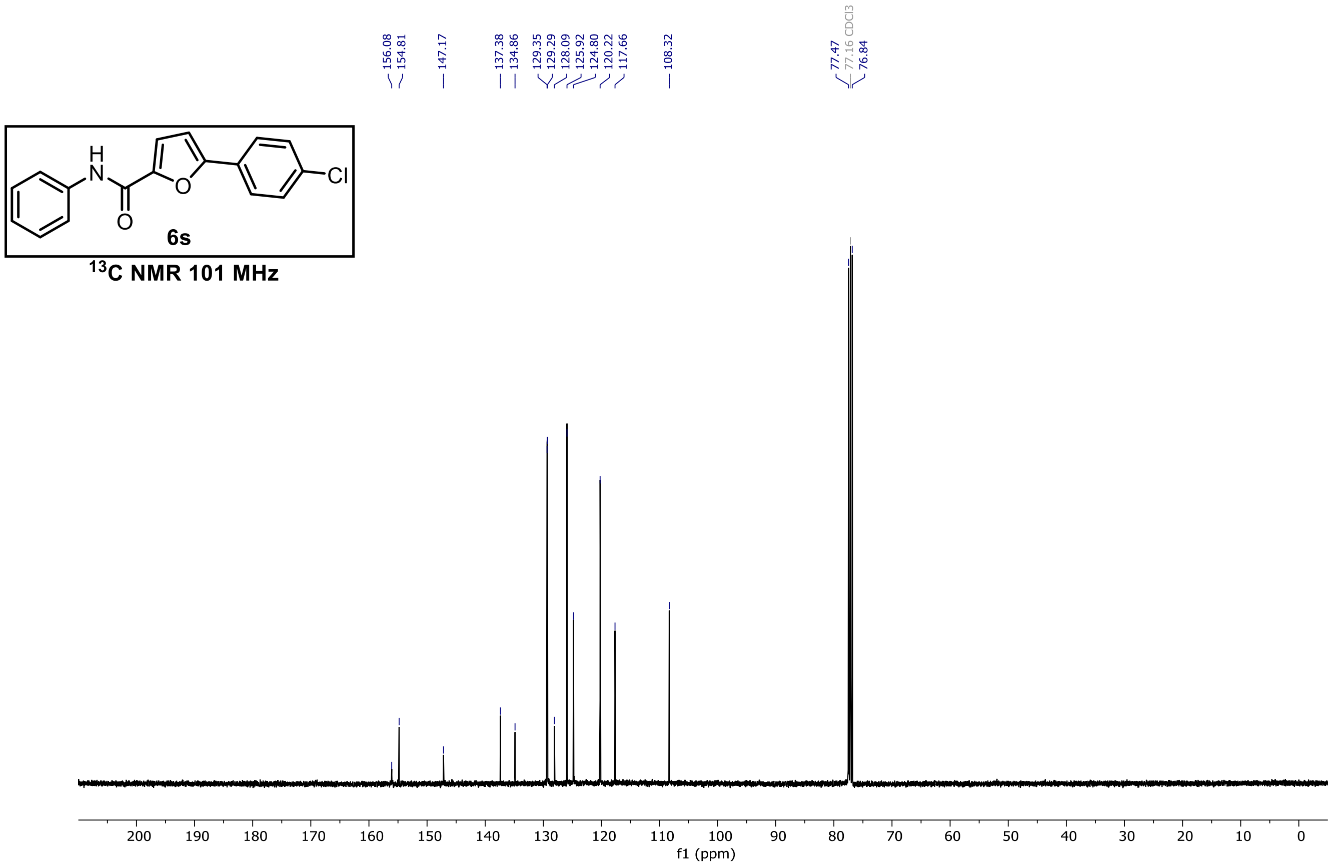
6s
13C NMR 101 MHz
156.08 154.81 147.17 137.38 134.86 129.35 129.29 128.09 125.92 124.80 120.22 117.66 108.32 77.47 77.16 CDCl3 76.84
f1 (ppm)

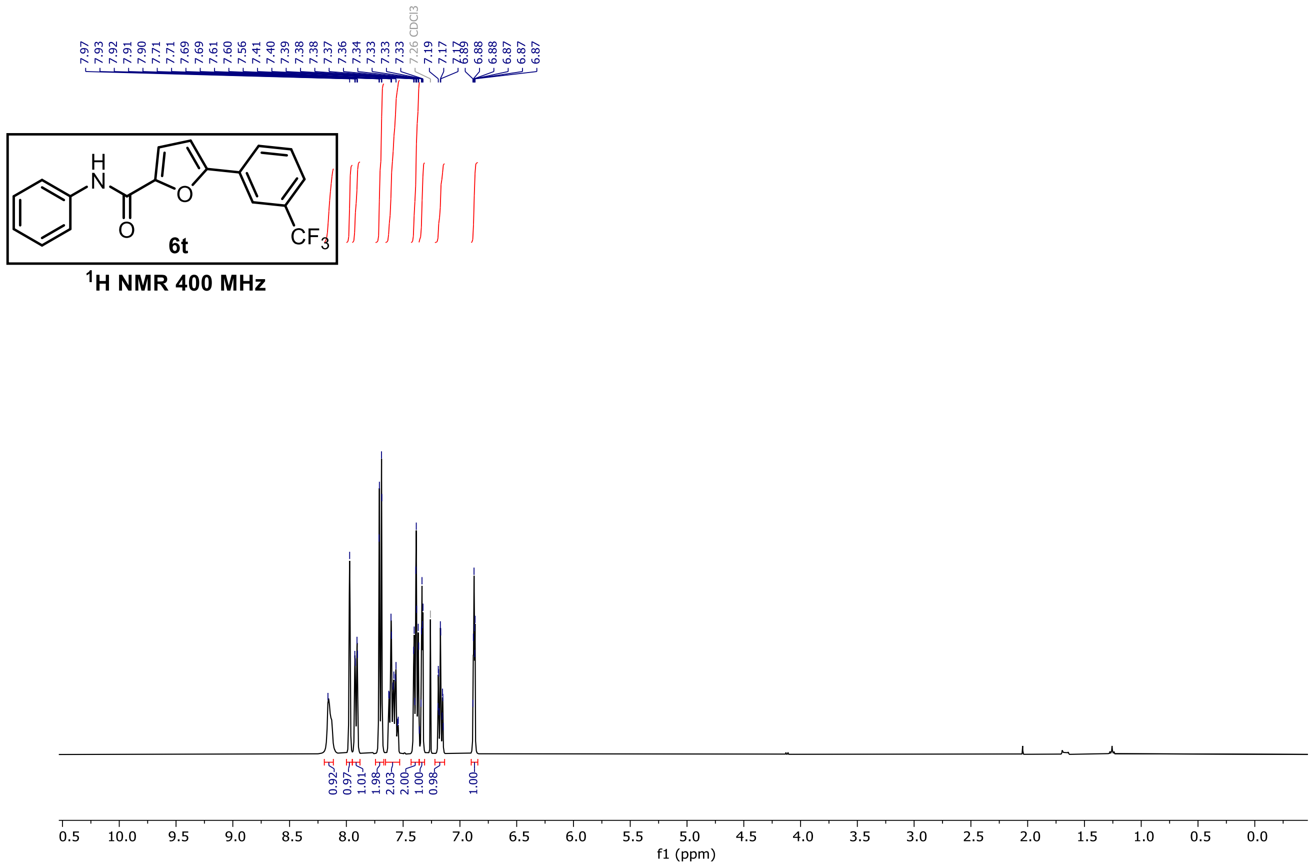
7.97
7.93
7.92
7.91
7.90
7.71
7.71
7.69
7.69
7.61
7.60
7.56
7.41
7.40
7.39
7.38
7.38
7.37
7.36
7.34
7.33
7.33
7.33
7.26 CDCl3
7.19
7.17
7.17
6.89
6.88
6.87
6.87
H
N
O
O
CF3
6t
1H NMR 400 MHz
0.92
0.97
1.01
1.98
2.03
2.00
1.00
0.98
1.00
f1 (ppm)

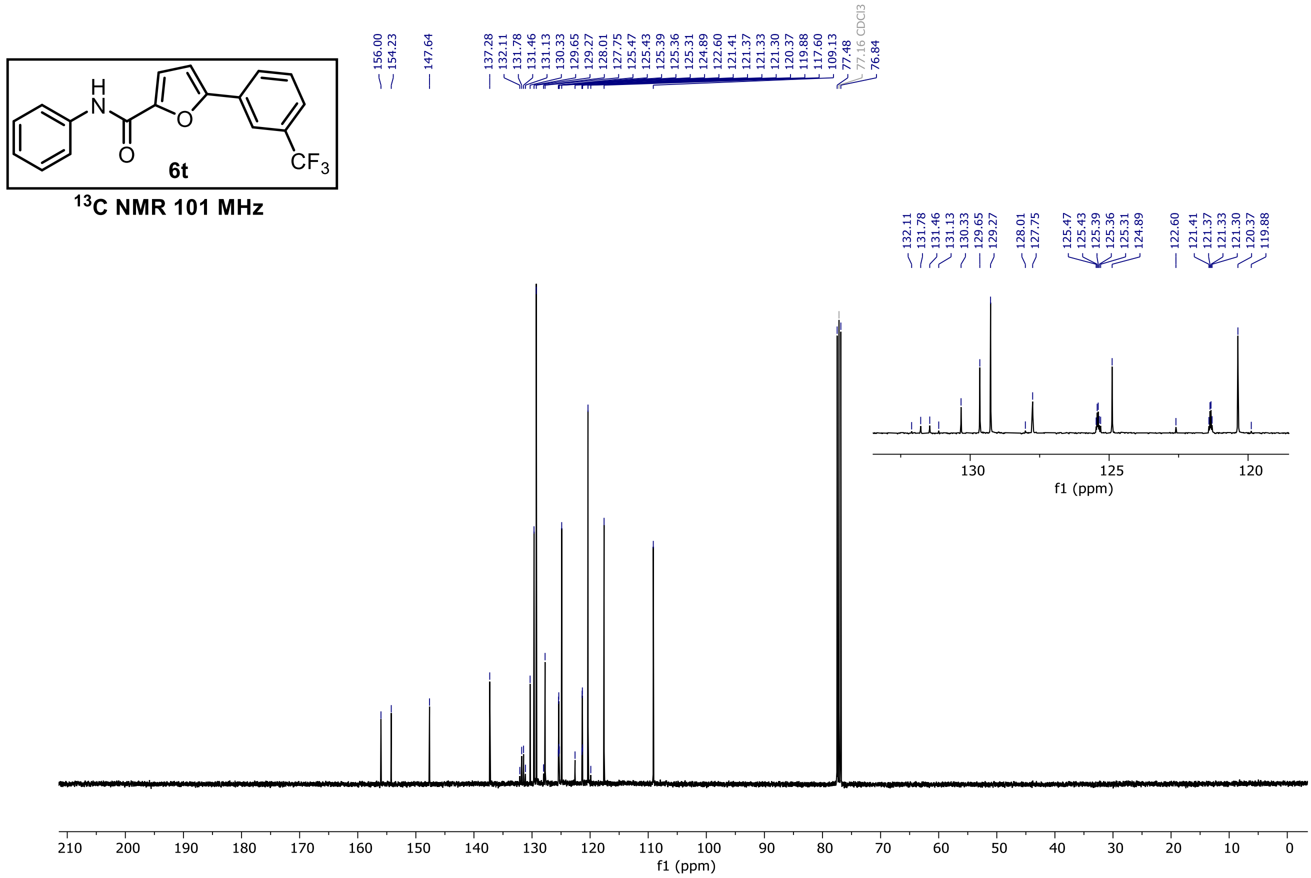
156.00
154.23
147.64
137.28
132.11
131.78
131.46
131.13
130.33
129.65
129.27
128.01
127.75
125.47
125.43
125.39
125.36
125.31
124.89
122.60
121.41
121.37
121.33
121.30
120.37
119.88
117.60
109.13
77.48
77.16 CDCl3
76.84
H
N
O
O
CF3
6t
13C NMR 101 MHz
f1 (ppm)

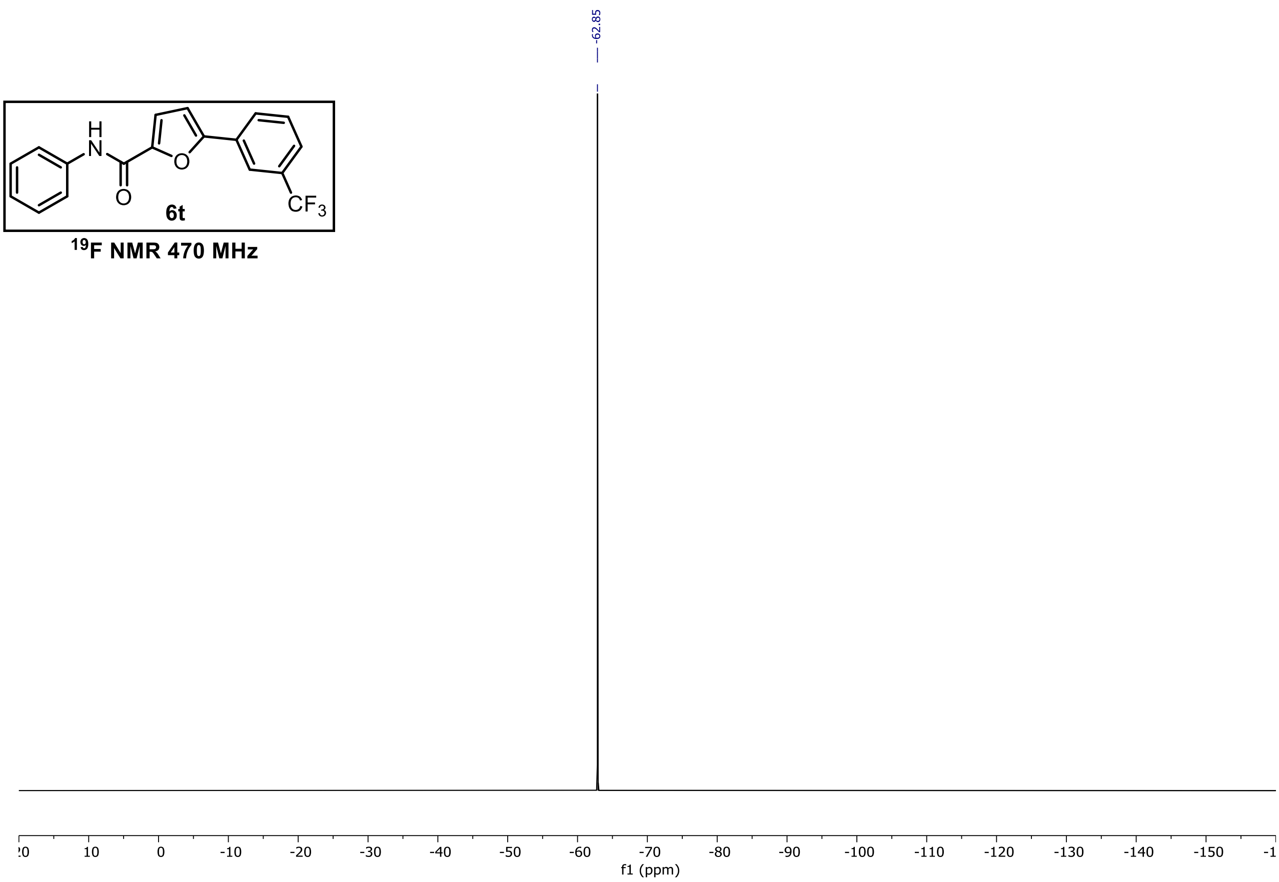

-62.85
6t
^{19}F NMR 470 MHz
f1 (ppm)

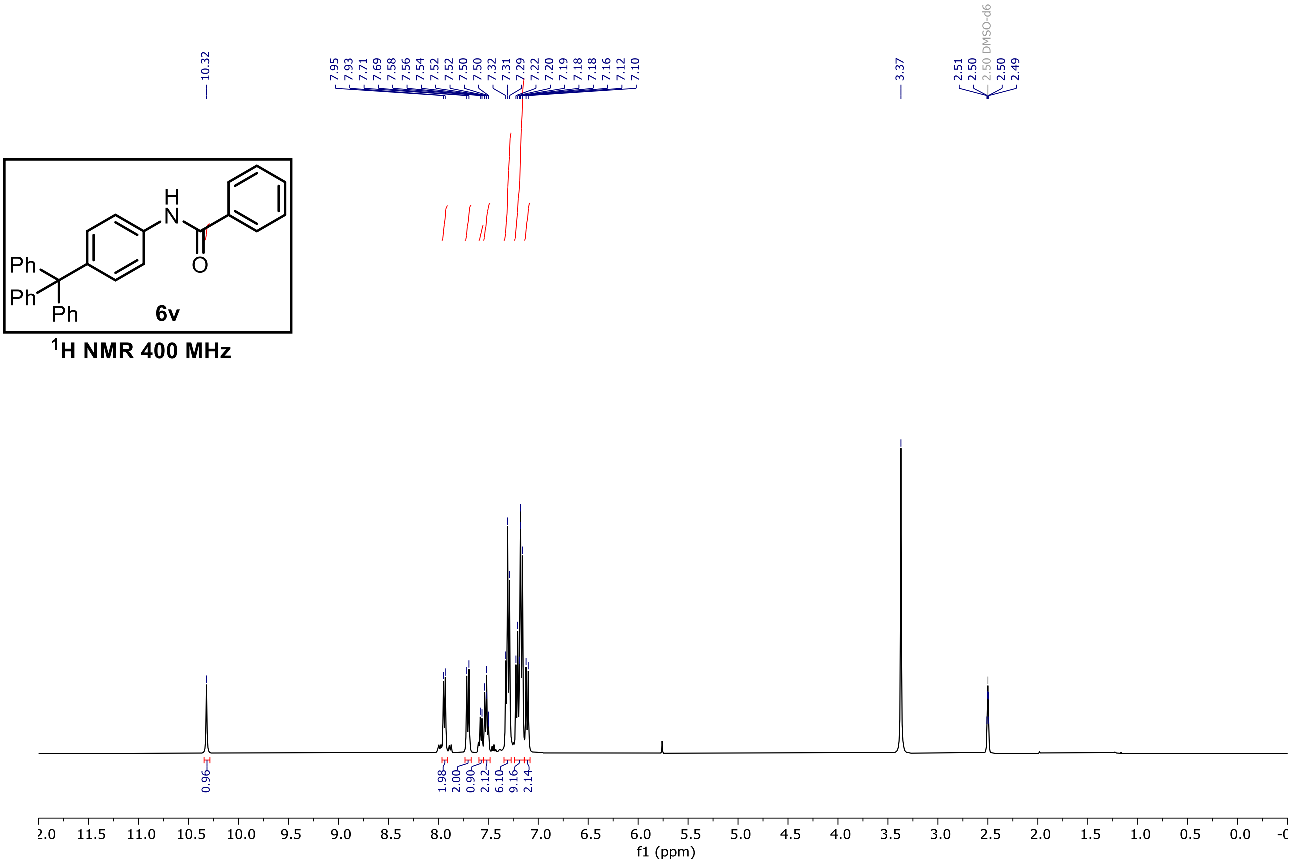

10.32
7.95
7.93
7.71
7.69
7.58
7.56
7.54
7.52
7.52
7.50
7.50
7.32
7.31
7.29
7.22
7.20
7.19
7.18
7.18
7.16
7.12
7.10
3.37
2.51
2.50
2.50 DMSO-d6
2.50
2.49
6v
^{1}H NMR 400 MHz
0.96
1.98
2.00
0.90
2.12
6.10
9.16
2.14
f1 (ppm)

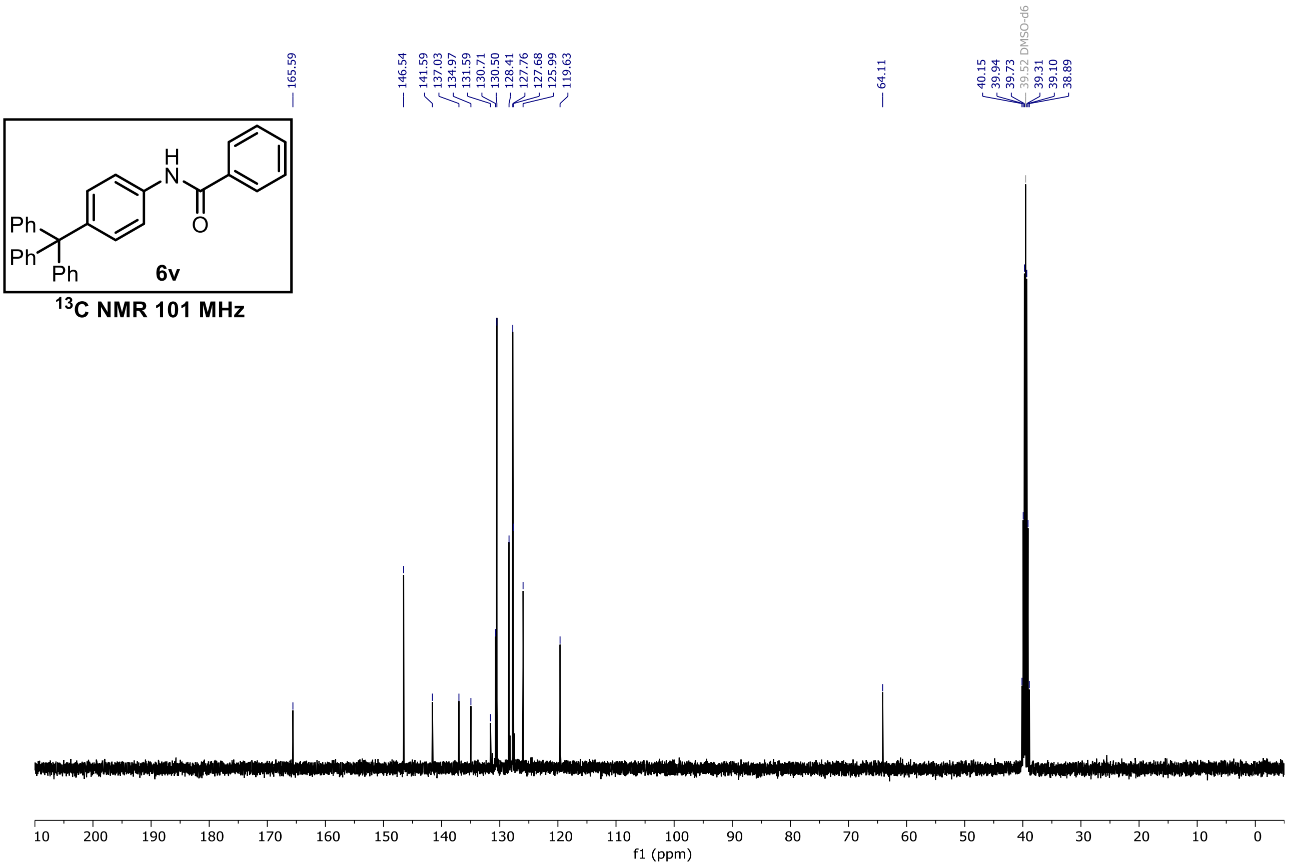
165.59
146.54
141.59
137.03
134.97
131.59
130.71
130.50
128.41
127.76
127.68
125.99
119.63
64.11
40.15
39.94
39.73
39.52 DMSO-d6
39.31
39.10
38.89
H
N
O
Ph
Ph
Ph
6v
13C NMR 101 MHz
f1 (ppm)

11.2. NMR spectra of target compounds

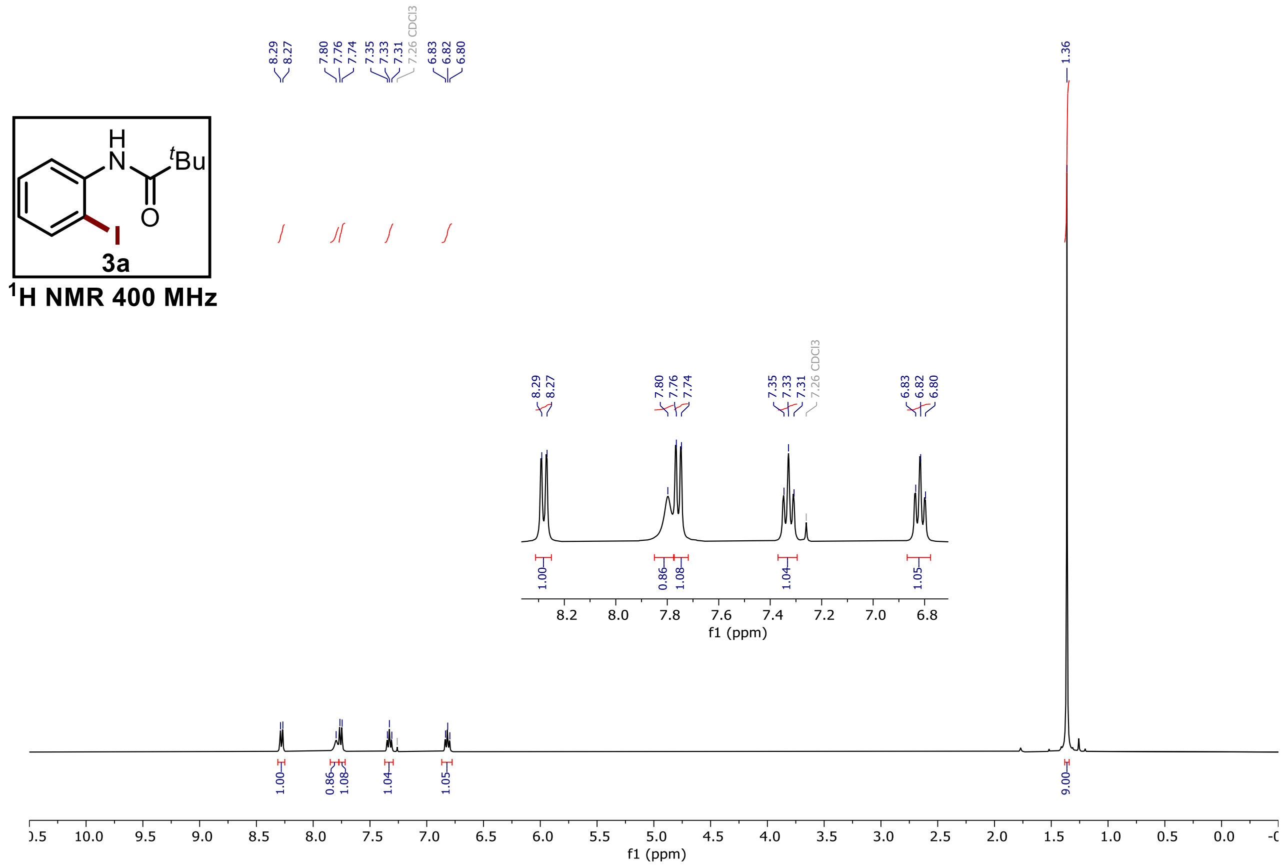
3a
1H NMR 400 MHz
f1 (ppm)

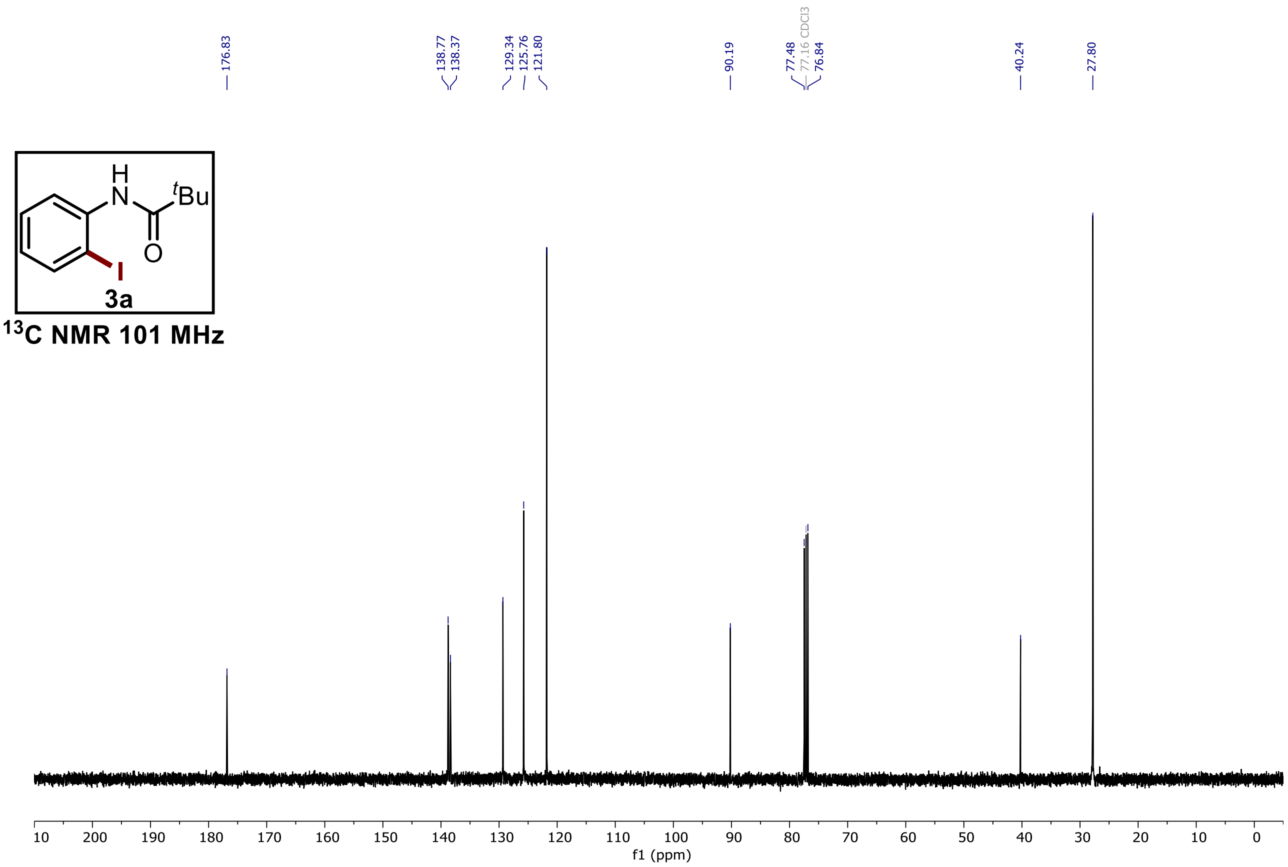
3a
13C NMR 101 MHz
f1 (ppm)

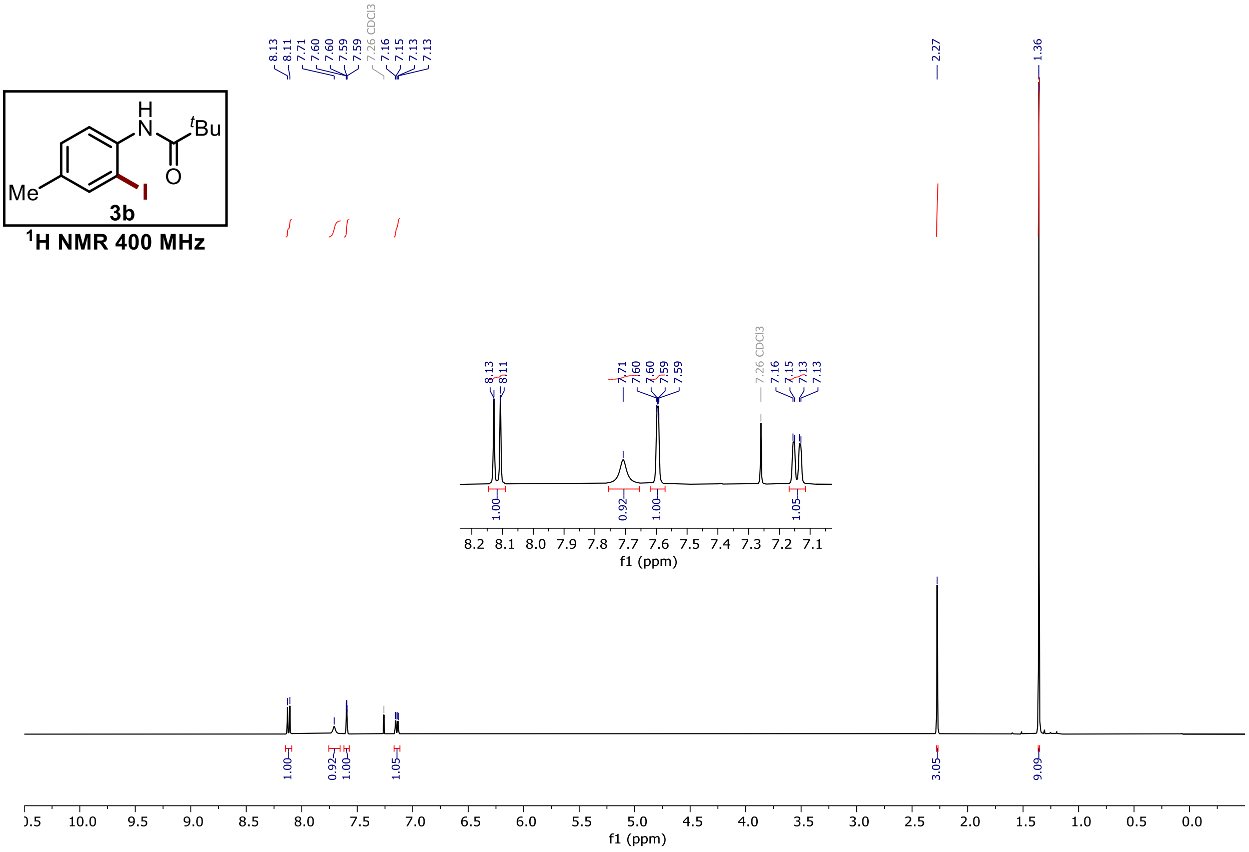
3b
1H NMR 400 MHz
Me
I
O
tBu
H
N
8.13
8.11
7.71
7.60
7.60
7.59
7.59
7.26 CDCl3
7.16
7.15
7.13
7.13
2.27
1.36
1.00
0.92
1.00
1.05
3.05
9.09
f1 (ppm)

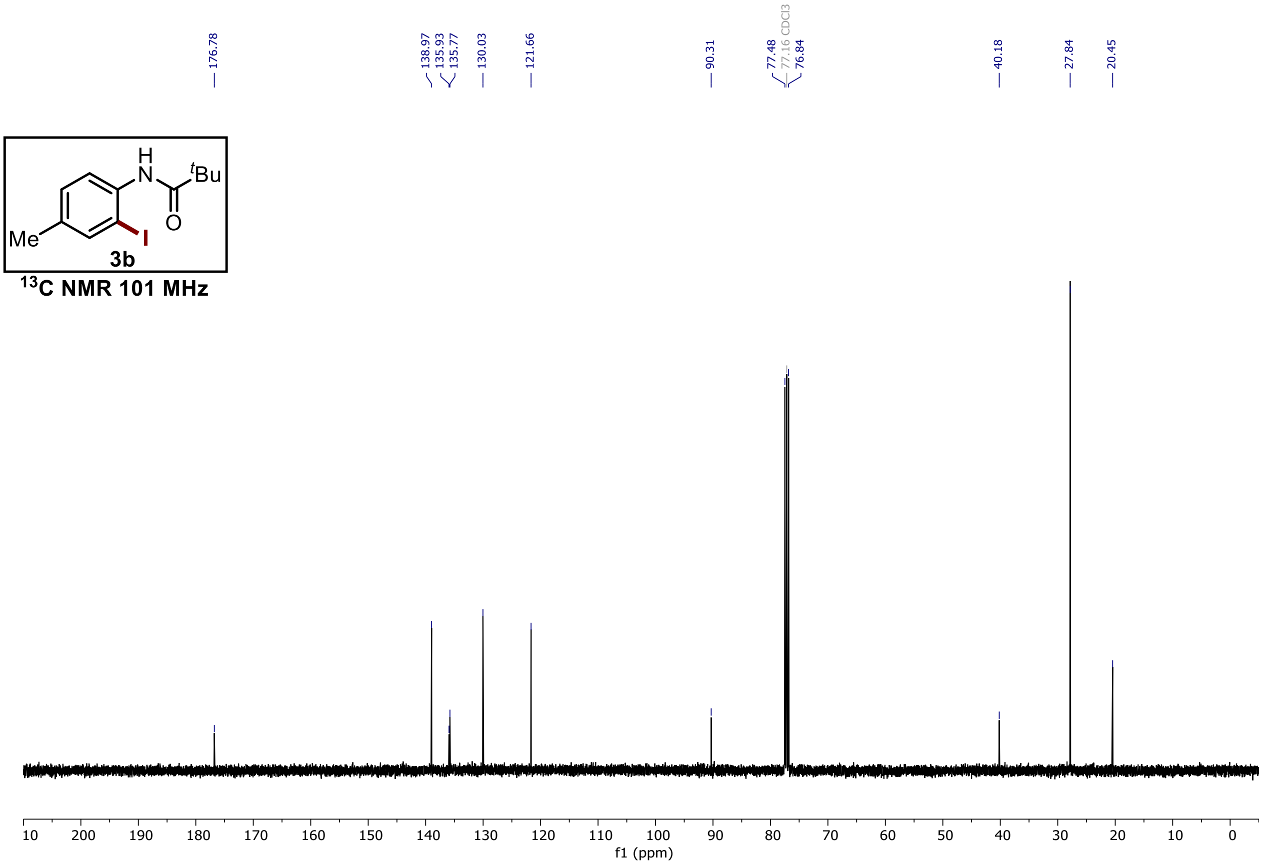
3b
13C NMR 101 MHz
Me
I
O
tBu
H
N
176.78
138.97
135.93
135.77
130.03
121.66
90.31
77.48
77.16 CDCl3
76.84
40.18
27.84
20.45
f1 (ppm)

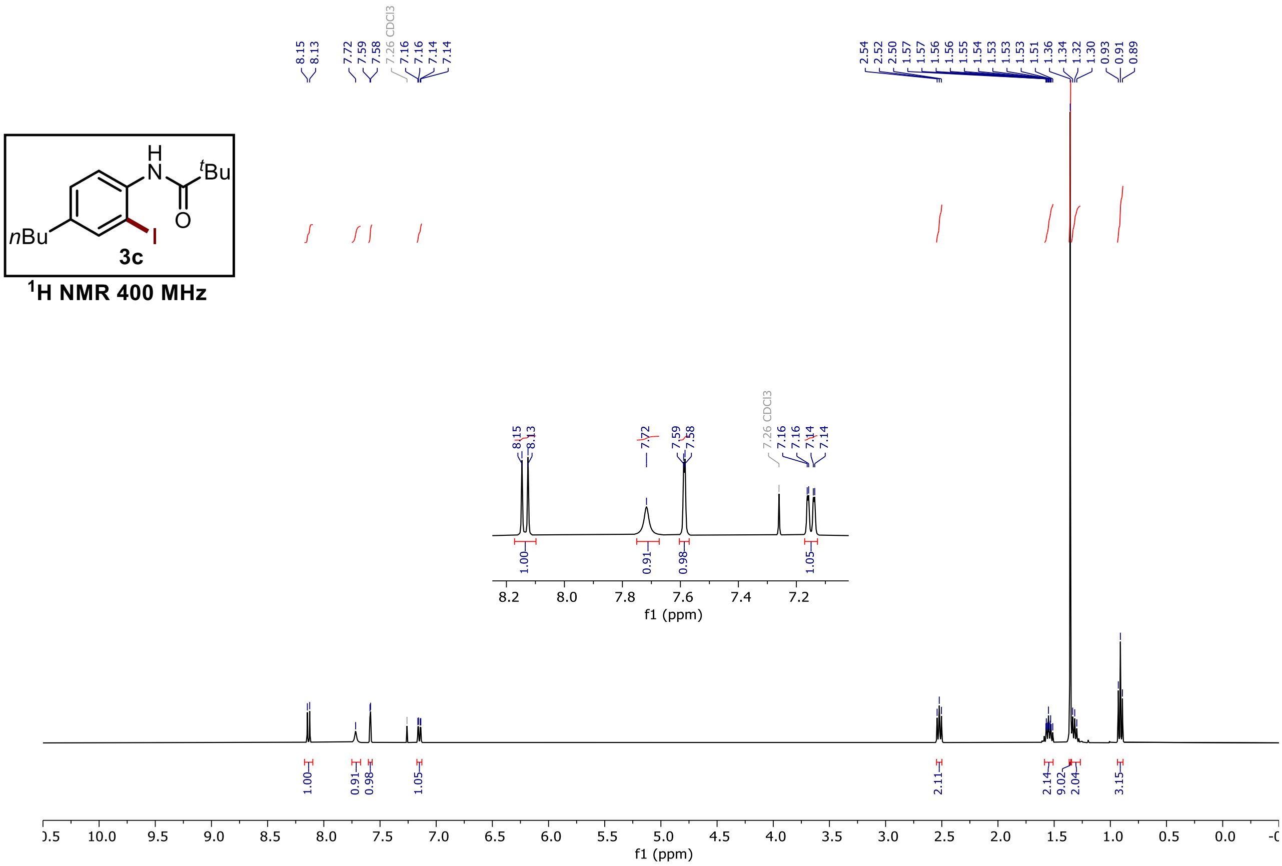

3c
^{1}H NMR 400 MHz
f1 (ppm)

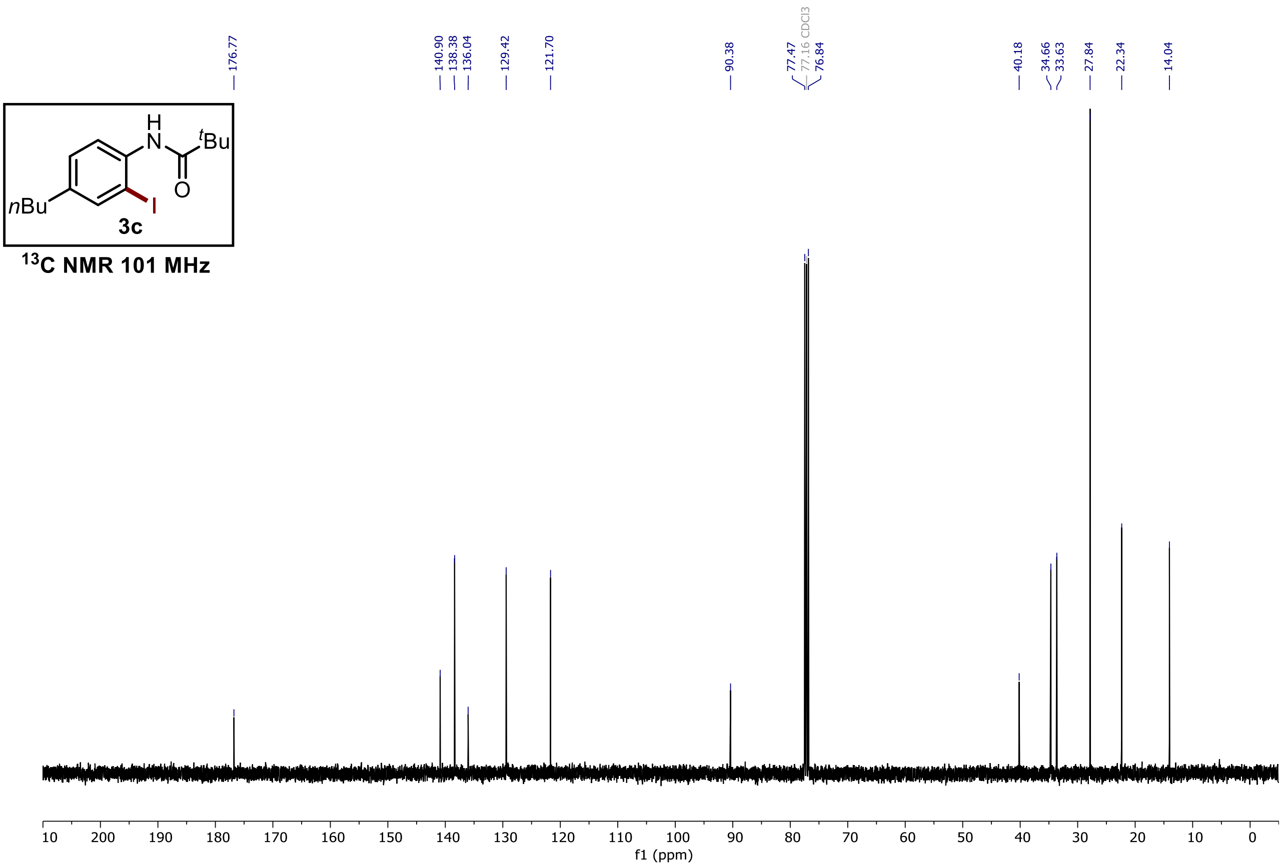

3c
^{13}C NMR 101 MHz
f1 (ppm)

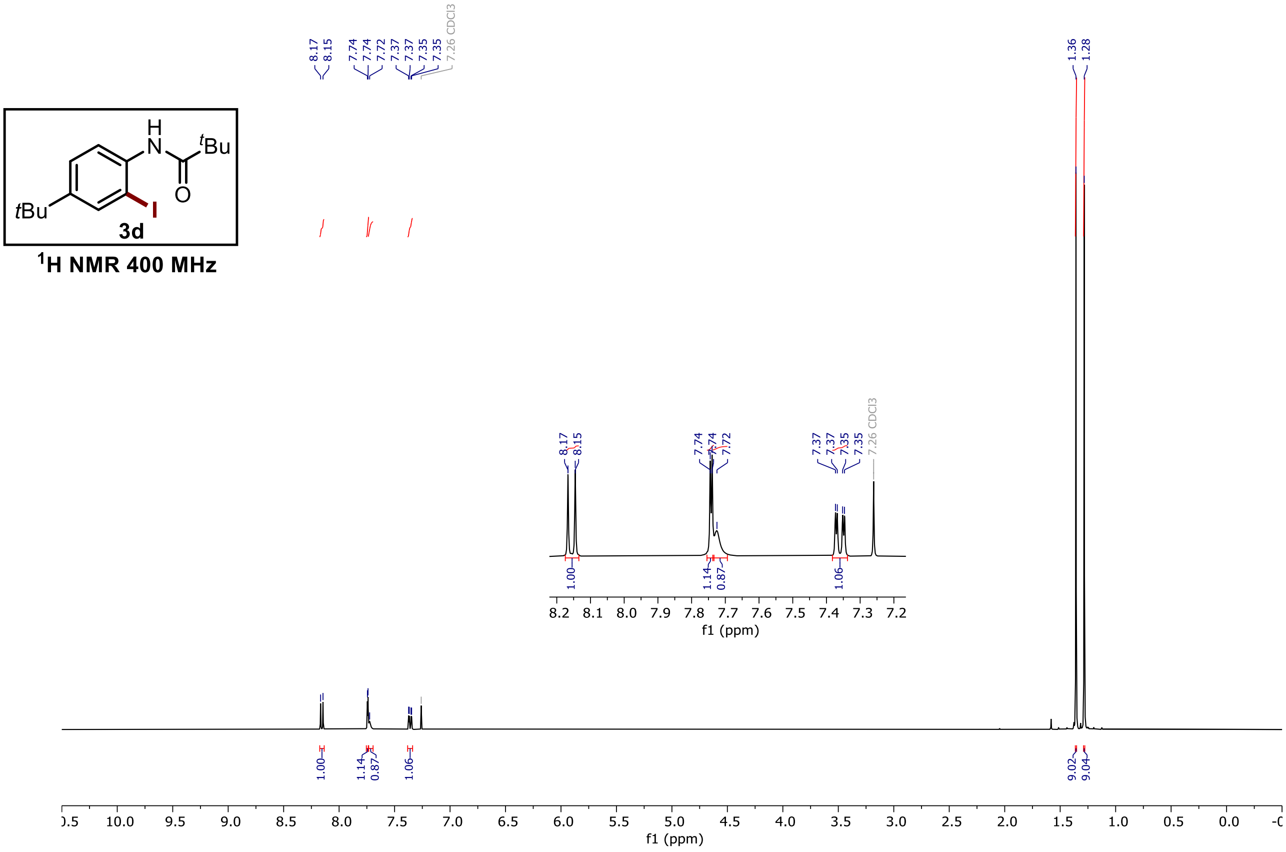

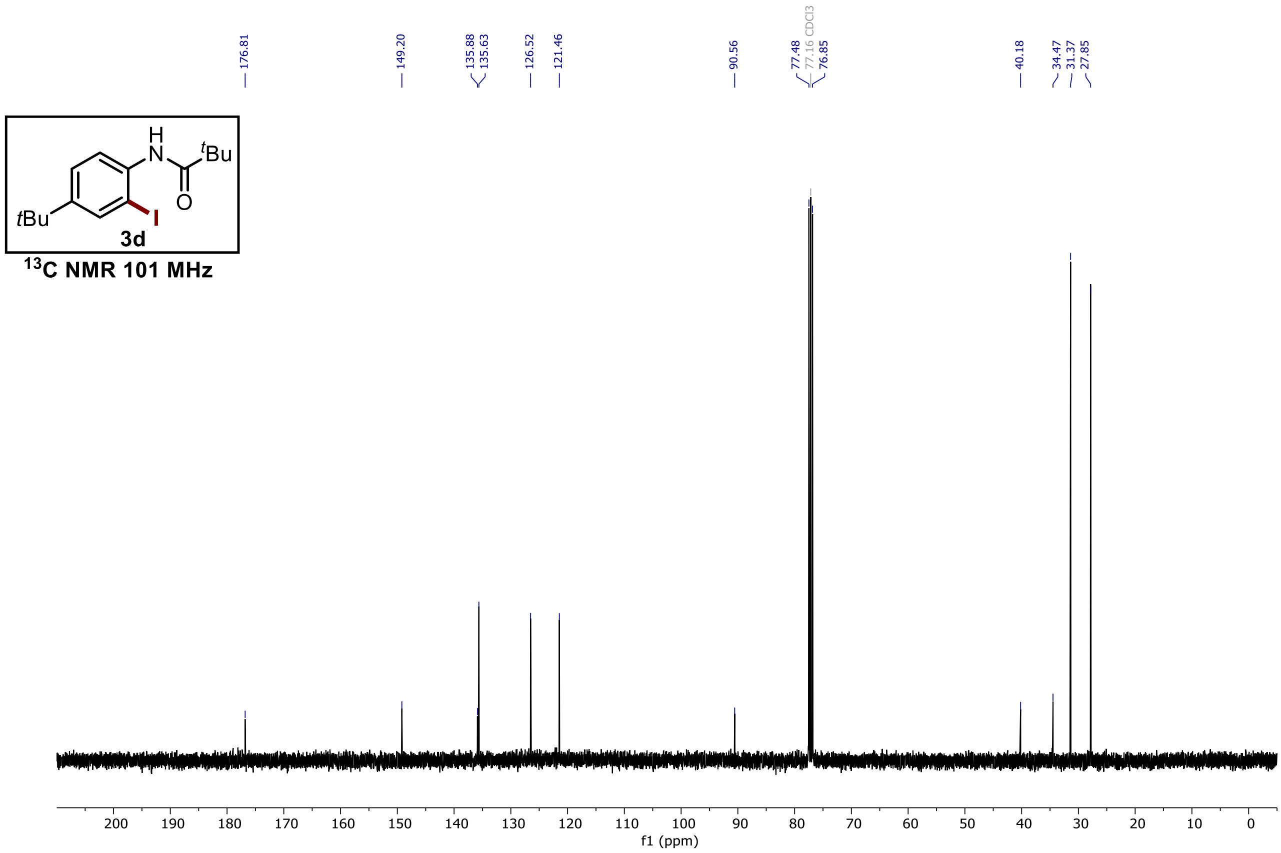

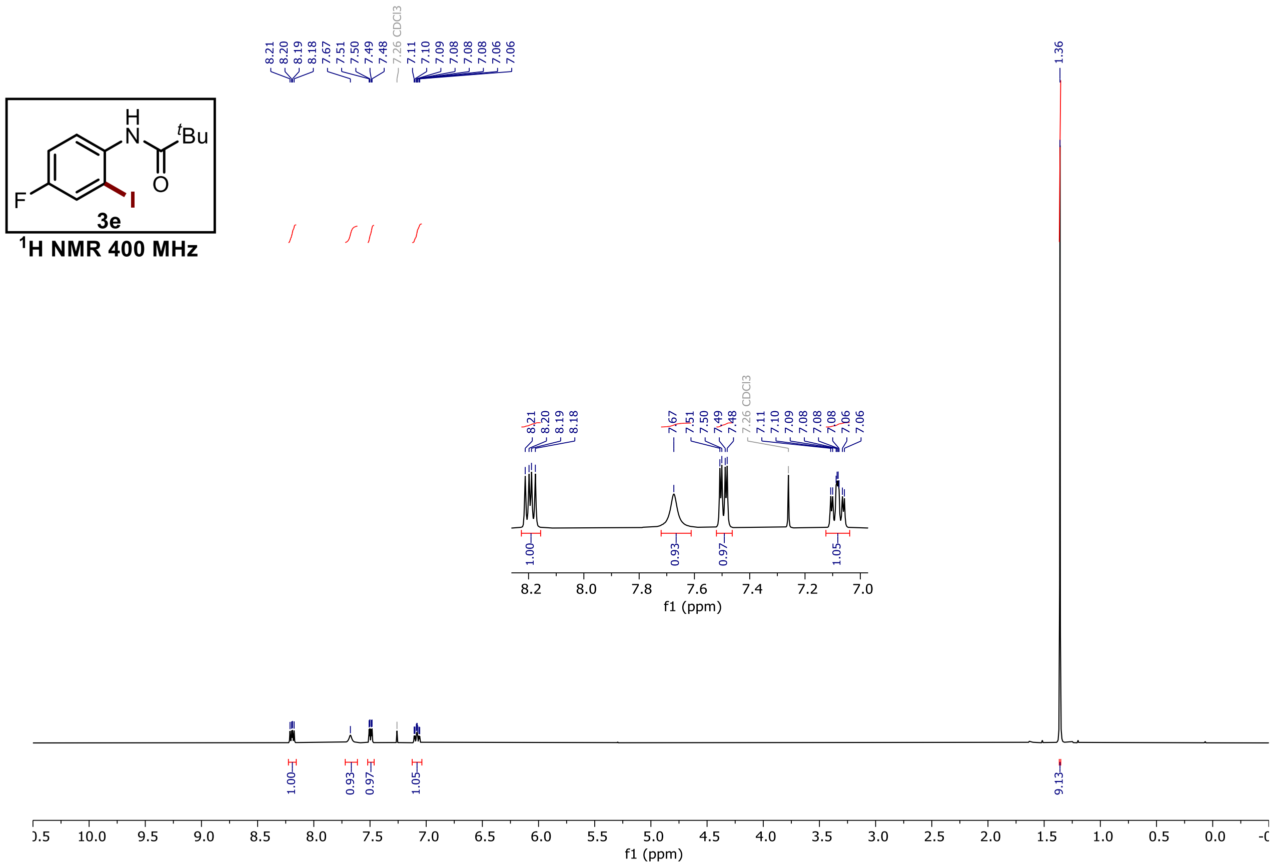
3e
1H NMR 400 MHz
f1 (ppm)

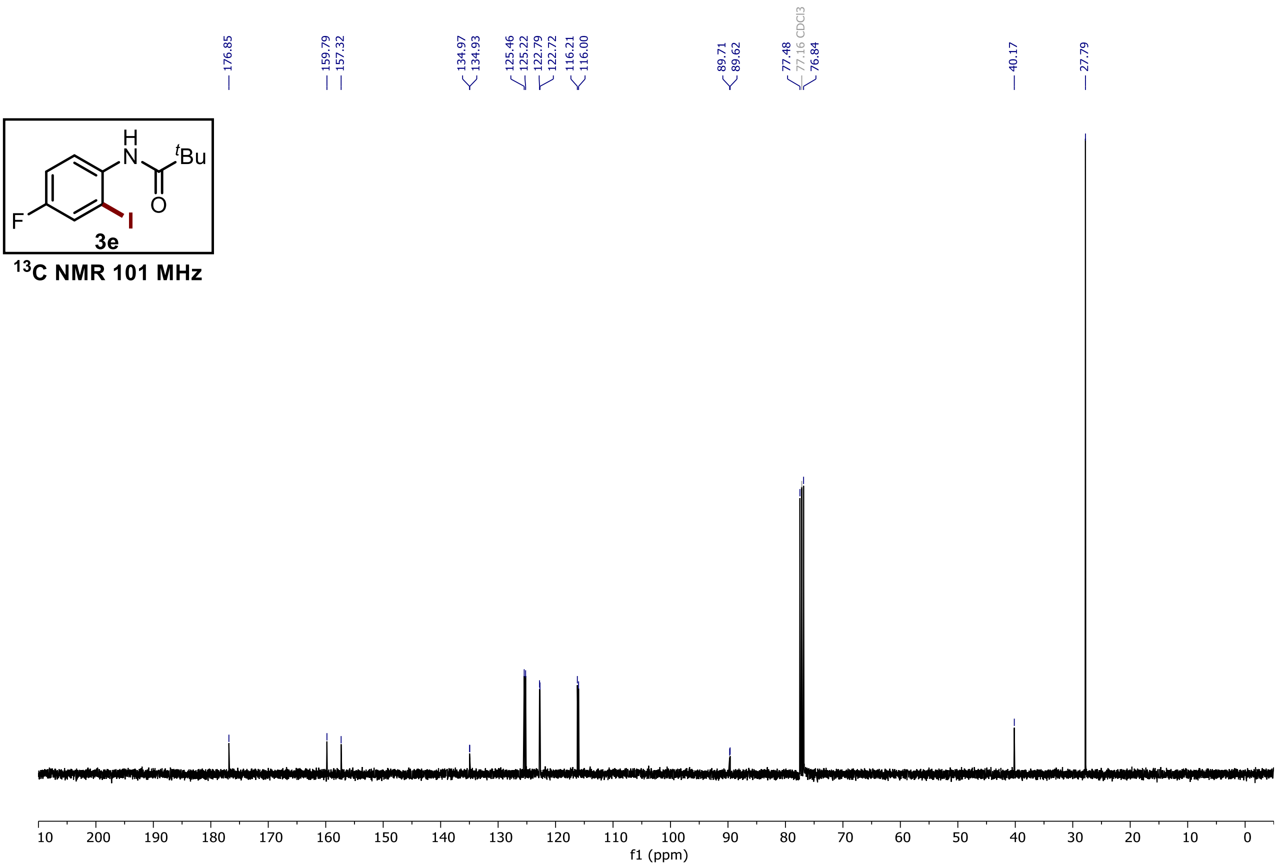
3e
13C NMR 101 MHz
f1 (ppm)

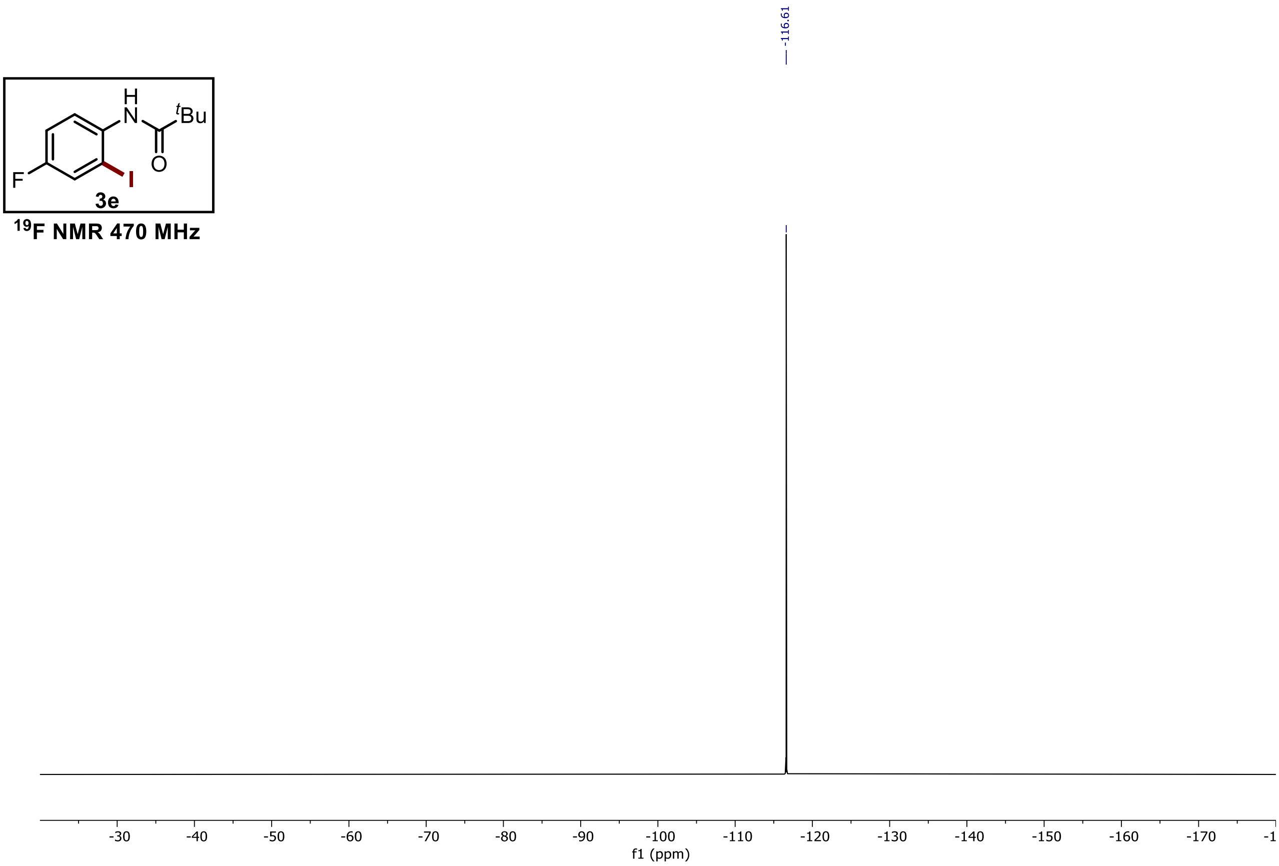
3e
19F NMR 470 MHz

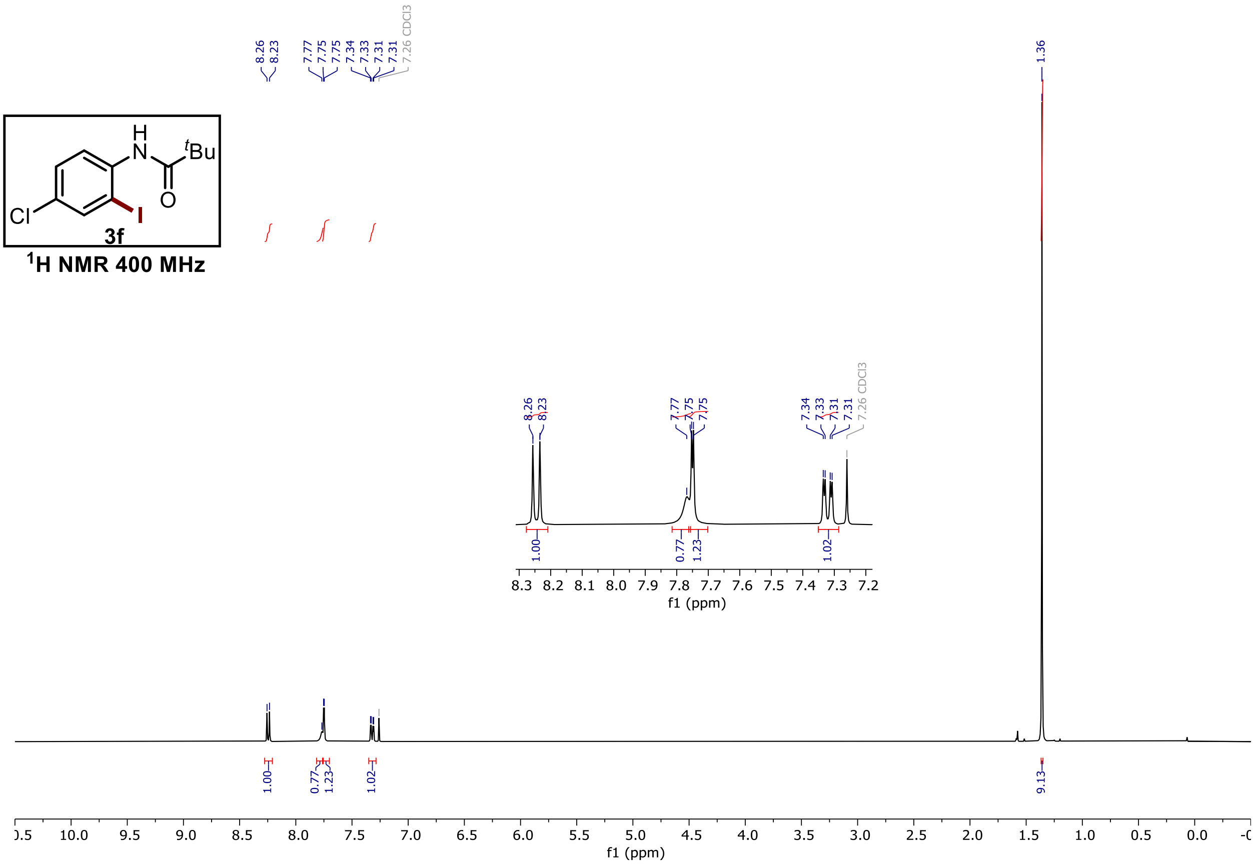
3f
1H NMR 400 MHz

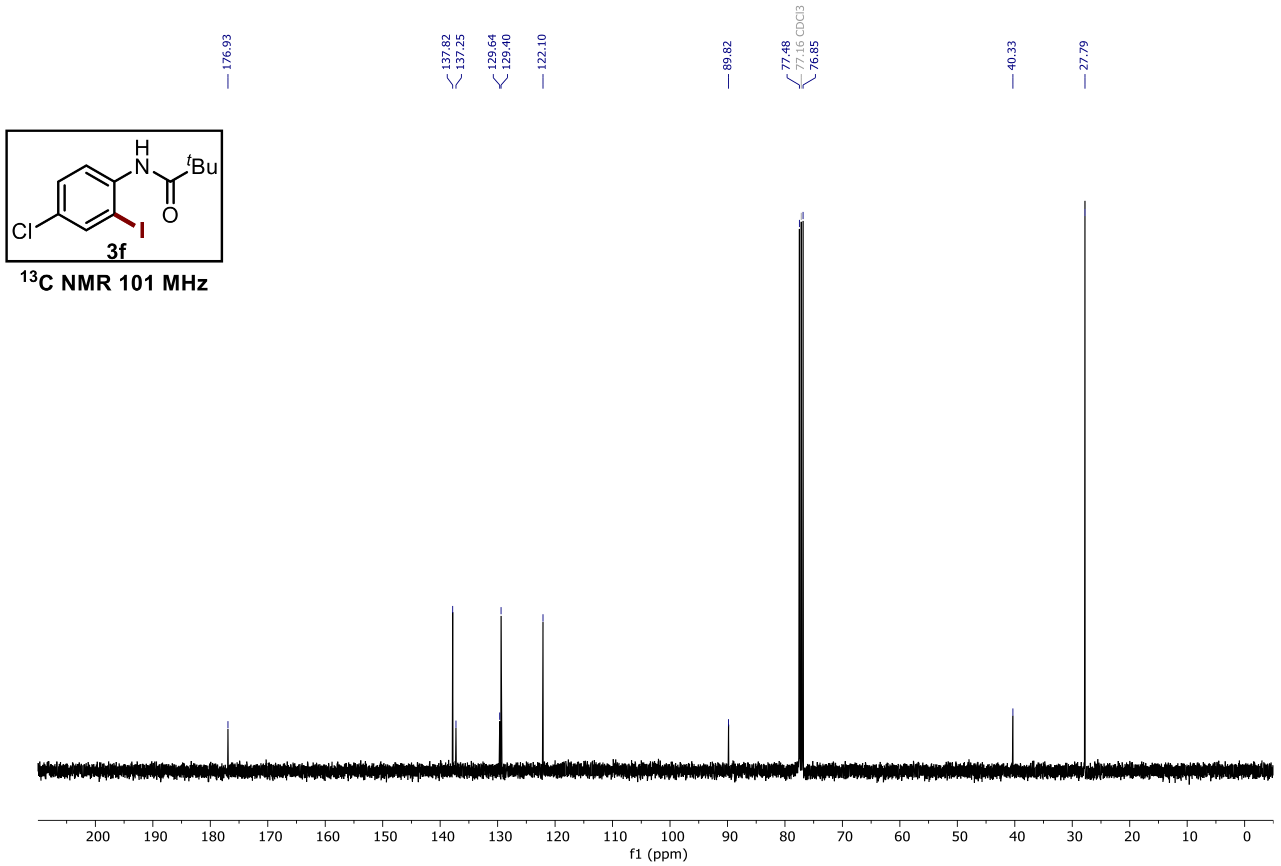
176.93
137.82
137.25
129.64
129.40
122.10
89.82
77.48
77.16 CDCl3
76.85
40.33
27.79
Cl
H
N
tBu
O
I
3f
13C NMR 101 MHz
f1 (ppm)

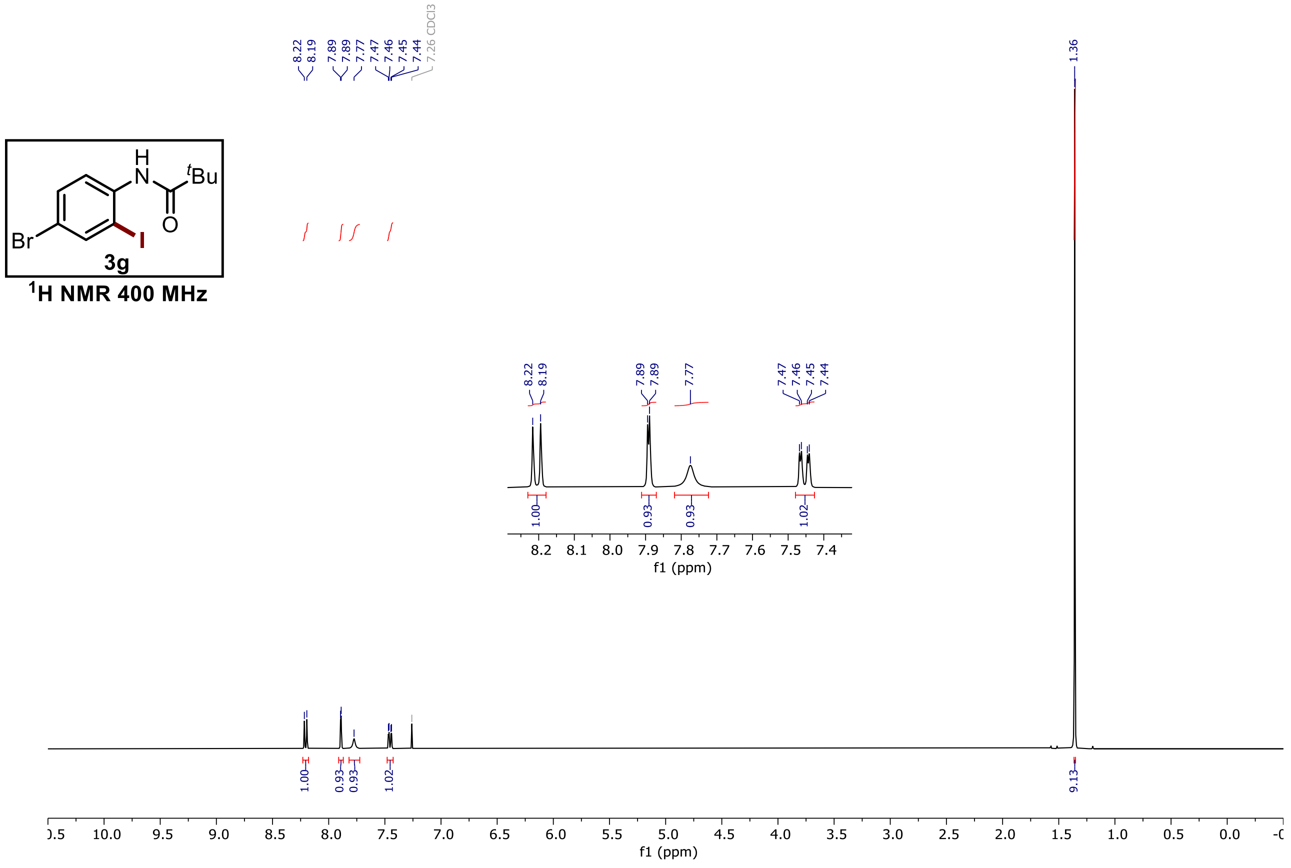
8.22
8.19
7.89
7.89
7.77
7.47
7.46
7.45
7.44
7.26 CDCl3
1.36
Br
H
N
tBu
O
I
3g
1H NMR 400 MHz
1.00
0.93
0.93
1.02
9.13
f1 (ppm)

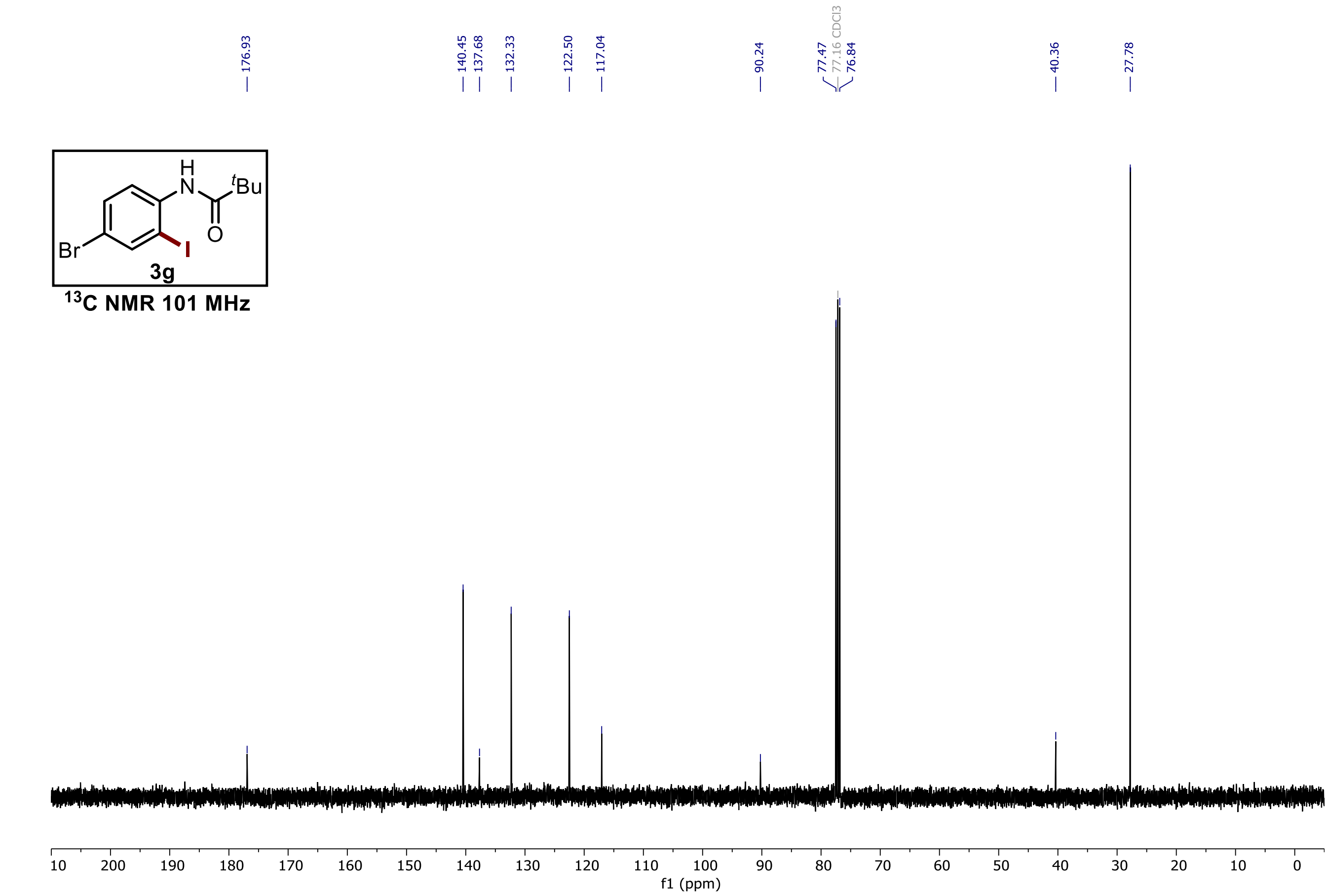
176.93
140.45
137.68
132.33
122.50
117.04
90.24
77.47
77.16 CDCl3
76.84
40.36
27.78
3g
13C NMR 101 MHz
f1 (ppm)

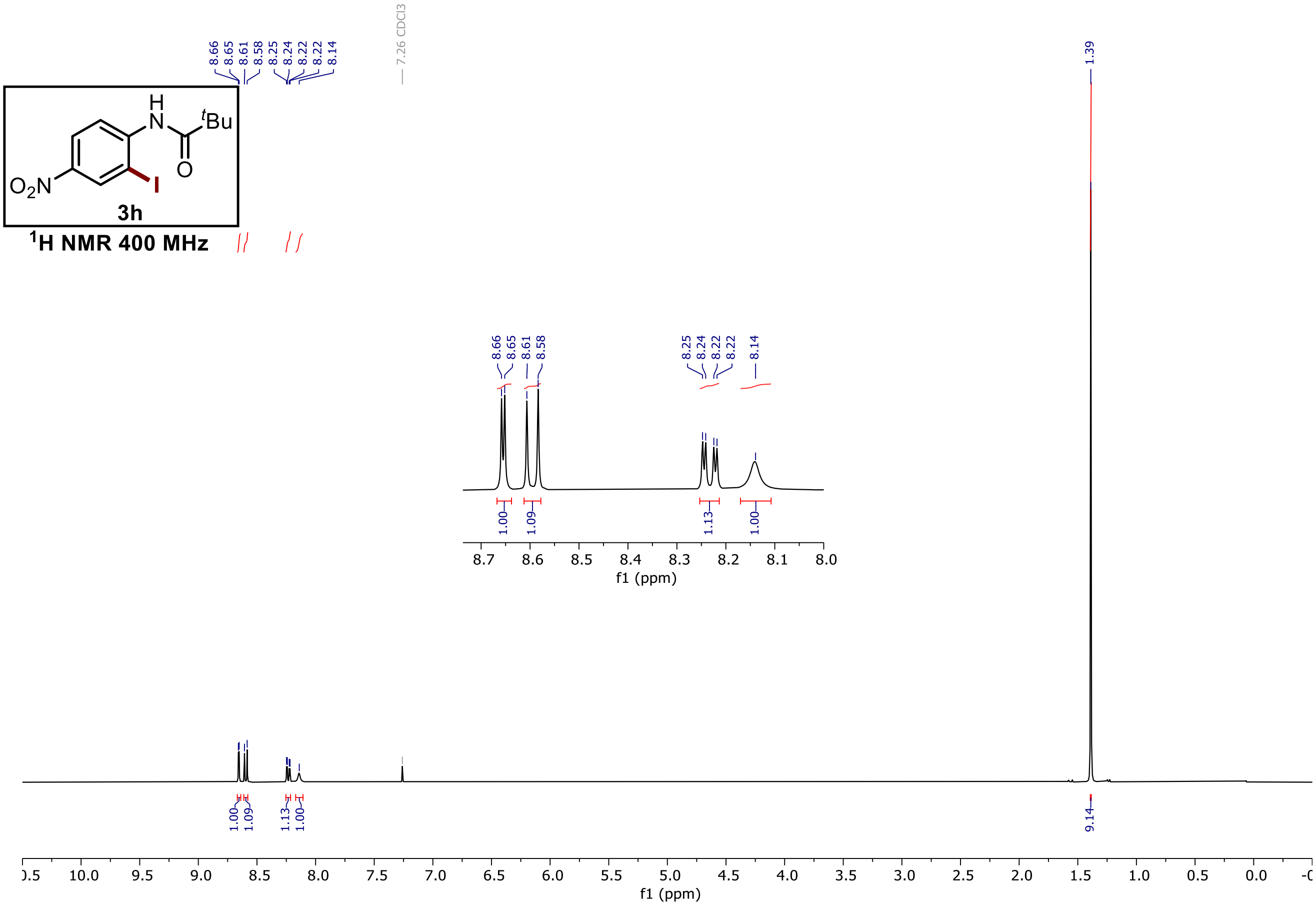
8.66
8.65
8.61
8.58
8.25
8.24
8.22
8.22
8.14
7.26 CDCl3
1.39
3h
1H NMR 400 MHz
1.00
1.09
1.13
1.00
9.14
f1 (ppm)

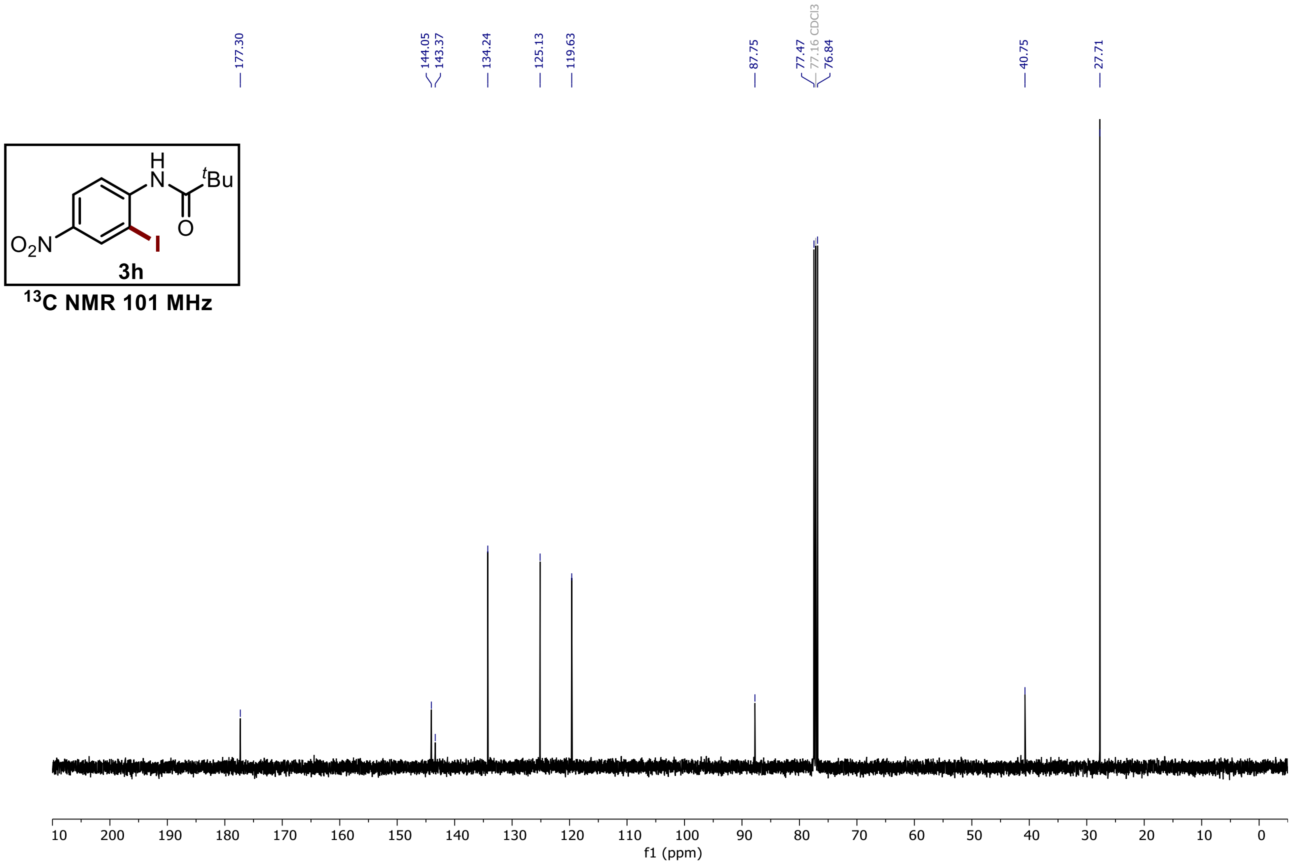

177.30
144.05
143.37
134.24
125.13
119.63
87.75
77.47
77.16 CDCl3
76.84
40.75
27.71
O2N
H
N
tBu
O
I
3h
13C NMR 101 MHz
f1 (ppm)

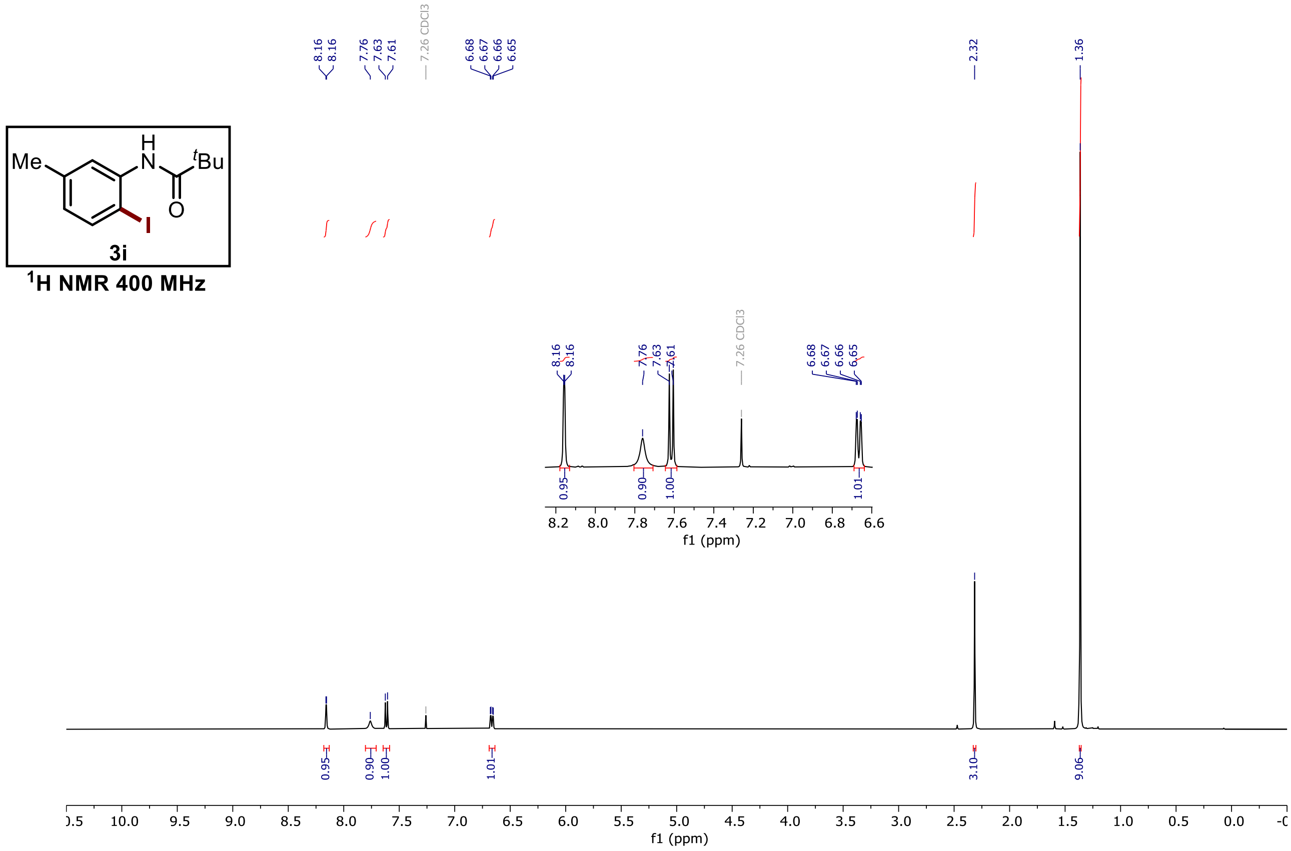

8.16
8.16
7.76
7.63
7.61
7.26 CDCl3
6.68
6.67
6.66
6.65
2.32
1.36
Me
H
N
tBu
O
I
3i
1H NMR 400 MHz
0.95
0.90
1.00
1.01
3.10
9.06
f1 (ppm)

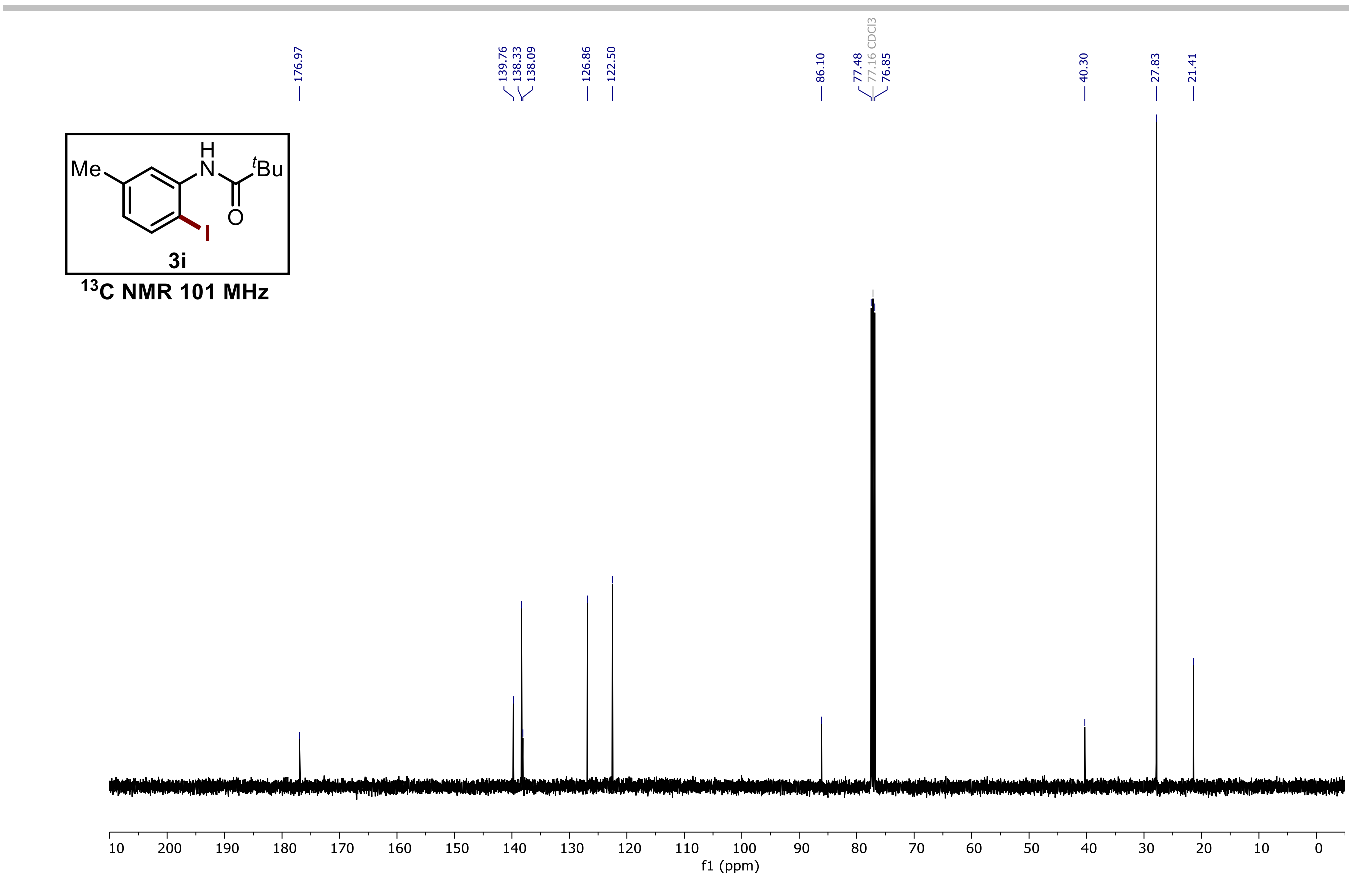

3i
13C NMR 101 MHz
176.97
139.76
138.33
138.09
126.86
122.50
86.10
77.48
77.16 CDCl3
76.85
40.30
27.83
21.41
f1 (ppm)

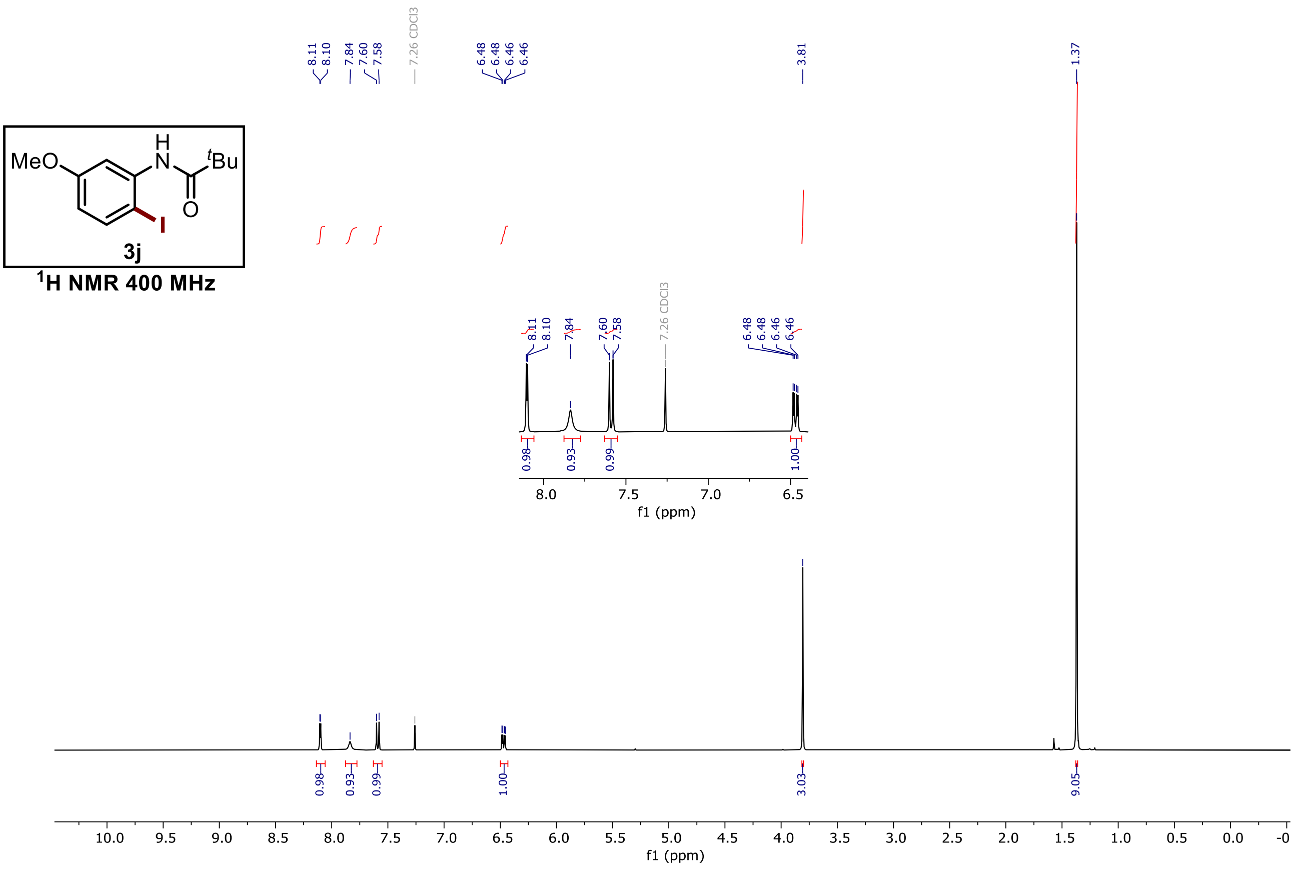

3j
1H NMR 400 MHz
8.11
8.10
7.84
7.60
7.58
7.26 CDCl3
6.48
6.48
6.46
6.46
3.81
1.37
0.98
0.93
0.99
1.00
3.03
9.05
f1 (ppm)

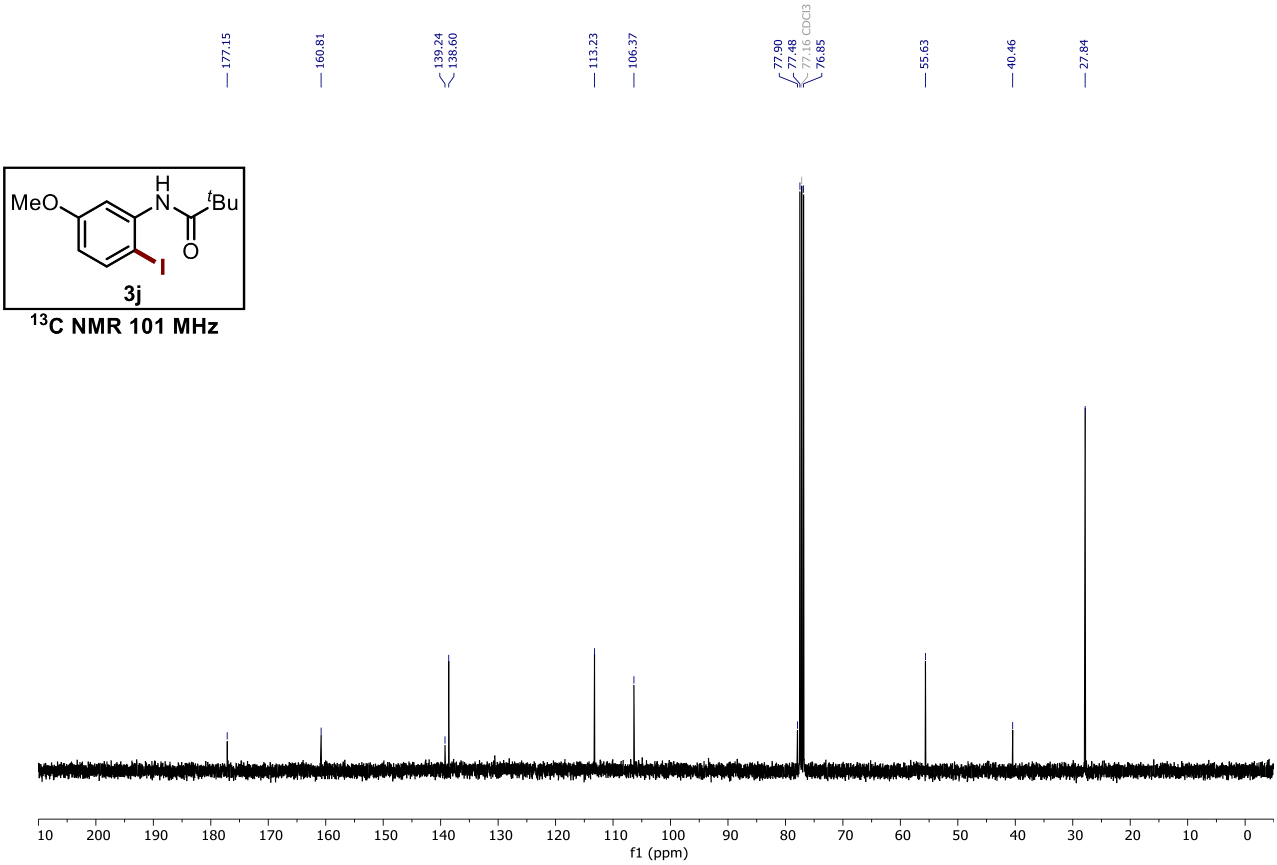

177.15
160.81
139.24
138.60
113.23
106.37
77.90
77.48
77.16 CDCl3
76.85
55.63
40.46
27.84
MeO
H
N
tBu
O
I
3j
13C NMR 101 MHz
f1 (ppm)

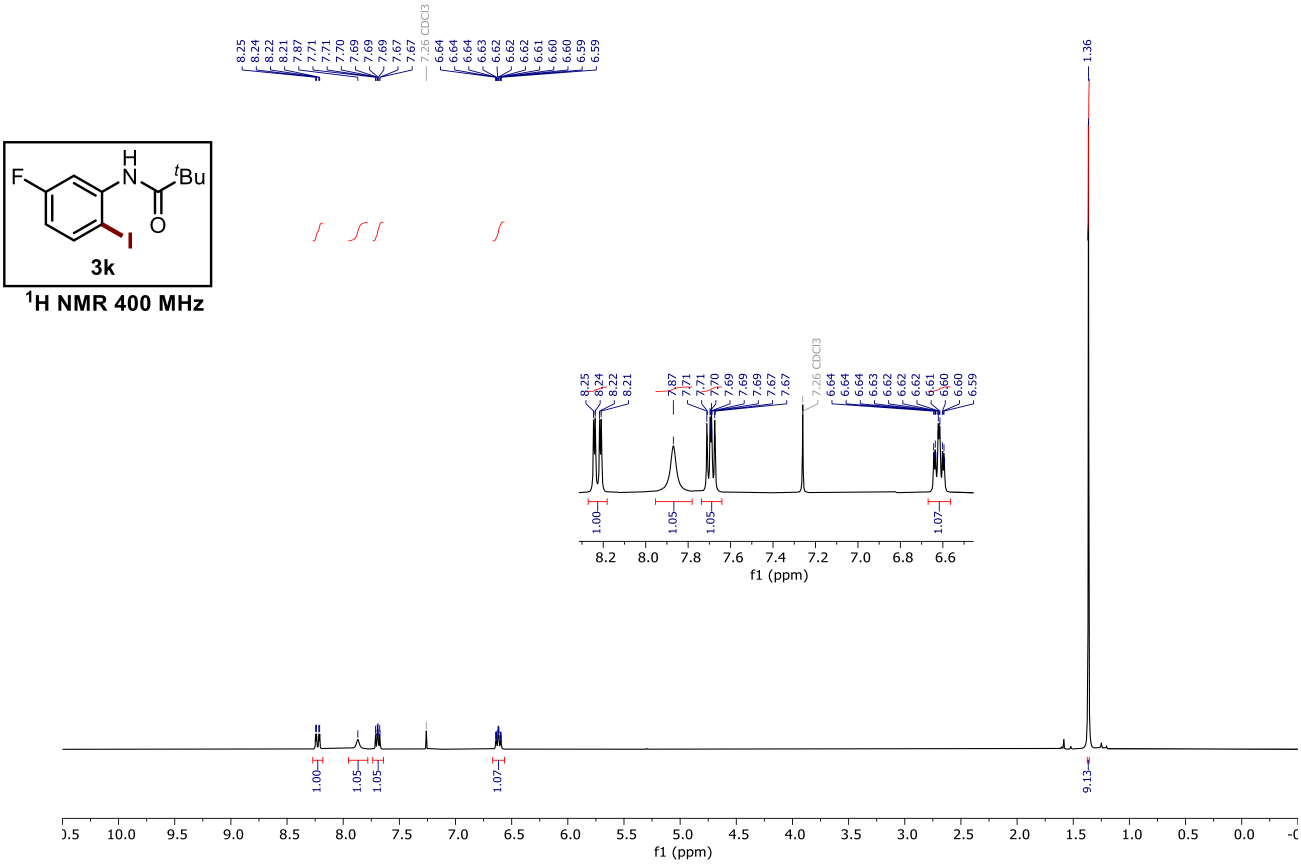

8.25
8.24
8.22
8.21
7.87
7.71
7.71
7.70
7.69
7.69
7.69
7.67
7.67
7.26 CDCl3
6.64
6.64
6.64
6.63
6.62
6.62
6.62
6.61
6.60
6.60
6.59
6.59
1.36
F
H
N
tBu
O
I
3k
1H NMR 400 MHz
1.00
1.05
1.05
1.07
9.13
f1 (ppm)

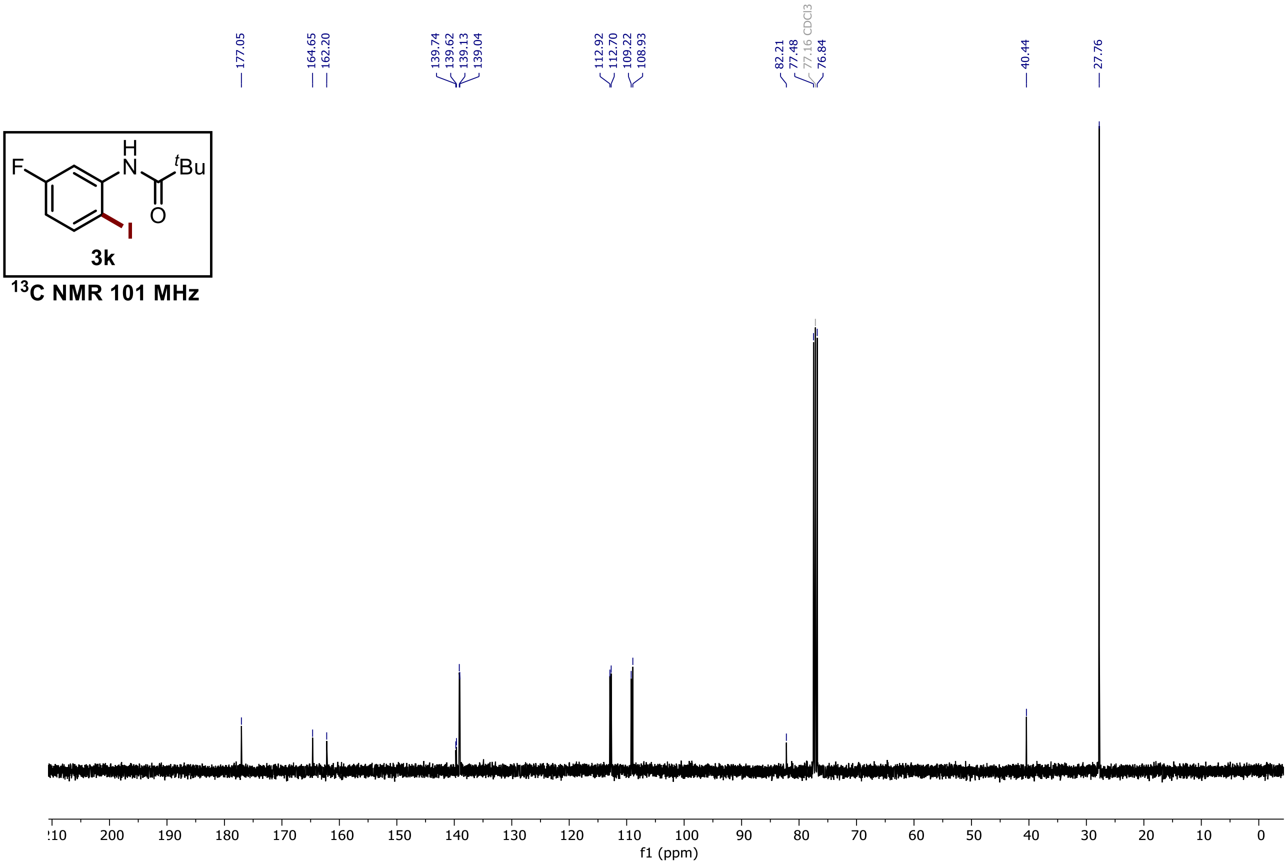
177.05
164.65
162.20
139.74
139.62
139.13
139.04
112.92
112.70
109.22
108.93
82.21
77.48
77.16 CDCl3
76.84
40.44
27.76
F
H
N
tBu
O
I
3k
13C NMR 101 MHz
f1 (ppm)

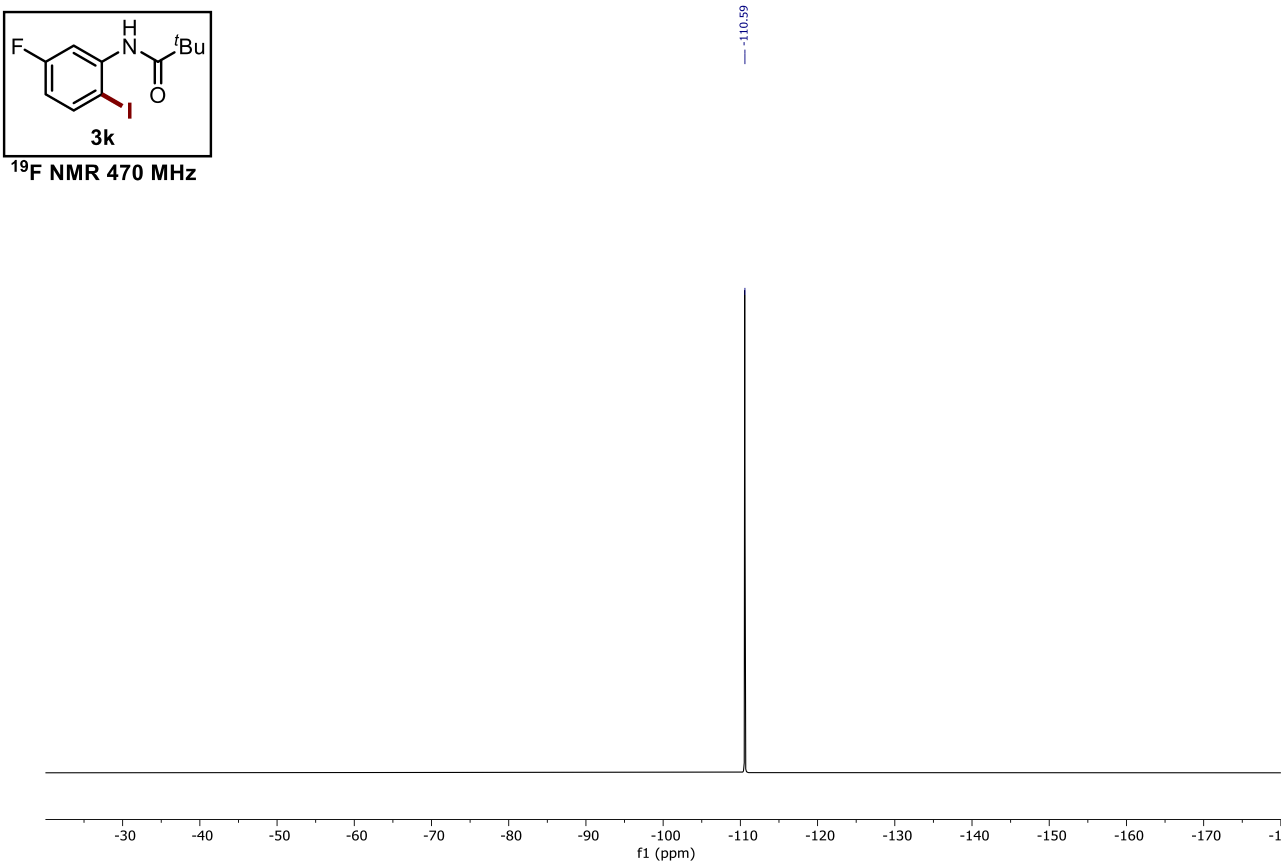
-110.59
F
H
N
tBu
O
I
3k
19F NMR 470 MHz
f1 (ppm)

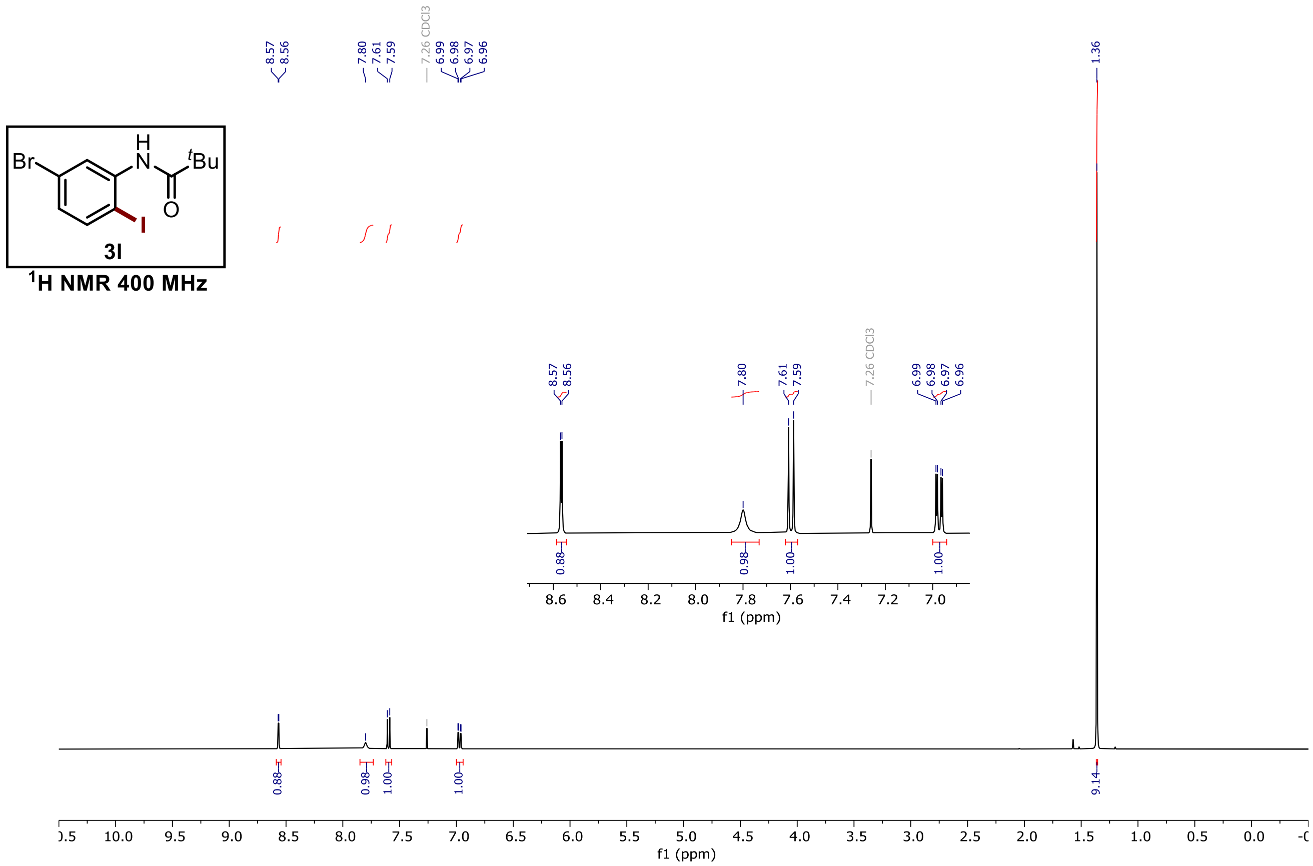

Br
H
N
tBu
O
I
3l
1H NMR 400 MHz
8.57
8.56
7.80
7.61
7.59
7.26 CDCl3
6.99
6.98
6.97
6.96
1.36
0.88
0.98
1.00
1.00
9.14
f1 (ppm)

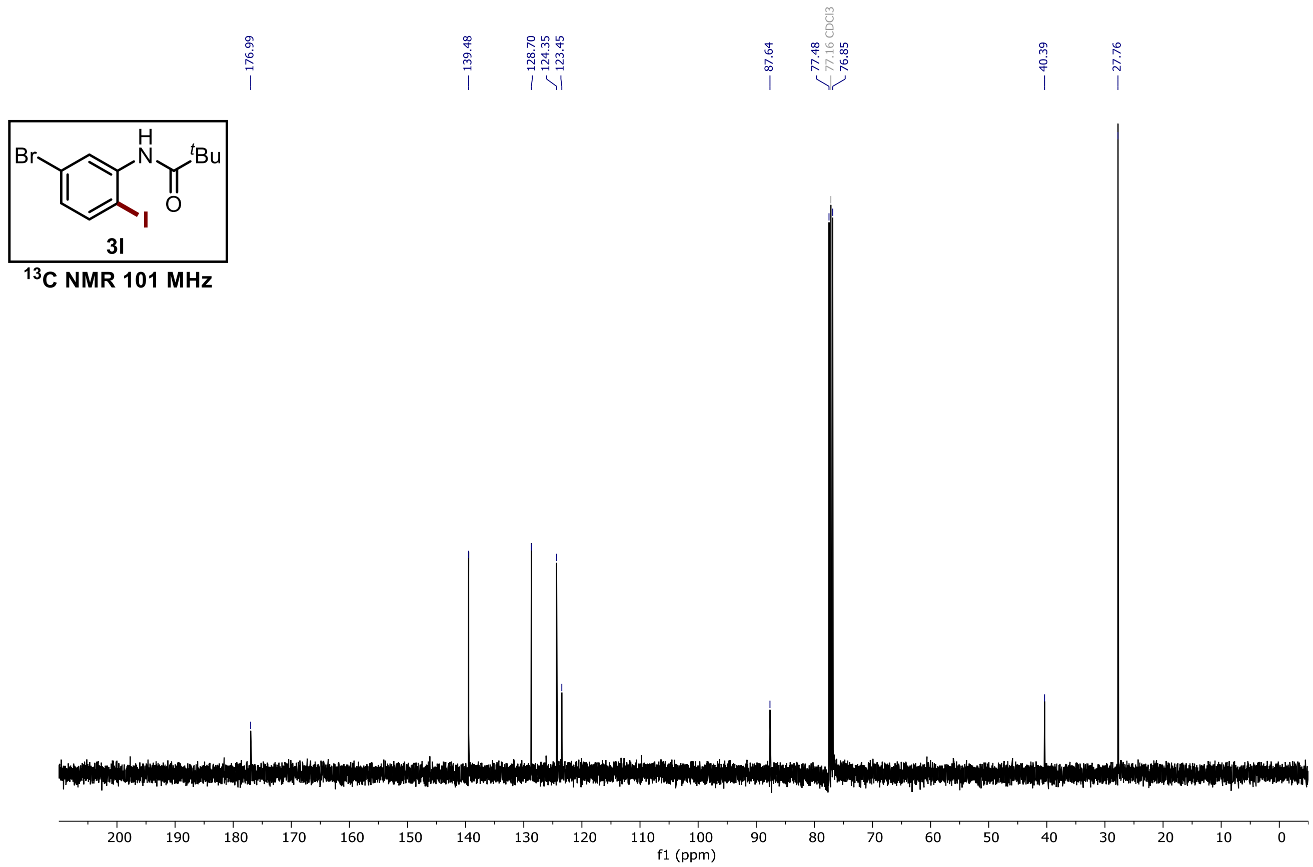

Br
H
N
tBu
O
I
3l
13C NMR 101 MHz
176.99
139.48
128.70
124.35
123.45
87.64
77.48
77.16 CDCl3
76.85
40.39
27.76
f1 (ppm)

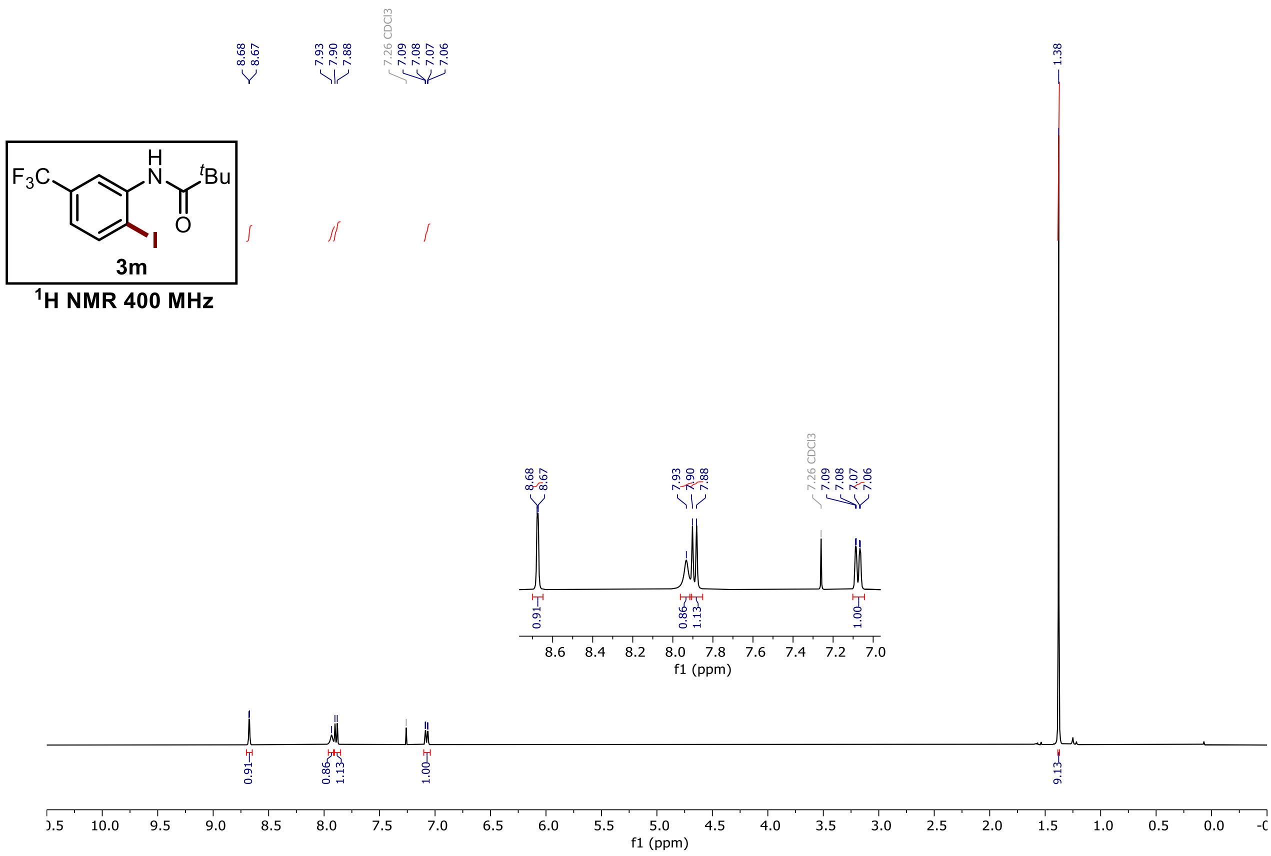
F3C
H
N
tBu
O
I
3m
1H NMR 400 MHz
8.68
8.67
7.93
7.90
7.88
7.26 CDCl3
7.09
7.08
7.07
7.06
1.38
0.91
0.86
1.13
1.00
9.13
f1 (ppm)

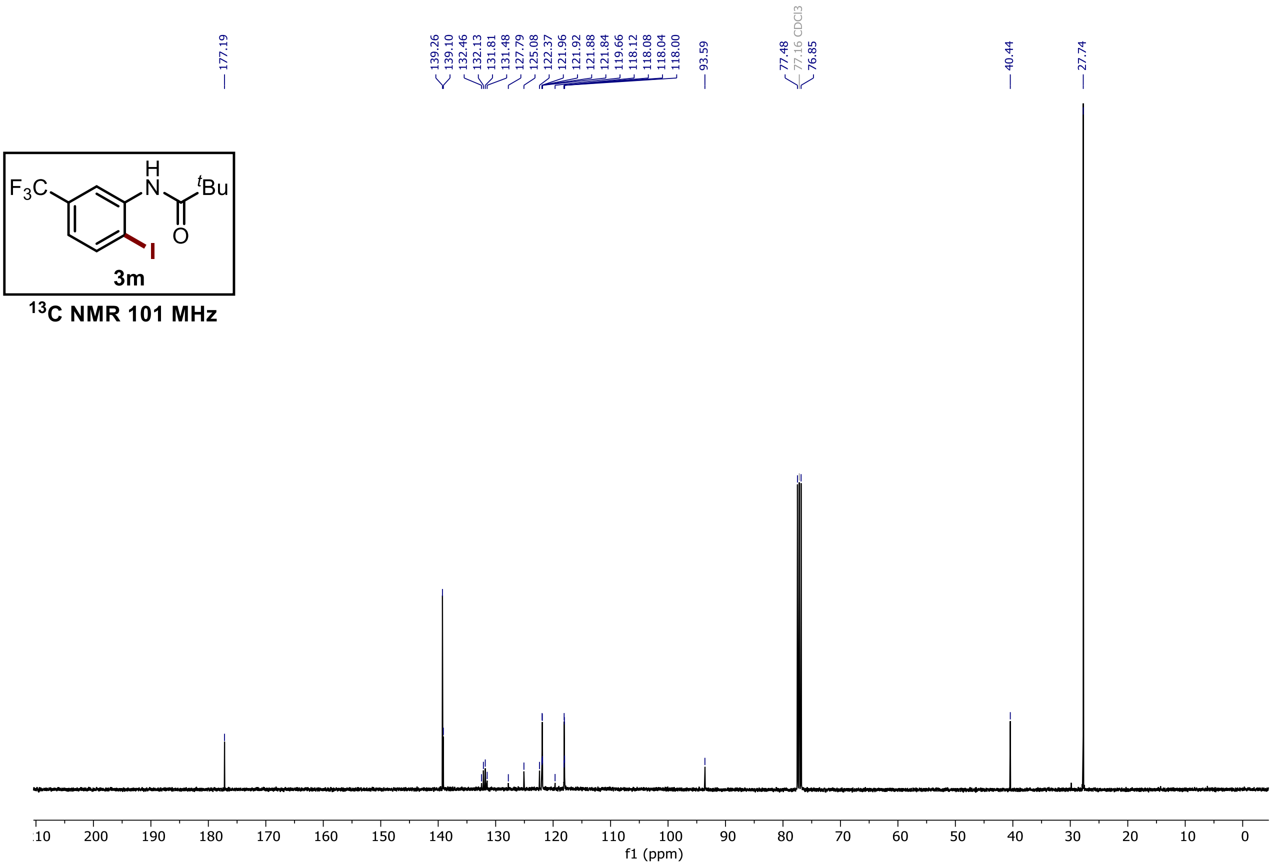
F3C
H
N
tBu
O
I
3m
13C NMR 101 MHz
177.19
139.26
139.10
132.46
132.13
131.81
131.48
127.79
125.08
122.37
121.96
121.92
121.88
121.84
119.66
118.12
118.08
118.04
118.00
93.59
77.48
77.16 CDCl3
76.85
40.44
27.74
f1 (ppm)

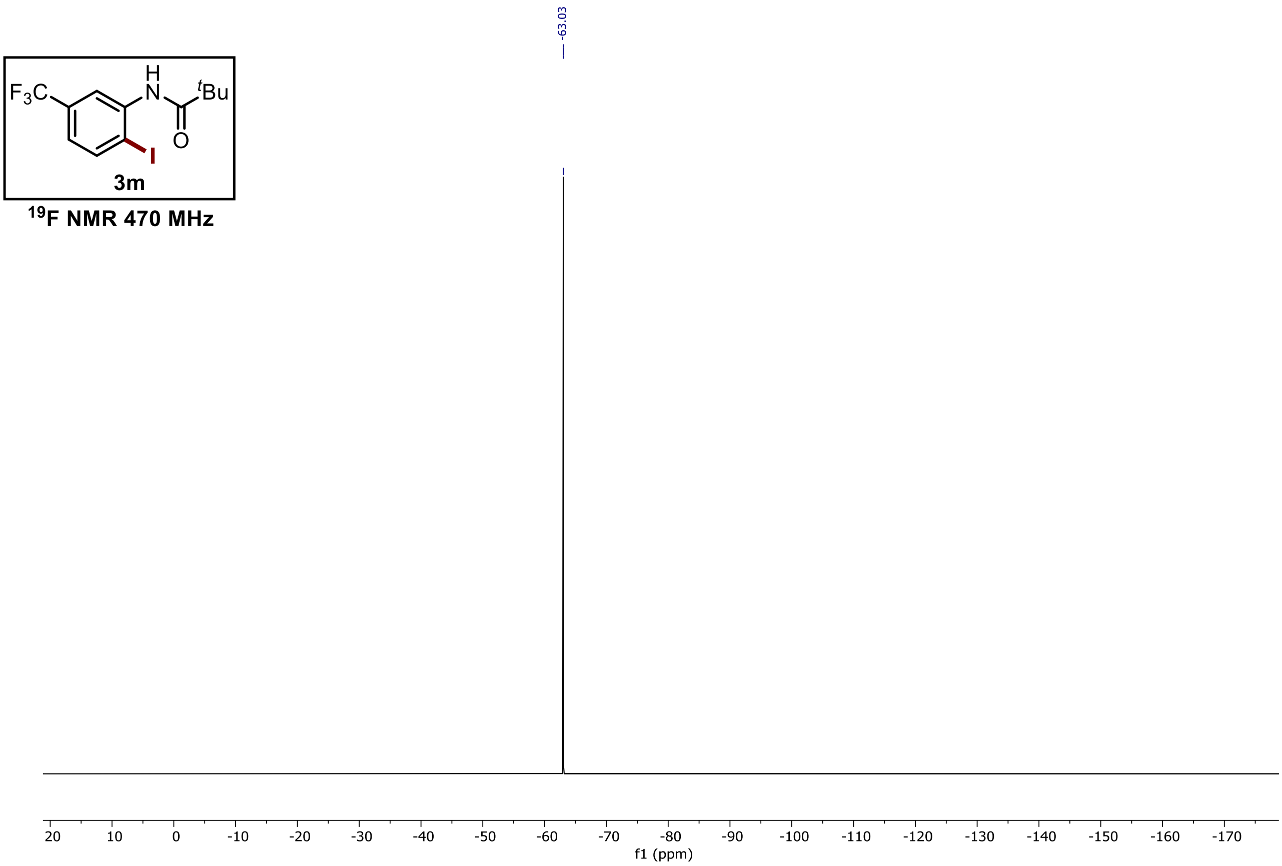
F3C
H
N
tBu
O
I
3m
19F NMR 470 MHz
-63.03
f1 (ppm)

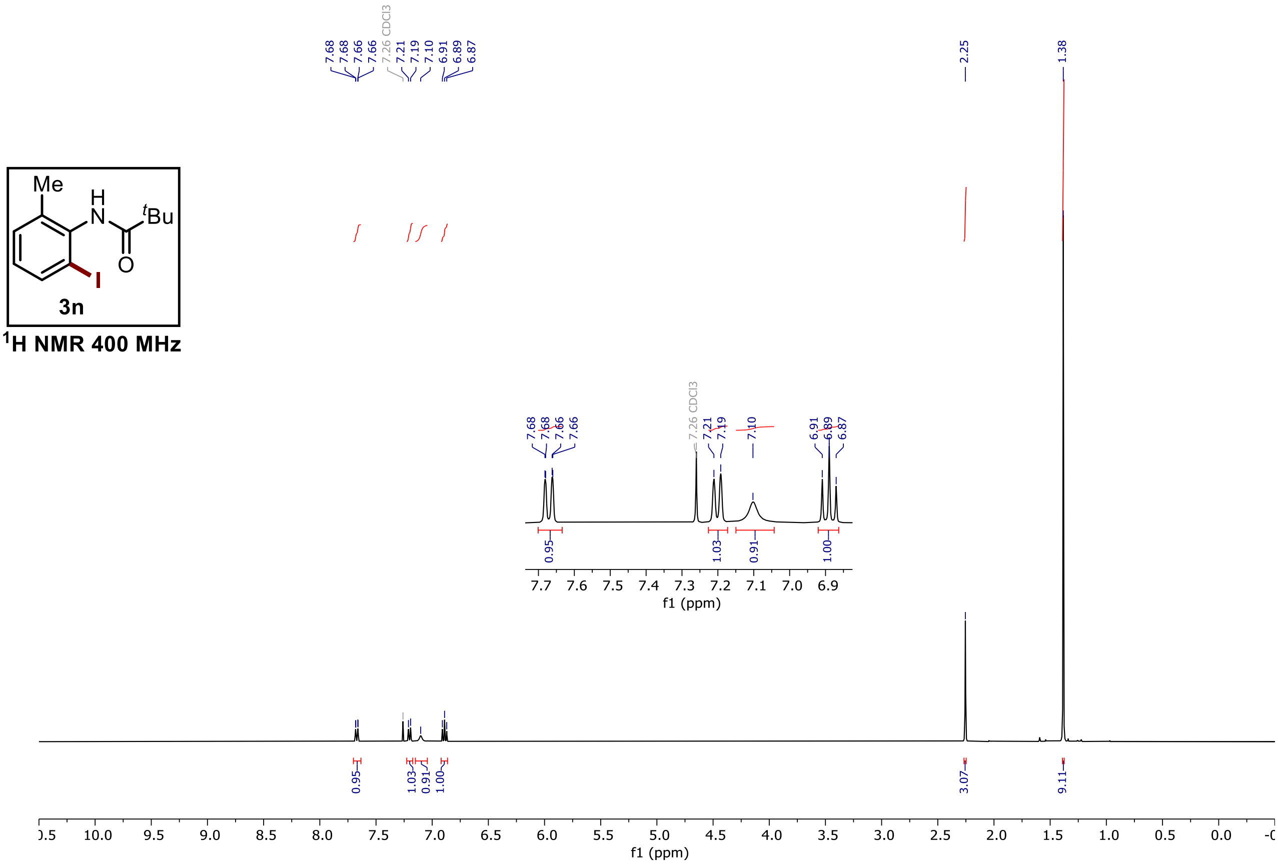
Me
H
N
tBu
O
I
3n
1H NMR 400 MHz
7.68
7.68
7.66
7.66
7.26 CDCl3
7.21
7.19
7.10
6.91
6.89
6.87
2.25
1.38
0.95
1.03
0.91
1.00
3.07
9.11
f1 (ppm)

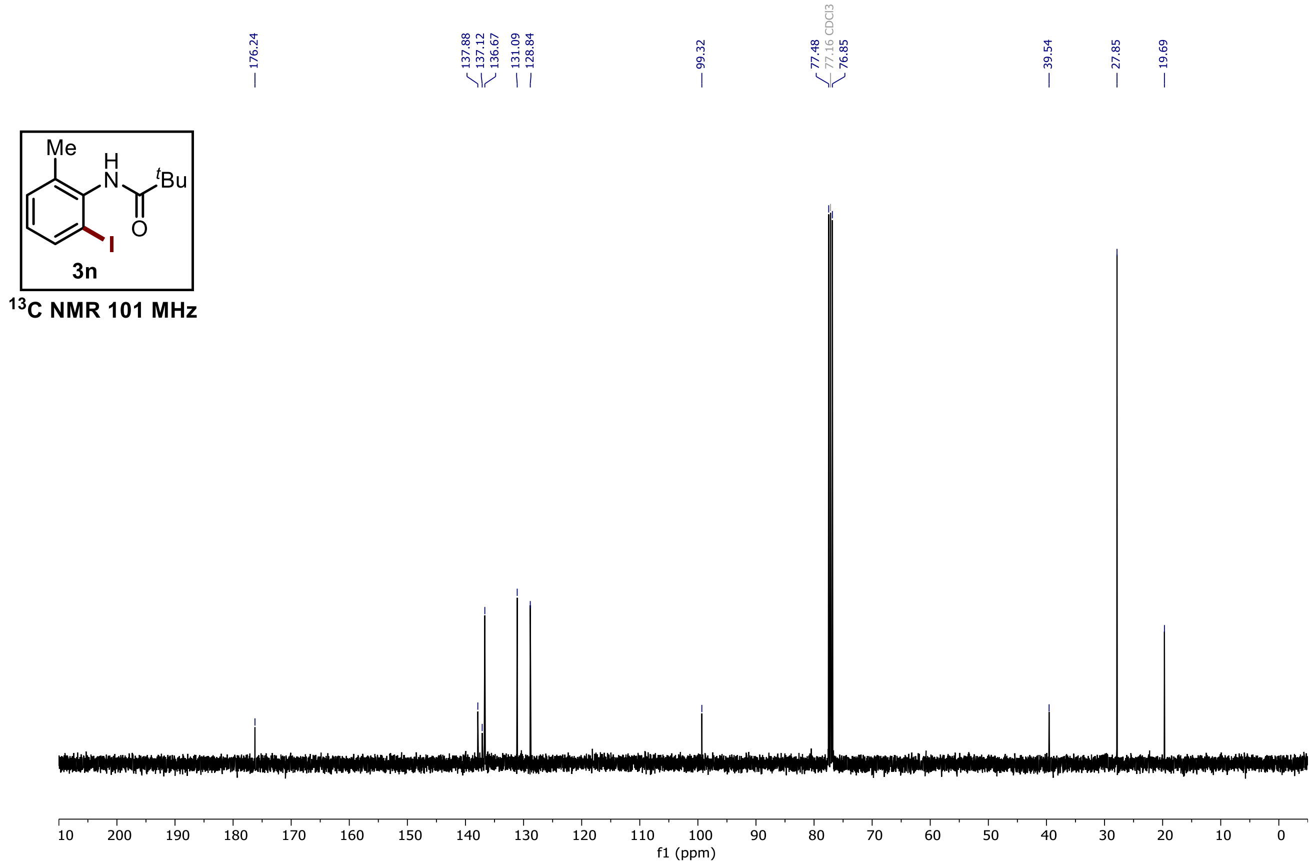

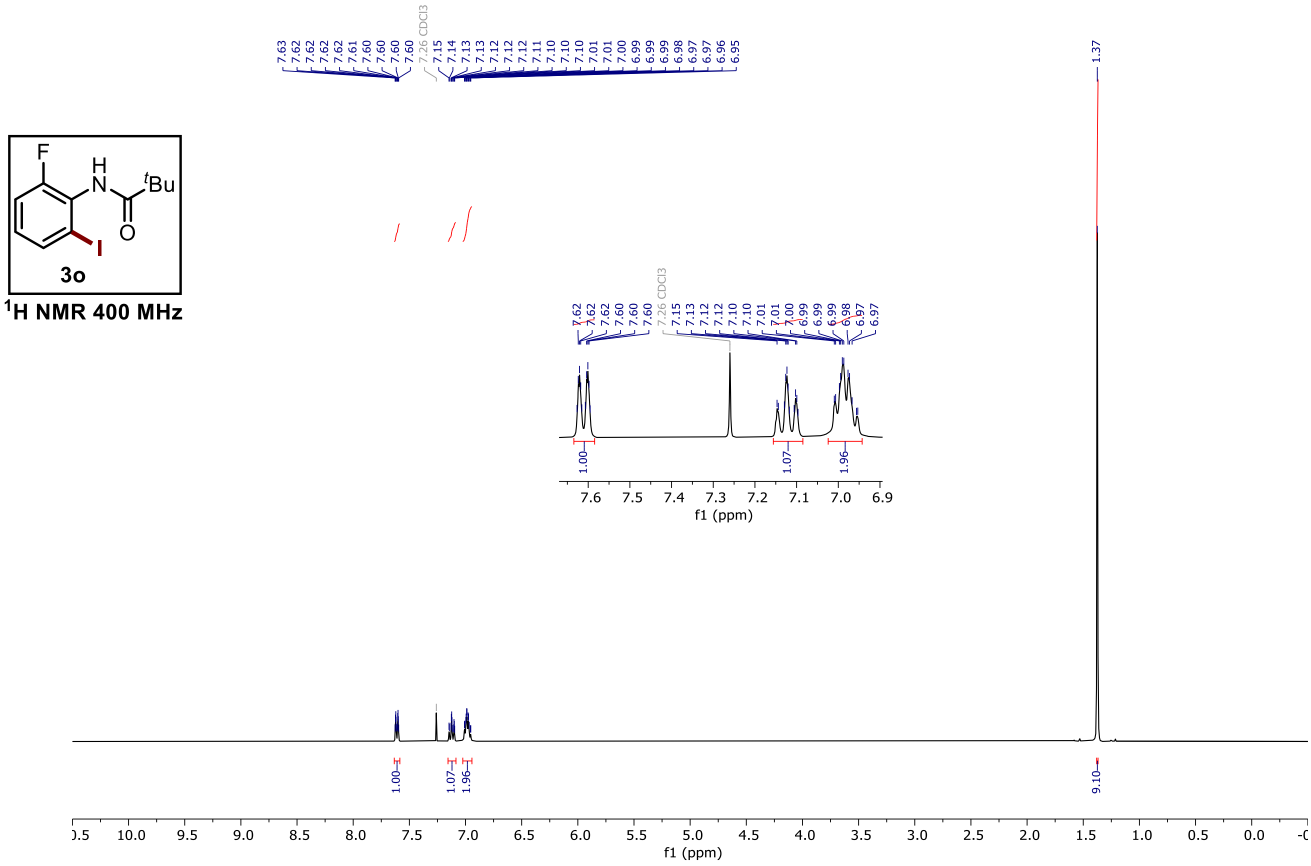

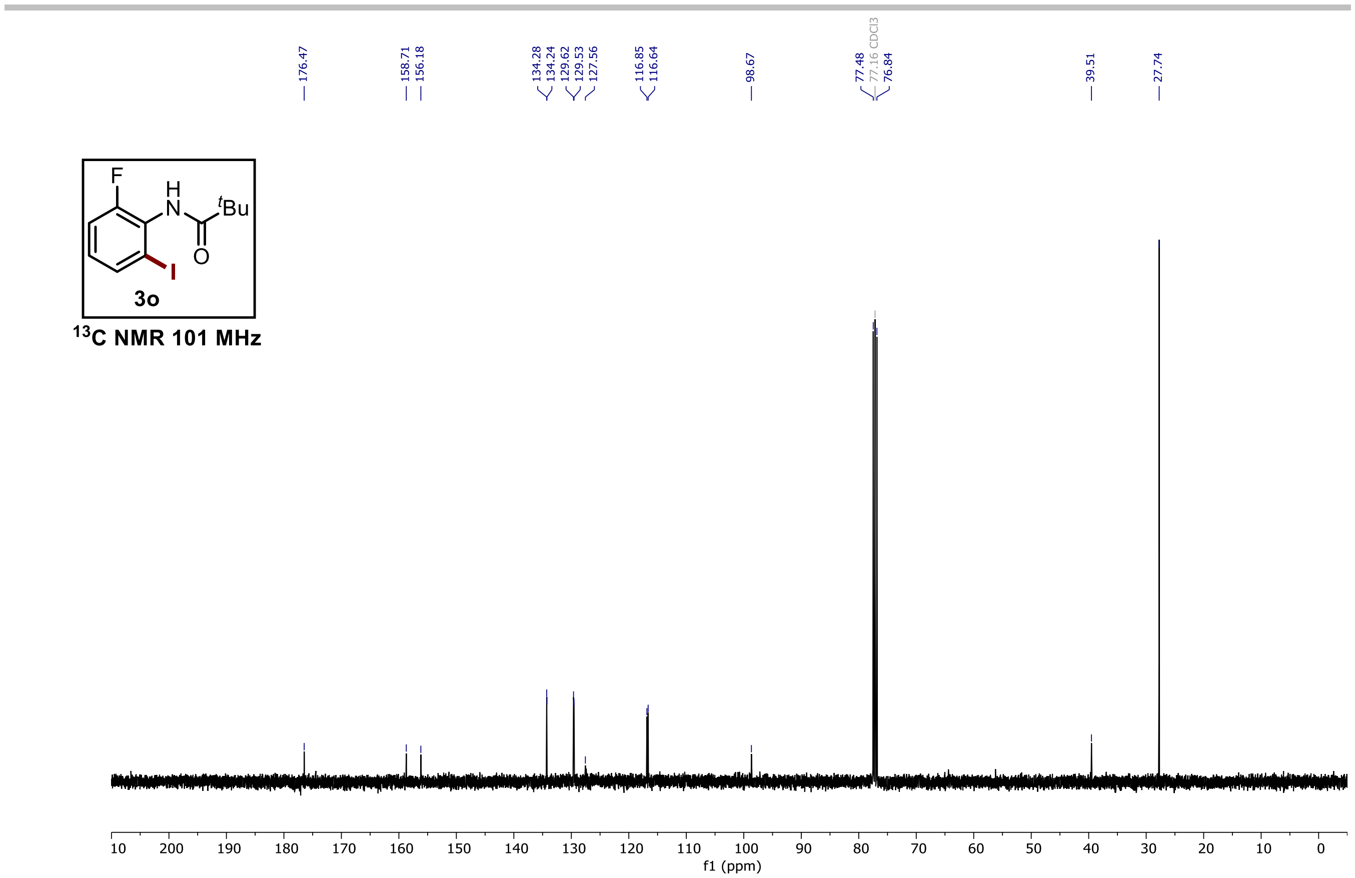
176.47
158.71
156.18
134.28
134.24
129.62
129.53
127.56
116.85
116.64
98.67
77.48
77.16 CDCl3
76.84
39.51
27.74
F
H
N
tBu
O
I
3o
13C NMR 101 MHz
f1 (ppm)

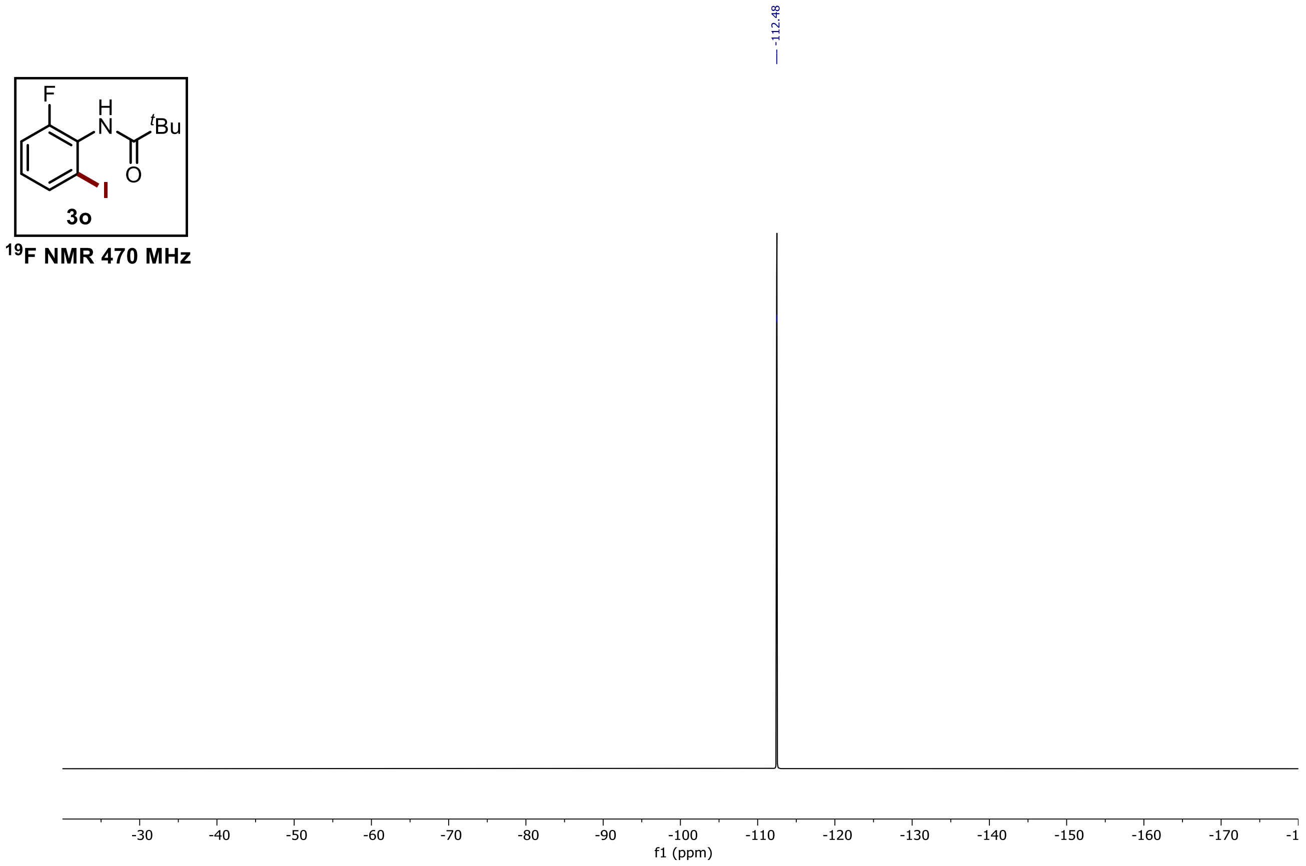
-112.48
F
H
N
tBu
O
I
3o
19F NMR 470 MHz
f1 (ppm)

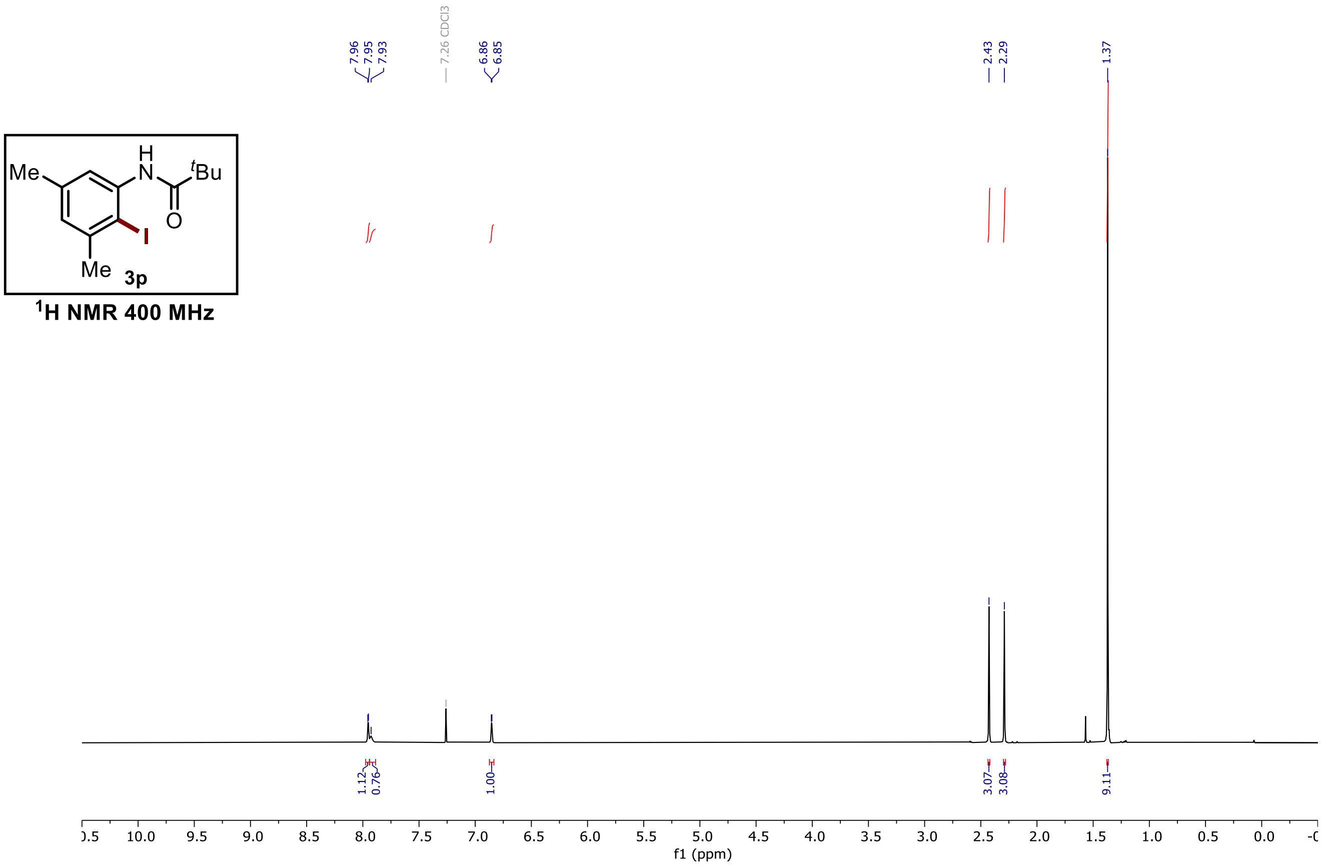
Me
H
N
tBu
O
I
Me 3p
1H NMR 400 MHz
7.96
7.95
7.93
7.26 CDCl3
6.86
6.85
2.43
2.29
1.37
1.12
0.76
1.00
3.07
3.08
9.11
f1 (ppm)

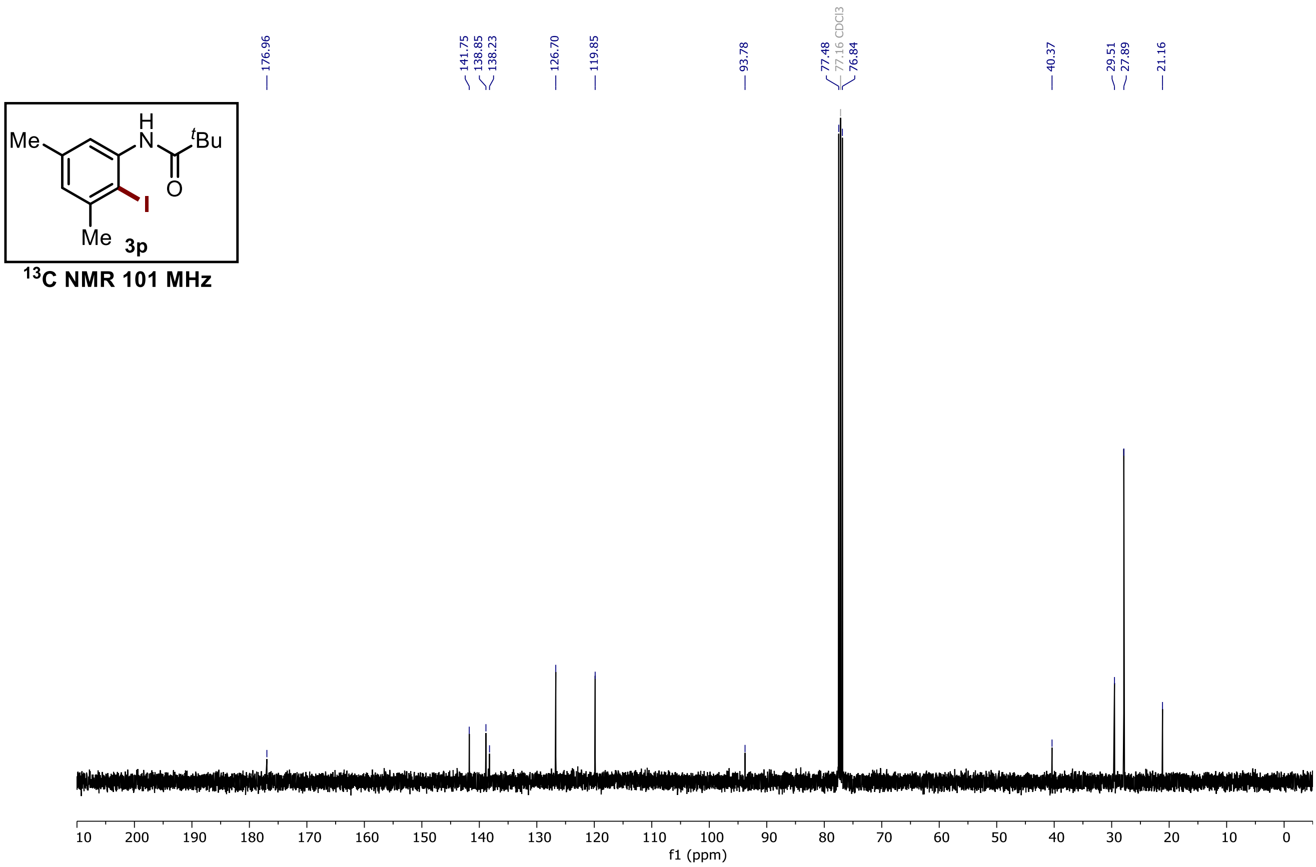
Me
H
N
tBu
O
I
Me 3p
13C NMR 101 MHz
176.96
141.75
138.85
138.23
126.70
119.85
93.78
77.48
77.16 CDCl3
76.84
40.37
29.51
27.89
21.16
f1 (ppm)

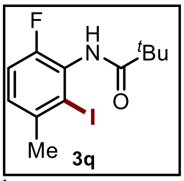

^{1}H NMR 400 MHz

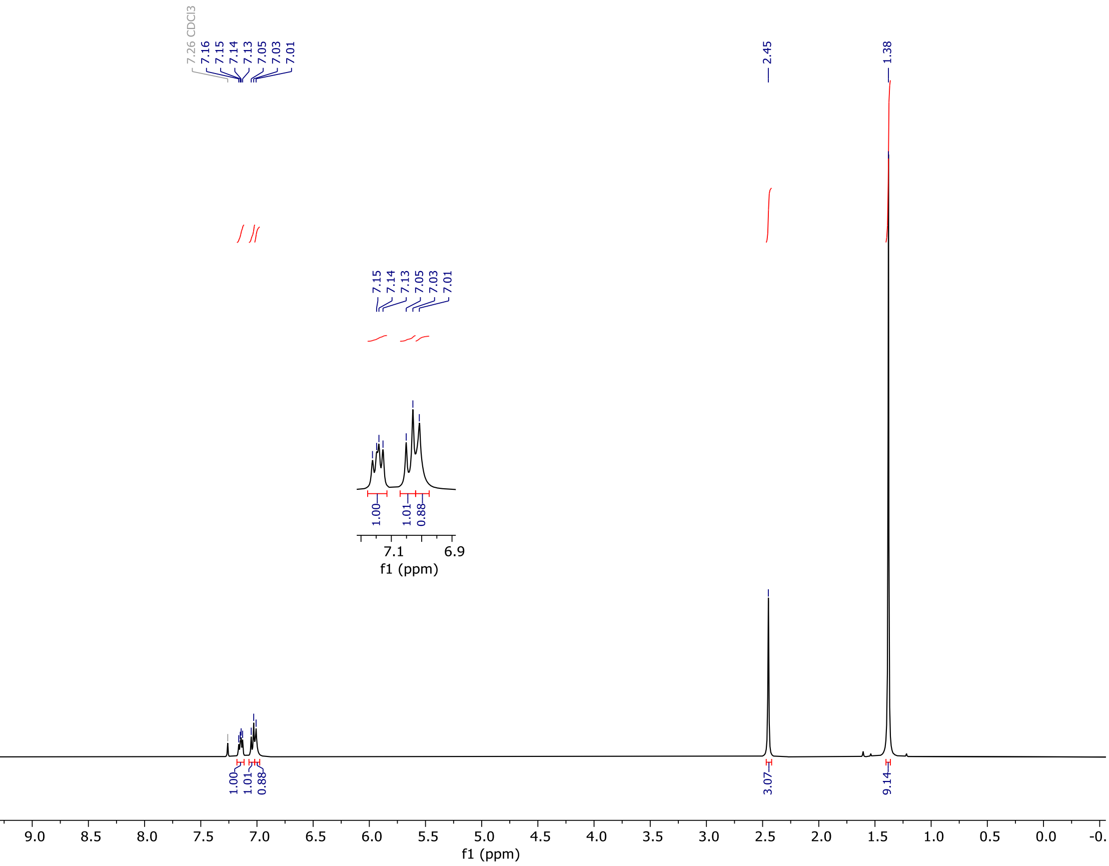

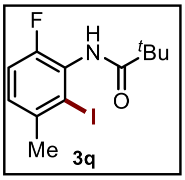

^{13}C NMR 101 MHz

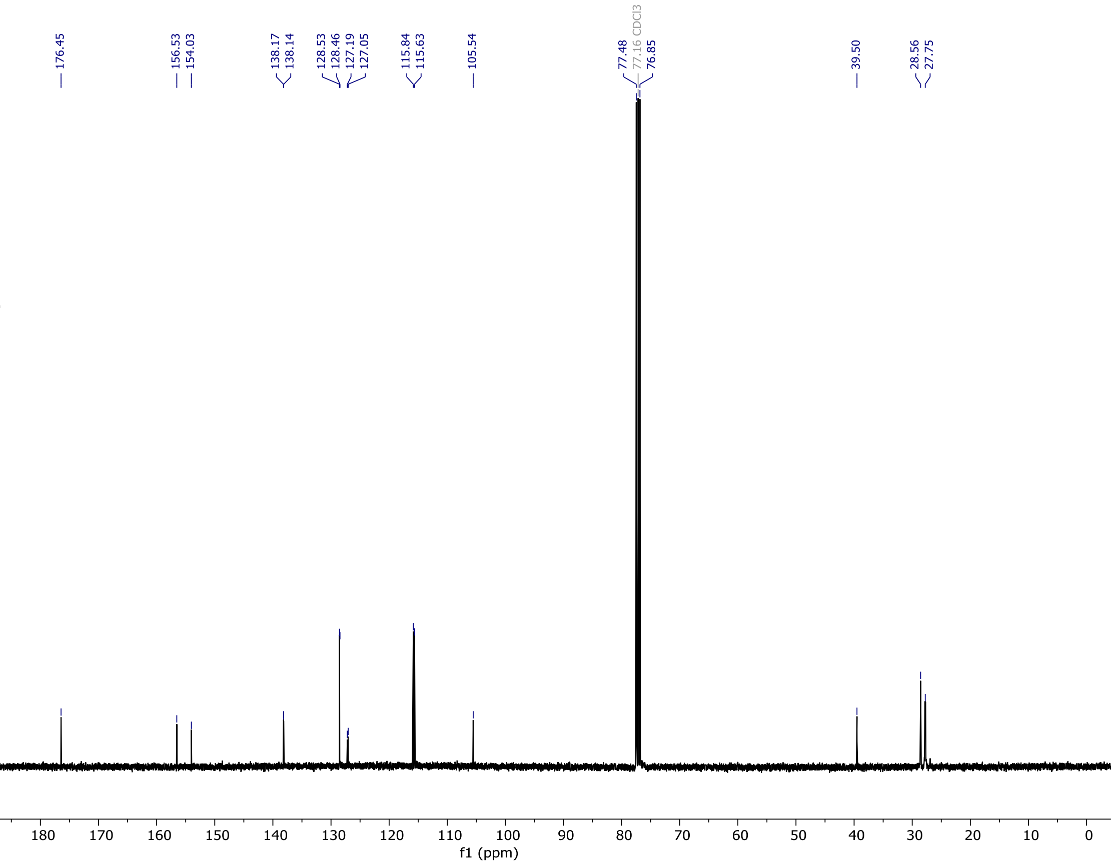

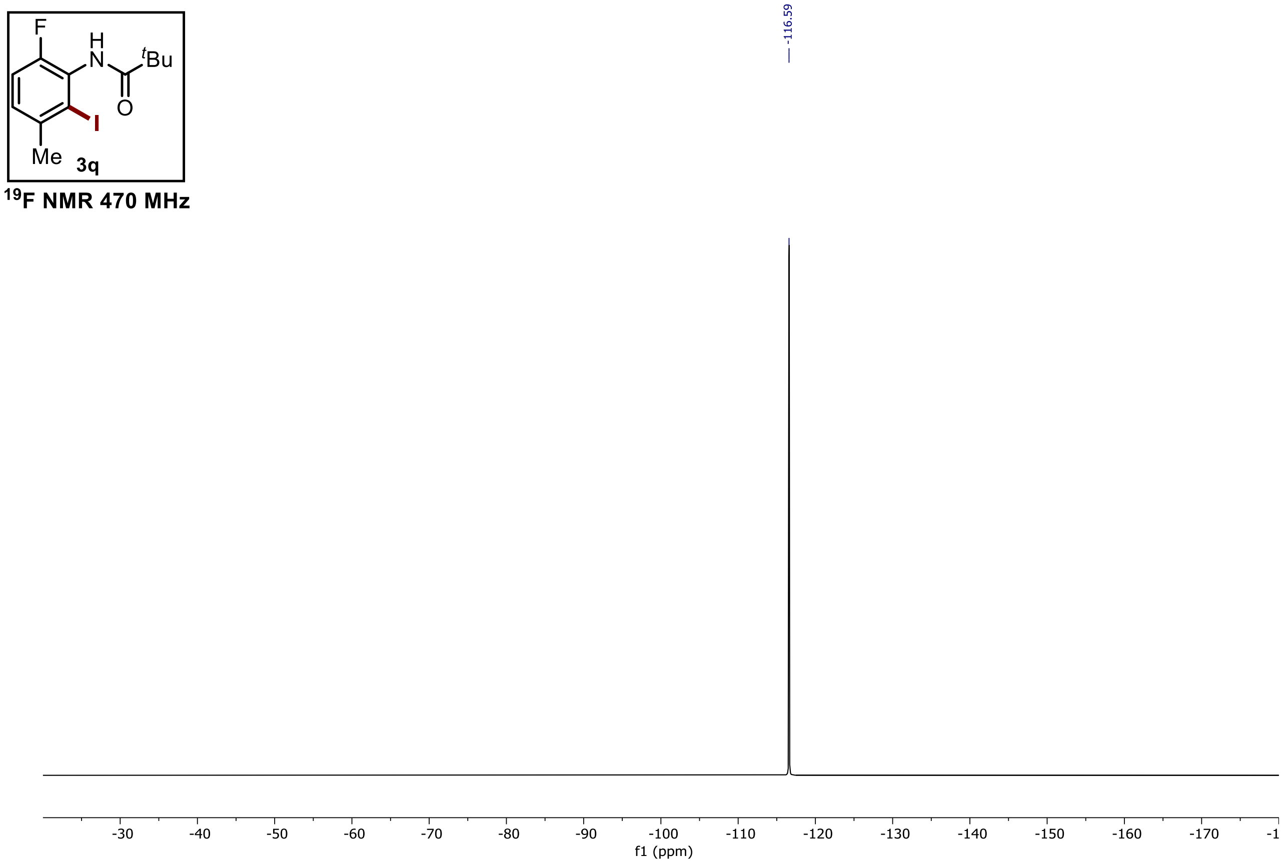
F
H
N
tBu
O
I
Me
3q
19F NMR 470 MHz
-116.59
-30
-40
-50
-60
-70
-80
-90
-100
-110
-120
-130
-140
-150
-160
-170
f1 (ppm)

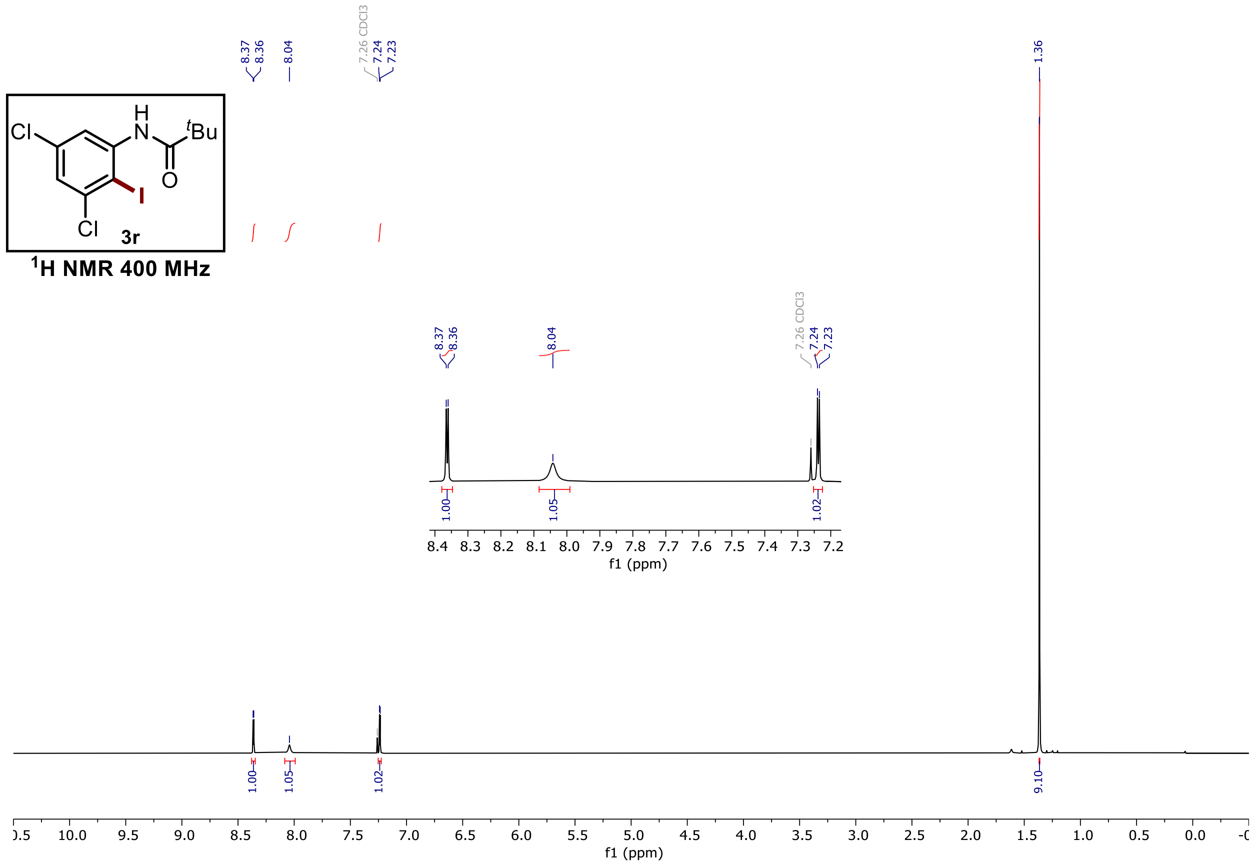
Cl
H
N
tBu
O
I
Cl
3r
1H NMR 400 MHz
8.37
8.36
8.04
7.26 CDCl3
7.24
7.23
1.36
1.00
1.05
1.02
9.10
8.4 8.3 8.2 8.1 8.0 7.9 7.8 7.7 7.6 7.5 7.4 7.3 7.2
f1 (ppm)
10.0 9.5 9.0 8.5 8.0 7.5 7.0 6.5 6.0 5.5 5.0 4.5 4.0 3.5 3.0 2.5 2.0 1.5 1.0 0.5 0.0

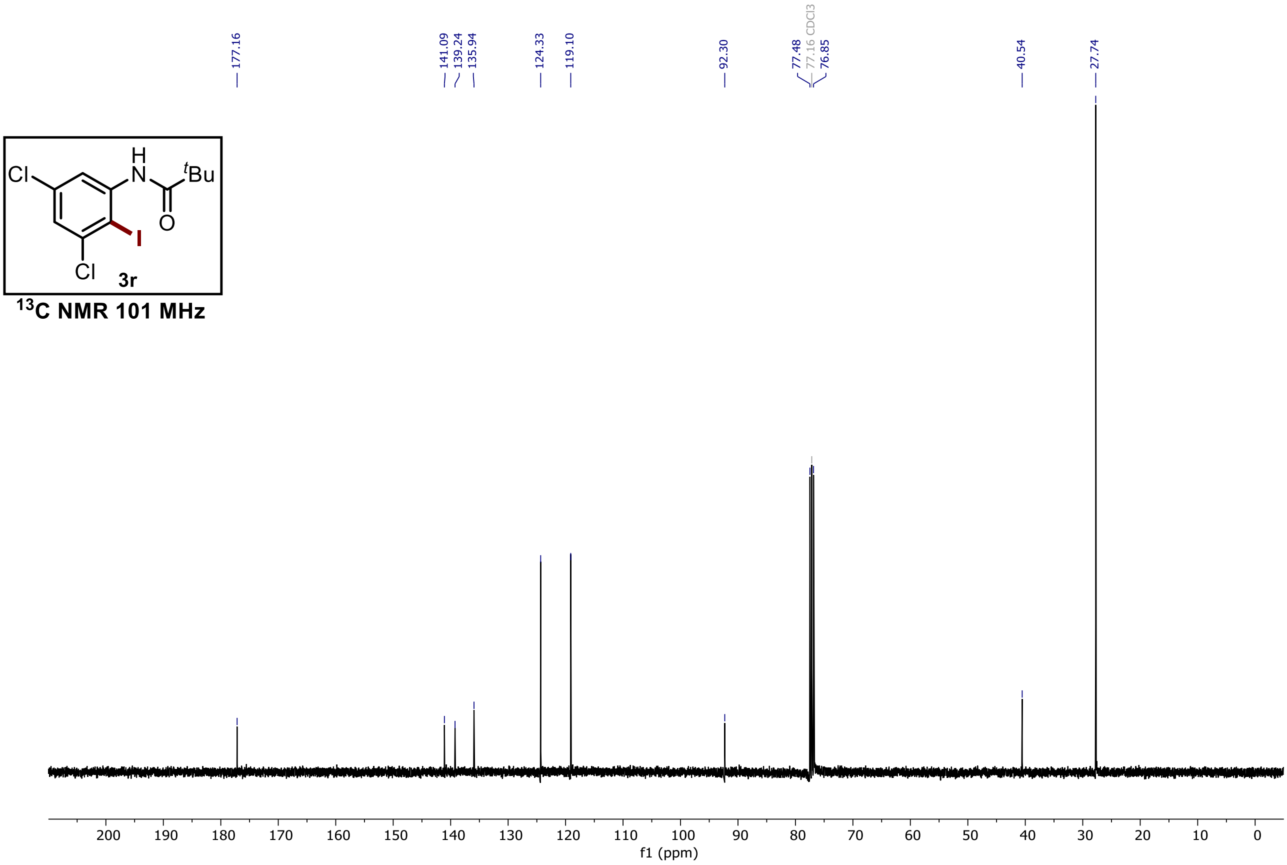
177.16
141.09
139.24
135.94
124.33
119.10
92.30
77.48
77.16 CDCl3
76.85
40.54
27.74
Cl
H
N
tBu
O
I
Cl
3r
13C NMR 101 MHz
f1 (ppm)

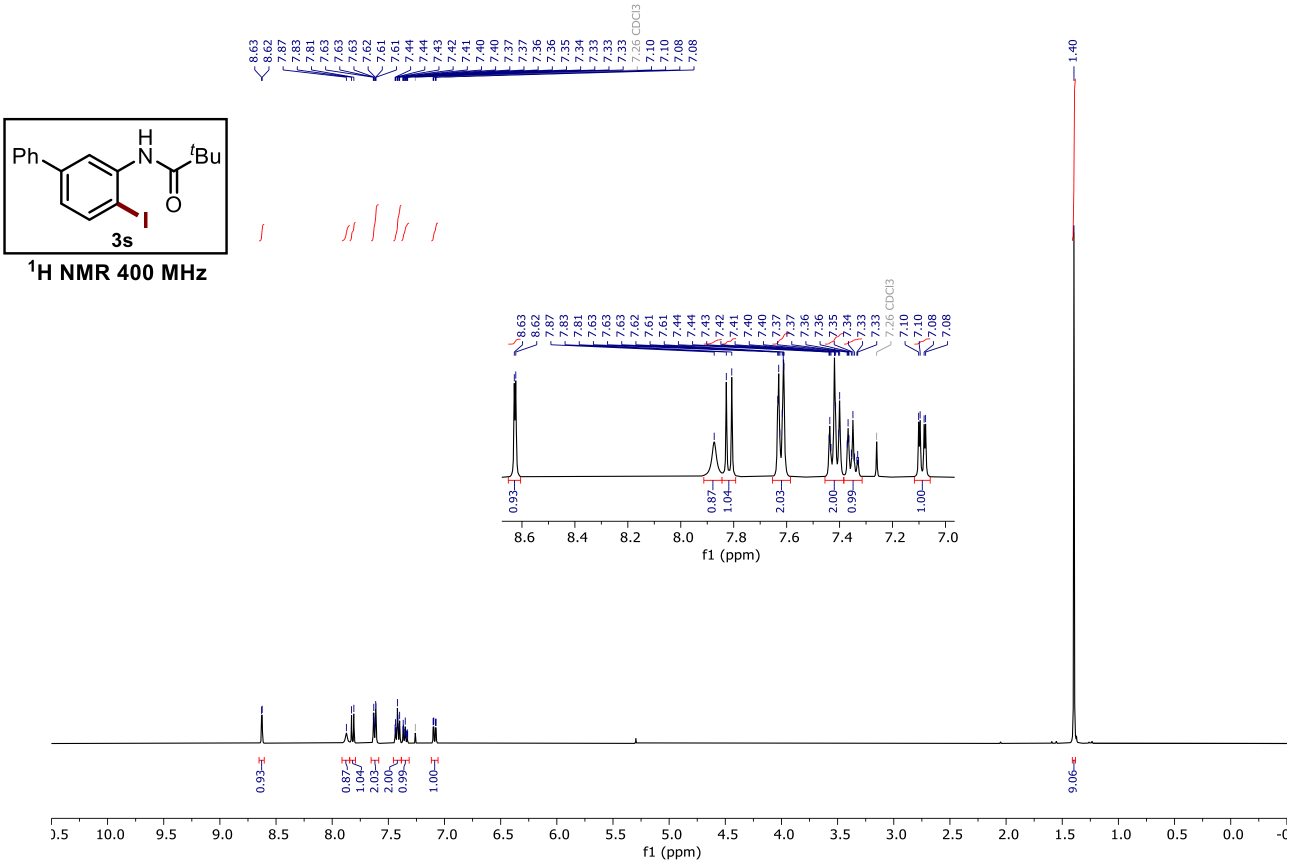
1.40
7.26 CDCl3
Ph
H
N
tBu
O
I
3s
1H NMR 400 MHz
f1 (ppm)

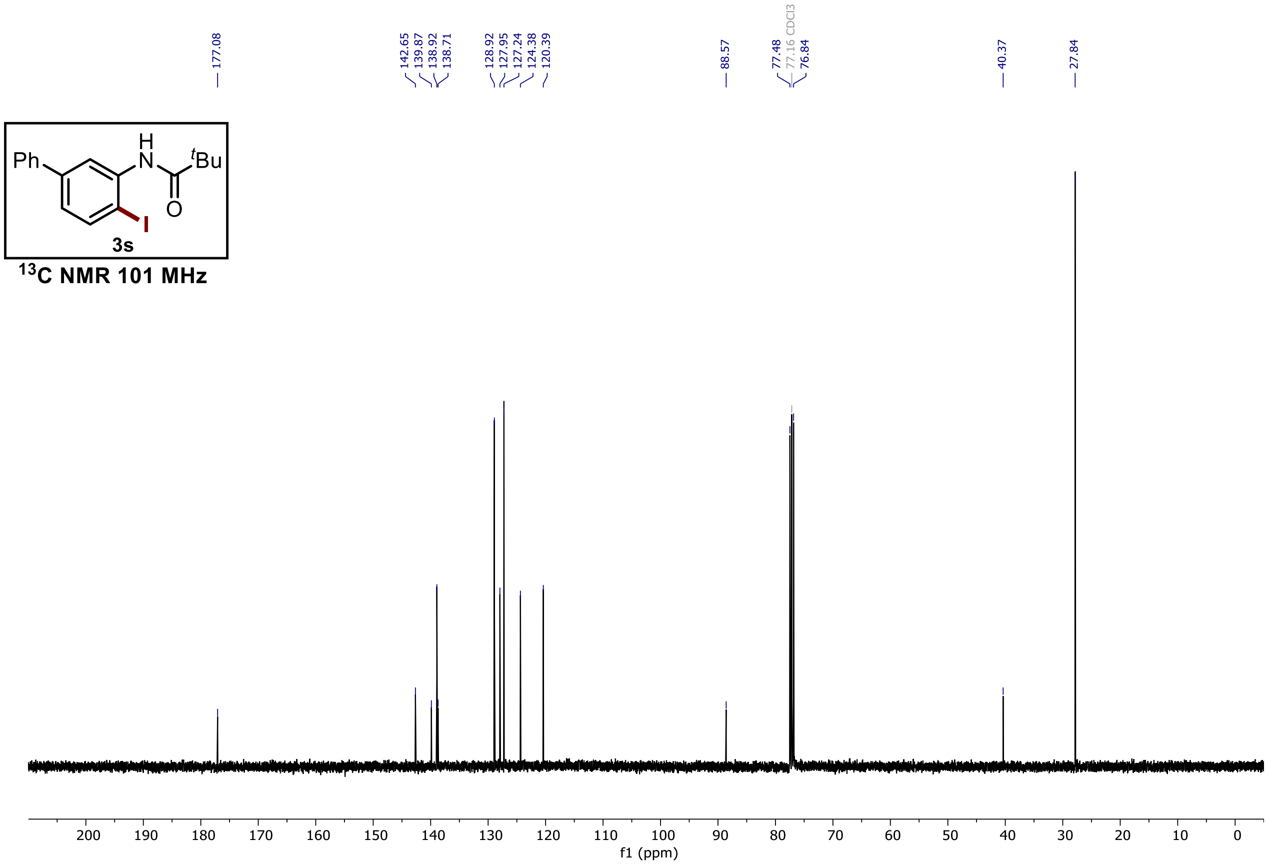

3s
^{13}C NMR 101 MHz
177.08
142.65
139.87
138.92
138.71
128.92
127.95
127.24
124.38
120.39
88.57
77.48
77.16 CDCl3
76.84
40.37
27.84
f1 (ppm)

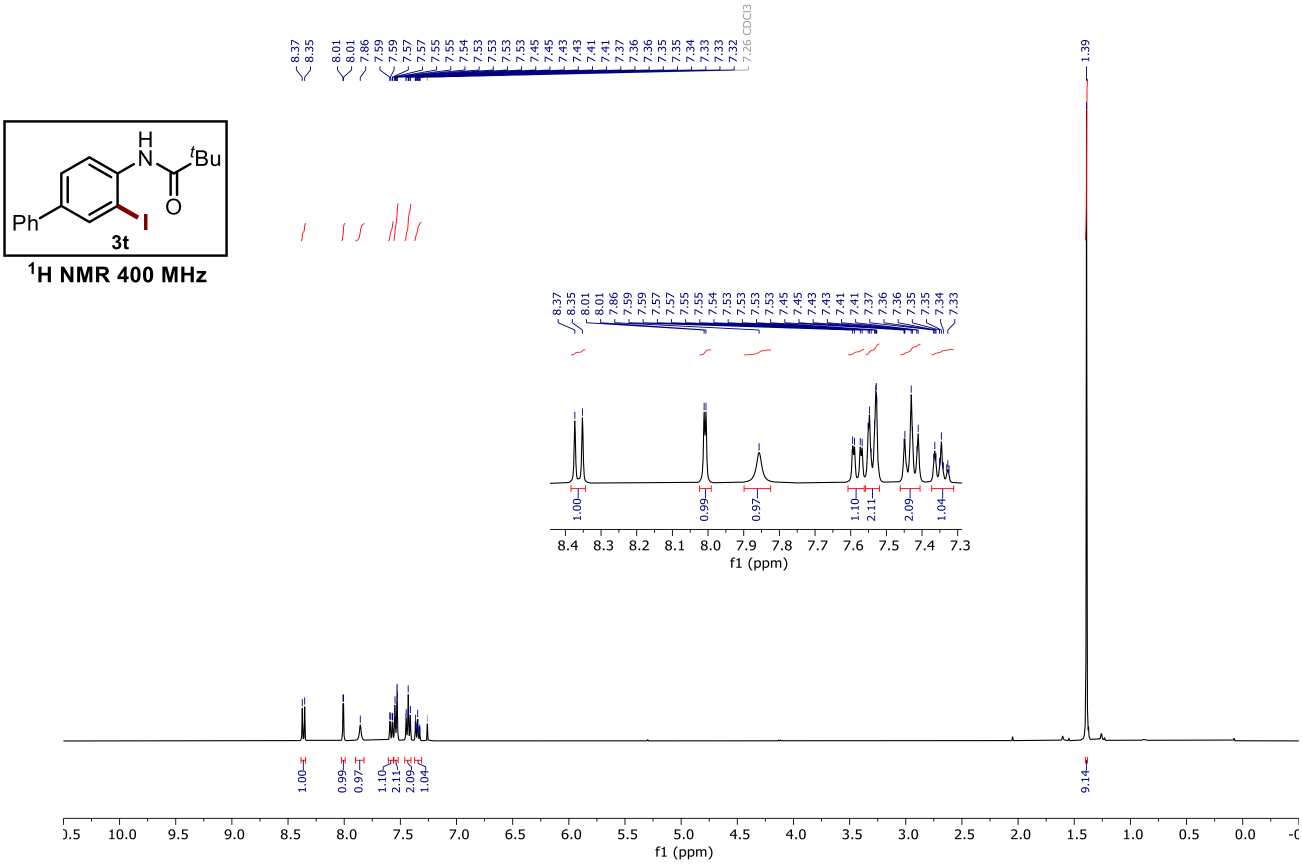

3t
^{1}H NMR 400 MHz
8.37
8.35
8.01
8.01
7.86
7.59
7.59
7.57
7.57
7.55
7.55
7.54
7.53
7.53
7.53
7.53
7.45
7.45
7.43
7.43
7.41
7.41
7.37
7.36
7.36
7.35
7.35
7.34
7.33
7.33
7.32
7.26 CDCl3
1.39
1.00
0.99
0.97
1.10
2.11
2.09
1.04
9.14
f1 (ppm)

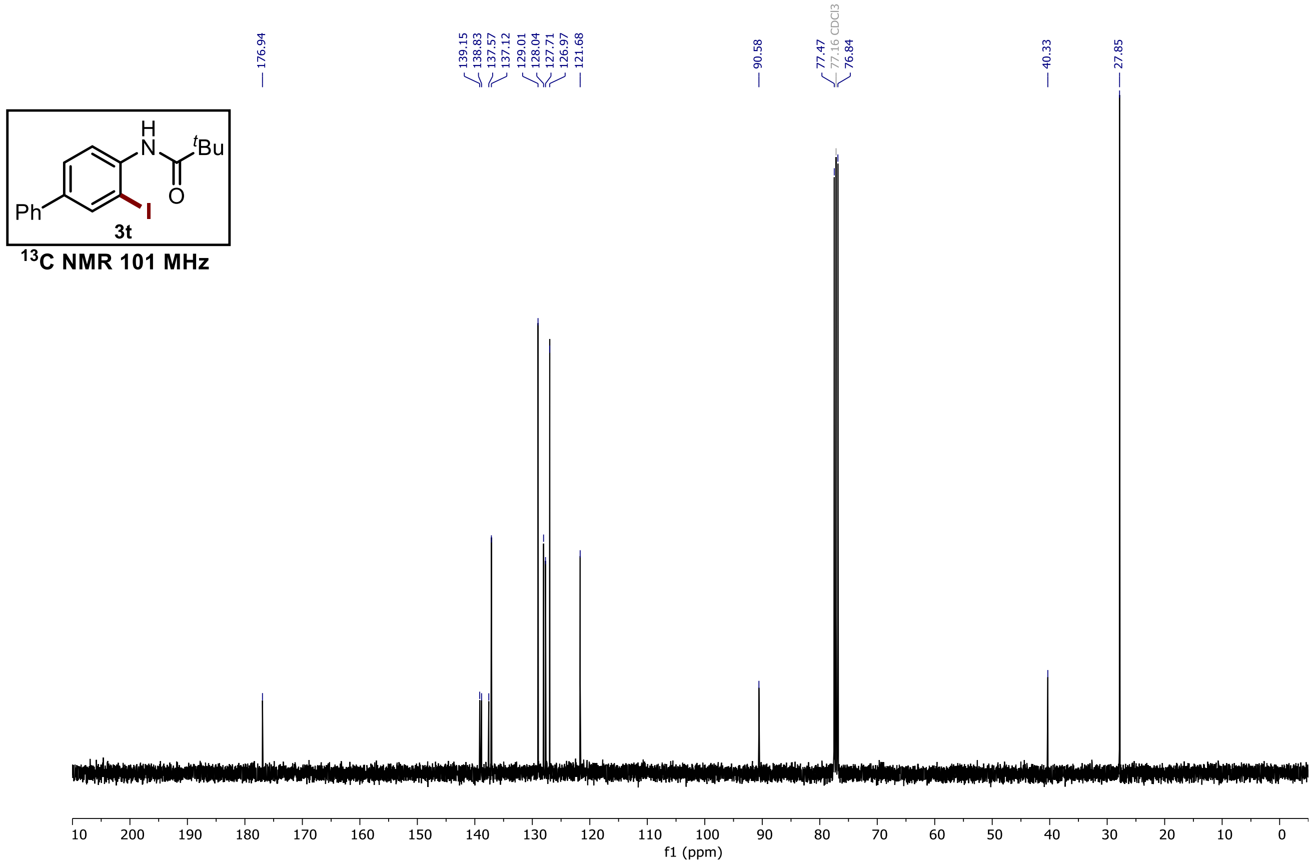

176.94
139.15
138.83
137.57
137.12
129.01
128.04
127.71
126.97
121.68
90.58
77.47
77.16 CDCl3
76.84
40.33
27.85
H
N
tBu
O
Ph
I
3t
13C NMR 101 MHz
f1 (ppm)

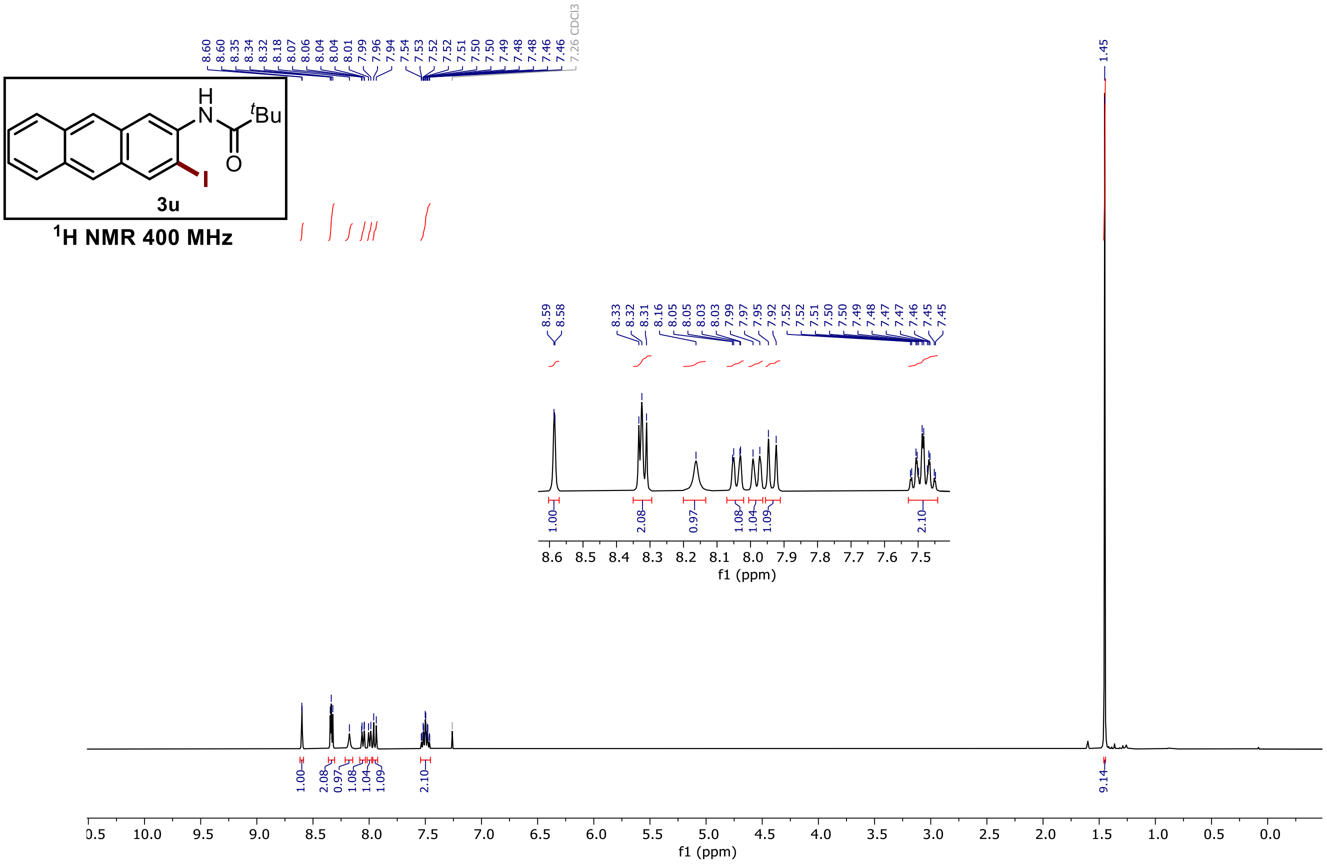

8.60
8.60
8.35
8.34
8.32
8.18
8.07
8.06
8.04
8.04
8.01
7.99
7.96
7.94
7.54
7.53
7.52
7.52
7.51
7.50
7.50
7.49
7.48
7.48
7.46
7.46
7.26 CDCl3
1.45
H
N
tBu
O
I
3u
1H NMR 400 MHz
1.00
2.08
0.97
1.08
1.04
1.09
2.10
9.14
f1 (ppm)

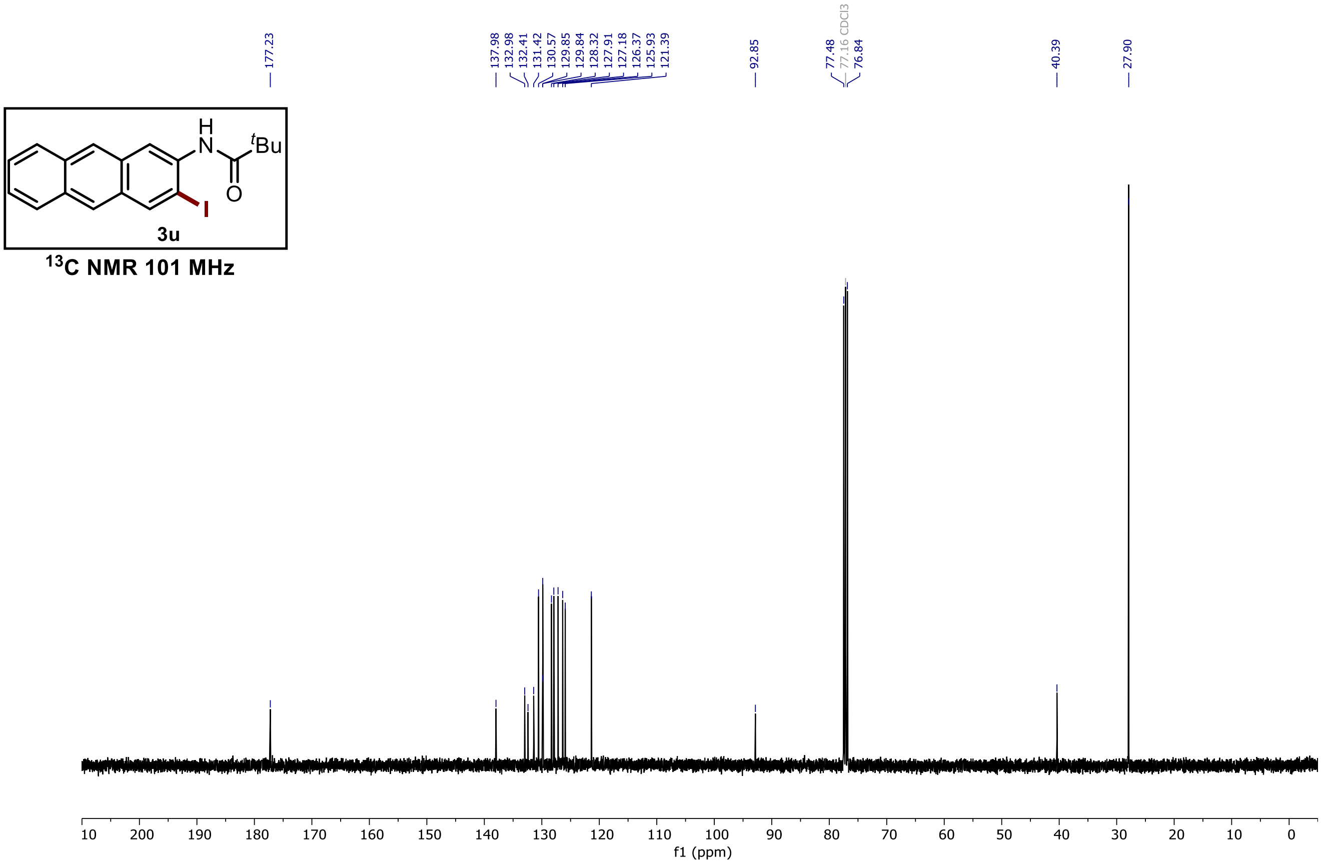

^{13}C NMR 101 MHz

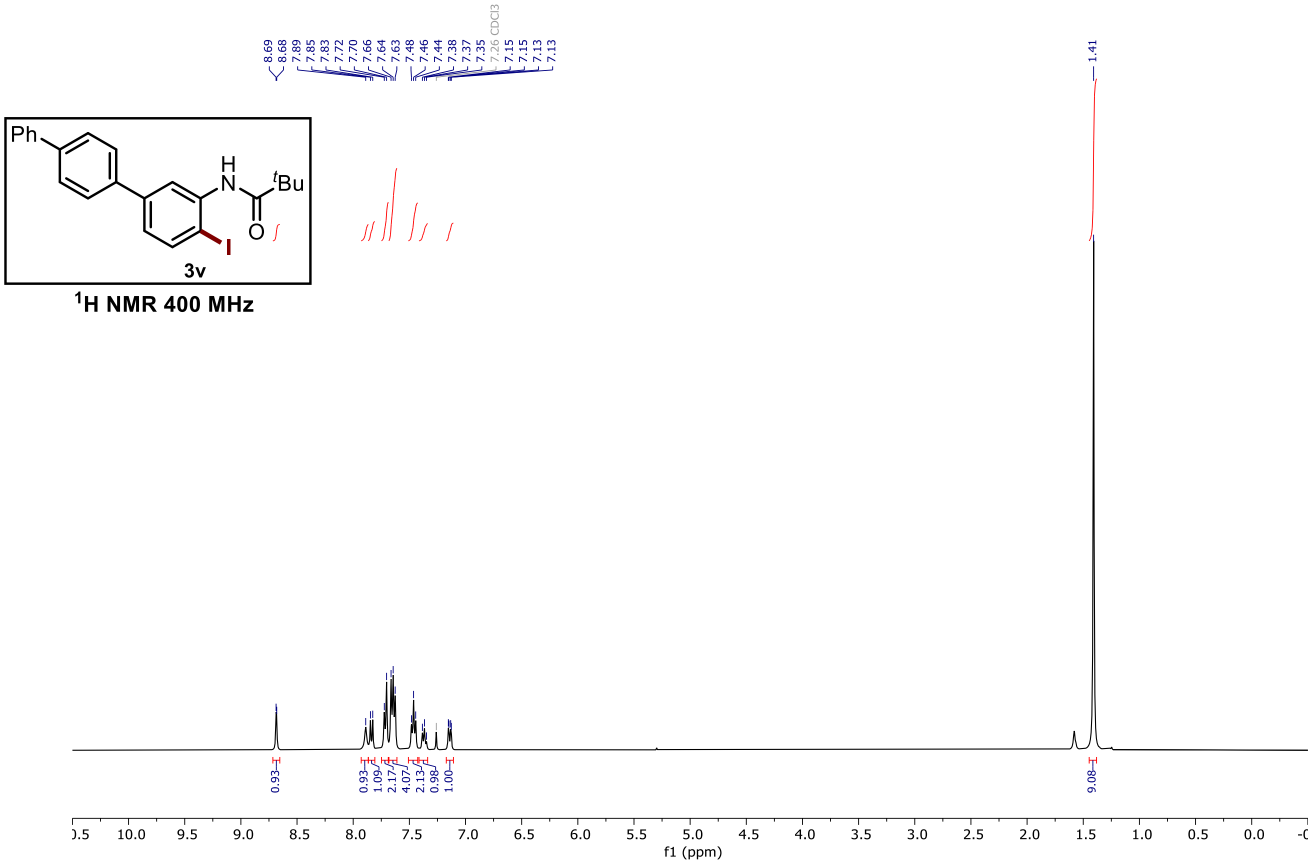

^{1}H NMR 400 MHz

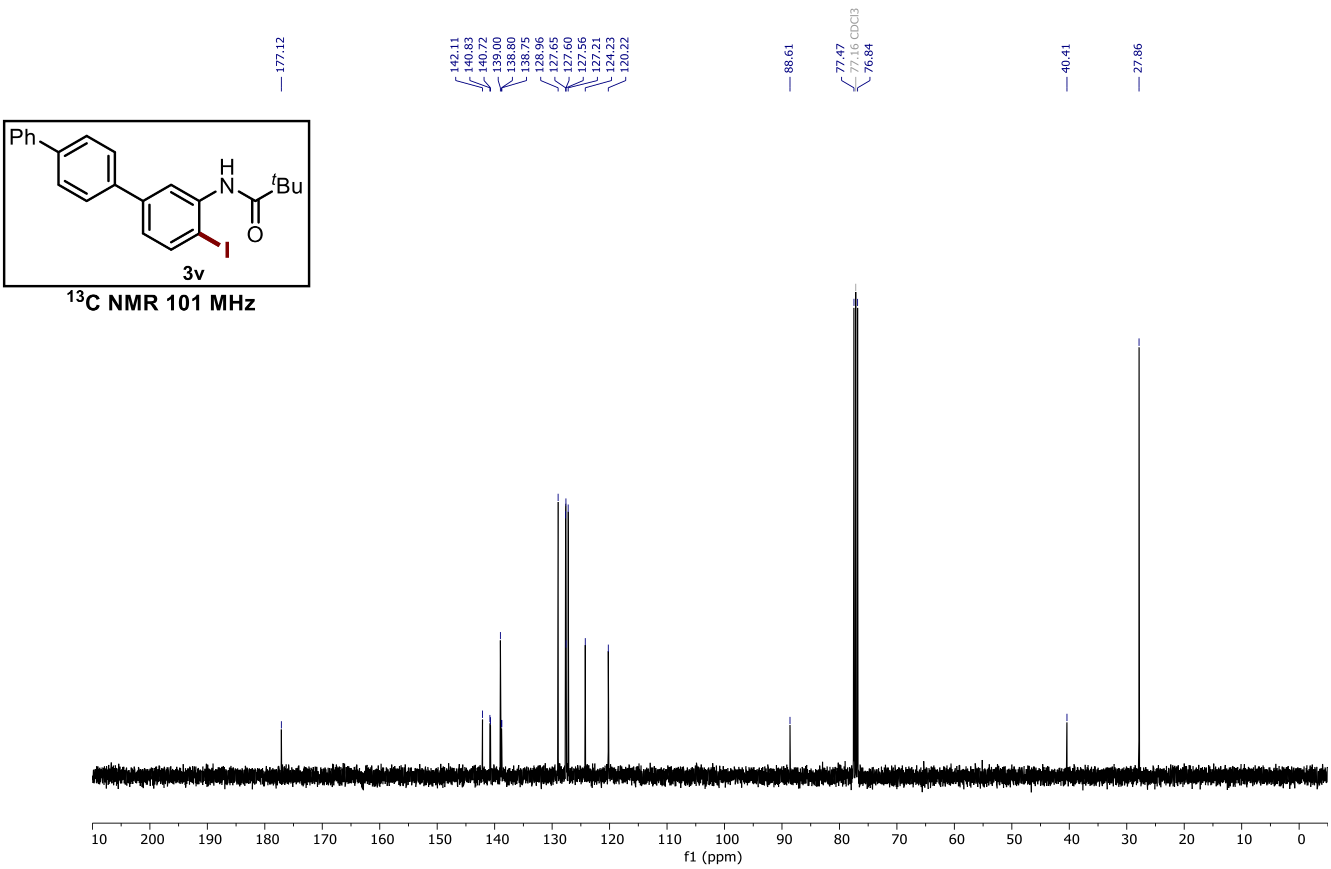
Ph
H
N
tBu
O
I
3v
13C NMR 101 MHz
177.12
142.11
140.83
140.72
139.00
138.80
138.75
128.96
127.65
127.60
127.56
127.21
124.23
120.22
88.61
77.47
77.16 CDCl3
76.84
40.41
27.86
f1 (ppm)

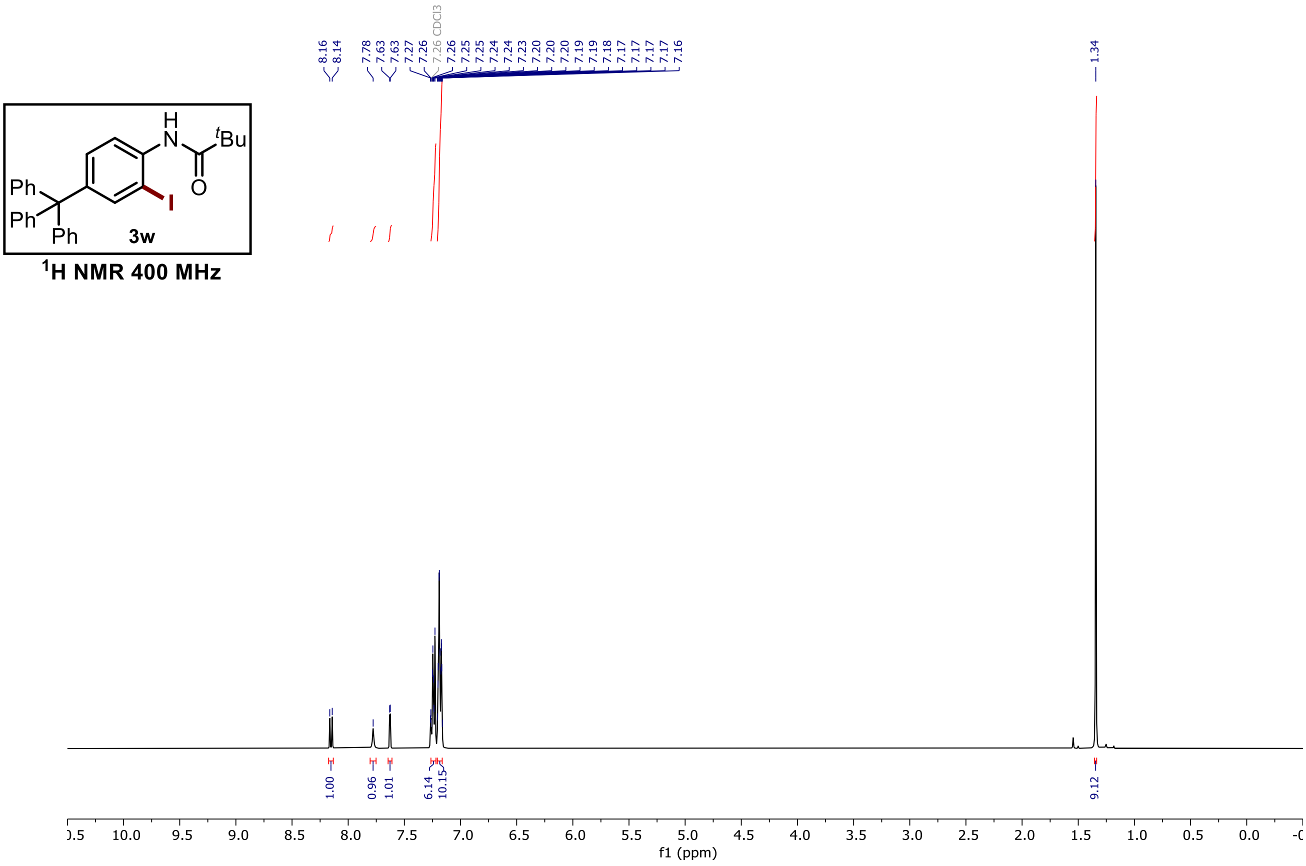
H
N
tBu
O
I
Ph
Ph
Ph
3w
1H NMR 400 MHz
8.16
8.14
7.78
7.63
7.63
7.27
7.26
7.26 CDCl3
7.26
7.25
7.25
7.24
7.24
7.23
7.20
7.20
7.20
7.19
7.19
7.18
7.17
7.17
7.17
7.17
7.16
1.34
1.00
0.96
1.01
6.14
10.15
9.12
f1 (ppm)

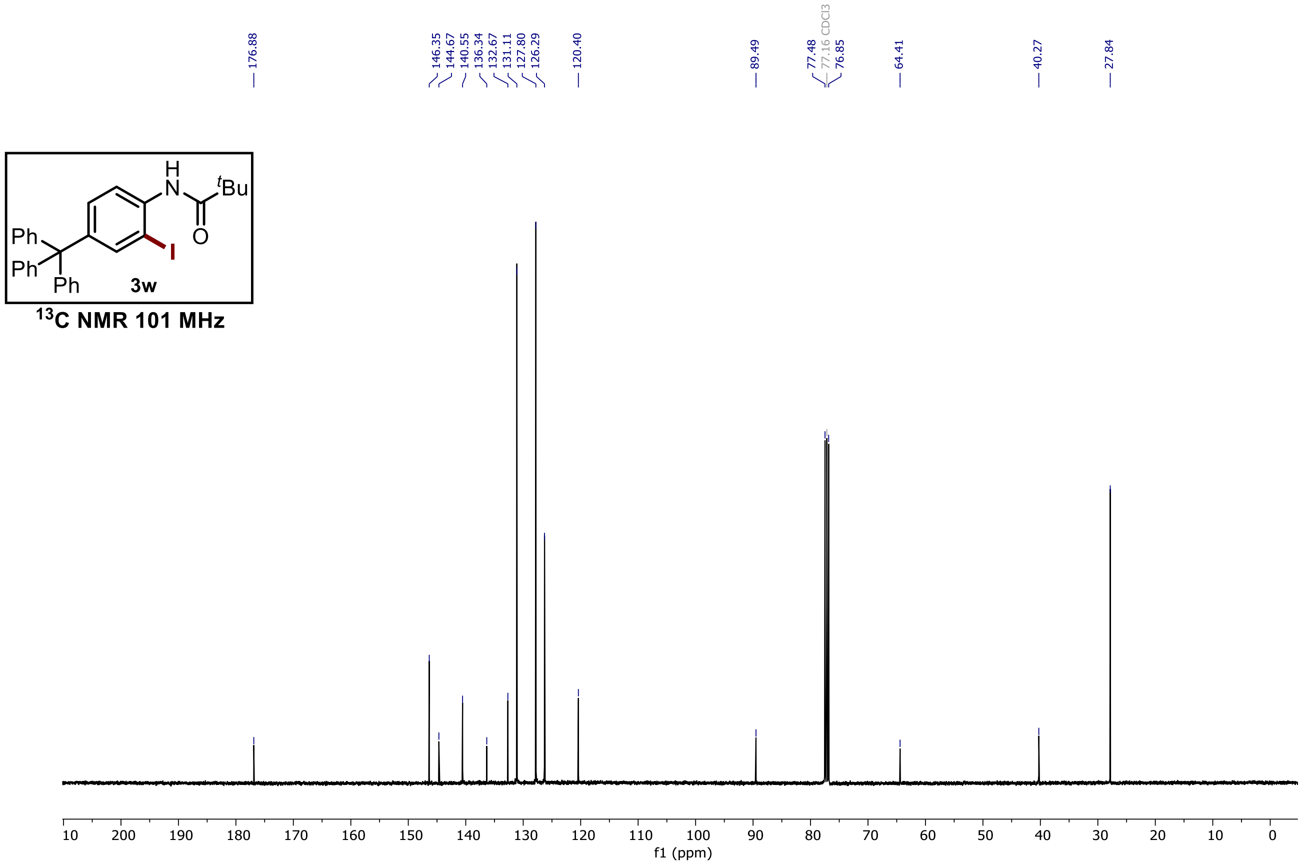
176.88
146.35
144.67
140.55
136.34
132.67
131.11
127.80
126.29
120.40
89.49
77.48
77.16 CDCl3
76.85
64.41
40.27
27.84
Ph
Ph
Ph
H
N
tBu
O
I
3w
13C NMR 101 MHz
f1 (ppm)

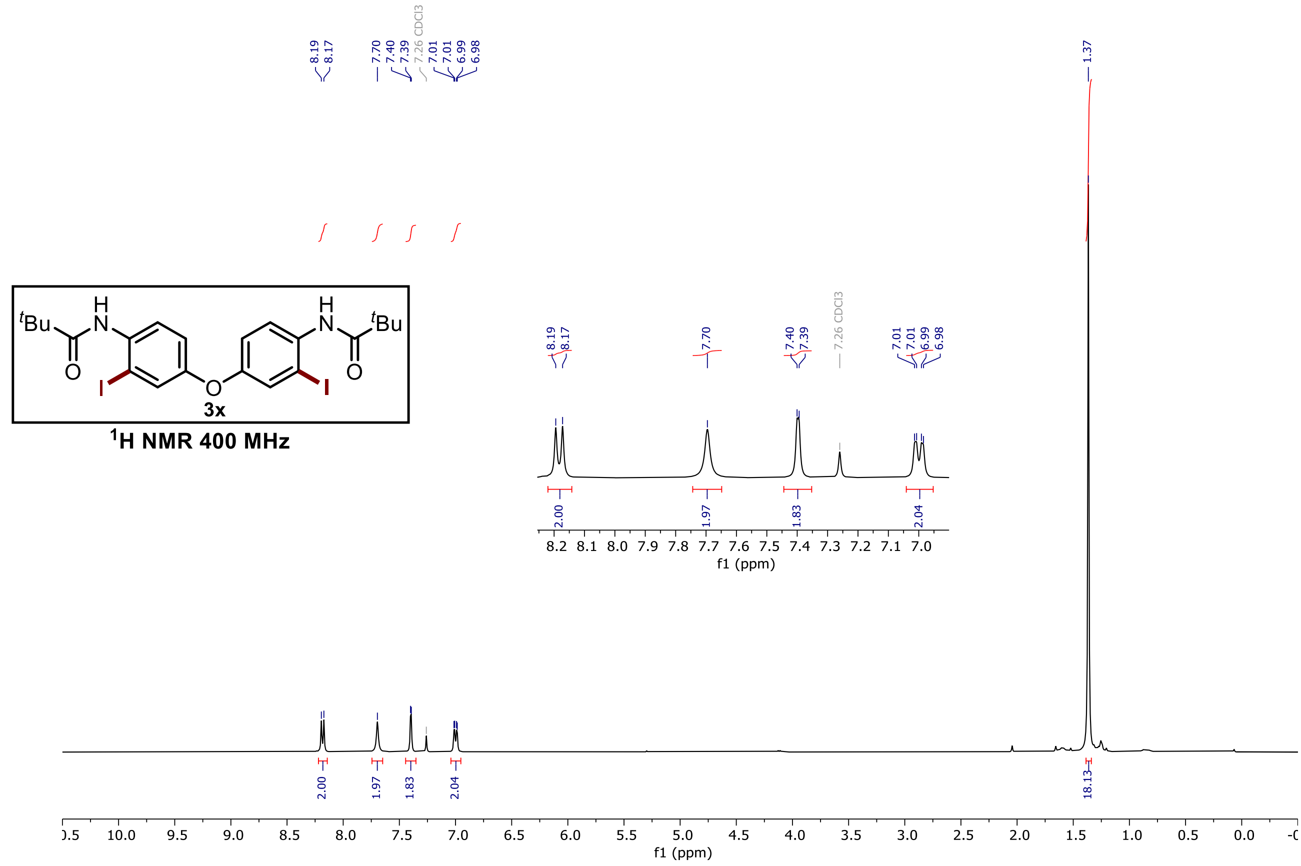
8.19
8.17
7.70
7.40
7.39
7.26 CDCl3
7.01
7.01
6.99
6.98
1.37
tBu
H
N
O
I
3x
1H NMR 400 MHz
2.00
1.97
1.83
2.04
18.13
f1 (ppm)

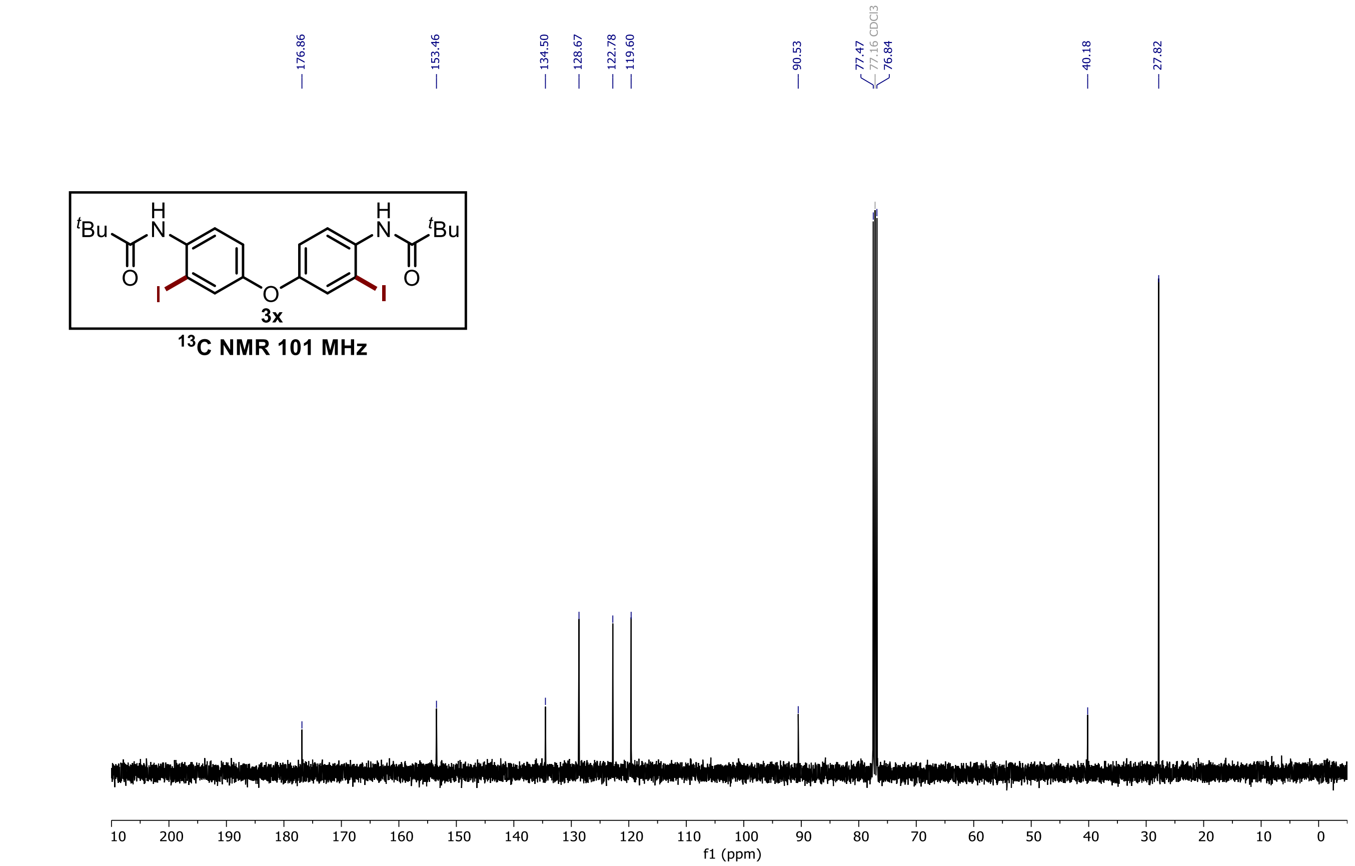
176.86
153.46
134.50
128.67
122.78
119.60
90.53
77.47
77.16 CDCl3
76.84
40.18
27.82
3x
13C NMR 101 MHz
f1 (ppm)

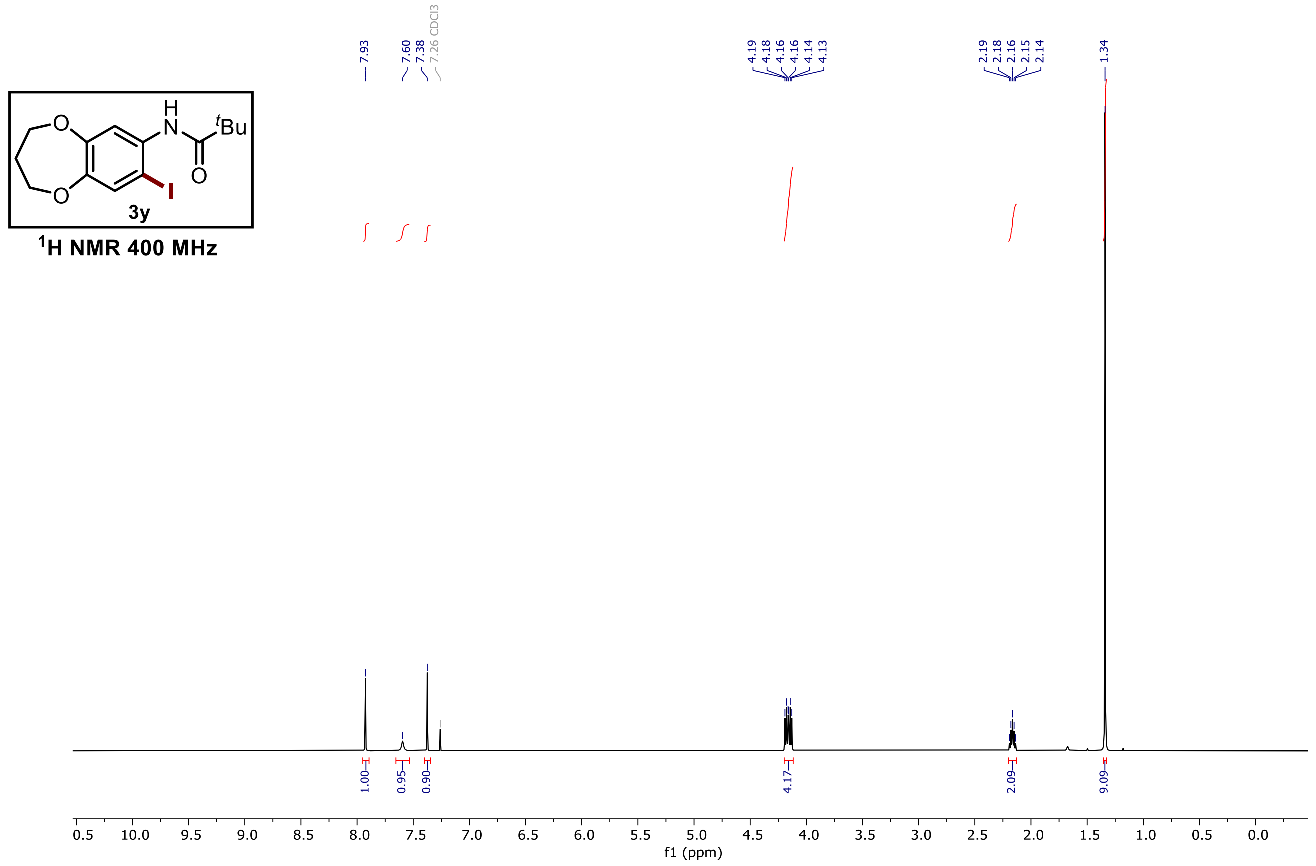
7.93
7.60
7.38
7.26 CDCl3
4.19
4.18
4.16
4.16
4.14
4.13
2.19
2.18
2.16
2.15
2.14
1.34
3y
1H NMR 400 MHz
1.00
0.95
0.90
4.17
2.09
9.09
f1 (ppm)

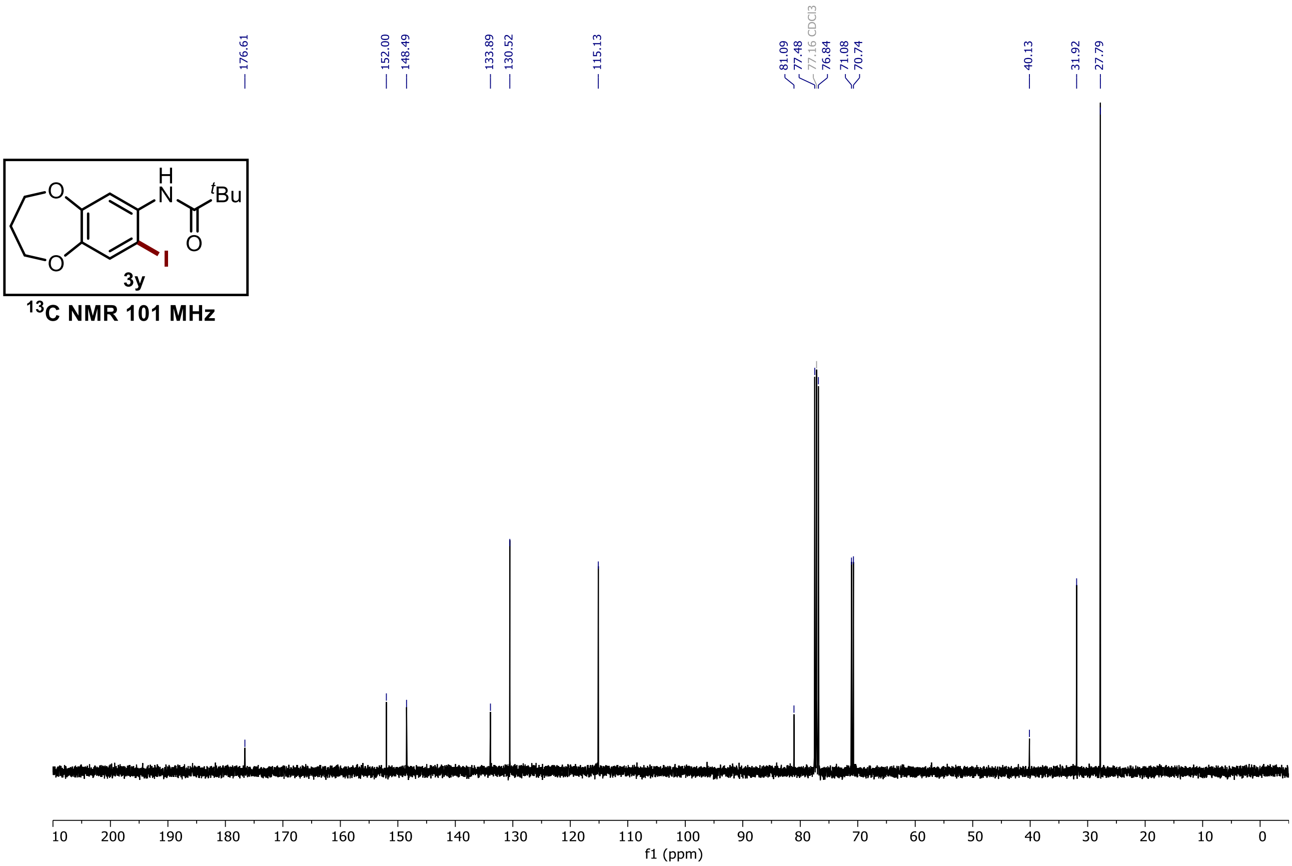
176.61
152.00
148.49
133.89
130.52
115.13
81.09
77.48
77.16 CDCl3
76.84
71.08
70.74
40.13
31.92
27.79
3y
13C NMR 101 MHz
f1 (ppm)

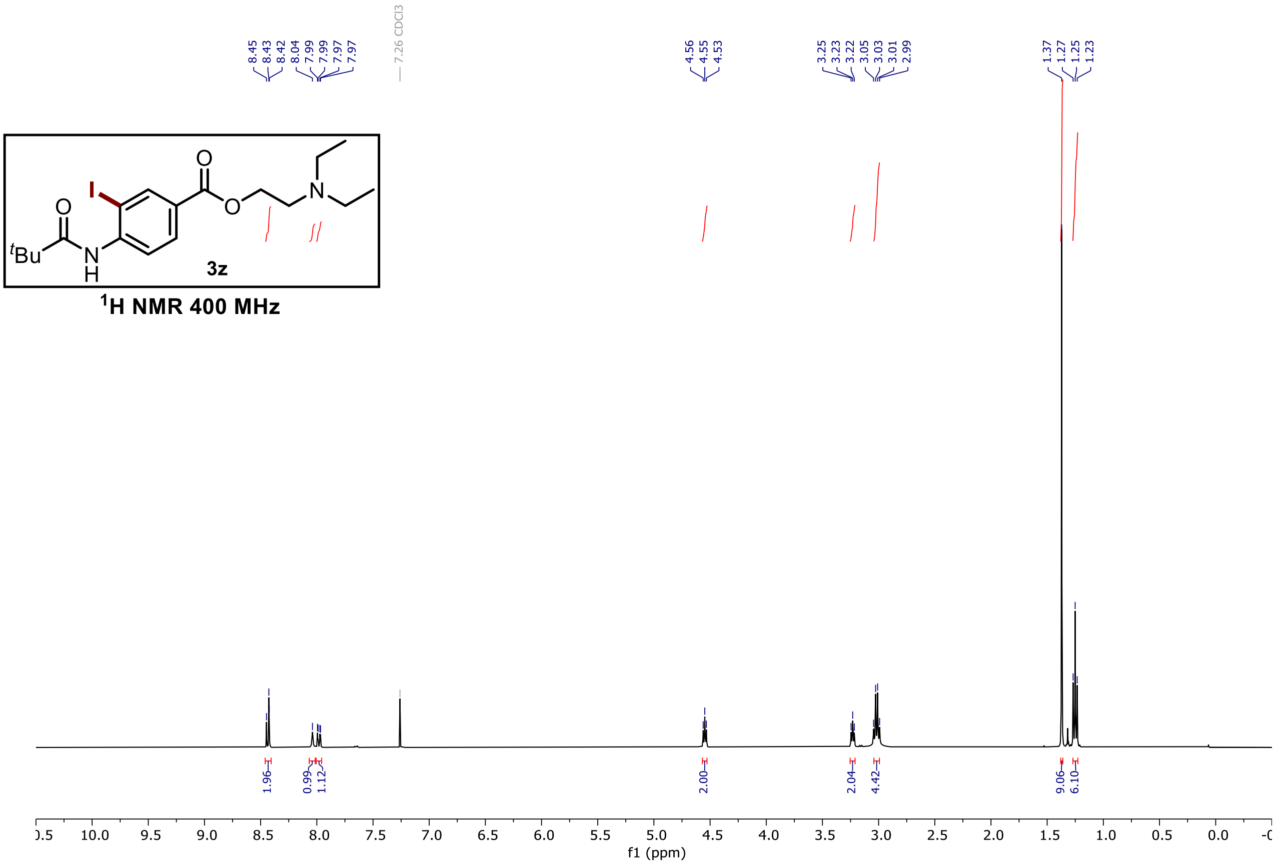
8.45
8.43
8.42
8.04
7.99
7.99
7.97
7.97
7.26 CDCl3
4.56
4.55
4.53
3.25
3.23
3.22
3.05
3.03
3.01
2.99
1.37
1.27
1.25
1.23
3z
1H NMR 400 MHz
1.96
0.99
1.12
2.00
2.04
4.42
9.06
6.10
f1 (ppm)

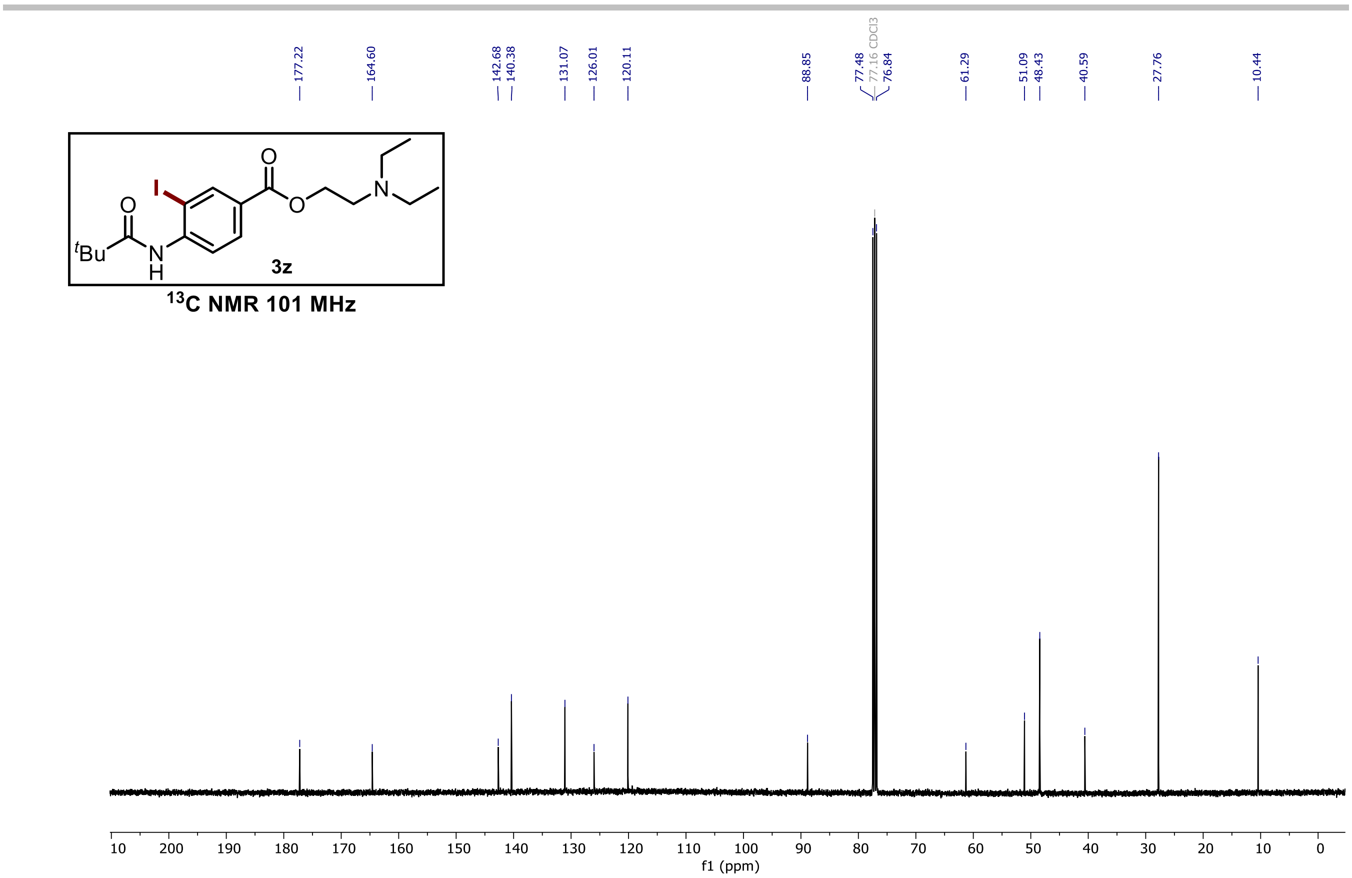

3z
13C NMR 101 MHz
177.22
164.60
142.68
140.38
131.07
126.01
120.11
88.85
77.48
77.16 CDCl3
76.84
61.29
51.09
48.43
40.59
27.76
10.44
f1 (ppm)

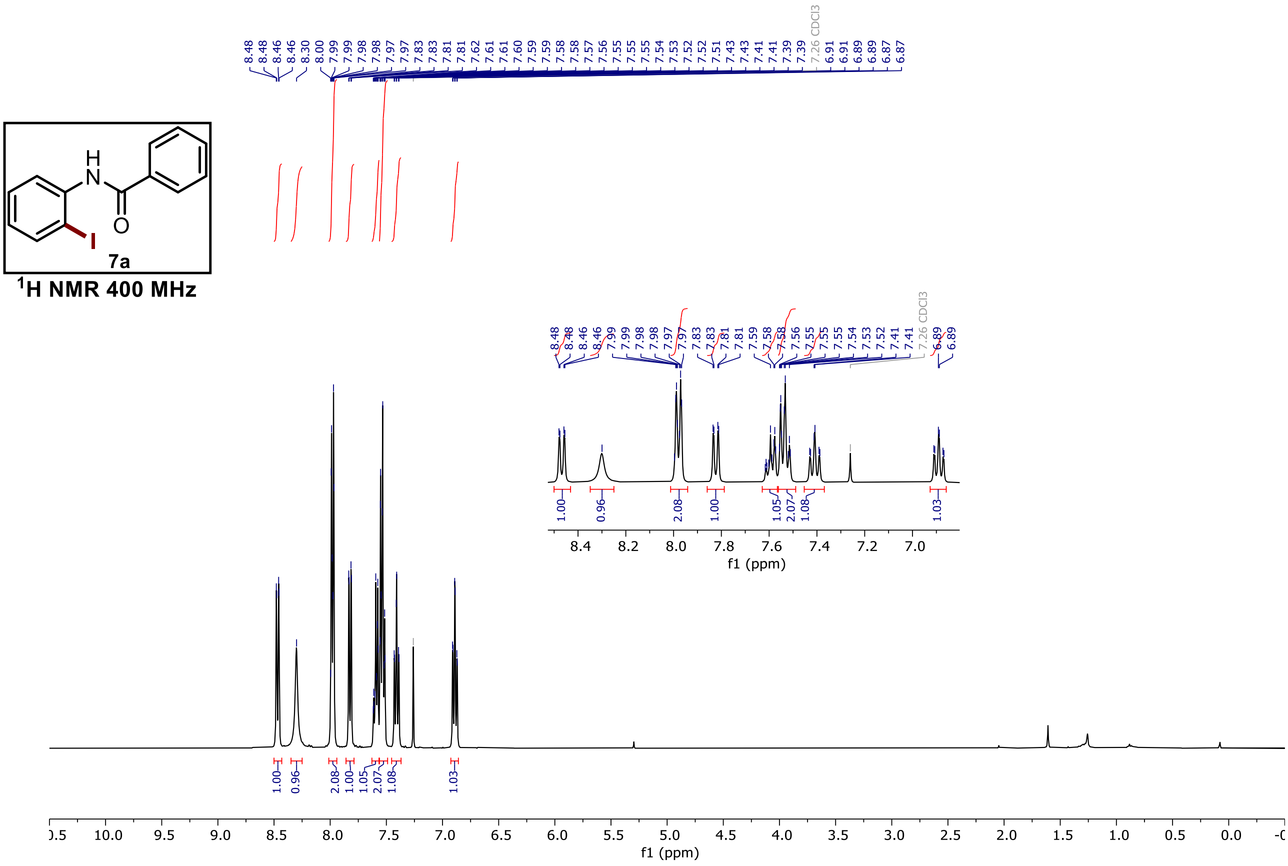

7a
1H NMR 400 MHz
7.26 CDCl3
1.00
0.96
2.08
1.00
1.05
2.07
1.08
1.03
f1 (ppm)

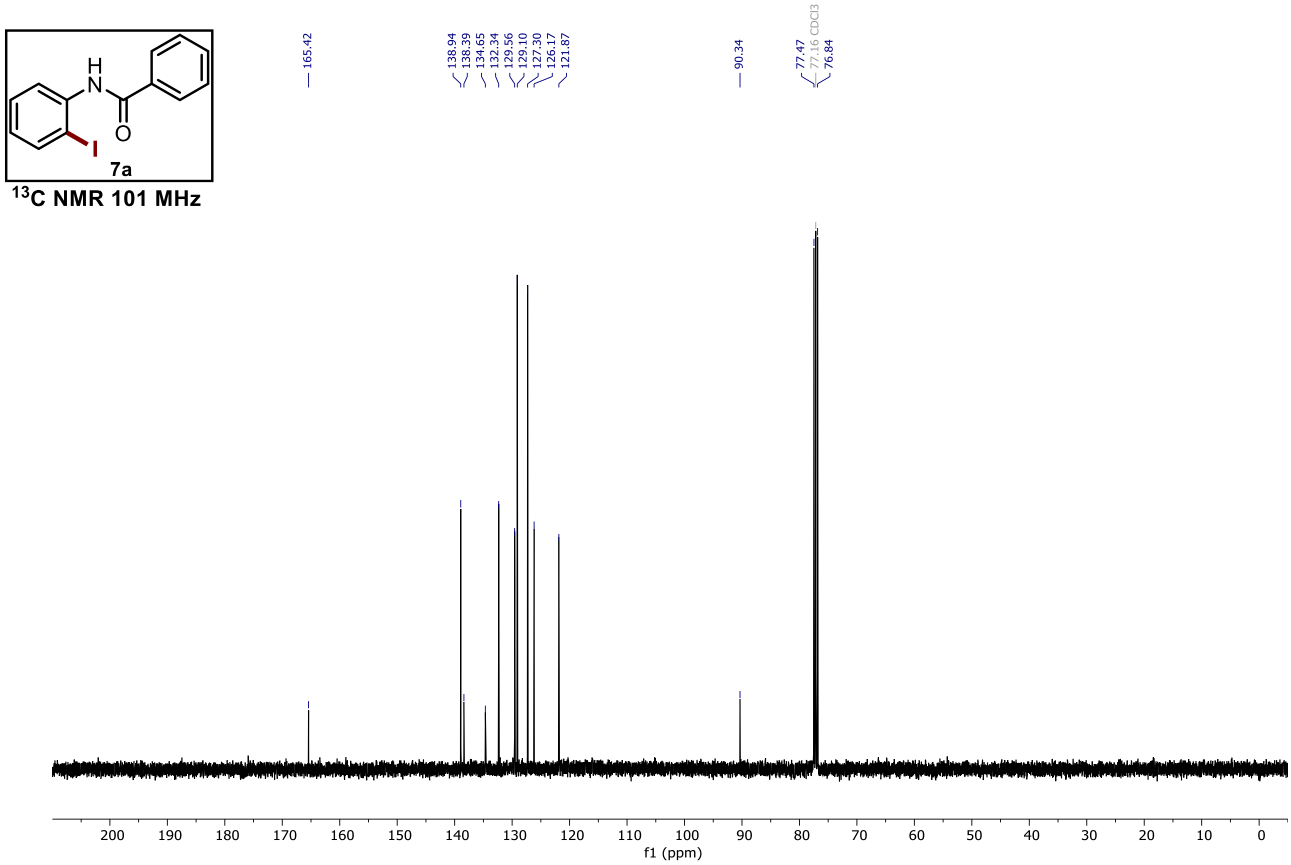
7a
13C NMR 101 MHz
165.42
138.94
138.39
134.65
132.34
129.56
129.10
127.30
126.17
121.87
90.34
77.47
77.16 CDCl3
76.84
f1 (ppm)

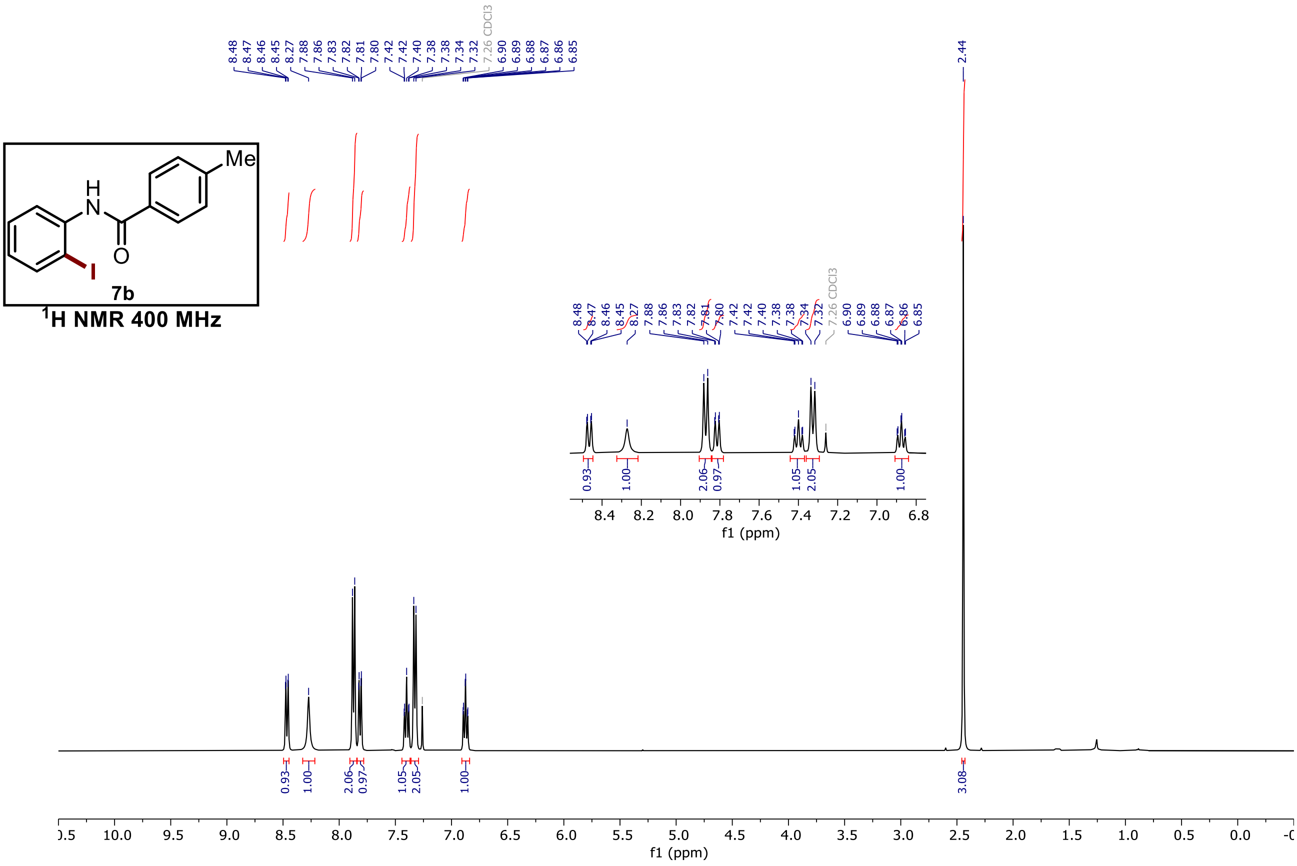
Me
7b
1H NMR 400 MHz
8.48
8.47
8.46
8.45
8.27
7.88
7.86
7.83
7.82
7.81
7.80
7.42
7.42
7.40
7.38
7.38
7.34
7.32
7.26 CDCl3
6.90
6.89
6.88
6.87
6.86
6.85
2.44
0.93
1.00
2.06
0.97
1.05
2.05
1.00
3.08
f1 (ppm)

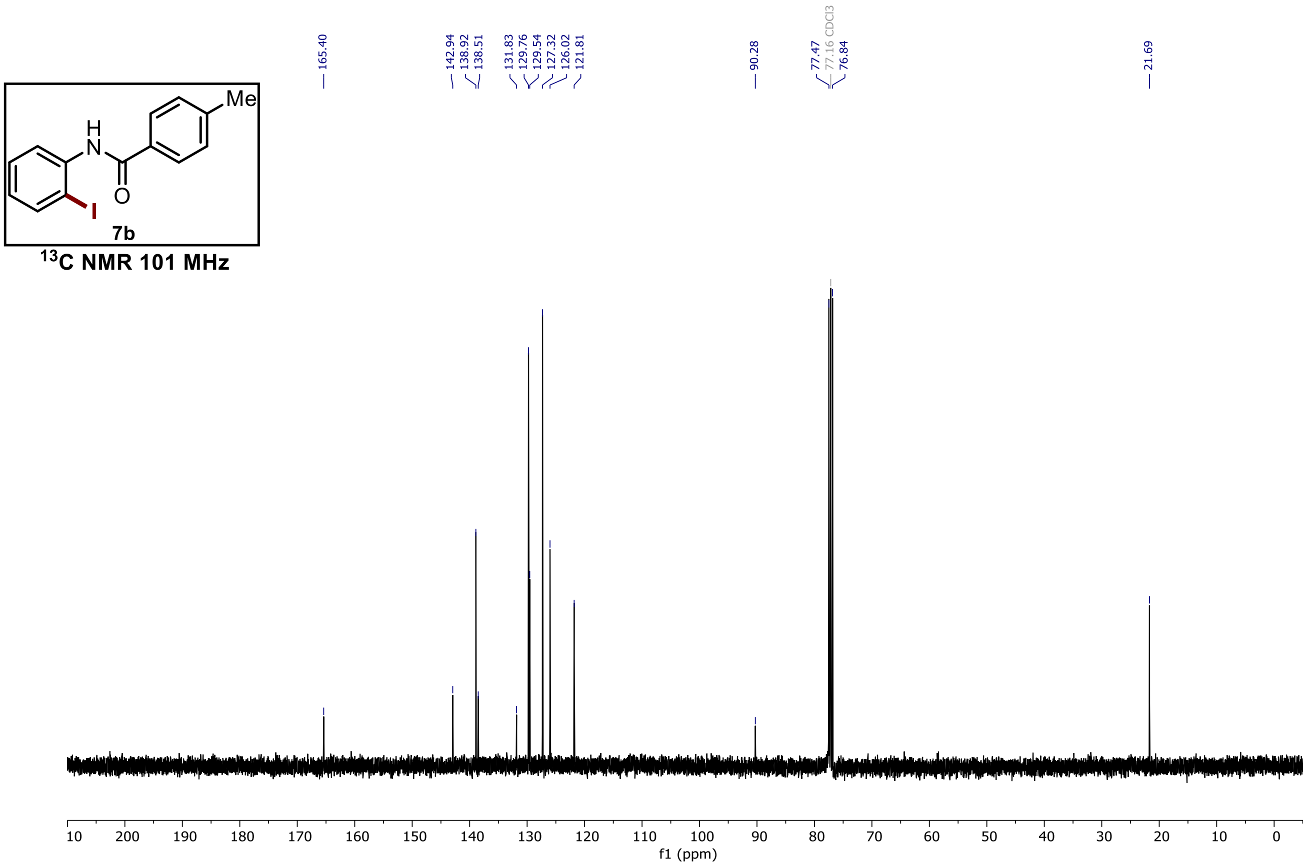
Me
H
N
O
I
7b
13C NMR 101 MHz
f1 (ppm)

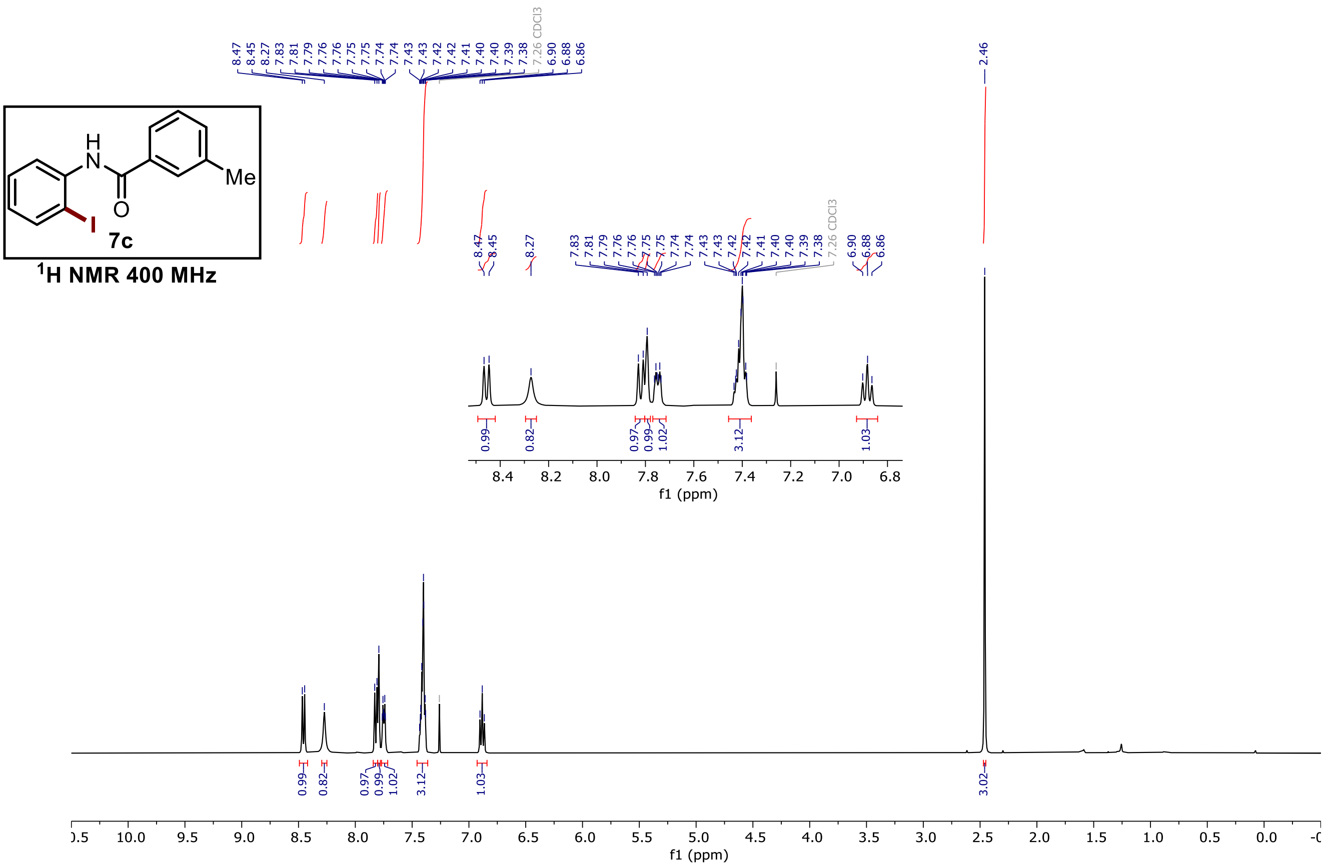
Me
H
N
O
I
7c
1H NMR 400 MHz
f1 (ppm)

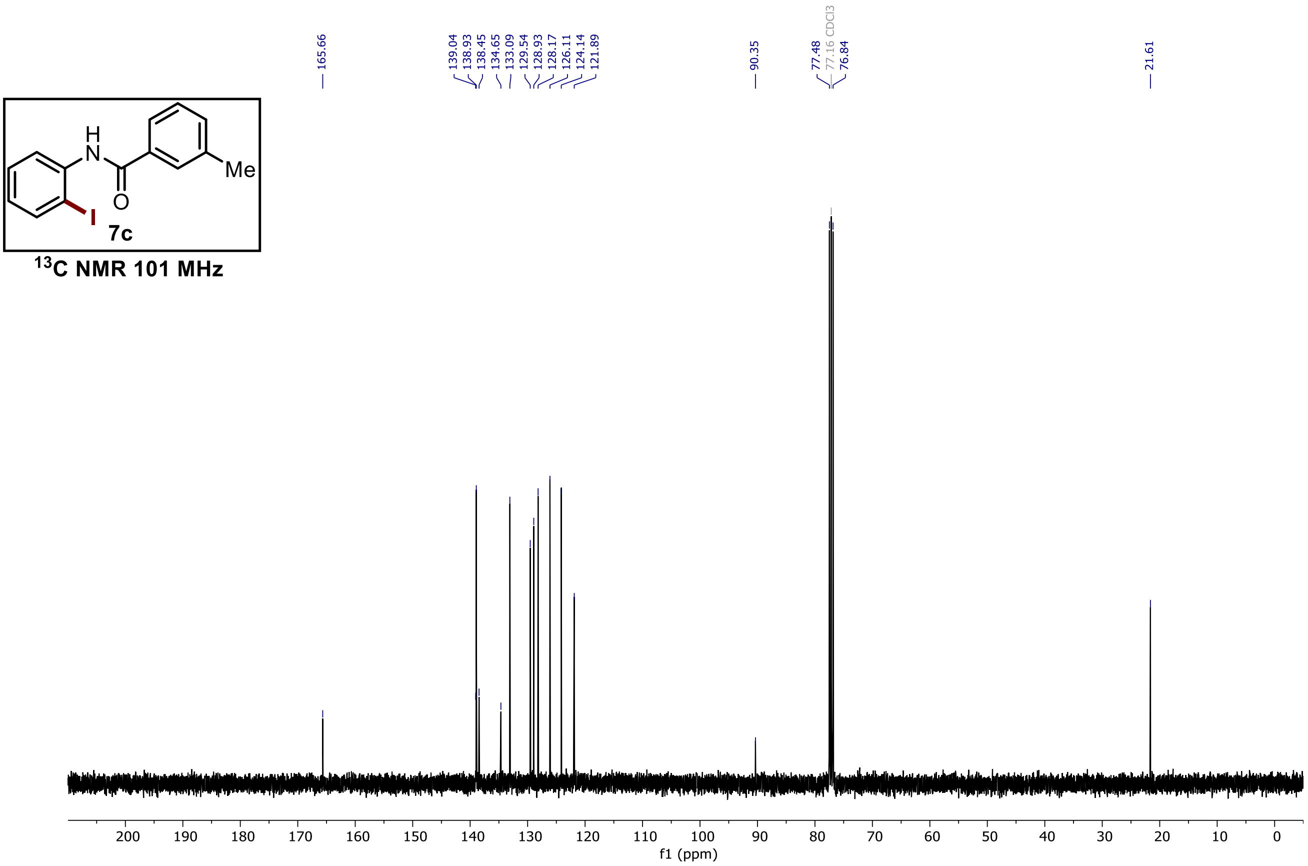

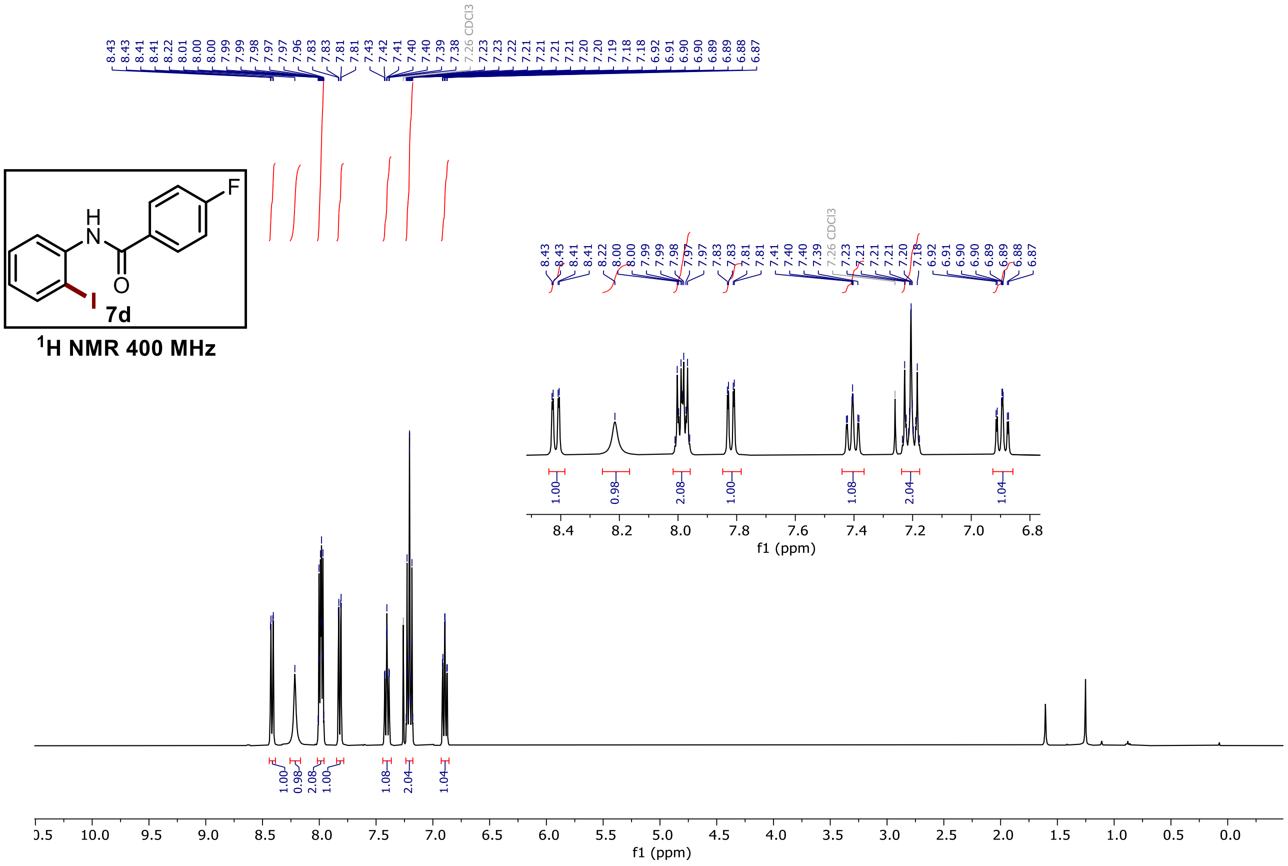

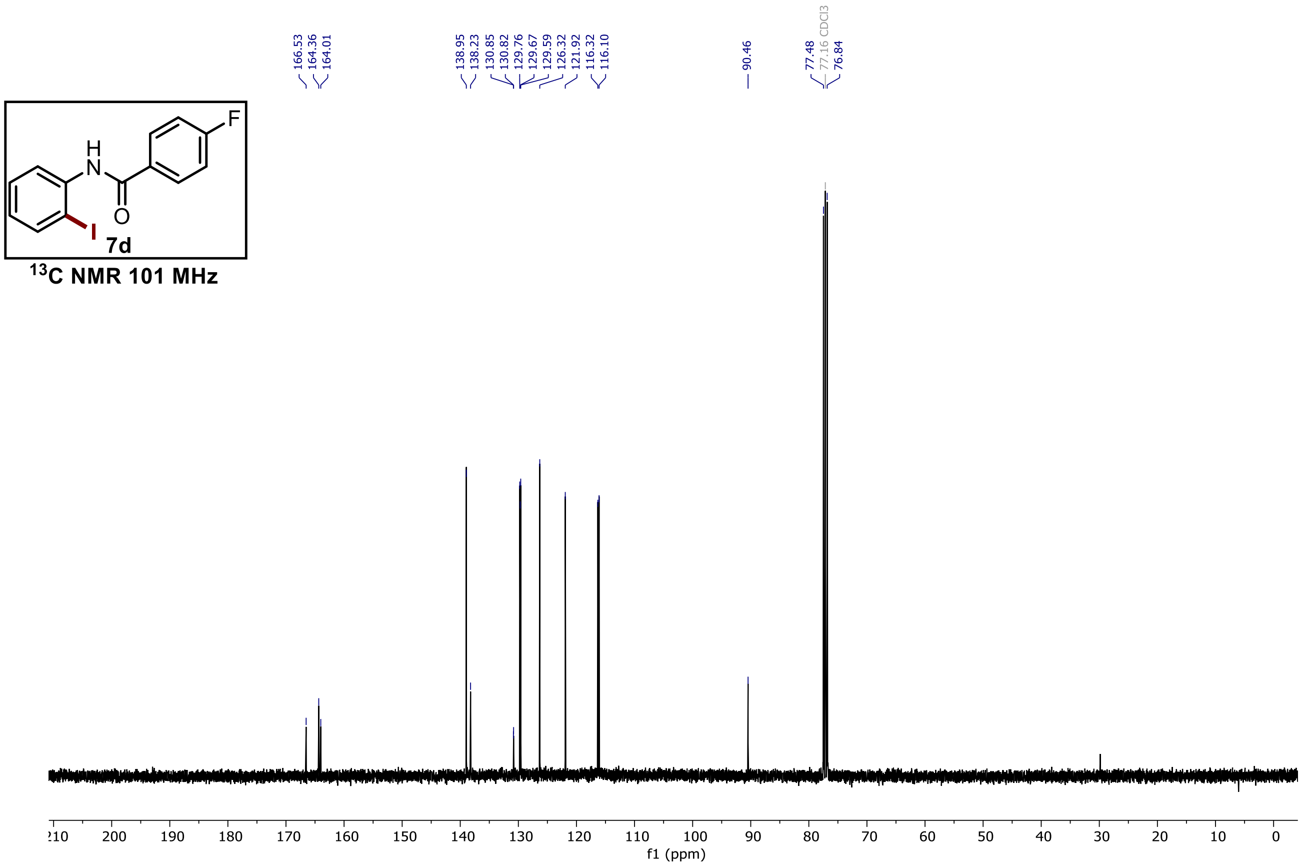

^{13}C NMR 101 MHz

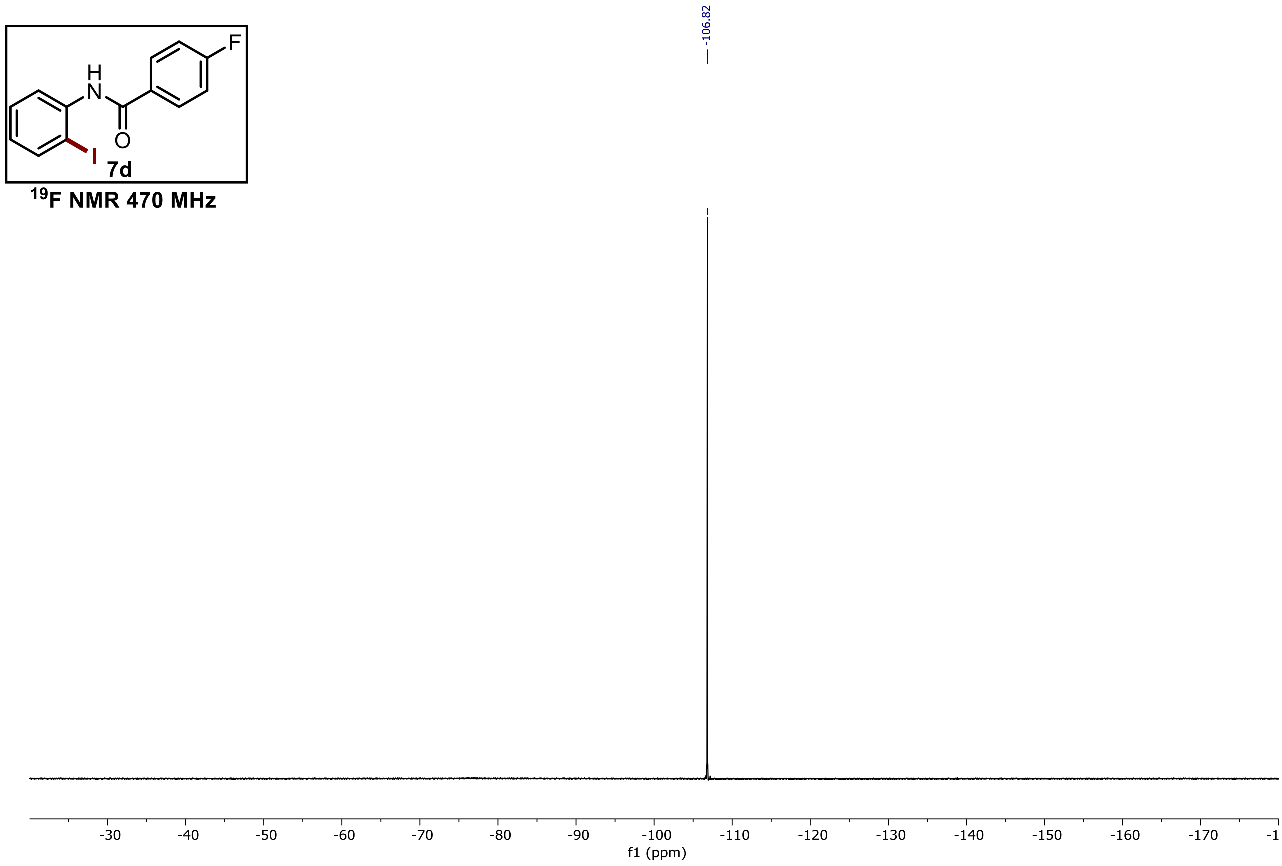

^{19}F NMR 470 MHz

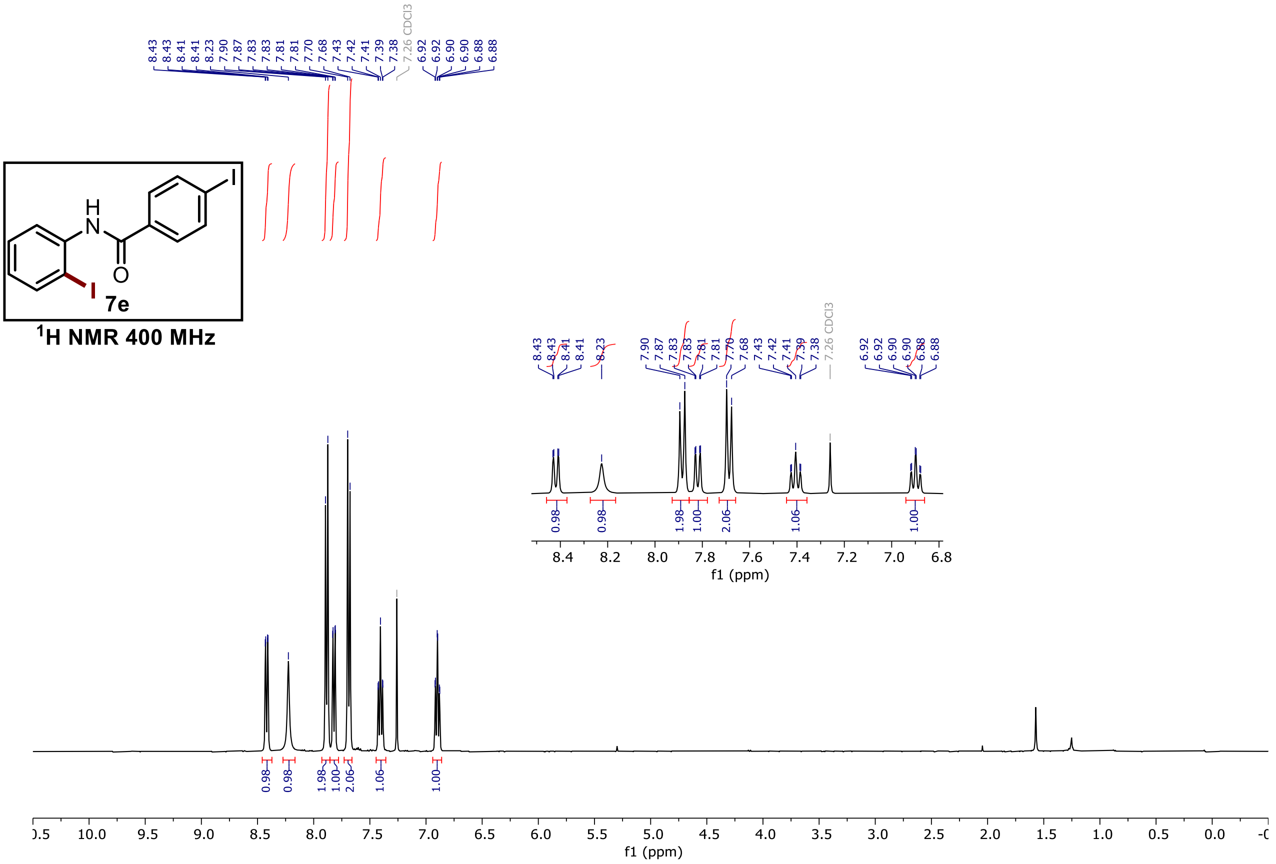
7e
1H NMR 400 MHz

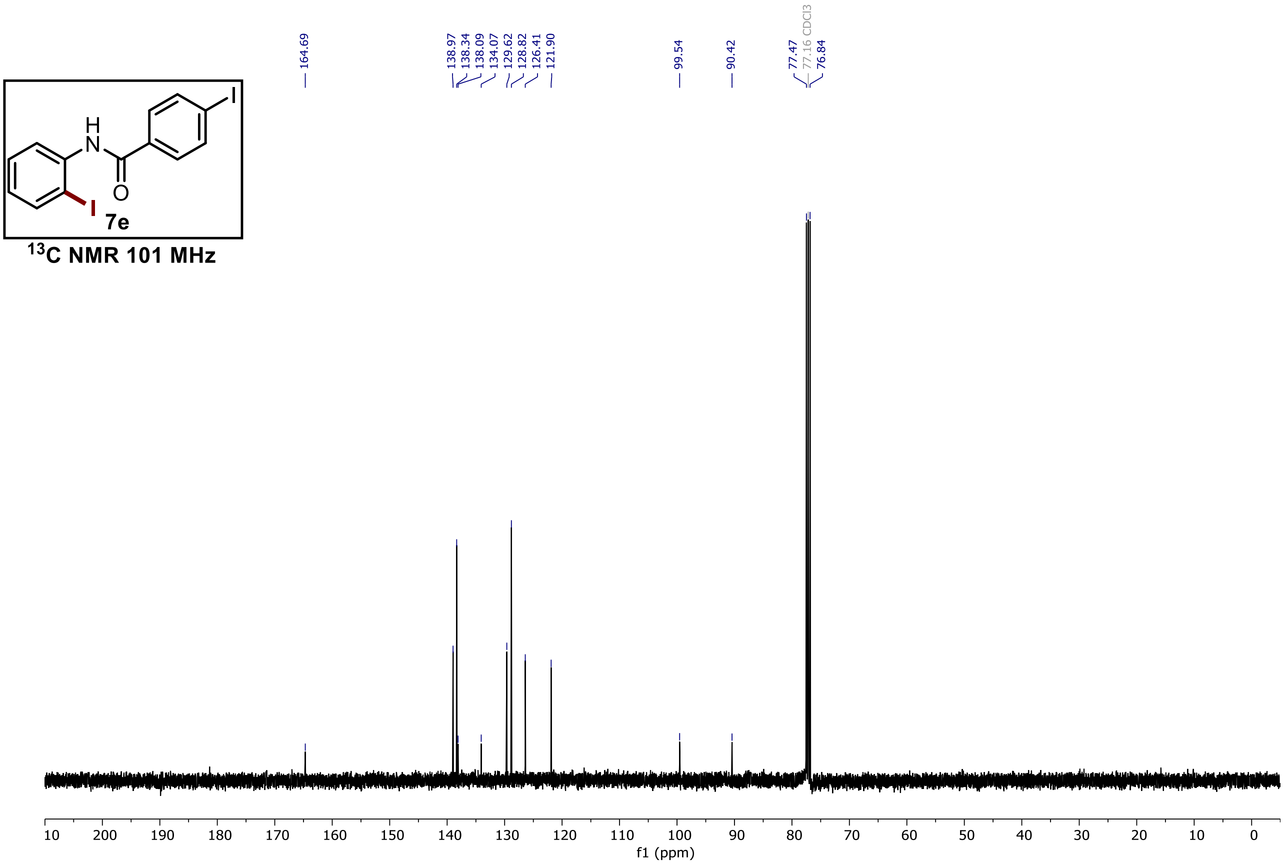
7e
13C NMR 101 MHz

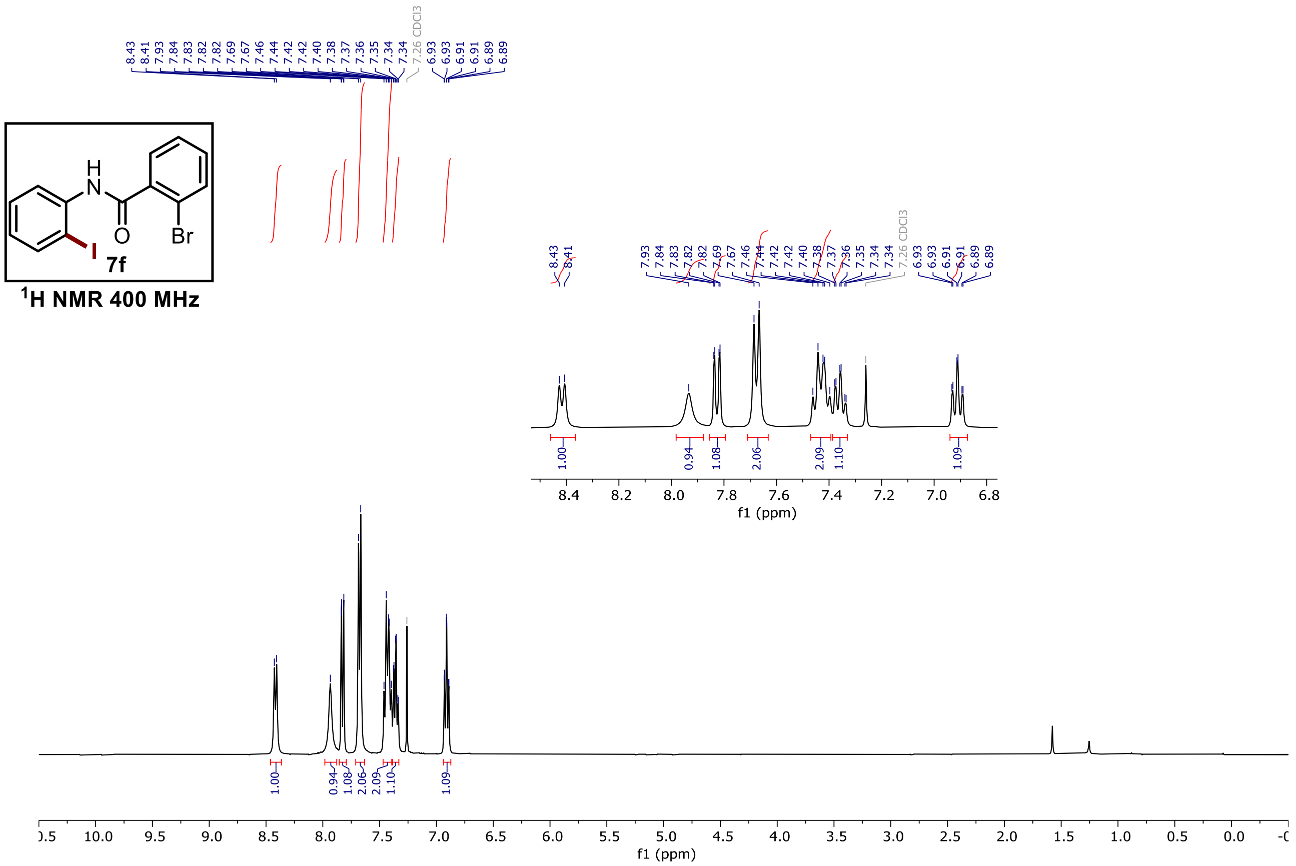
7f
Br
I
H
N
O
1H NMR 400 MHz
7.26 CDCl3
f1 (ppm)

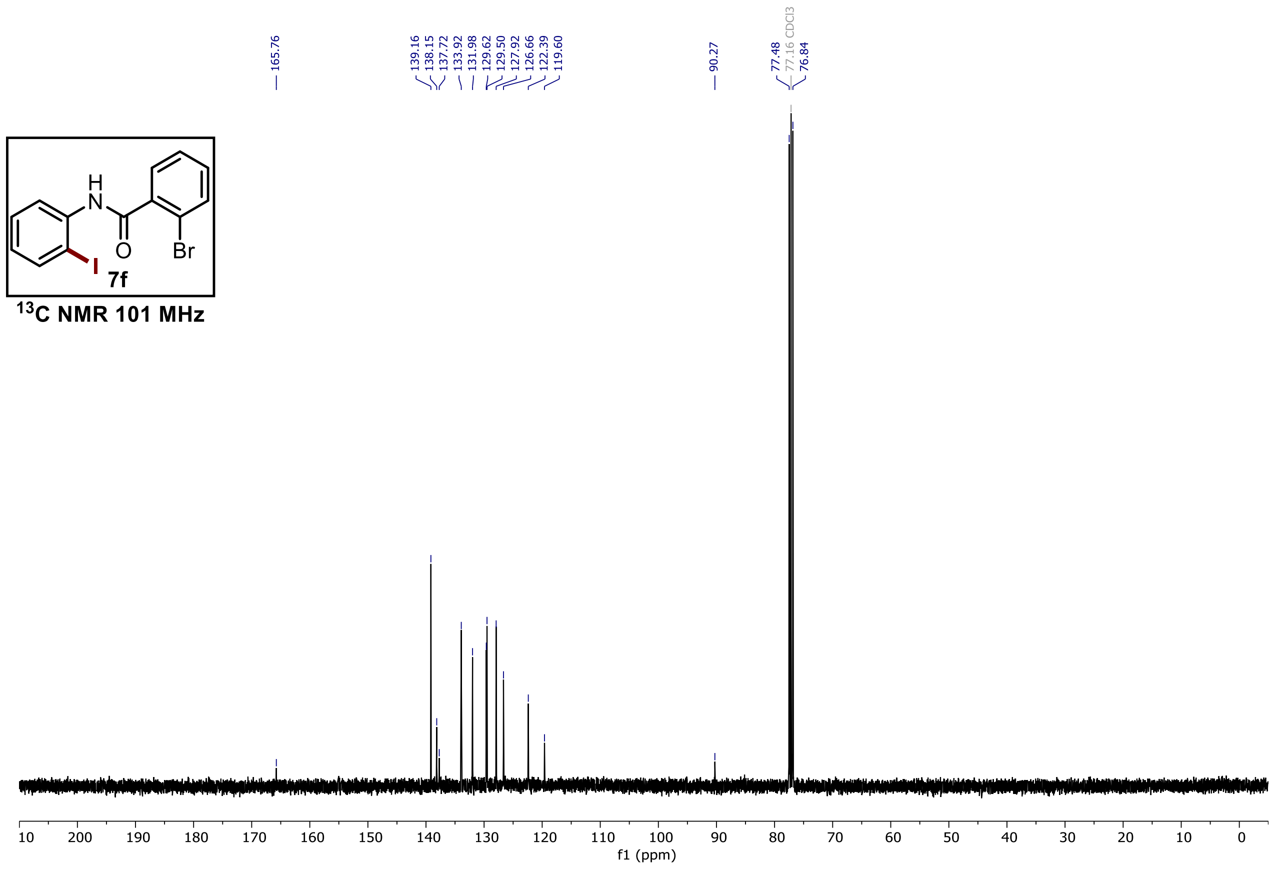
7f
Br
I
H
N
O
13C NMR 101 MHz
165.76
139.16
138.15
137.72
133.92
131.98
129.62
129.50
127.92
126.66
122.39
119.60
90.27
77.48
77.16 CDCl3
76.84
f1 (ppm)

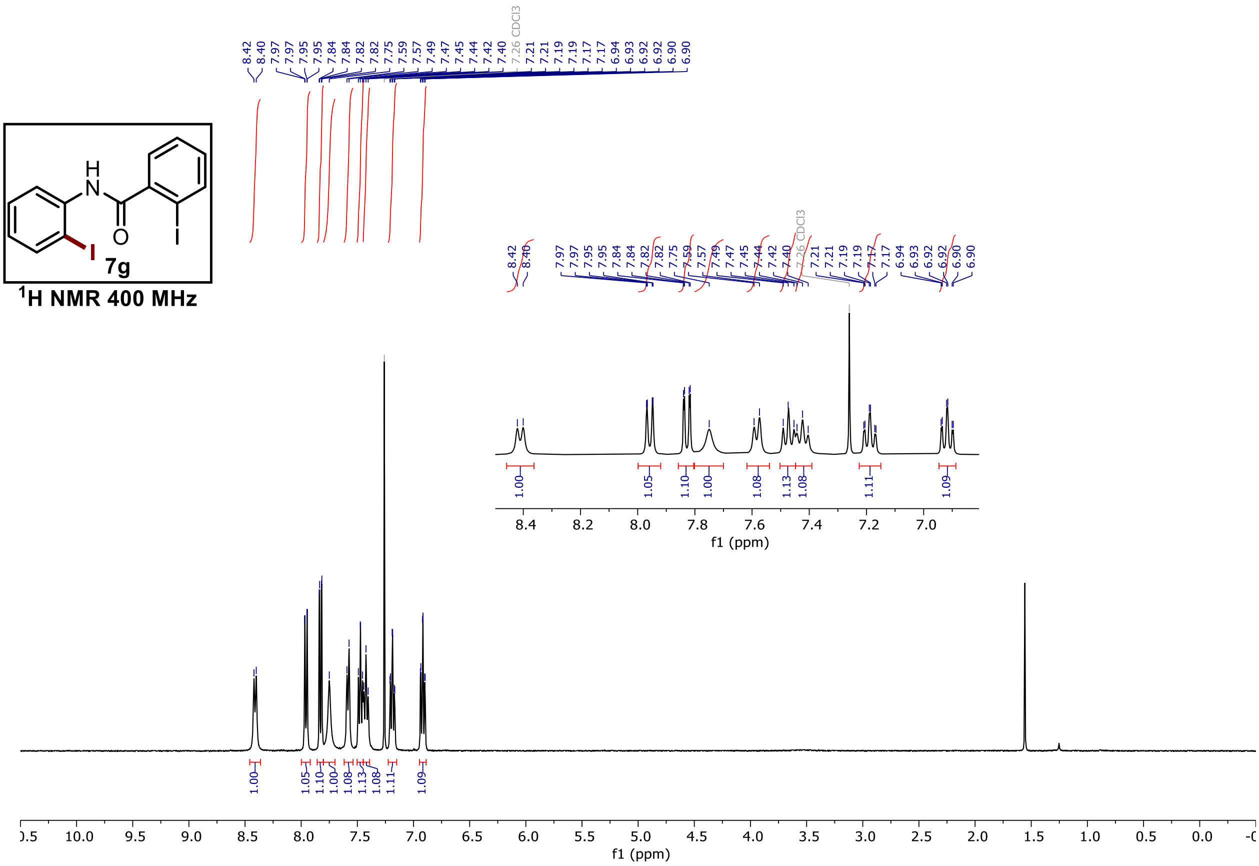
7g
1H NMR 400 MHz
f1 (ppm)

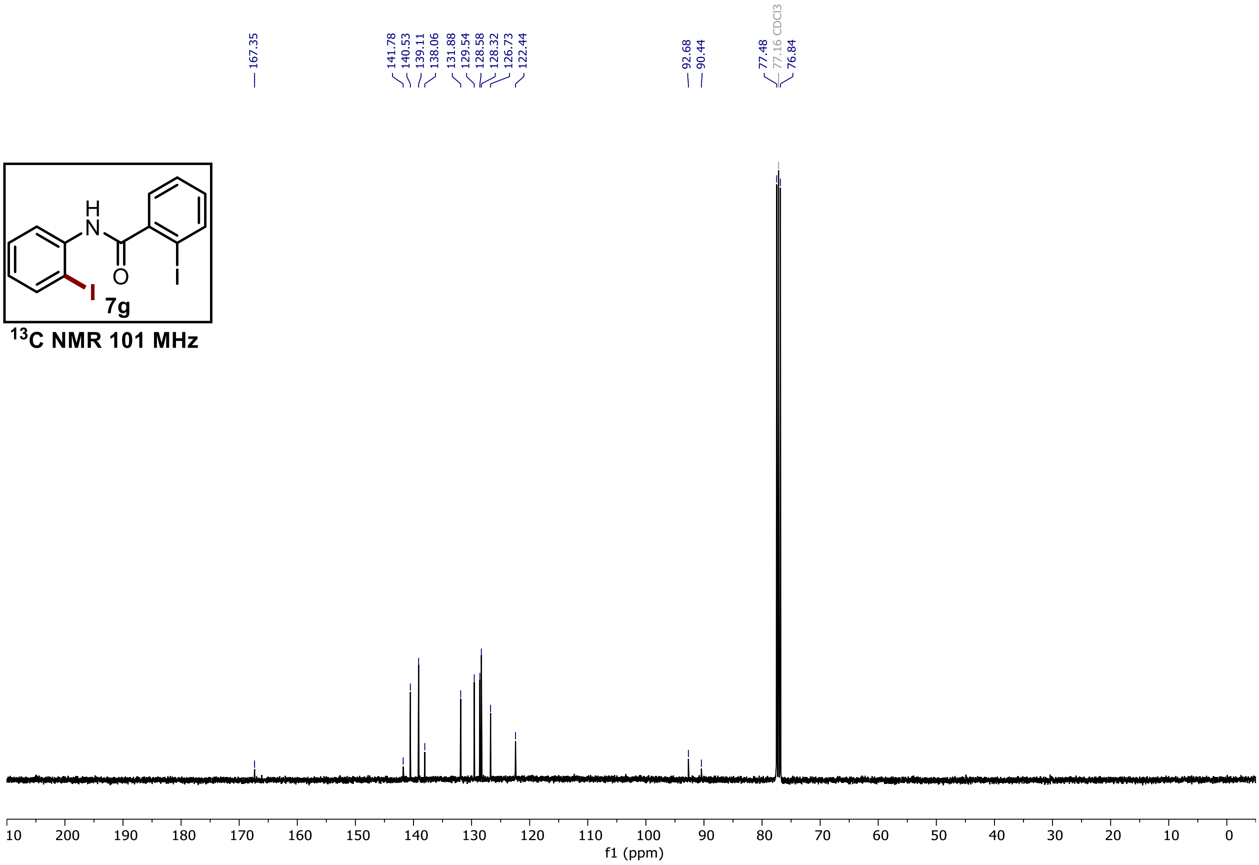
7g
13C NMR 101 MHz
167.35
141.78
140.53
139.11
138.06
131.88
129.54
128.58
128.32
126.73
122.44
92.68
90.44
77.48
77.16 CDCl3
76.84
f1 (ppm)

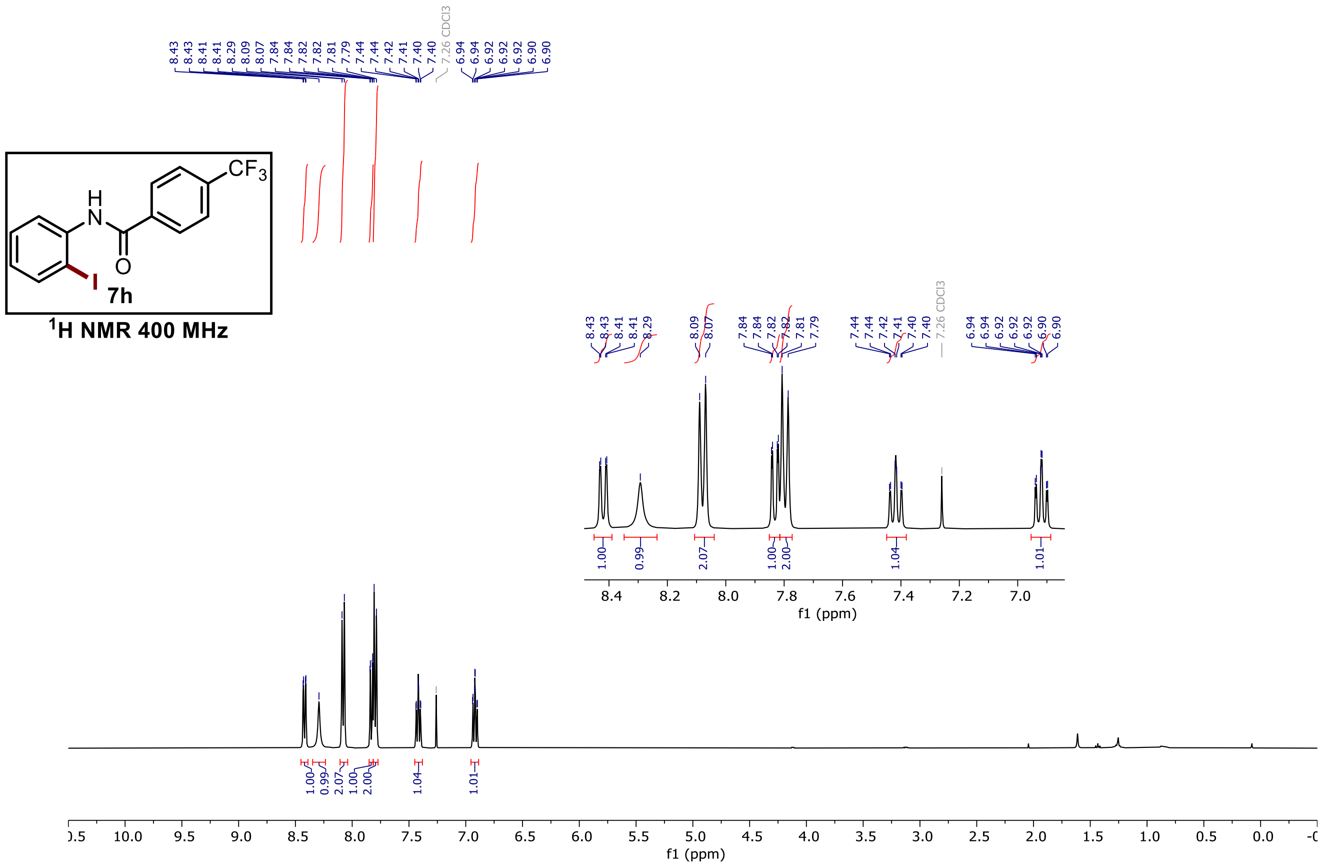

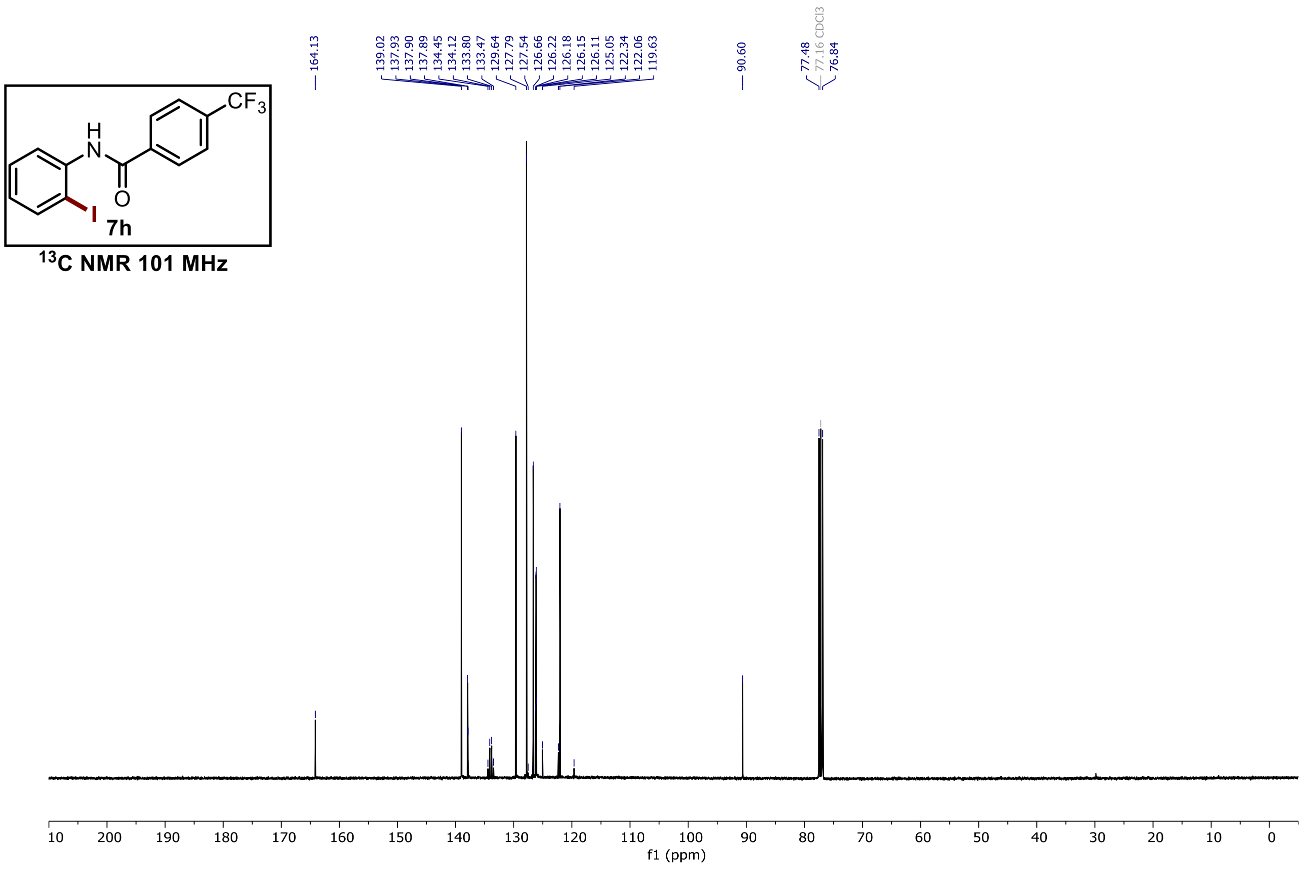

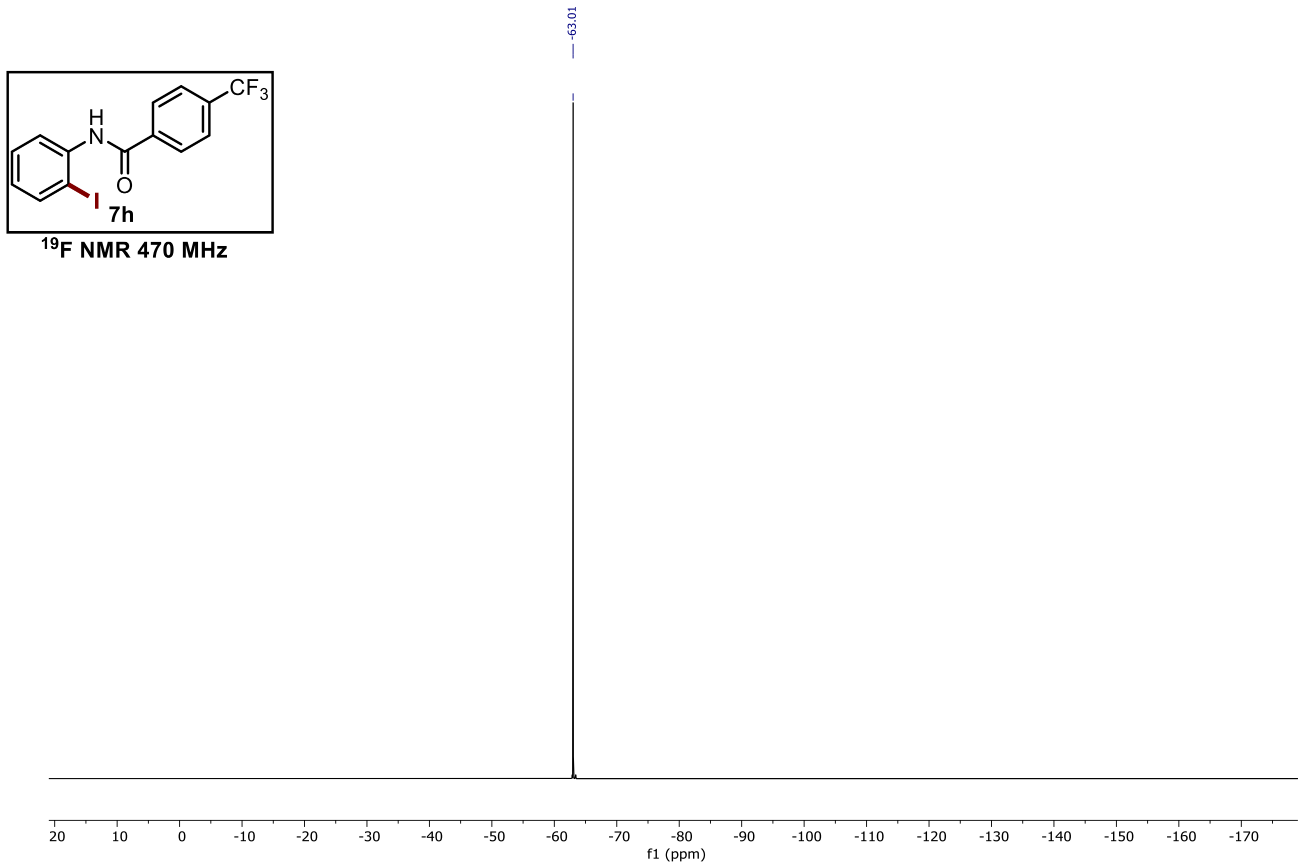

CF3
H
N
O
I
7h
19F NMR 470 MHz
-63.01
f1 (ppm)

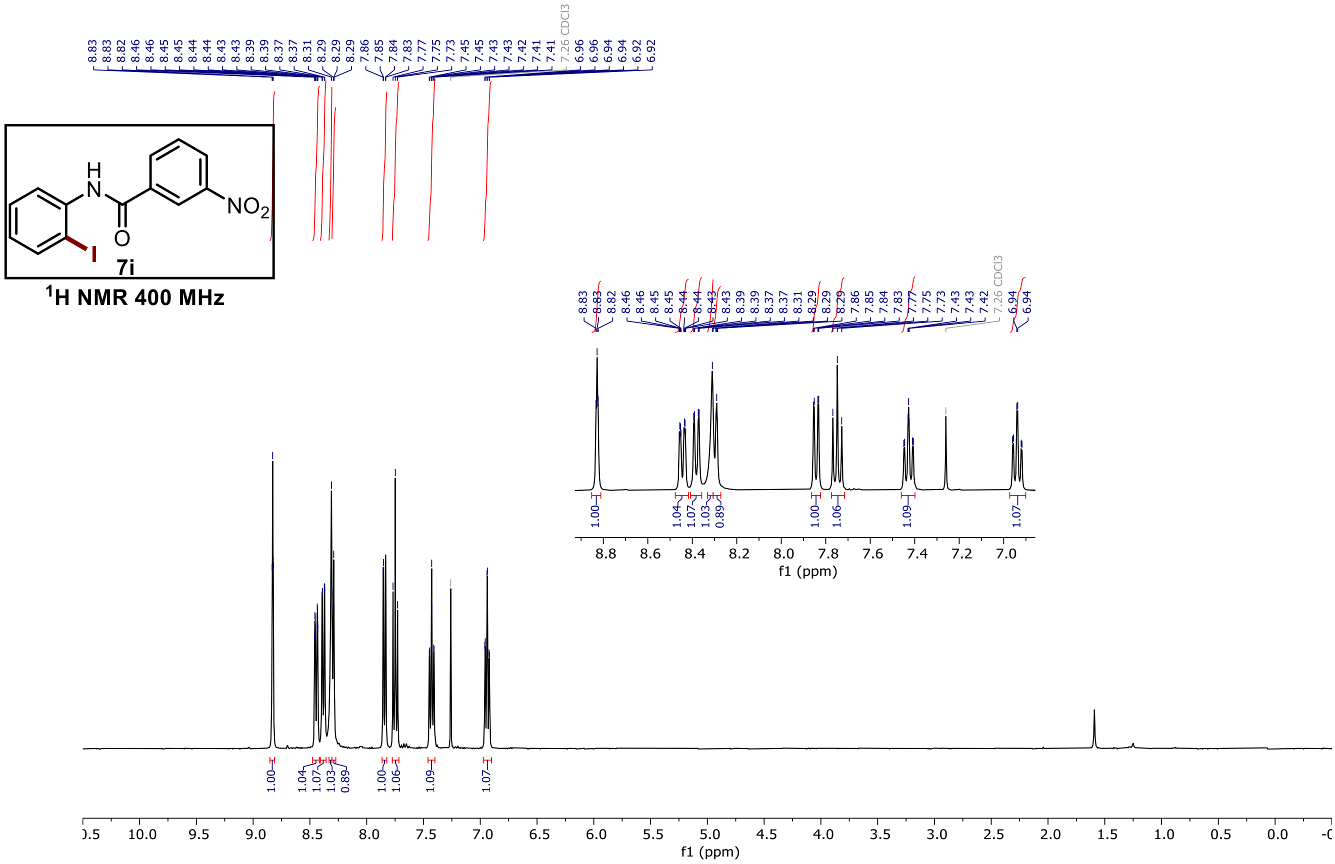

H
N
O
NO2
I
7i
1H NMR 400 MHz
7.26 CDCl3
f1 (ppm)

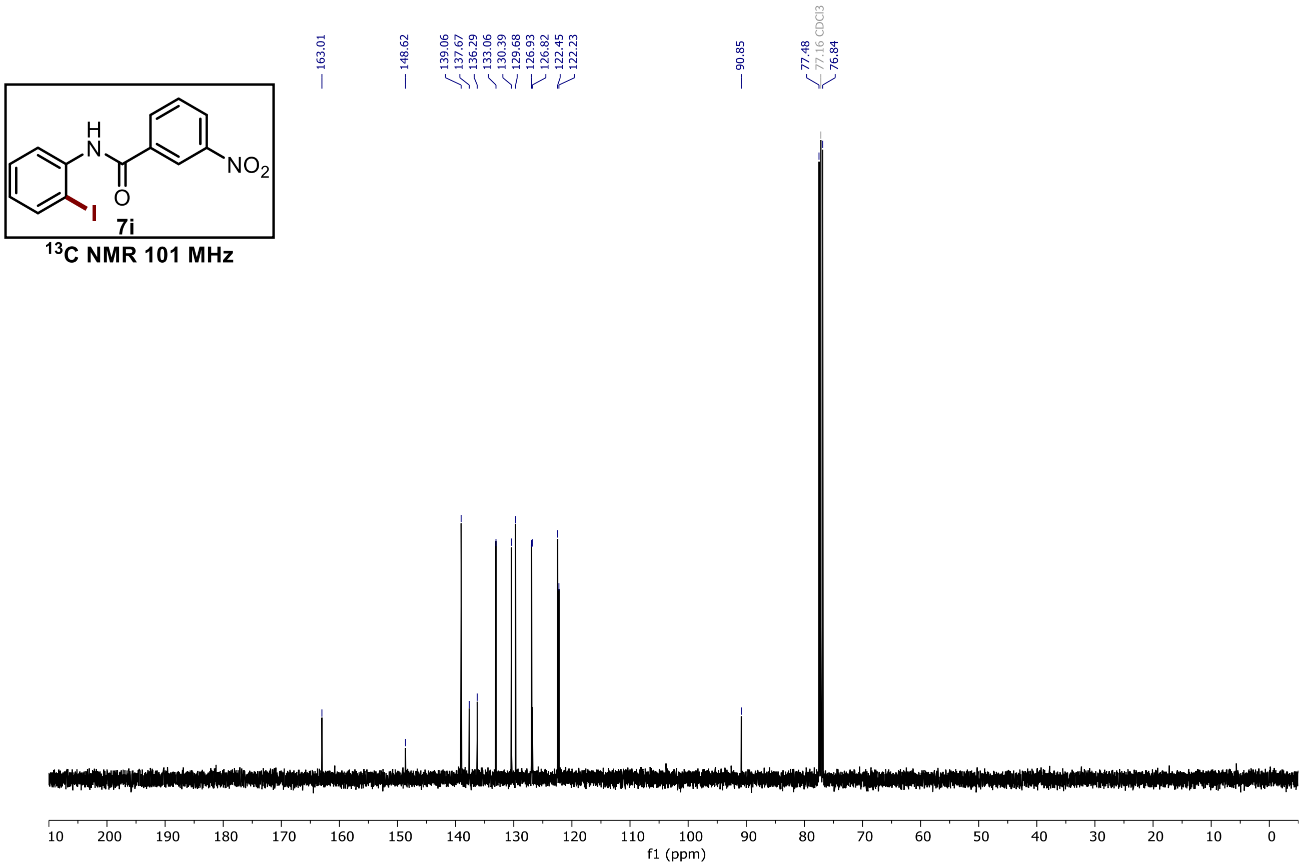
7i
NO2
13C NMR 101 MHz
163.01
148.62
139.06
137.67
136.29
133.06
130.39
129.68
126.93
126.82
122.45
122.23
90.85
77.48
77.16 CDCl3
76.84
f1 (ppm)

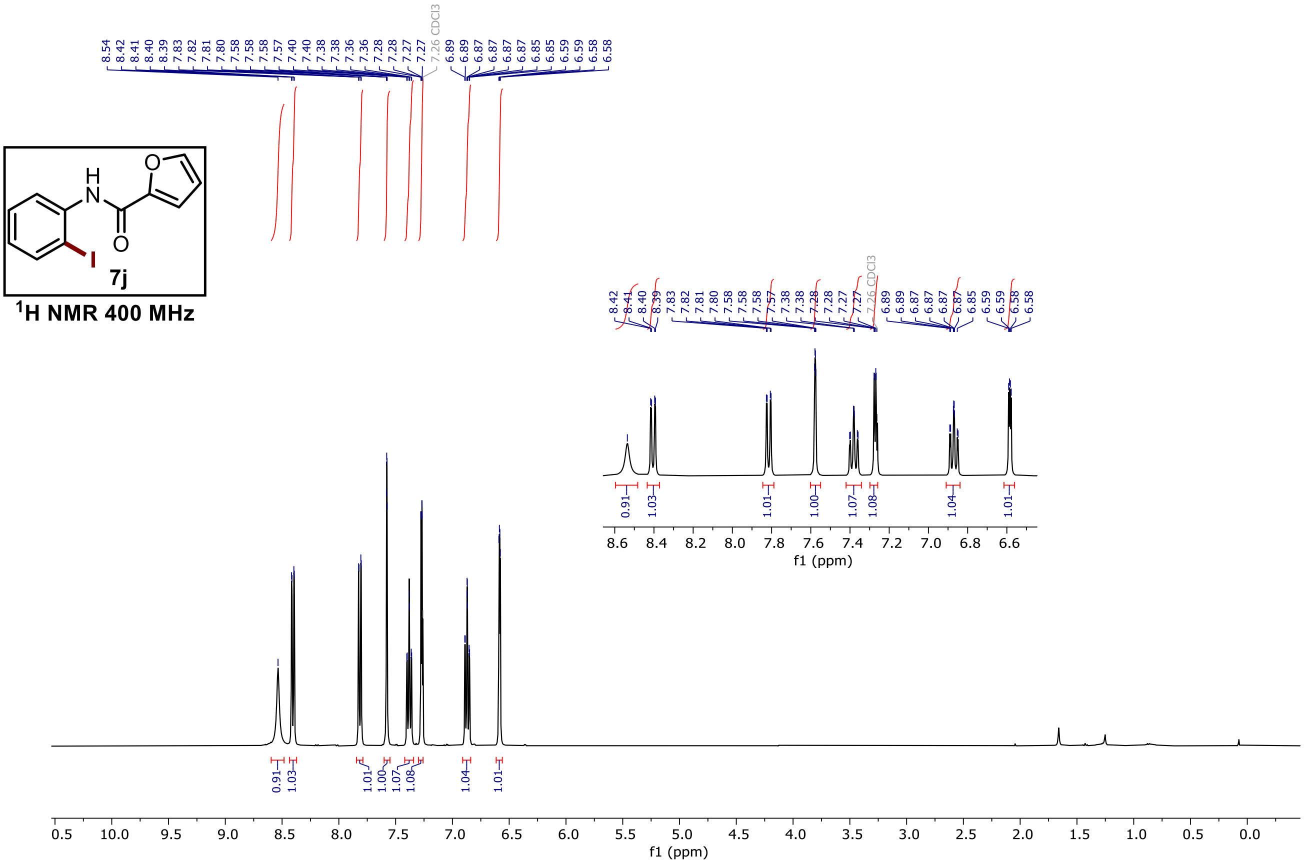
7j
1H NMR 400 MHz
8.54
8.42
8.41
8.40
8.39
7.83
7.82
7.81
7.80
7.58
7.58
7.58
7.57
7.40
7.40
7.38
7.38
7.36
7.36
7.28
7.28
7.27
7.27
7.26 CDCl3
6.89
6.89
6.87
6.87
6.87
6.87
6.85
6.85
6.59
6.59
6.58
6.58
0.91
1.03
1.01
1.00
1.07
1.08
1.04
1.01
f1 (ppm)

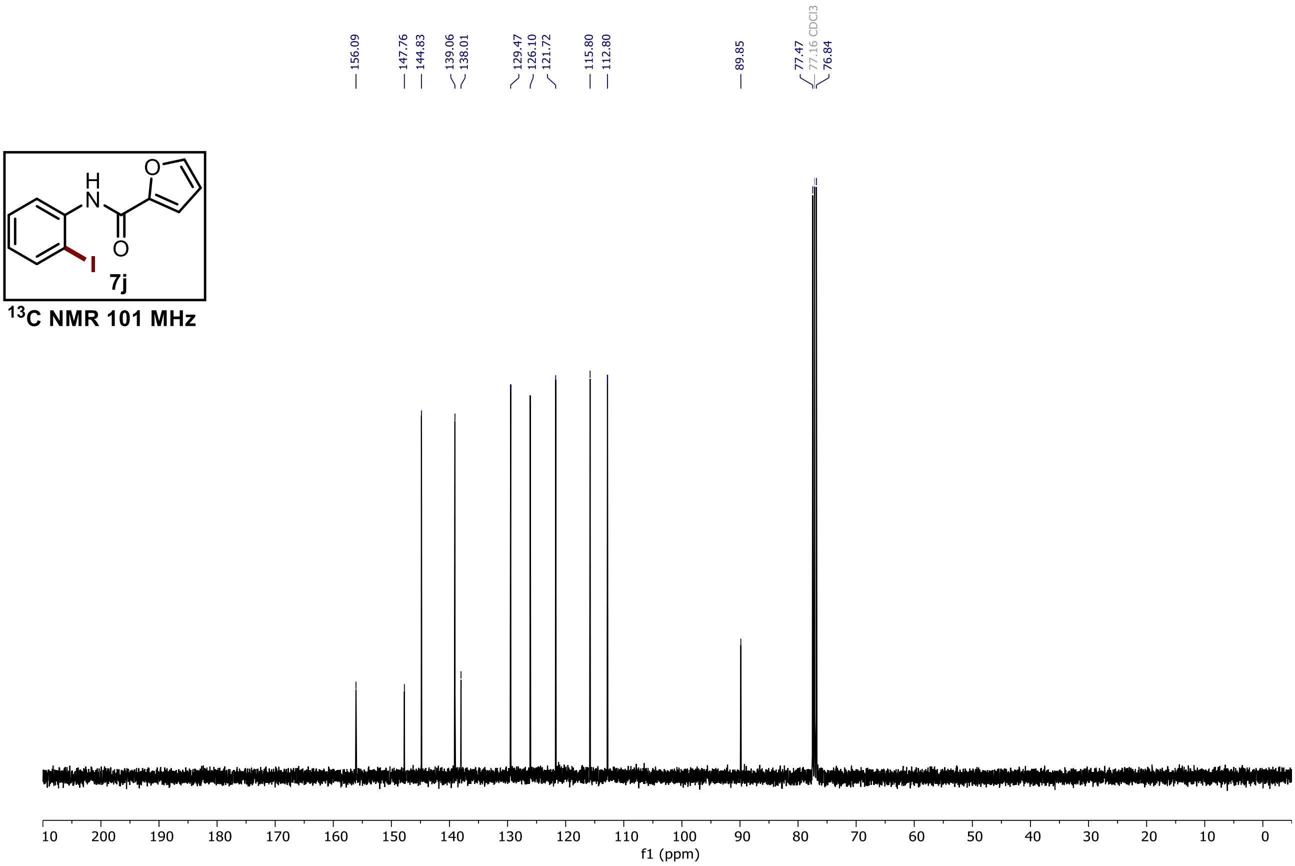
156.09
147.76
144.83
139.06
138.01
129.47
126.10
121.72
115.80
112.80
89.85
77.47
77.16 CDCl3
76.84
7j
^{13}C NMR 101 MHz
f1 (ppm)

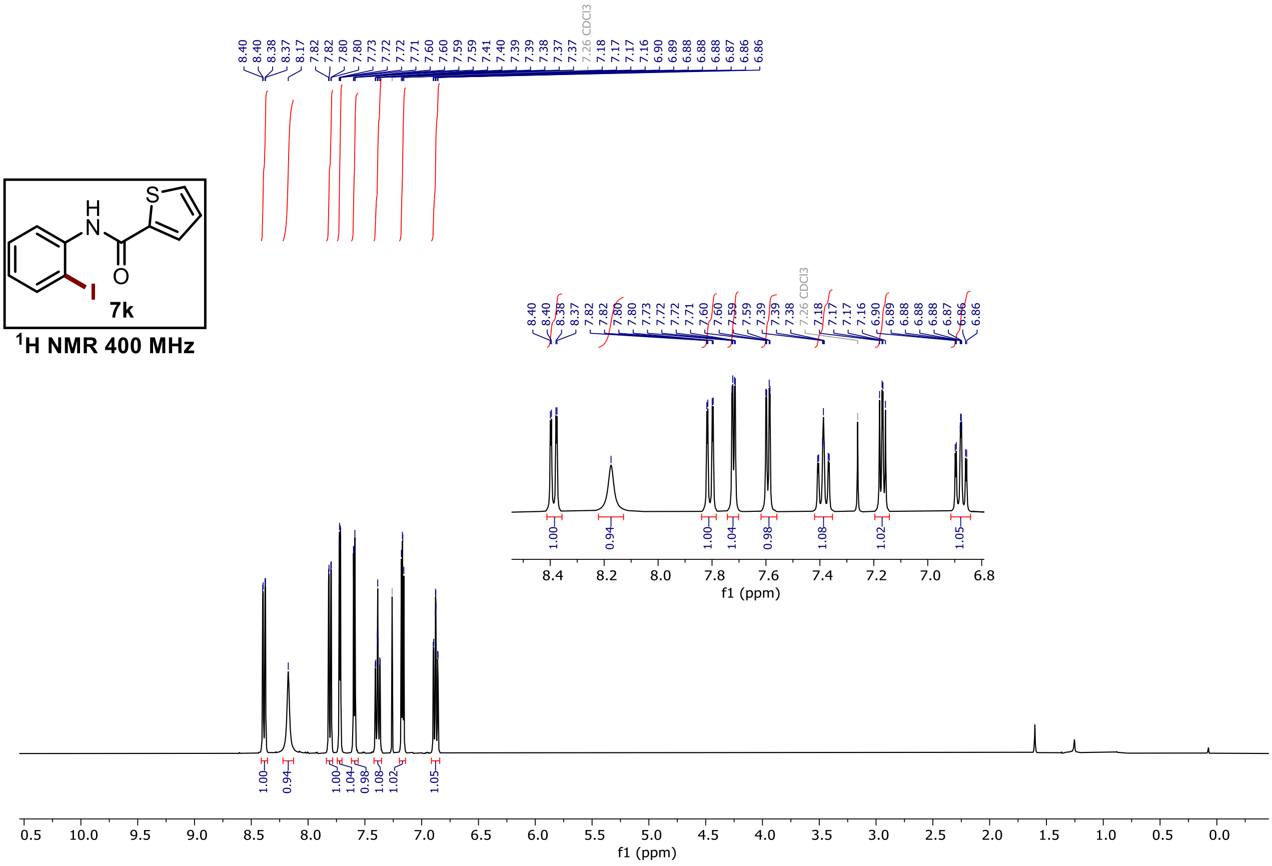
8.40
8.40
8.38
8.37
8.17
7.82
7.80
7.80
7.73
7.72
7.72
7.71
7.60
7.60
7.59
7.59
7.41
7.40
7.39
7.39
7.38
7.37
7.37
7.26 CDCl3
7.18
7.17
7.17
7.16
6.90
6.89
6.88
6.88
6.88
6.87
6.86
6.86
7k
^{1}H NMR 400 MHz
1.00
0.94
1.00
1.04
0.98
1.08
1.02
1.05
f1 (ppm)

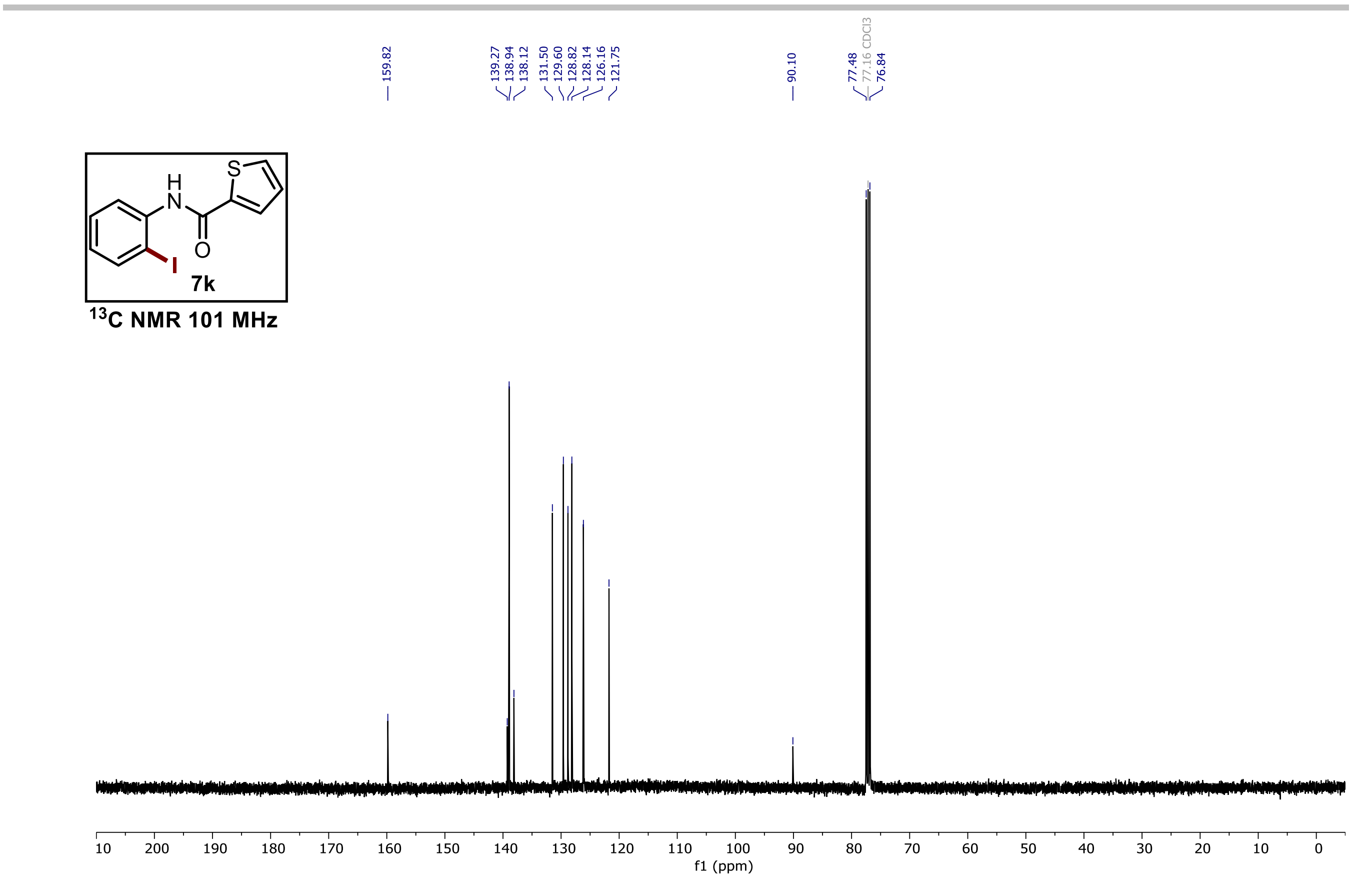
7k
13C NMR 101 MHz
159.82
139.27
138.94
138.12
131.50
129.60
128.82
128.14
126.16
121.75
90.10
77.48
77.16 CDCl3
76.84
f1 (ppm)

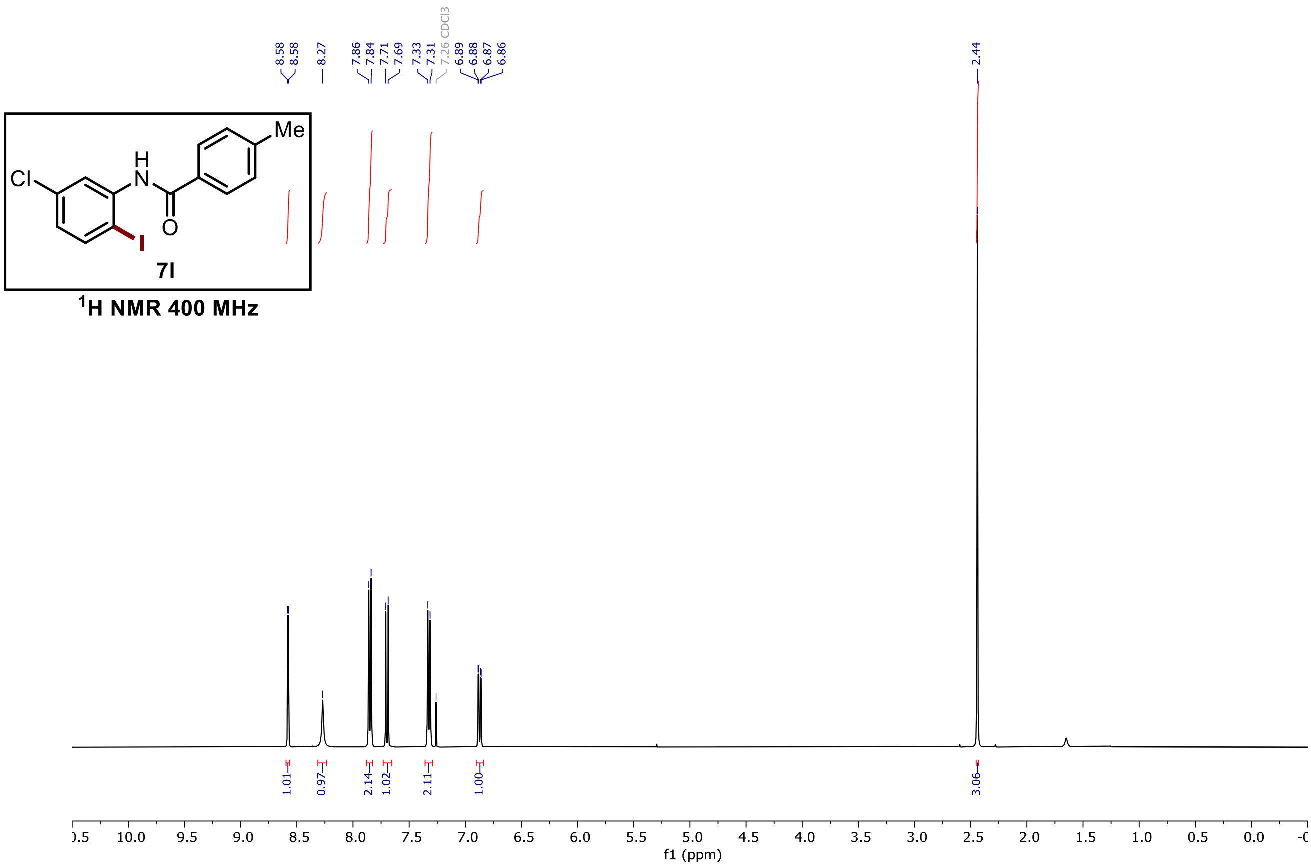
7l
1H NMR 400 MHz
8.58
8.58
8.27
7.86
7.84
7.71
7.69
7.33
7.31
7.26 CDCl3
6.89
6.88
6.87
6.86
2.44
1.01
0.97
2.14
1.02
2.11
1.00
3.06
f1 (ppm)

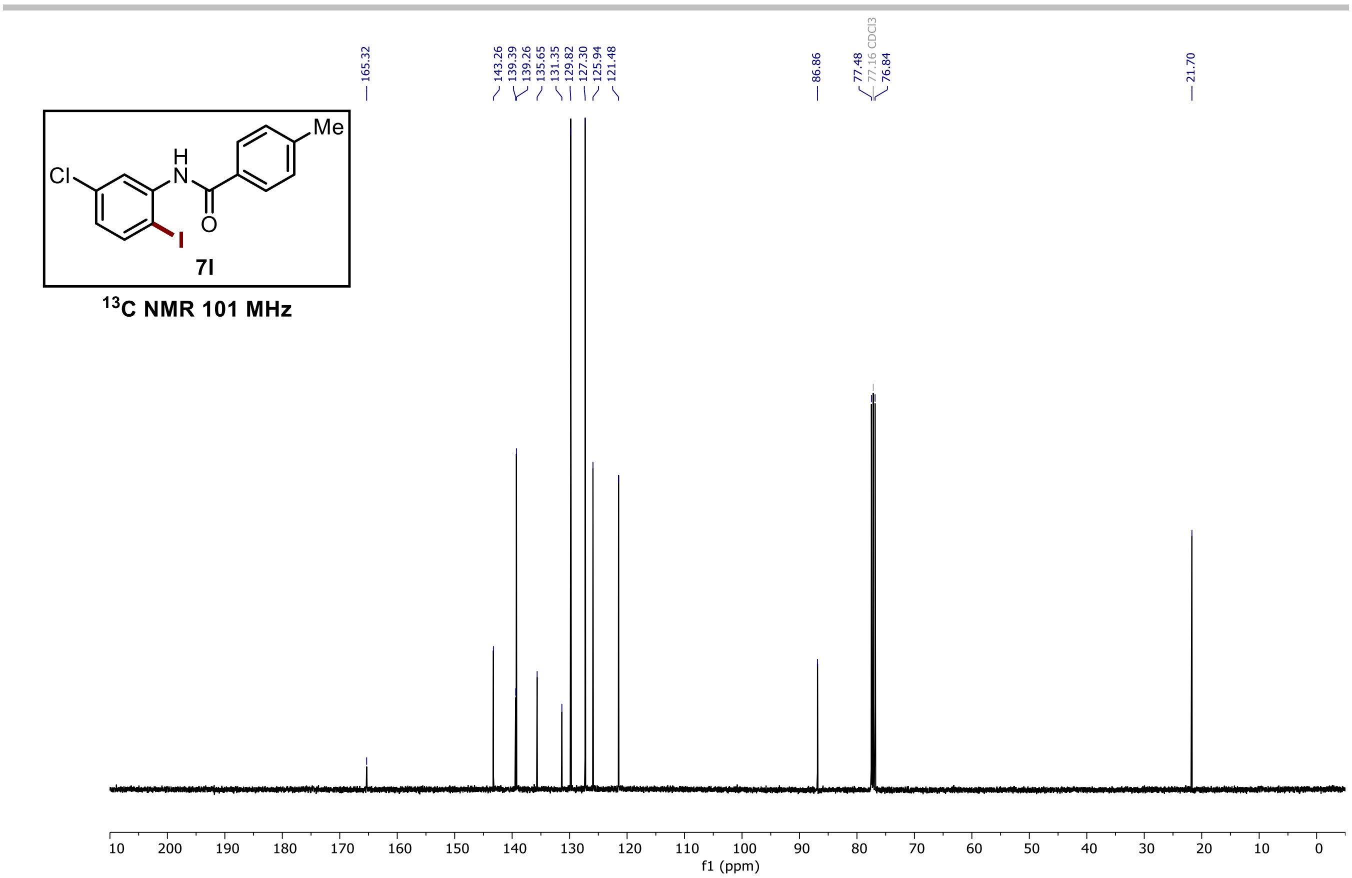

165.32
143.26
139.39
139.26
135.65
131.35
129.82
127.30
125.94
121.48
86.86
77.48
77.16 CDCl3
76.84
21.70
Cl
H
N
O
Me
I
7l
13C NMR 101 MHz
f1 (ppm)

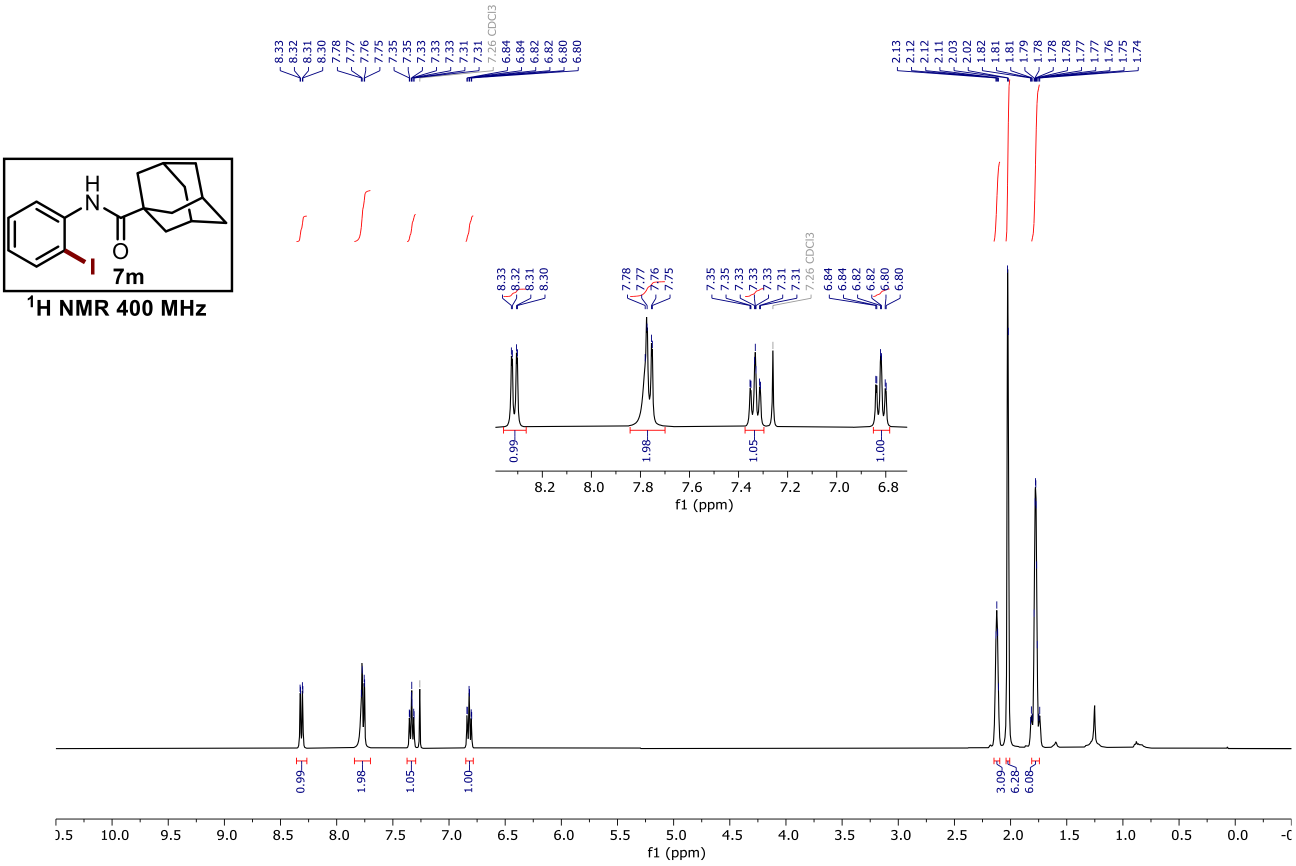

8.33
8.32
8.31
8.30
7.78
7.77
7.76
7.75
7.35
7.35
7.33
7.33
7.33
7.31
7.31
7.26 CDCl3
6.84
6.84
6.82
6.82
6.80
6.80
2.13
2.12
2.12
2.11
2.03
2.02
1.82
1.81
1.81
1.79
1.78
1.78
1.77
1.77
1.76
1.75
1.74
H
N
O
I
7m
1H NMR 400 MHz
0.99
1.98
1.05
1.00
3.09
6.28
6.08
f1 (ppm)

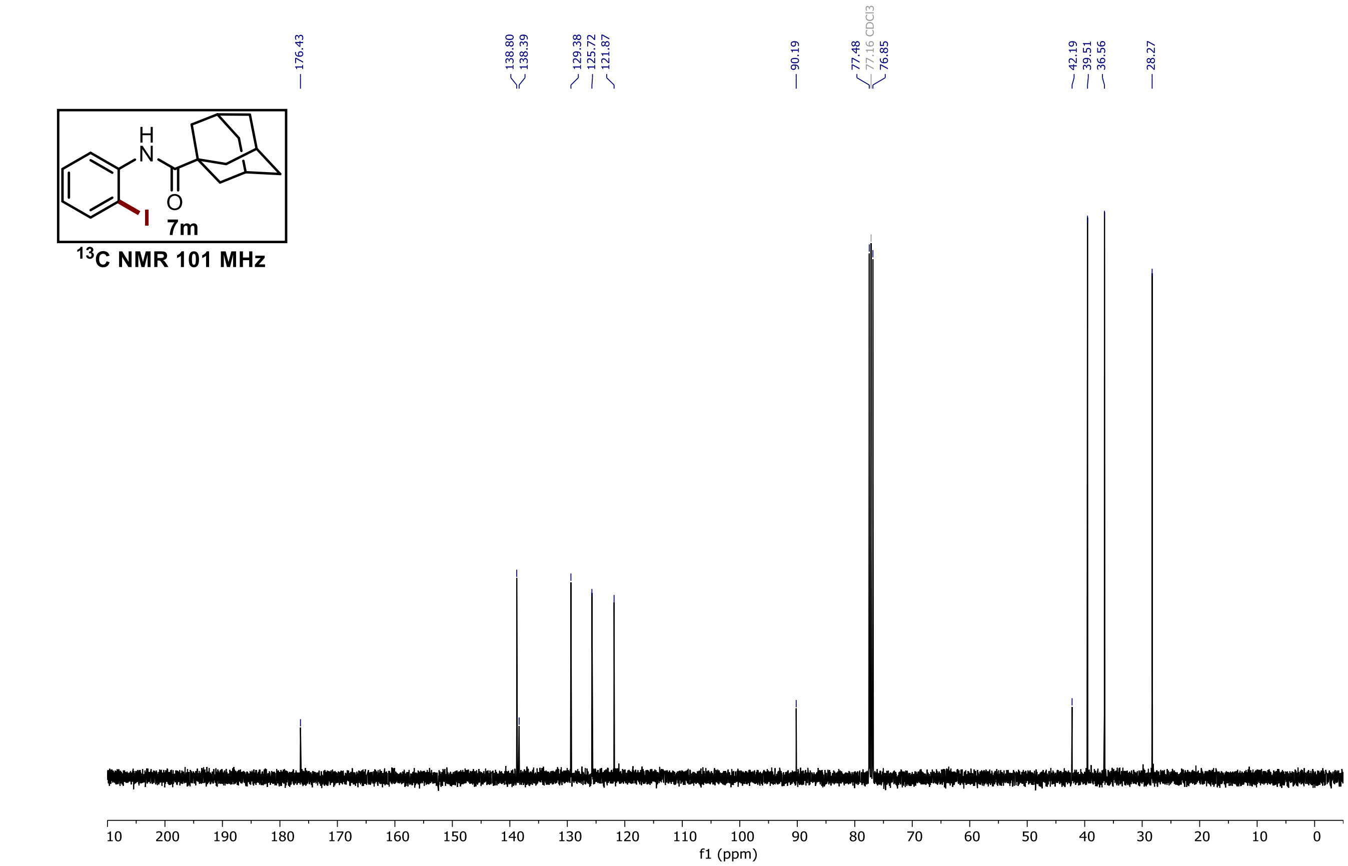
7m
^{13}C NMR 101 MHz
176.43
138.80
138.39
129.38
125.72
121.87
90.19
77.48
77.16 CDCl3
76.85
42.19
39.51
36.56
28.27
f1 (ppm)

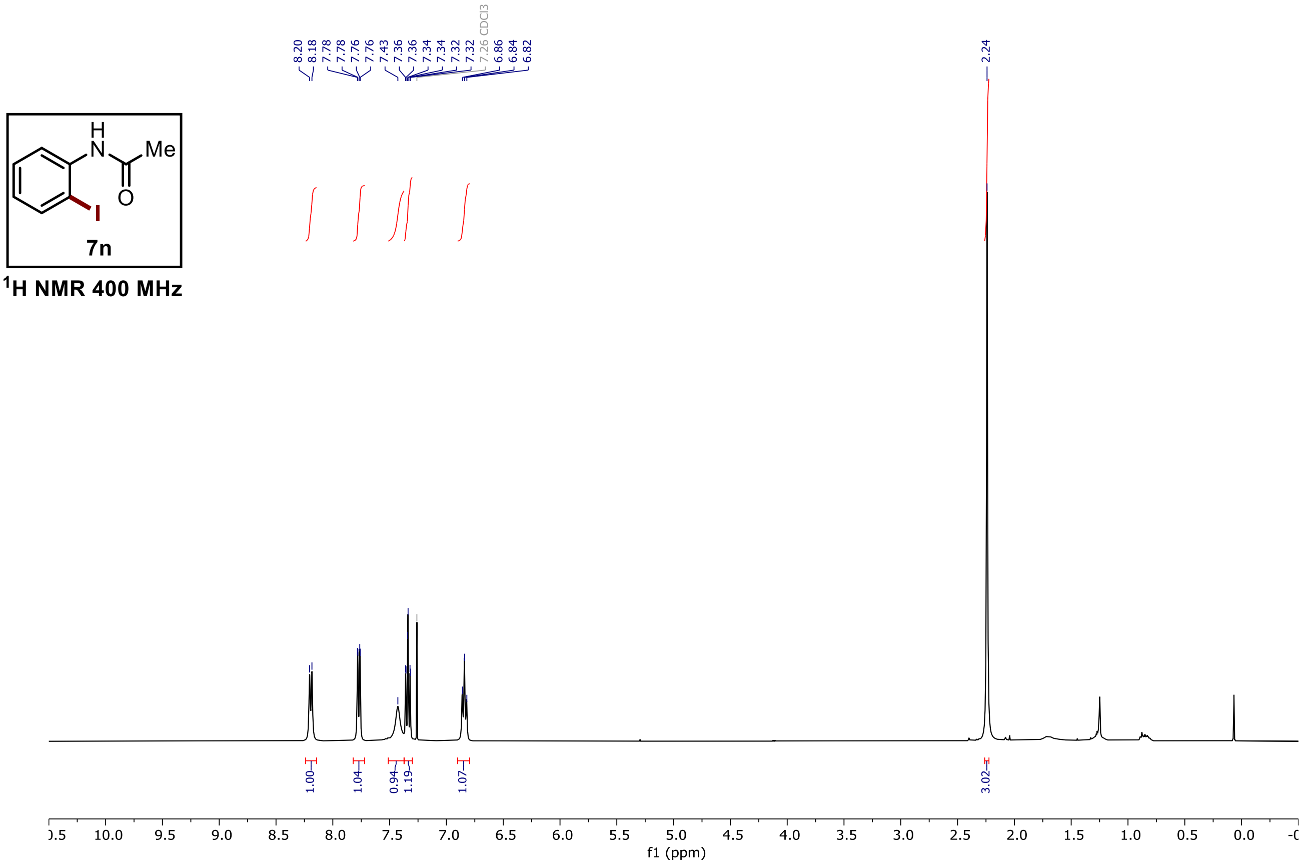
7n
^{1}H NMR 400 MHz
8.20
8.18
7.78
7.78
7.76
7.76
7.43
7.36
7.36
7.34
7.34
7.32
7.32
7.26 CDCl3
6.86
6.84
6.82
2.24
1.00
1.04
0.94
1.19
1.07
3.02
f1 (ppm)

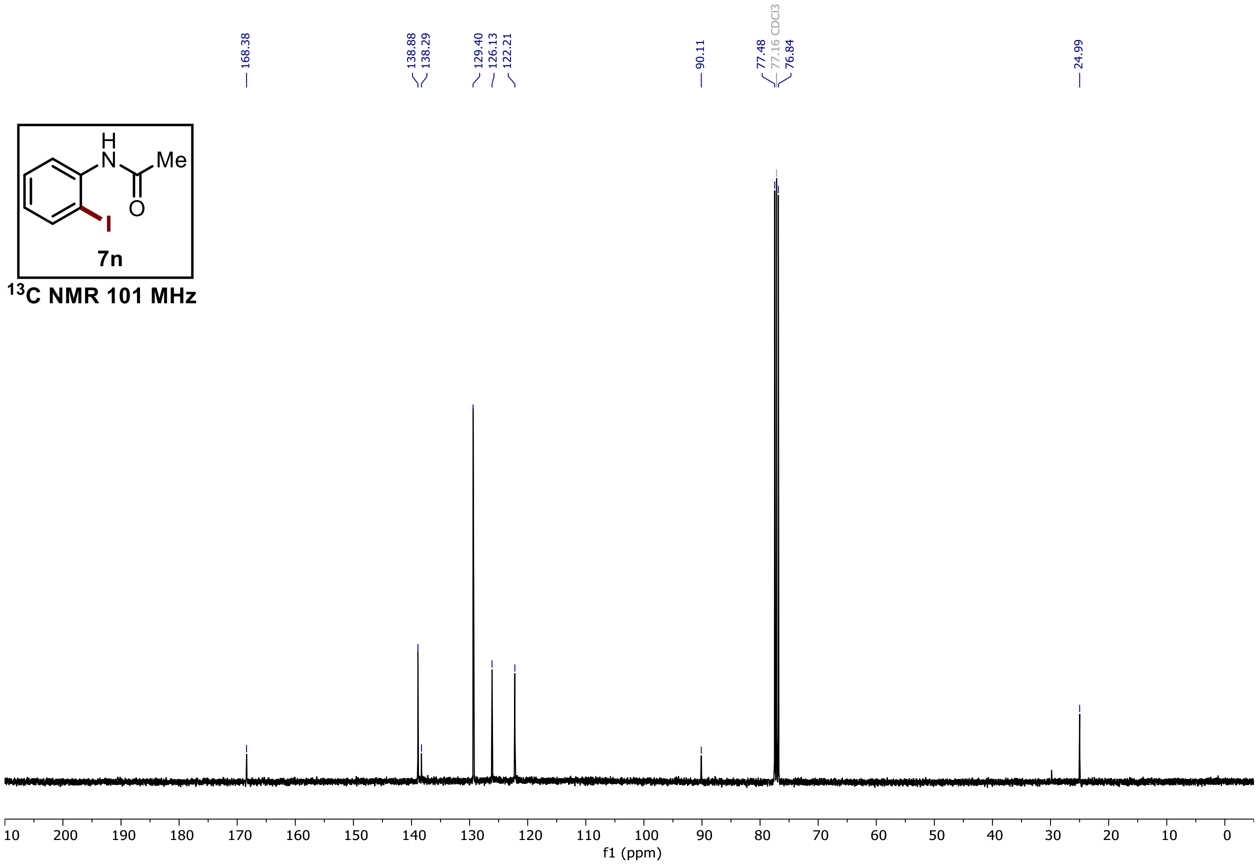
168.38
138.88
138.29
129.40
126.13
122.21
90.11
77.48
77.16 CDCl3
76.84
24.99
7n
13C NMR 101 MHz
f1 (ppm)

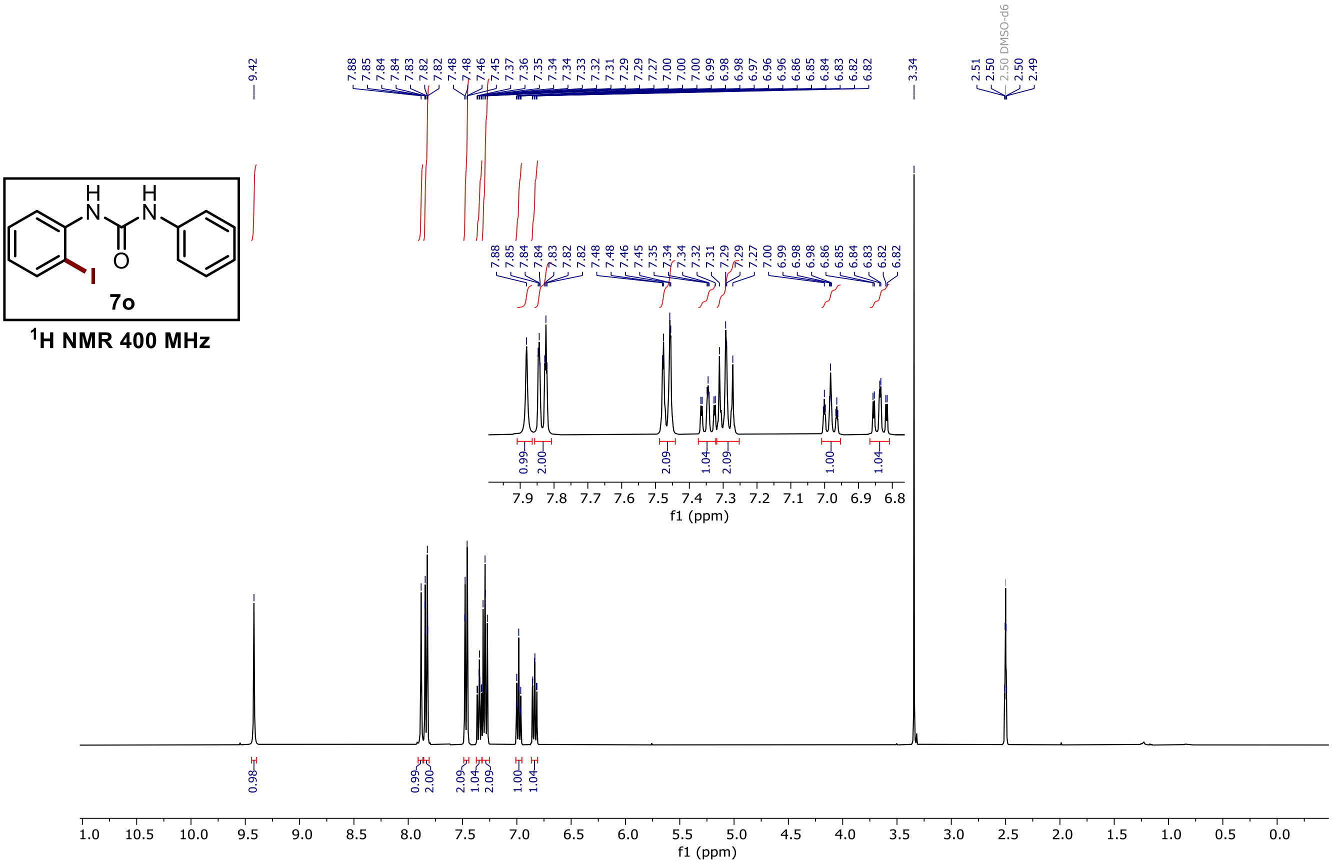
9.42
3.34
2.50 DMSO-d6
7o
1H NMR 400 MHz
0.98
0.99
2.00
2.09
1.04
2.09
1.00
1.04
f1 (ppm)

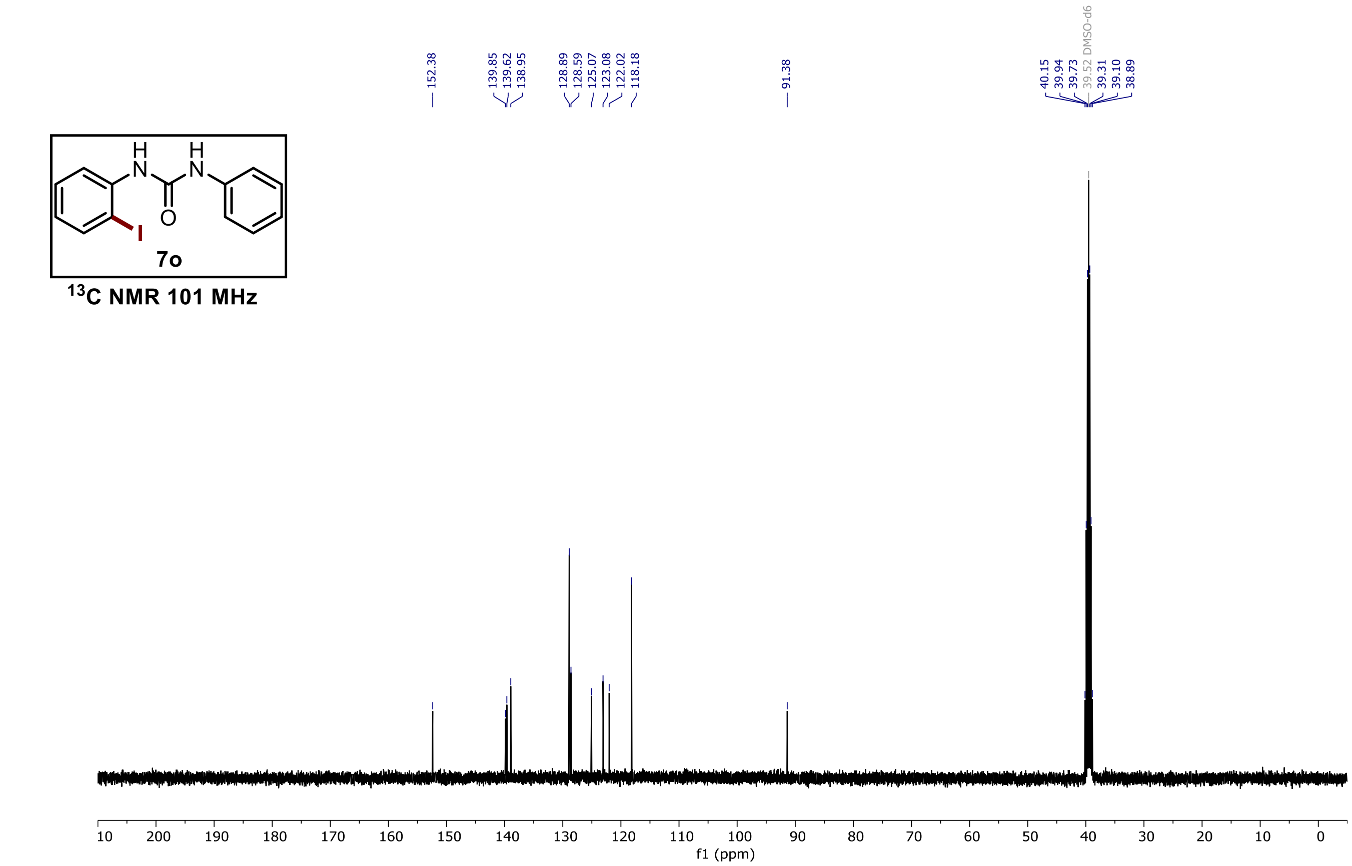
152.38
139.85
139.62
138.95
128.89
128.59
125.07
123.08
122.02
118.18
91.38
40.15
39.94
39.73
39.52 DMSO-d6
39.31
39.10
38.89
7o
13C NMR 101 MHz
f1 (ppm)

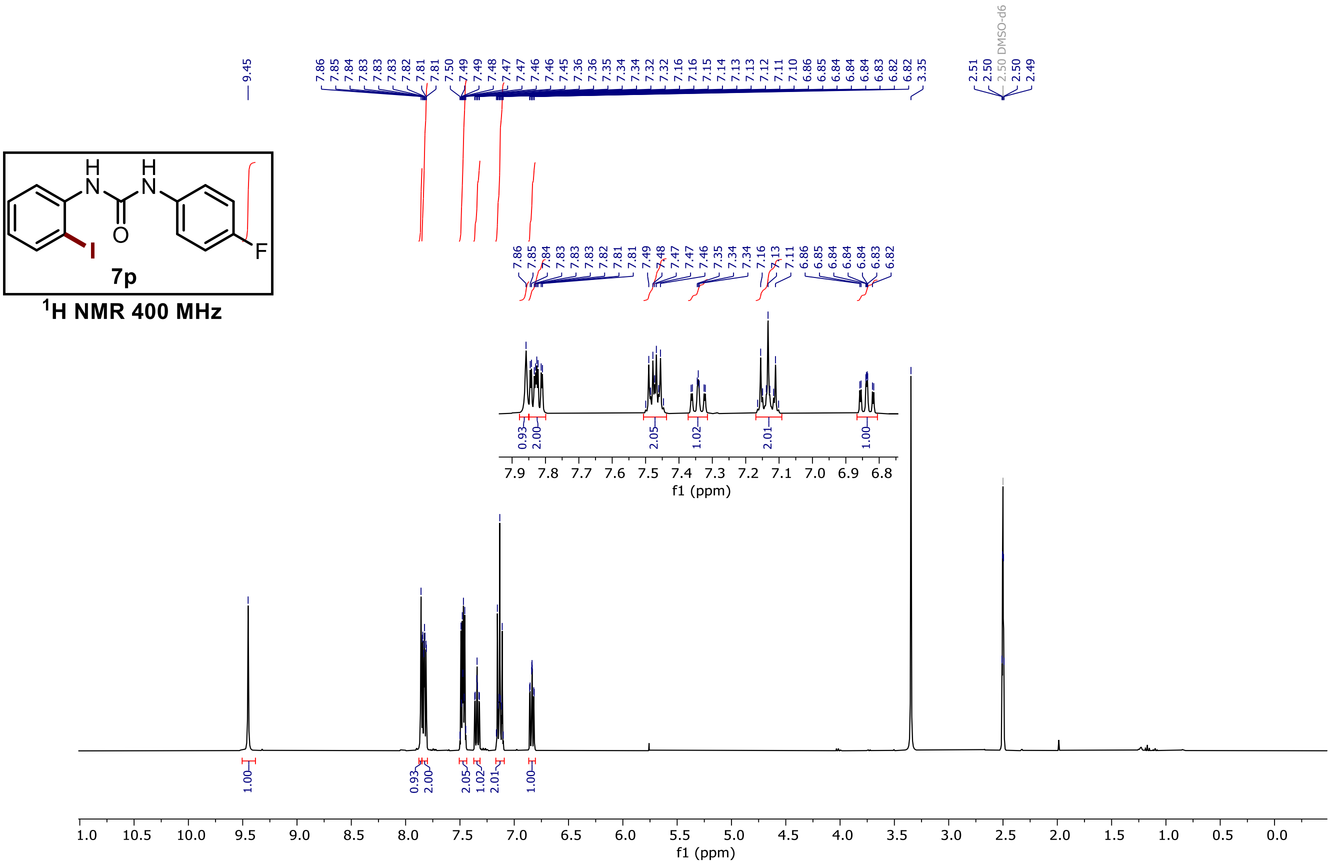
9.45
7.86
7.85
7.84
7.83
7.83
7.83
7.82
7.81
7.81
7.50
7.49
7.49
7.48
7.47
7.47
7.46
7.46
7.45
7.36
7.36
7.35
7.34
7.34
7.32
7.32
7.16
7.15
7.14
7.13
7.13
7.12
7.11
7.10
6.86
6.85
6.84
6.84
6.84
6.83
6.82
6.82
3.35
2.51
2.50
2.50 DMSO-d6
2.50
2.49
7p
1H NMR 400 MHz
f1 (ppm)

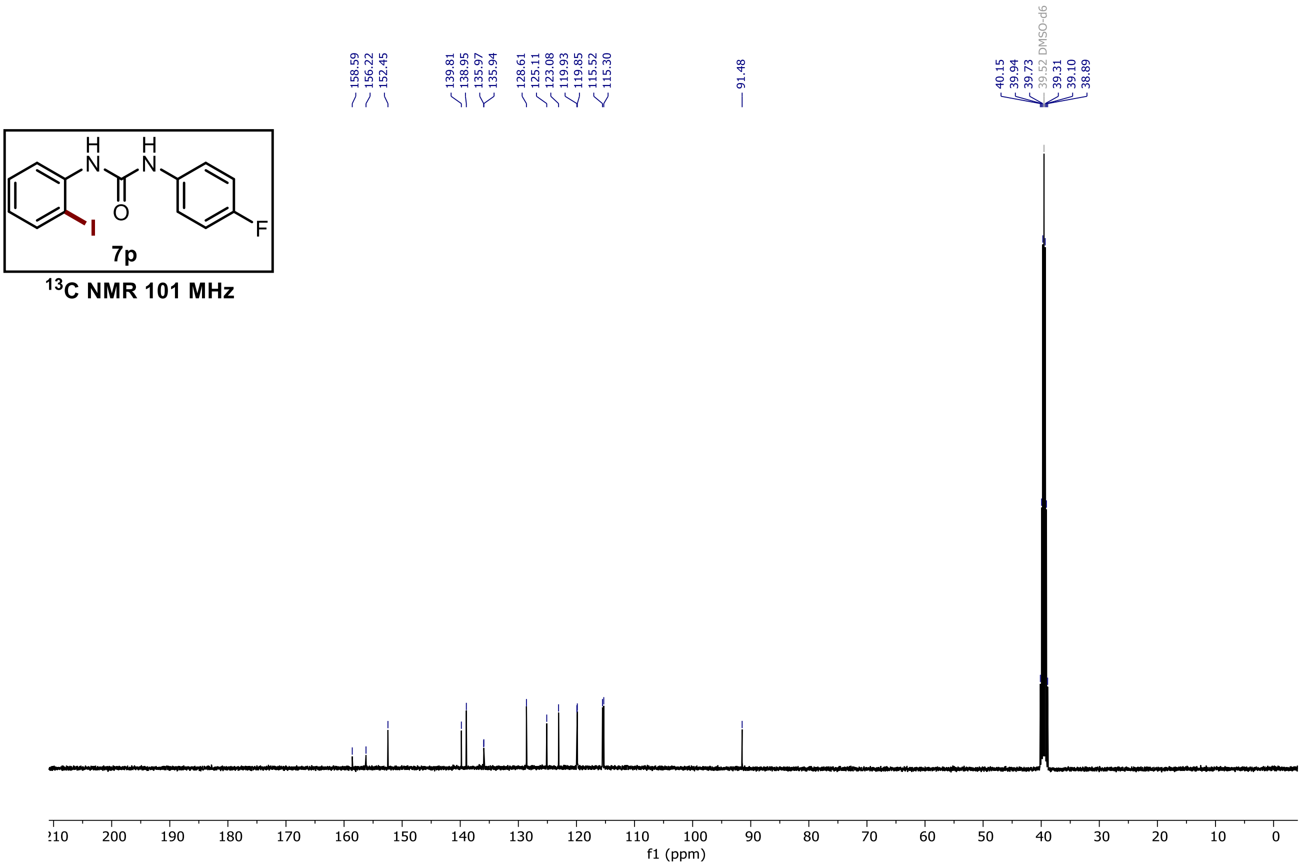

158.59
156.22
152.45
139.81
138.95
135.97
135.94
128.61
125.11
123.08
119.93
119.85
115.52
115.30
91.48
40.15
39.94
39.73
39.52 DMSO-d6
39.31
39.10
38.89
7p
13C NMR 101 MHz
f1 (ppm)

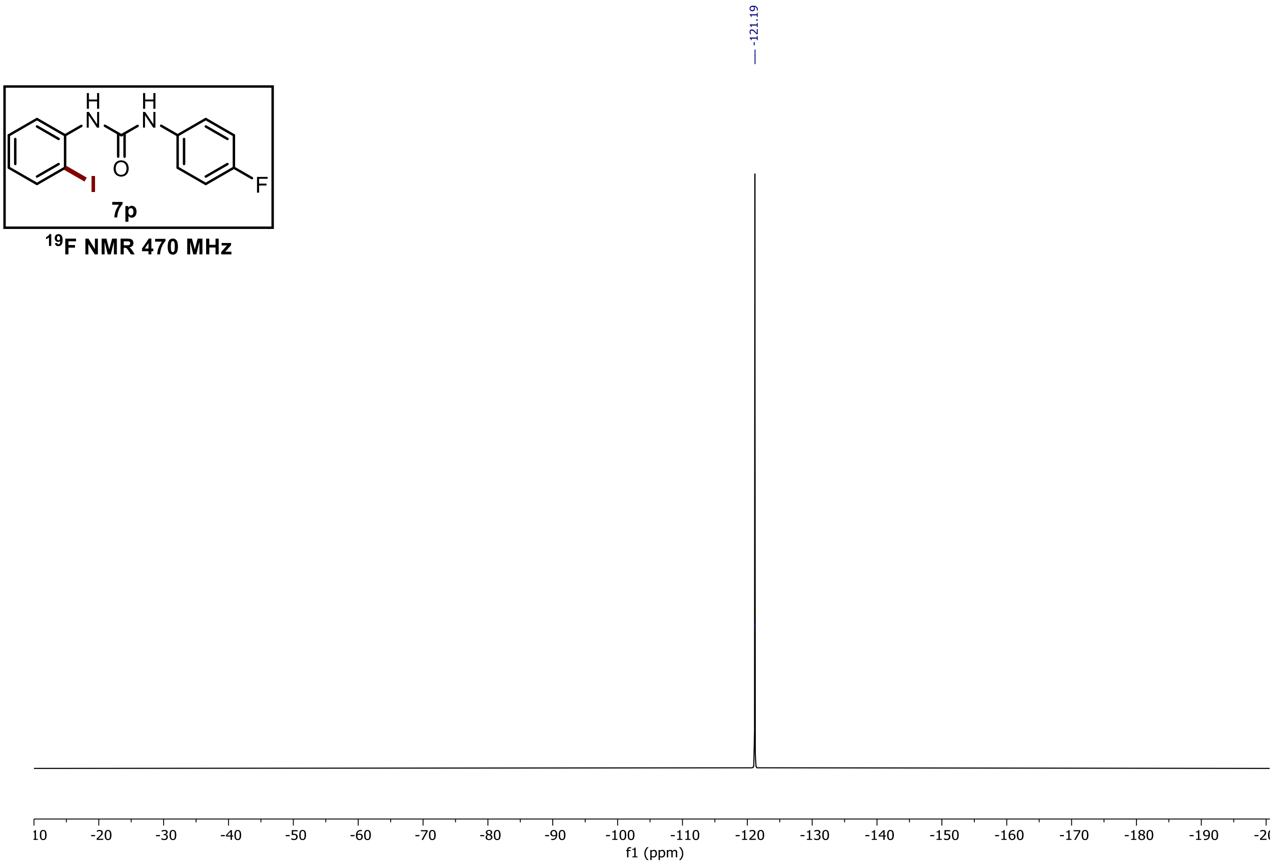

-121.19
7p
19F NMR 470 MHz
f1 (ppm)

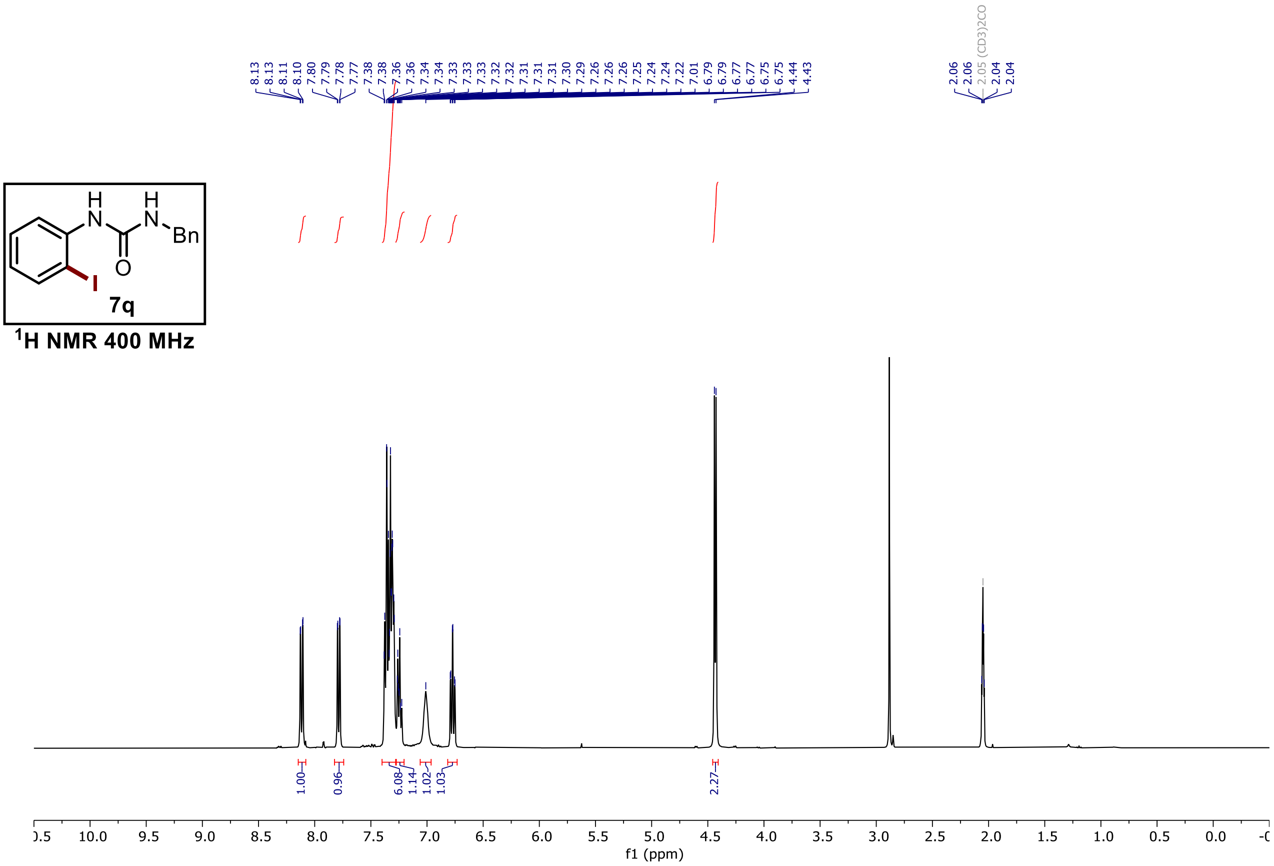
7q
^{1}H NMR 400 MHz
f1 (ppm)

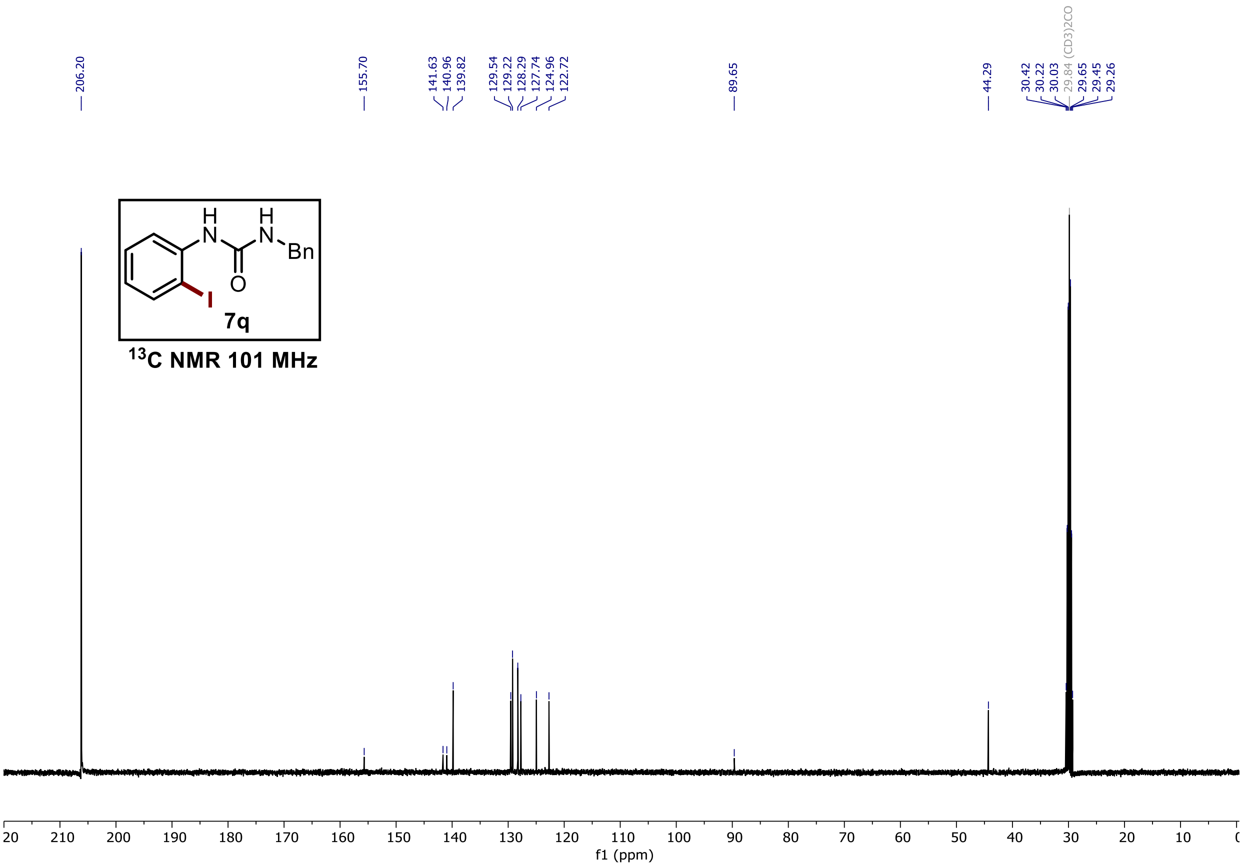
7q
^{13}C NMR 101 MHz
f1 (ppm)

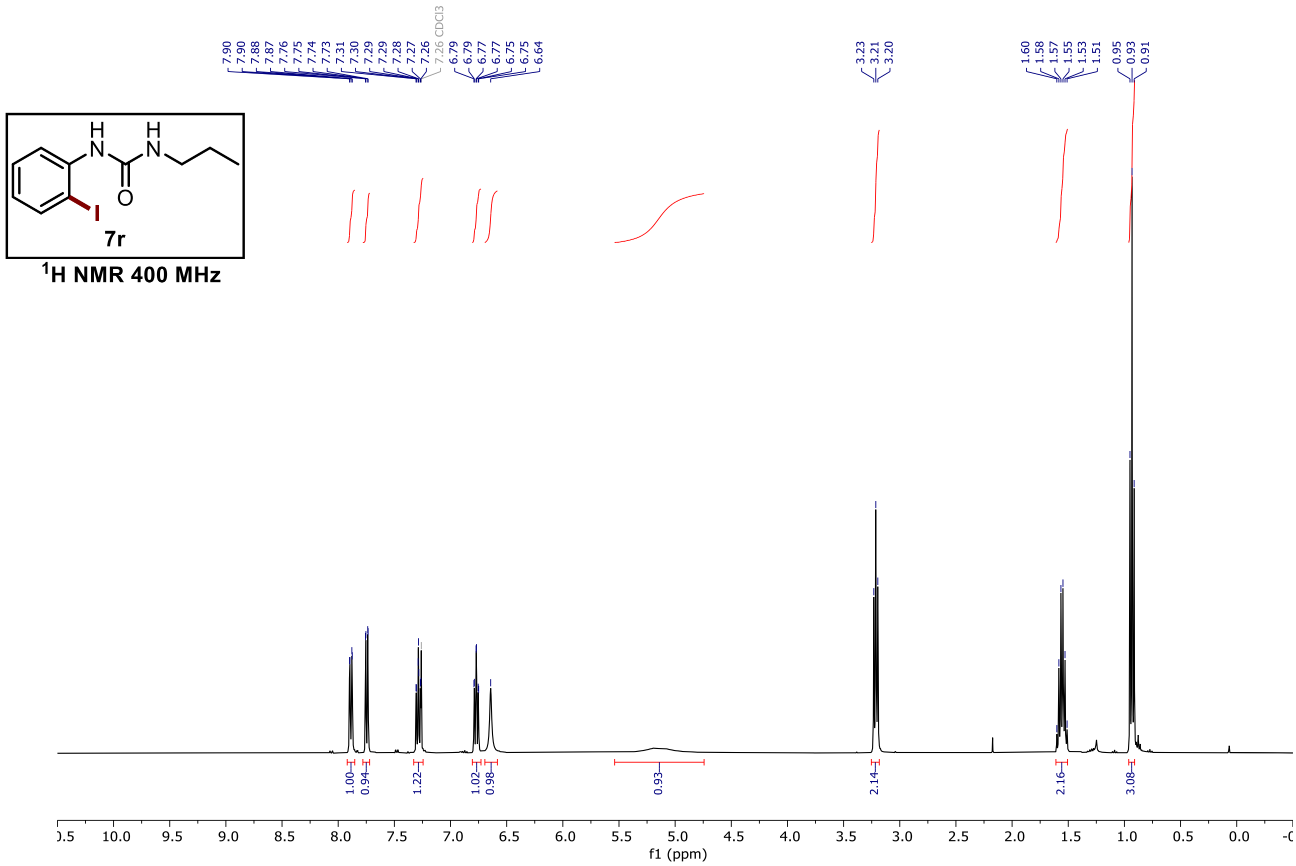
7r
¹H NMR 400 MHz
f1 (ppm)

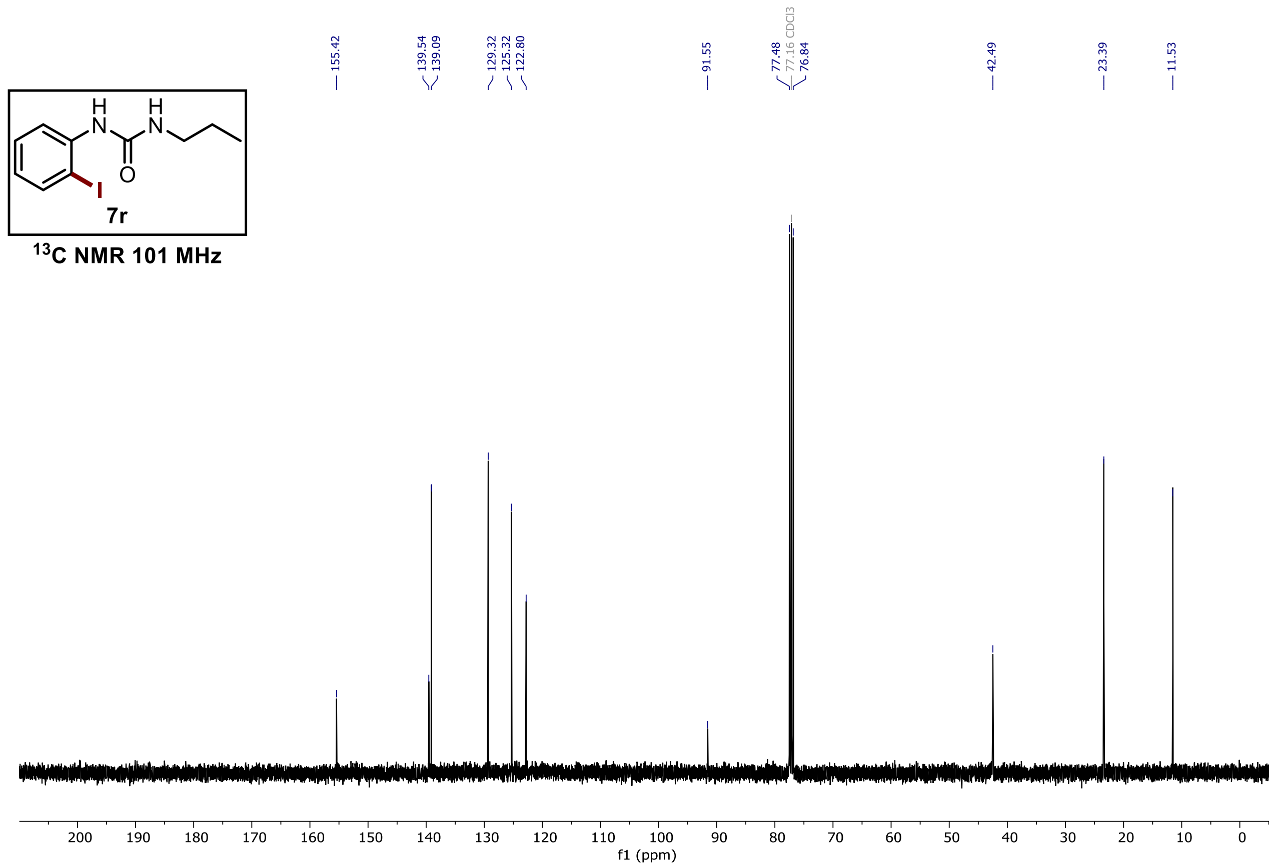
7r
¹³C NMR 101 MHz
f1 (ppm)

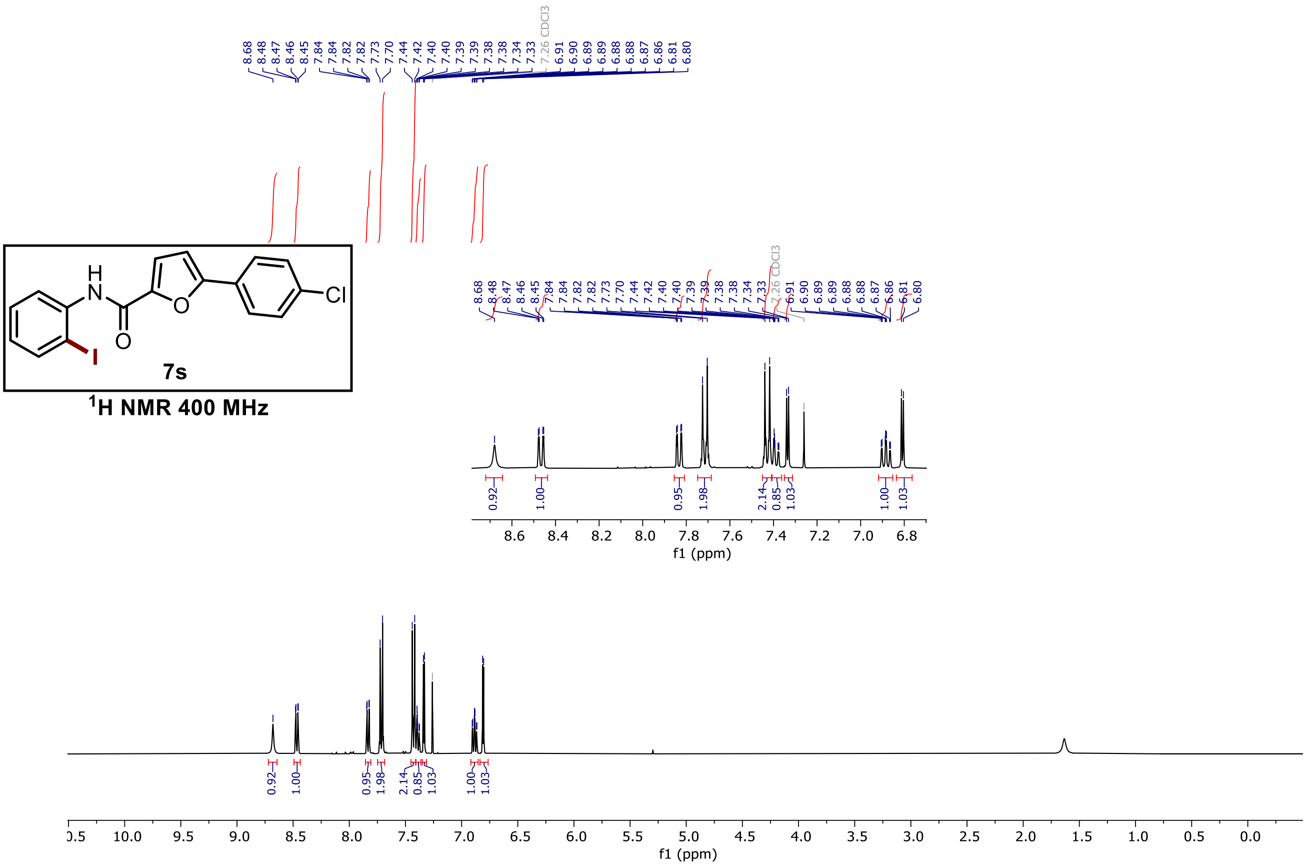

7s

^{1}H NMR 400 MHz

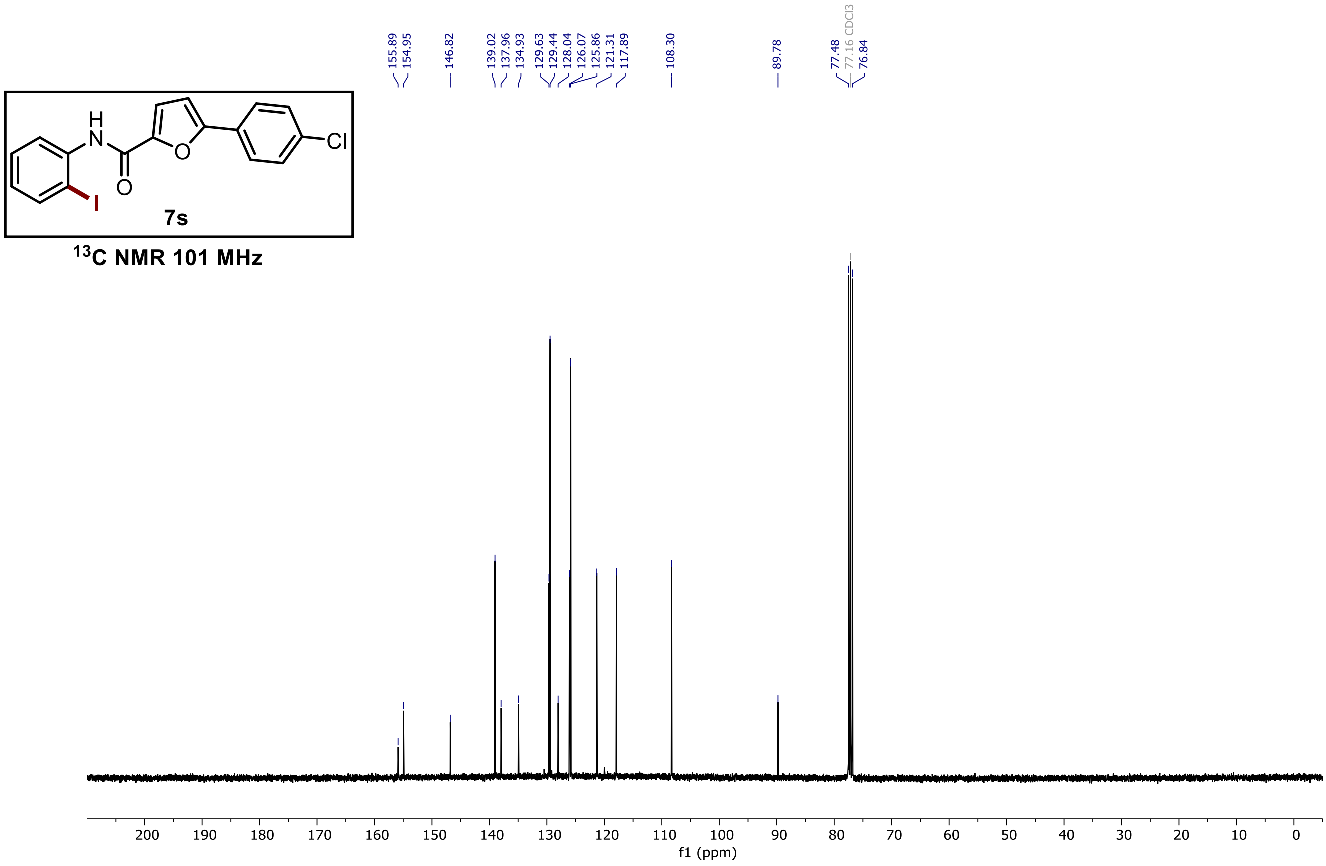

7s

^{13}C NMR 101 MHz

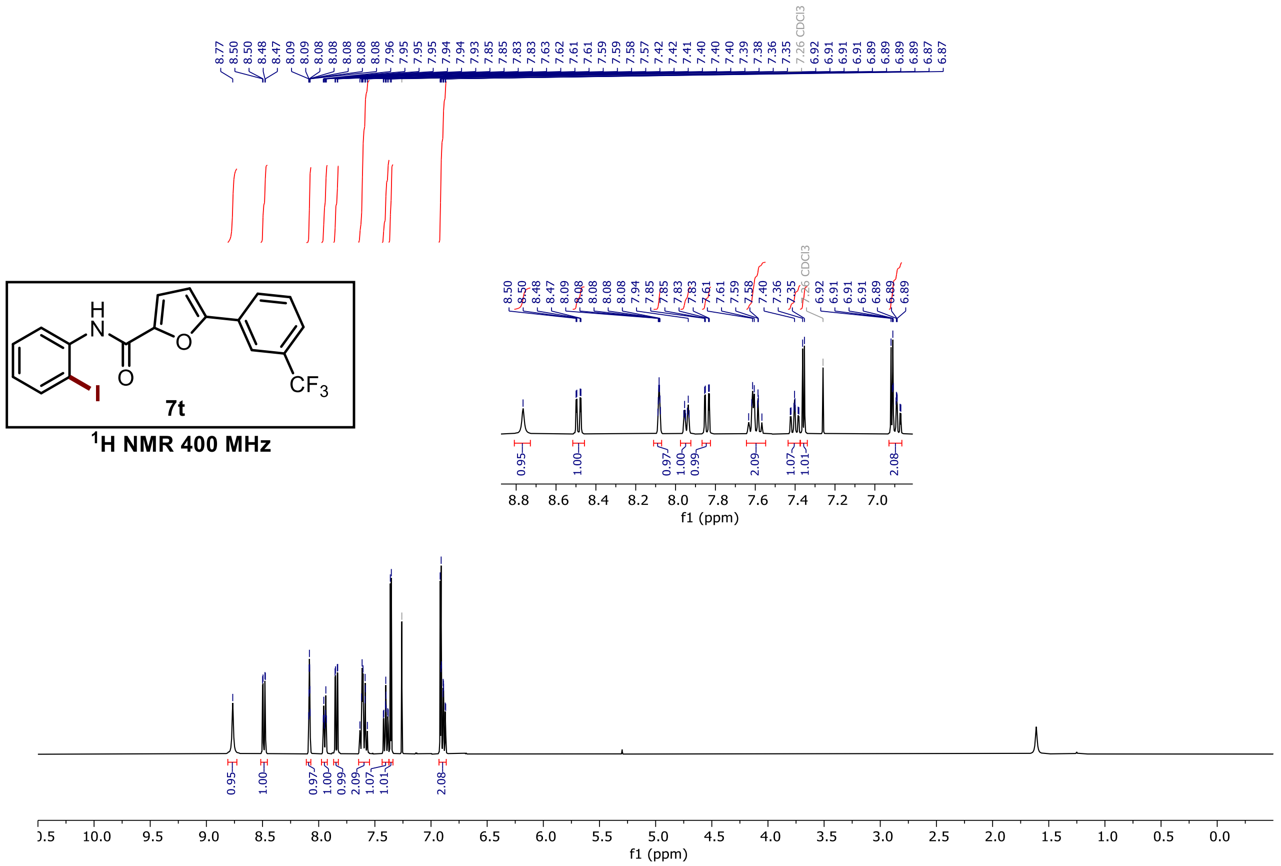

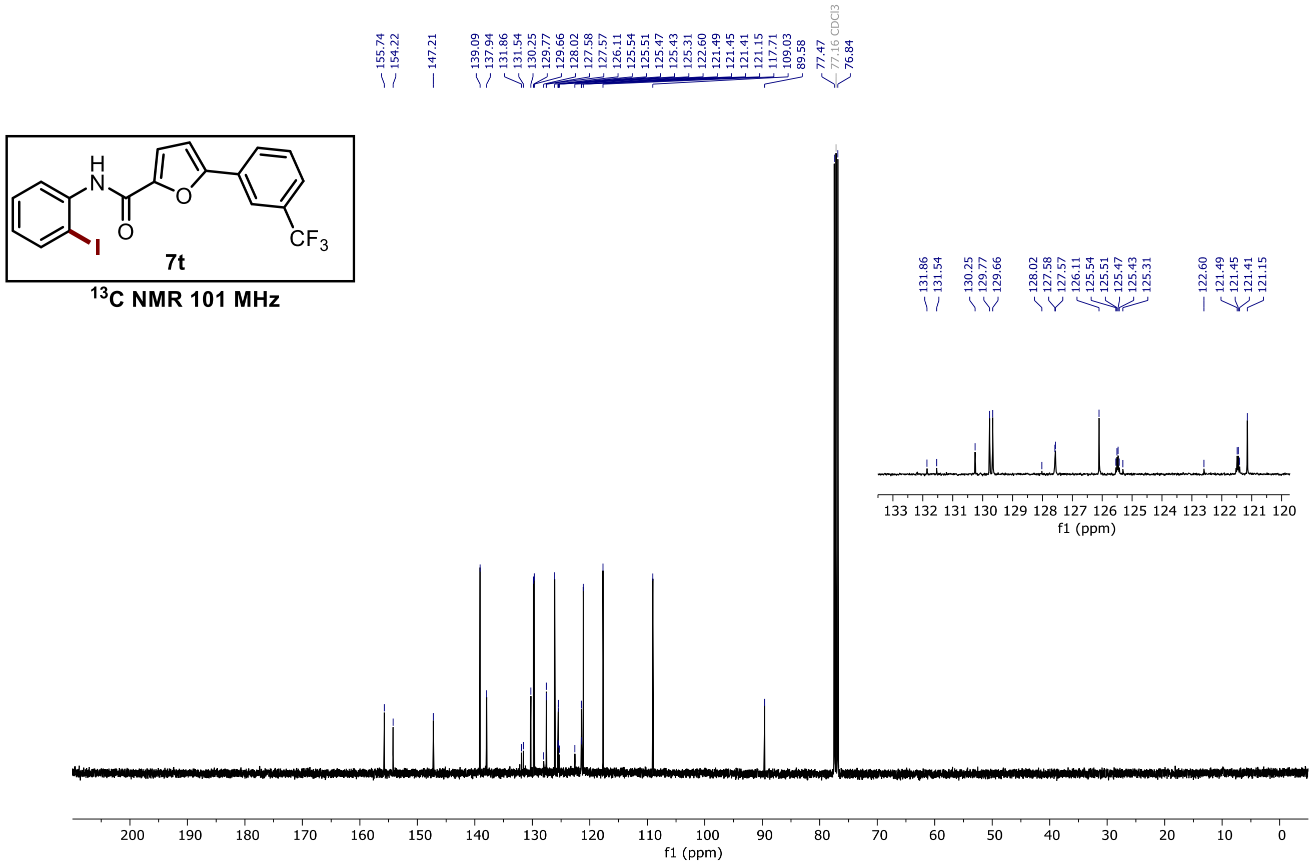

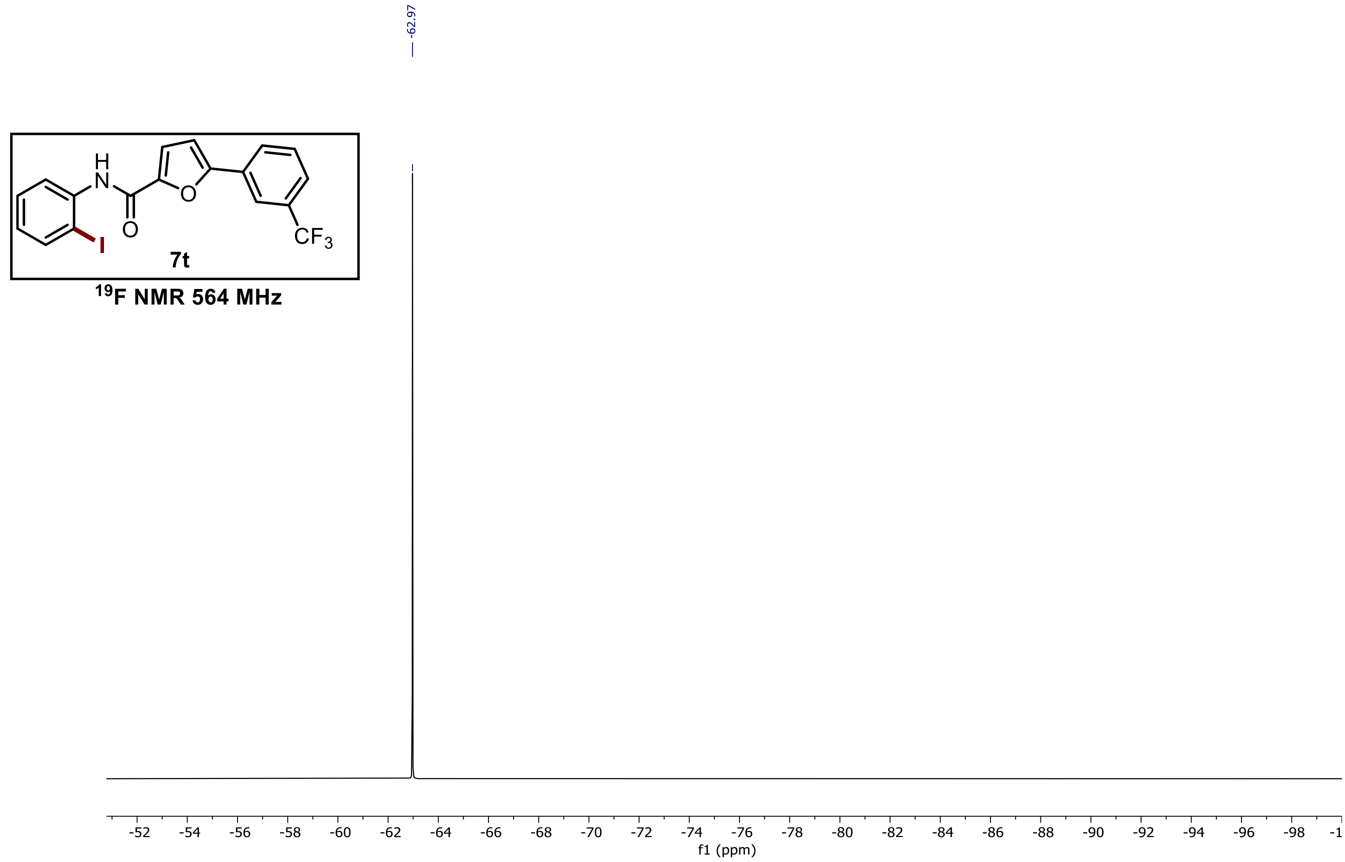

7t
^{19}F NMR 564 MHz
-62.97
f1 (ppm)

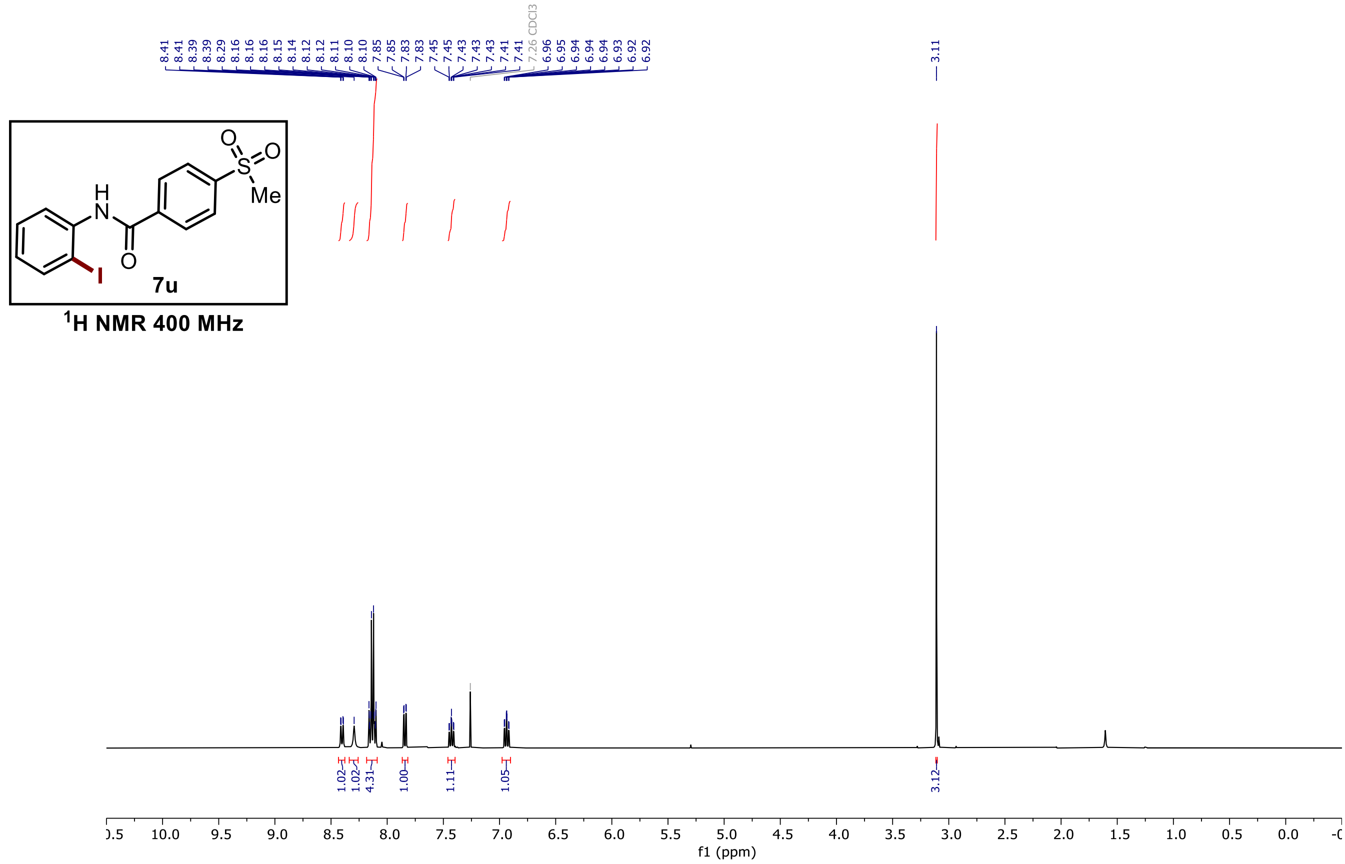

7u
^{1}H NMR 400 MHz
3.11
7.26 CDCl3
f1 (ppm)

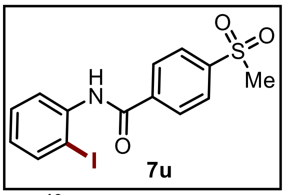

7u

^{13}C NMR 101 MHz

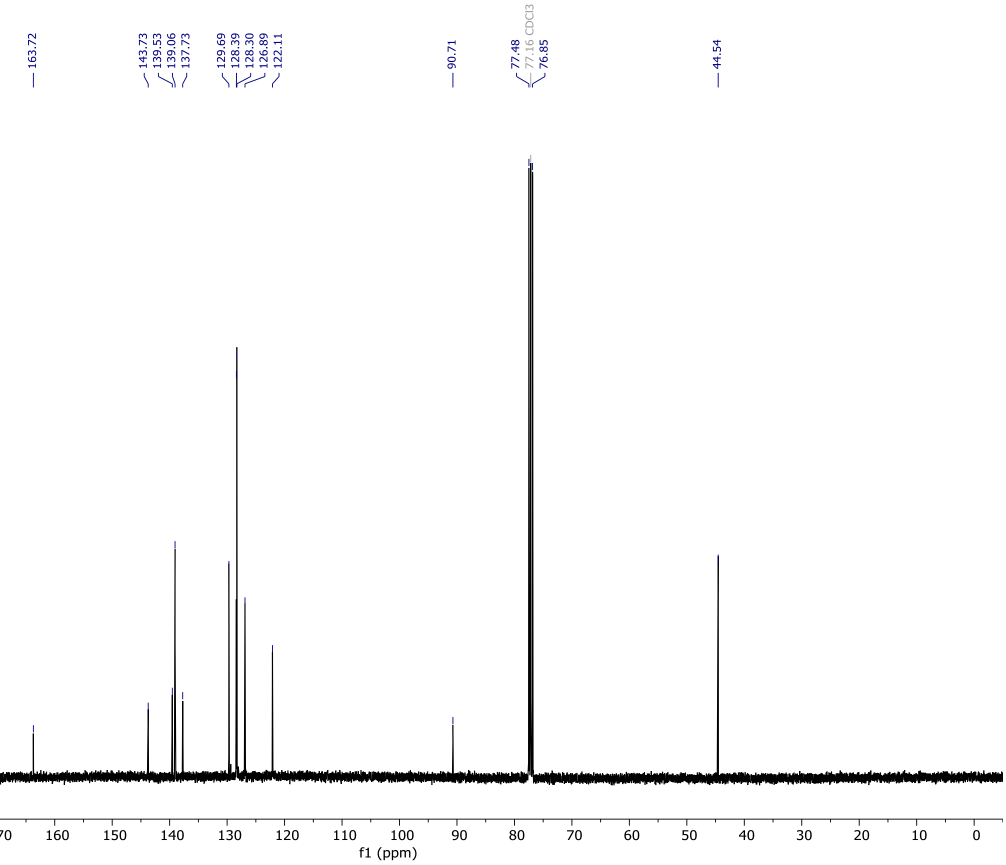

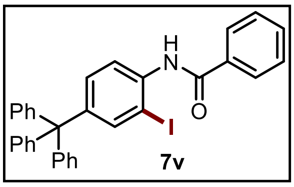

7v

^{1}H NMR 400 MHz

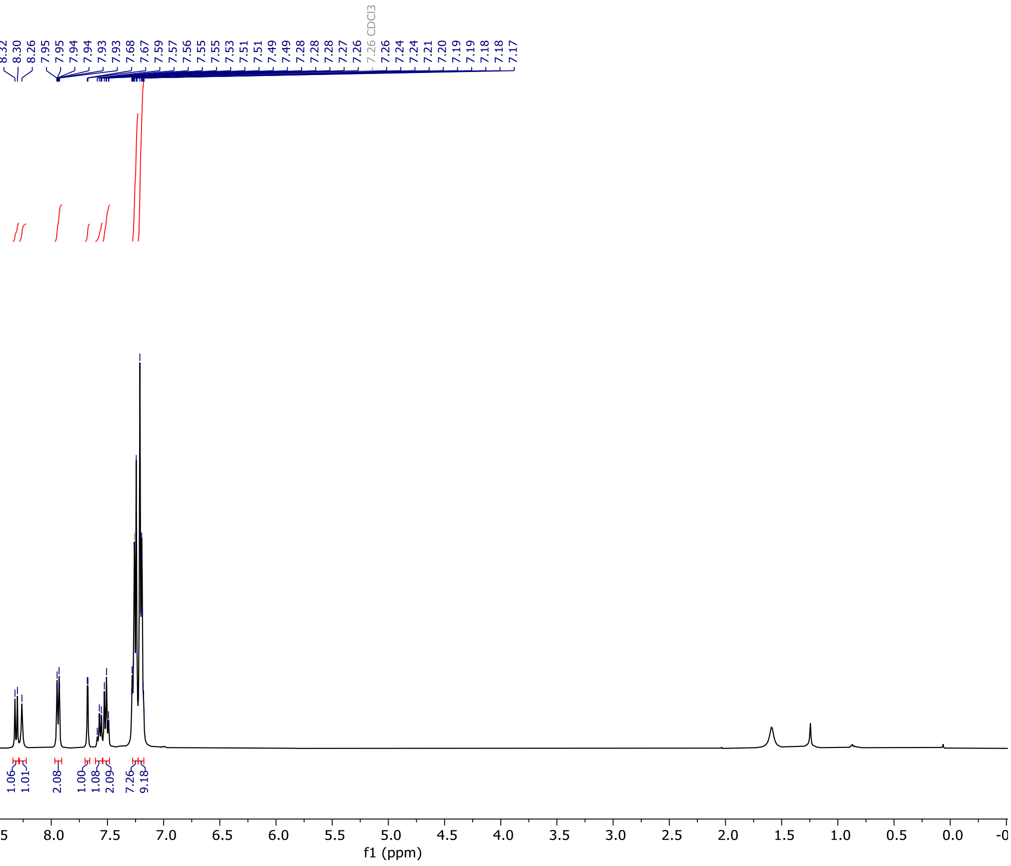

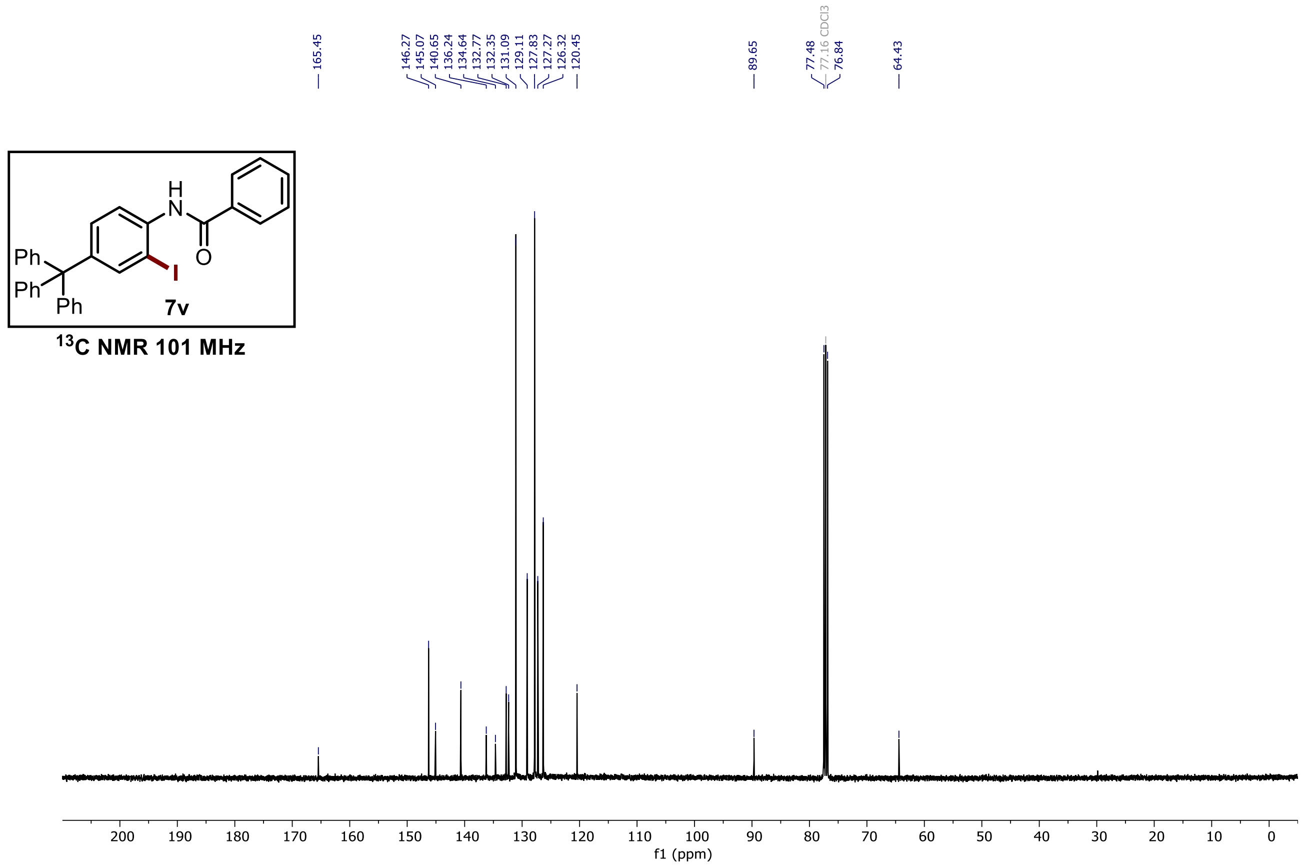
165.45
146.27
145.07
140.65
136.24
134.64
132.77
132.35
131.09
129.11
127.83
127.27
126.32
120.45
89.65
77.48
77.16 CDCl3
76.84
64.43
Ph
Ph
Ph
H
N
O
I
7v
13C NMR 101 MHz
f1 (ppm)

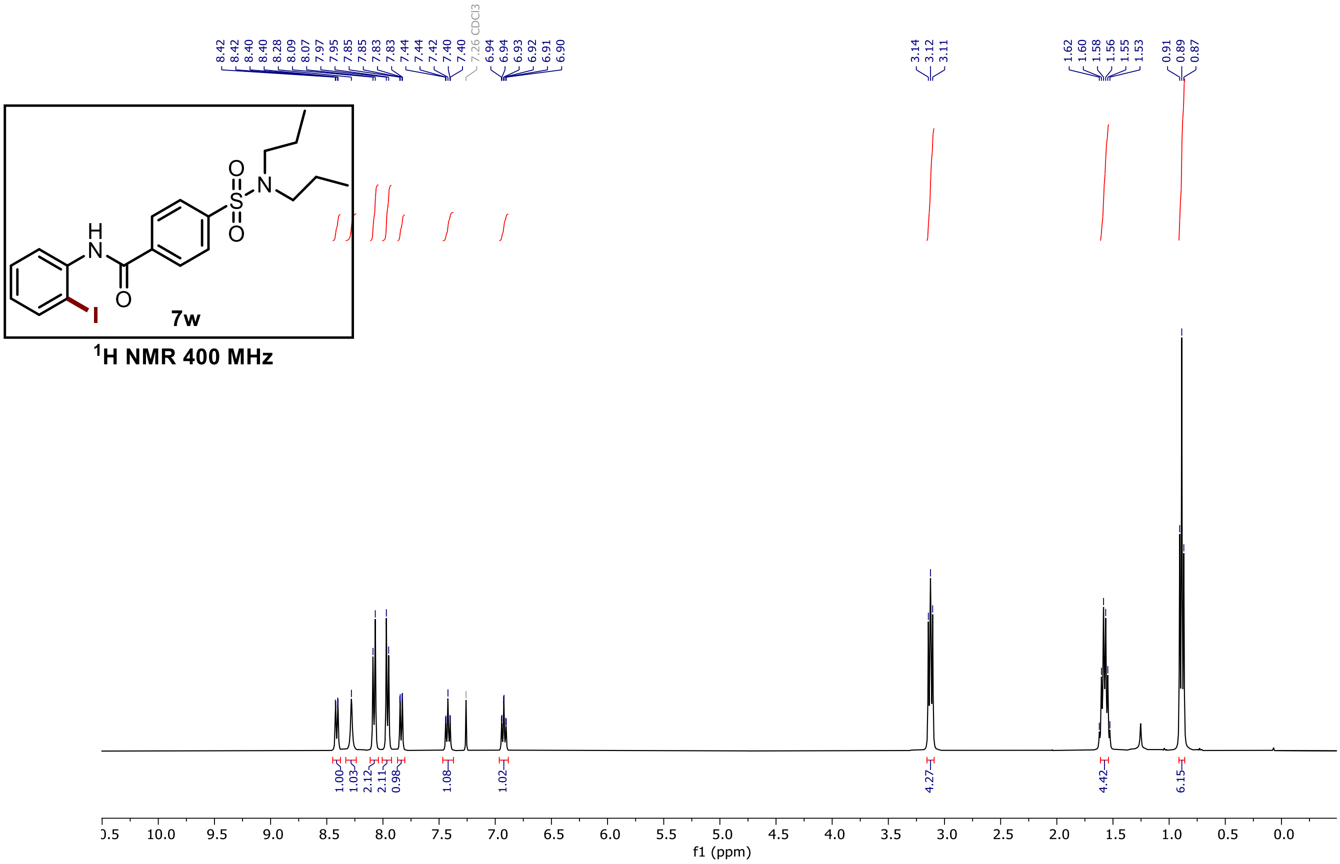
8.42
8.42
8.40
8.40
8.28
8.09
8.07
7.97
7.95
7.85
7.85
7.83
7.83
7.44
7.44
7.42
7.40
7.40
7.26 CDCl3
6.94
6.94
6.93
6.92
6.91
6.90
3.14
3.12
3.11
1.62
1.60
1.58
1.56
1.55
1.53
0.91
0.89
0.87
O
S
O
N
H
N
O
I
7w
1H NMR 400 MHz
1.00
1.03
2.12
2.11
0.98
1.08
1.02
4.27
4.42
6.15
f1 (ppm)

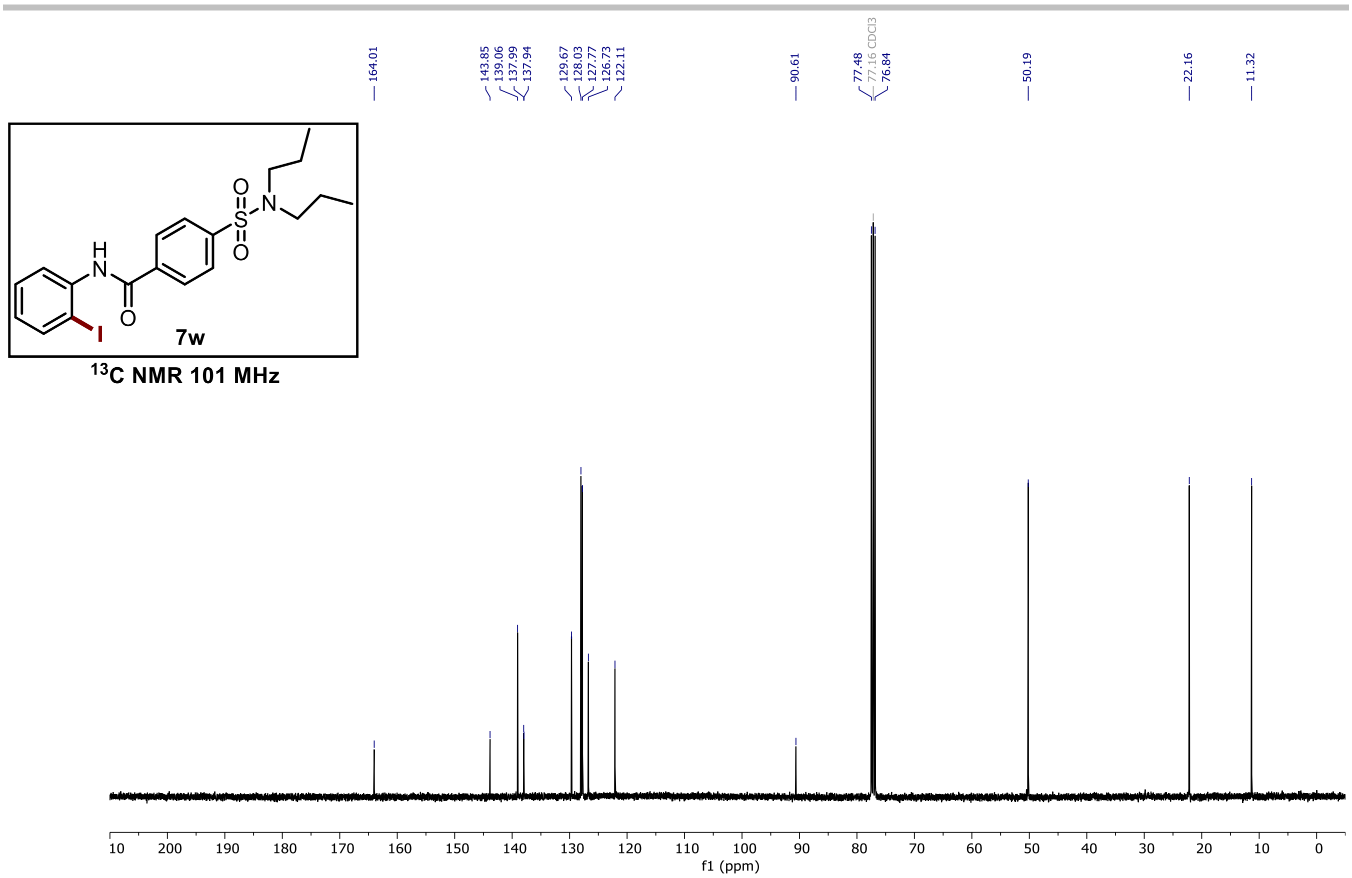
164.01
143.85
139.06
137.99
137.94
129.67
128.03
127.77
126.73
122.11
90.61
77.48
77.16 CDCl3
76.84
50.19
22.16
11.32
7w
^{13}C NMR 101 MHz
f1 (ppm)

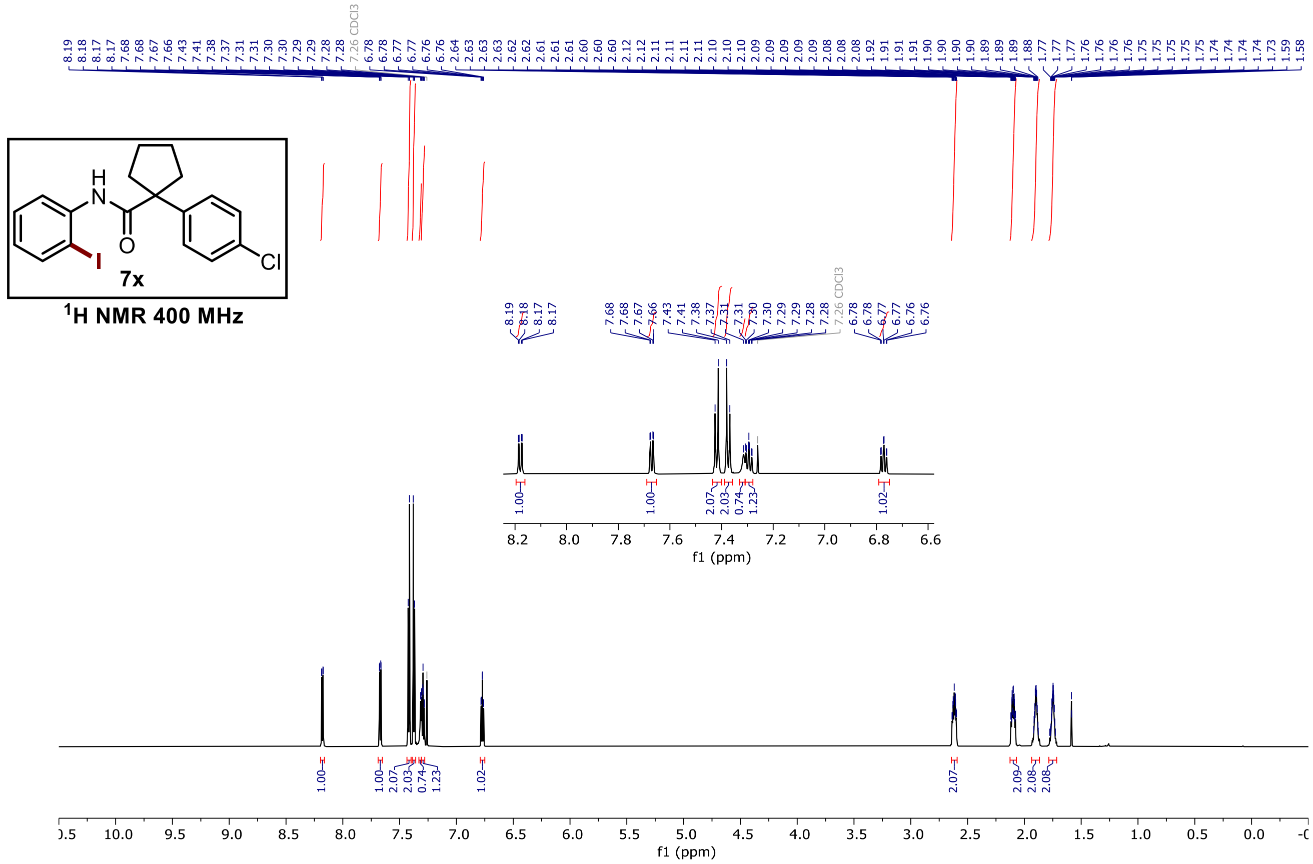
7x
^{1}H NMR 400 MHz
7.26 CDCl3
1.00
1.00
2.07
2.03
0.74
1.23
1.02
2.07
2.09
2.08
2.08
f1 (ppm)

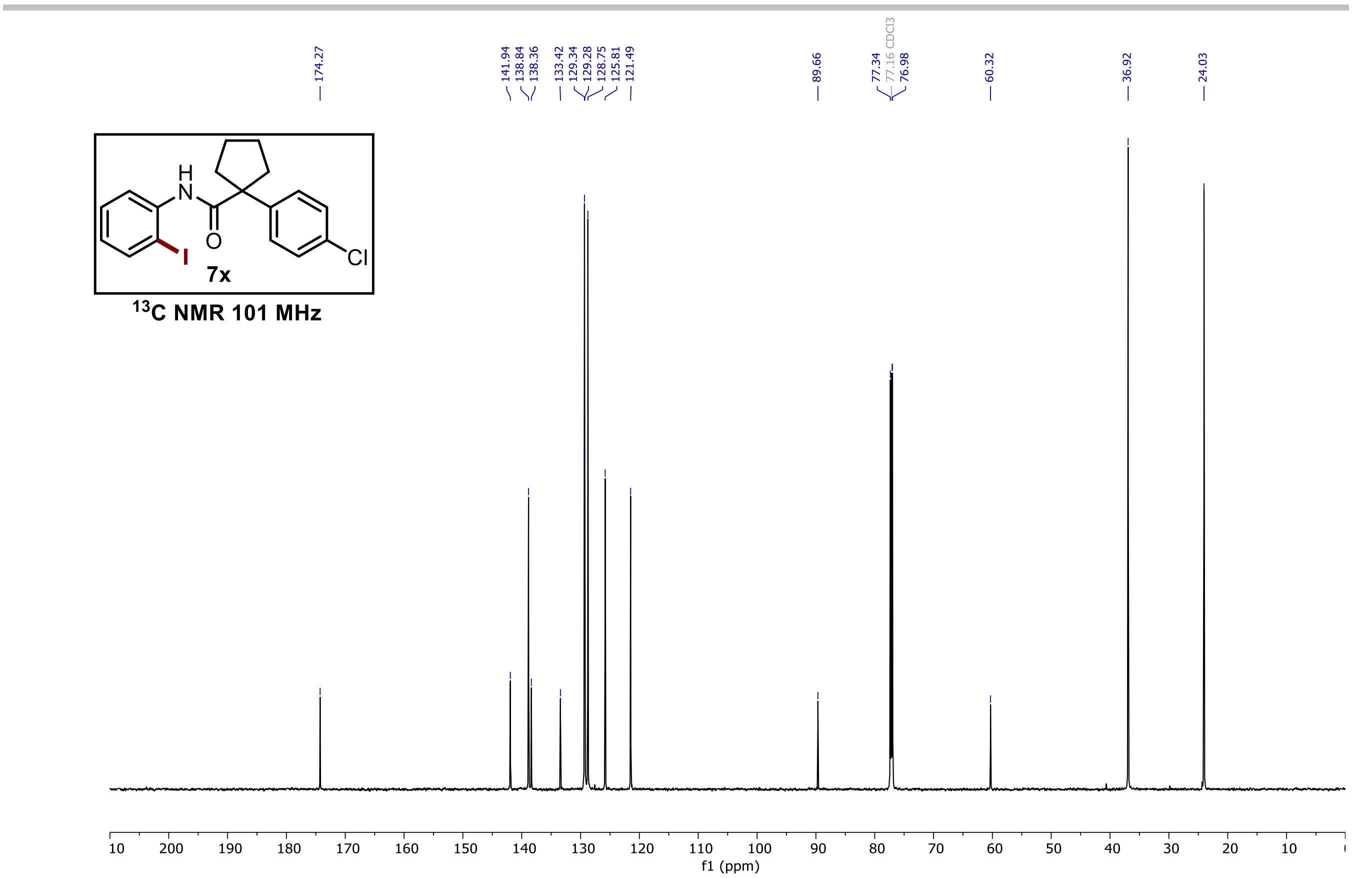

174.27
141.94
138.84
138.36
133.42
129.34
129.28
128.75
125.81
121.49
89.66
77.34
77.16 CDCl3
76.98
60.32
36.92
24.03
7x
f1 (ppm)

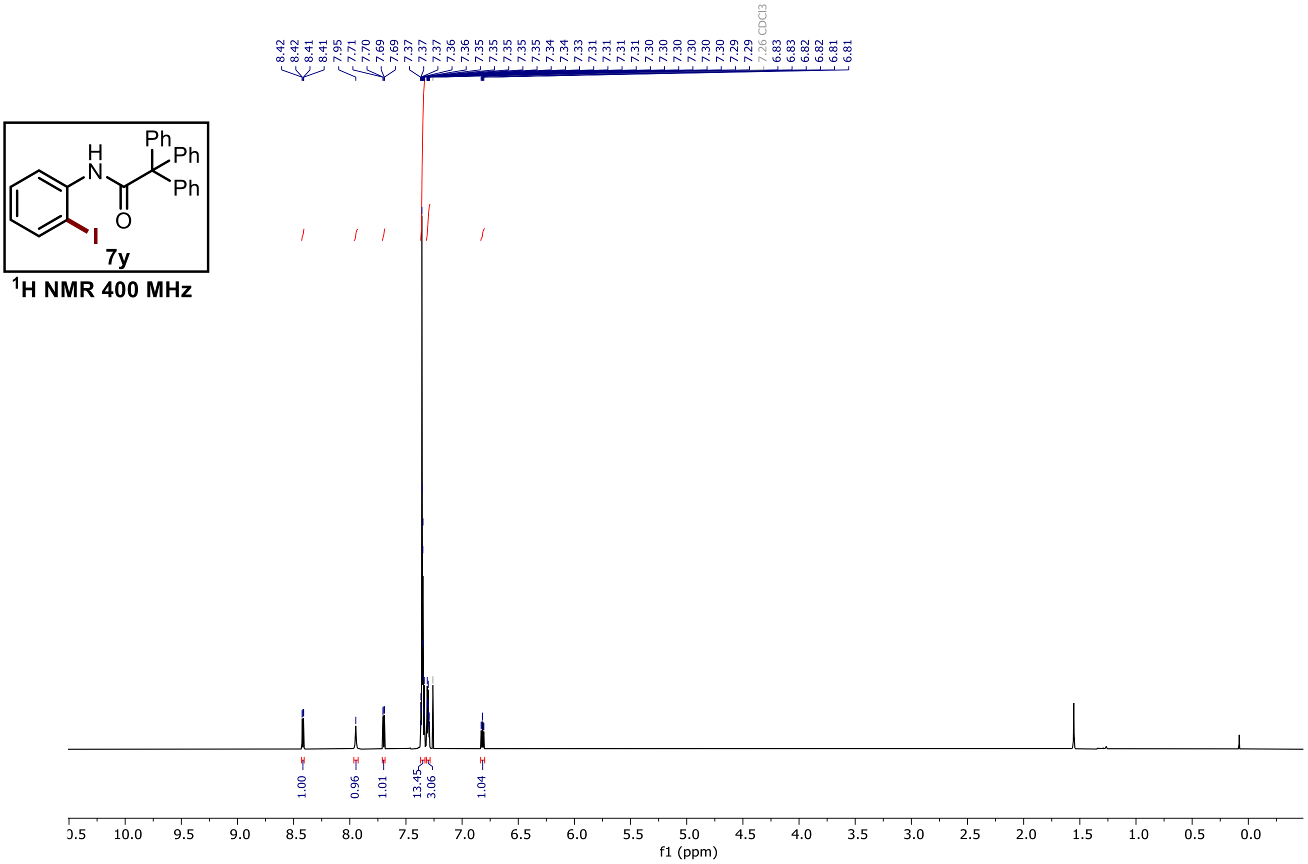

7y
f1 (ppm)

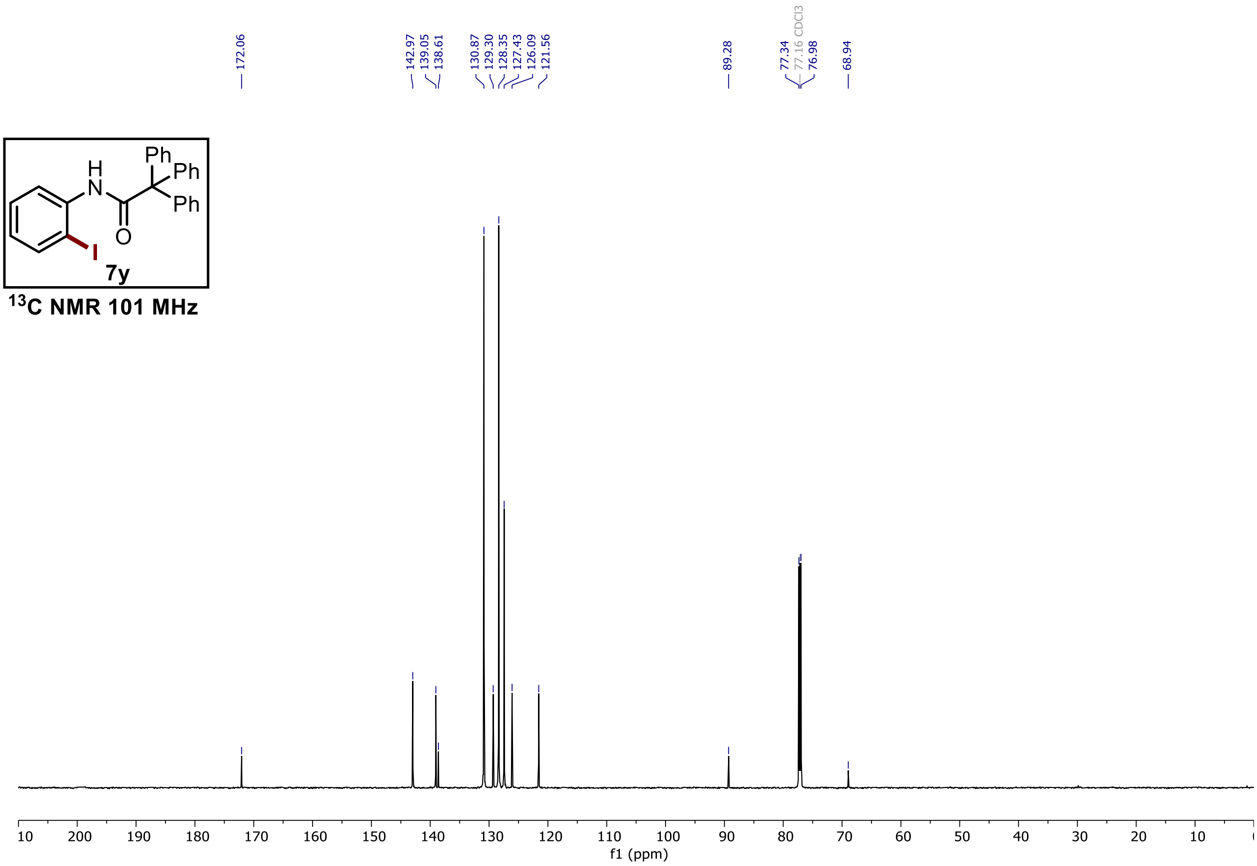

^{13}C NMR 101 MHz

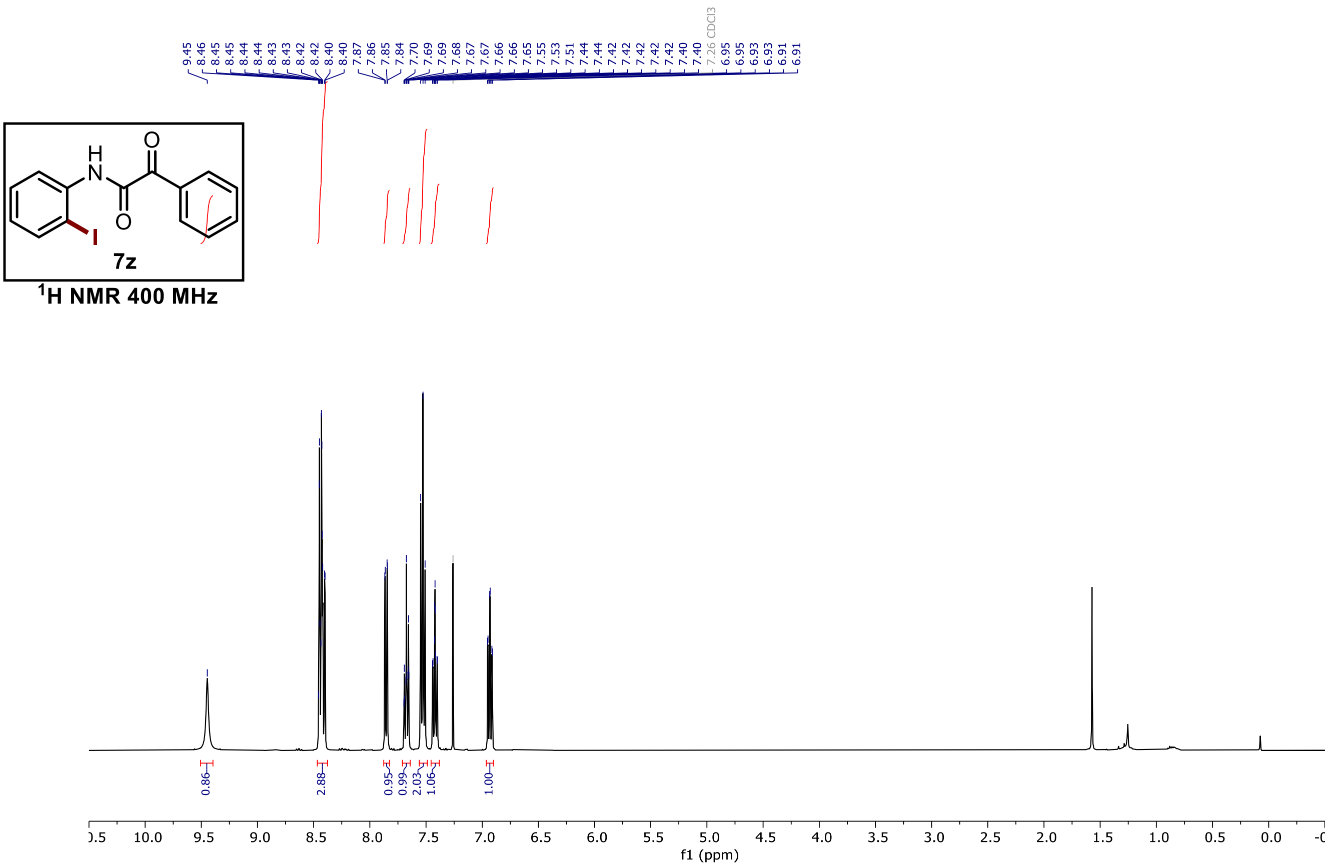

^{1}H NMR 400 MHz

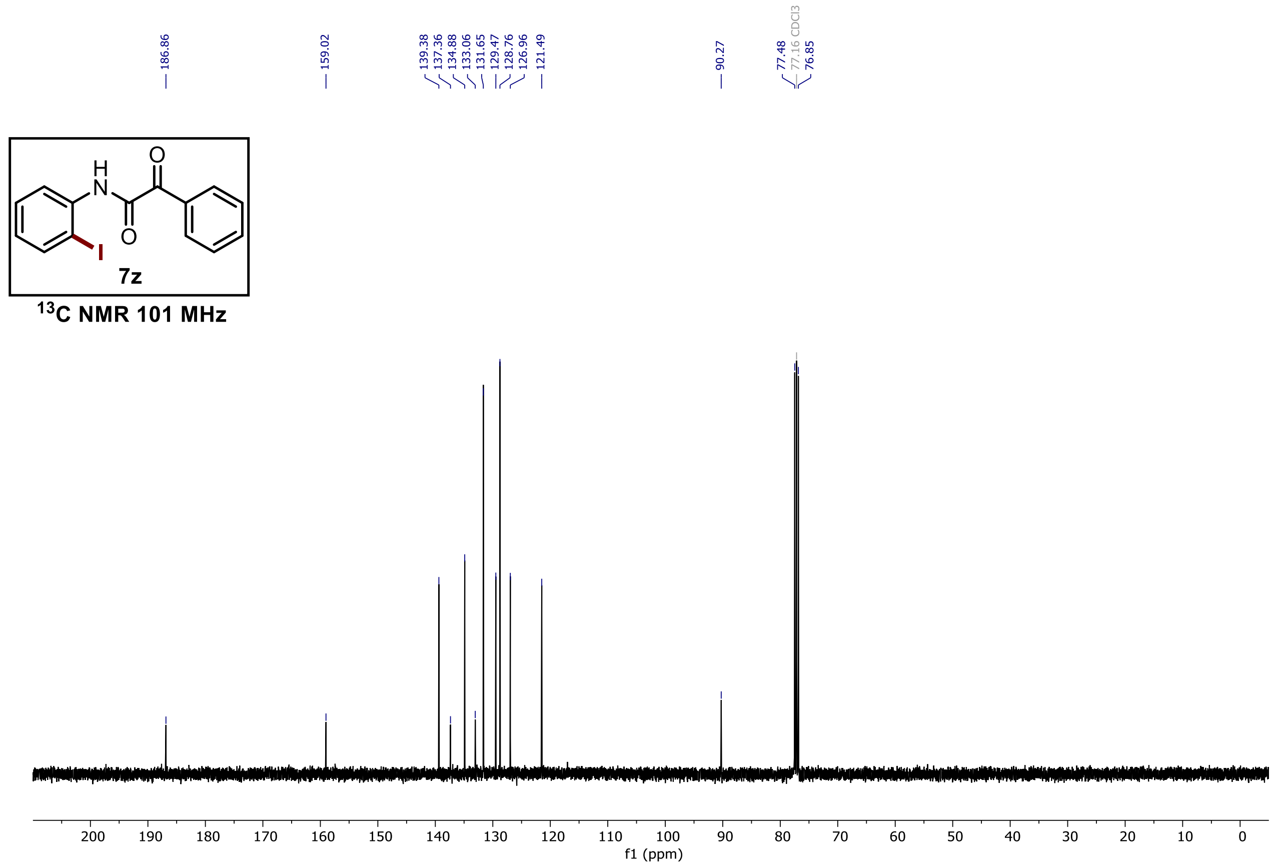
186.86
159.02
139.38
137.36
134.88
133.06
131.65
129.47
128.76
126.96
121.49
90.27
77.48
77.16 CDCl3
76.85
7z
13C NMR 101 MHz
f1 (ppm)

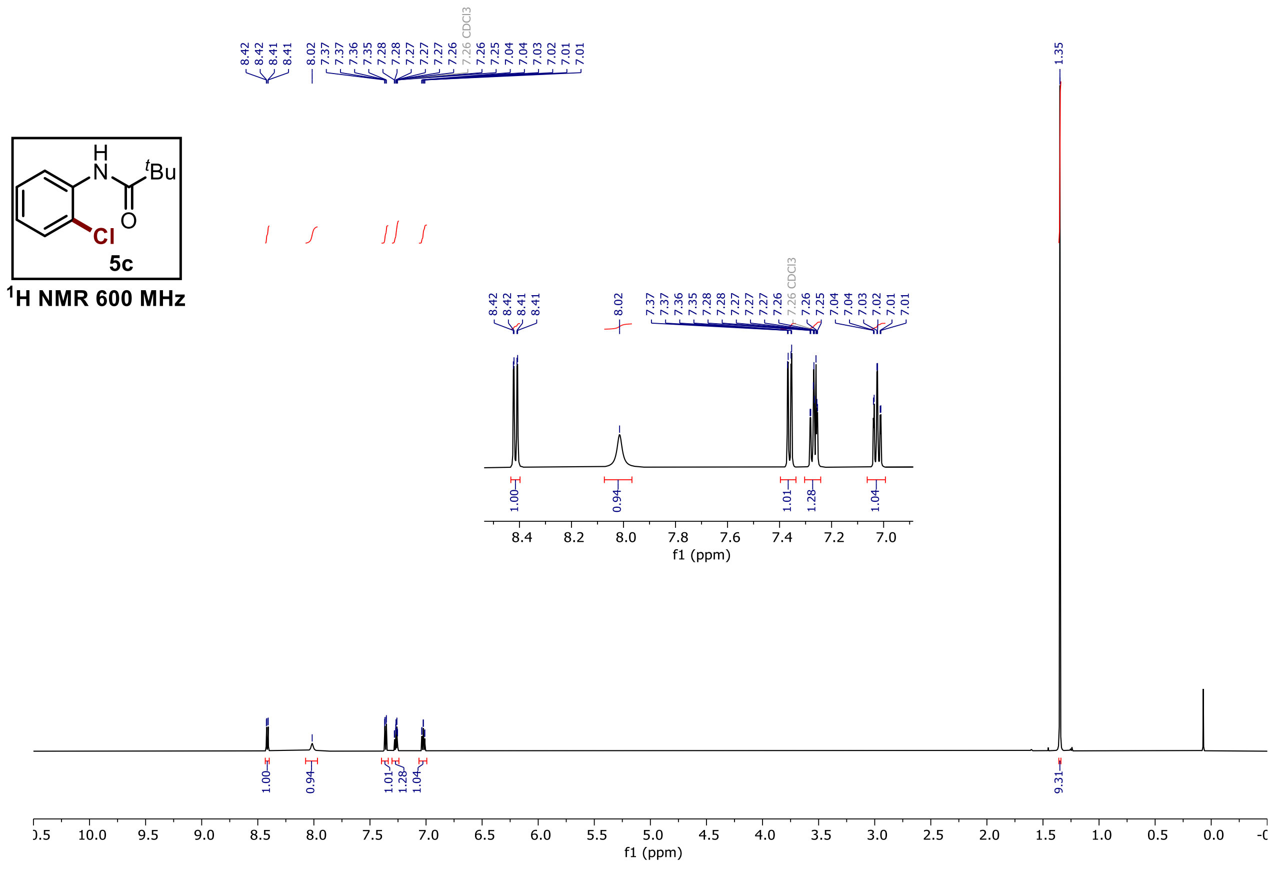
8.42
8.42
8.41
8.41
8.02
7.37
7.37
7.36
7.35
7.28
7.28
7.27
7.27
7.27
7.26
7.26 CDCl3
7.26
7.25
7.04
7.04
7.03
7.02
7.01
7.01
1.35
tBu
Cl
5c
1H NMR 600 MHz
1.00
0.94
1.01
1.28
1.04
9.31
f1 (ppm)

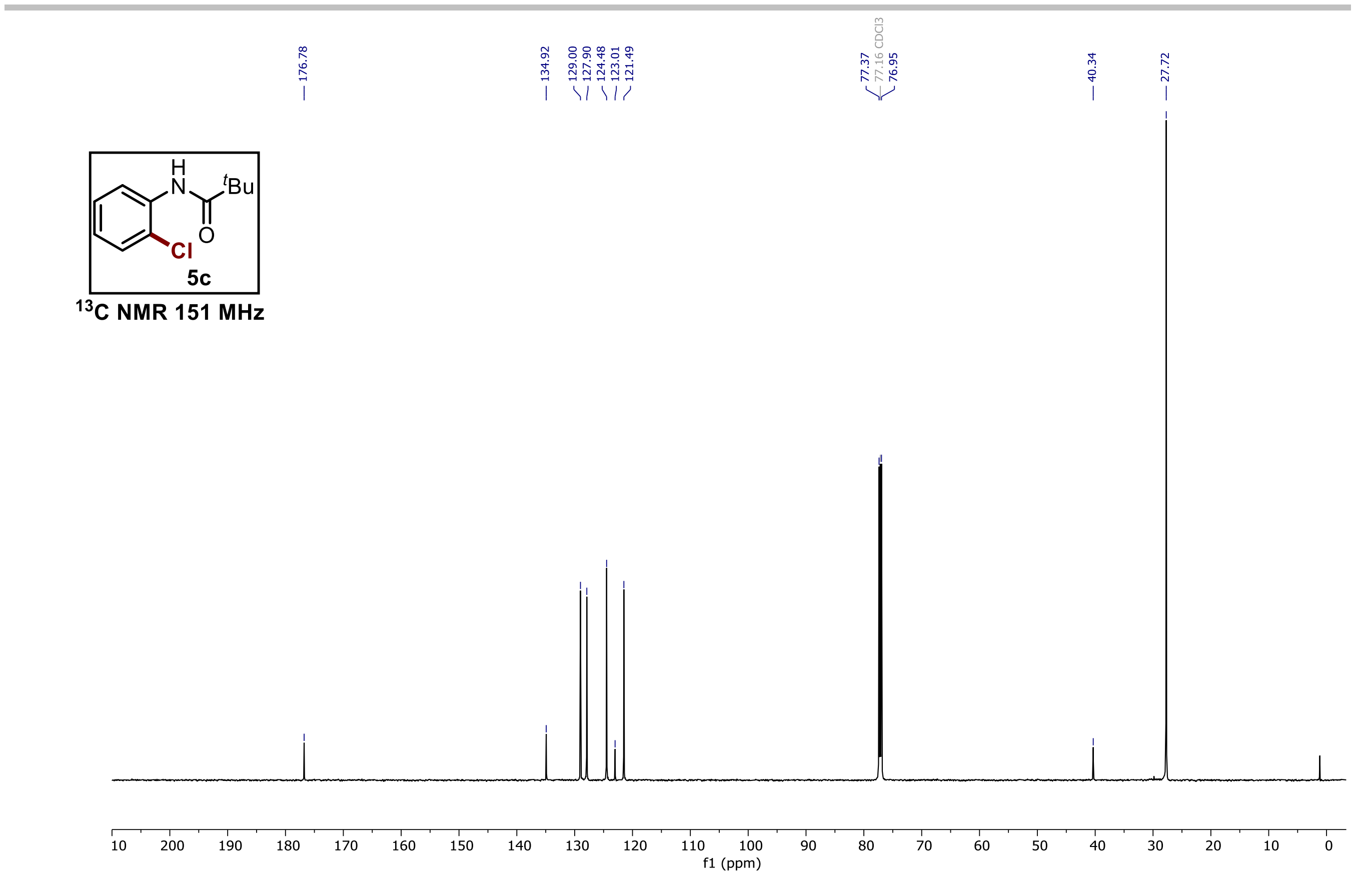
176.78
134.92
129.00
127.90
124.48
123.01
121.49
77.37
77.16 CDCl3
76.95
40.34
27.72
H
N
tBu
O
Cl
5c
13C NMR 151 MHz
f1 (ppm)

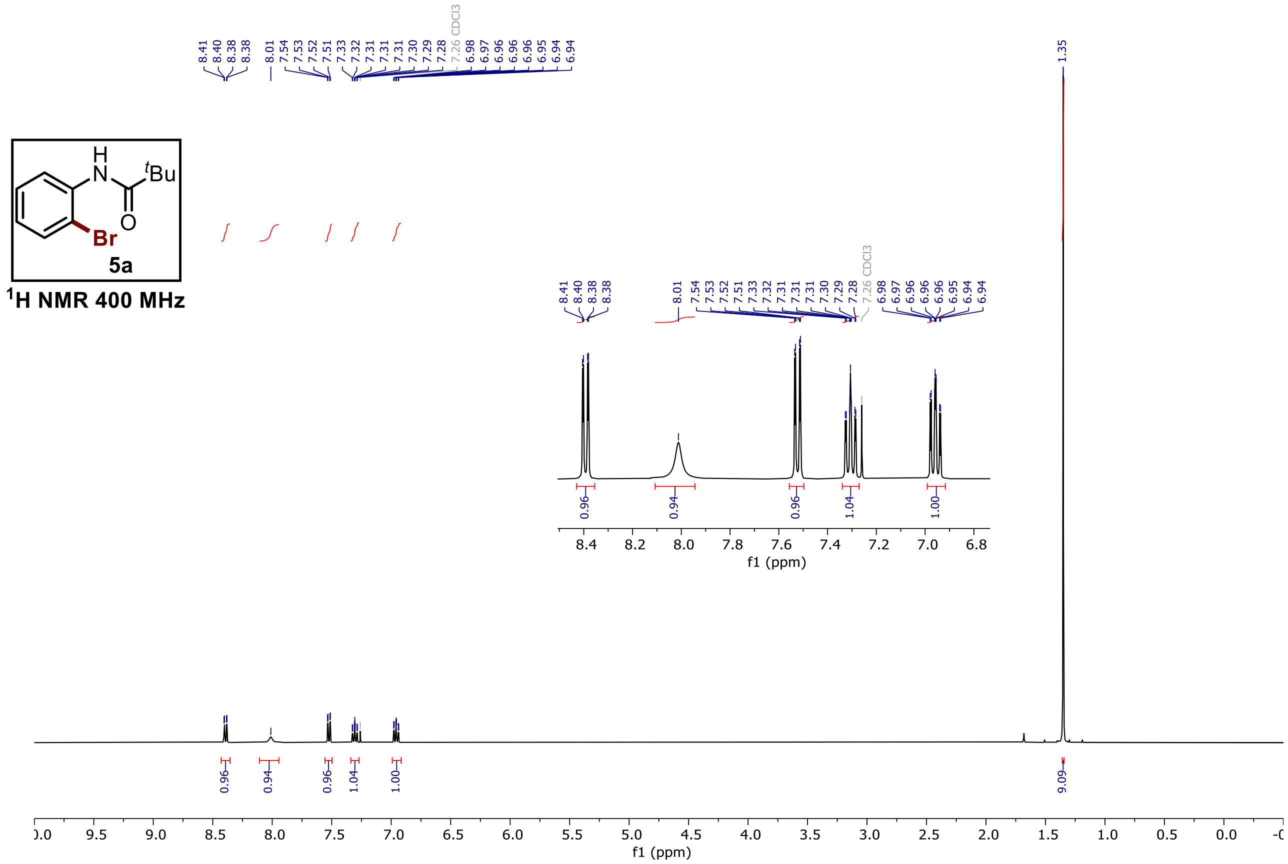
8.41
8.40
8.38
8.38
8.01
7.54
7.53
7.52
7.51
7.33
7.32
7.31
7.31
7.31
7.30
7.29
7.28
7.26 CDCl3
6.98
6.97
6.96
6.96
6.96
6.95
6.94
6.94
1.35
H
N
tBu
O
Br
5a
1H NMR 400 MHz
0.96
0.94
0.96
1.04
1.00
9.09
f1 (ppm)

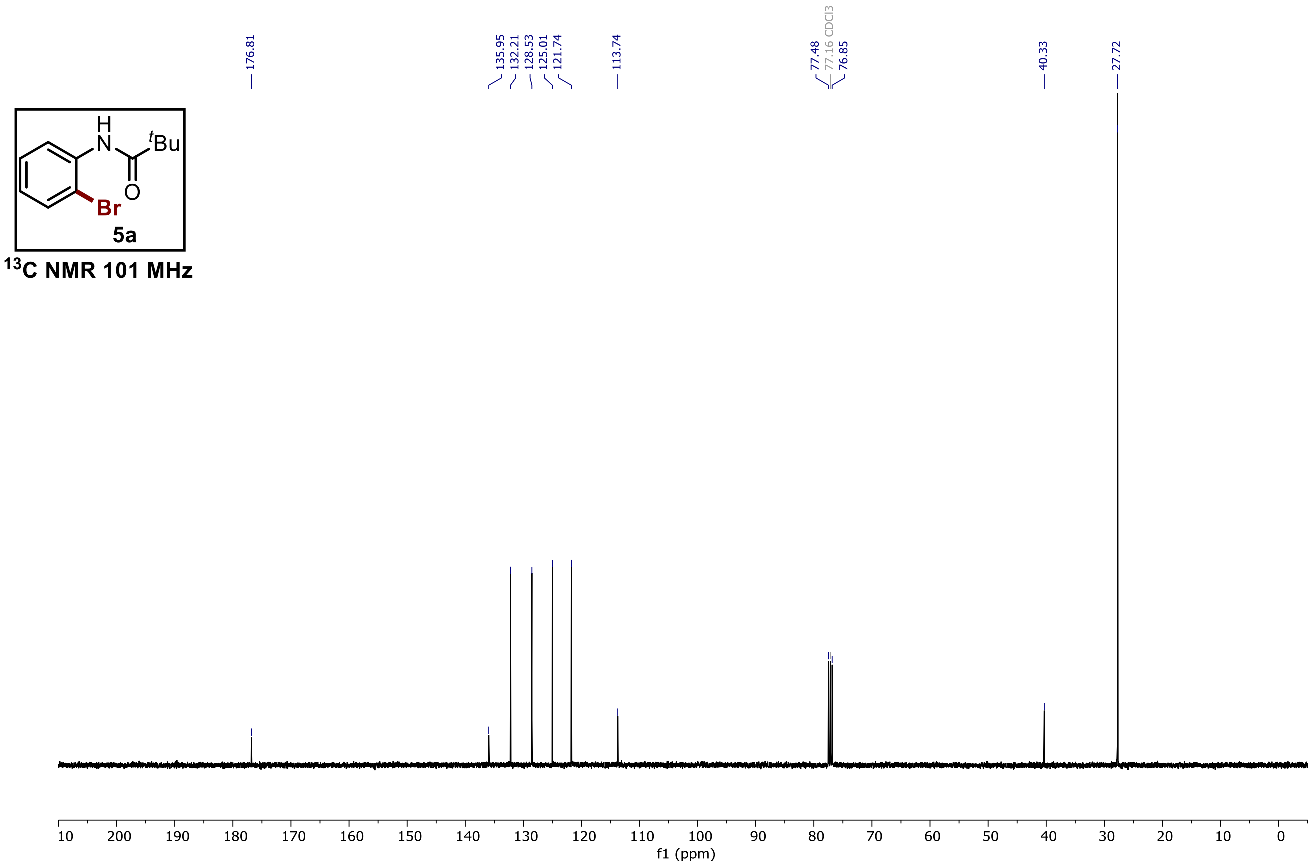

5a
13C NMR 101 MHz
176.81
135.95
132.21
128.53
125.01
121.74
113.74
77.48
77.16 CDCl3
76.85
40.33
27.72
f1 (ppm)

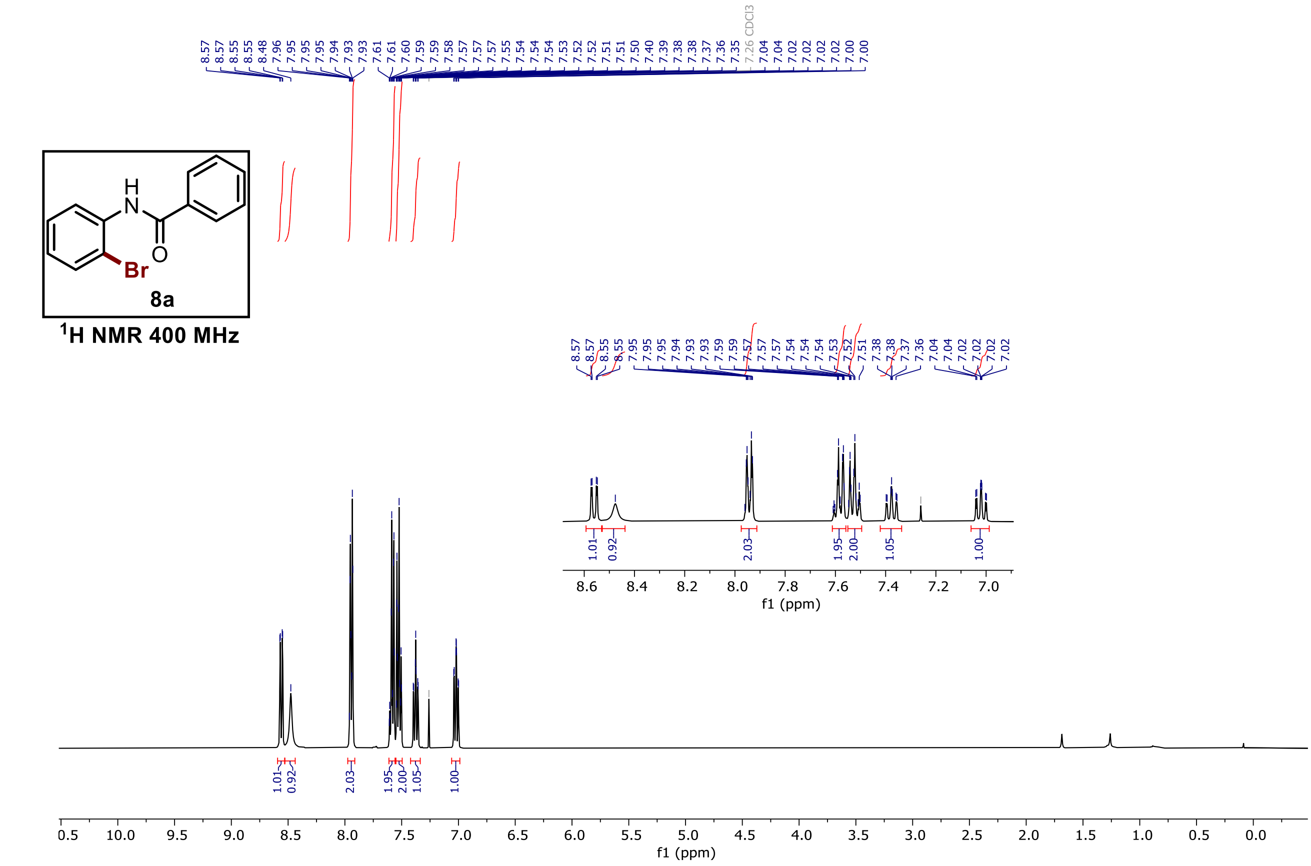

8a
1H NMR 400 MHz
7.26 CDCl3
1.01
0.92
2.03
1.95
2.00
1.05
1.00
f1 (ppm)

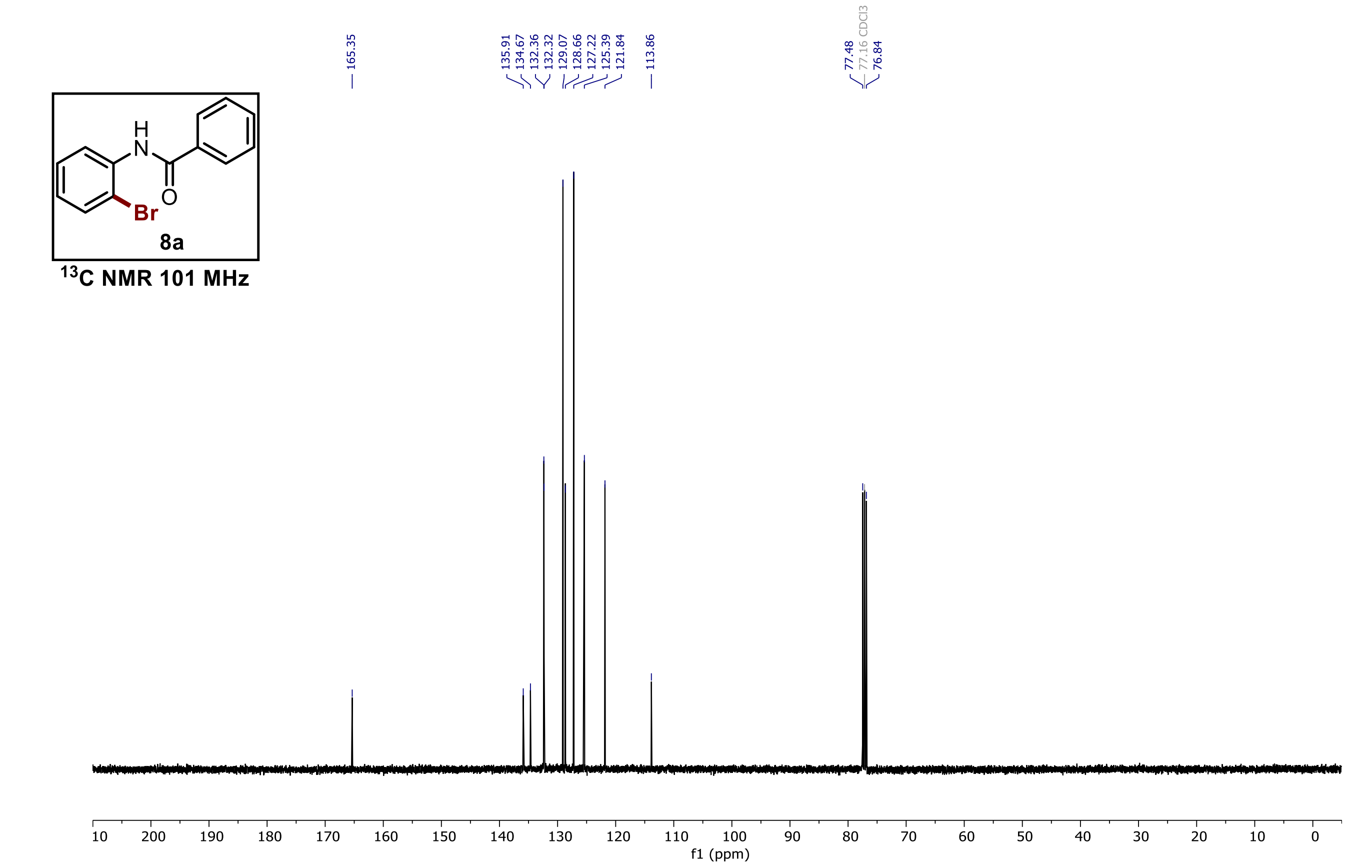
165.35
135.91
134.67
132.36
132.32
129.07
128.66
127.22
125.39
121.84
113.86
77.48
77.16 CDCl3
76.84
8a
13C NMR 101 MHz
f1 (ppm)

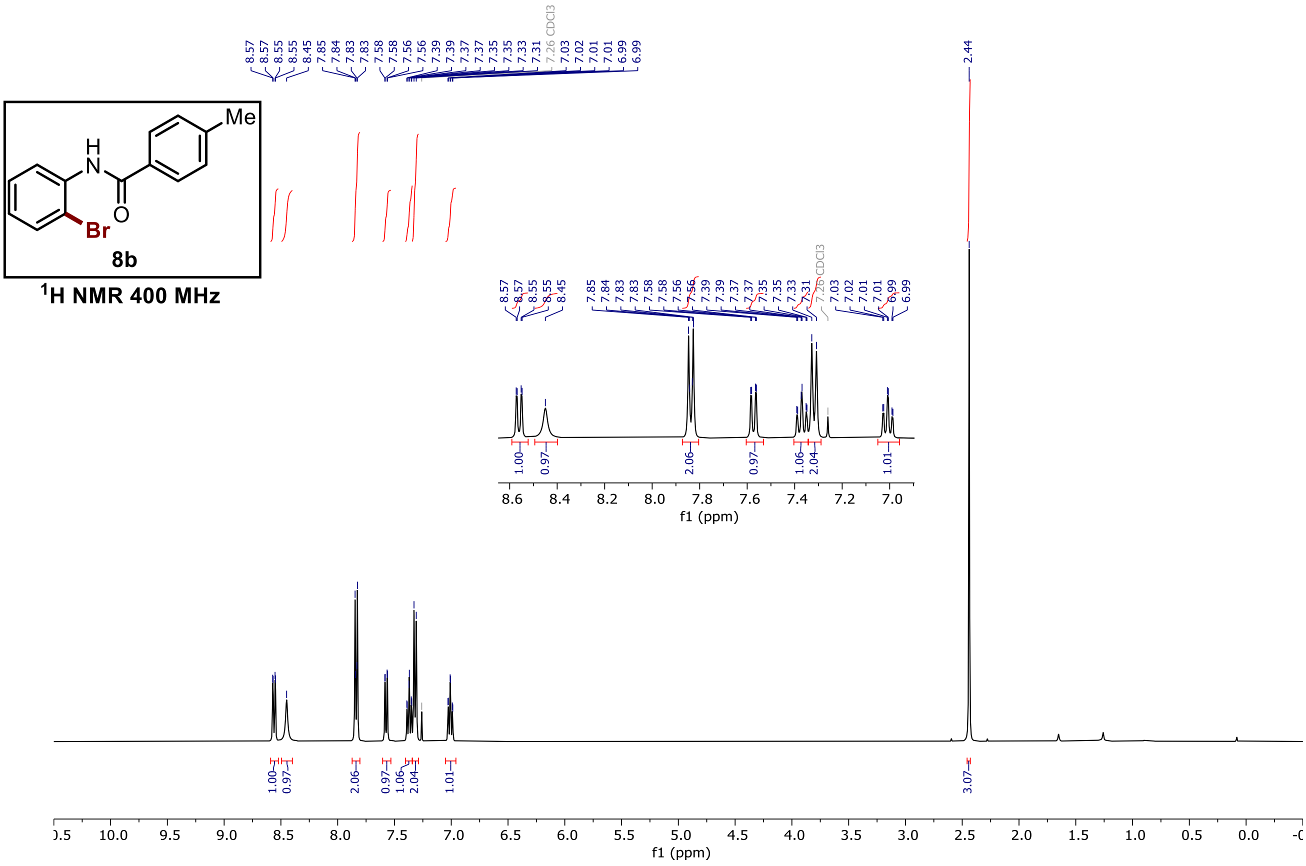
8b
1H NMR 400 MHz
2.44
f1 (ppm)

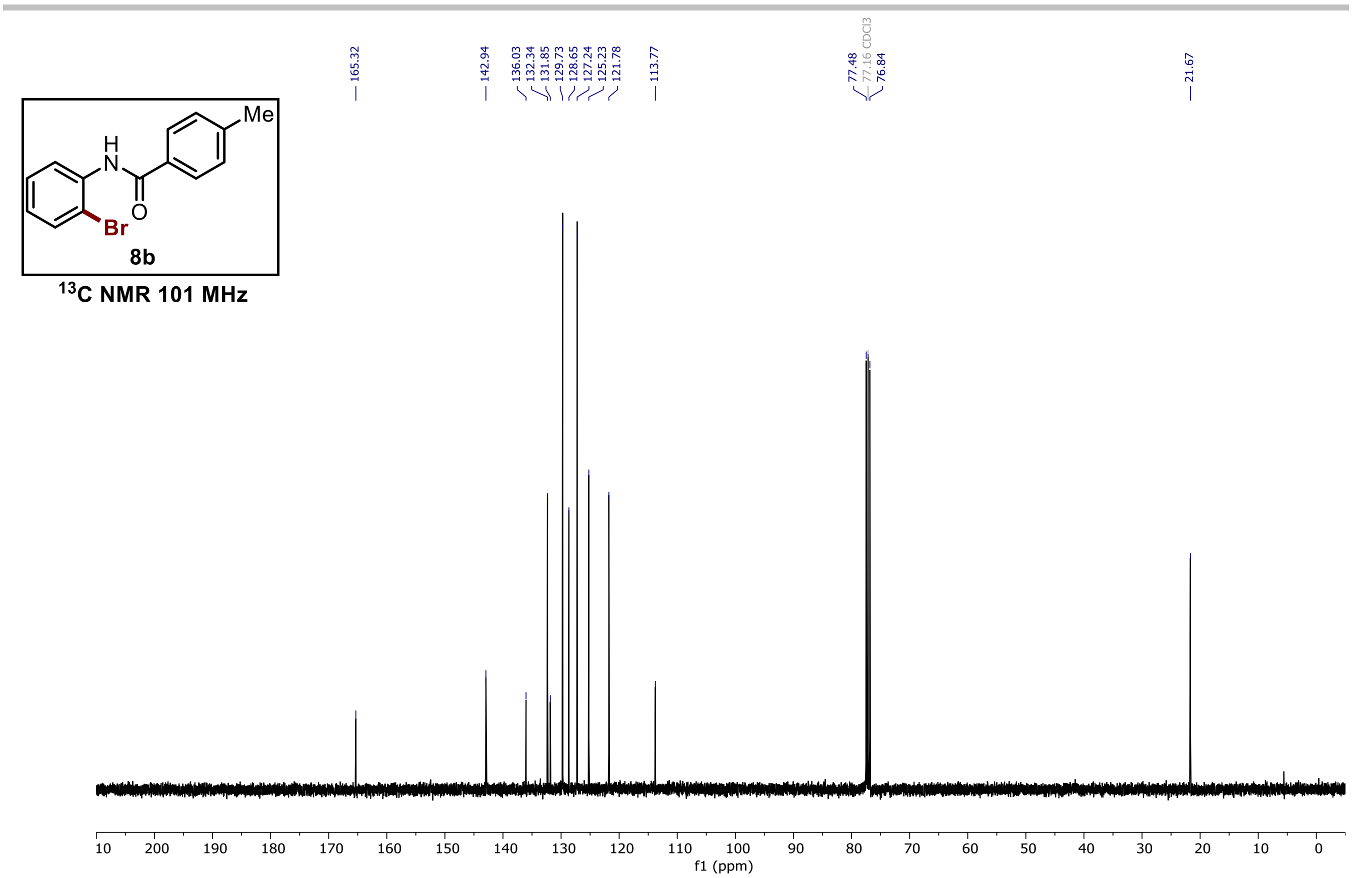
165.32
142.94
136.03
132.34
131.85
129.73
128.65
127.24
125.23
121.78
113.77
77.48
77.16 CDCl3
76.84
21.67
Me
H
N
O
Br
8b
^{13}C NMR 101 MHz
f1 (ppm)

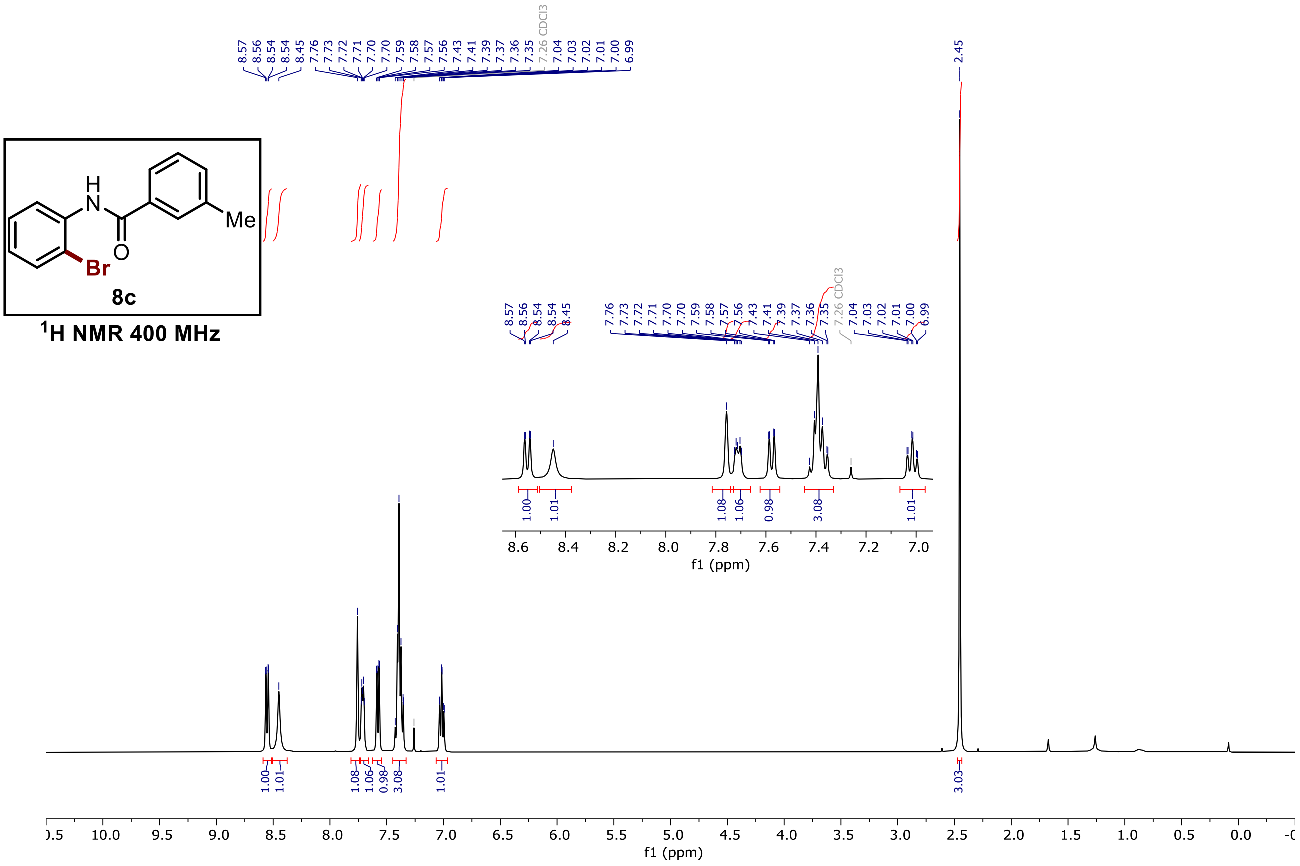
2.45
7.26 CDCl3
Me
H
N
O
Br
8c
^{1}H NMR 400 MHz
1.00
1.01
1.08
1.06
0.98
3.08
1.01
3.03
f1 (ppm)

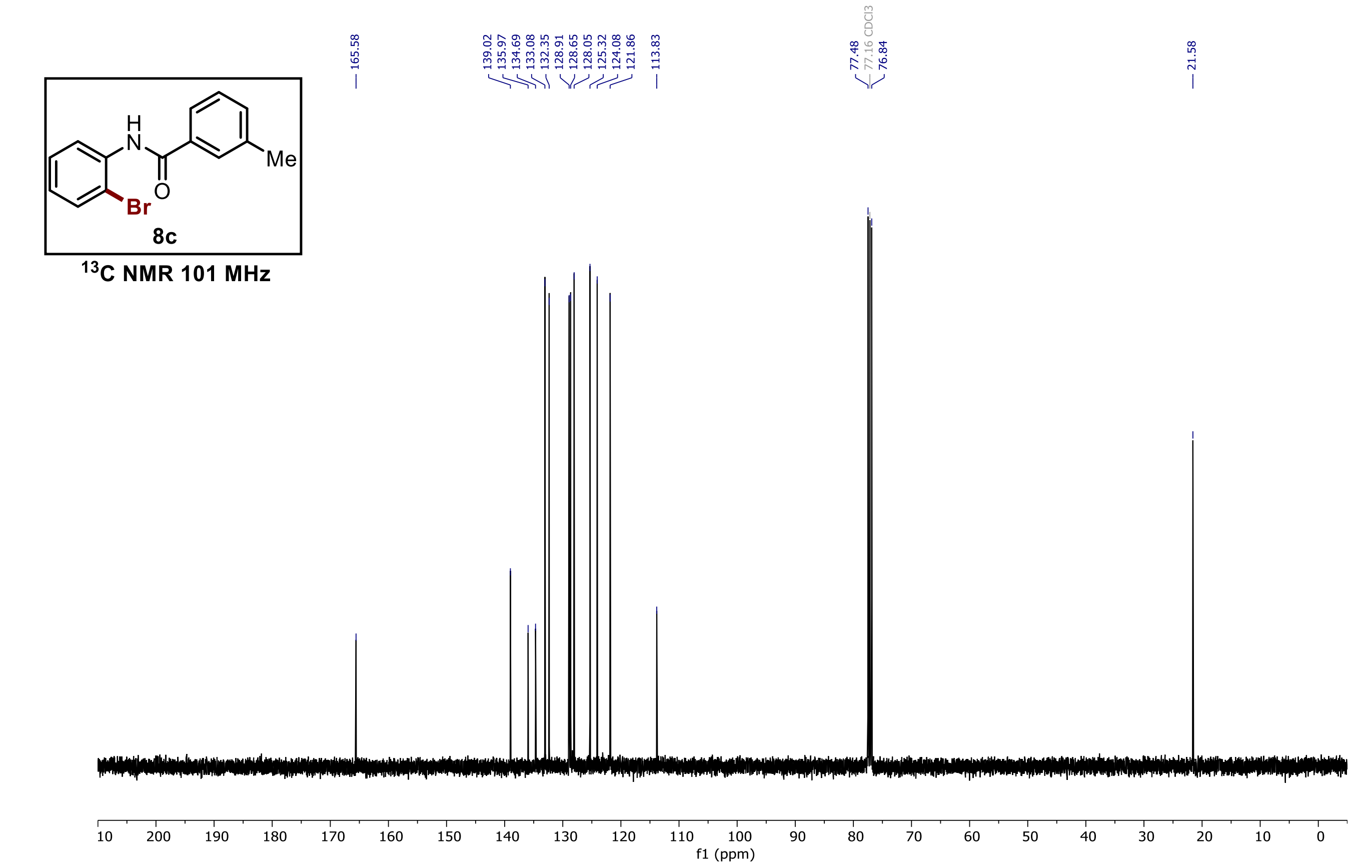
165.58
139.02
135.97
134.69
133.08
132.35
128.91
128.65
128.05
125.32
124.08
121.86
113.83
77.48
77.16 CDCl3
76.84
21.58
H
N
Me
O
Br
8c
^{13}C NMR 101 MHz
f1 (ppm)

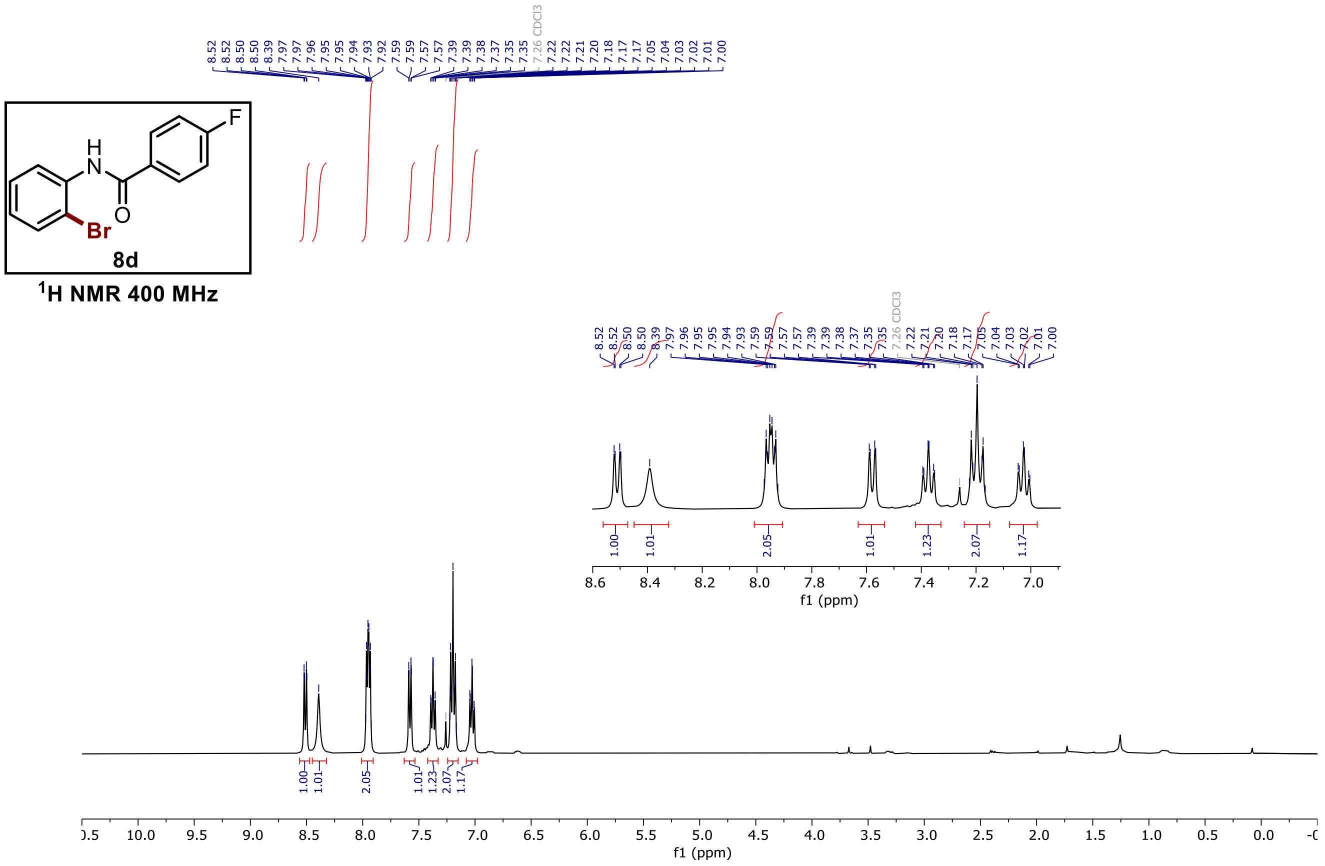
8.52
8.52
8.50
8.50
8.39
7.97
7.96
7.95
7.95
7.94
7.93
7.92
7.59
7.59
7.57
7.57
7.39
7.39
7.38
7.37
7.35
7.35
7.26 CDCl3
7.22
7.22
7.21
7.20
7.18
7.17
7.17
7.05
7.04
7.03
7.02
7.01
7.00
H
N
F
O
Br
8d
^{1}H NMR 400 MHz
1.00
1.01
2.05
1.01
1.23
2.07
1.17
f1 (ppm)

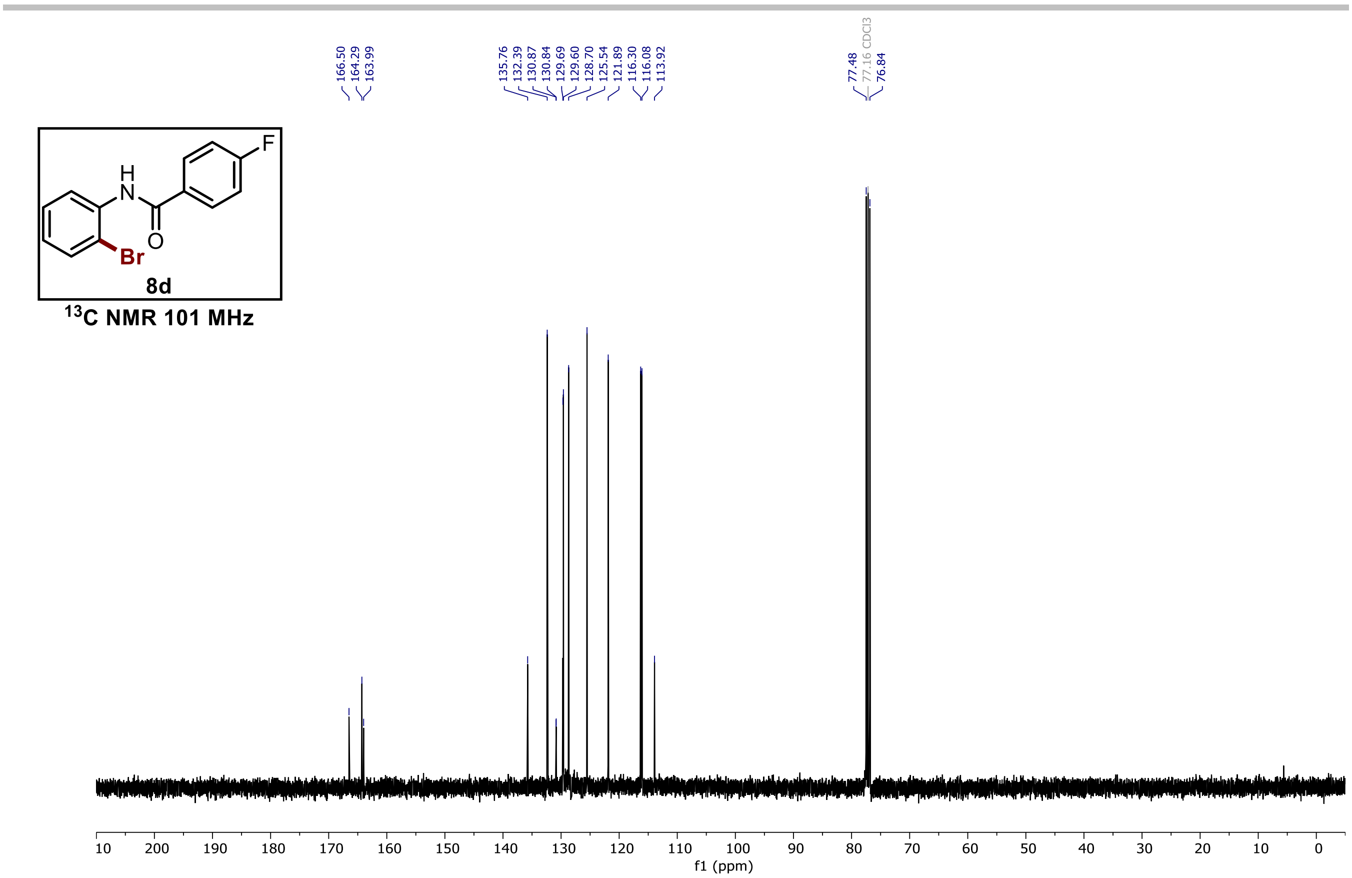
166.50
164.29
163.99
135.76
132.39
130.87
130.84
129.69
129.60
128.70
125.54
121.89
116.30
116.08
113.92
77.48
77.16 CDCl3
76.84
H
N
O
F
Br
8d
13C NMR 101 MHz
f1 (ppm)

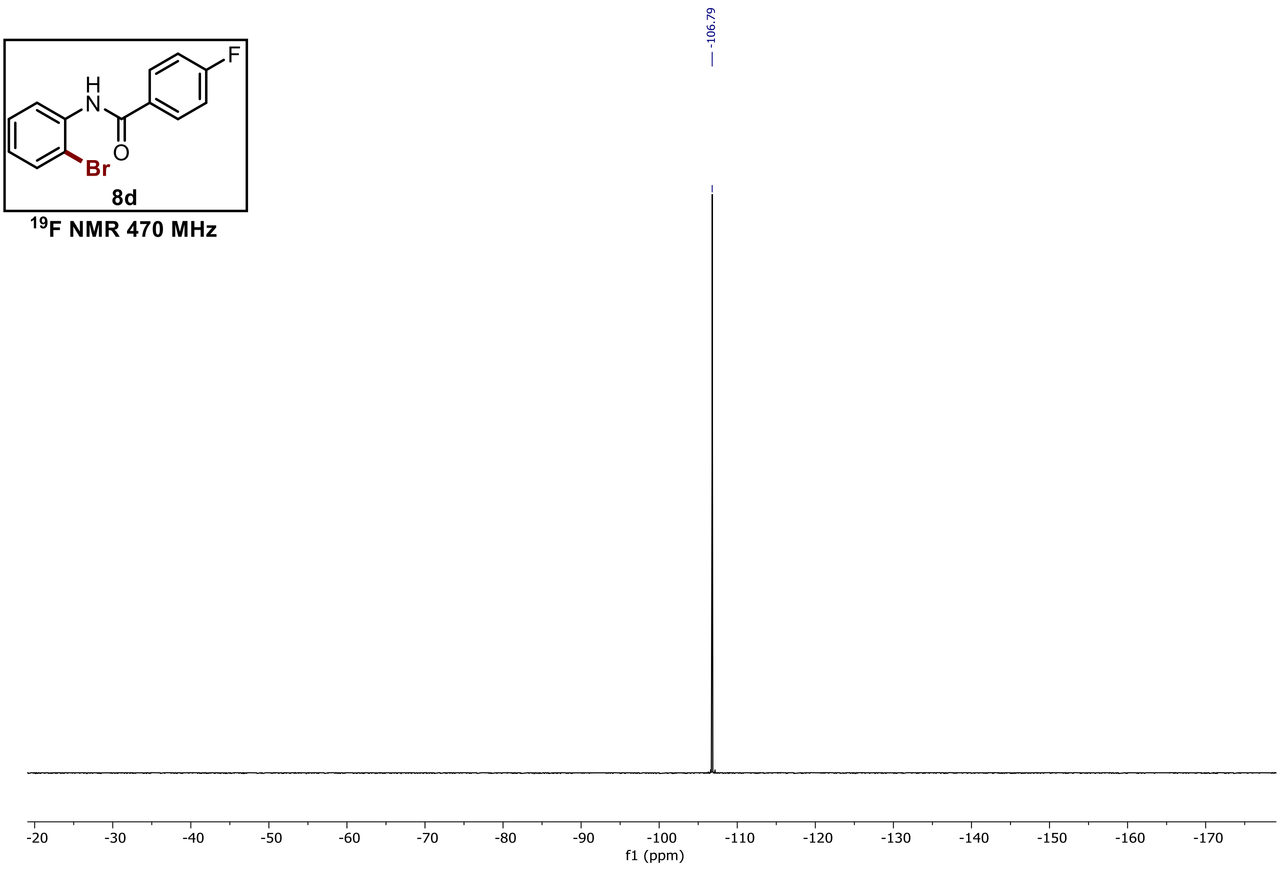
-106.79
H
N
O
F
Br
8d
19F NMR 470 MHz
f1 (ppm)

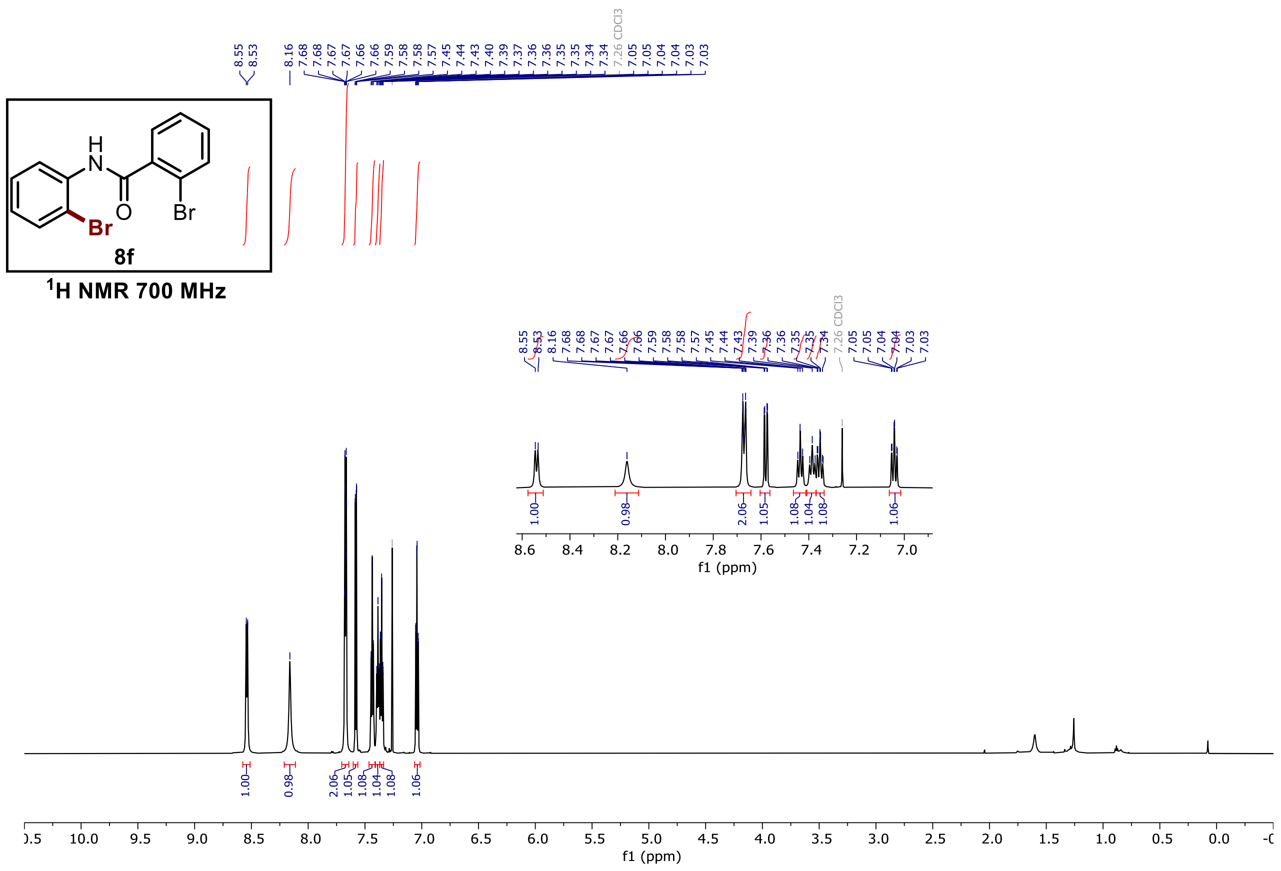
8f
1H NMR 700 MHz
f1 (ppm)

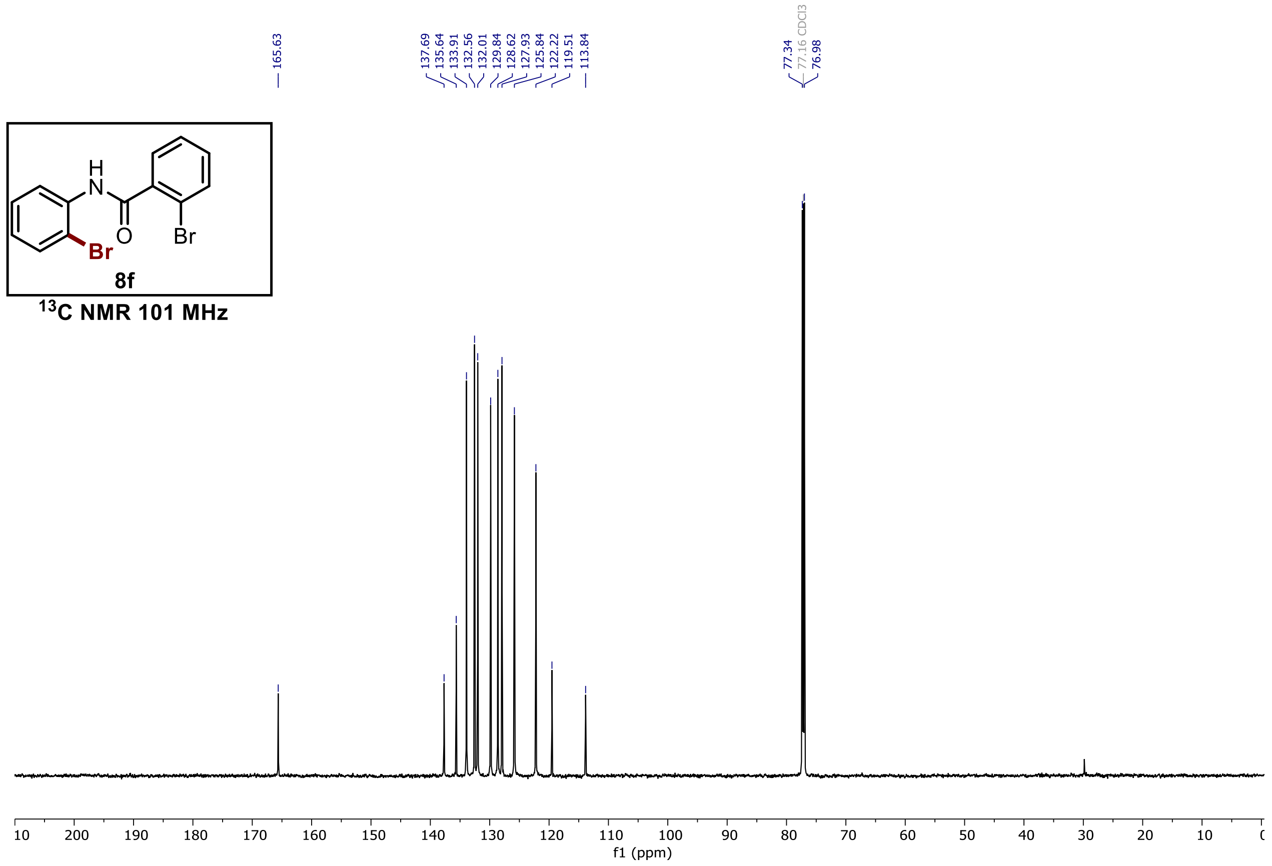
8f
13C NMR 101 MHz
f1 (ppm)

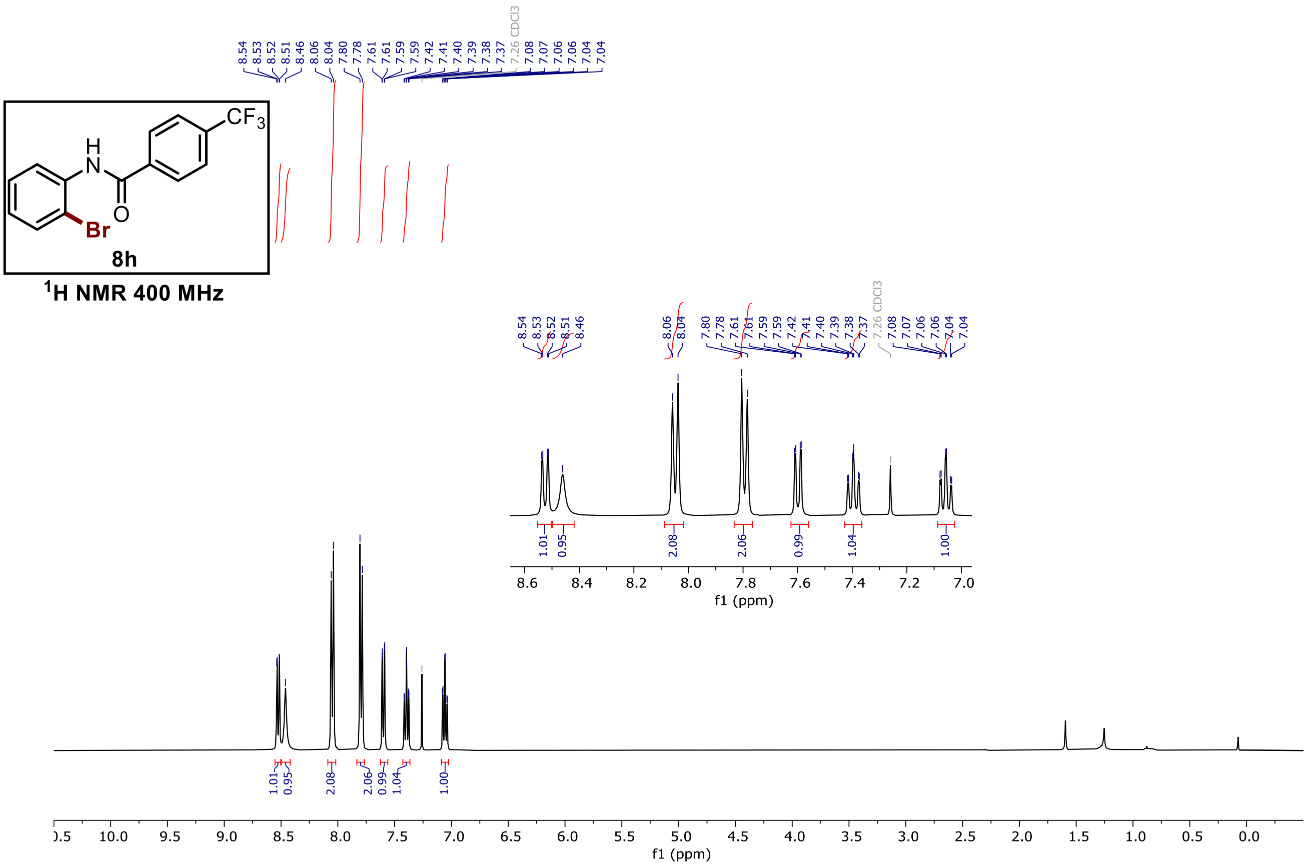

8h

^{1}H NMR 400 MHz

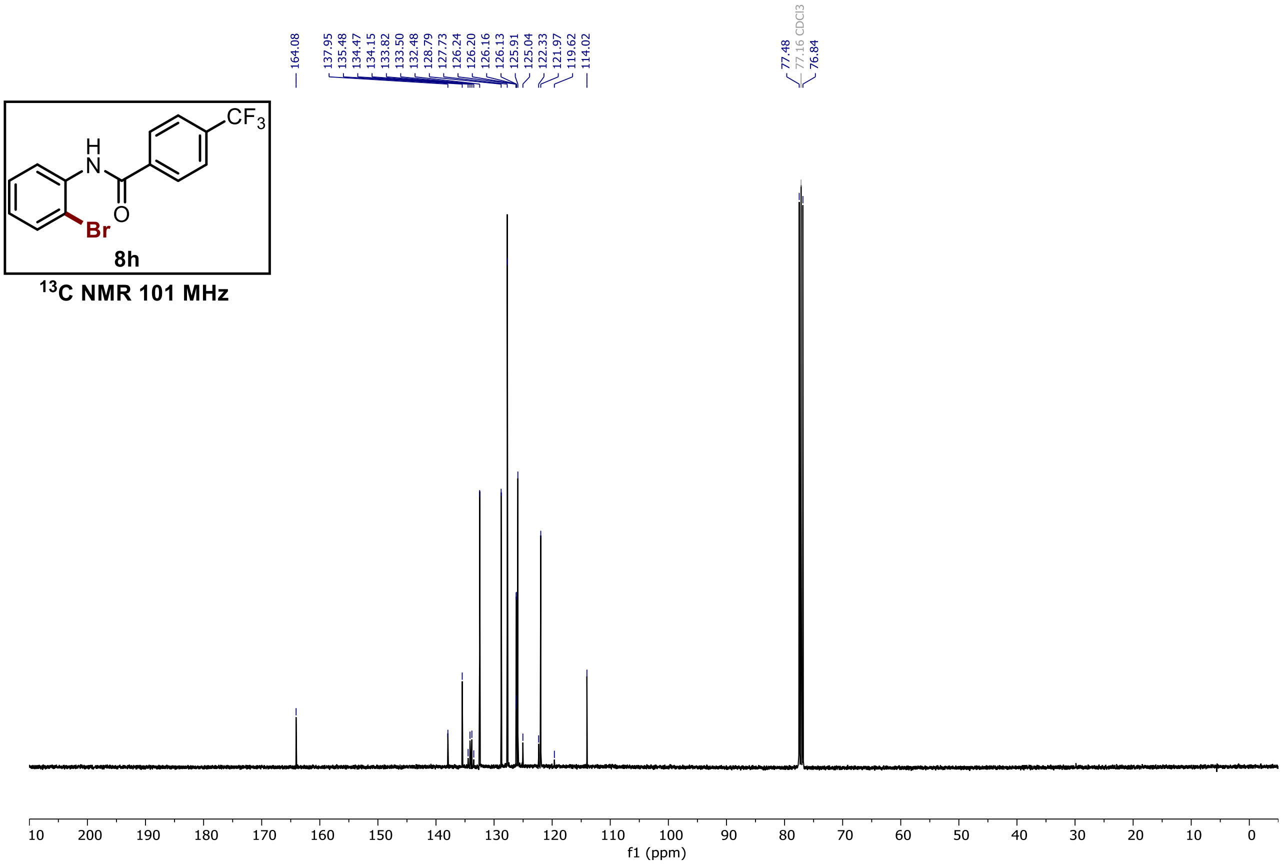

8h

^{13}C NMR 101 MHz

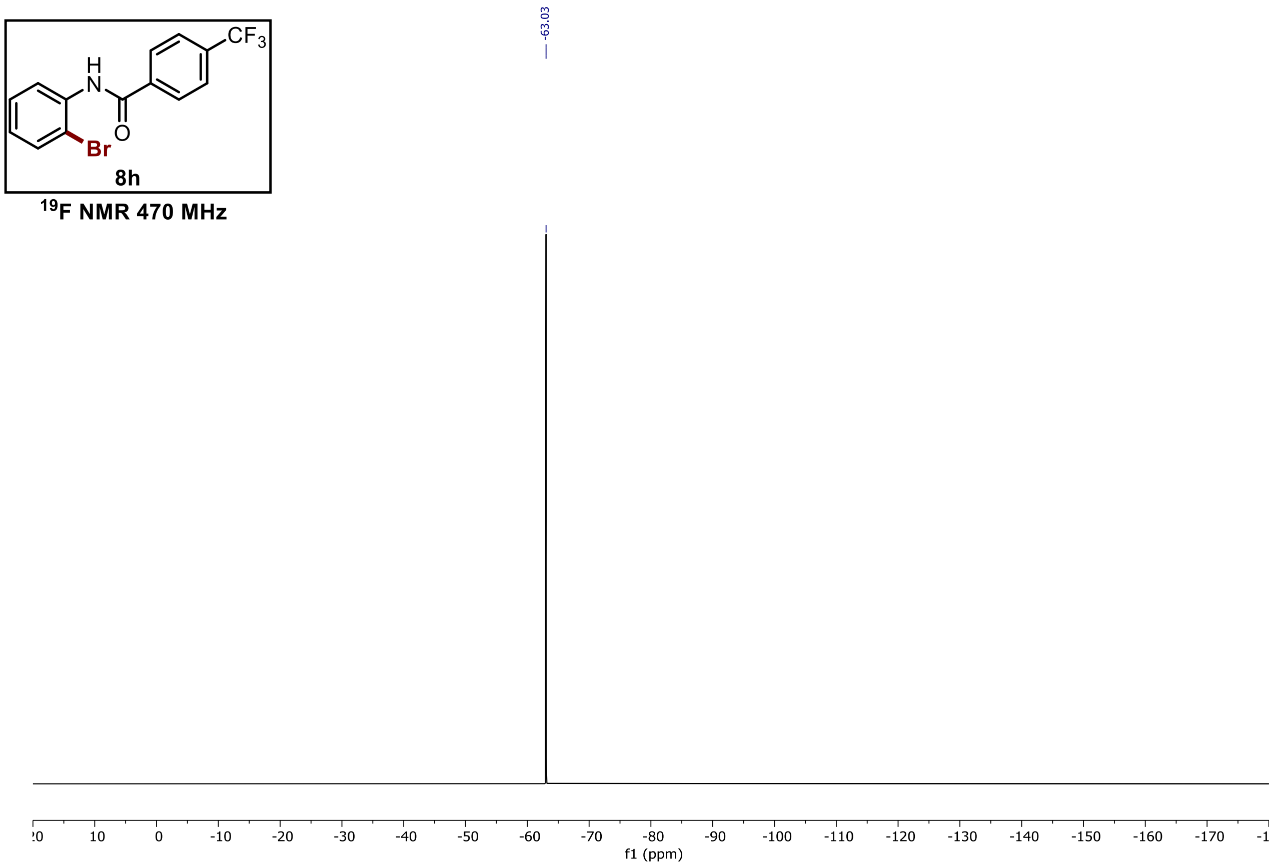

8h
^{19}F NMR 470 MHz
-63.03
f1 (ppm)

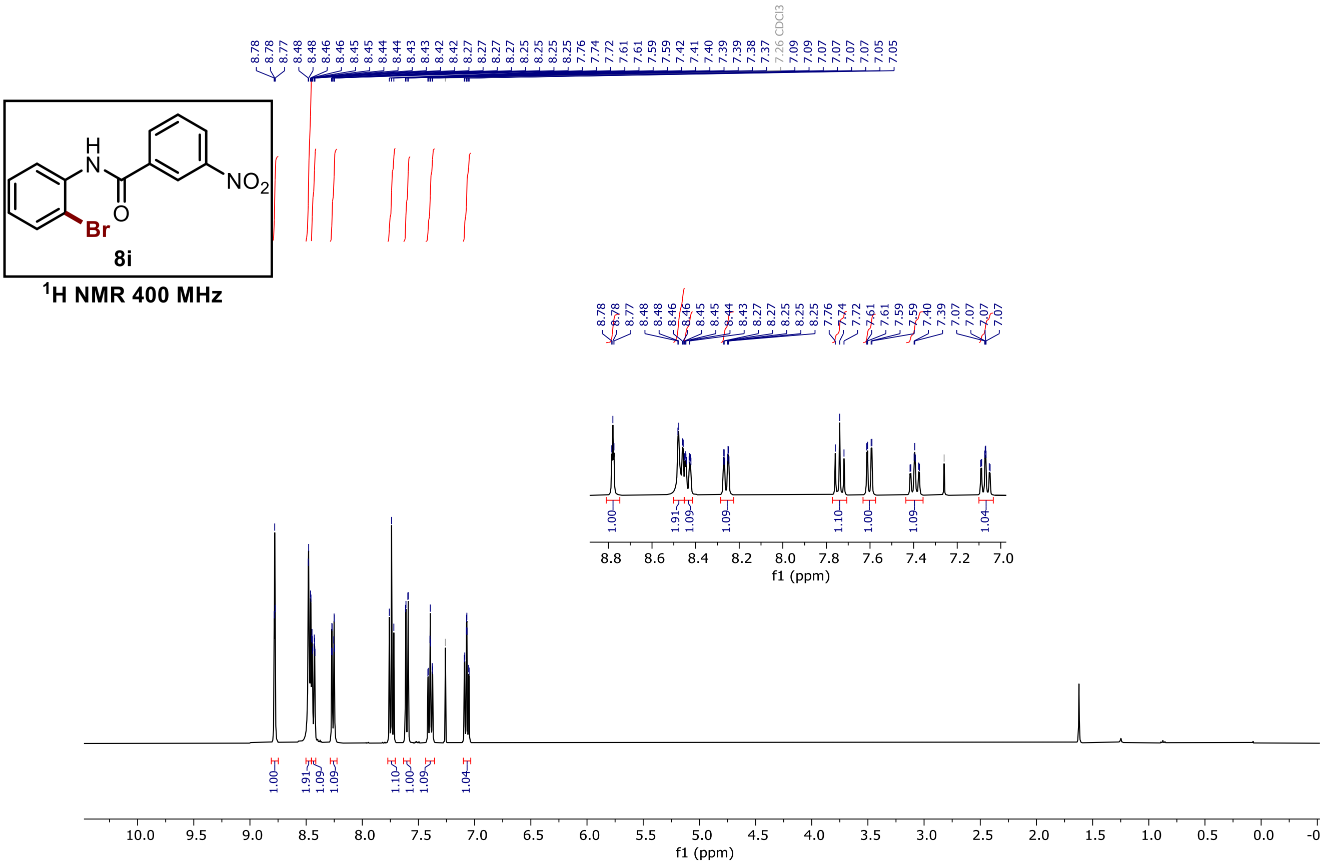

8i
^{1}H NMR 400 MHz
7.26 CDCl3
f1 (ppm)

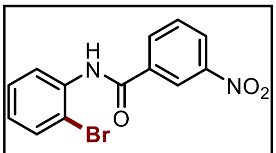

8i

^{13}C NMR 101 MHz

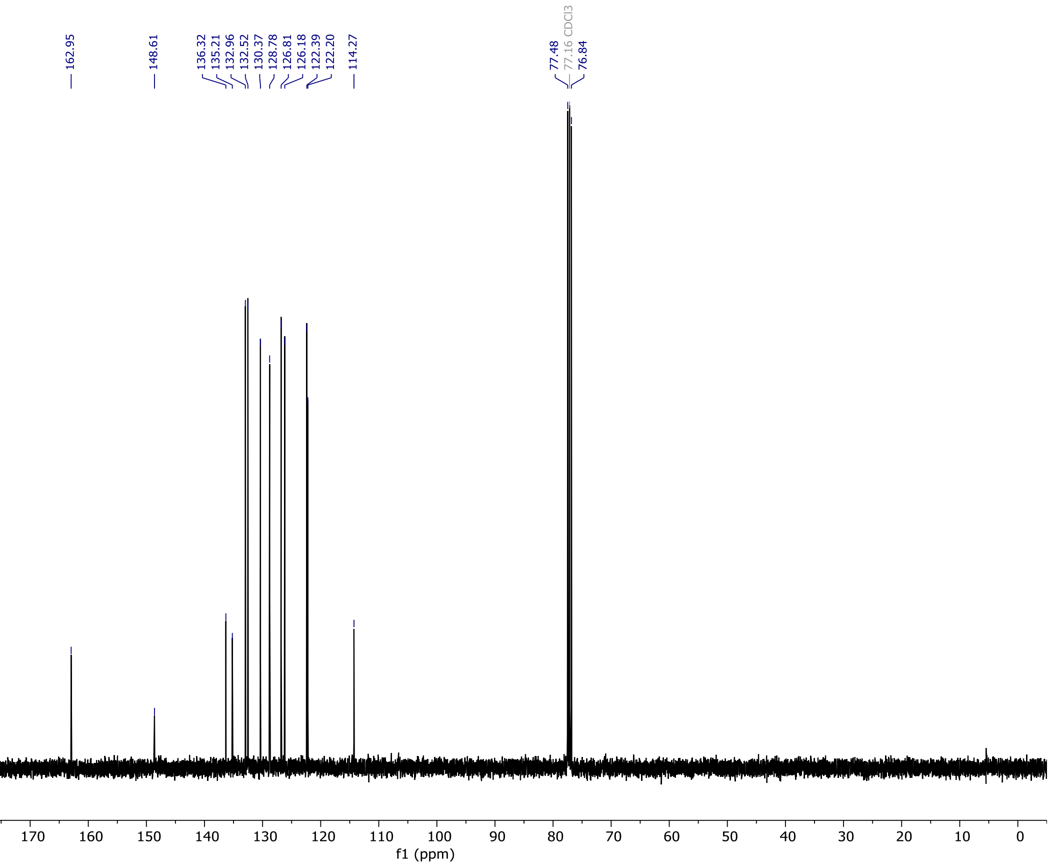

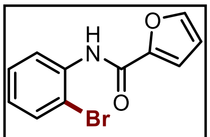

8j

^{1}H NMR 400 MHz

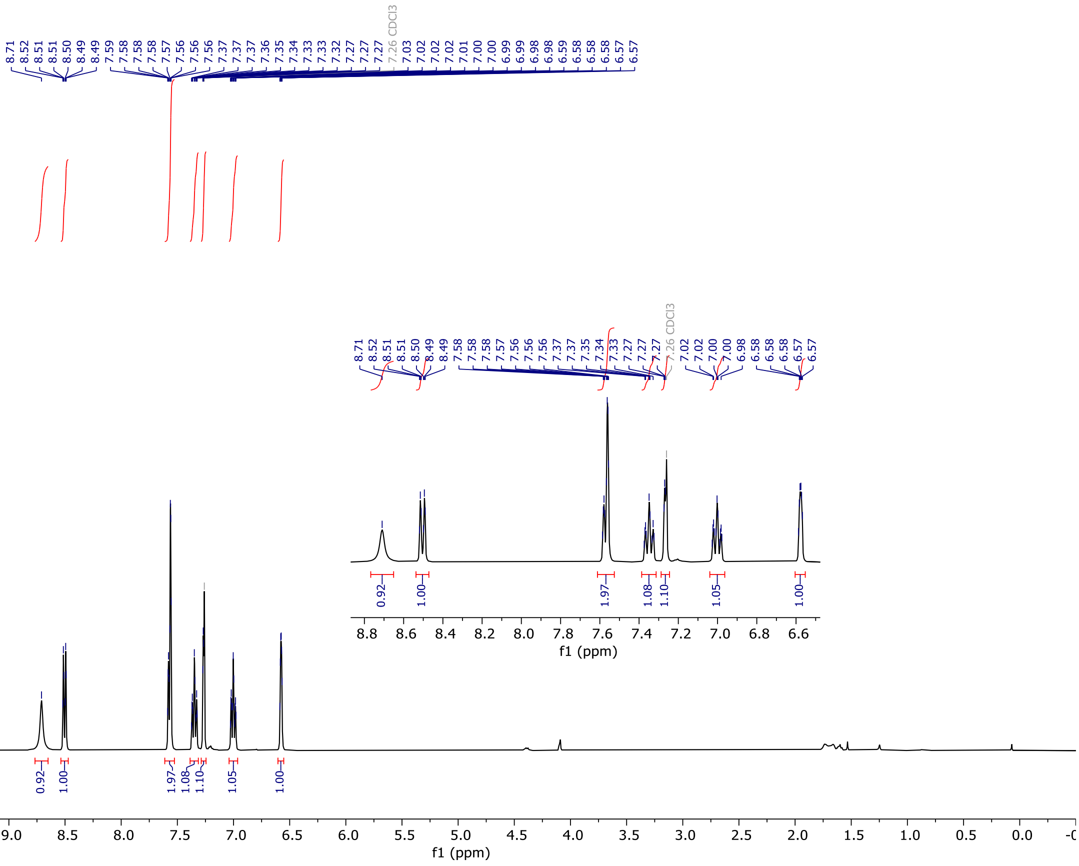

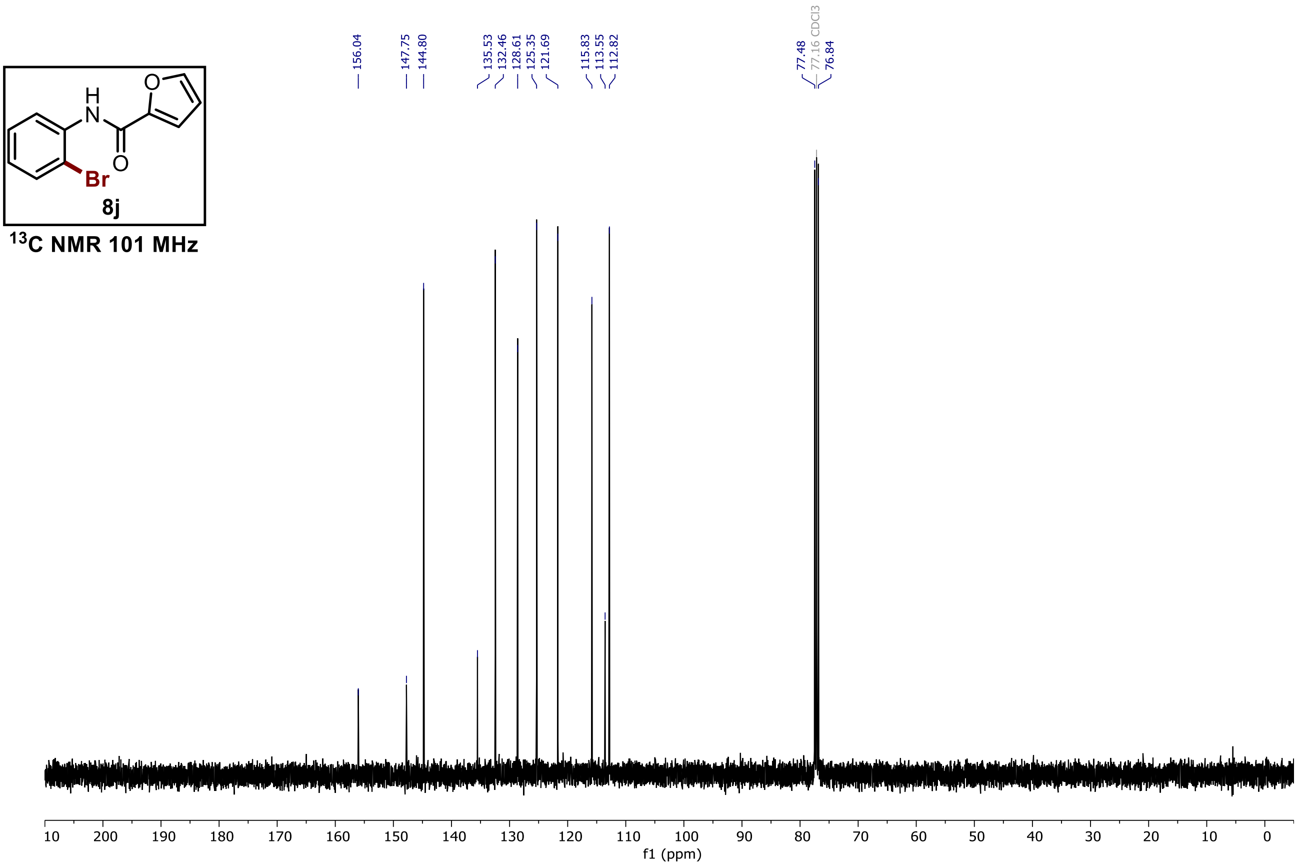

8j
^{13}C NMR 101 MHz
156.04
147.75
144.80
135.53
132.46
128.61
125.35
121.69
115.83
113.55
112.82
77.48
77.16 CDCl3
76.84
f1 (ppm)

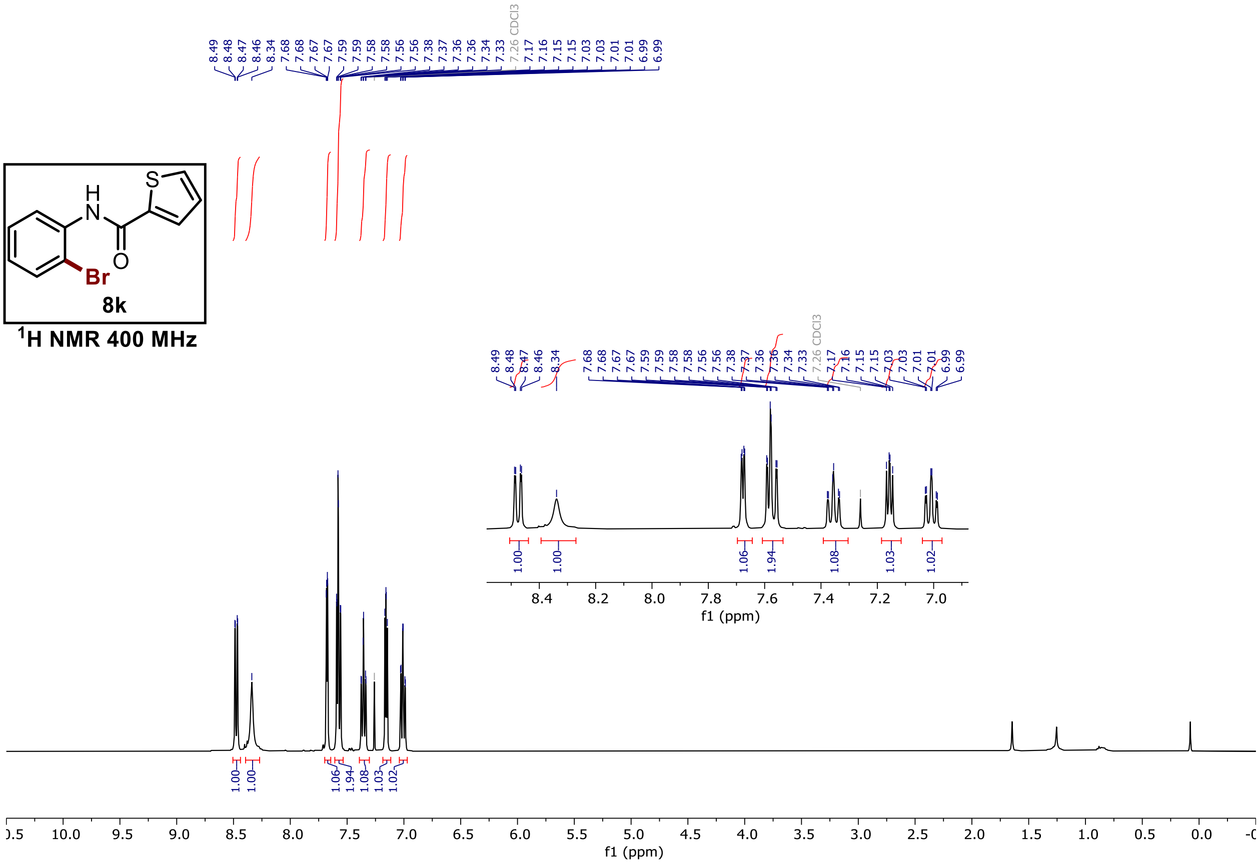

8k
^{1}H NMR 400 MHz
8.49
8.48
8.47
8.46
8.34
7.68
7.68
7.67
7.67
7.59
7.59
7.58
7.58
7.56
7.56
7.38
7.37
7.36
7.36
7.34
7.33
7.26 CDCl3
7.17
7.16
7.15
7.15
7.03
7.03
7.01
7.01
6.99
6.99
1.00
1.00
1.06
1.94
1.08
1.03
1.02
f1 (ppm)

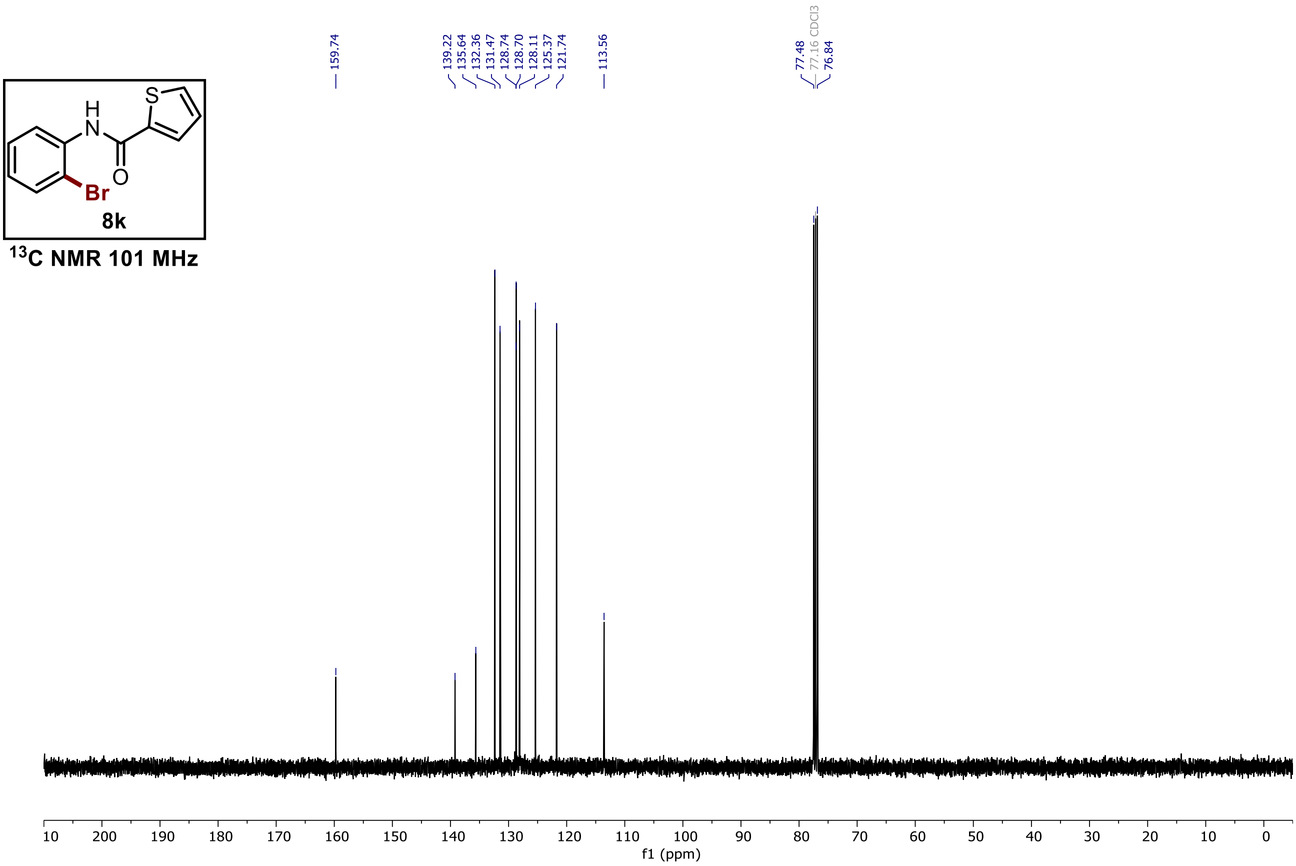
8k
13C NMR 101 MHz
159.74
139.22
135.64
132.36
131.47
128.74
128.70
128.11
125.37
121.74
113.56
77.48
77.16 CDCl3
76.84
f1 (ppm)

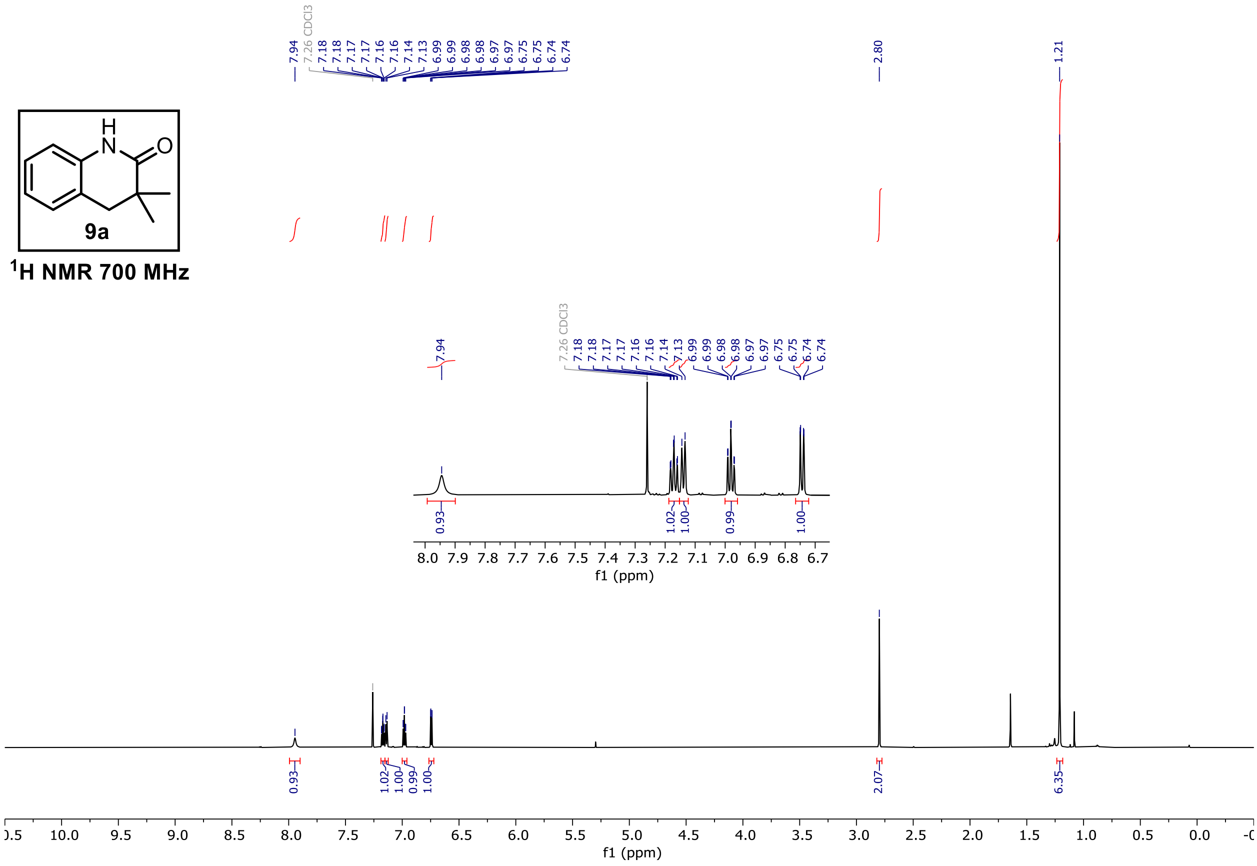
9a
1H NMR 700 MHz
7.94
7.26 CDCl3
7.18
7.18
7.17
7.17
7.16
7.16
7.14
7.13
6.99
6.99
6.98
6.98
6.97
6.97
6.75
6.75
6.74
6.74
2.80
1.21
0.93
1.02
1.00
0.99
1.00
2.07
6.35
f1 (ppm)

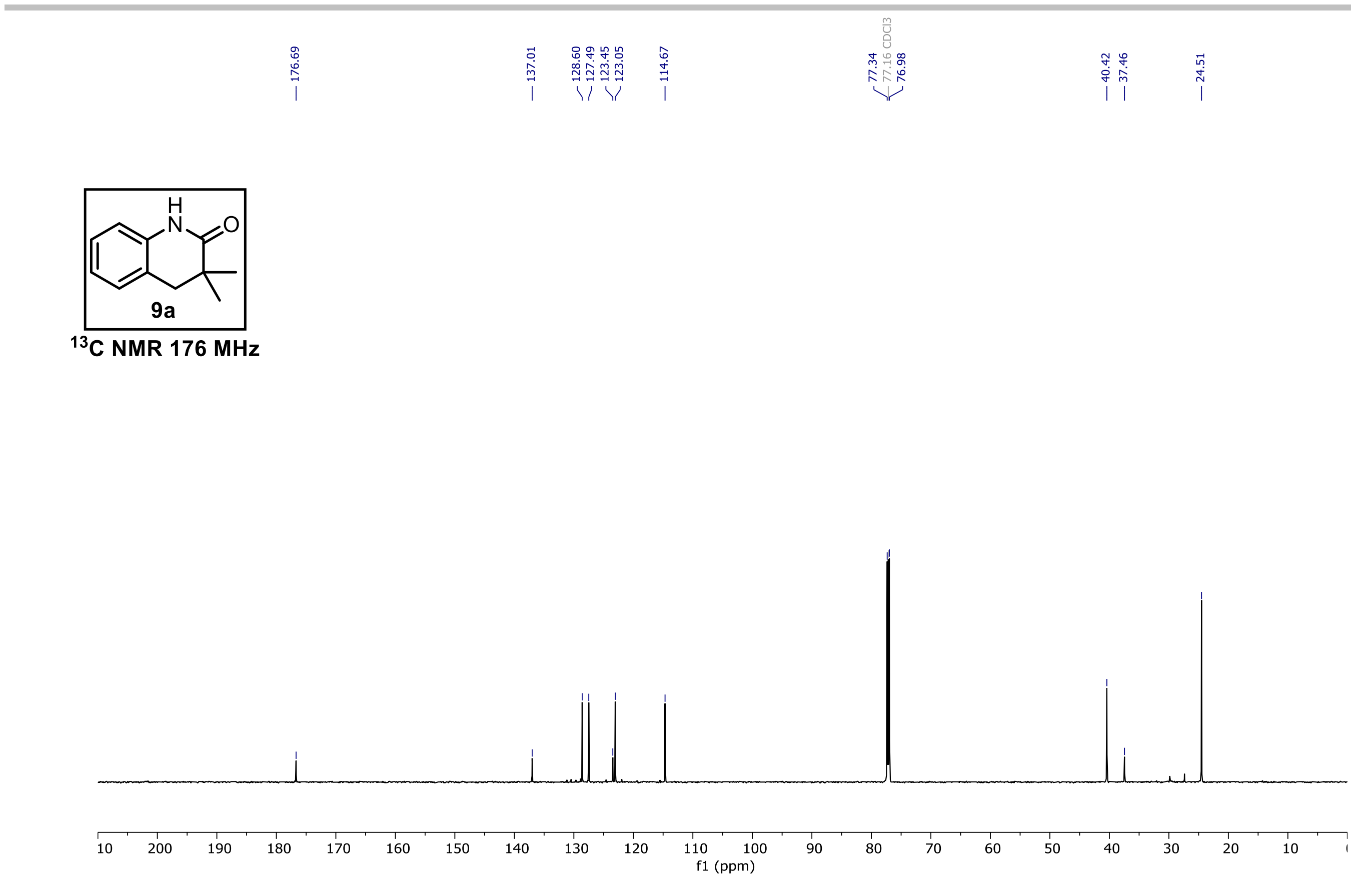
9a
13C NMR 176 MHz
176.69
137.01
128.60
127.49
123.45
123.05
114.67
77.34
77.16 CDCl3
76.98
40.42
37.46
24.51
f1 (ppm)

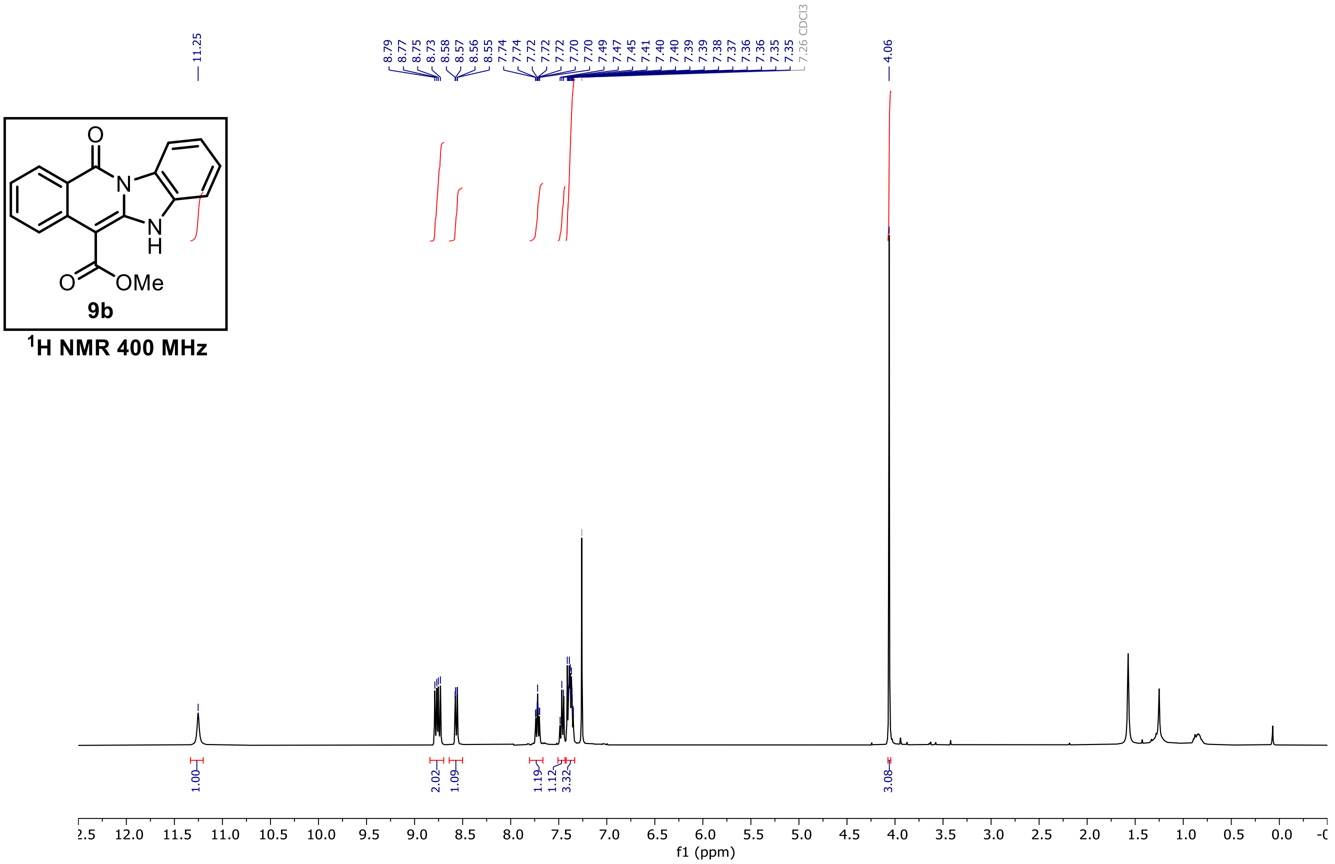
9b
1H NMR 400 MHz
11.25
8.79
8.77
8.75
8.73
8.58
8.57
8.56
8.55
7.74
7.74
7.72
7.72
7.72
7.70
7.70
7.49
7.47
7.45
7.41
7.40
7.40
7.39
7.39
7.38
7.37
7.36
7.35
7.35
7.26 CDCl3
4.06
1.00
2.02
1.09
1.19
1.12
3.32
3.08
f1 (ppm)

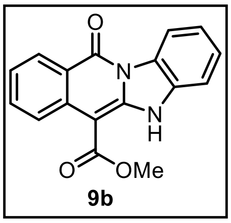

^{13}C NMR 101 MHz

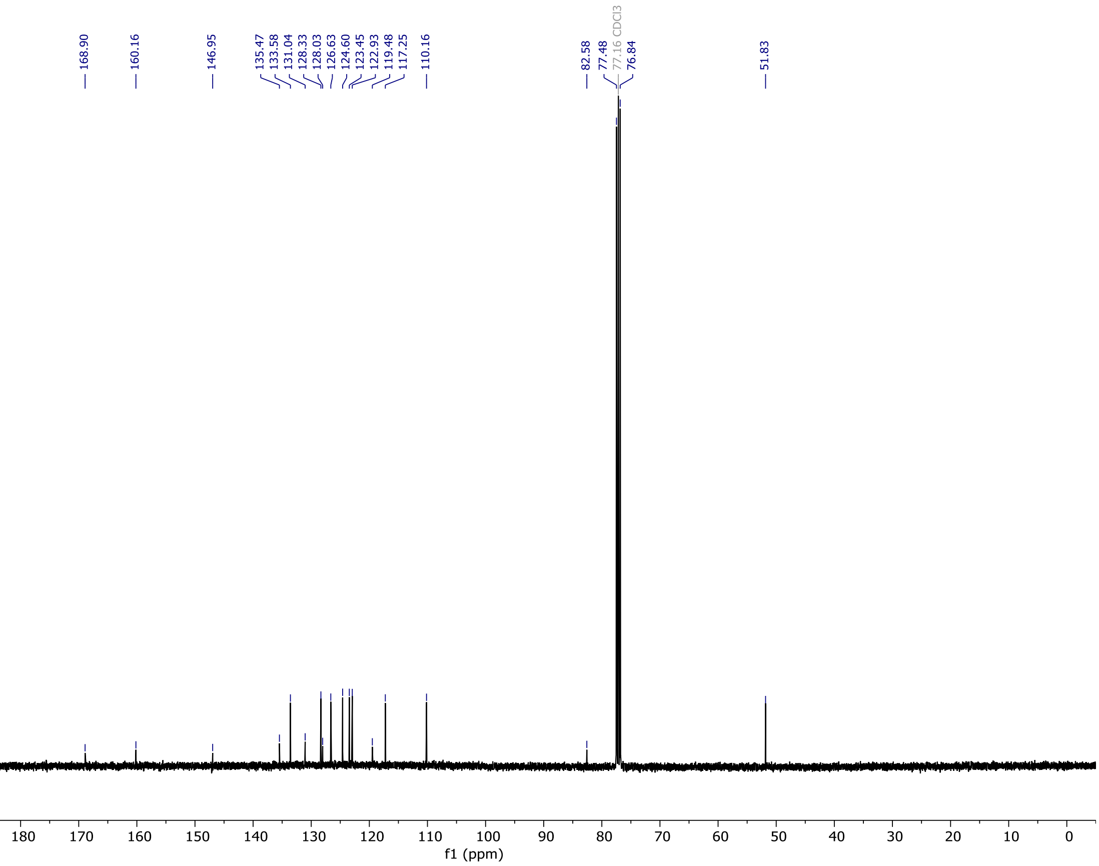

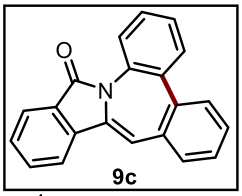

^{1}H NMR 400 MHz

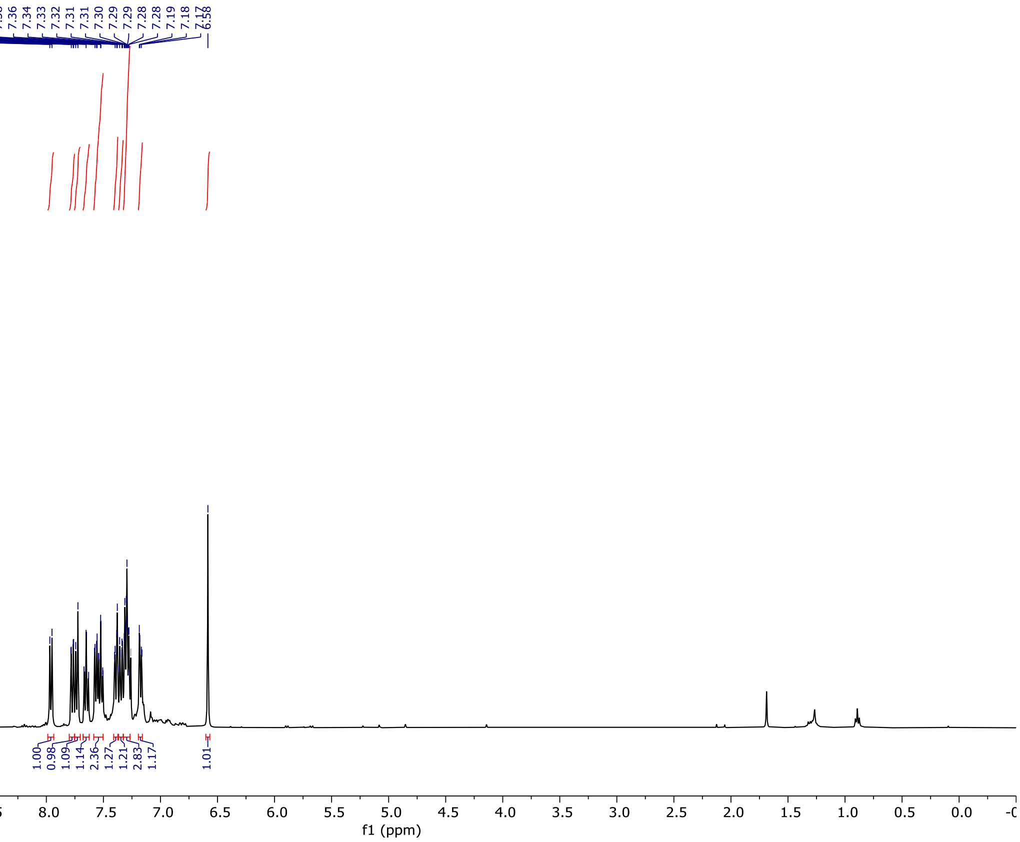

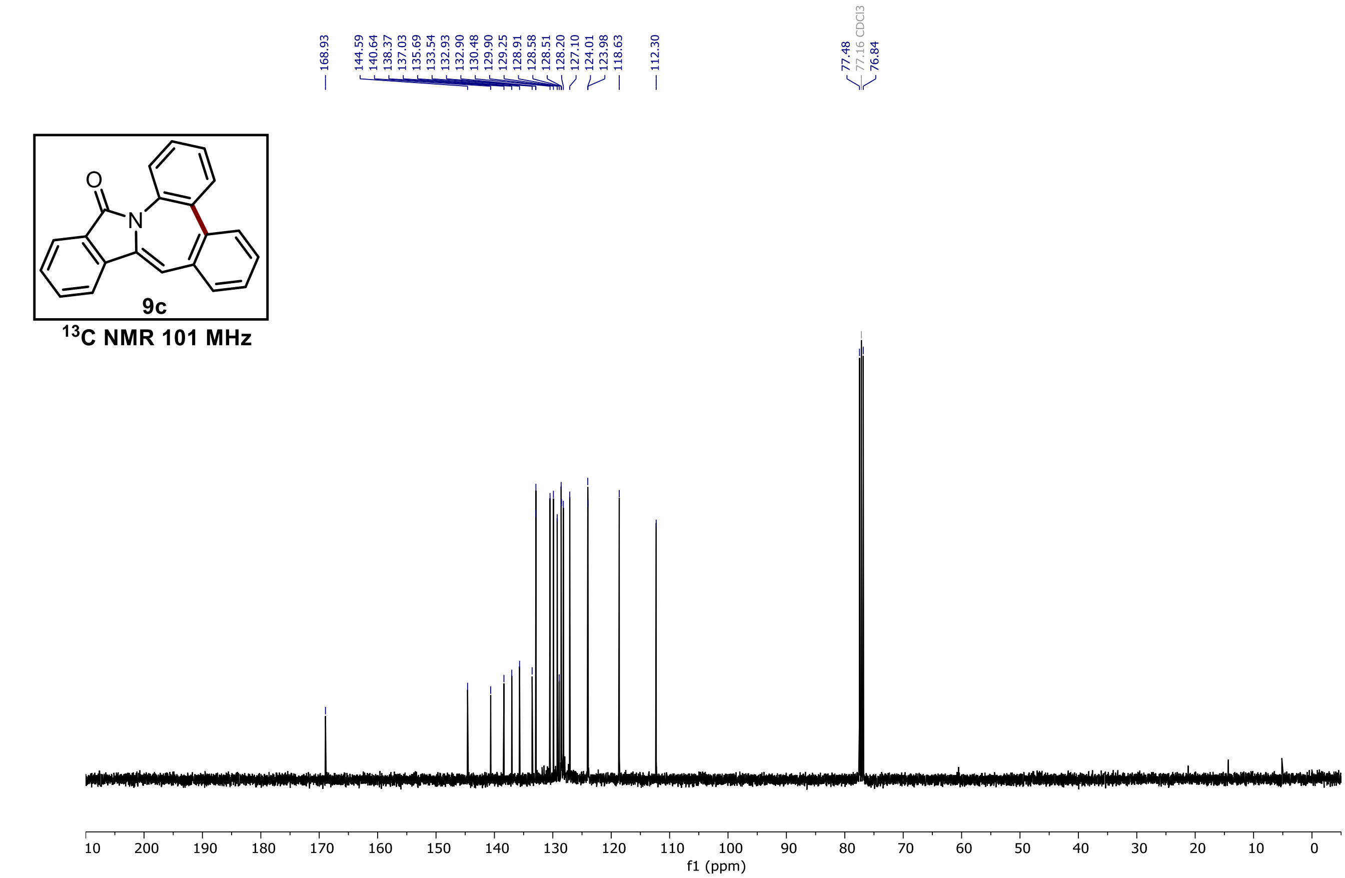

9c

^{13}C NMR 101 MHz

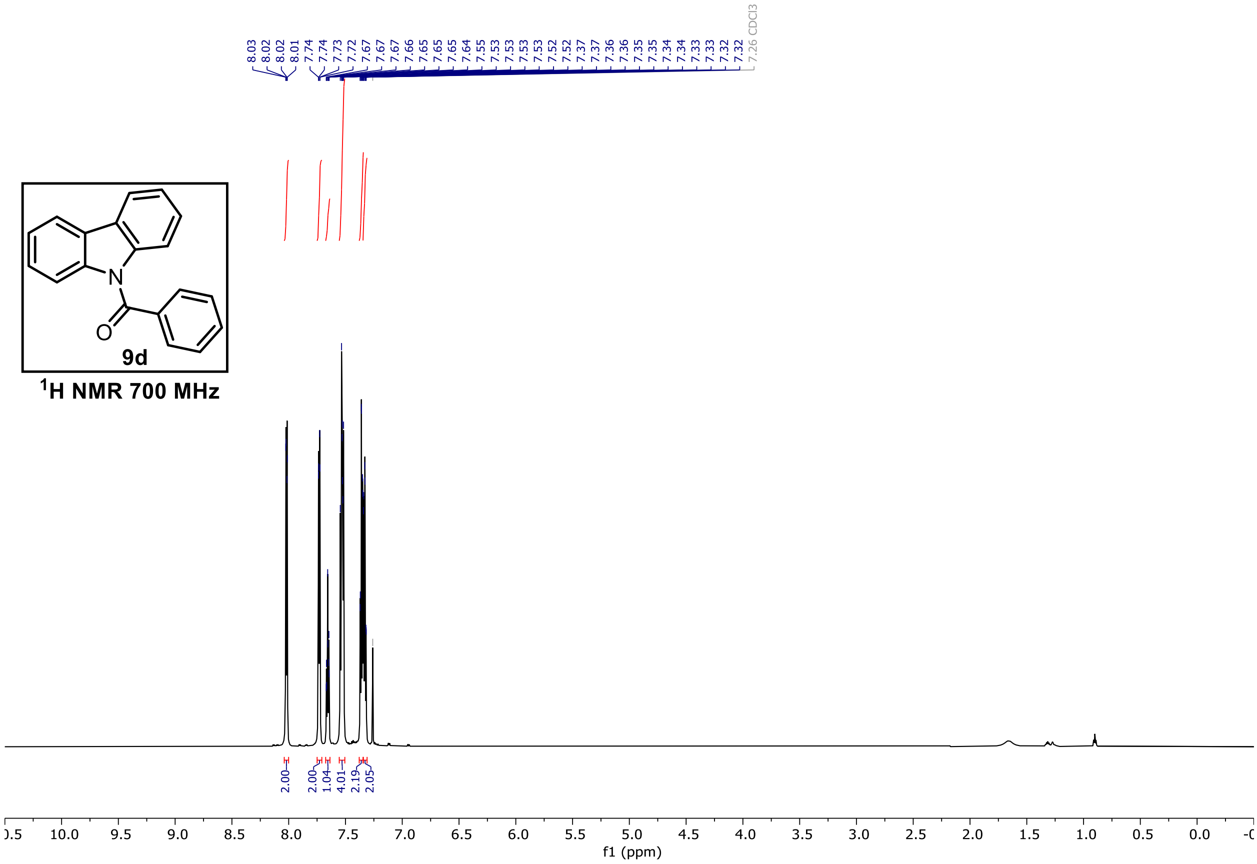

9d

^{1}H NMR 700 MHz

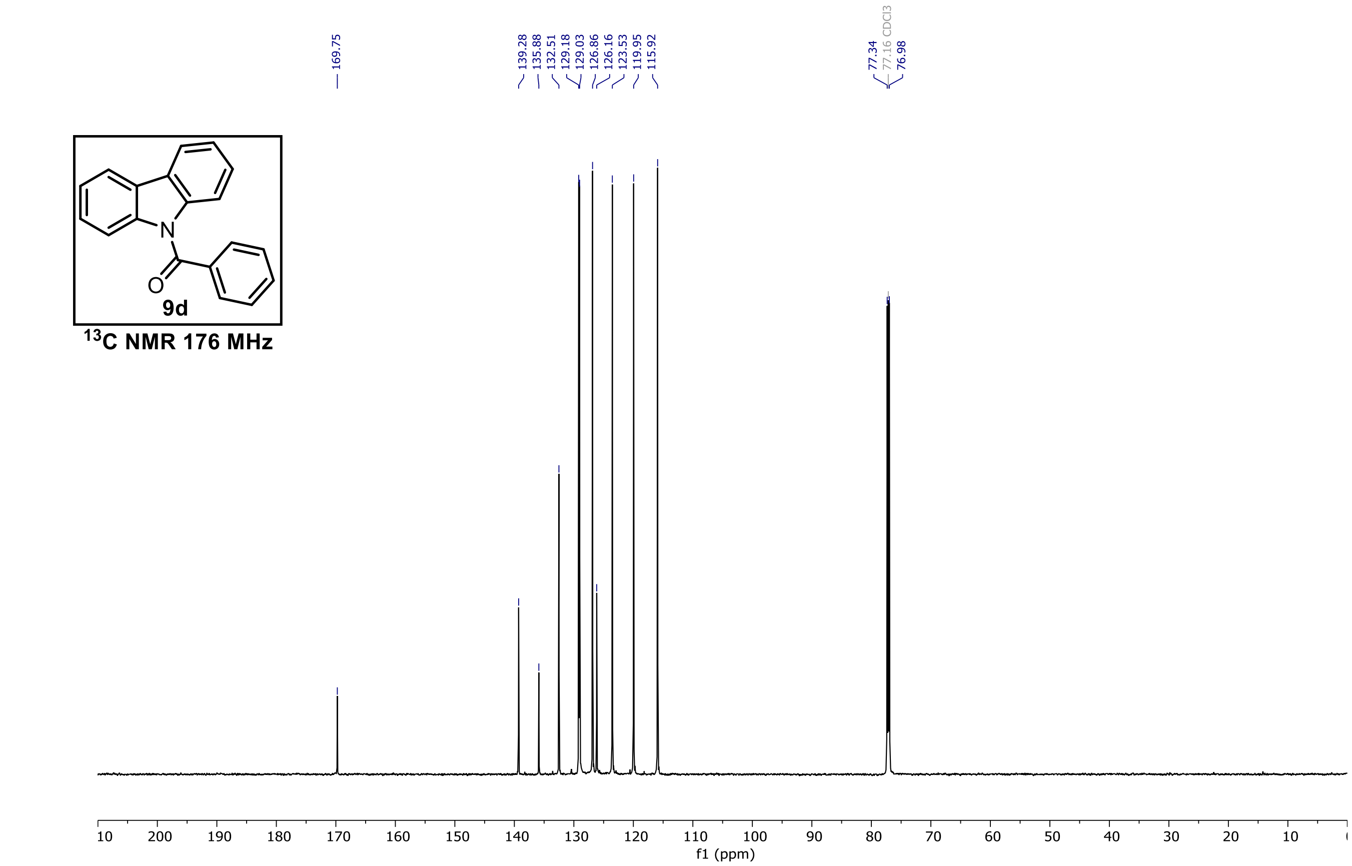
9d
13C NMR 176 MHz
f1 (ppm)

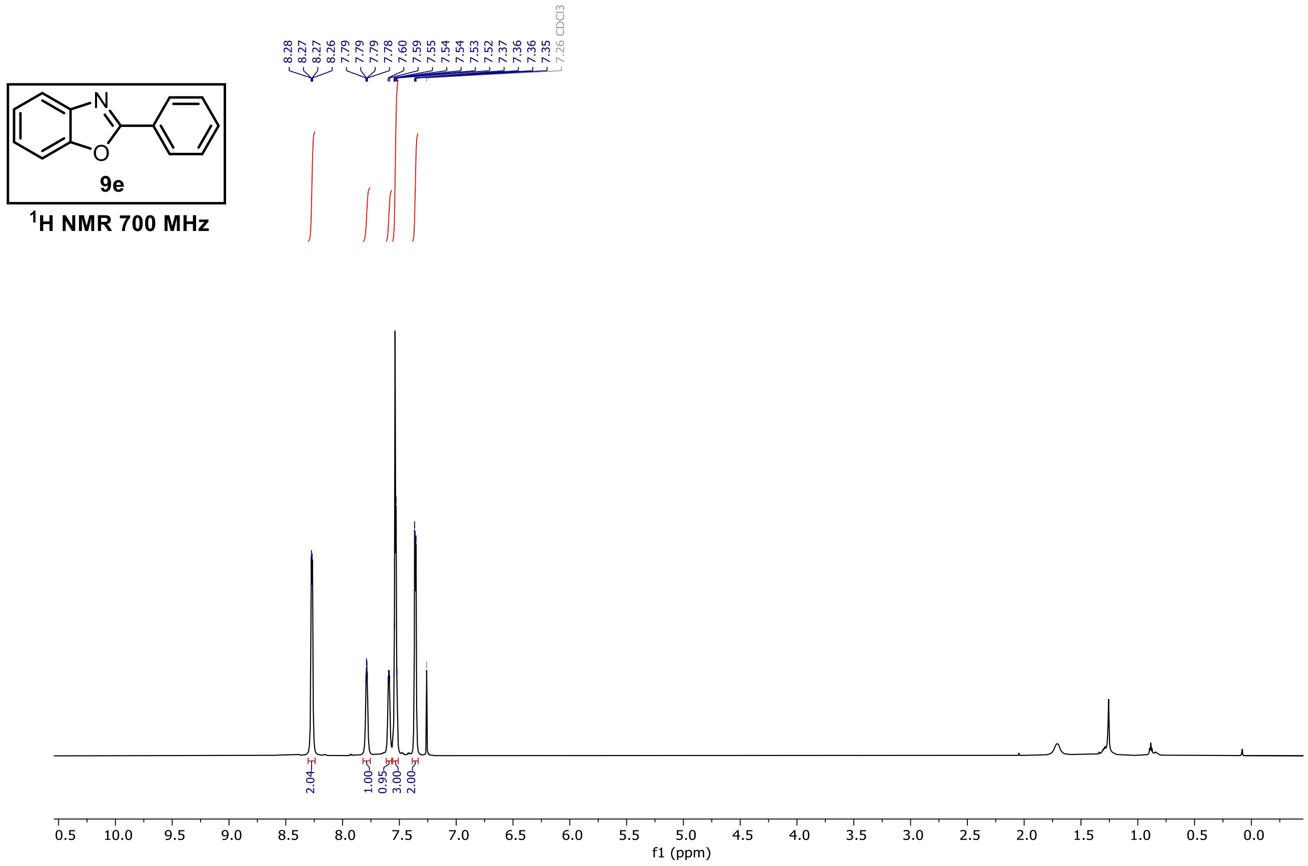
9e
1H NMR 700 MHz
f1 (ppm)

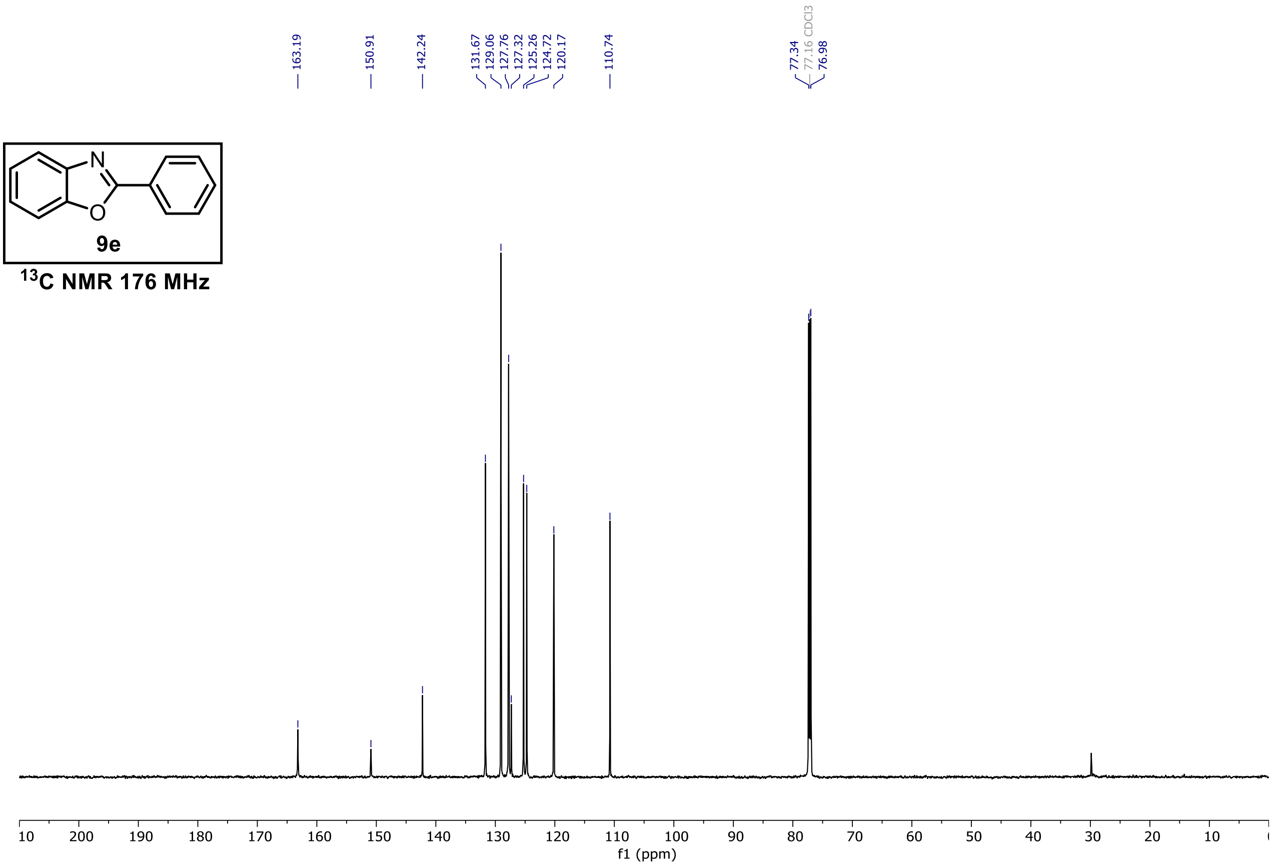
163.19
150.91
142.24
131.67
129.06
127.76
127.32
125.26
124.72
120.17
110.74
77.34
77.16 CDCl3
76.98
N
O
9e
^{13}C NMR 176 MHz
f1 (ppm)

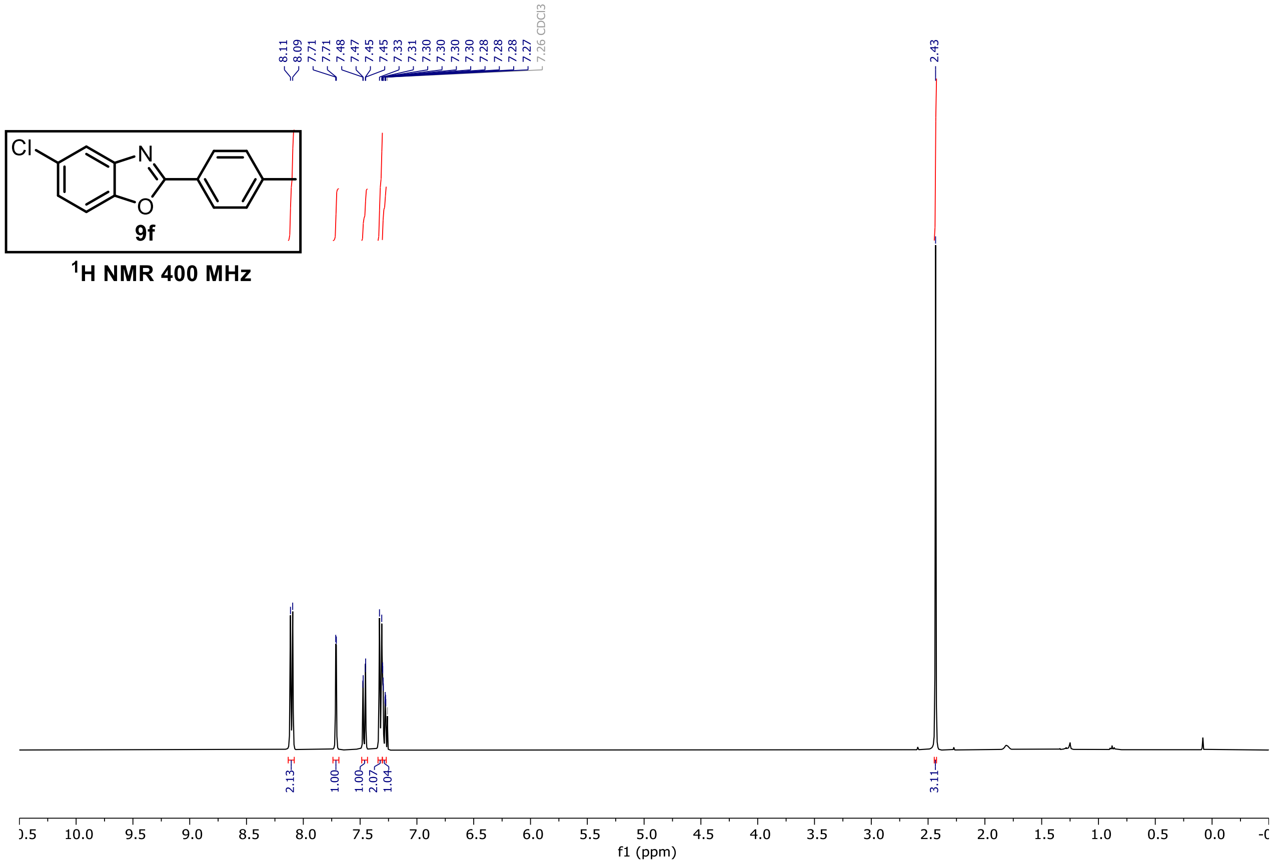
8.11
8.09
7.71
7.71
7.48
7.47
7.45
7.45
7.33
7.31
7.30
7.30
7.30
7.30
7.28
7.28
7.28
7.27
7.26 CDCl3
2.43
Cl
N
O
9f
^{1}H NMR 400 MHz
2.13
1.00
1.00
2.07
1.04
3.11
f1 (ppm)

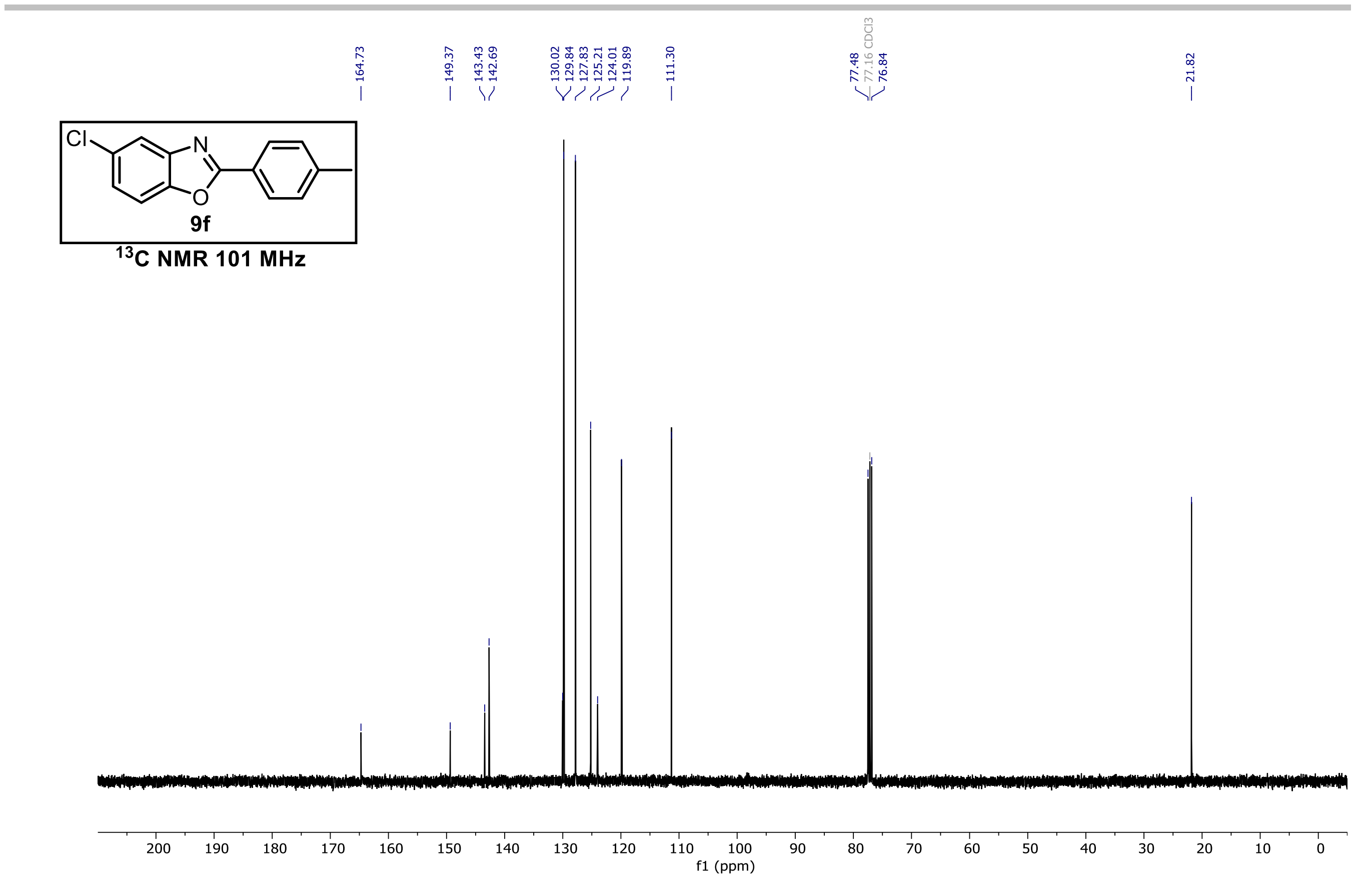

164.73
149.37
143.43
142.69
130.02
129.84
127.83
125.21
124.01
119.89
111.30
77.48
77.16 CDCl3
76.84
21.82
Cl
N
O
9f
13C NMR 101 MHz
f1 (ppm)

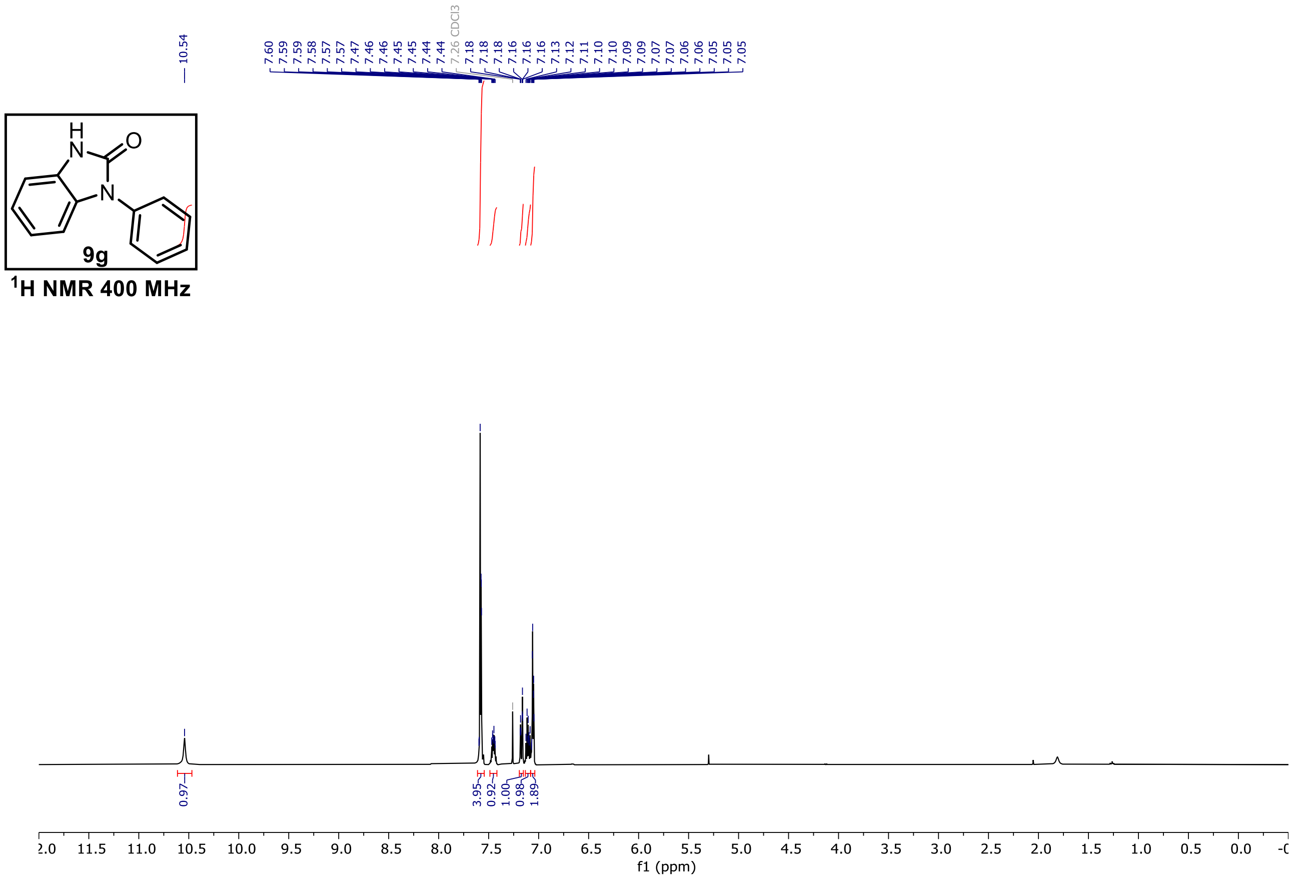

10.54
7.60
7.59
7.59
7.58
7.57
7.57
7.47
7.46
7.45
7.45
7.44
7.44
7.26 CDCl3
7.18
7.18
7.18
7.16
7.16
7.16
7.13
7.12
7.11
7.10
7.10
7.09
7.07
7.07
7.06
7.06
7.05
7.05
7.05
H
N
O
N
9g
1H NMR 400 MHz
0.97
3.95
0.92
1.00
0.98
1.89
f1 (ppm)

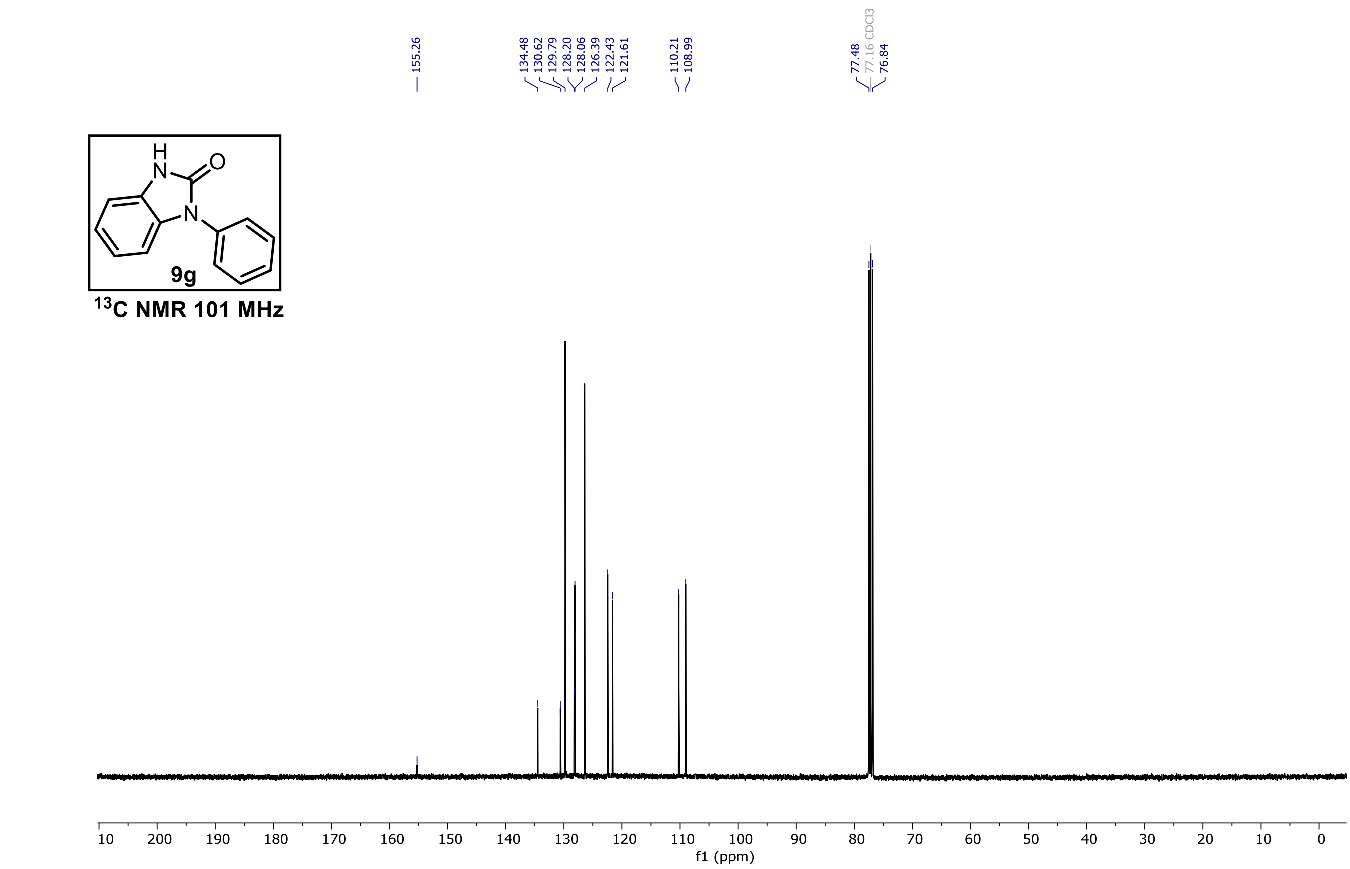
155.26
134.48
130.62
129.79
128.20
128.06
126.39
122.43
121.61
110.21
108.99
77.48
77.16 CDCl3
76.84
9g
^{13}C NMR 101 MHz
f1 (ppm)

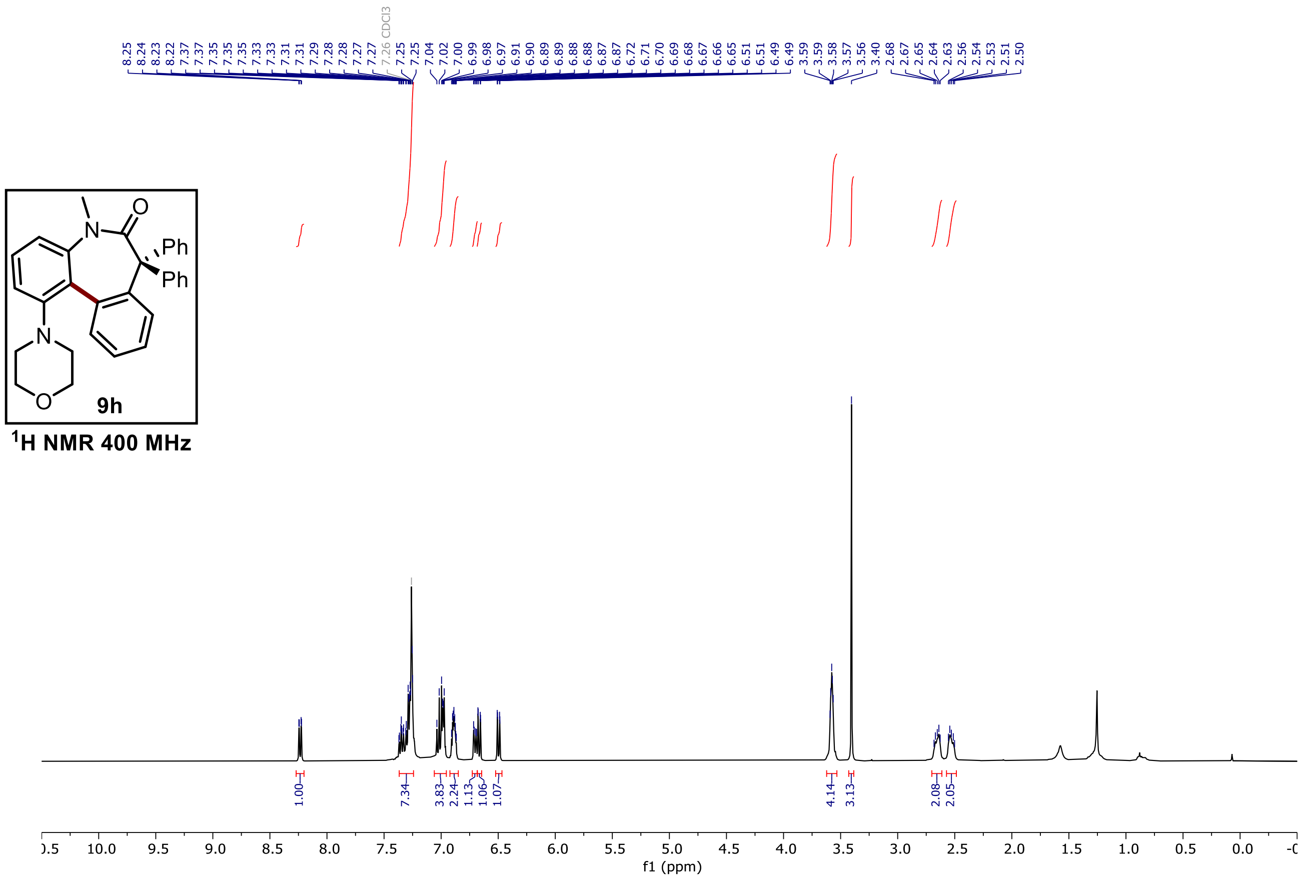
8.25
8.24
8.23
8.22
7.37
7.37
7.35
7.35
7.35
7.33
7.33
7.31
7.31
7.29
7.28
7.28
7.27
7.27
7.26 CDCl3
7.25
7.25
7.04
7.02
7.00
6.99
6.98
6.97
6.91
6.90
6.89
6.89
6.88
6.88
6.87
6.87
6.72
6.71
6.70
6.69
6.68
6.67
6.66
6.51
6.51
6.49
6.49
3.59
3.59
3.58
3.57
3.56
3.40
2.68
2.67
2.65
2.64
2.63
2.56
2.54
2.53
2.51
2.50
Ph
Ph
9h
^{1}H NMR 400 MHz
1.00
7.34
3.83
2.24
1.13
1.06
1.07
4.14
3.13
2.08
2.05
f1 (ppm)

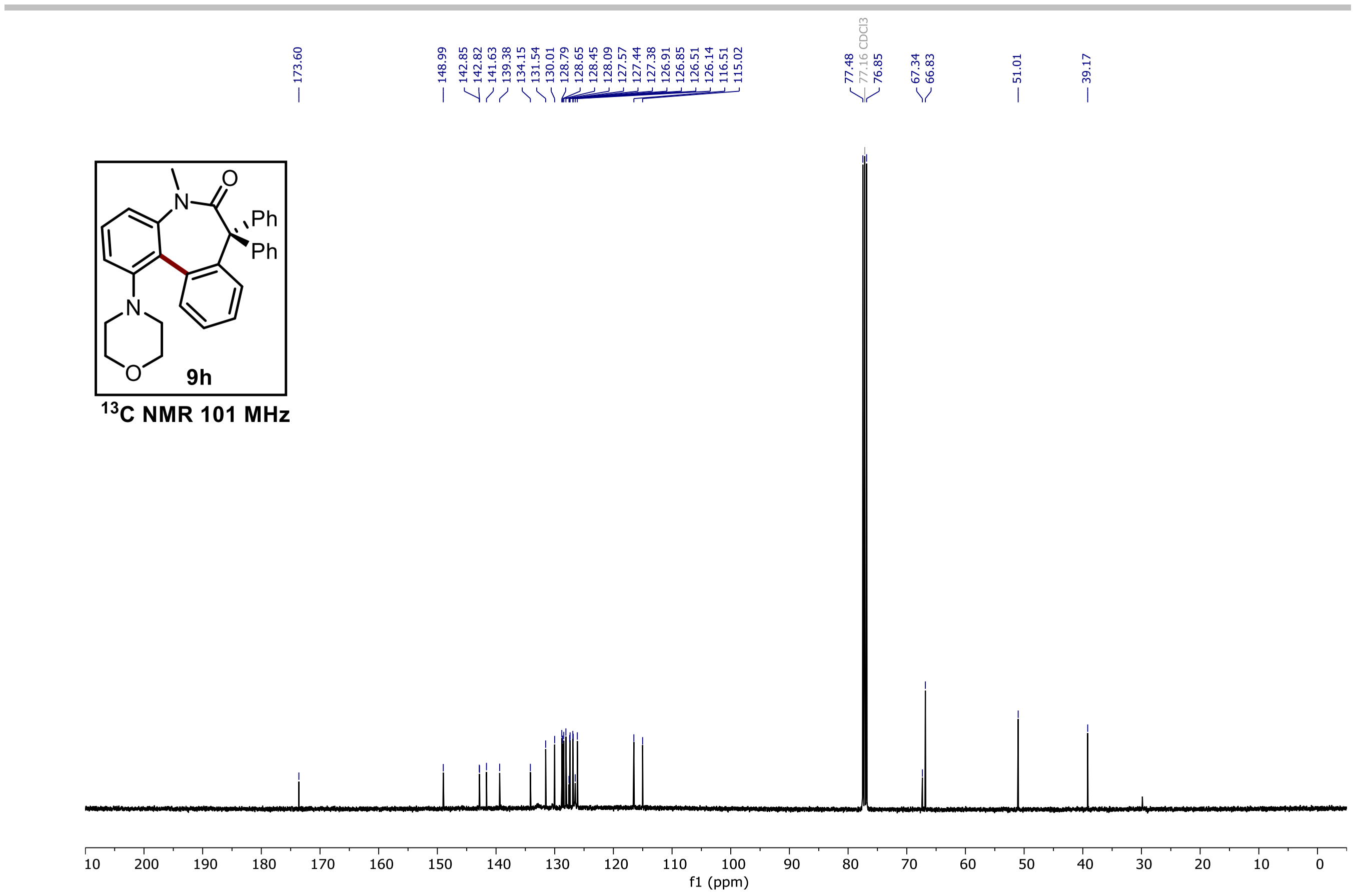
173.60
148.99
142.85
142.82
141.63
139.38
134.15
131.54
130.01
128.79
128.65
128.45
128.09
127.57
127.44
127.38
126.91
126.85
126.51
126.14
116.51
115.02
77.48
77.16 CDCl3
76.85
67.34
66.83
51.01
39.17
N
O
Ph
Ph
N
O
9h
¹³C NMR 101 MHz
10
200
190
180
170
160
150
140
130
120
110
100
90
80
70
60
50
40
30
20
10
0
f1 (ppm)
